# The genetic mechanism of heterosis utilization in maize improvement

**DOI:** 10.1186/s13059-021-02370-7

**Published:** 2021-05-10

**Authors:** Yingjie Xiao, Shuqin Jiang, Qian Cheng, Xiaqing Wang, Jun Yan, Ruyang Zhang, Feng Qiao, Chuang Ma, Jingyun Luo, Wenqiang Li, Haijun Liu, Wenyu Yang, Wenhao Song, Yijiang Meng, Marilyn L. Warburton, Jiuran Zhao, Xiangfeng Wang, Jianbing Yan

**Affiliations:** 1 National Key Laboratory of Crop Genetic Improvement, Huazhong Agricultural University, Wuhan, 430070 China; 2 National Maize Improvement Center, Department of Crop Genomics and Bioinformatics, College of Agronomy and Biotechnology, China Agricultural University, Beijing, 100193 China; 3 Key Laboratory of Biology and Genetics Improvement of Maize in Arid Area of Northwest Region, Ministry of Agriculture, Northwest A&F University, Shaanxi, China; 4 Beijing Key Laboratory of Maize DNA Fingerprinting and Molecular Breeding, Beijing Academy of Agricultural & Forestry Sciences, Beijing, 100097 China; 5 College of Life Science, Hebei Agricultural University, Baoding, 071001 China; 6 United States Department of Agriculture-Agricultural Research Service, Corn Host Plant Resistance Research Unit, Box 9555, MS 39762 Mississippi State, USA; 7 Hubei Hongshan Laboratory, Wuhan, 430070 China

**Keywords:** Maize, Heterosis, Genomic selection, Floral transition, Molecular design breeding

## Abstract

**Background:**

In maize hybrid breeding, complementary pools of parental lines with reshuffled genetic variants are established for superior hybrid performance. To comprehensively decipher the genetics of heterosis, we present a new design of multiple linked F1 populations with 42,840 F1 maize hybrids, generated by crossing a synthetic population of 1428 maternal lines with 30 elite testers from diverse genetic backgrounds and phenotyped for agronomic traits.

**Results:**

We show that, although yield heterosis is correlated with the widespread, minor-effect epistatic QTLs, it may be resulted from a few major-effect additive and dominant QTLs in early developmental stages. Floral transition is probably one critical stage for heterosis formation, in which epistatic QTLs are activated by paternal contributions of alleles that counteract the recessive, deleterious maternal alleles. These deleterious alleles, while rare, epistatically repress other favorable QTLs. We demonstrate this with one example, showing that *Brachytic2* represses the *Ubiquitin3* locus in the maternal lines; in hybrids, the paternal allele alleviates this repression, which in turn recovers the height of the plant and enhances the weight of the ear. Finally, we propose a molecular design breeding by manipulating key genes underlying the transition from vegetative-to-reproductive growth.

**Conclusion:**

The new population design is used to dissect the genetic basis of heterosis which accelerates maize molecular design breeding by diminishing deleterious epistatic interactions.

**Supplementary Information:**

The online version contains supplementary material available at 10.1186/s13059-021-02370-7.

## Background

Crop breeding began with the initial domestication of wild ancestors to generate phenotypes suited for human use [1]. Dozens of domestication-related genes have been characterized and contribute to our understanding of the genetic basis of crop domestication [2, 3]. DNA sequences targeted during domestication events exhibit significantly reduced nucleotide diversity due to artificial selection, and beneficial variants are now mostly fixed in landraces and modern germplasm [4]. Crop improvement involves the selection of additional sets of genes, and useful variants at these sequences also accumulated over time in improved germplasm [5, 6]. Various breeding goals and adaptation to diverse environments have caused a widely differing distribution of alleles across populations with more subtle effects on phenotypic morphology, as compared to variation in domestication-related alleles [7]. While this rich pool of potential genetic variants may further improve future crop yield, their minor effects on desirable traits complicate their identification and isolation in the analysis of small populations.

Approximately three percent of all maize genes were subjected to selection during domestication and improvement [8, 9]. The incorporation of useful alleles of these genes in breeding schemes is generally implemented via crossing between individuals to allow the accumulation and fine-tuning of phenotypic alterations that result from DNA recombination and the reshuffling of causal variants [10]. Thus, most artificial selection has essentially worked to reshape gene networks, rather than on single genes [11]. Although we generally define domestication and improvement as distinct phenomena, the genes and variants influencing each have been — and are still being — cooperatively selected and adopted to achieve trait improvement. Breeding success would benefit from a better understanding of this process, which remains elusive to date.

The improvement of quantitative traits such as flowering time, plant stature, grain yield, environmental adaptation, and biotic and abiotic stress resistance depends on the selection of biological interactions between multiple genes (polygenic interactions) [12]. To achieve the desired goal for a target trait, breeders develop populations by crossing a panel of breeding materials to generate novel combinations of favorable alleles and diversified polygenic interactions that can be selected for optimal traits [13, 14]. Most genes encoding polygenic traits contribute subtle effects to the overall quantitative trait expression, as the reshaped gene-interaction networks involve dozens to hundreds of genes. For this reason, genotype-to-phenotype (G2P) prediction or genomic selection (GS) models using whole-genome variations have been effective solutions to predict hybrid performance for plant breeding [15–19].

Maize (*Zea mays*) was one of the earliest crops to benefit from the power of heterosis by breeding filial one (F_1_) hybrids exhibiting superior vigor for plant growth and grain yield. The mystery of heterosis has been explored for over a century, but the underlying mechanism remains insufficiently understood [20]. One of the hypotheses for heterosis is the “dominance” model proposing that hybrid vigor of F_1_s is the result of dominance complementation of many recessive, slightly deleterious alleles at different loci in the parental genomes [21, 22]. This hypothesis was further validated by Yang et al., in which genome-wide identification of deleterious mutations were identified and proved that dominance complementation of deleterious alleles contributed to the formation of heterosis [23]. The second hypothesis for heterosis is overdominance, that the heterozygosity at individual locus causes the superior phenotype compared to either homozygous states [24]. There are several genes supporting overdominance in crops [13, 25, 26]. The development of molecular marker and next-generation sequencing (NGS) technologies has allowed large genomic-scale mapping studies in all major crops. These analyses, based on segregating populations very often derived only from two parents, have empowered the dissection of the genetic architecture of heterosis, mostly focusing on grain yield [13, 27–32].

The occurrence and strength of heterosis varies greatly, depending on the germplasm origins of the parental lines; thus, the genetic diversity of a population created with only two parents will never be sufficient to identify all heterotic quantitative trait loci (QTLs). Additional limitations of bi-parental populations further restrict the effective detection of epistatic QTLs. For example, each segregating F_2_ population must be very large to ensure sufficient statistical power. Furthermore, any two interacting loci involved in heterosis must be segregating in the F_2_ population, or epistasis will not be detected. These two limitations are the major reasons why many previous studies of heterosis underestimated the role that epistasis plays [28, 33].

Here, we present a new genetic design that overcomes these limitations and analyzes multiple linked F_1_ populations. It was created by crossing inbred lines, developed as a synthetic population, with inbred lines typical of diverse heterotic groups from around the world. This design may allow the comprehensive dissection of heterotic QTLs and associated effects. Identification of heterosis-determining genes may refine our understanding of the mechanism behind heterosis formation. This new mechanistic knowledge may in turn accelerate the process of creating and fixing new heterotic patterns between different pools of germplasm, reducing genetic vulnerability and ultimately enhancing yield in maize improvement.

**Fig. 1:**
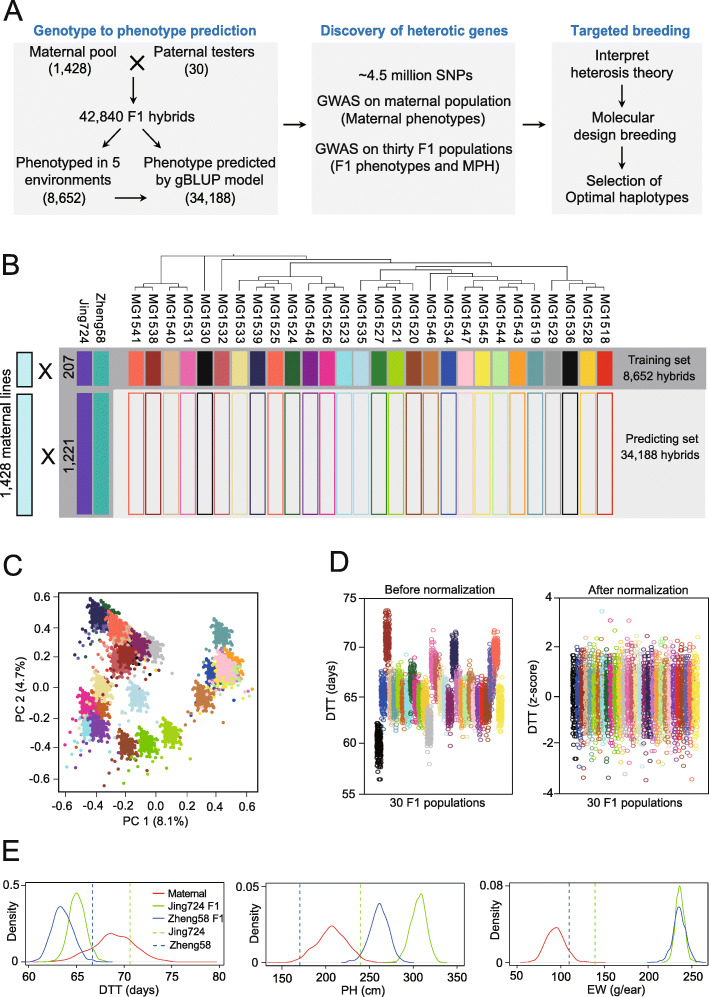
Genetic design of the thirty F_1_ hybrid populations. **a** Flowchart illustrating the integration of genotype to phenotype (G2P), genome-wide association studies (GWAS), and molecular design breeding (MDB) to achieve big data-assisted targeted breeding. **b** Schematic illustration of the North Carolina-II design used to generate the 42,840 F_1_ combinations by crossing 1428 maternal lines with 30 paternal testers. The training set is composed of 8652 hybrids with field-measured phenotypes (dark gray background). The remaining 34,188 hybrids constitute the candidate set whose phenotypes were predicted with the G2P model. **c** Principle component analysis (PCA) diagram of the thirty F_1_ hybrid populations showing strong population stratification. **d** Effect of phenotypic stratification across the thirty F_1_ hybrid populations, exemplified here with days to tasseling (DTT), before (left) and after (right) normalizing absolute trait values to *z*-scores within each F_1_ population. **e** Strong heterosis performance of the Zheng58 and Jing724 F_1_ hybrids compared to their parental inbred lines for the three traits under study

We constructed 30 F_1_ populations by crossing 1428 previously reported inbred lines from the CUBIC (Complete-diallel plus Unbalanced Breeding-derived Inter-Cross) synthetic population as a maternal pool [34] with 30 paternal testers from diverse heterotic groups. We performed genome-wide association studies (GWAS) on all populations to identify heterosis and trait-associated genes involved in maize improvement. Interrogation of the 42,840 F_1_ combinations uncovered the critical roles played by polygenic interactions and provided the framework to propose a theoretical model of the gene-regulation networks at work during floral transition. Based on this model, we used targeted genes and their associated effective variants to demonstrate the successful implementation of molecular design breeding (MDB) to facilitate selection of optimal genotypic combinations to fine-tune desired phenotypes. With the integration of G2P, GWAS and MDB on an actual breeding population, our work presents an exemplary solution to apply big data-driven decision-making strategies to target breeding for crop improvement (Fig. 1a).

## Results

### Superior heterosis performance of the thirty F_1_ populations

We employed the North Carolina II (NC-II) design to construct thirty inter-related F_1_ populations by crossing 1428 maternal lines from the CUBIC population [34] with thirty paternal tester lines (Additional file 1). While the maternal pool represents locally adapted alleles for China, the paternal testers encompass a broad range of advantageous alleles from improved foreign germplasm to maximize genetic complementation in the F_1_ hybrids (see the “Methods” section). Setting up all crosses between the 1428 and 30 parental lines would generate 42,840 F_1_ combinations but would also constitute a major phenotyping bottleneck by obtaining trait data in the field. Considering the clear pedigree of the CUBIC lines, we hypothesized that G2P prediction may be performed to infer missing F_1_ phenotypes, making it unnecessary to plant all F_1_ populations. We planted a training dataset consisting of 8652 F_1_ hybrids in five locations and measured three major traits—days to tasseling (DTT), plant height (PH), and ear weight (EW)—on 17 individual plants for each hybrid. We also used the commercial hybrid ZhengDan958 as a control and planted it once for every 50 rows to adjust for phenotypic heterogeneity across fields. We then used the measured phenotypes from the 8652 F_1_s to train the G2P model and predict the phenotypes of the remaining 34,188 F_1_ hybrids.

To ensure full coverage of all paternal and maternal genotypes, the training set included two complete F_1_ populations consisting of 2856 hybrids between the 1428 maternal lines and the two paternal tester lines Zheng58 and Jing724, which have been widely used in Chinese commercial breeding programs. The remaining 28 F_1_ populations were represented by 5796 F_1_ hybrids between a subset of 207 maternal lines and the other 28 paternal testers (Fig. 1b). Phylogenetic analysis of the 1458 maternal and paternal parents showed an even distribution of the 207 maternal training lines, interspersed with the 1221 predicted maternal lines, illustrating the unbiased representation of the training set (Additional file 2: Figure S1a). We recreated the genotypes for all 42,840 F_1_ combinations from the over 4.5 million high-quality single nucleotide polymorphisms (SNPs) obtained from whole-genome resequencing of the 1458 parental lines (see the “Methods” section). Principle component analysis (PCA) based on the F_1_ genotypes showed stratified clustering patterns, suggesting that population structure is a critical factor to consider in both G2P predictions and GWAS analyses (Fig. 1c). We also observed paternally oriented stratification in F_1_ phenotypes (Fig. 1d, left panel). We transformed the absolute phenotypic values to relative rankings using the *z*-score algorithm (Fig. 1d, right panel; Additional file 2: Figure S1b; see the “Methods” section).

A comparison of parental and F_1_ phenotypes revealed heterosis for the three traits across the thirty F_1_ populations. For DTT, 28 F_1_ populations exhibited earlier flowering times than their corresponding parents, with the exception of the two tropical testers, indicating that heterosis for DTT is expressed as early flowering (Fig. 1e; Additional file 2: Figure S2). In the case of the PH and EW traits, all F_1_ phenotypic values were two to three times higher than the parental lines and exhibited strong mid-parent heterosis (MPH). Among the 8652 F_1_ hybrids with measured phenotypes, 554 hybrids (or 6.4%) showed earlier DTT, shorter PH, and higher EW when compared to the commercial control variety ZhengDan958, illustrating the potential of our F_1_ hybrid population for the breeding of early-flowering, high-yielding, and compact cultivars.

### G2P prediction enhances GWAS detection power

**Fig. 2:**
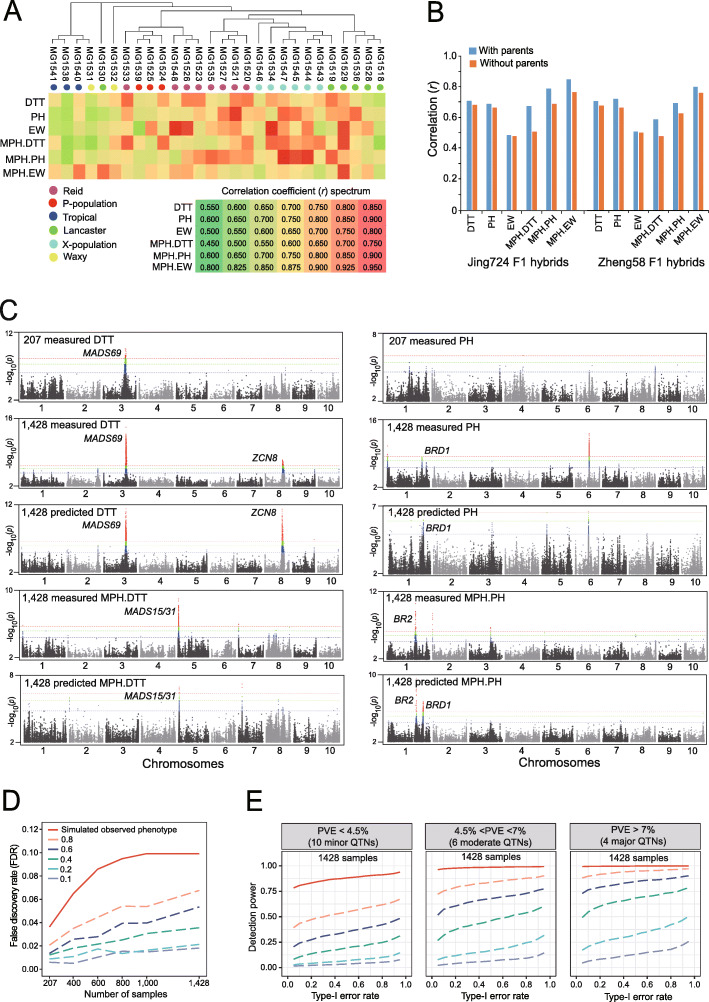
Genotype-to-phenotype (G2P) prediction enhances GWAS power. **a** Evaluation of the G2P precision using the first prediction scheme of 207 × 27 hybrids as training samples versus the remaining 207 hybrids as testing samples. The red, yellow, and green color scale represents the correlation coefficient (*r*) between measured and predicted phenotypes from high, moderate, to low *r* values, respectively. **b** Evaluation of G2P precision using the second prediction scheme in which 207 × 30 + 1221 Zheng58 F_1_ hybrids were used as the training set to predict 1221 Jing724 F_1_ hybrids (left panel), and vice versa (right panel). The accuracy of G2P prediction when including parental phenotypes as fixed effects to correct for population stratification was higher than G2P prediction without parental phenotypes. **c** Comparison of GWAS signals, illustrated as Manhattan plots, in the Zheng58 F_1_ population for DTT and mid-parent heterosis of DTT (MPH.DTT; left panel), plant height (PH), and mid-parent heterosis of PH (MPH.PH; right panel) using 207 (“207 measured”) and 1428 samples with measured phenotypes (“1,428 measured”), and 1428 samples with 207 measured plus 1221 predicted phenotypes (“1,428 predicted”). **d** False discovery rates (FDRs) of GWAS detection of the 20 spike-in QTNs under the circumstances of different prediction accuracy and population size. The GWAS detection for false positives is declared at the significant threshold of *p*-value ≤ 1e− 05, based on adjusted Bonferroni correction. **e** Detection power of the 20 spike-in QTNs with major, moderate, and minor effects by GWAS in the population of 1428 samples using the simulated phenotypes at the six levels of prediction accuracy. The GWAS detection of true positives is declared by a significant threshold based on a series of type I errors (*α*) from 0.05 to 0.95

We used the genomic best linear unbiased prediction (gBLUP) model to infer the phenotypes of the 34,188 F_1_ combinations we did not phenotype in the field (see the “Methods” section). Because the parental phenotypes exhibited correlations with F_1_ phenotypes (Additional file 2: Figure S3), they were used as fixed effects in gBLUP to reduce the influence stemming from population stratification. We evaluated the gBLUP model by partitioning the training and testing samples in two ways. First, to evaluate the influence of population stratification, we used 27 of the 28 F_1_ hybrid populations (corresponding to 5589 F_1_ samples, or 207 maternal lines × 27 parental lines) for training purposes, while we used the remaining 207 samples (207 maternal lines × 1 missing parental line) for testing. We repeated this procedure 28 times to derive predictability, measured as the Pearson correlation coefficient (*r*) between measured and predicted phenotypes for each F_1_ population. The average predictabilities of the 28 F_1_ hybrid populations for the three traits were 0.76 (DTT), 0.81 (PH), and 0.66 (EW); for the mid-parent heterosis, estimates were 0.61 (MPH.DTT), 0.805 (MPH.PH), and 0.89 (MPH.EW) (Fig. 2a; Additional file 3). The predictabilities across the 28 sets varied as a function of their heterotic groups, with the lowest predictabilities seen for the tropical and waxy groups, and the highest predictabilities for the X-population and Reid groups (Fig. 2a).

The second model testing method was designed to evaluate the predictability of the Zheng58 and Jing724 hybrids. We used the 6210 (207 maternal lines × 30 paternal lines) hybrids plus the 1221 Zheng58 hybrids as the training model to predict the performance of the 1221 Jing724 hybrids. We then repeated the same exercise, this time using the Jing724 hybrids alongside the 6210 hybrids in a new training set to predict the 1221 Zheng58 hybrids. When parental phenotypes were considered as fixed effects in the gBLUP model, predictabilities were 0.695 (DTT), 0.67 (PH), and 0.44 (EW) for Jing724 hybrids and 0.70 (DTT), 0.72 (PH), and 0.47 (EW) for Zheng58 hybrids (Fig. 2b).

The complete determination of 42,840 F_1_ phenotypes (both measured and inferred) enlarged the effective population dataset fivefold, which may greatly enhance GWAS detection power. Indeed, GWAS signals significantly rose in GWAS analyses performed on the full dataset, which includes all 1428 maternal lines across the thirty F_1_ populations, when compared to the training set of 207 maternal lines (Additional file 4). As illustrated by the Zheng58 F_1_ population, GWAS for DTT on the 207 hybrid subset only detected a peak on chromosome 3 at the *MADS69* locus, but GWAS with all 1428 hybrids (combining measured DTT from 207 F_1_s and predicted DTT from 1221 F_1_s) detected an additional peak on chromosome 8 at the *ZEA CENTRORADIALIS 8* (*ZCN8*) locus (Fig. 2c). The *MADS69*-*RAP2*.*7*-*ZCN8* pathway is the core flowering activation module in maize [35]: the detection of both *MADS69* and *ZCN8* peaks confirms the increased GWAS power in the larger hybrid set. The GWAS signal of the *MADS69* peak using 1428 predicted DTT values (*p*-value = 1e− 12) was weaker than when using measured DTT values (*p*-value = 1e− 16) on the same samples, but was nevertheless one order of magnitude higher than that obtained with the subset of 207 samples. GWAS on both measured and predicted MPH.DTT detected a peak at the *MADS15*/*31* locus, a pair of tandemly duplicated MADS-box transcription factor genes.

We detected an unknown PH QTL on chromosome 6 in GWAS of the combined 1428 hybrids for both measured and predicted PH, although this peak was absent in the subset of 207 hybrids. We detected a peak at the *Brachytic2* (*BR2*) [36] locus in the full 1428 hybrid set for both measured and predicted MPH.PH. However, a peak at the start of chromosome 2 (peak SNP: chr2.s_537241) was only detected in the full 1428 hybrid set when using measured MPH.PH values and was absent when using predicted MPH.PH values, most likely reflecting the extremely low minor allele frequency (MAF = 0.023) of the peak SNP among the 1428 hybrids. These results demonstrate that G2P prediction is an effective strategy that greatly reduces field labor and phenotyping expense without sacrificing GWAS power.

### Quantitative evaluation of GWAS accuracy with simulation analysis

When using predicted phenotypes for GWAS in the enlarged population containing 1428 samples, the risk of false discovery of excessive unrelated QTLs must be excluded. To quantitatively evaluate the GWAS accuracy, we performed a series of simulation analyses to infer the false discovery rate (FDR) and detection power (DP) under 36 circumstances of different prediction accuracy and population size. These analyses were based on simulating a polygenic trait determined by 20 spike-in QTNs (quantitative trait nucleotides) that contribute major, moderate, and minor effects to the phenotype (see the “Methods” section).

We first evaluated whether predicted phenotypes may cause excessive false discovery of QTNs that do not belong to the 20 spike-in QTNs, by defining the FDR using the equation $$ FDR=\raisebox{1ex}{${N}_1$}\!\left/ \!\raisebox{-1ex}{$\left({N}_1+{N}_0\right)$}\right. $$ where *N*_1_ is the number of false QTNs and *N*_0_ is the number of spike-in QTNs. When directly using the simulated observed phenotypes for GWAS without considering prediction error (*r*^2^ = 1.0), FDR still existed and increased with the increment of population size (Fig. 2d). When different levels of prediction errors (*r*^2^ = 0.8, 0.6, 0.4, 0.2, 0.1) were considered, FDR was not increased, rather decreased along with the decreased prediction accuracy. This pattern indicated that discovery of false-positive QTNs may always occur even if observed phenotypes were used for GWAS; on the contrary, inaccuracy in the predicted phenotypes may not cause the discovery of more false-positive QTNs compared to the use of observed phenotypes for GWAS. The possible explanation for this scenario is that inaccuracy in the predicted phenotypes may cause a dramatic decrease of the number of significant SNPs, which lowered the total amount of detected QTNs (Additional file 2: Figure S4). Thus, we may conclude that inaccuracy in the predicted phenotypes will not introduce excessive false-positive QTNs.

We then evaluated the influence of the phenotype prediction accuracy on the detection power (DP) for GWAS. In this analysis, we used a permutation-based method to define a significance threshold (*α* = 0.05) to detect spike-in QTNs by shuffling the simulated observed phenotypes for 1000 times. The DP was defined as the proportion of successful detections out of the 500 times of GWAS for each QTN, and the averaged DP of all the 20 spike-in QTNs was computed to represent the overall DP under each circumstance of prediction accuracy and population size. DP of GWAS was determined by all of the three factors including population size, QTN effects and prediction accuracy (Additional file 2: Figure S5). When the type-I error rate was controlled at *α* = 0.05 and the accuracy of predicted phenotype reached *r*^2^ = 0.8, major and moderate QTNs had about 87.5 and 75.0% probability to be detected in the population containing all of the 1428 samples, respectively; even though the accuracy dropped to *r*^2^ = 0.6, the probabilities of detecting major and moderate QTNs were still about 75.0 and 50.0% respectively (Fig. 2e). When only 207 samples were used for GWAS, we only had about 25.0% probability to discover major and moderate QTNs.

Therefore, the above simulation analysis indicated that detection power of GWAS can be greatly improved due to enlarged population size, since genomic variants with small genetic effects are mostly missed in the small population of 207 samples. At the same time, utilization of prediction phenotypes with relatively high prediction accuracy may not cause excessive false discovery of QTLs unrelated to a studied trait, such as the DTT and PH traits with high heritability determined by a small number of major-effect QTLs. This conclusion can be further confirmed using an alternative way of simulation analysis (Additional file 2: Figure S6; see the “Methods” section).

### Identification of heterotic QTLs across thirty F_1_ populations

**Fig. 3:**
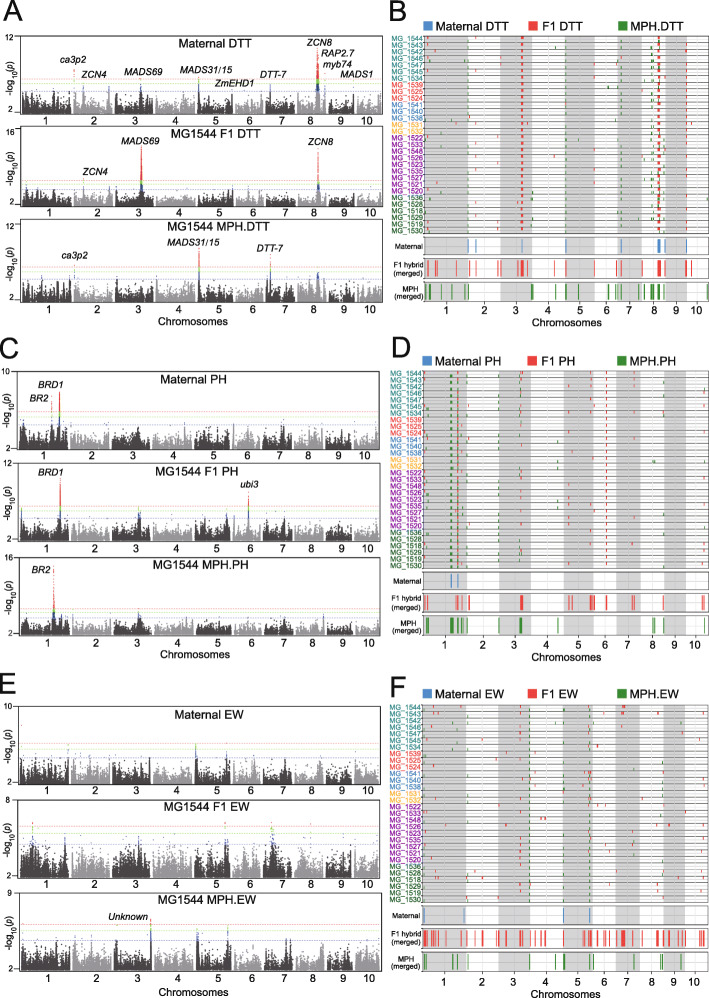
Detection of heterotic QTLs by GWAS across thirty F_1_ populations. **a** Comparison of the GWAS signals, illustrated as Manhattan plots, for DTT in the maternal population, and for DTT and mid-parent heterosis of DTT (MPH.DTT) in the MG1544 F_1_ population. The three dashed lines colored in red, green, and blue represent three significance thresholds with *p*-value = 2.2e− 07, 1e− 06, and 1e− 05, respectively. **b** Summary of QTLs for DTT (red) and MPH.DTT (green) detected in each F_1_ population and merged QTLs from the thirty F_1_ populations, compared to the QTLs in the maternal population (blue). The three dashed lines colored in red, green, and blue represent three significance thresholds with *p*-value = 2.2e− 07, 1e− 06, and 1e− 05, respectively. **c** Comparison of the GWAS signals, illustrated as Manhattan plots, for plant height (PH) and MPH.PH detected in the MG1544 F_1_ population. The three dashed lines colored in red, green, and blue represent three significance thresholds with *p*-value = 2.2e− 07, 1e− 06, and 1e− 05, respectively. **d** Summary of QTLs for PH (red) and MPH.PH (green) detected in each F_1_ population and merged QTLs across the thirty F_1_ populations, compared to the QTLs in the maternal population (blue). **e** Comparison of the GWAS signals, illustrated as Manhattan plots, for ear weight (EW) and MPH.EW detected in the MG1544 F_1_ population. The three dashed lines colored in red, green, and blue represent three significance thresholds with *p*-value = 2.2e− 07, 1e− 06, and 1e− 05, respectively. **f** Summary of QTLs for EW (red) and MPH.EW (green) across the thirty F_1_ populations, compared to the QTLs in the maternal population (blue)

Comparing the GWAS signals between maternal and F_1_ populations facilitates the dissection of heterotic QTLs and the genes associated with the three traits. As exemplified by one F_1_ population derived from crosses between all maternal lines and the MG1544 tester, we detected the *ZCN8* and *RAP2*.*7* peaks as significant associations in both maternal and F_1_ populations. However, the *MADS69* peak was vastly more significant in the F_1_ population than in the maternal population (Fig. 3a). We identified several peaks at previously cloned (names in uppercase) and putative (names in lowercase) flowering time genes in the maternal population, including *EARLY HEADING DATE 1* (Zm*EHD1*), *MADS1*, *MADS15/31*, *ZCN4*, *CCAAT*-*HAP3*-*transcription factor 32* (*ca3p2*, also called Zm*NF*-*YB3*), and *myb74*. Several genes showed weaker GWAS peaks when using the F_1_ phenotypes, for example, *ca3p2*, *MADS15/31*, and *DTT*-*7* (an unknown peak on chromosome 7), although they rose above the significance threshold when using MPH.DTT for GWAS (Fig. 3a, bottom panel).

We detected 8 QTLs for DTT in the maternal population, 24 QTLs in the F_1_ hybrid population, and 23 QTLs when using the mid-parent heterosis values from the F_1_ hybrid population (Additional file 5). The eight major flowering time QTLs identified in the maternal population were all detected using either DTT or MPH.DTT values in the F_1_ population as well (Fig. 3b; see the “Methods” section). Moreover, the 24 DTT QTLs and 23 MPH.DTT QTLs are mostly non-overlapping, with the exception of the wide peak at *ZCN8* and *RAP2*.*7* (which we will refer to as *ZCN8*/*RAP2*.*7*). Of the 47 QTLs (24 for DTT and 23 for MPH.DTT) in F_1_ hybrids, we detected *MADS69* and *ZCN8*/*RAP2*.*7* across all thirty populations, while the other QTLs peaks at the *MADS15/31*, *ca3p2*, *MADS1*, *DTT*-*7*, *myb74*, and *ZCN4* loci were detected 29, 23, 22, 21, 9, and 8 times, respectively, with moderate signals. In a long genomic region spanning from ~ 75 to ~ 88 Mbp on chromosome 8 upstream the *ZCN8*/*RAP2*.*7* QTLs (~ 123 Mbp), we identified a series of fragmented QTLs for MPH.DTT in 22 F_1_ populations, although this region does not contain previously reported flowering time genes (Fig. 3b and Additional file 5).

GWAS for PH in the maternal population detected two significant peaks. One was associated with the *BR2* locus (peak SNP: chr1.s_201665854), the causal SNP being a rare allele derived from inbred Zong3 [36]. The other peak mapped to the *BRASSINOSTEROID*-*DEFICIENT DWARF 1* (*BRD1*) gene (peak SNP: chr1.s_248796560) [37, 38] (Fig. 3c). In the F_1_ population, we observed an additional peak on chromosome 6 (peak SNP: chr6.s_95877243), overlapping with the *ubiquitin3* (*ubi3*) gene previously mapped in GWAS studies for plant and ear height [39]. The height of the *BR2* peak associated with PH greatly diminished in the F_1_ population, but we did detect this QTL when using MPH.PH phenotypic values. The distinct GWAS signals for the *BR2* and *BRD1* genes for PH and MPH.PH suggests distinct genetic effects underlying heterosis of PH. Moreover, we identified 18 additional QTLs of moderate significance for PH and 14 for MPH.PH multiple times across the thirty F_1_ populations (Fig. 3d; Additional file 5).

Only a few major QTLs were significantly associated with EW in both maternal and F_1_ populations. We could not identify clear underlying candidate genes within these QTL regions. Three QTLs with moderate signals were detected in MPH.EW, suggestive of the strong heterosis controlling the EW trait (Fig. 3e). Relaxing our significance criteria when calling a QTL from GWAS only added four EW-related QTLs in the maternal population, but added 48 and 15 in the GWAS results for EW and MPH.EW phenotypes from all F_1_ populations, respectively (Fig. 3f; Additional file 5).

### Forms of genetic effects implied by the differential GWAS signals across maternal and F_1_ populations

**Fig. 4:**
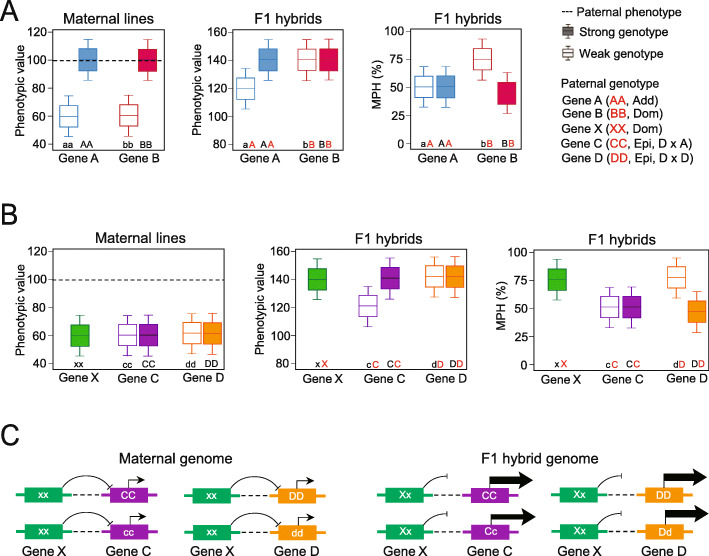
Schematic illustration of the inference of additive, dominant, and epistatic QTLs. **a** Schematic illustration of the inference of additive and dominant QTLs represented by two genes, Gene A and Gene B, respectively, based on the differential patterns of GWAS signals across maternal and F_1_ populations, and the MPH of F_1_ hybrids. Strong (uppercase letter) and weak (lowercase letter) genotypes are defined by the corresponding strong and weak phenotype of the maternal lines, respectively. The paternal genome always provides a strong allele, marked in red. **b** Schematic illustration of the inference of epistatic QTLs represented by two genes, Gene C and Gene D, that are epistatically repressed by unknown Gene X in maternal genomes. When the maternal Gene x allele is combined with the dominant X allele from the paternal genome, Gene C and Gene D are activated and exhibit additive and dominant effects, respectively. D × A and D × D represent the epistatic effects of *d*ominant by *a*dditive and *d*ominant by *d*ominant interactions, respectively. **c** A putative model for the interpretation of the regulatory interactions between Genes C and D, and Gene X. In the maternal genome, Gene X with homozygous, recessive alleles represses the transcription of Genes C and D (left panel). In the F_1_ hybrid, the dominant X allele from the paternal genome complements the recessive x allele of Gene X, and the repressive effect on Gene C and D is relieved. Then, transcription of Gene C and D is activated with different expression dosages based on their additive and dominant effects

Comparing GWAS signals across maternal and F_1_ populations and derived F_1_ MPH calculations is a straightforward way to infer genetic effects underlying the QTLs contributing to heterosis. Summing the GWAS signals across the thirty F_1_ populations identified 166 QTLs for the three traits (Additional file 6), which we categorized into three QTL classes with additive, dominant, or epistatic effects according to the scenarios (Fig. 4a, b). The first class includes QTLs (exemplified by Gene A) detected in both maternal and F_1_ populations, suggesting additive effects as the maternal and paternal alleles equally contribute to F_1_ phenotypes. The second class includes dominant QTLs (exemplified by Gene B) detected in the maternal population but not in the F_1_ populations, that are caused by dominance complementation of the paternal allele against the recessive maternal allele in F_1_ hybrids. QTLs with complete dominance effects can be also detected by GWAS on the derived F_1_ MPH values, but additive QTLs are undetectable (Fig. 4a, rightmost panel).

The third class includes QTLs with effects that differ from the expected action from a single gene, as exemplified by Genes C and D (Fig. 4b). Gene C has an additive effect, and gene D has a dominant effect, but these are undetectable in the maternal population and only detected in the F_1_ populations. The unexpected behavior of this class of QTLs in the maternal population is most likely epistatically repressed by another unidentified QTL, as exemplified by the Gene X executing recessive, repressive effects on Genes C and D (Fig. 4b). Gene X is undetectable by GWAS in the maternal population due to extremely low frequency (MAF << 0.05) or absence of the activating allele, when the population size is insufficient to detect allele segregation. In F_1_ hybrids, the activating allele contributed from the paternal genome dominantly complements the maternal allele of Gene X to release its repressive effect, thus resulting in detection of Genes C and D. Then, Genes C and D can be classified as epistatically controlled QTLs (epistatic QTL). Therefore, attributed to the unique design of the half-sibling F_1_ population in the present study, one of the genetic mechanisms of heterosis utilization is explained for single-cross breeding in modern maize industry. Although an advantageous QTL normally segregates in one heterotic group, the beneficial effect of the QTL is epistatically controlled by another QTL; this deleterious epistasis is diminished by the beneficial alleles contributed from another heterotic group, therefore resulting the expression of significant heterosis observed from either F_1_ phenotypes or MPH values (Fig. 4b, middle and rightmost panel).

Although various direct or indirect molecular interactions may underlie the observed epistasis, one possible mechanism may involve transcriptional regulation that may cause changes in gene expression dosage, assuming that Gene X imposes a strong repressive effect on Genes C and D (Fig. 4c). While transcription of Genes C and D is epistatically repressed by Gene X in the maternal genome, alleviation of the repressive effect of Gene X in the hybrid genome activates Genes C and D. This change in the molecular network in F_1_ hybrids results in the detection of Gene C (additive effect) in the F_1_ population but not when using the derived MPH phenotypic values, while Gene D (dominant effect) is undetectable in the F_1_ population but is detected when using the derived MPH values (Fig. 4c).

### Epistatic interactions between QTLs contributed to heterosis of PH and EW

**Fig. 5:**
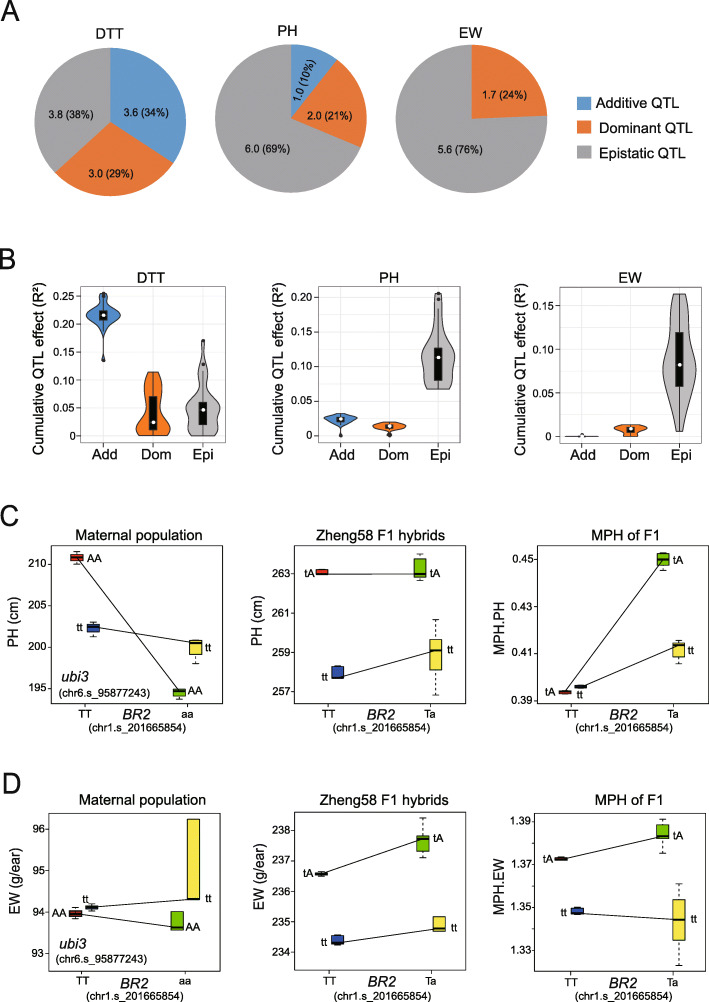
Epistatic Interactions Contributing to the Heterosis of PH and EW. **a** Proportions of additive, dominant, and epistatic QTLs detected by GWAS for the three traits. The number of QTLs for each trait were averaged over the thirty F_1_ populations. **b** Different contributions of additive (Add), dominant (Dom), and epistatic (Epi) effects to DTT, PH, and EW. We computed the cumulative QTL effects for the three classes of QTLs based on the significant QTLs detected in each of the thirty F_1_ populations. **c** An epistatic interaction between the *BR2* and *ubi3* QTLs contributes to the heterotic performance of PH in F_1_ hybrids. In the maternal population (left panel), the *ubi3*-AA genotype exhibited the shortest PH in the *BR2*-aa background (green boxes), indicating that *BR2*-aa suppresses *ubi3*-AA. In the Zheng58 (*BR2*-TT and *ubi3*-tt in Zheng58) F_1_ population (middle panel), the *ubi3*-tA genotype exhibited the tallest PH in the *BR2*-Ta background (green boxes), indicating that the suppression of *ubi3* by *BR2* is relieved due to the complementation of the recessive *BR2*-t allele by the dominant *BR2*-T allele in F_1_ hybrids. The superior heterotic performance is also reflected in the MPH of PH (right panel). Uppercase bases represent dominant alleles and lowercase bases represent recessive alleles. **d** Epistatic interaction between *BR2* and *ubi3* QTLs contributes to the heterotic performance of EW in F_1_ hybrids

Across the 166 heterosis-related QTLs detected across the thirty F_1_ populations, the three forms of genetic effects exhibited different proportions of additive (5.4%), dominant (5.4%), and epistatic (89.2%) QTLs contributing to DTT, PH, and EW. In addition, these proportions differed in each F_1_ population. For example, while DTT showed relatively equal numbers of the three QTL classes, epistatic QTLs contributed much more to PH and EW (Fig. 5a). Additive QTLs drove most of the variation in DTT when compared to dominant and epistatic QTLs. By contrast, epistatic QTL contributed most to PH and EW and accounted for the highest contribution to F_1_ heterosis for these traits (Fig. 5b), which is consistent with the observation that EW experienced the strongest heterosis and DTT the lowest heterosis.

The high proportion of epistatic effects contributing to PH and EW may be attributable to QTL interactions, as exemplified by the genetic interaction detected between the dominant QTL *BR2* on chromosome 1 and the epistatic QTL *ubi3* on chromosome 6 (Fig. 3c). We hypothesized this epistatic interaction agreed with the scenario illustrated in Fig. 4b, since *ubi3* was only detected in the F_1_ population but not in the maternal population. Genetic interaction analysis between the *BR2* and *ubi3* loci indicated that the most likely scenario called upon the homozygous *ubi3*-AA genotypes as being repressed by the recessive *BR2*-aa genotypes in the maternal population. The resulting repression prevented the detection of the *ubi3* QTL by GWAS in the maternal population. In F_1_ hybrids, however, the recessive *BR2*-a allele is complemented by the dominant *BR2*-T allele from the paternal genome, removing the epistatic repression and thus allowing the *ubi3*-T allele to fully express the PH trait (Fig. 5c).

Furthermore, the 32 lines carrying the AA genotypes at both *BR2* and *ubi3* exhibited the lowest EWs in the maternal population, but their EWs significantly increased to become the best performing genotypic group in the Zheng58 F_1_ population (Fig. 5d). Thus, although *ubi3*-AA is the favorable genotype that positively contributes to EW, its beneficial effect is repressed by the unfavorable, recessive *BR2*-aa genotype. In the F_1_ hybrids, the introduction of the favorable, dominant *BR2*-T allele from the paternal genome complements the deleterious effects of the *BR2*-a allele to activate the beneficial effects of the *ubi3*-A alleles. This combination of alleles results in the expression of superior heterosis for EW in the F_1_ hybrids. It is worth noting that we detected the *BR2* and *ubi3* QTLs across all thirty F_1_ populations, indicating that the epistatic interaction between the *BR2* and *ubi3* QTLs is a common mechanism for maize heterosis for this trait, warranting further investigation (Fig. 3d).

### Genetic inference of polygenic interactions during floral transition

Photoperiodic adaptation to different environments involves the precise timing of the vegetative-to-reproductive transition to achieve stable yield production under each day-length regime [40]. Genes initiating floral transition may communicate with those terminating vegetative growth, resulting in a balance between flowering time and plant height, as early-flowering results in shorter plants while delayed flowering allows further increases in height due to prolonged vegetative growth [41]. *BRD1*, encoding a brassinosteroid C-6 oxidase [38], was detected by GWAS as a major-effect QTL for PH and offered us a good target to understand the underlying regulatory networks (Fig. 3c). To study the effects of *BRD1* within these networks, we divided the 1428 maternal lines into two groups according to their genotype at the tag SNP (chr1.s_248796560) associated with the *BRD1* peak. The tall PH group (215.3 ± 20.2 cm) included 548 lines bearing the chr1.s_248796560-CC genotype (*BRD1*-CC) and the short PH group (207.5 ± 20.0 cm) included 834 lines with the chr1.s_248796560-TT genotype (*BRD1*-TT).

**Fig. 6:**
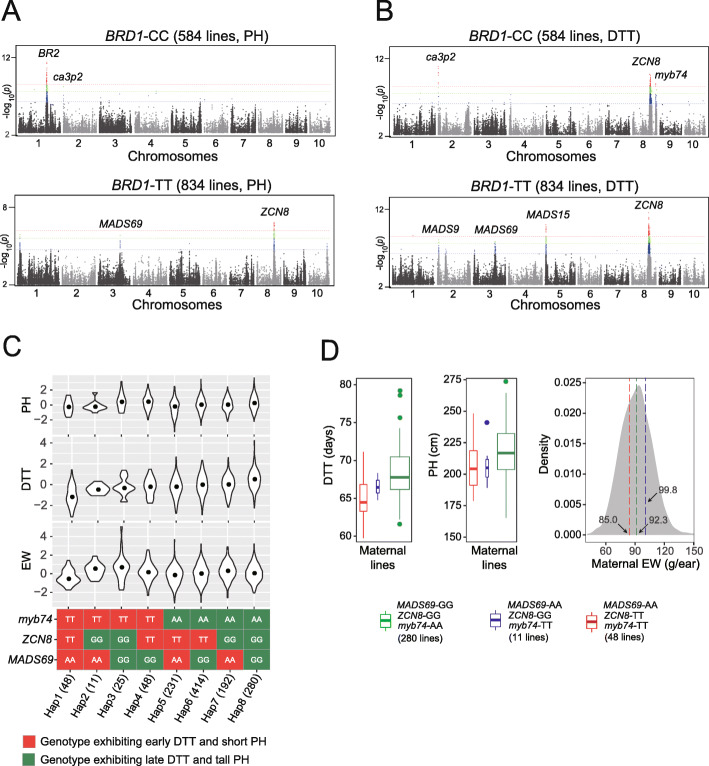
Polygenic interactions mediating floral transition inferred by conditional GWAS. **a**, **b** Manhattan plots for GWAS of PH (**a**) and DTT (**b**) run separately as two subgroups of 584 maternal lines (*BRD1*-CC, top panels) and 834 maternal lines (*BRD1*-TT, bottom panels), divided according to the genotype for the peak SNP of chr1.s_248796560 in the *BRD1* QTL. The three dashed lines colored in red, green, and blue represent three significance thresholds with *p*-value = 2.2e− 07, 1e− 06, and 1e− 05, respectively. **c** Selection of the optimal genotypic combinations, namely Haplotype 1 (Hap1) and Haplotype 2 (Hap2) of the genes *MADS69*, *ZCN8*, and *myb74* that favor early DTT and short PH without affecting EW. The 11 Hap2-bearing maternal lines exhibit early DTT, reduced PH and normal EW, and thus are determined to be the optimal haplotype combination. **d** Comparison of the DTT, PH, and EW phenotypes of the 11 maternal lines carrying the optimal haplotype (Hap2, blue) with the phenotypes of 48 Hap1 (red) and 280 Hap8 lines (green)

We performed GWAS for PH separately on the *BRD1*-CC and *BRD1*-TT groups (referred to as conditional GWAS in the following text). Without the overwhelming masking effect of *BRD1*, we detected additional peaks within the two groups, including peaks at the *BR2* and *ca3p2* loci in the tall PH group, and peaks at the *MADS69* and *ZCN8* loci in the short PH group (Fig. 6a). GWAS for DTT using the same two groups also exhibited differential signals: we detected peaks for the *ca3p2*, *ZCN8*, and *myb74* loci in the tall PH group, and a different set of peaks in the short PH group mapping to the *MADS9*, *MADS69*, *MADS15*, and *ZCN8* loci (Fig. 6b). To further infer the genotypic interactions between *BRD1* and the *BR2*, *ca3p2*, *MADS69*, and *ZCN8* genes, we further subdivided the *BRD1*-CC and *BRD1*-TT groups into subgroups according to their genotypes at the tag SNPs associated with the four genes (subdivided one at a time) to compare their PH and DTT phenotypes. We observed different DTT and PH phenotypes in the subgroups for each of the four genes in the different *BRD1* backgrounds, suggesting that *BRD1* may genetically interact with these (and potentially other) genes to affect PH and DTT (Additional file 2: Figure S7).

The fact that we identified different sets of DTT- and PH-related genes by conditional GWAS in contrasting *BRD1* genotypic backgrounds suggest complex polygenic interactions during the transition from vegetative growth to the reproductive phase. The different phenotypes observed with the various allelic combinations at these genes also strongly suggest that gene-regulation networks are being reshaped. Successful MDB thus becomes possible, whereby coordinated selection of favorable genotypes will generate optimal haplotype combinations to achieve the desired, balanced status of multiple target traits. In the current study, we set our primary breeding goal as the selection of compact maize cultivars suited for mechanical harvesting. Thus, we targeted parental lines carrying genotypes favorable for short PH and early DTT, without reducing EW, for selection with molecular design at the abovementioned genes. Among the three flowering time genes exhibiting differential GWAS signals in the contrasting *BRD1* genotypic backgrounds, the early-flowering genotypes *ZCN8*-TT, *MADS69*-AA and *myb74*-TT exhibited different effects on PH and EW (Additional file 2: Figure S7). While *ZCN8*-TT caused short PH and lighter EW, *MADS69*-AA caused short PH without significantly affecting EW (*p*-value = 0.369). Since *myb74*-TT caused significantly earlier DTT (*p*-value = 2.4e− 11) without affecting PH or EW, we also included this gene as an additional target for MDB.

When considering the four genes in our MDB trial, the two *BRD1* genotypes showed no discernible differences in PH for any of the early DTT genotypes of the three flowering time genes (Additional file 2: Figure S8). We therefore excluded the *BRD1* genotype in the current MDB trial. Although the combination of the early DTT genotypes at the *ZCN8*, *MADS69*, and *myb74* loci (denoted as Hap1, short for Hapotype1) showed the earliest DTT and shortest PH, EW also dropped to the lowest weight compared to other combinations, probably due to a strong negative effect of *ZCN8*-TT on EW (*p*-value = 4.2e− 7) (Fig. 6c; Additional file 2: Figure S9). Thus, to balance DTT, PH, and EW, the optimal combination for the three flowering time genes is *ZCN8*-GG, *MADS69*-AA, and *myb74*-TT (referred to as Hap2). Of all maternal lines, only 11 shared these alleles, but they displayed intermediate DTT, reduced PH and, importantly, unaffected EW (Fig. 6d), as designed. In addition, their F_1_ hybrids with Zheng58 and Jing724 exhibited a similar trend of earlier flowering time and reduced plant stature without affecting ear weight (Additional file 2: Figure S10).

## Discussion

Maize was among the first domesticated crops for which heterosis was exploited for breeding [42] and is an ideal model plant for studying heterosis [20, 22]. In this study, we analyzed the genotypes and phenotypes of 42,820 F_1_ hybrids derived from thirty F_1_ populations. We created these hybrids by crossing the CUBIC lines developed from indigenous Chinese HZS germplasm with thirty diverse elite tester lines of various genetic backgrounds. Such a large and varied dataset presents a valuable genetic resource for identifying heterosis-related genes and QTLs and facilitates a dissection of the contribution that heterosis has brought to maize improvement in the modern seed industry. We also illustrate the integrative use of G2P and MDB to implement big data-assisted decision-making in maize breeding. With a set goal of breeding compact maize cultivars suited for mechanical harvesting, we used MDB to select optimal genotypic combinations favoring short, early-flowering plants without sacrificing yield. The successful implementation of MDB is based on an assumed pathway involving polygenic interactions for floral transition that balances flowering time and plant stature. This assumption is supported by the understanding that crop improvement via artificial selection has reshaped gene-regulation networks to fine-tune phenotypic alternations [14, 41].

### Dominance complementation of deleterious alleles activated epistasis

Heterosis has been reported to result from dominant and epistatic effects involving many genes with complex allelic, intra-genomic, and inter-genomic interactions [43]. The unique design of half-sibling F_1_ populations composed by a diverse panel of complementing heterotic groups provides the unprecedented opportunity to dissect the genetic basis of heterosis utilization in maize improvement. With such a design, we discovered heterosis-related QTLs that might have been overlooked if only one heterotic background was analyzed. For this type of QTLs (Genes C and D), even if they are beneficial to traits and normally segregate in one population, their advantageous effects may be epistatically repressed by another QTLs (Gene X). When the population is hybridized with inbred lines from a complementing group that may generate excellent heterotic effects, such deleterious epistasis is diminished, resulting in expression of superior agronomic traits in F_1_ hybrids. We believe this is a common mechanism of heterosis utilization for single-cross breeding in modern maize industry.

We uncovered three forms of genetic effects underlying heterosis, the most prevalent of which was due to epistatically controlled QTLs (89.2%), with a small proportion of dominant QTLs (~ 5.4%). We have several explanations to the detection of dominant and epistatically controlled QTLs for heterosis. During the process of constructing the 1404 CUBIC lines in six generations of open pollinations and six generations of self-pollinations, the deleterious alleles with large effects negative to fitness might have been eliminated, resulting in the accumulation of many slightly deleterious mutations maintained. On the other hand, given that deleterious allele in maize are often rare [44], the number of founder lines in our study may be limited to detect such rare and modest-effect mutations. That may be why our study directly detected few dominant QTLs through GWAS with very strict significant threshold, although the overall contribution of dominance complementation of deleterious alleles to heterosis can be observed [23]. More importantly, in our design, we propose the dominant complementation of deleterious allele in F_1_ hybrids may release its repressive influence on the detection of downstream QTLs, that were defined as the epistatically controlled QTL. The prevalence of epistatically controlled QTL in our data may support the finding that dominance complementation is crucial for heterosis and may help explaining the co-contribution of dominant QTLs and epistatically controlled QTLs to hybrid vigor.

Molecular interpretation of the abovementioned interaction was exemplified by the relationship of the *BR2* and *ubi3* loci, in which dominance complementation may disrupt the epistatic repression of advantageous alleles to recover superior F_1_ phenotypes (Fig. 5c, d). Heterotic effects arising from such disruption may initially occur on a few large-effect loci in early developmental stages but may then cascade into genome-wide epistatic effects causing heterosis in traits expressed in later developmental stages, such as grain yield (Fig. 5b). Thus, although each epistatic QTL may contribute only a minor effect, their prevalence and their early action in networks amplify the effects arising from additive and dominant QTLs and can ultimately lead to superior performance of F_1_ genotypes. We clearly detected distinct epistatic QTLs in different genetic backgrounds, offering an explanation as to why conclusions from previous heterosis studies using one or a few populations lacked consistency.

**Fig. 7:**
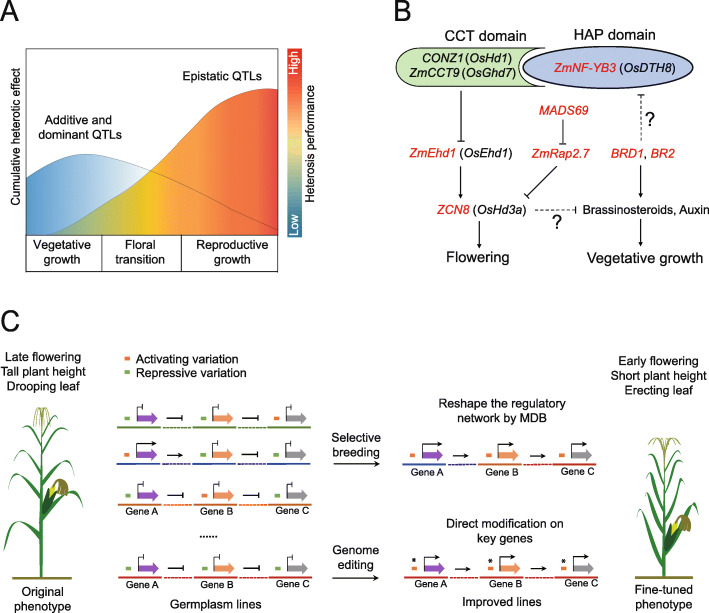
A proposed model of polygenic interactions underlying floral transition facilitating molecular design breeding. **a** Schematic model positing that yield heterosis is mainly attributable to the cumulative effects of epistatic QTLs during the entire developmental process. **b** The hypothetical regulatory pathway underlying floral transition in maize, including the placement of the putative functions of Zm*NF*-*YB3*, *BRD1*, and *BR2*. Orthologous flowering time genes in rice are given in parentheses. Indirect interactions with unknown mechanisms are indicated as dotted lines. Genes detected by GWAS analysis in this study are highlighted in red. **c** Trait improvement with MDB-assisted selective breeding and genome editing. We assume here that three genes form a regulatory network that we can manipulate for refined adjustment of flowering time and plant stature. In selective breeding, since Gene A serves as an epistatic regulator of Genes B and C, natural variants with activating functions for the three genes must be all pyramided into one inbred line. We can now do this via MDB to select the optimal genotypic combination. We can also do this with genome editing, by artificially modifying the repressive variants into their activating variants to achieve the desired improvement goal

The prevalent genetic epistasis identified in this study may be supported by a genome-wide trans-eQTL regulatory network in maize [45], while the allelic interactions between parental genomes contributing to heterosis may arise from the epigenomic reprogramming of key genes that promote growth, fertility, and fitness [46]. On this basis, we suggest a hypothetical model to extend our understanding of maize heterosis. Under this model, yield heterosis results from multiple QTL effects accumulated during the entire developmental process of a hybrid plant (Fig. 7a). During the early stages of vegetative growth, additive and dominant QTLs contribute major effects to plant stature. Once floral transition is initiated, flowering time QTLs with additive and dominant effects interact with the QTLs responsible for vegetative growth to coordinate communication between the vegetative and reproductive modules and to balance flowering time and plant stature. Simultaneously, dominance complementation epistatically allows the activation of additional QTLs, reflected by the genome-wide prevalence of epistatic QTLs contributing to final yield heterosis in each plant.

### Polygenic interactions in maize improvement sets the theoretical basis for molecular design breeding

Selective breeding increases population genetic diversity and the frequency of rare, favorable alleles, followed by artificial selection of desired phenotypes to improve a given trait [3]. The genetic design of the 1404 CUBIC lines developed from the 24 elite founders allows us to interrogate the genomic basis of traits related to improvement in modern crop breeding. We uncovered polygenic interactions in multiple aspects of biological processes contributing to maize improvement, such as the interaction between *BR2* and *ubi3*, which demonstrate the role of epistasis in heterosis. In addition, the results from conditional GWAS based on contrasting genotypes at the major-effect gene *BRD1* indicates the existence of complex polygenic interactions, according to the differential GWAS signals of DTT and PH genes presented in the two subgroups of CUBIC lines. These QTLs mapped to multiple previously reported genes, including *ZCN8*, *MADS69*, *RAP2*.*7*, Zm*EHD1*, *BRD1*, *BR2*, and *ca3p2*, suggesting that these genes function as a network subjected to artificial selection to fine-tune phenotypes.

A role for all the abovementioned genes has been previously described in the control of vegetative growth and floral transition. The developmental process of vegetative-to-reproductive transition is one of the most critical stages in crop breeding, and breeders must select the optimal regulatory pattern to balance flowering time and plant stature. One selection target for this balance has been the *ca3p2* locus, the only QTL detected for both DTT and PH. This gene exhibited highly tissue-specific expression patterns in early developing embryo, tassels 12 days after pollination, V3 leaves, stems and shoot apical meristem tissues (Additional file 2: Figure S11) [47]. Collectively, these results point to a function for *ca3p2* in floral transition and tissue differentiation, and perhaps in bridging the vegetative and reproductive growth modules. The *ca3p2* gene encodes a HEME ACTIVATOR PROTEIN3b (HAP3b) subunit of the CCAAT-box-binding transcription factor belonging to the Nuclear Factor Y (NF-Y) family. It was therefore previously called Zm*NF*-*YB3* [48].

The Zm*NF*-*YB3* gene is homologous to the rice (*Oryza sativa*) gene *DAYS TO HEADING 8* (*DTH8*, also named Os*Ghd8*). This gene encodes a protein with a HAP3h domain that physically interacts with the CCT domain-containing proteins HEADING DATE 1 (*Os*HD1) and *Os*Ghd7 to suppress flowering under long day conditions through the downregulation of EARLY HEADING DATE (Os*Ehd1*) and Os*Hd3a* transcription [49–52]. *Os*DTH8 also exhibits pleiotropic effects on plant height and grain yield, presumably through its indirect interaction with phytohormone signaling pathways [49]. The Zm*NF*-*YB3* gene thus most likely executes a similar function as Os*DTH8* in suppressing flowering, independently of the *MADS69*-*RAP2*.*7*-*ZCN8* module. In addition, the concurrent detection of Zm*NF*-*YB3* with the *BR2* and *BRD1* QTLs suggests the involvement of the brassinosteroid (BR) and auxin phytohormones in coordinating floral transition. This hypothesis is consistent with previous evidence pointing to the importance of BR signaling during floral transition, perhaps involving direct communication between BR and auxin [53–55]. Based on previously characterized orthologous flowering genes in maize and rice, and the new information presented here, we propose a theoretical model that includes the participation of *Zm*NF-YB3 and integrates BR and auxin signaling in the maize flowering pathways (Fig. 7b). Although the model is composed of genetically inferred and indirect interactions that have not yet been validated, it also presents a big picture that can guide future investigations on the mechanisms of floral transition in maize.

We postulate that the design of optimal genotypic combinations is possible due to the underlying processes reshaping gene-regulation networks in the examined germplasm, which result from the recombination between effective regulatory variants in target genes. The above theoretical model of a polygenic network forms a practical basis to employ molecular design with natural sequence variation to fine-tune a phenotype. A role of *BRD1* in the regulation of leaf angle has been validated [56], suggesting that *BRD1* may also be a key gene for the selection of compact maize cultivars featuring early-flowering time, short plant height, and erect leaves, phenotypes that will make such cultivars well-suited for mechanical harvesting. Two biotechnology-facilitated methods may accelerate the achievement of this projected breeding goal. We have described MDB-assisted selective breeding with natural variation; the other method is the direct modification of key genes by genome editing to create artificial variation (Fig. 7c). In selective breeding, multiple factors may restrict the efficiency of MDB, including the allele frequency of the underlying regulatory variation and the complexity of epistatic interactions. Thus, a large effective population size is critical for MDB to select desired genotypic combinations on a sufficient number of candidates. Genome editing may be used to overcome this obstacle by directly creating artificial variation on multiple key gene sequences to form new regulatory patterns.

In summary, our hypothesized model and predicted underlying regulatory changes provides a powerful method to direct hybrid breeding and heterosis utilization for crop improvement in which artificial selection on polygenic interactions can bring together the optimal timing and dosage of relevant gene expression. This will replace the “black box” of phenotypic or GS breeding and allow desired phenotypes to be designed and achieved directly, speeding both gain from selection and the release of new varieties with desirable traits.

## Conclusions

To tackle the century-old mystery of heterosis, we assembled a large-scale novel population design with multiple half-sibling F_1_ populations. This ever-large synthetic population with diverse genetic backgrounds provides the maize community a rich resource for quantitative genetic research, gene mining, and breeding applications for agronomic traits and heterosis. Integrating the art-of-state technology of next-generation sequencing and machine learning, we discovered abundant epistatically controlled loci and genes contributing to heterosis, whose effects are undetectable under one background and thus probably be overlooked in traditional works. The epistatic reformation by complementary hybridization of the maternal and paternal genomes provides the new angle to understand the hybrid superior. The optimal gene haplotypes that dosage-sensitively balanced the plant maturity, plant height, and yield can be assembled or created by genome editing technology, that provided informative guidance and perspective for the future precise breeding.

## Methods

### Development of the thirty F_1_ populations by NC-II design

Development of the CUBIC population used as the maternal inbred lines in this study has been previously reported in Liu et al [34]. The 1404 recombinant inbred lines in the CUBIC population are descendants of 24 elite founder lines that mostly belong to the Lvda Red Cob (LRC) and SiPingTou (SPT) heterotic groups. LRC and SPT have been the most widely used indigenous germplasm in Northern and Central China over the last century, representing a very large genetic pool of local adaptive alleles for the various growing conditions encountered in China [57]. The 30 paternal testers are mostly foreign imported maize inbred lines that have been further improved by Chinese breeders. These lines have diverse genetic backgrounds covering six heterotic groups: Reid, Lancaster, waxy, tropic, P-population, and X-population germplasm.

Crossing the 1428 maternal lines and 30 paternal testers will theoretically generate 42,840 F_1_ combinations. We first planted 8652 F_1_ hybrids in 2014 and 2015 to collect field-measured phenotypes and generate the training population. We planted all 1428 maternal lines and two F_1_ populations, corresponding to a total of 2856 F_1_ hybrids, in 2014 in five locations to collect the phenotypic data. These two F_1_ populations are derived from the crossing of 1428 maternal lines with the Zheng58 and Jing724 testers. We collected phenotypic data over two consecutive years to test for repeatability and calibrate for systematic bias within the dataset. The five chosen locations were the cities of Yushu (Jilin Province, 43° 42′ N, 125° 18′ E), Shenyang (Liaoning Province, 42° 03′ N, 123° 33′ E), Beijing (40° 10′ N, 116° 21′ E), Baoding (Hebei Province, 38° 39′ N, 115° 51′ E), and Xinxiang (Henan Province, 35° 27′ N, 114° 01′ E) in Northern China. In 2015, we planted the 30 paternal testers and 6210 F_1_ hybrids resulting from the crossing of 207 randomly selected maternal lines with the 30 paternal testers in the same five locations to collect phenotypic data. We also included the hybrid cultivar ZhengDan958 as a control once every 50 rows.

### Phenotypic data collection and processing

We planted the 237 parental lines and 8652 F_1_ hybrids in the field following a completely randomized design, in which 17 individual plants per inbred line or F_1_ hybrid were planted as a row. In each location, we planted the maternal line Chang7-2 once every 30 rows as control within the plots of parental lines, and the hybrid cultivar ZhengDan958 once every 50 rows as control within the plots of F_1_ hybrids. We later used the phenotypes measured for Chang7-2 and ZhengDan958 to correct for spatial heterogeneity in the field. We collected phenotypic data for three representative agronomic traits: days to tasseling (DTT), measured as the interval from sowing date to the date when tassels appeared on at least ten individual plants per line; plant height (PH), measured as the vertical height from the ground to the top of the tassel; and ear weight (EW), measured as the average weight of five fully formed ears in the middle of each row to avoid edge effects within the plots.

To reduce the influence from the environment (years and locations), we computed the best linear unbiased prediction (BLUP) value of each F_1_ hybrid and each parental line for the phenotypic data in the five locations and 2 years, using a mixed linear model in the R package “lme4” [58]. BLUP values for each phenotype were then used for subsequent analysis. We computed the middle-parent heterosis (MPH) score as $MPH=\left({y}_h-\frac{y_m+{y}_p}{2}\right)/\frac{y_m+{y}_p}{2}$, where *y*_*h*_ is the hybrid phenotypic values and *y*_*m*_ and *y*_*p*_ are the phenotypes from the maternal and paternal line, respectively. Because phenotypic variation caused by population stratification may introduce systematic bias in genomic selection (GS) prediction, we normalized BLUP values calculated for the F_1_ hybrids within each set of F_1_ populations to relative values using the *z*-score algorithm: ${z}_i=\left({y}_i-\overline{y_{.}}\right)/ sd\left({y}_{.}\right)$, where *y*_*i*_ is the phenotype of the F_1_ hybrid or parental line *i*, $\overline{y_{.}}$ is the mean phenotype of the F_1_ hybrid or parental line and *sd*(*y*_._) is the standard deviation of the phenotype in the F_1_ hybrid or parental line. The *z*-scores were subsequently used in GS predictions.

### Genotypic data processing

We called 14.8 million single nucleotide polymorphisms (SNPs) from whole-genome resequencing of all 1428 maternal lines and 30 paternal testers by using the same pipeline described in the previously reported analysis of the CUBIC population [34]. Before inferring the genotypes of all 42,840 F_1_ hybrids, we performed a series of SNP filtering steps using the following criteria: (1) if a SNP was heterozygous in a parent, the SNP in the hybrid was annotated as missing; (2) SNPs showing a heterozygous rate ≥ 10% in the maternal population and heterozygous rate ≥ 1/30 in the paternal population were removed; (3) SNPs showing no polymorphism in either the maternal or paternal populations were removed; (4) SNPs showing minor allele frequency (MAF) < 0.02 in parental lines were removed; (5) SNPs showing minor homozygous genotype < 0.5% (210 lines) out of the 42,840 F_1_ hybrids were removed. SNP filtering resulted in 4,549,828 high-quality and informative SNPs remaining for downstream analysis. We then inferred the genotypes of all 42,840 F_1_ hybrids from the genotypes of their maternal and paternal parents for these 4,549,828 SNPs. Missing genotypes of the 42,840 F_1_ hybrids were imputed using the software Beagle version 4.0 [59]. We performed a population structure analysis using the FlashPCA software [60], and constructed the phylogenetic tree for the 1458 parental lines using the unweighted neighbor-joining method implemented in the R package “phangorn” [61].

### G2P prediction of the 34,188 F_1_ hybrids

We used data from the 8652 F_1_ hybrids (20.2% of the samples, consisting of the modeling population with known genotypes and phenotypes) to train the model to predict the phenotypes of the remaining 34,188 F_1_ hybrids (79.8% of the samples, comprising the predicting population with known genotypes). The 1:4 sample division is a commonly used ratio in the seed industry to perform G2P-assisted breeding, considering the balance between phenotyping cost and prediction precision [62]. We utilized the genomic best linear unbiased prediction (gBLUP) model to complete the phenotypes of samples from the thirty F_1_ hybrid populations using the “sommer” package in R [63]. The model used to implement gBLUP was:

$$ y= X\beta + Zu+\varepsilon $$

where *y* is a vector of phenotypes, *X* is a designated matrix for the fixed effects, *β* is a vector of fixed effects, *Z* is a designated matrix for random effects, *u* is a vector of additive genetic effects for an individual with variance $K{\sigma}_u^2$ in which *K* is the centered genomic relationship matrix (cGRM) deduced by the GEMMA (Genome-wide Efficient Mixed Model Association) software [64] based on all of the ~ 4.5 million high-quality SNPs called from resquencing data of the 1458 parental lines, and *ε* is a vector of residual errors with variance $I{\sigma}_e^2$. To reduce the influence caused by population stratification, we treated the phenotypes of the maternal and paternal lines as covariates (fixed effects) in the model.

The G2P model took the *z*-score-transformed phenotypes of training samples in order to avoid the systemic bias across the thirty sets of F_1_ populations. When prediction was accomplished, the *z*-score form of predicted phenotypes were converted back to absolute values using the following algorithm applied within each F_1_ population: ${y}_i={z}_i\times sd(y)+\overline{y}$, where *z*_*i*_ is the *z*-score form of the predicted phenotypes of F_1_ hybrid *i* in the predicting population; $\overline{y}$ and *sd*(*y*) are the mean and standard deviation of the phenotypes of training samples, respectively. To evaluate model precision, we computed the Pearson correlation coefficient (*r*) between predicted phenotype and observed phenotype for the samples with known phenotypes.

### Simulation one: Evaluation of GWAS accuracy at the six levels of phenotype prediction accuracy

To quantitatively evaluate GWAS accuracy in terms of both false discovery rate (FDR) and detection power (DP), a series of simulation analyses were conducted. Based on the actual genotypes from the 4.5 million SNPs in the CUBIC population, we randomly selected a set of 100,000 SNPs for simulations. Assuming a polygenic trait is determined by 20 QTNs (quantitative trait nucleotides) contributing effects following an exponential distribution, the 20 QTN effects were expressed as *β*_*i*_ = 0.96^*i*^ where *i* = 1, 2, 3, …, 20 [65]. From 100,000 SNPs, the 20 QTNs were spiked into a set of randomly selected SNPs (0.25 ≤ MAF ≤ 0.35).

We first simulated the observed phenotype of one sample by summing the 20 QTNs’ contribution expressed as $y={\sum}_{i=1}^{20}{x}_i{\beta}_i$ where *x*_*i*_ and *β*_*i*_ are the numeric genotypic values and simulated QTN effects at the *i*th QTN, respectively. Contribution of each QTN was further represented by a value of PVE (phenotypic variance explained) expressed as *PVE* = *βf*(1 − *f*) where *β* is the QTN effect and *f* is allelic frequency in the 1428 samples. Based on PVEs, the 20 QTNs were categorized into three classes of QTNs with major (4 QTNs, PVE ≥ 7%), moderate (6 QTNs, 4.5% ≤ PVE < 7%), and minor (10 QTNs, PVE < 4.5%) contributing effects.

Then, we simulated the predicted phenotypes with six levels of prediction accuracy, so that the correlation between GWAS and G2P accuracies may be established. Simulation of predicted phenotype was based on the equation of *y*^′^ = *y* + *ε* where *y* is the simulated observed phenotype and *ε* is the residual representing the fluctuated prediction bias assumed following a normal distribution expressed as $$ N\left(0,\raisebox{1ex}{${\sigma}_y^2\left(1-{r}^2\right)$}\!\left/ \!\raisebox{-1ex}{${r}^2$}\right.\right) $$ where *r*^2^ is the squared correlation coefficient between the observed and predicted phenotypes. Thus, quantitative evaluation of FDR and DP of GWAS under six levels of prediction accuracy (*r*^2^ = 1.0, 0.8, 0.6, 0.4, 0.2, and 0.1) may be carried out. Because GWAS power is also influenced by population size, each simulation was performed for six times, using five randomized subsets (207, 400, 600, 800, and 1000 samples) out of the 1428 samples as well as the complete set. Then, 36 combinations of population size and prediction accuracy were set for one round of simulation and each round was repeated for 500 times to obtain averaged results of FDR and DP. During each time of repeat, a new set of 20 QTNs and predicted phenotypes with randomized residuals were generated. Finally, the whole simulation process generated a total of 18,000 sets of GWAS results for computing averaged FDR and DP under the six different circumstances of population size and prediction accuracy.

### Simulation two: Correlation between population size and GWAS detection power using a spike-in QTN under different genetic structures

To demonstrate that any enhancement of GWAS detection power using 1428 samples can be attributed to the enlarged population size compared to that of 207 samples, we conducted a simulation analysis to determine the correlation between population size and GWAS detection power. The GWAS simulation was conducted following a previously published procedure [66].

In this procedure, one SNP (0.25 ≤ MAF ≤ 0.35) was randomly picked from the ~ 4.5 million SNPs, and a spike-in QTN (quantitative trait nucleotide) effect was assigned to this SNP; The QTN effect is a designated coefficient of the standard deviation (SD) of the actual phenotypic distribution of Zheng58 F_1_ hybrids, in which 19 gradients of QTN effects ranging from 0.1 to 1.9 with a step of 0.1 were used for each round of simulation. Based on the genotypes (two alleles, e.g., AA and AG corresponding to high and low phenotypes, respectively) of the chosen SNP among Zheng58 F_1_ hybrids, the original phenotype was transformed to a simulated phenotype, calculated using the original phenotype of the AA hybrids + (QTN effect × SD) and AG hybrids − (QTN effect × SD). Under each QTN effect gradient, we randomly picked 100 SNPs to generate 100 simulated phenotypic distributions for the Zheng58 F_1_ hybrids. Next, we performed GWAS 100 times using the 100 sets of simulated phenotypes for the Zheng58 F_1_ hybrids. Using a *p*-value = 2.2 × 1e− 7 as the significance cutoff, we counted the number of significant SNPs (*n*) out of the 100 SNPs passing the cutoff and used the corresponding percentage (*n*/100) to represent the GWAS detection power. For each trait, we repeated the above procedure 19 times using the 19 QTN effect gradients to generate GWAS detection power predictions on the 207 and 1428 samples for a comparison. Finally, we conducted a simulation for effect and sample sizes that impact on the GWAS detection power under the genetic architectures of three real traits: DTT, PH, and EW in both measured and predicted phenotypic variations. With this simulation analysis, we deduced that a significant QTN with an effect of 0.4 would be detected using 1428 samples, while using 207 samples the QTN effect would have to be over 1.2 to be detected.

### GWAS analysis and QTL summary

To limit the influence of population stratification caused by the 30 paternal testers from different heterotic groups, we performed GWAS analysis within each set of F_1_ populations using the GEMMA software. The pairwise kinship matrix was calculated for each set of 1428 F_1_ hybrids using the centered genomic relationship matrix (GRM) algorithm in GEMMA based on the ~ 4.5 million SNPs. We then loaded the GRM with the model as a polygenic random effect. We performed a total of 183 rounds of GWAS on 1428 maternal inbred lines, for the three traits and their associated MPH values for each of the 30 F_1_ populations.

To better interpret the genetic architecture and identify key genes for the six traits, we summarized all QTLs for DTT, MPH.DTT, PH, MPH.PH, EW, and MPH.EW from the 183 sets of GWAS results in the maternal population and the 30 F_1_ populations as follows. We grouped significant SNPs (*p*-value ≤ 1e− 6 cutoff) that were spaced less than 800 kbp apart into one locus, 800 kbp being the length of linkage disequilibrium (LD) decay at *r*^2^ = 0.1 [34]. We calculated the pairwise LD between two adjacent loci; if any pairs of SNPs between the two loci had *r*^2^ > 0.1 and the two peaks were spaced < 5 Mbp apart, the two adjacent loci were further merged as a single locus. This process was performed until no more loci could be merged. Next, if a locus contained ≥ 10 significant SNPs, the locus was defined as a QTL in which the most significant SNP was chosen as the peak SNP. If a locus contained < 10 significant SNPs, an extended region of 400 kbp (half of the LD length) was searched for SNPs exceeding *p*-value = 1e− 4, until the locus contained ≥ 10 SNPs at this suggestive level, at which point it was called a QTL with the most significant SNP chosen as the peak SNP. After summarizing QTLs for each trait or MPH associated with each trait within each F_1_ population, we generated an overall QTL summary by combining the QTLs over the 30 F_1_ populations. This was done by merging the overlapping QTLs from within any two F_1_ populations. Therefore, for each trait, three sets of merged QTL maps were generated, including QTLs identified in the maternal population; and QTLs of the trait and of the MPH.Trait in hybrid populations.

## Supplementary Information

**Additional file 1.** Heterotic group classification of the 30 paternal testers.

**Additional file 2.** Figures S1-S11.

**Additional file 3.** Prediction precision of the thirty F1 populations.

**Additional file 4.** Manhattan plots for GWAS of three traits across all populations.

**Additional file 5.** QTL summary for three traits detected by GWAS across the maternal and 30 F1 populations.

**Additional file 6.** The merged QTLs and genetic effects detected by the three traits.

**Additional file 7.** Review history.

## Data Availability

Raw whole-genome sequencing reads of CUBIC population (1428 maternal lines) and 30 paternal inbred lines are available on NCBI with the Bioproject accession number PRJNA597703 [67]. The genotypic and phenotypic data of the 1458 parental inbred lines and the 42,820 F_1_ hybrids were publicly available in the ZEAMAP database [68]. The scripts for phenotypic data normalization, BLUP calculation across multiple environments, genomic prediction, and data simulation for GWAS power can be accessed at GitHub [69], which is licensed under the GNU General Public License v3.0. Correspondence and requests for materials and data should be addressed to J-B.Y. (yjianbing@mail.hzau.edu.cn).
