# Supplementary material for: The genetic mechanism of heterosis utilization in maize improvement
Source: Genome Biol. 2021 May 10;22:148. doi: 10.1186/s13059-021-02370-7 (PMC8108465; doi:10.1186/s13059-021-02370-7)
Supplement: Supplementary file 2 — Additional file 2. Figures S1-S11. [file 13059_2021_2370_MOESM2_ESM.docx]

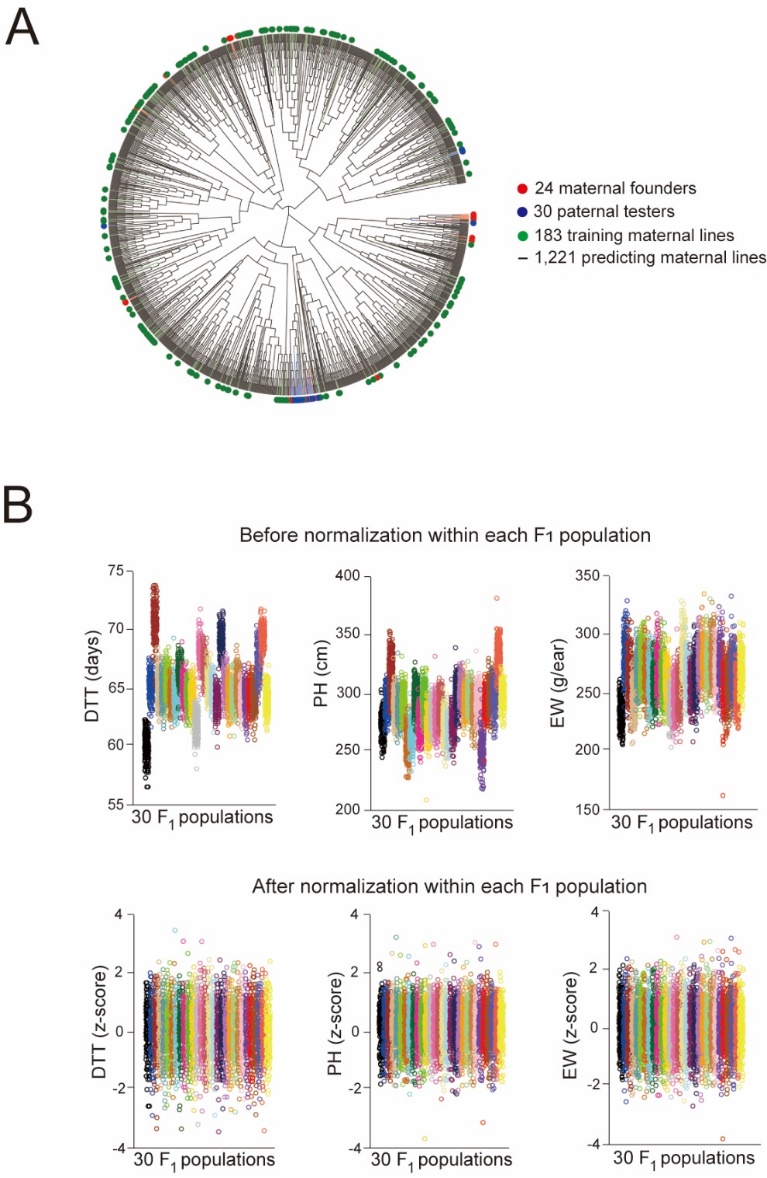


**Figure S1.** Phenotypic stratification caused by the heterotic groups. **A**) Phylogenetic tree of the 24 maternal founders, 30 paternal testers, 183 training lines and 1,221 predicting lines. **B**) Before and after z-score normalization of F_1_ phenotypes.


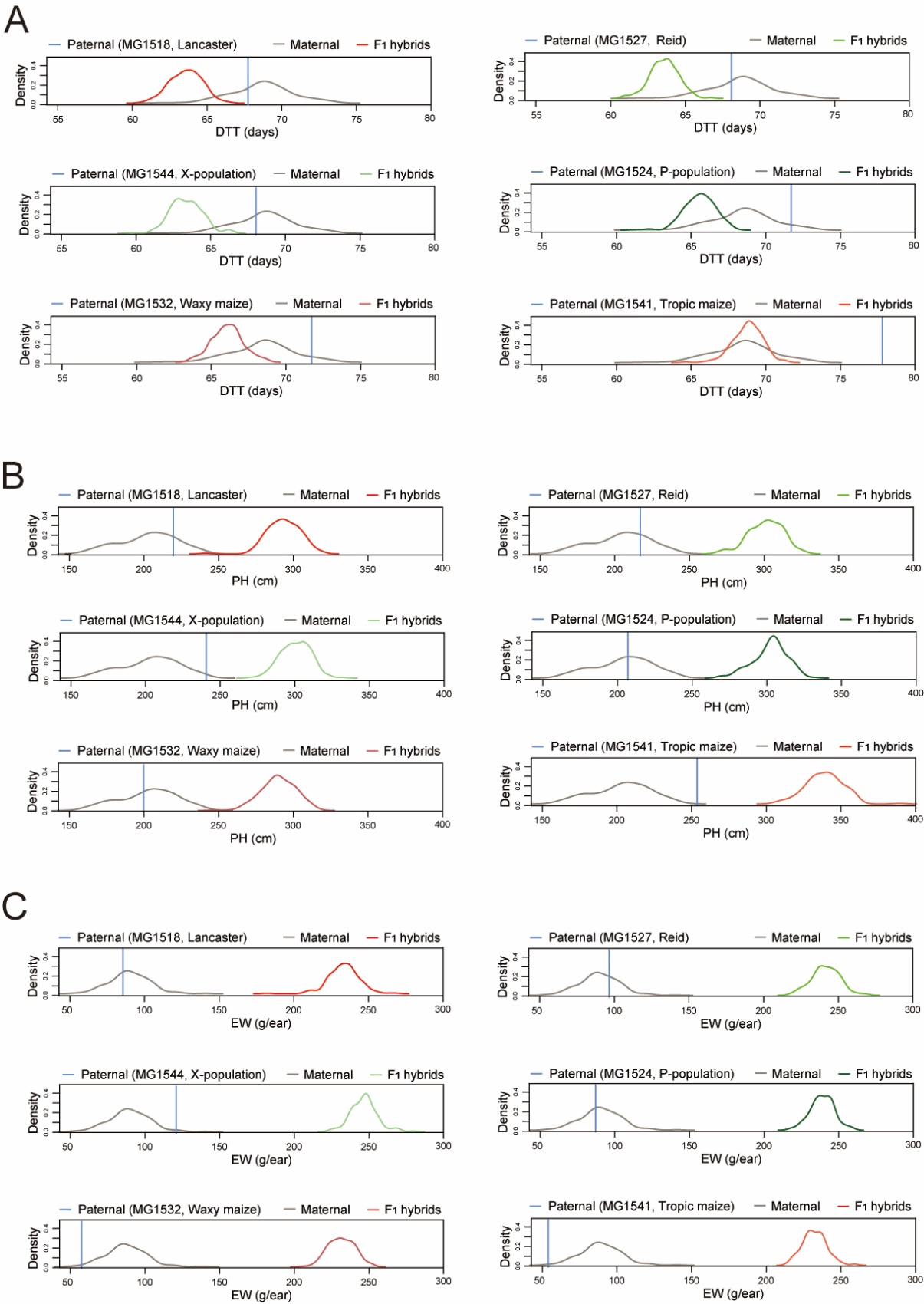


**Figure S2.** Superior heterotic performance of **A**) DTT, **B**) PH and **C**) EW in the maternal population and six F_1_ hybrid populations generated from crossings of six representative paternal testers from the six heterotic groups, namely MG1518 from Lancaster group, MG1527 from Reid group, MG1544 from X-population, MG1524 from P-population, MG1532 from waxy maize and MG1541 from tropic maize.


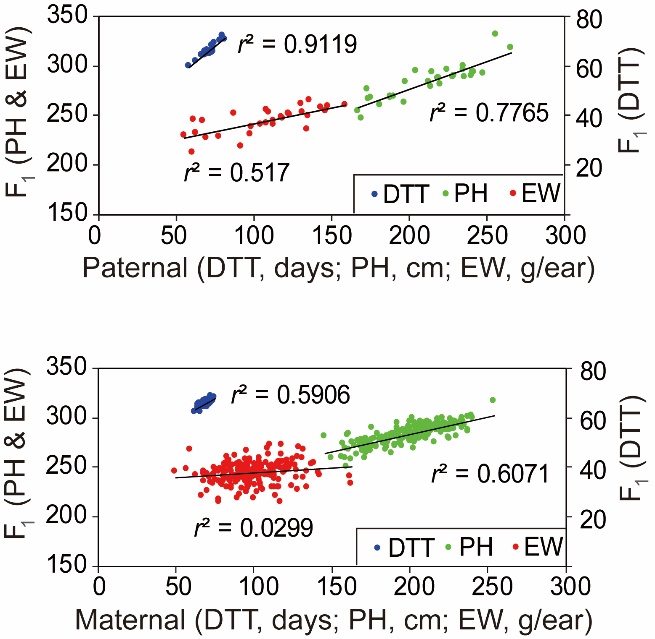


**Figure S3.** The phenotypes measured in the F_1_ hybrids correlate with those of the paternal lines (top panel) and maternal lines (bottom panel). Each dot represents the average phenotypic value of the F_1_ hybrids sharing the same parental line *versus* the phenotypic value of the corresponding parental line.


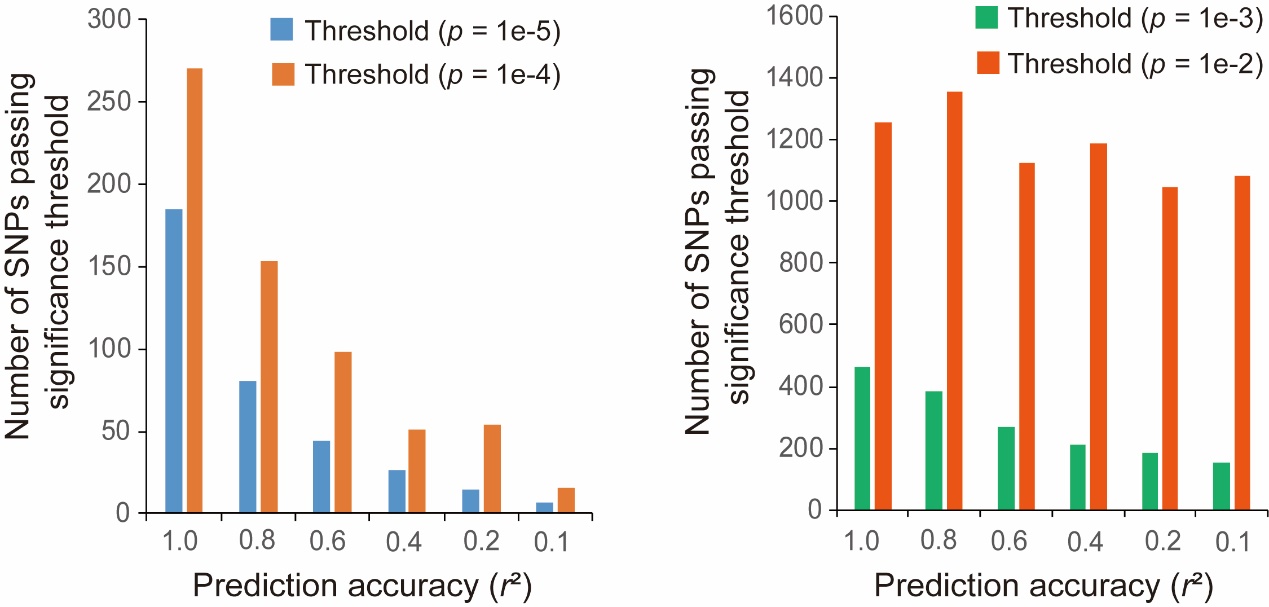


**Figure S4.** Numbers of the SNPs passing the significance thresholds of *p* value = 1e-5, 1e-4, 1e-3, 1e-2 at the six levels of prediction accuracies.


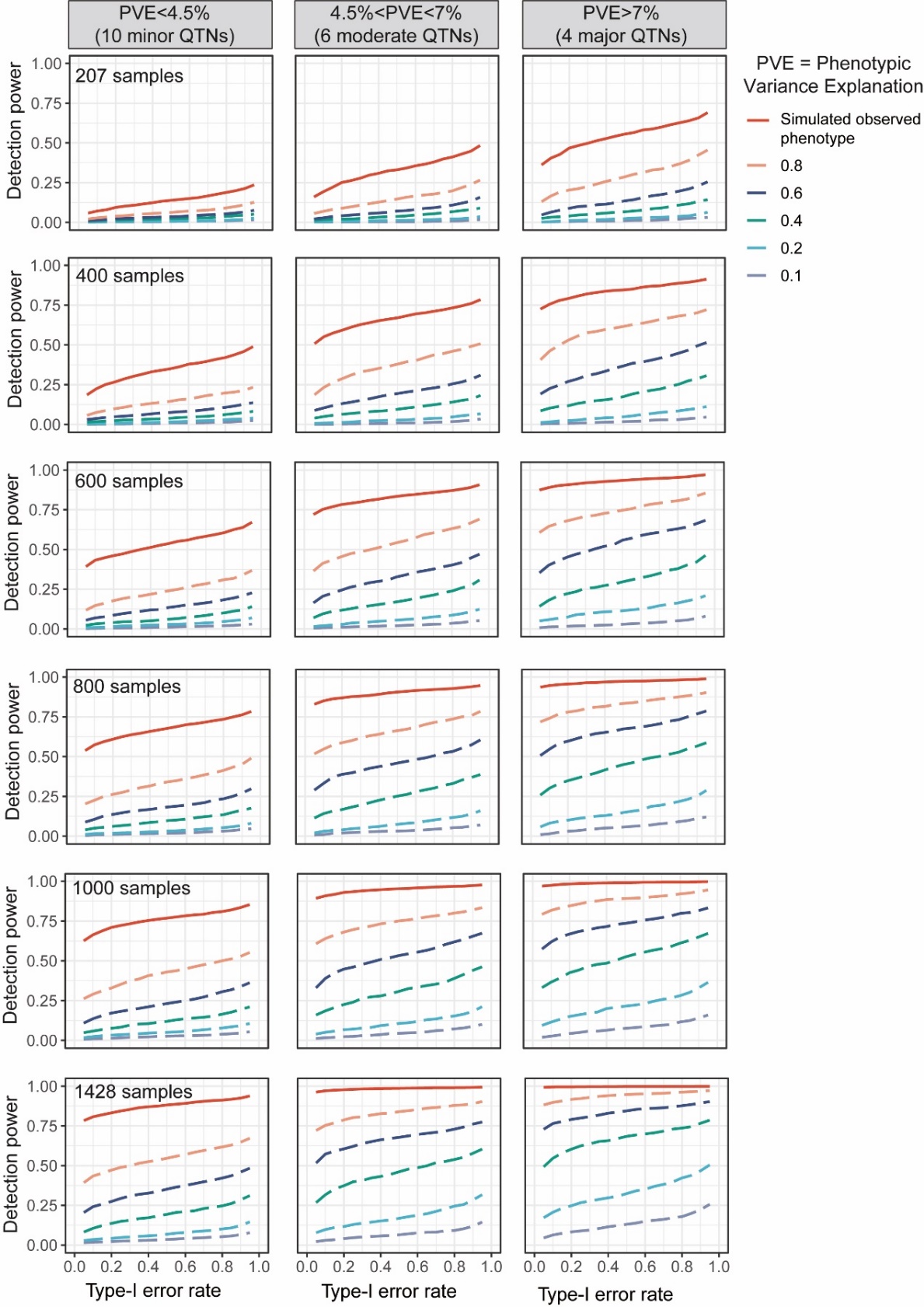


**Figure S5**. GWAS detection powers measured by the 20 spike-in QTNs at different levels of prediction accuracy and population size. The 20 spike-in QTNs were classified as 4 major, 6 moderate and 10 minor QTNs.


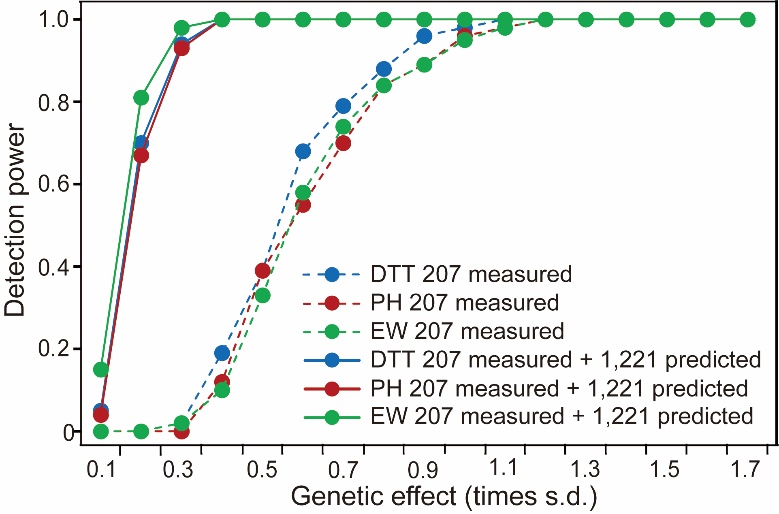


**Figure S6**. Simulation analysis indicating that GWAS power mainly improves due to larger sample size, since variants with small genetic effects missed in the small population of 207 samples can be detected in the larger population of 1,428 samples.


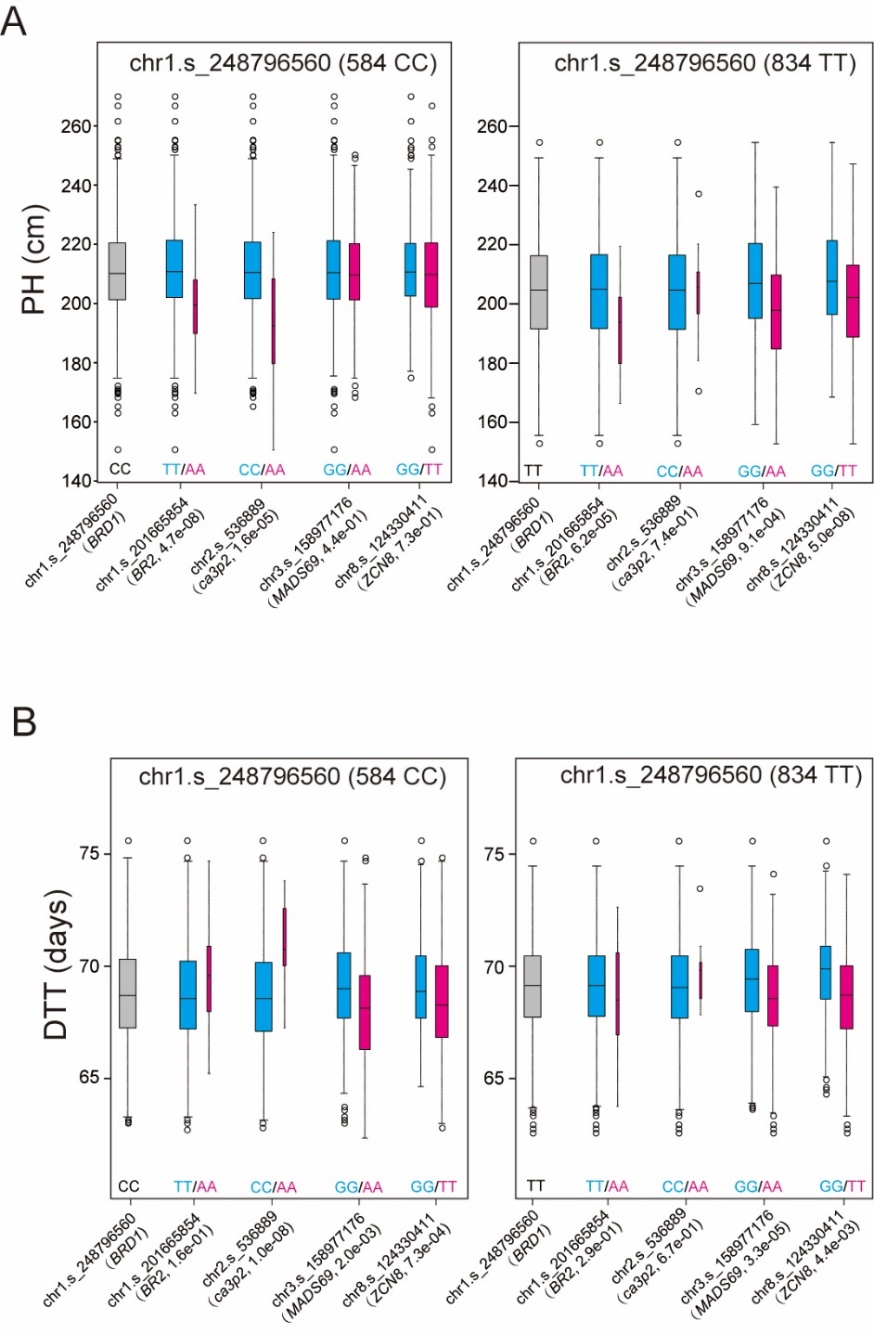


**Figure S7.** Phenotypic distributions of **A**) PH and **B**) DTT for the maternal lines carrying different genotypes for *BR2*, *ca3p2*, *MADS69*, *ZCN8* in the *BRD1*-CC and *BRD1*-TT backgrounds. In the *BRD1*-CC group, the lines bearing the *BR2*-AA and *ca3p2*-AA genotypes exhibited significantly shorter PH than those bearing *BR2*-TT and *ca3p2*-CC genotypes, respectively; however, in the *BRD1*-TT group the difference was weaker. In the case of *MADS69* and *ZCN8*, the PH in lines bearing the *MADS69*-AA and *ZCN8*-TT genotypes showed no significant differences with those bearing the *MADS69*-GG and *ZCN8*-GG genotypes in the *BRD1*-CC group, but a significant difference in PH was observed within the *BRD1*-TT group. When comparing DTT with the same subgroup division, *ca3p2*-AA lines exhibited significantly shorter DTT than *ca3p2*-CC lines in the *BRD1*-CC group, while DTT became equal in the *BRD1*-TT group. In addition, *MADS69*-AA lines exhibited slightly earlier DTT than *MADS69*-GG lines in the *BRD1*-TT group, while the two genotypes of *ZCN8* showed no differences in the two genotypic backgrounds of *BRD1*.


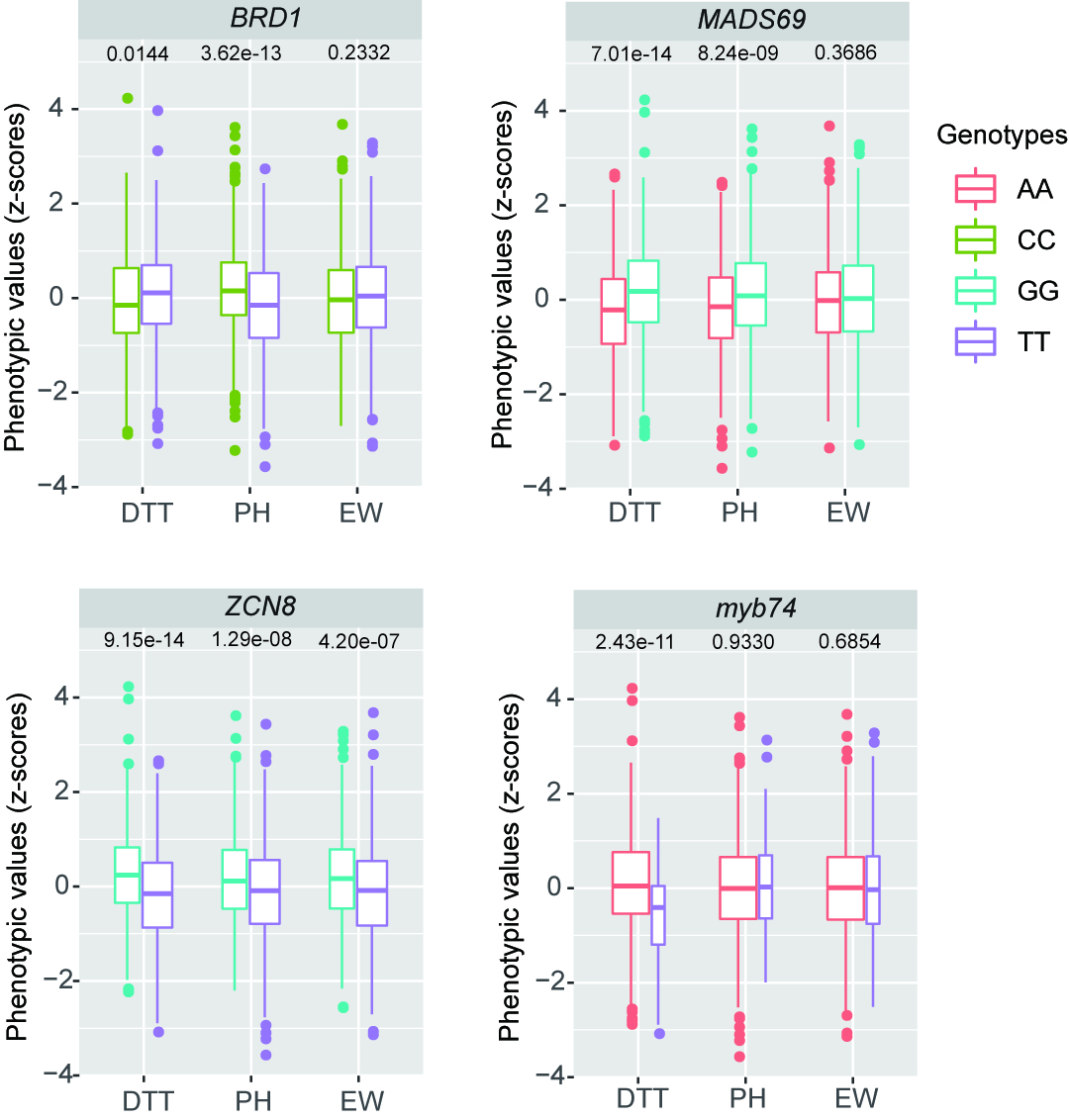


**Figure S8.** Phenotypic distribution of the two groups of maternal lines bearing the two contrasting genotypes for *BRD1*, *MADS69*, *ZCN8* and *myb74*.


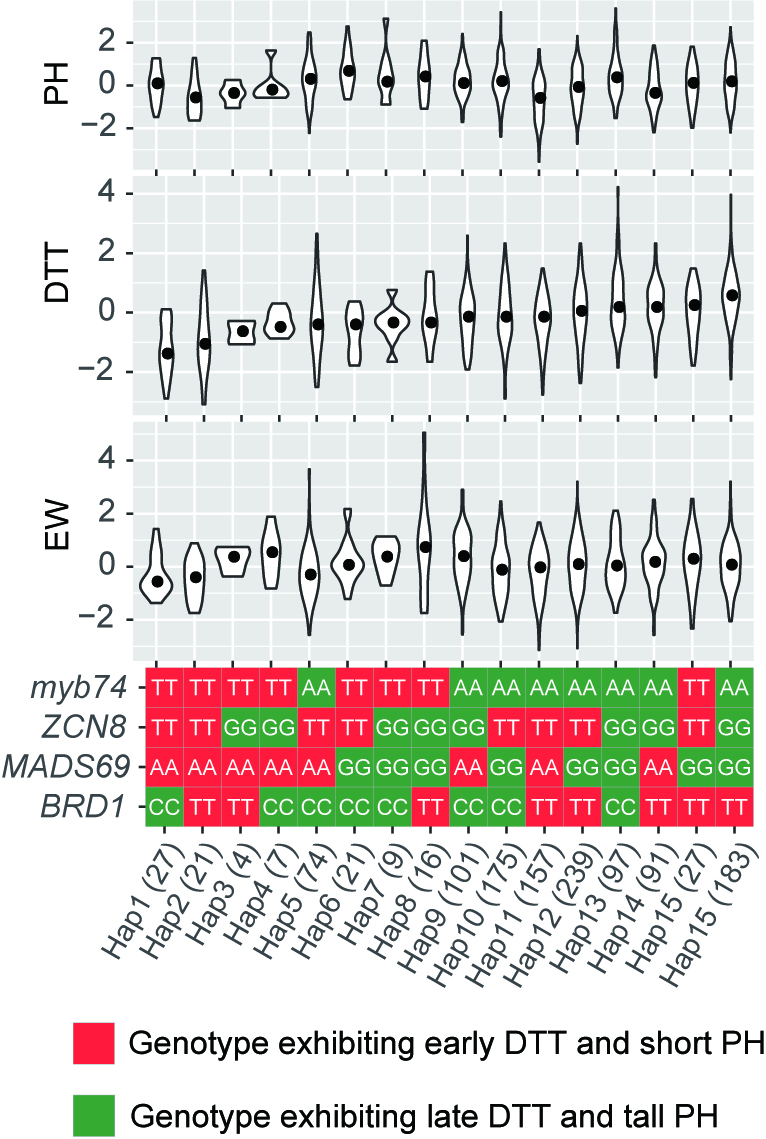


**Figure S9.** Fifteen combinations (Hap1 to Hap15 haplotypes) of the genotypes of *ZCN8*, *MADS69*, *myb74* and *BRD1* detected among the 1,428 maternal lines. The numbers in the brackets indicate the number of lines bearing the corresponding haplotypes. Phenotypic distributions of DTT, PH and EW of the lines corresponding to each type of combinations were plotted as violin plots.


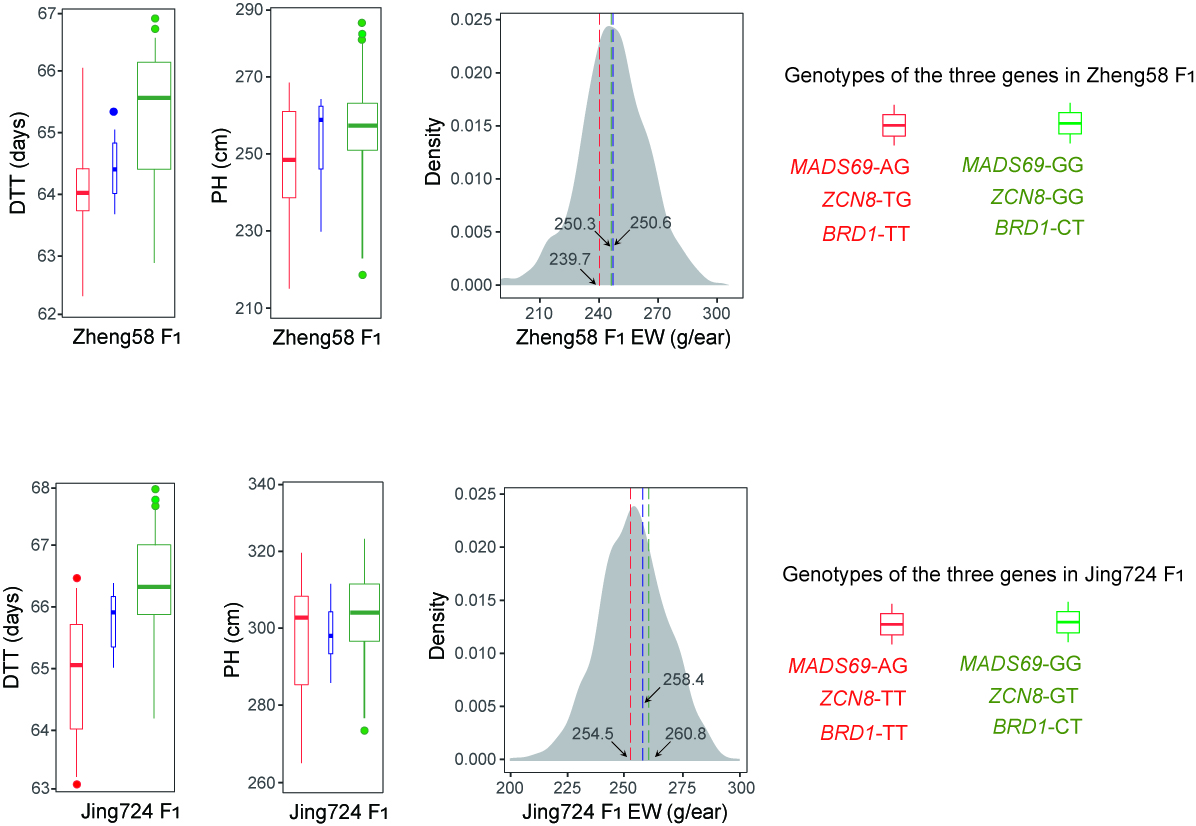


**Figure S10.** Comparison of the F_1_ phenotypes of the 11 lines in Hap2, 48 lines in Hap1 and 280 lines in Hap8 crossed with Zheng58 (upper panel) and Jing724 (lower panel).


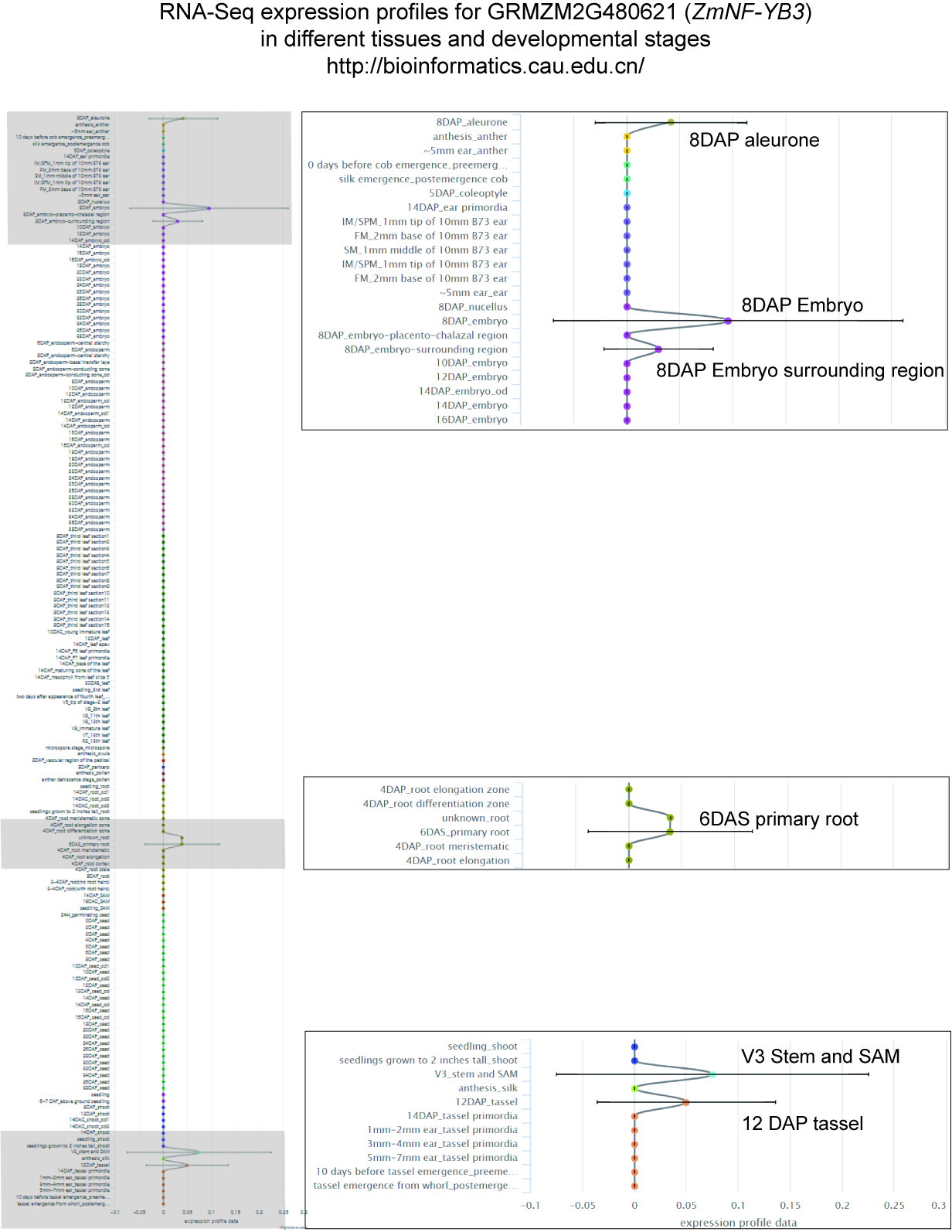


**Figure S11.** Expression profiles for *GRMZM2G480621* (Zm*NF-YB3*, *ca3p2*) in different tissues and developmental stages in maize B73.
