# Supplementary material for: The genetic mechanism of heterosis utilization in maize improvement
Source: Genome Biol. 2021 May 10;22:148. doi: 10.1186/s13059-021-02370-7 (PMC8108465; doi:10.1186/s13059-021-02370-7)
Supplement: Supplementary file 4 — Additional file 4. Manhattan plots for GWAS of three traits across all populations. [file 13059_2021_2370_MOESM4_ESM.pdf]

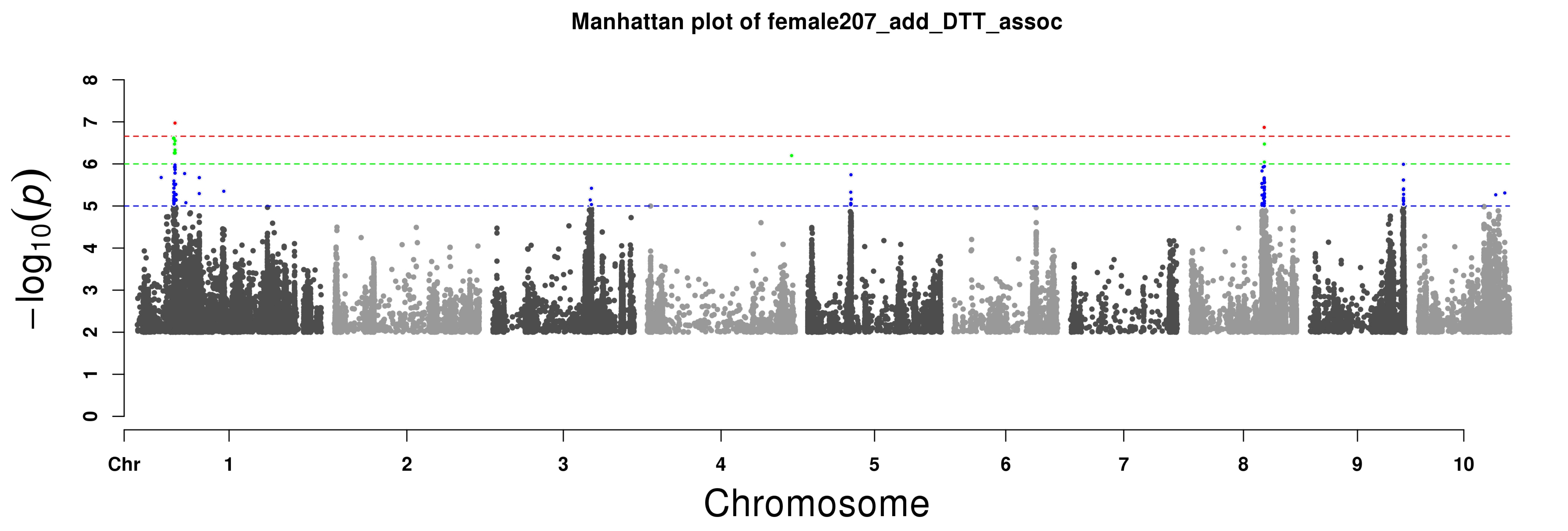

Manhattan plot of female207_add_DTT_assoc
$-\log_{10}(p)$
0
1
2
3
4
5
6
7
8
Chr
1
2
3
4
5
6
7
8
9
10
Chromosome

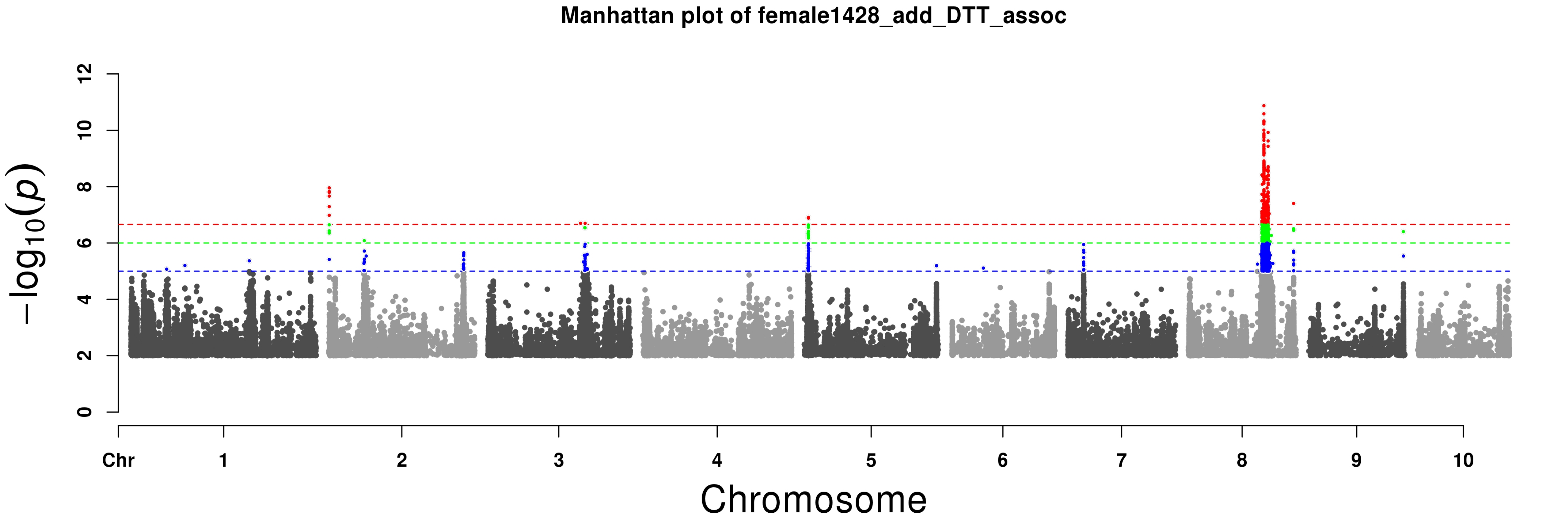

Manhattan plot of female1428_add_DTT_assoc
$-\log_{10}(p)$
0
2
4
6
8
10
12
Chr
1
2
3
4
5
6
7
8
9
10
Chromosome

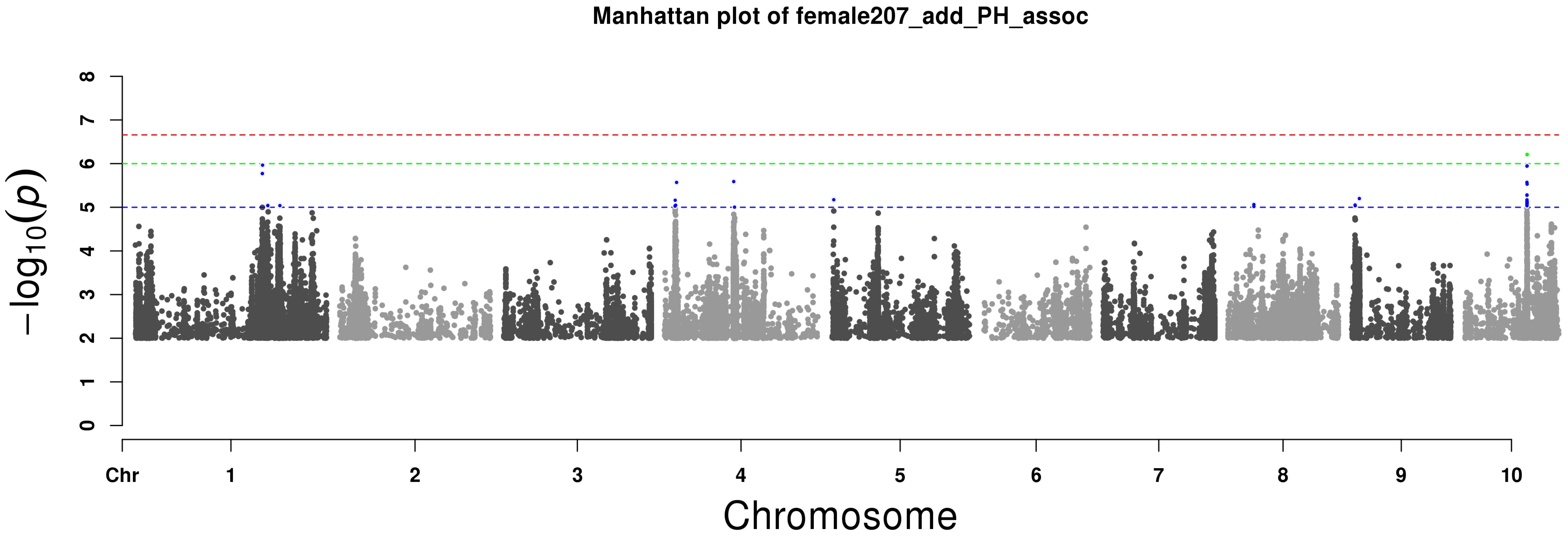

Manhattan plot of female207_add_PH_assoc
$-\log_{10}(p)$
0
1
2
3
4
5
6
7
8
Chr
1
2
3
4
5
6
7
8
9
10
Chromosome

Manhattan plot of female1428_add_PH_assoc

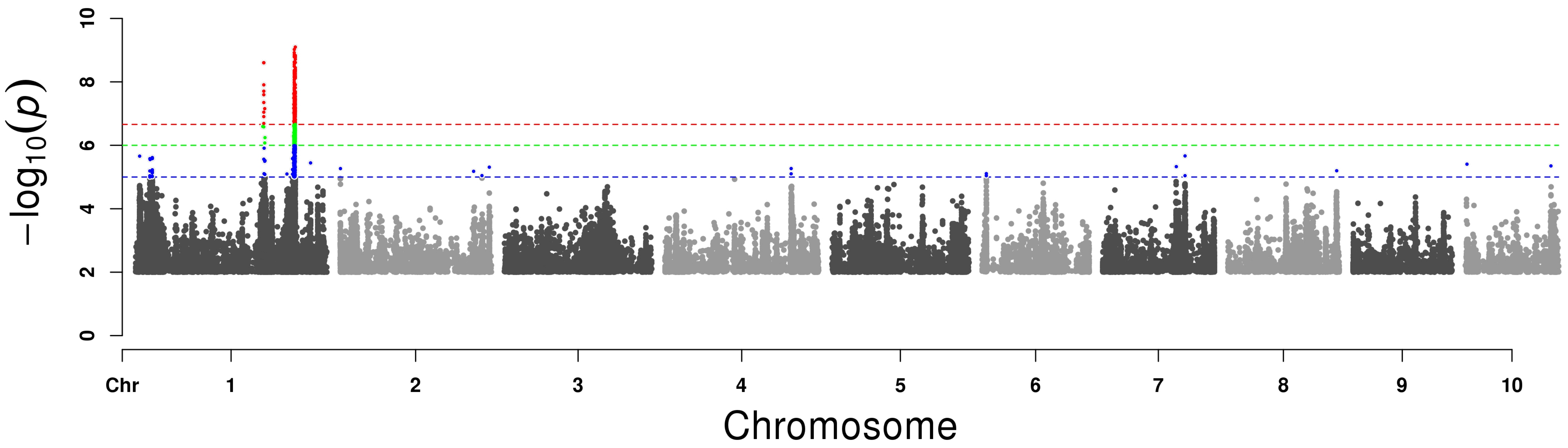

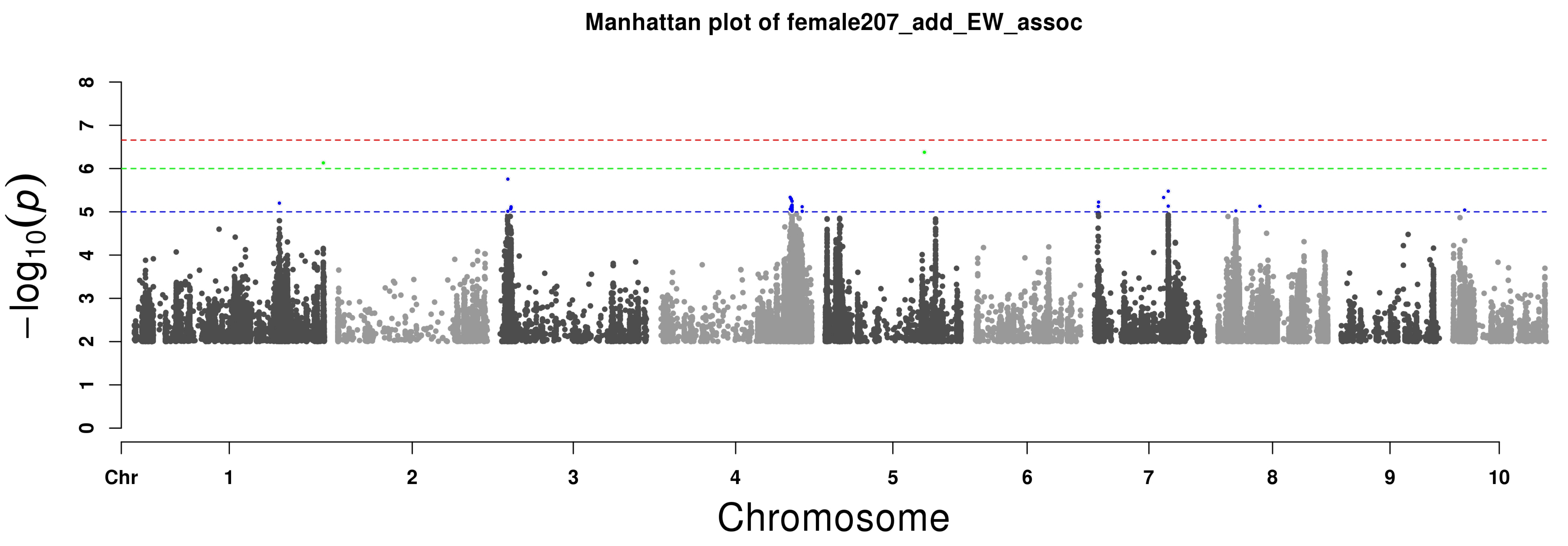
Manhattan plot of female207_add_EW_assoc
$-\log_{10}(p)$
0
1
2
3
4
5
6
7
8
Chr
1
2
3
4
5
6
7
8
9
10
Chromosome

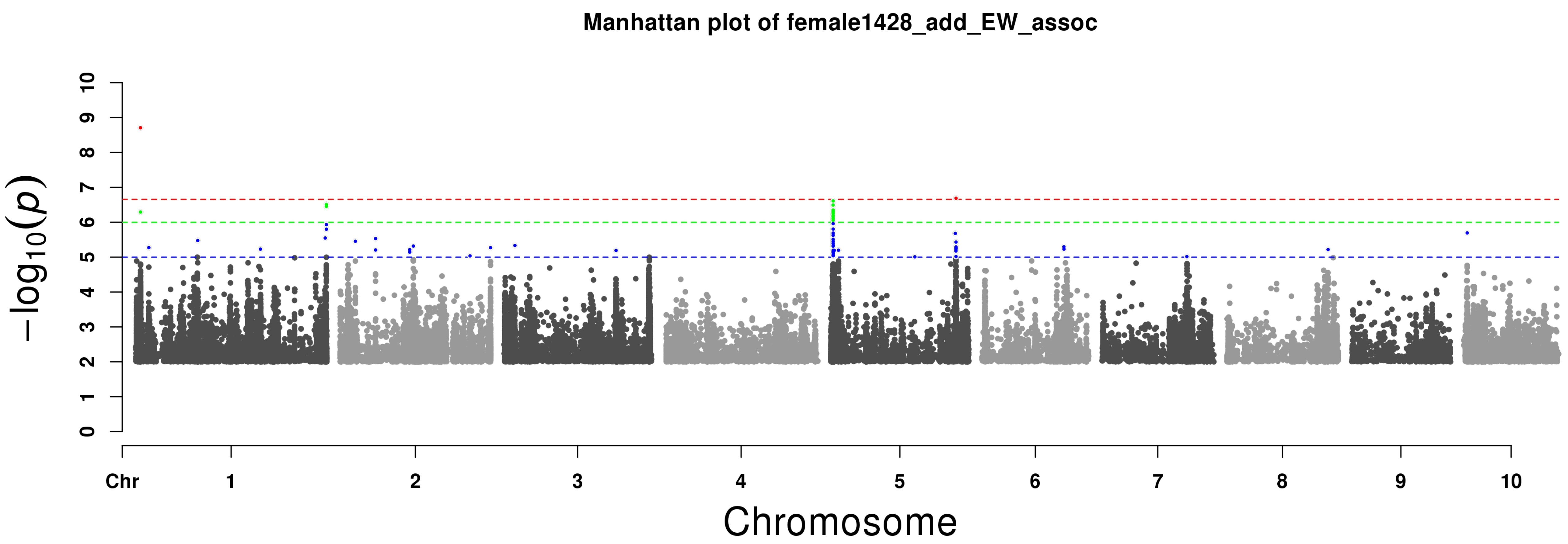
Manhattan plot of female1428_add_EW_assoc
$-\log_{10}(p)$
0
1
2
3
4
5
6
7
8
9
10
Chr
1
2
3
4
5
6
7
8
9
10
Chromosome

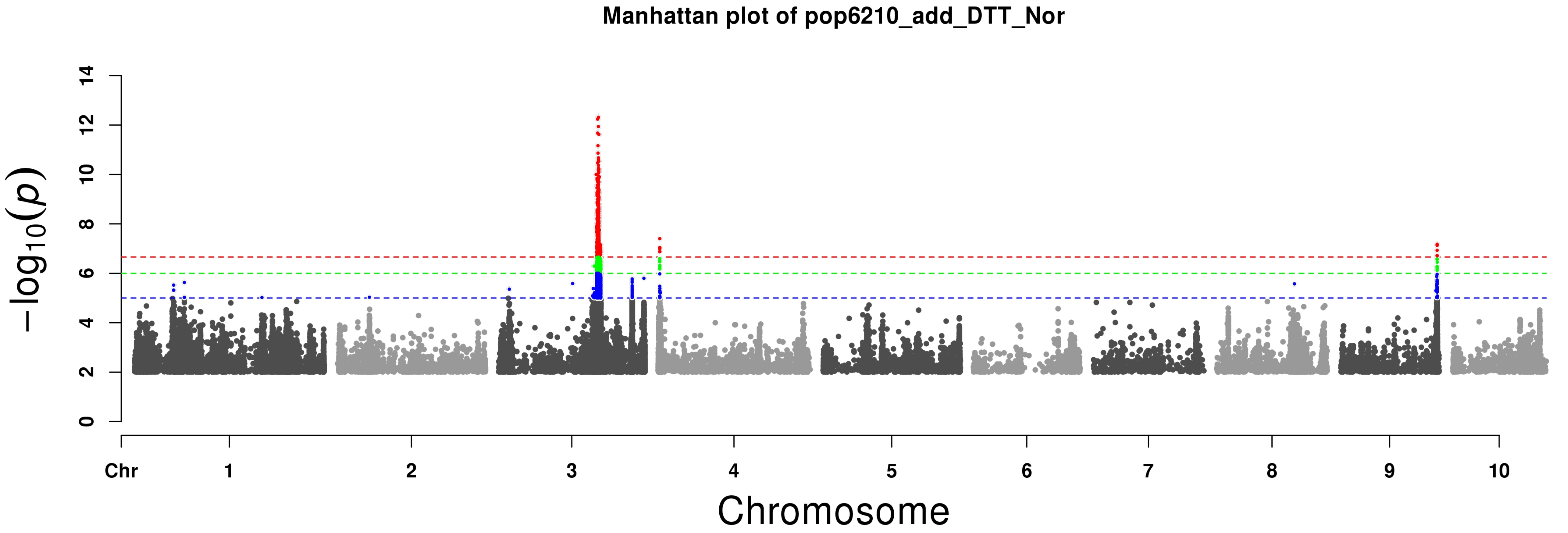

Manhattan plot of pop6210_add_DTT_Nor
$-\log_{10}(p)$
0
2
4
6
8
10
12
14
Chr
1
2
3
4
5
6
7
8
9
10
Chromosome

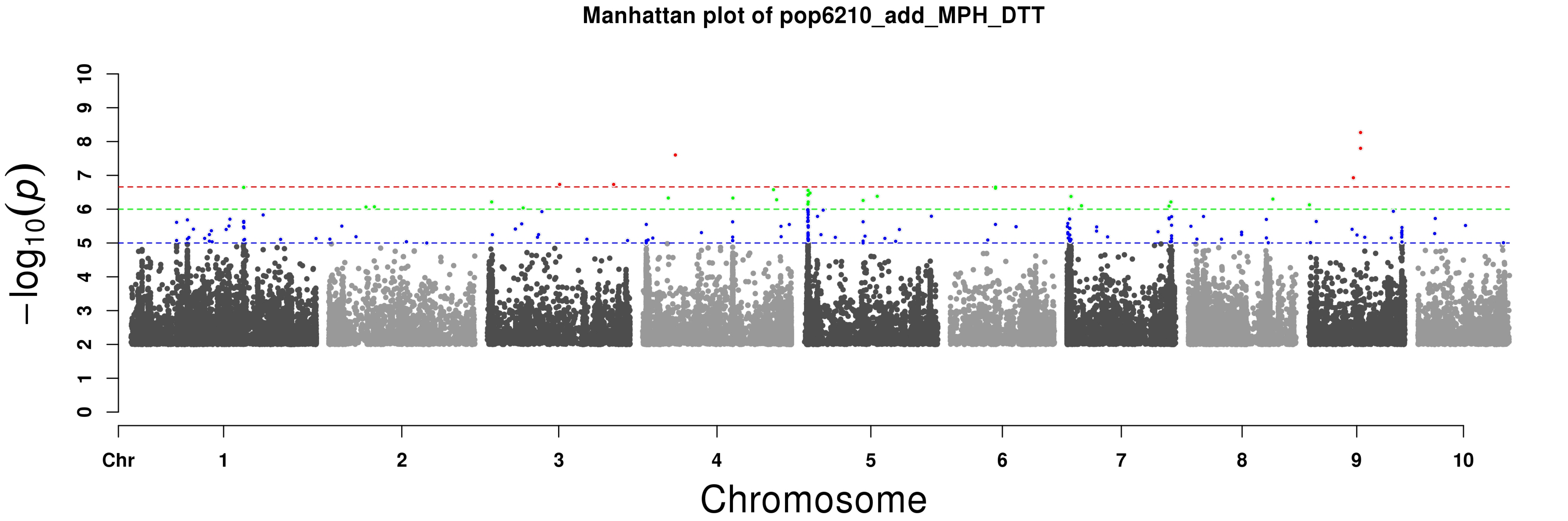

Manhattan plot of pop6210_add_MPH_DTT
$-\log_{10}(p)$
0
1
2
3
4
5
6
7
8
9
10
Chr
1
2
3
4
5
6
7
8
9
10
Chromosome

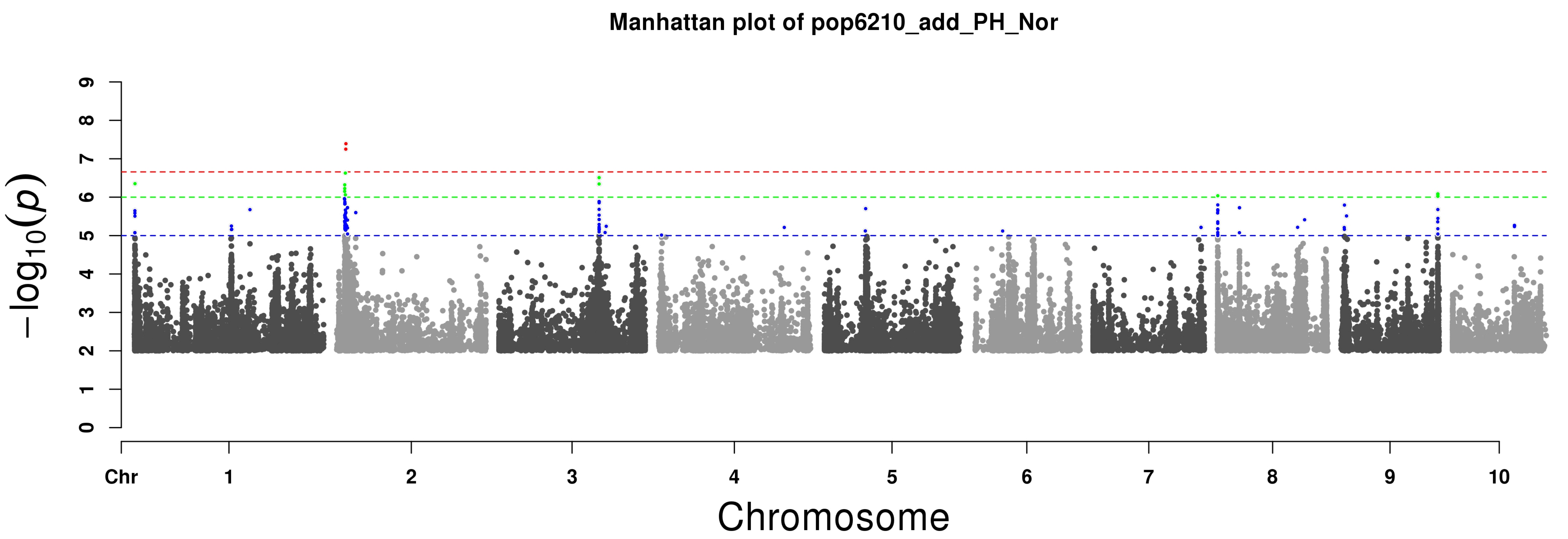
Manhattan plot of pop6210_add_PH_Nor
$-\log_{10}(p)$
0
1
2
3
4
5
6
7
8
9
Chr
1
2
3
4
5
6
7
8
9
10
Chromosome

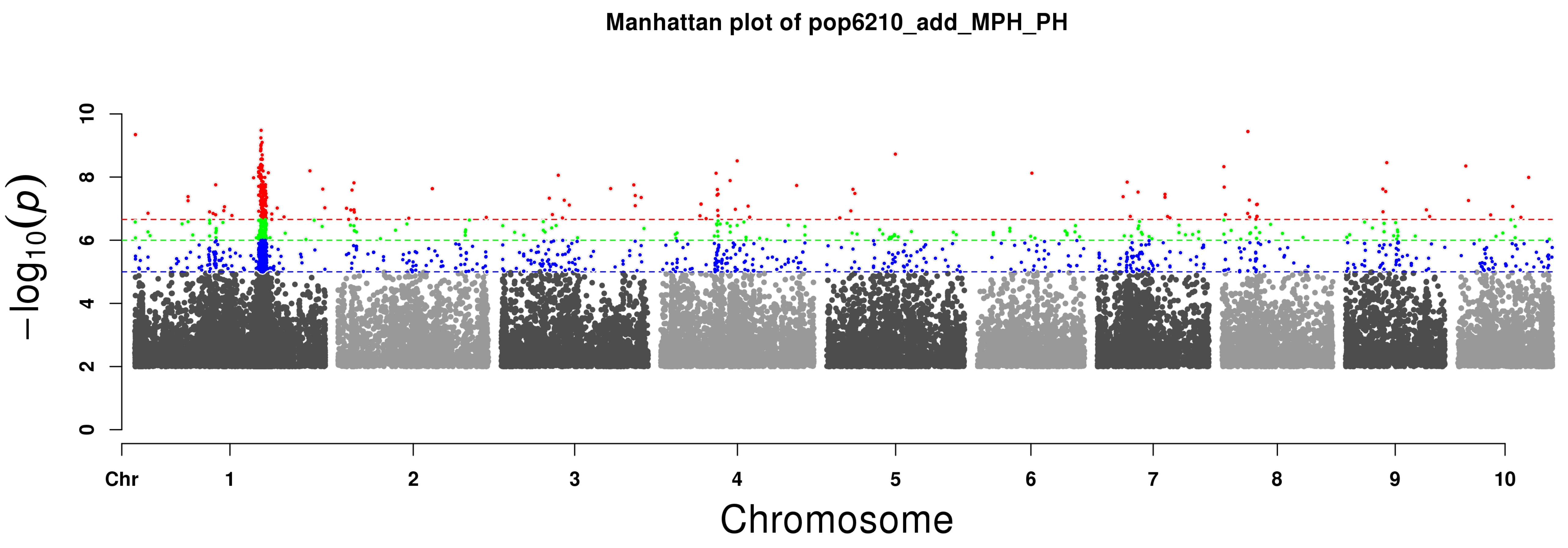
Manhattan plot of pop6210_add_MPH_PH
$-\log_{10}(p)$
0
2
4
6
8
10
Chr
1
2
3
4
5
6
7
8
9
10
Chromosome

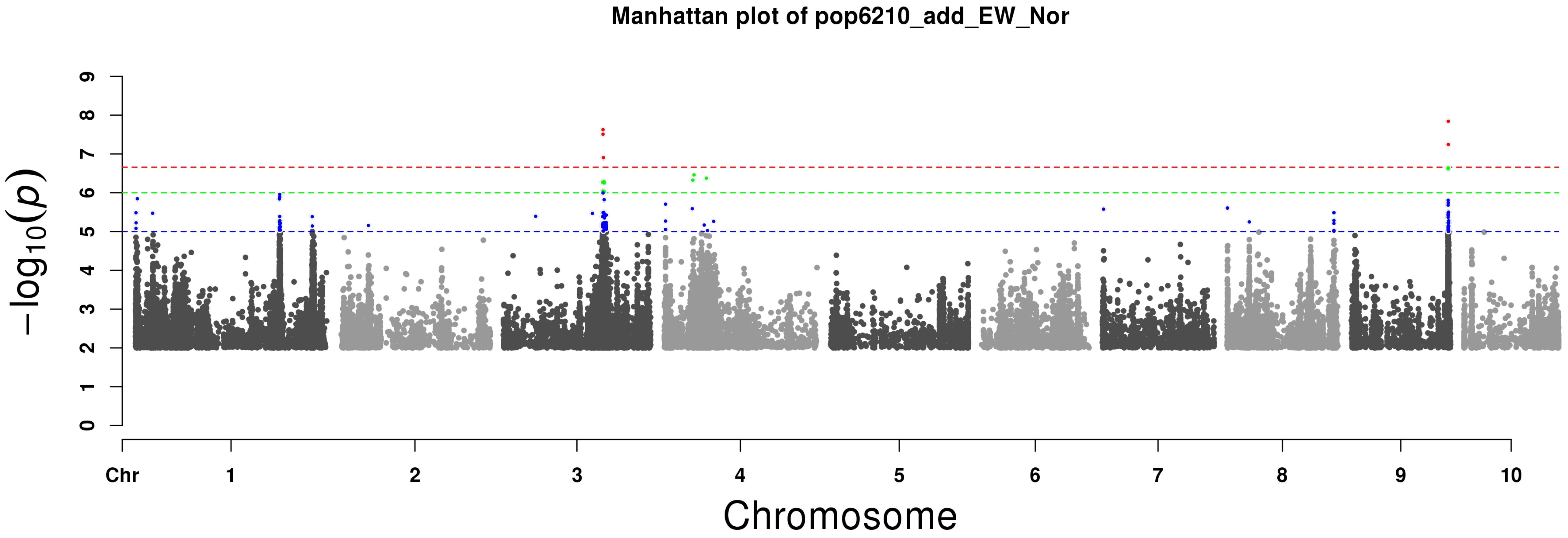
Manhattan plot of pop6210_add_EW_Nor
$-\log_{10}(p)$
0
1
2
3
4
5
6
7
8
9
Chr
1
2
3
4
5
6
7
8
9
10
Chromosome

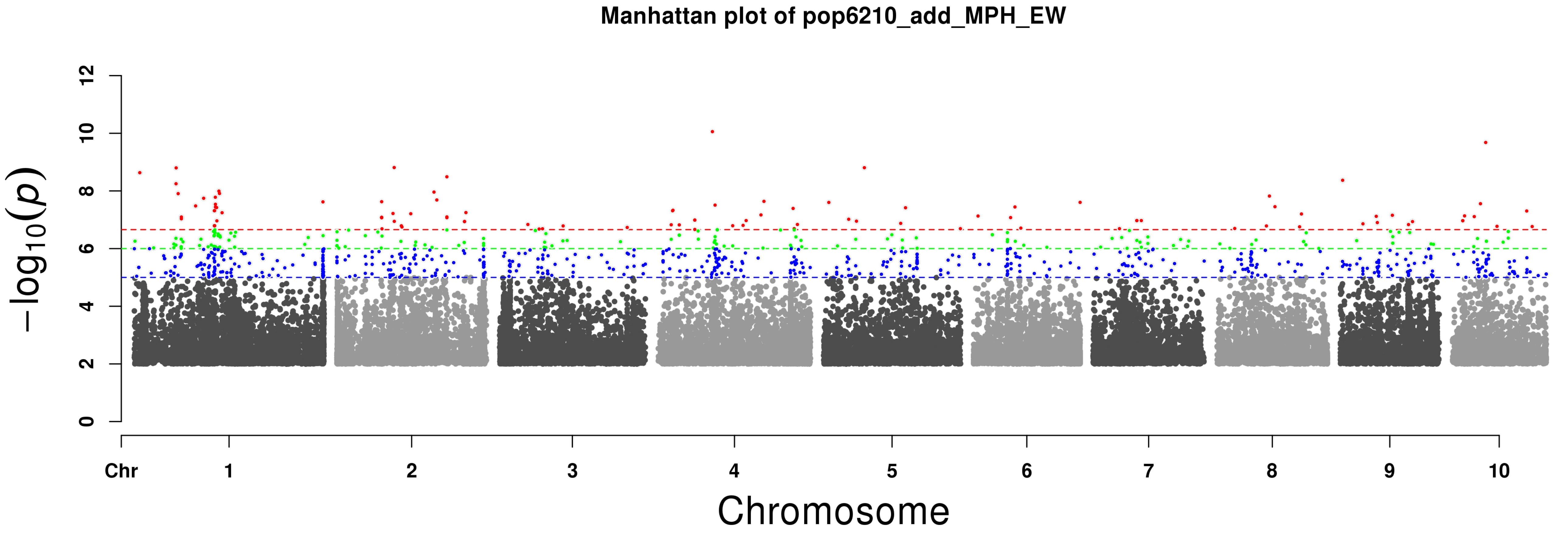

Manhattan plot of pop6210_add_MPH_EW
$-\log_{10}(p)$
0
2
4
6
8
10
12
Chr
1
2
3
4
5
6
7
8
9
10
Chromosome

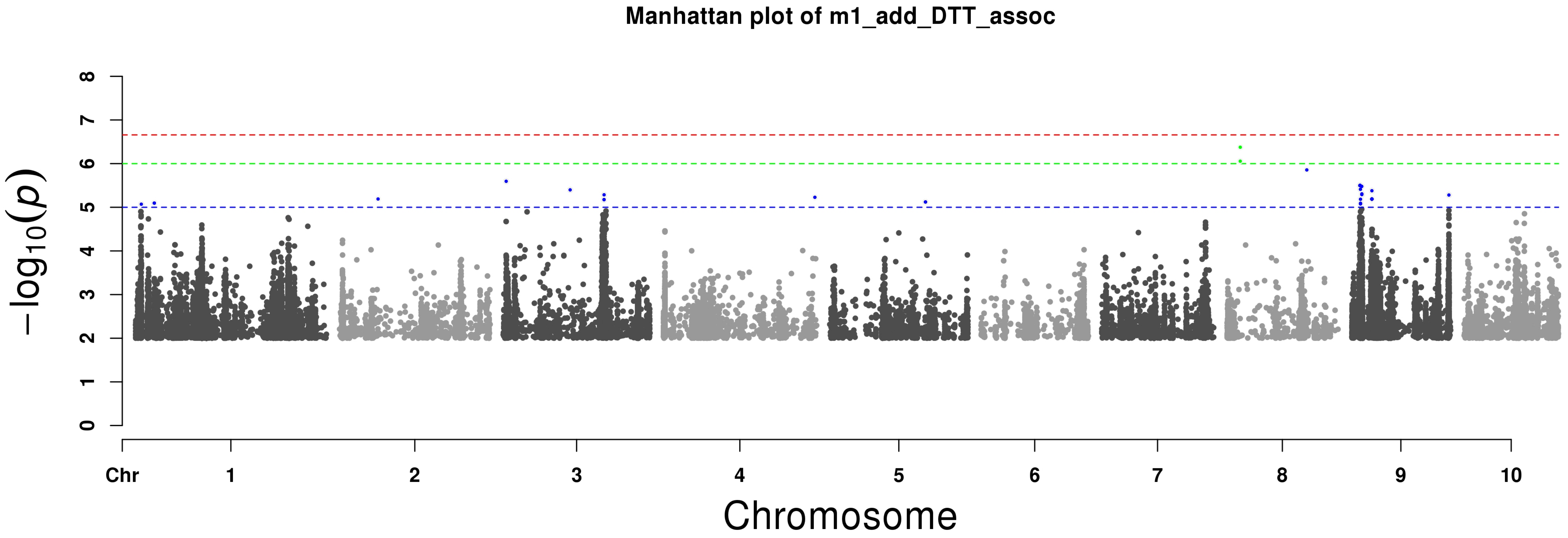

Manhattan plot of m1_add_DTT_assoc
$-\log_{10}(p)$
0
1
2
3
4
5
6
7
8
Chr
1
2
3
4
5
6
7
8
9
10
Chromosome

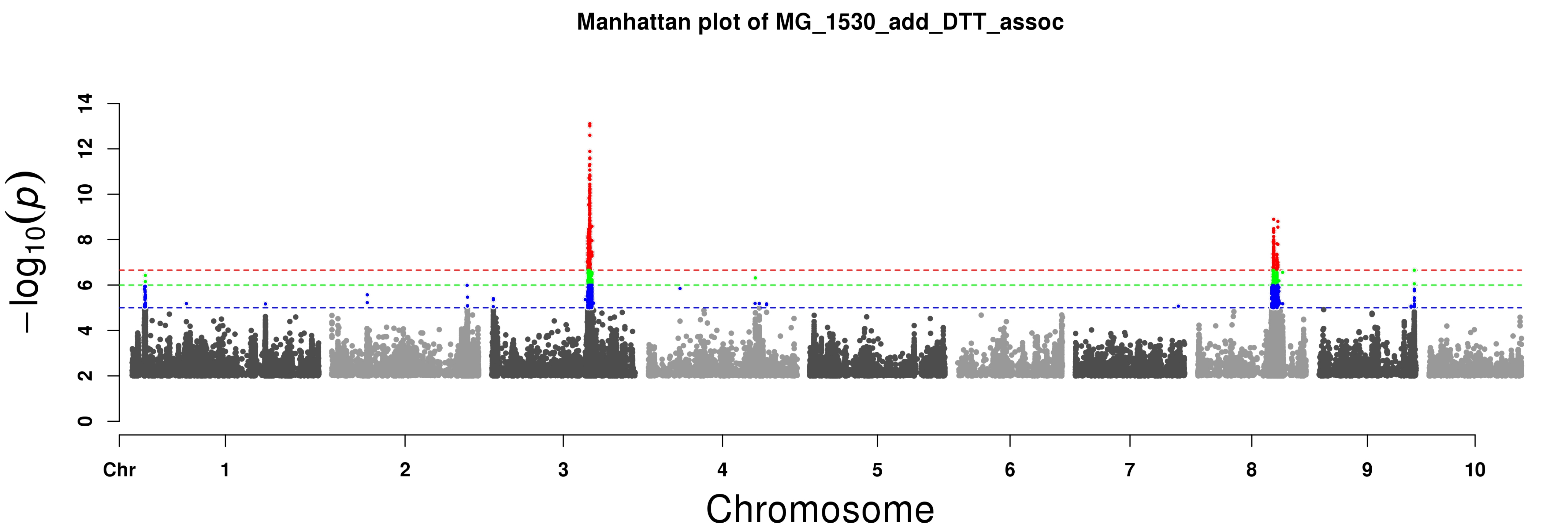

Manhattan plot of MG_1530_add_DTT_assoc
$-\log_{10}(p)$
0
2
4
6
8
10
12
14
Chr
1
2
3
4
5
6
7
8
9
10
Chromosome

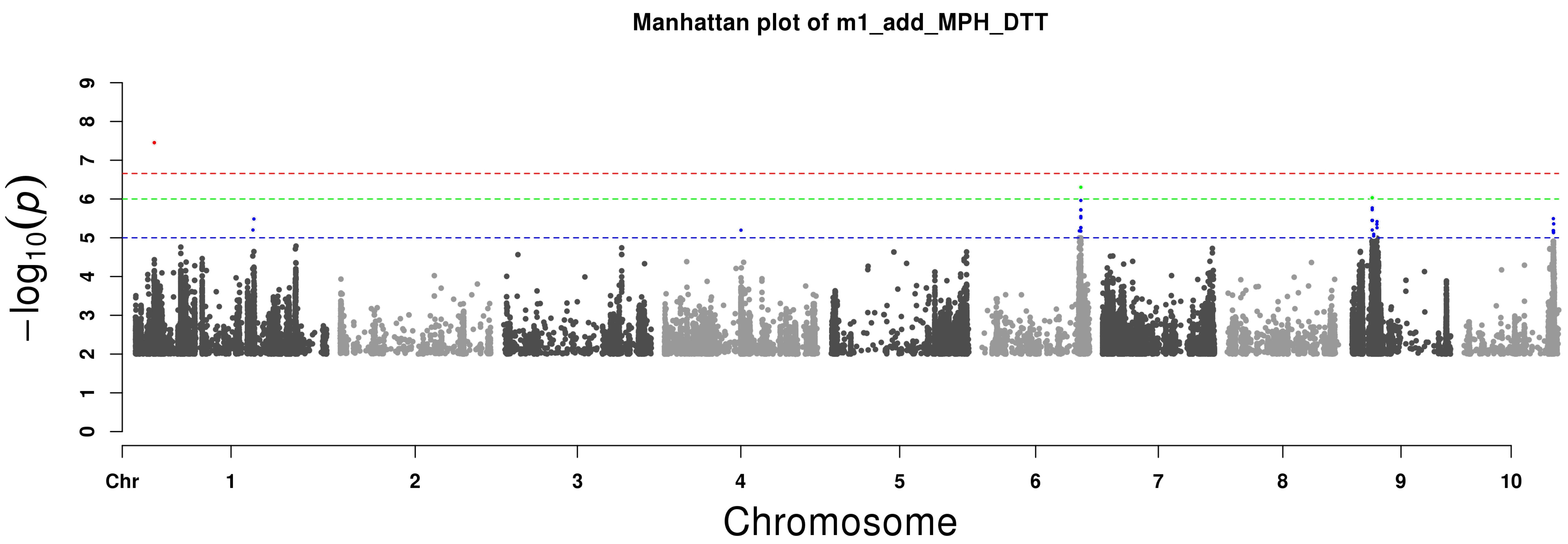
Manhattan plot of m1_add_MPH_DTT
$-\log_{10}(p)$
0
1
2
3
4
5
6
7
8
9
Chr
1
2
3
4
5
6
7
8
9
10
Chromosome

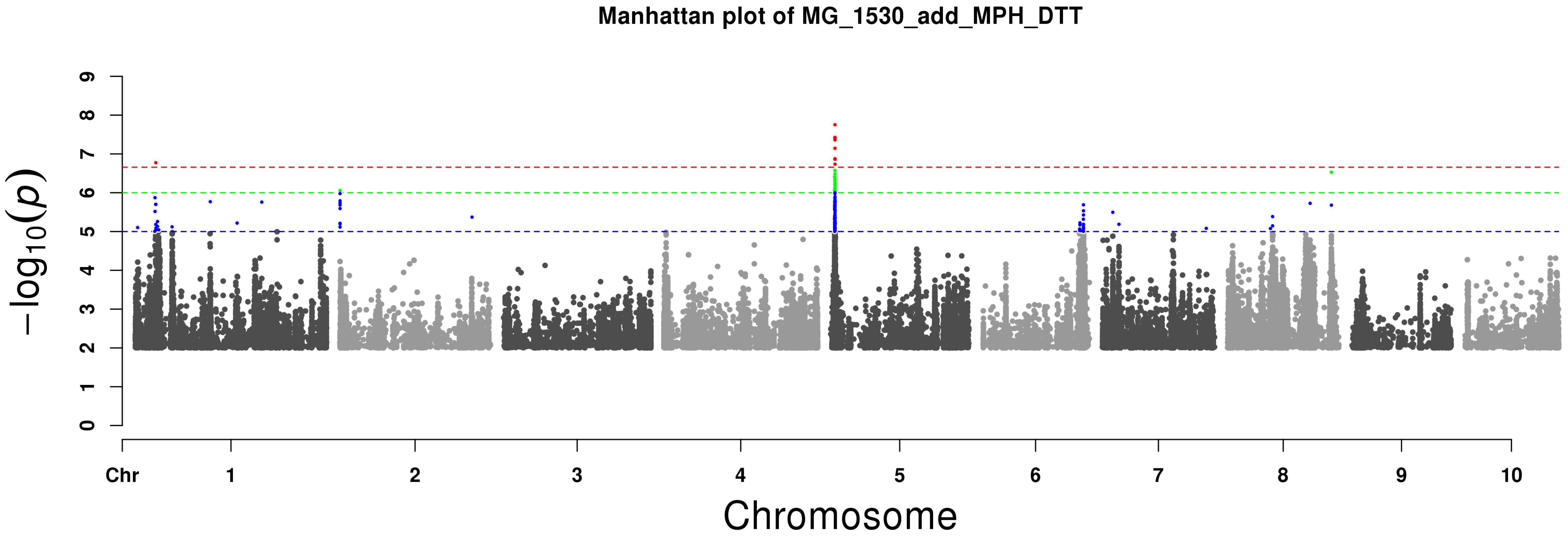

Manhattan plot of MG_1530_add_MPH_DTT
$-\log_{10}(p)$
0
1
2
3
4
5
6
7
8
9
Chr
1
2
3
4
5
6
7
8
9
10
Chromosome

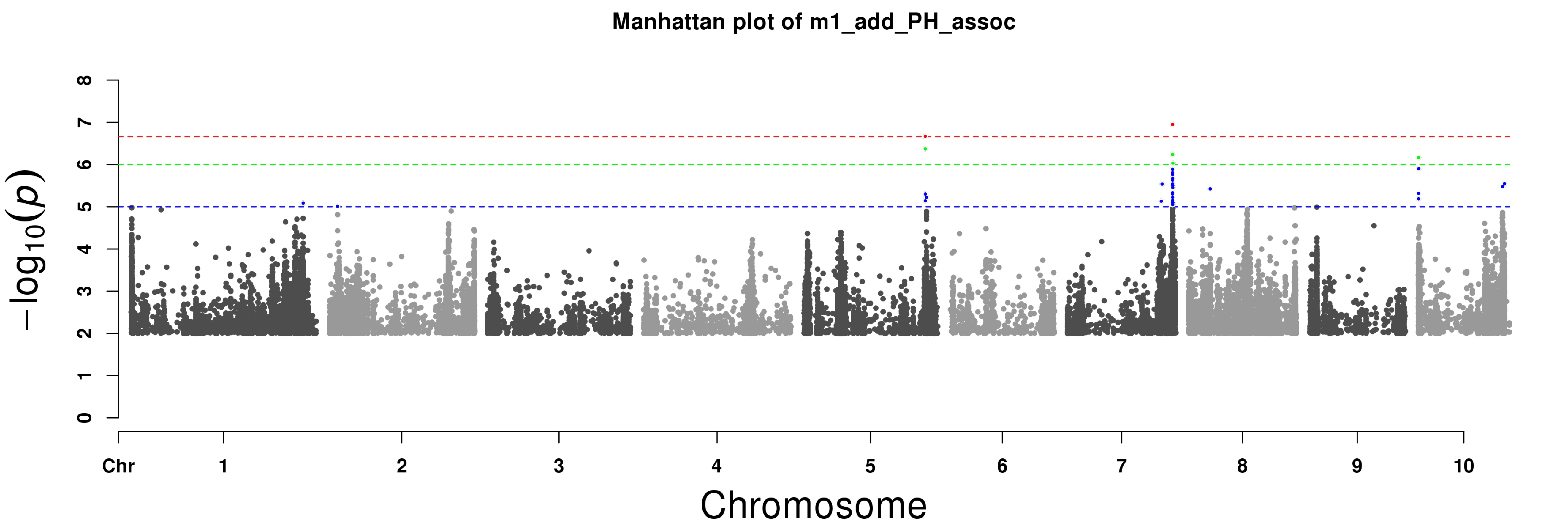
Manhattan plot of m1_add_PH_assoc
−log10(p)
0
1
2
3
4
5
6
7
8
Chr
1
2
3
4
5
6
7
8
9
10
Chromosome

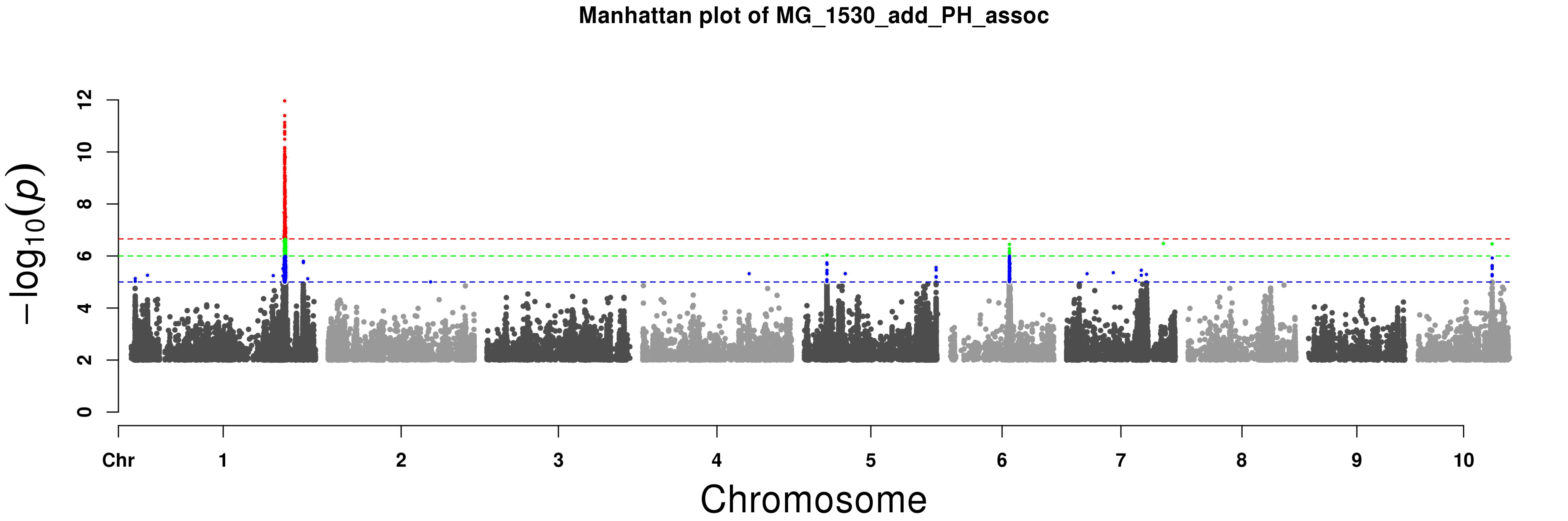
Manhattan plot of MG_1530_add_PH_assoc
−log10(p)
0
2
4
6
8
10
12
Chr
1
2
3
4
5
6
7
8
9
10
Chromosome

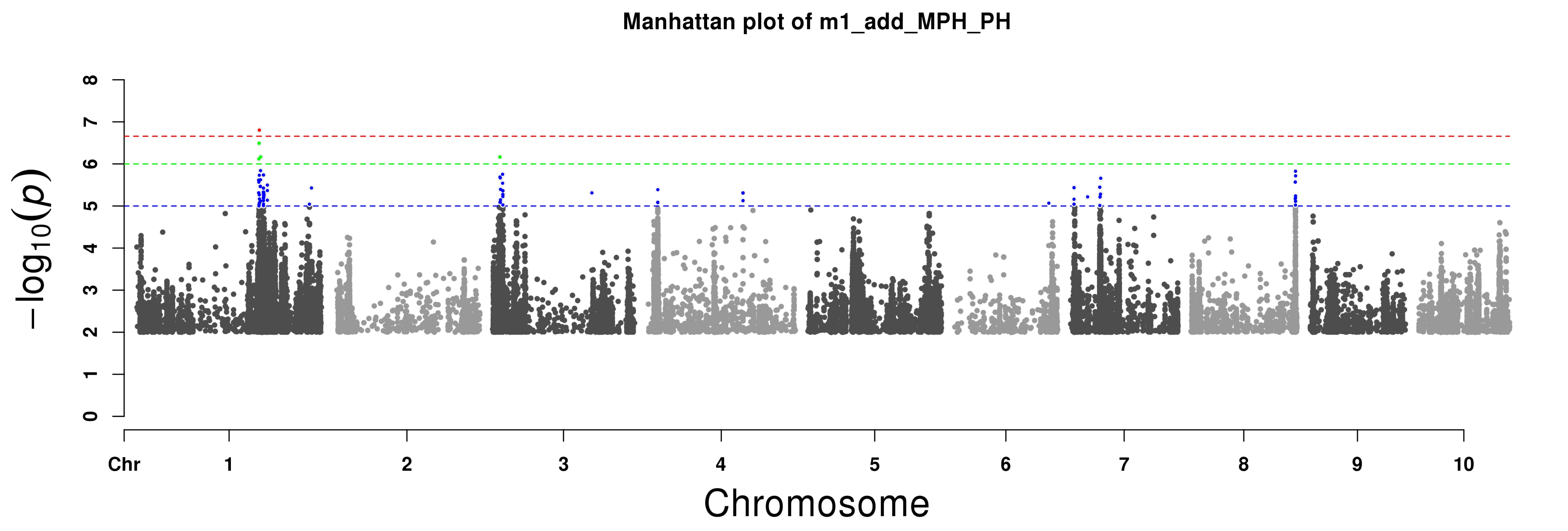

Manhattan plot of m1_add_MPH_PH
$-\log_{10}(p)$
0
1
2
3
4
5
6
7
8
Chr
1
2
3
4
5
6
7
8
9
10
Chromosome

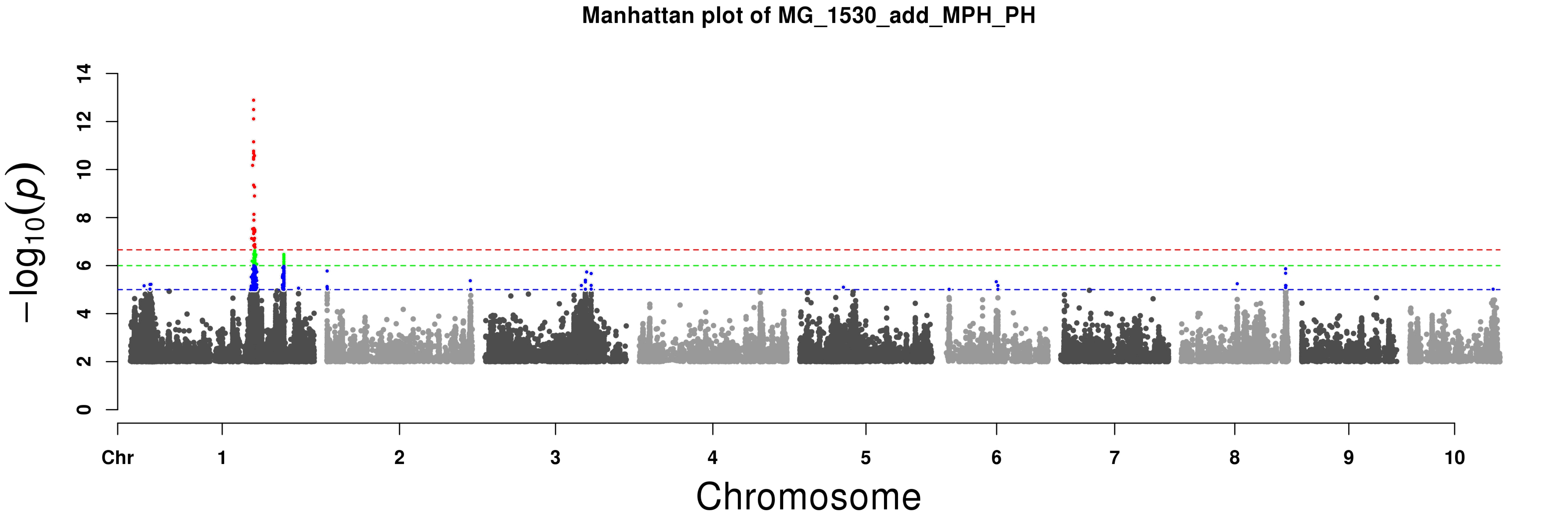

Manhattan plot of MG_1530_add_MPH_PH
$-\log_{10}(p)$
0
2
4
6
8
10
12
14
Chr
1
2
3
4
5
6
7
8
9
10
Chromosome

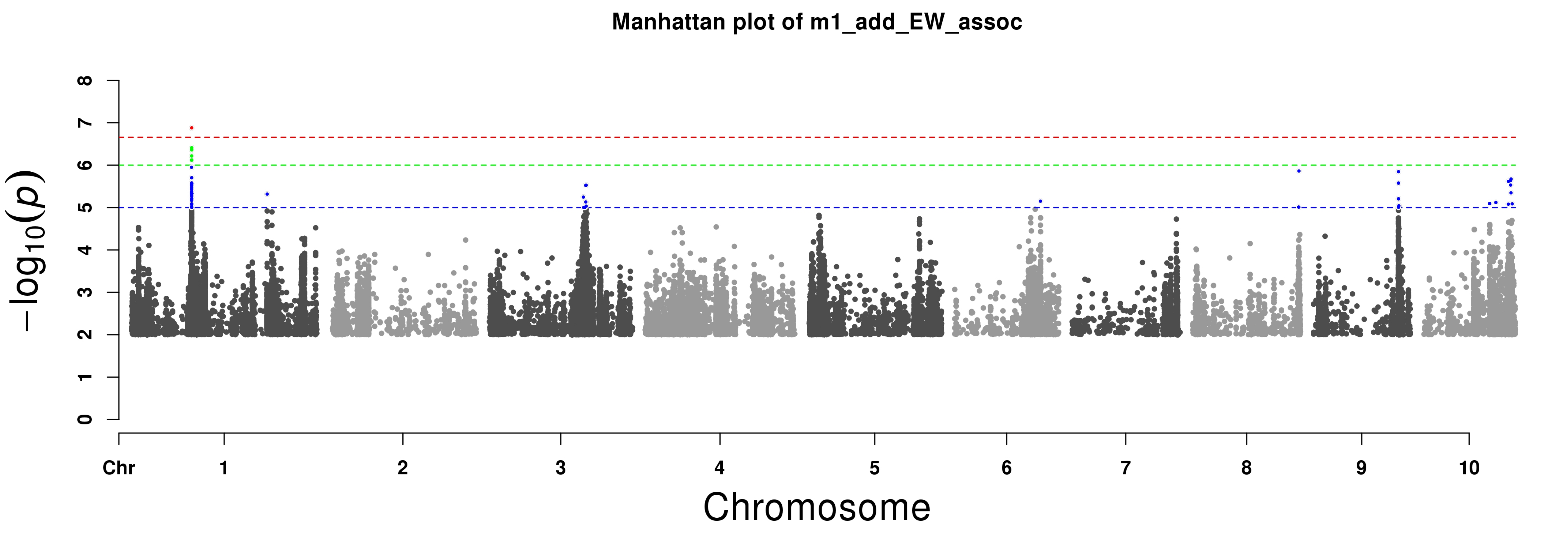

Manhattan plot of m1_add_EW_assoc
$-\log_{10}(p)$
0
1
2
3
4
5
6
7
8
Chr
1
2
3
4
5
6
7
8
9
10
Chromosome

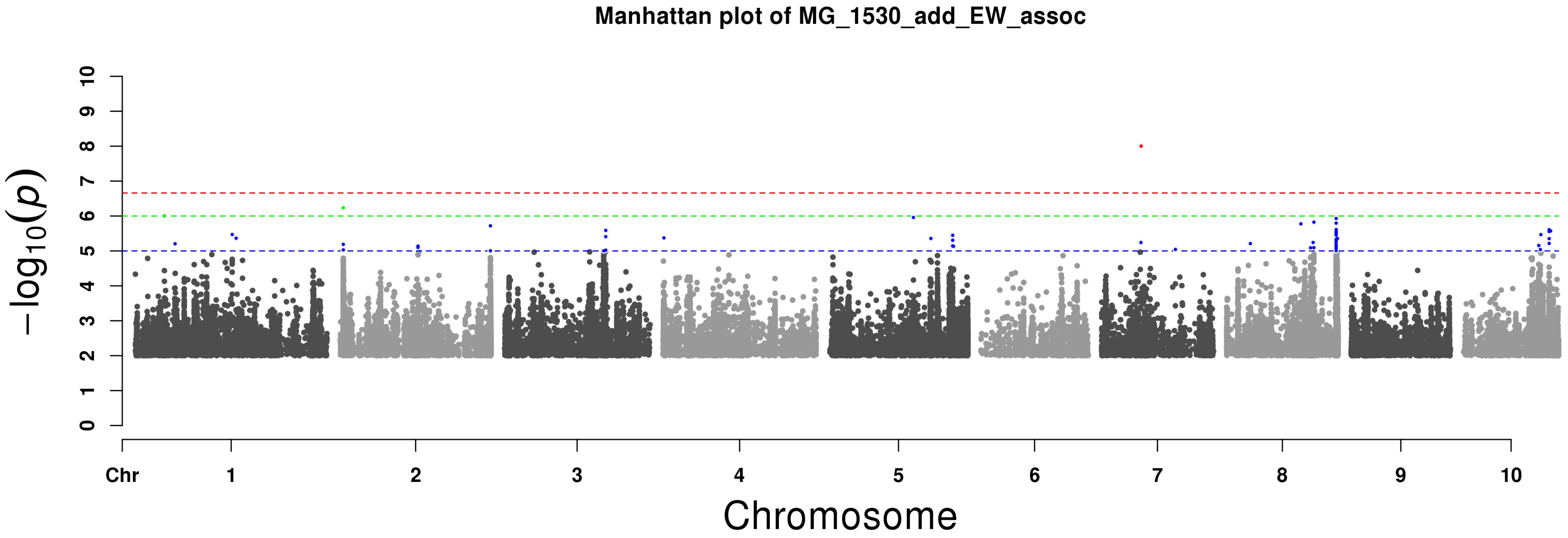

Manhattan plot of MG_1530_add_EW_assoc
−log10(p)
0
1
2
3
4
5
6
7
8
9
10
Chr
1
2
3
4
5
6
7
8
9
10
Chromosome

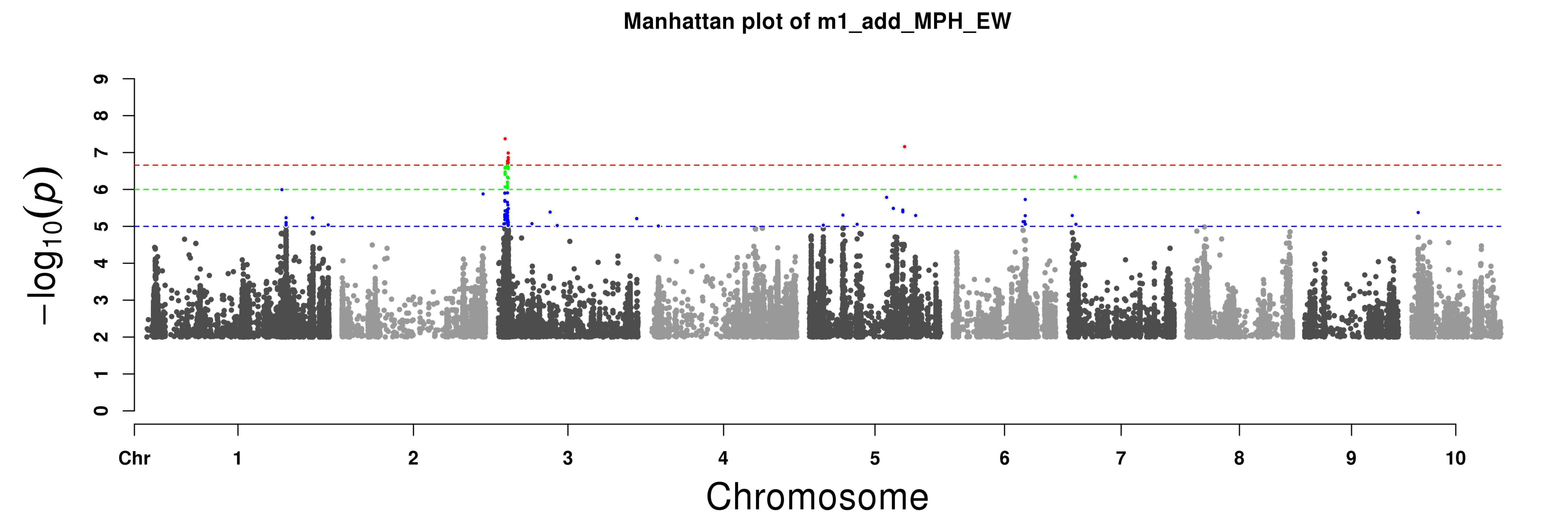
Manhattan plot of m1_add_MPH_EW
$-\log_{10}(p)$
0
1
2
3
4
5
6
7
8
9
Chr
1
2
3
4
5
6
7
8
9
10
Chromosome

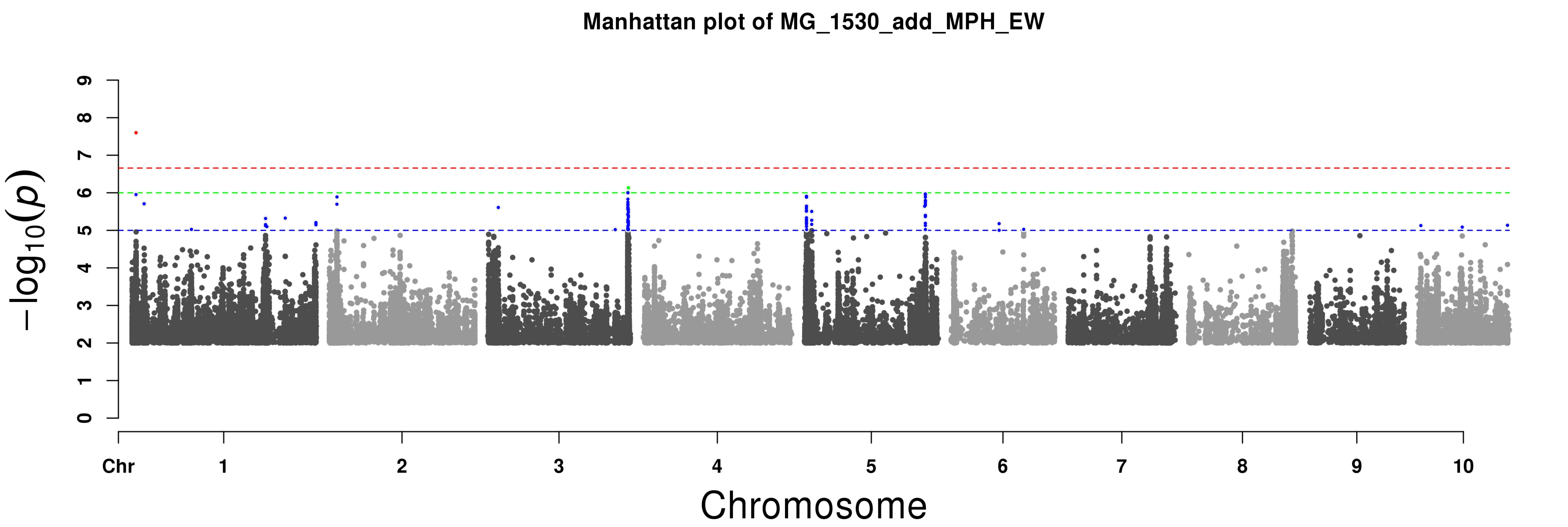
Manhattan plot of MG_1530_add_MPH_EW
$-\log_{10}(p)$
0
1
2
3
4
5
6
7
8
9
Chr
1
2
3
4
5
6
7
8
9
10
Chromosome

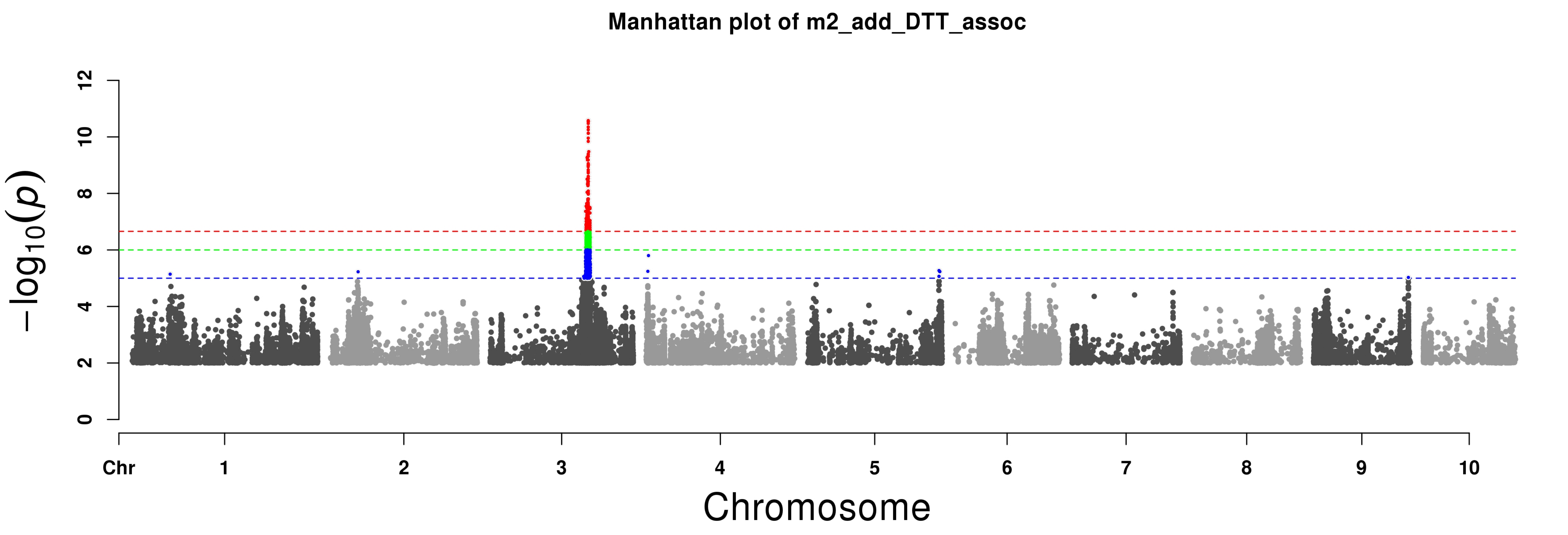

Manhattan plot of m2_add_DTT_assoc
$-\log_{10}(p)$
0
2
4
6
8
10
12
Chr
1
2
3
4
5
6
7
8
9
10
Chromosome

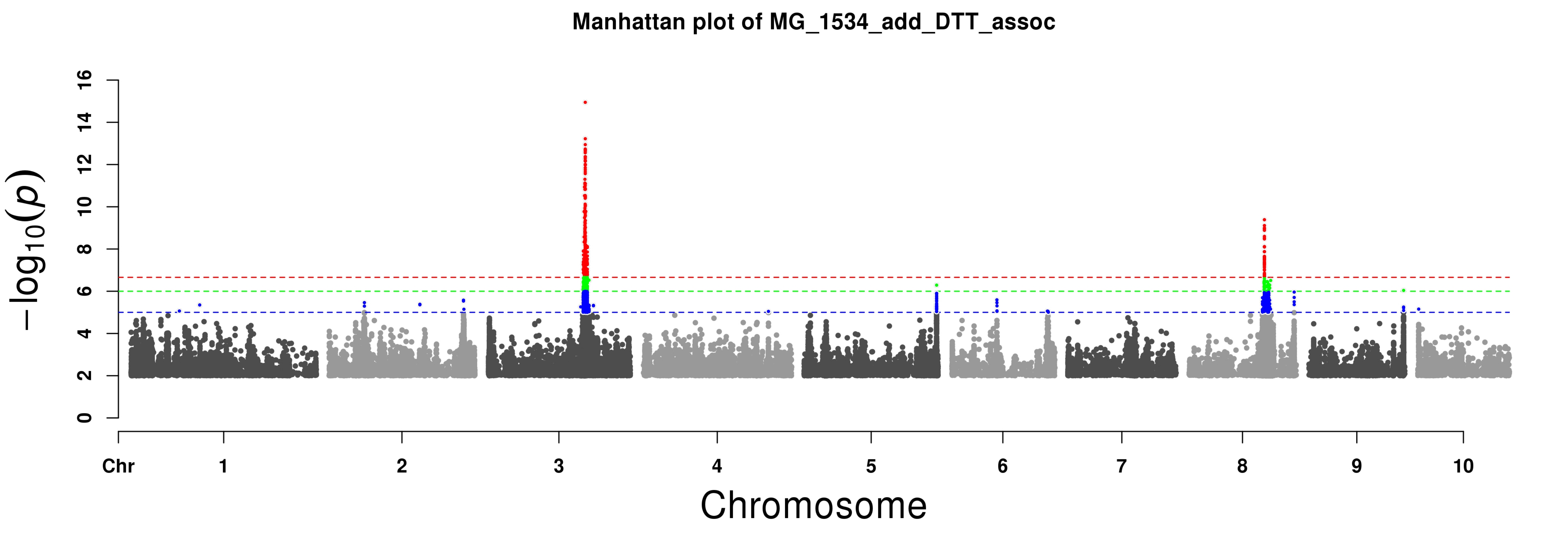

Manhattan plot of MG_1534_add_DTT_assoc
$-\log_{10}(p)$
0
2
4
6
8
10
12
14
16
Chr
1
2
3
4
5
6
7
8
9
10
Chromosome

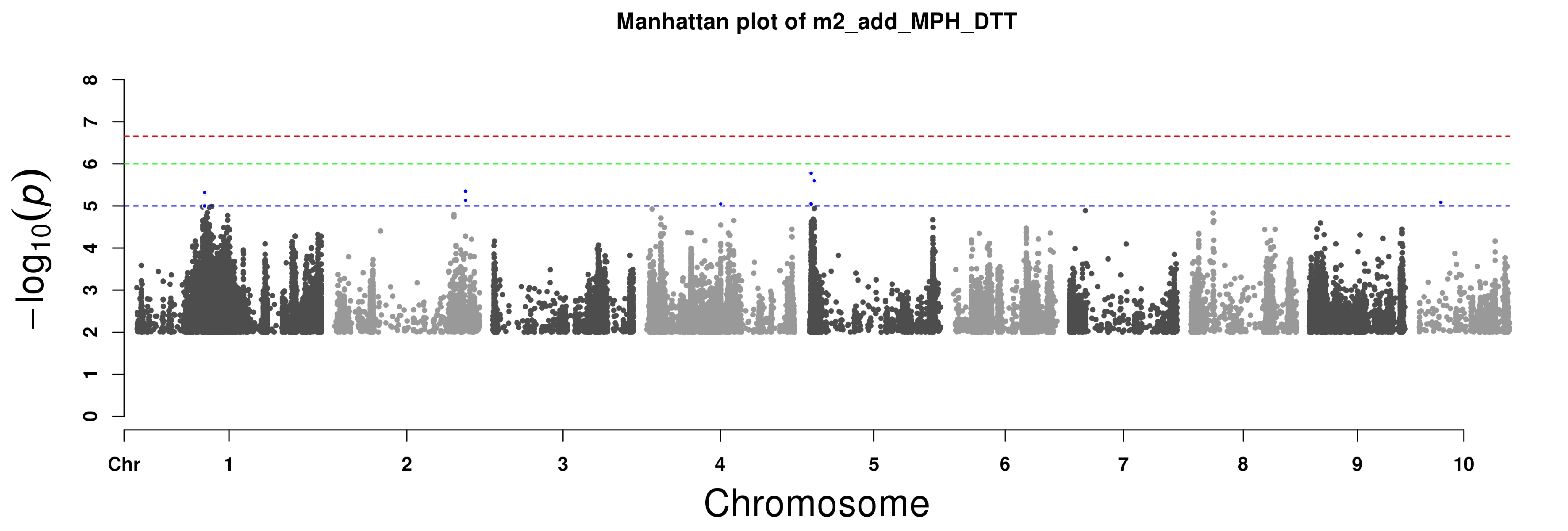

Manhattan plot of m2_add_MPH_DTT
$-\log_{10}(p)$
0
1
2
3
4
5
6
7
8
Chr
1
2
3
4
5
6
7
8
9
10
Chromosome

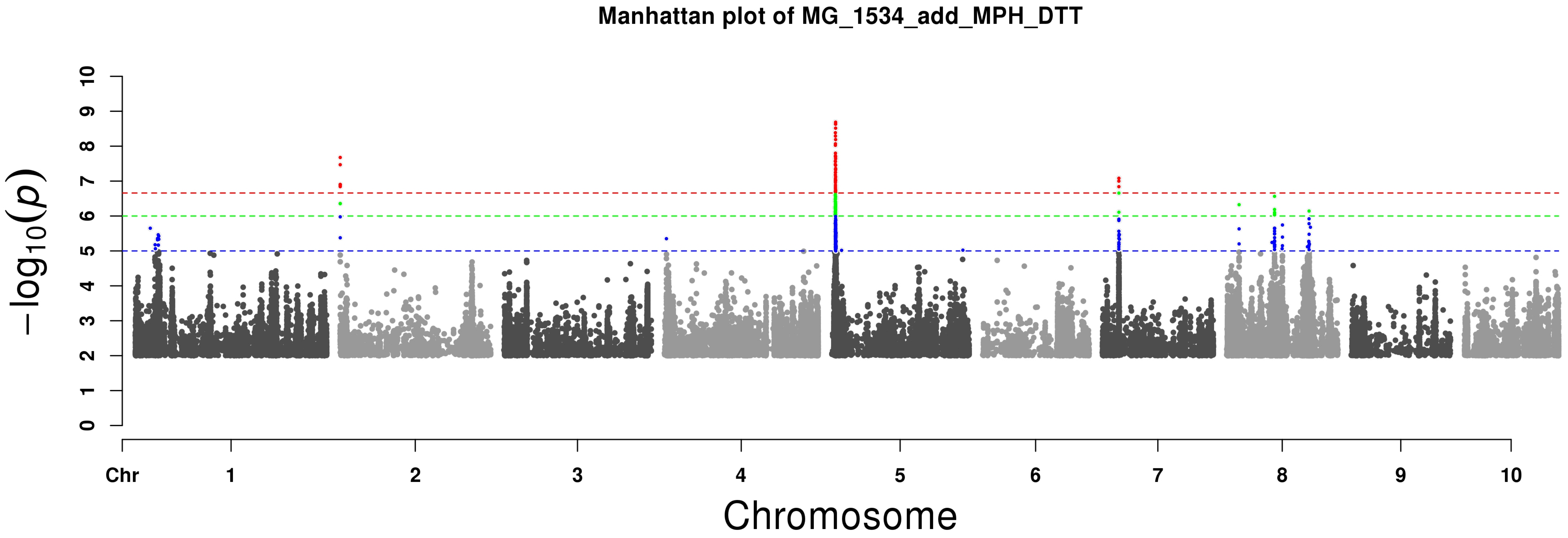
Manhattan plot of MG_1534_add_MPH_DTT
$-\log_{10}(p)$
0
1
2
3
4
5
6
7
8
9
10
Chr
1
2
3
4
5
6
7
8
9
10
Chromosome

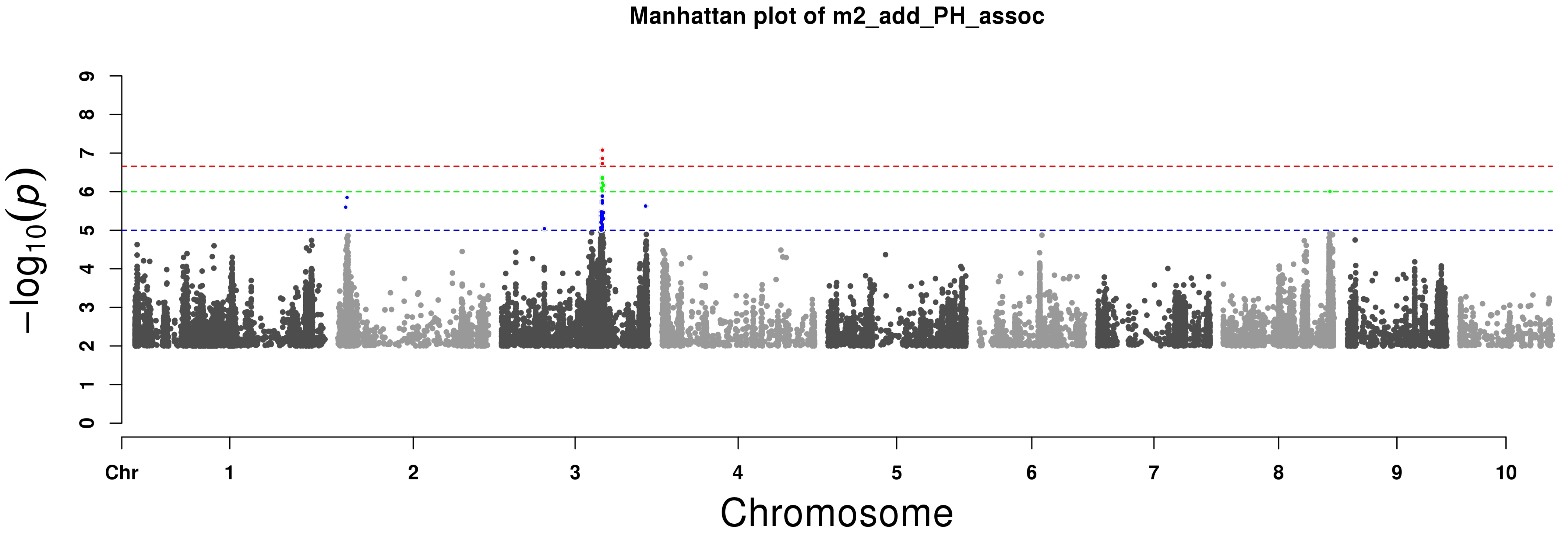
Manhattan plot of m2_add_PH_assoc
$-\log_{10}(p)$
0
1
2
3
4
5
6
7
8
9
Chr
1
2
3
4
5
6
7
8
9
10
Chromosome

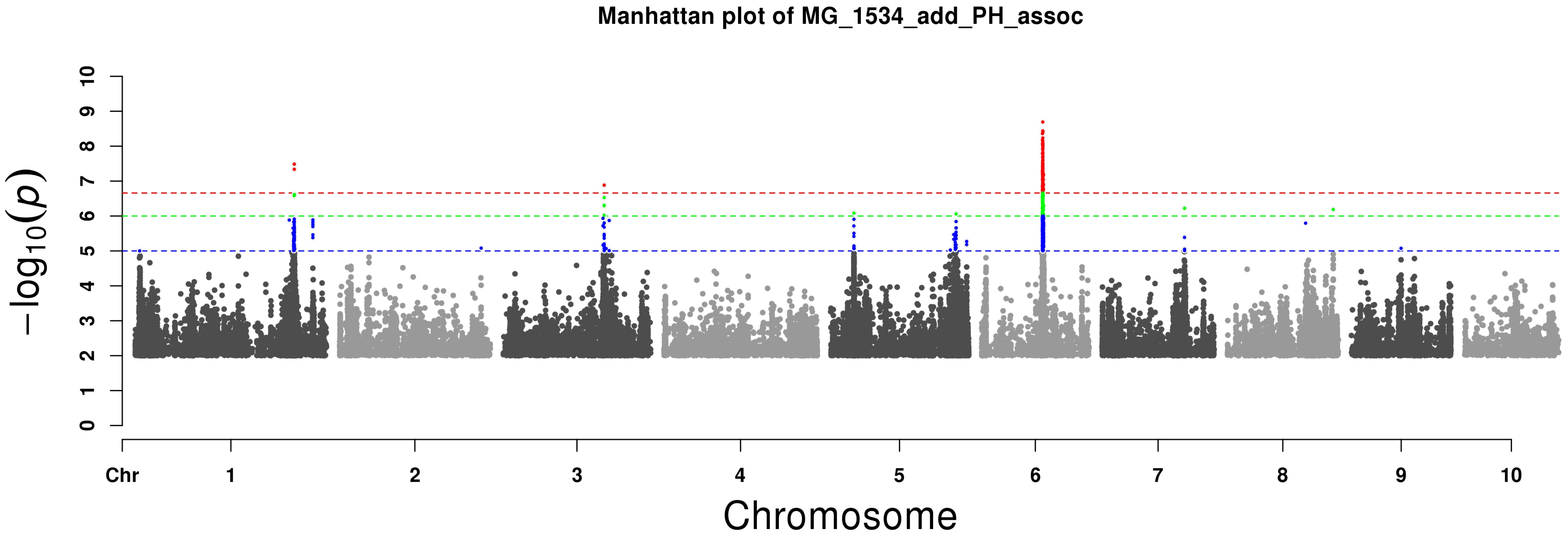

Manhattan plot of MG_1534_add_PH_assoc
$-\log_{10}(p)$
0
1
2
3
4
5
6
7
8
9
10
Chr
1
2
3
4
5
6
7
8
9
10
Chromosome

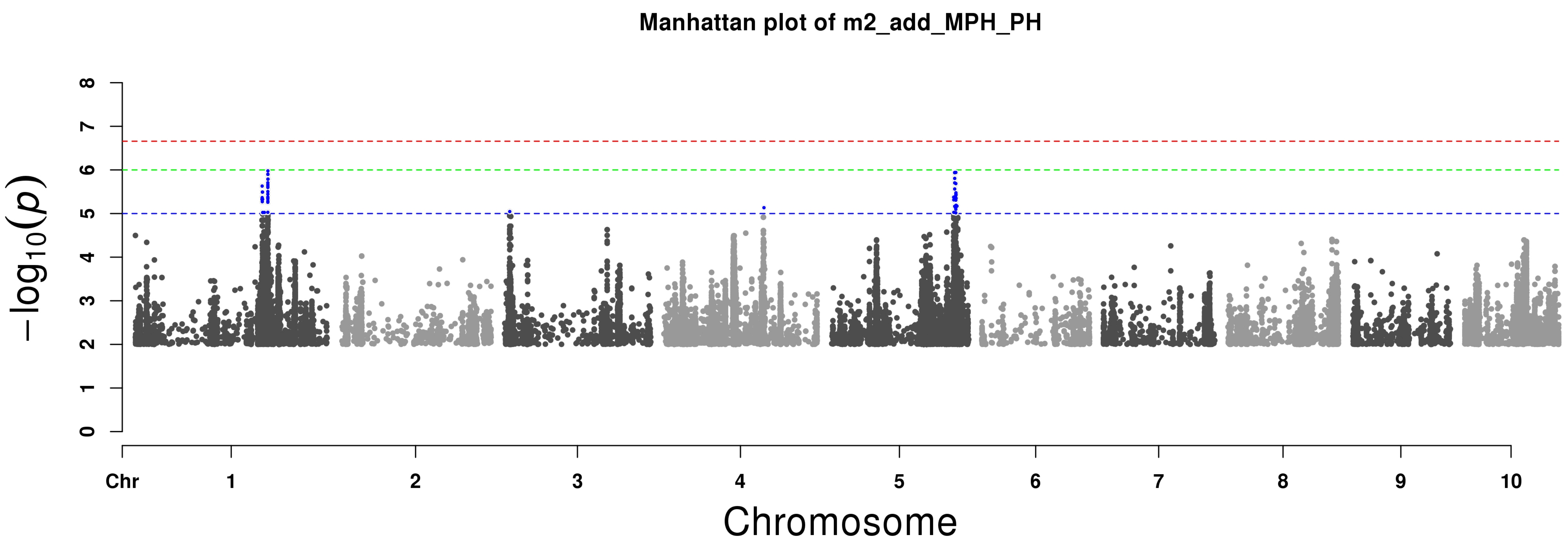

Manhattan plot of m2_add_MPH_PH
$-\log_{10}(p)$
0
1
2
3
4
5
6
7
8
Chr
1
2
3
4
5
6
7
8
9
10
Chromosome

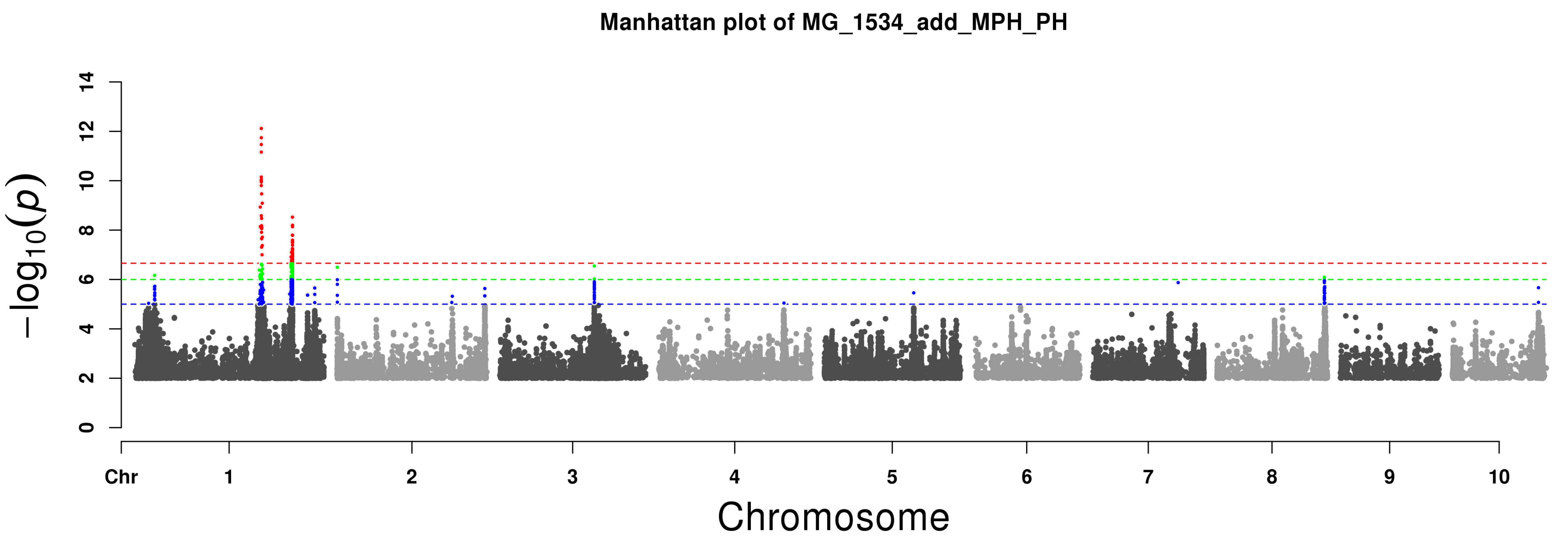
Manhattan plot of MG_1534_add_MPH_PH
$-\log_{10}(p)$
0
2
4
6
8
10
12
14
Chr
1
2
3
4
5
6
7
8
9
10
Chromosome

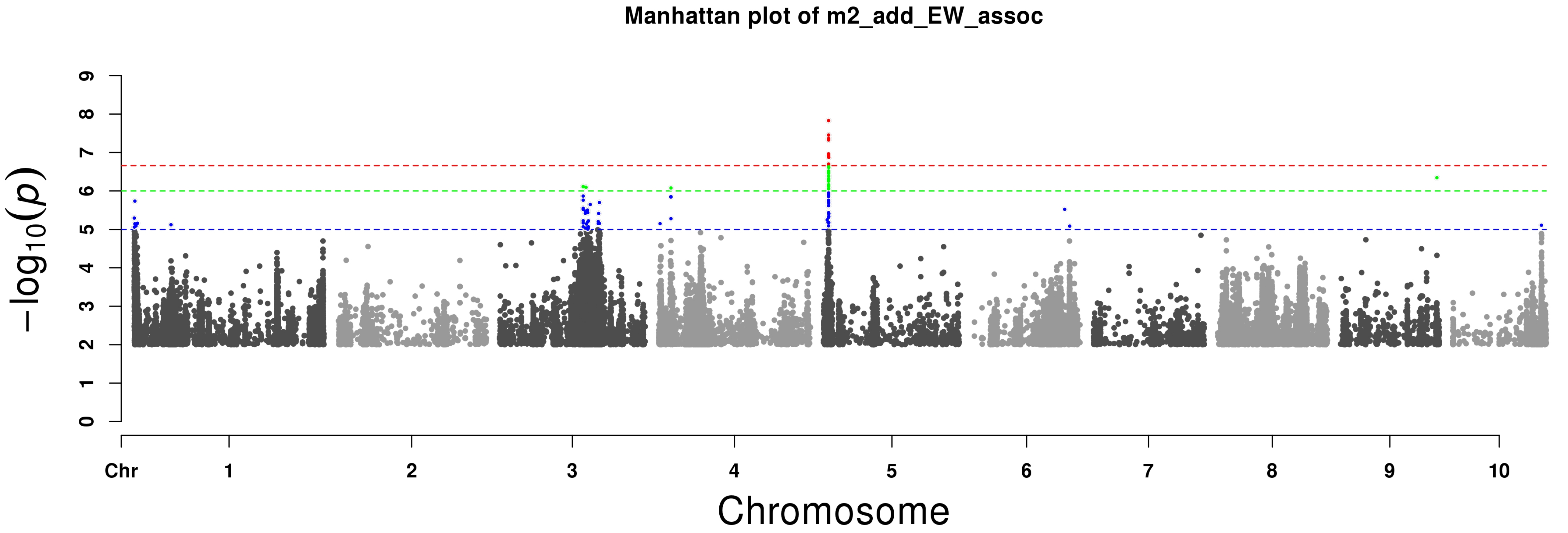

Manhattan plot of m2_add_EW_assoc
$-\log_{10}(p)$
0
1
2
3
4
5
6
7
8
9
Chr
1
2
3
4
5
6
7
8
9
10
Chromosome

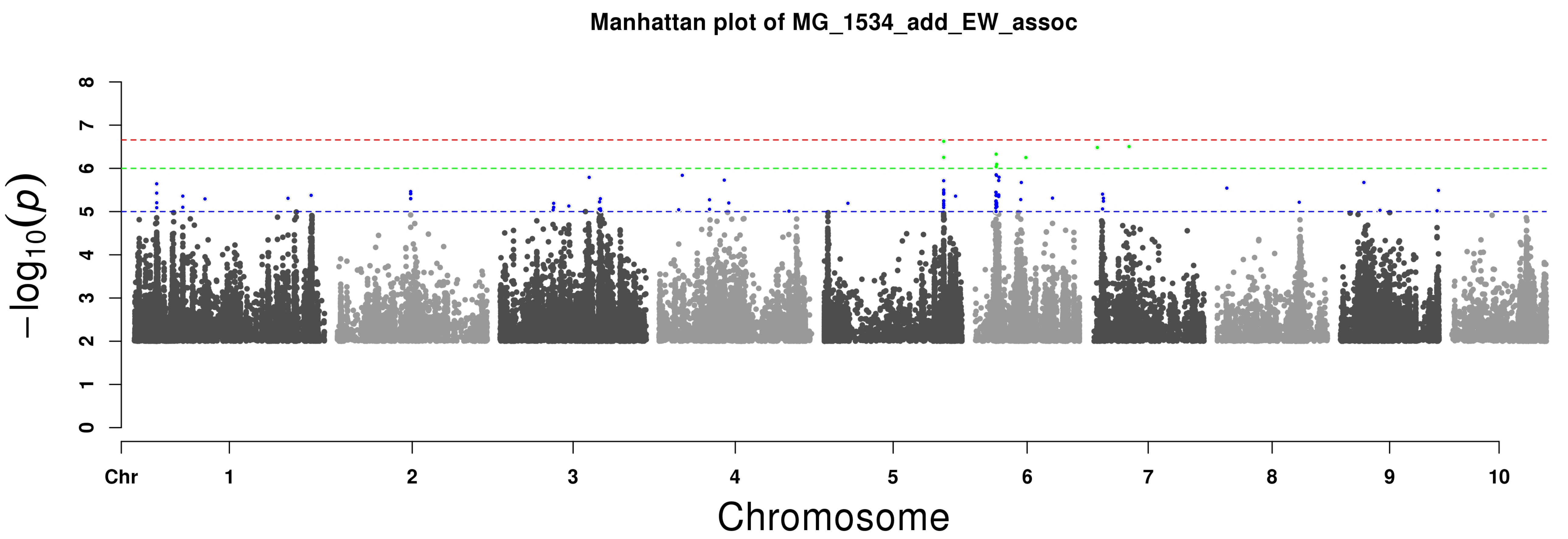

Manhattan plot of MG_1534_add_EW_assoc
$-\log_{10}(p)$
0
1
2
3
4
5
6
7
8
Chr
1
2
3
4
5
6
7
8
9
10
Chromosome

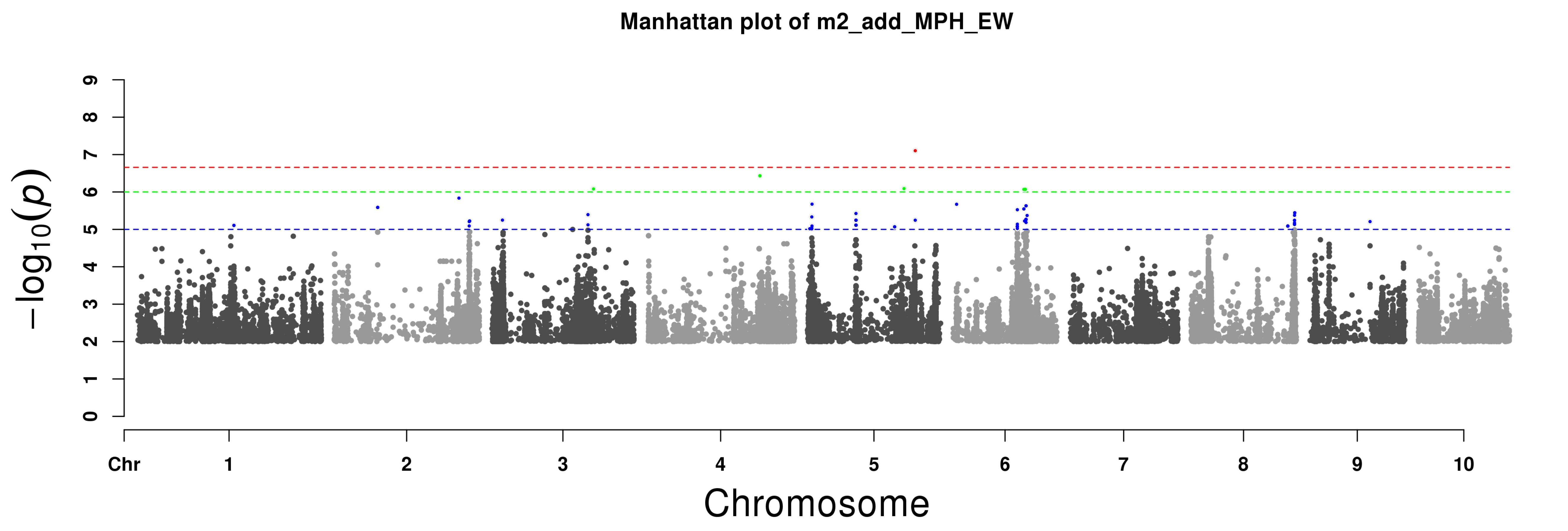

Manhattan plot of m2_add_MPH_EW
$-\log_{10}(p)$
0
1
2
3
4
5
6
7
8
9
Chr
1
2
3
4
5
6
7
8
9
10
Chromosome

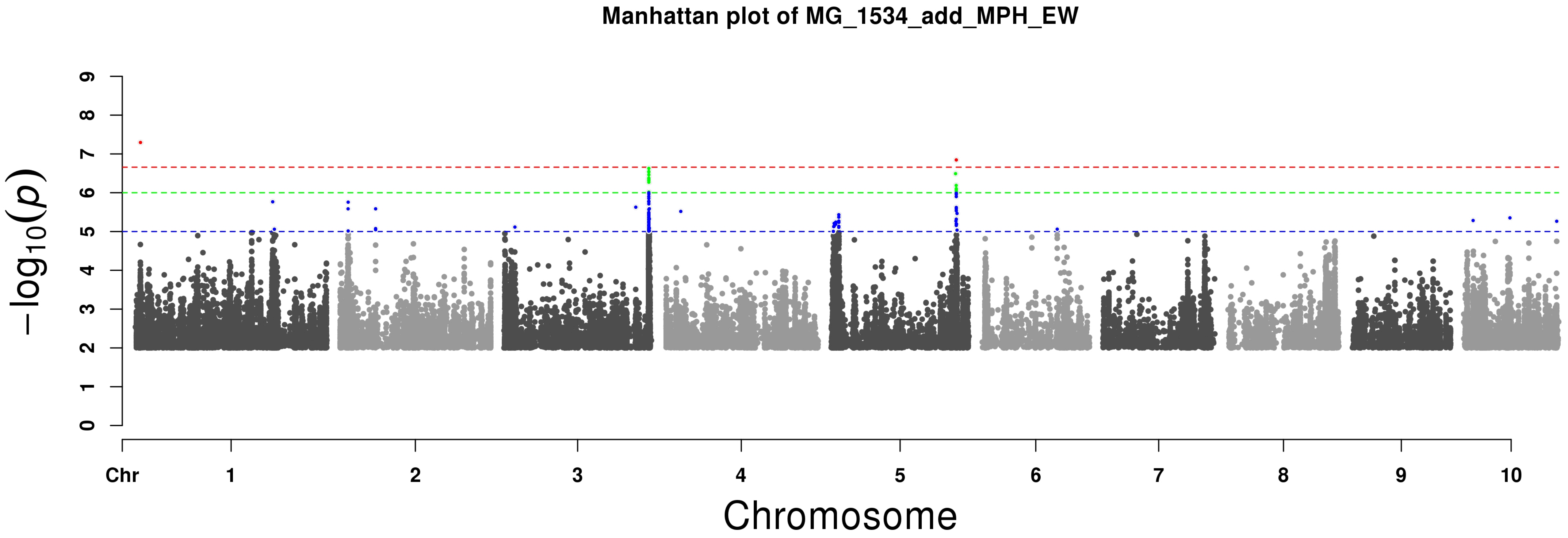

Manhattan plot of MG_1534_add_MPH_EW
$-\log_{10}(p)$
0
1
2
3
4
5
6
7
8
9
Chr
1
2
3
4
5
6
7
8
9
10
Chromosome

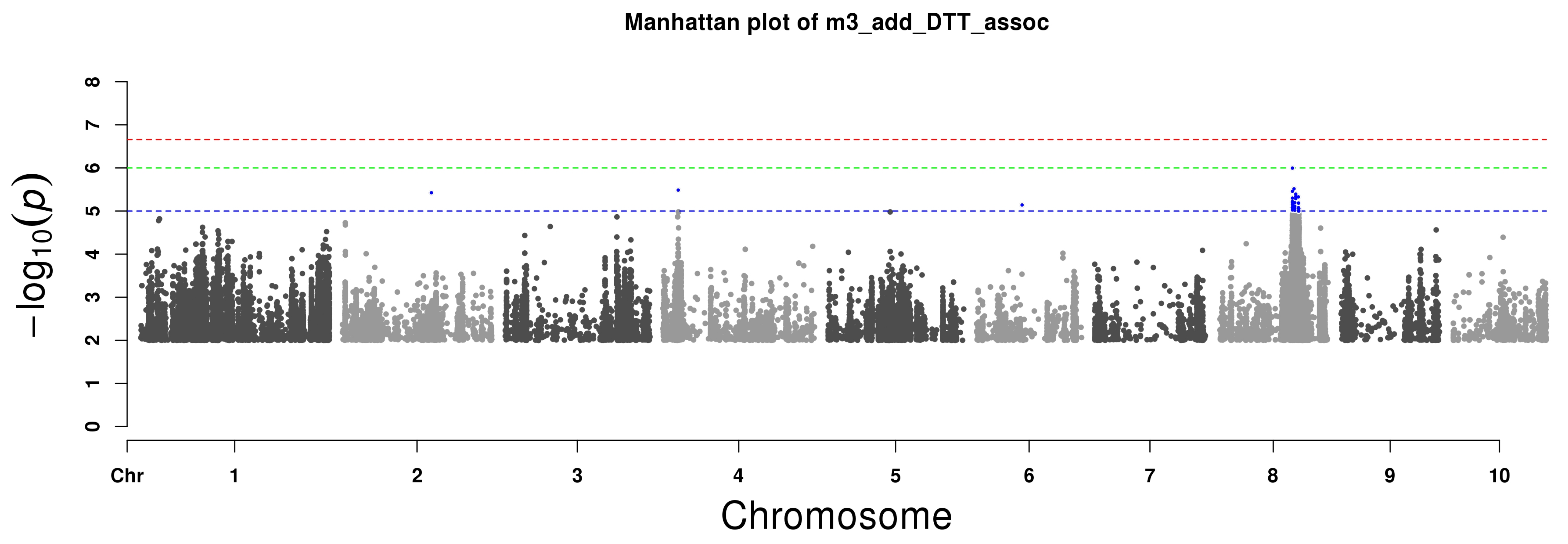

Manhattan plot of m3_add_DTT_assoc
$-\log_{10}(p)$
0
1
2
3
4
5
6
7
8
Chr
1
2
3
4
5
6
7
8
9
10
Chromosome

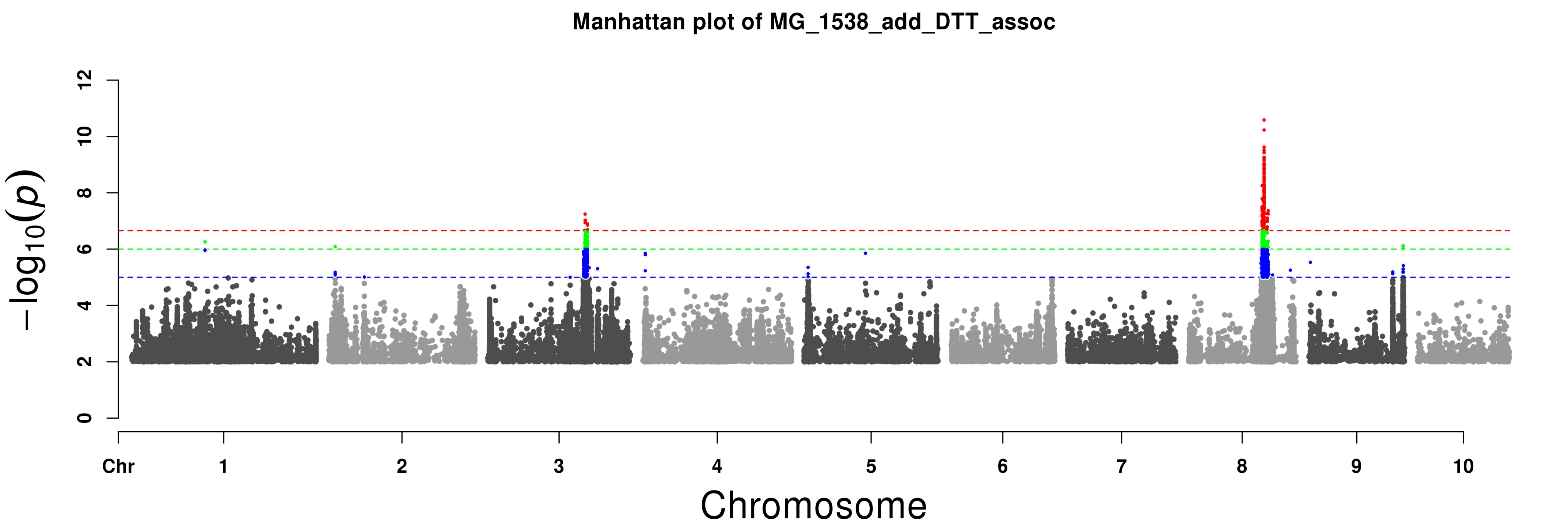

Manhattan plot of MG_1538_add_DTT_assoc
$-\log_{10}(p)$
0
2
4
6
8
10
12
Chr
1
2
3
4
5
6
7
8
9
10
Chromosome

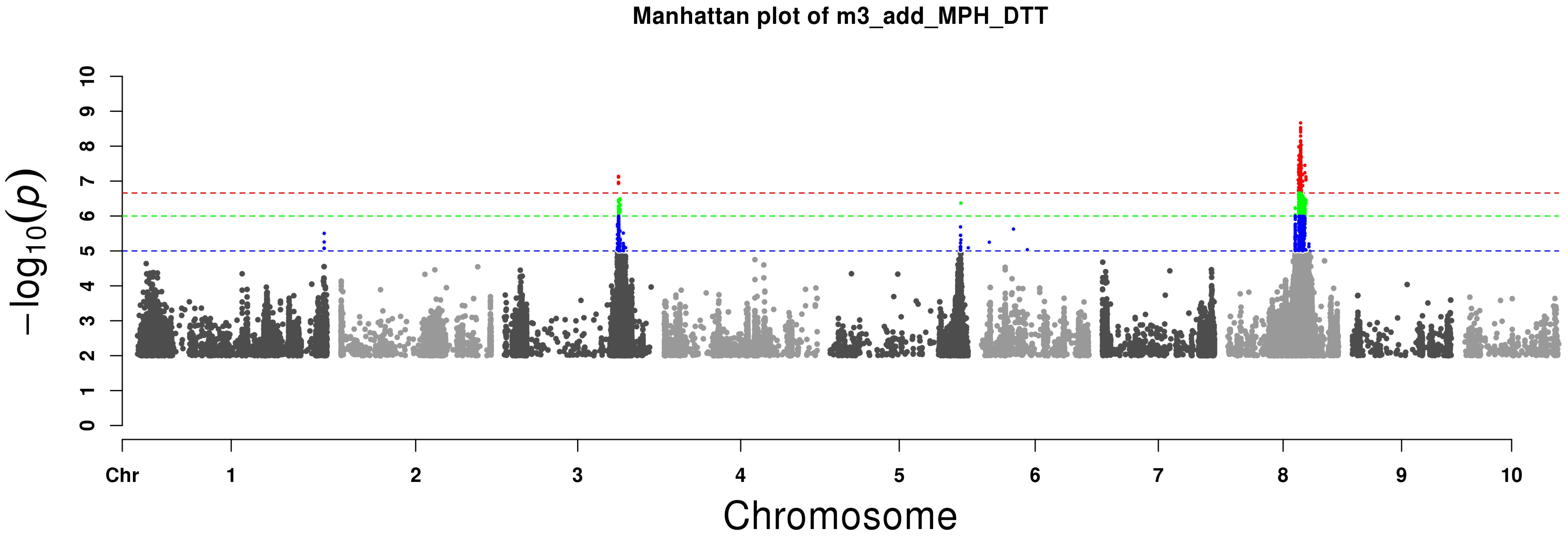

Manhattan plot of m3_add_MPH_DTT
$-\log_{10}(p)$
0
1
2
3
4
5
6
7
8
9
10
Chr
1
2
3
4
5
6
7
8
9
10
Chromosome

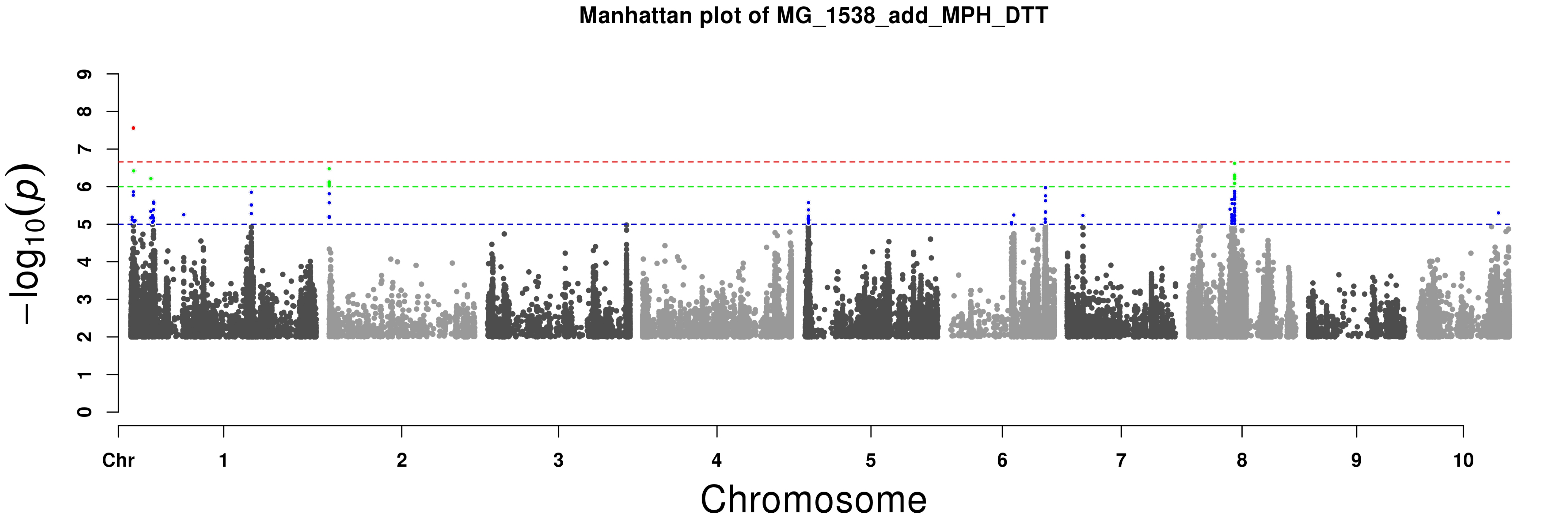
Manhattan plot of MG_1538_add_MPH_DTT
−log10(p)
0
1
2
3
4
5
6
7
8
9
Chr
1
2
3
4
5
6
7
8
9
10
Chromosome

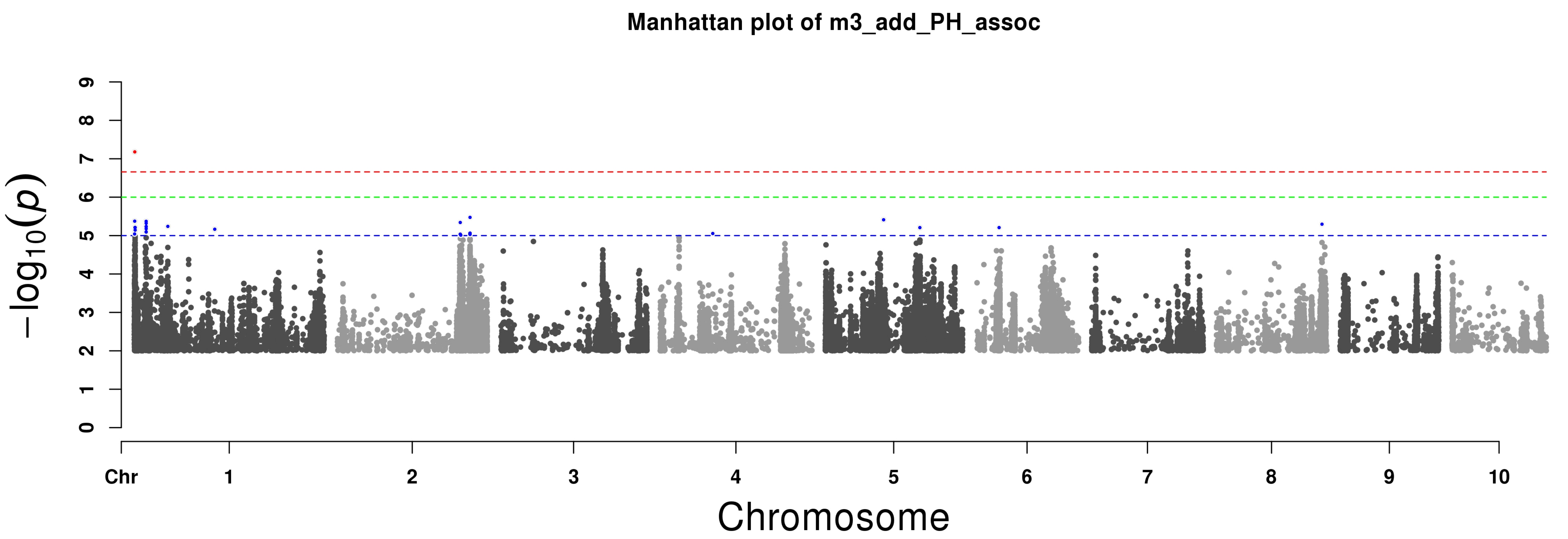
Manhattan plot of m3_add_PH_assoc
$-\log_{10}(p)$
0
1
2
3
4
5
6
7
8
9
Chr
1
2
3
4
5
6
7
8
9
10
Chromosome

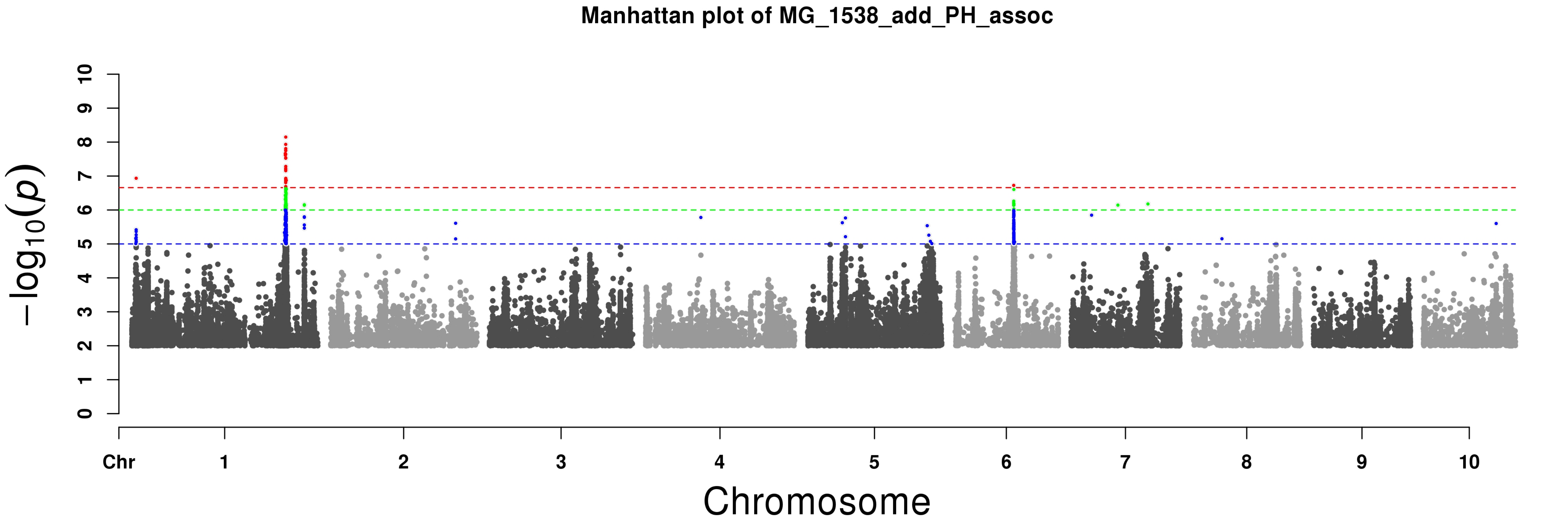

Manhattan plot of MG_1538_add_PH_assoc
−log10(p)
0
1
2
3
4
5
6
7
8
9
10
Chr
1
2
3
4
5
6
7
8
9
10
Chromosome

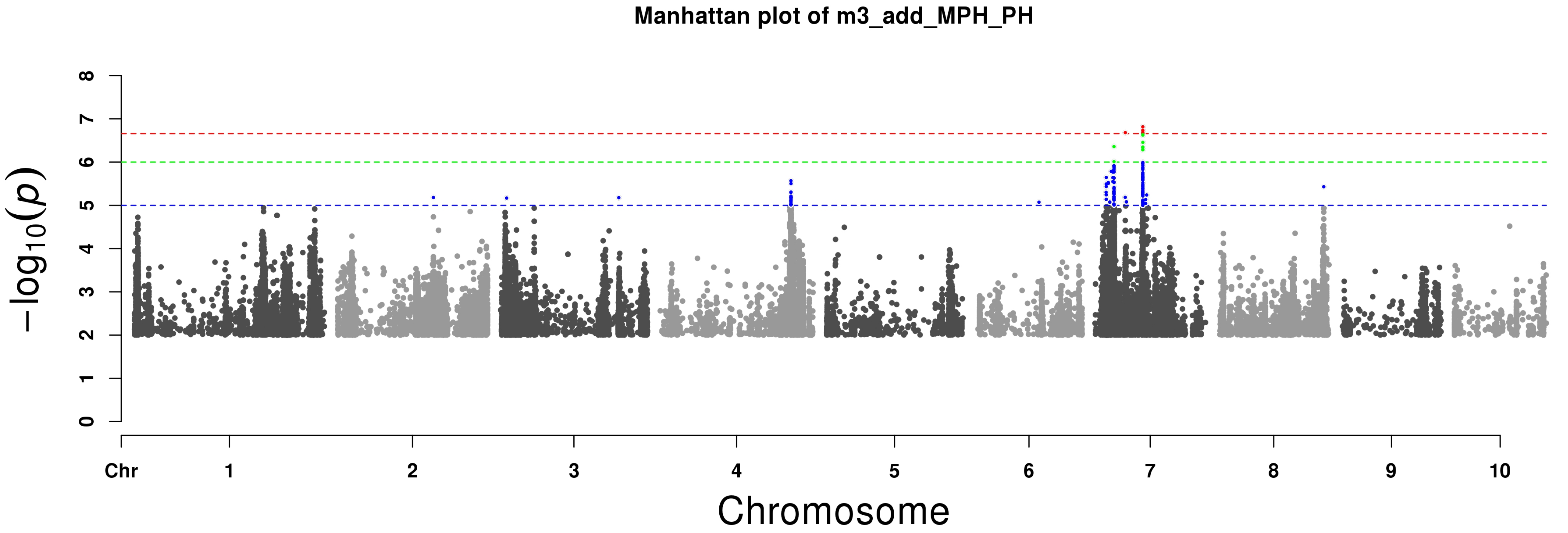
Manhattan plot of m3_add_MPH_PH
$-\log_{10}(p)$
0
1
2
3
4
5
6
7
8
Chr
1
2
3
4
5
6
7
8
9
10
Chromosome

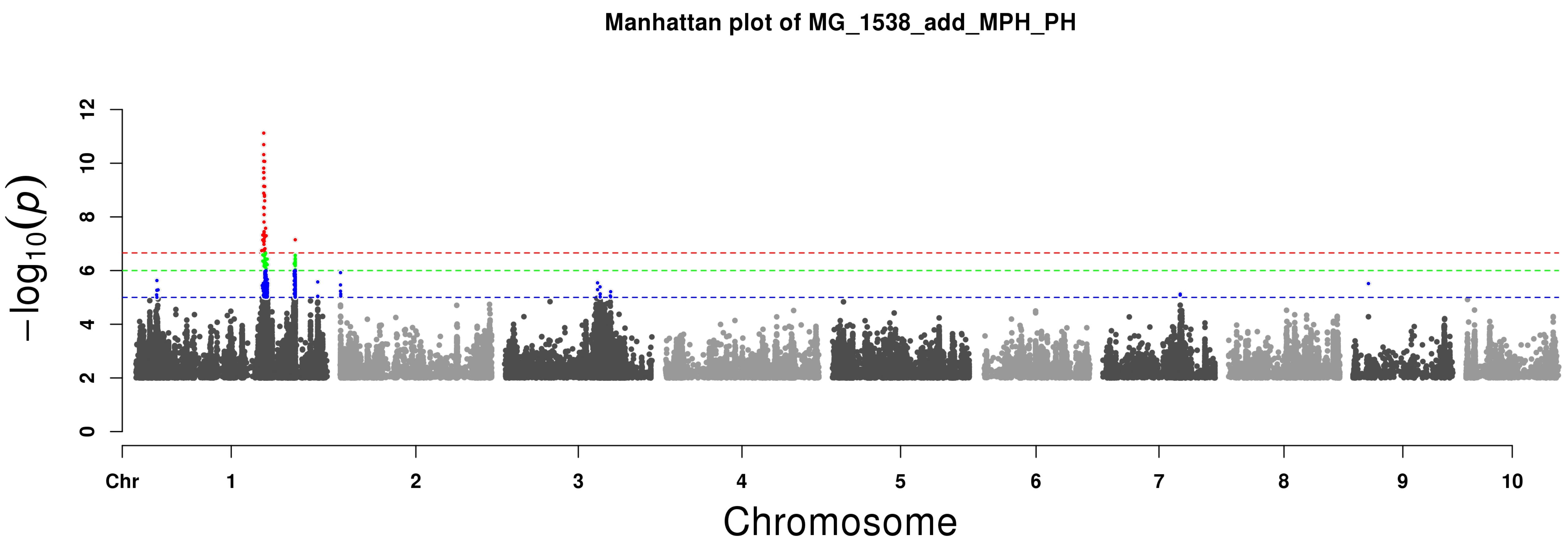

Manhattan plot of MG_1538_add_MPH_PH
$-\log_{10}(p)$
0
2
4
6
8
10
12
Chr
1
2
3
4
5
6
7
8
9
10
Chromosome

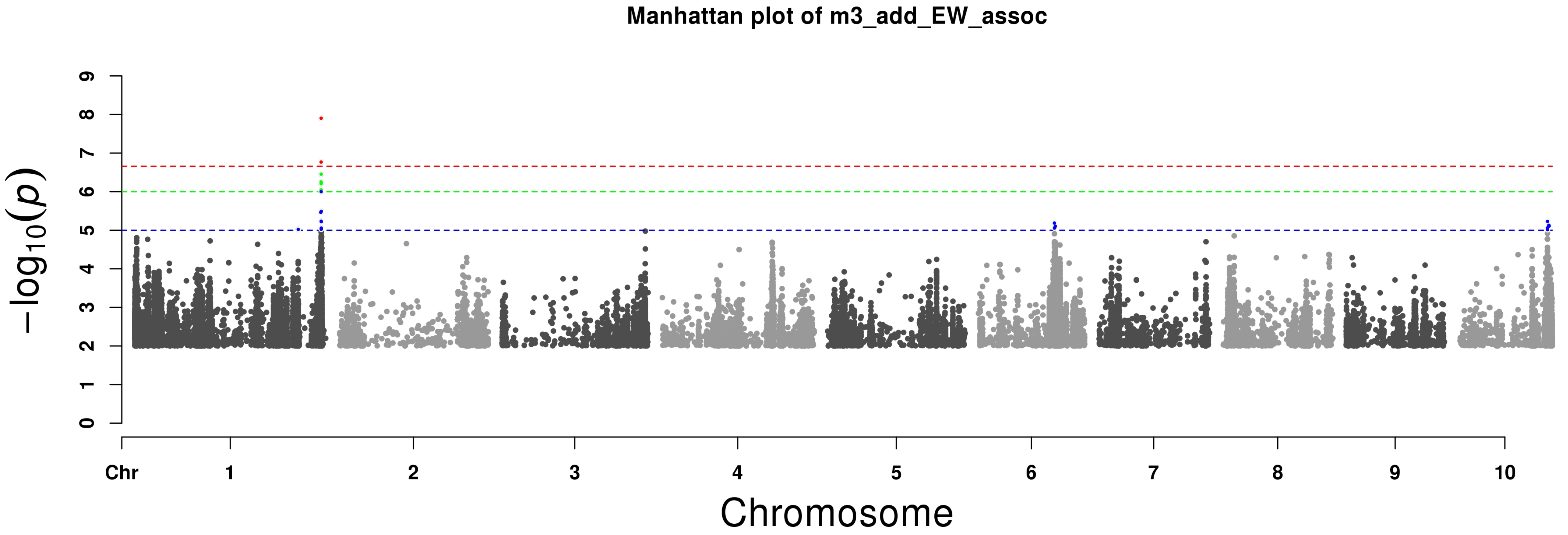

Manhattan plot of m3_add_EW_assoc
$-\log_{10}(p)$
0
1
2
3
4
5
6
7
8
9
Chr
1
2
3
4
5
6
7
8
9
10
Chromosome

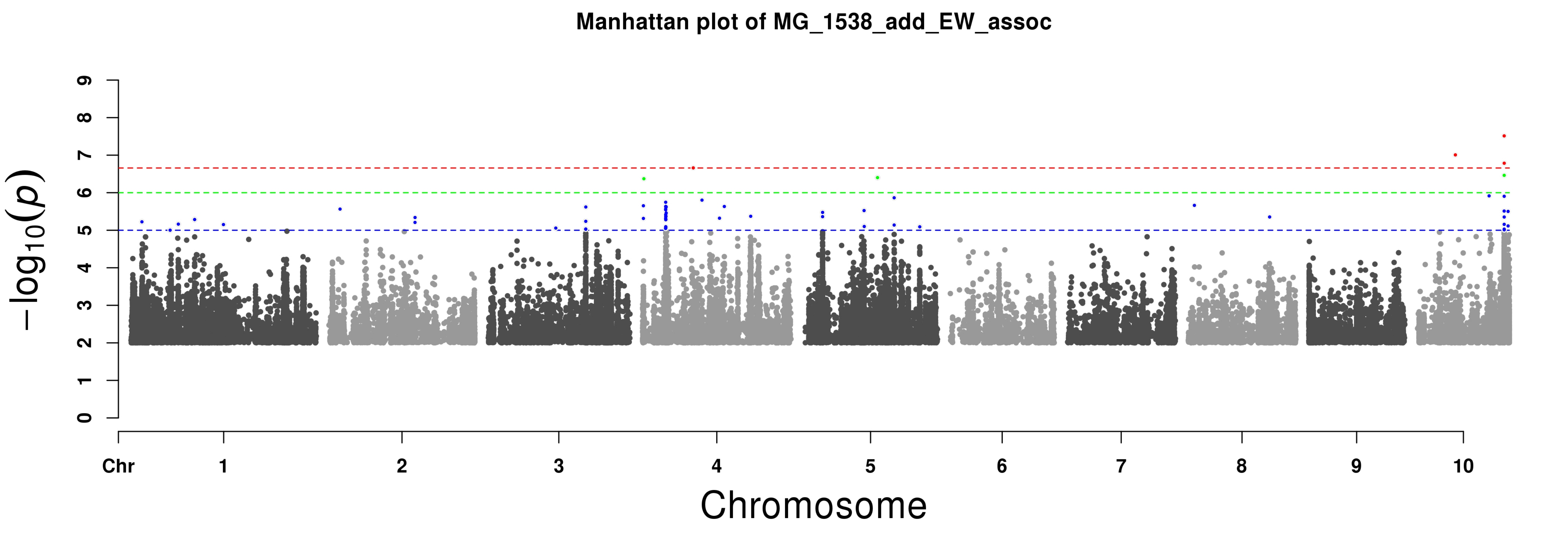

Manhattan plot of MG_1538_add_EW_assoc
$-\log_{10}(p)$
0
1
2
3
4
5
6
7
8
9
Chr
1
2
3
4
5
6
7
8
9
10
Chromosome

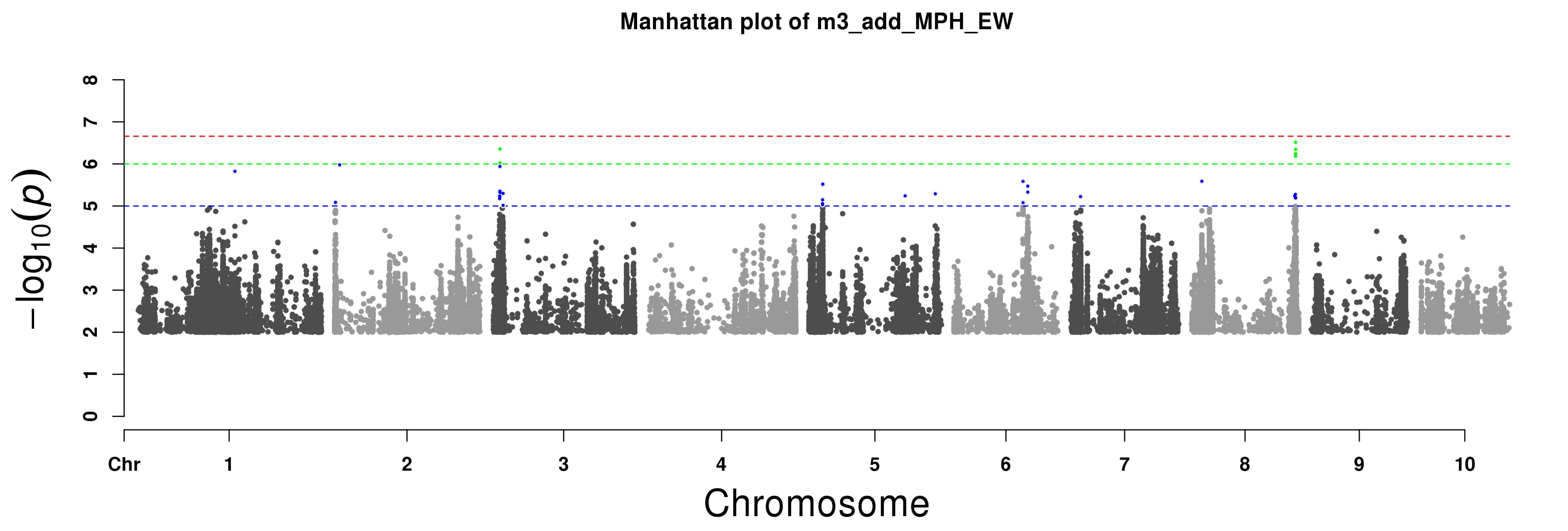

Manhattan plot of m3_add_MPH_EW
$-\log_{10}(p)$
0
1
2
3
4
5
6
7
8
Chr
1
2
3
4
5
6
7
8
9
10
Chromosome

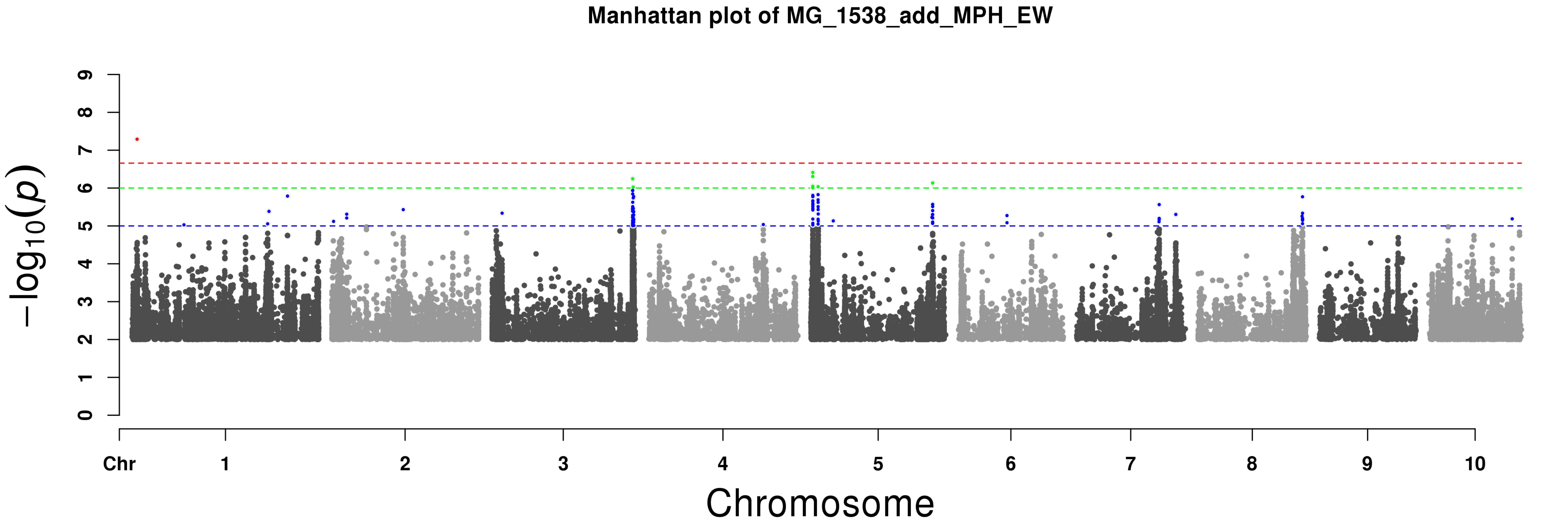

Manhattan plot of MG_1538_add_MPH_EW
$-\log_{10}(p)$
0
1
2
3
4
5
6
7
8
9
Chr
1
2
3
4
5
6
7
8
9
10
Chromosome

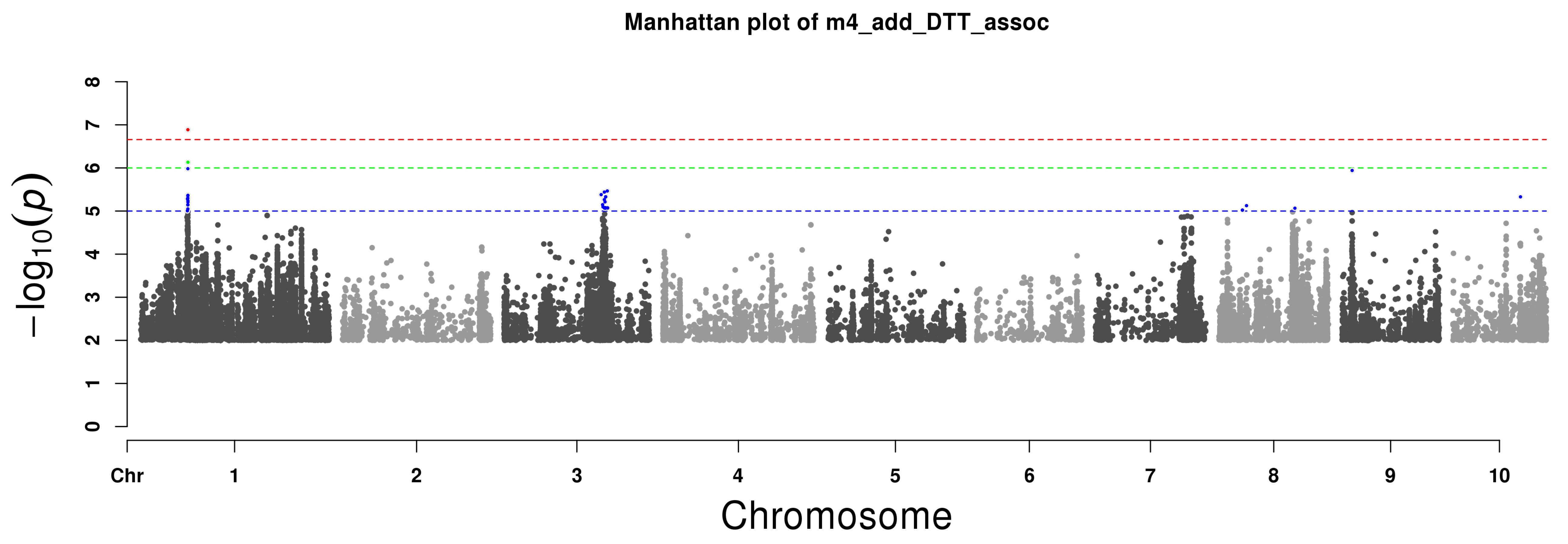

Manhattan plot of m4_add_DTT_assoc
$-\log_{10}(p)$
0
1
2
3
4
5
6
7
8
Chr
1
2
3
4
5
6
7
8
9
10
Chromosome

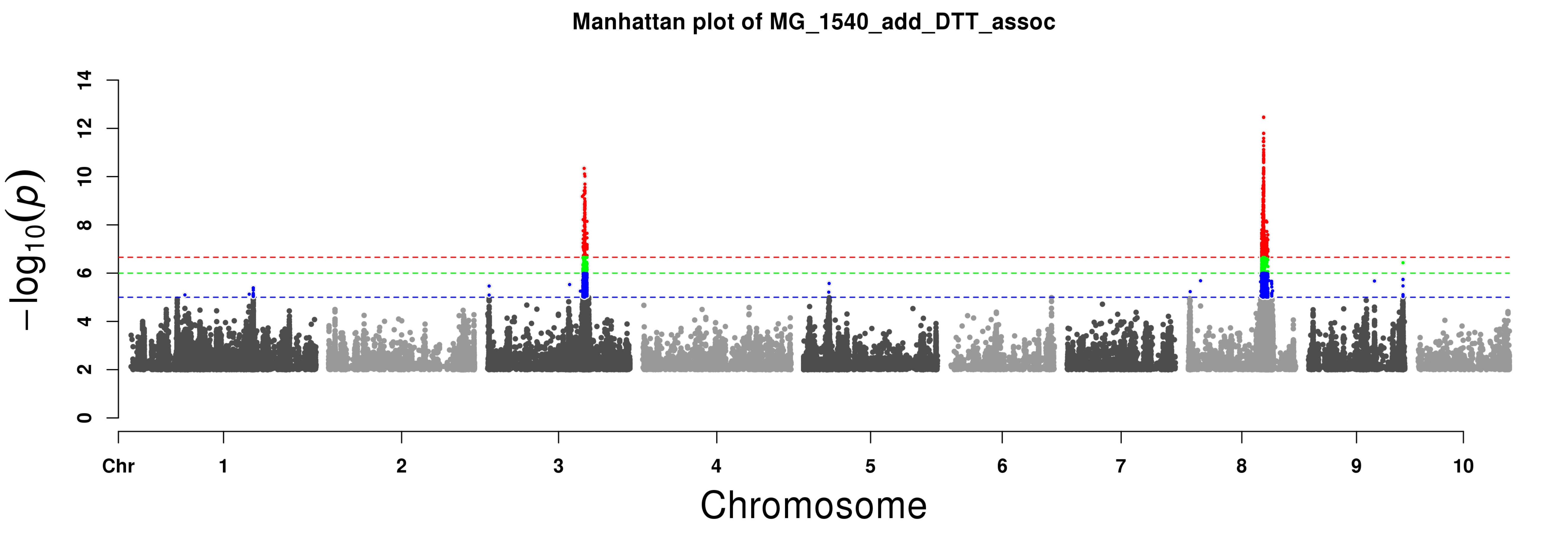
Manhattan plot of MG_1540_add_DTT_assoc
0
2
4
6
8
10
12
14
$-\log_{10}(p)$
Chr
1
2
3
4
5
6
7
8
9
10
Chromosome

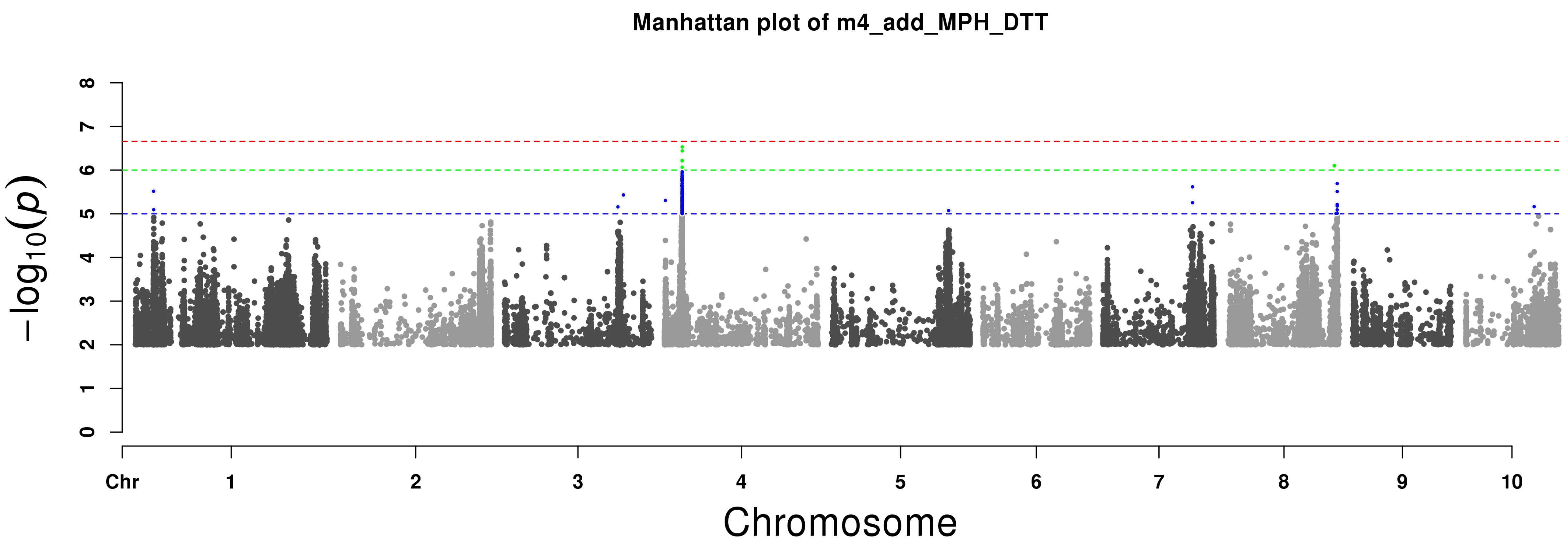

Manhattan plot of m4_add_MPH_DTT
$-\log_{10}(p)$
0
1
2
3
4
5
6
7
8
Chr
1
2
3
4
5
6
7
8
9
10
Chromosome

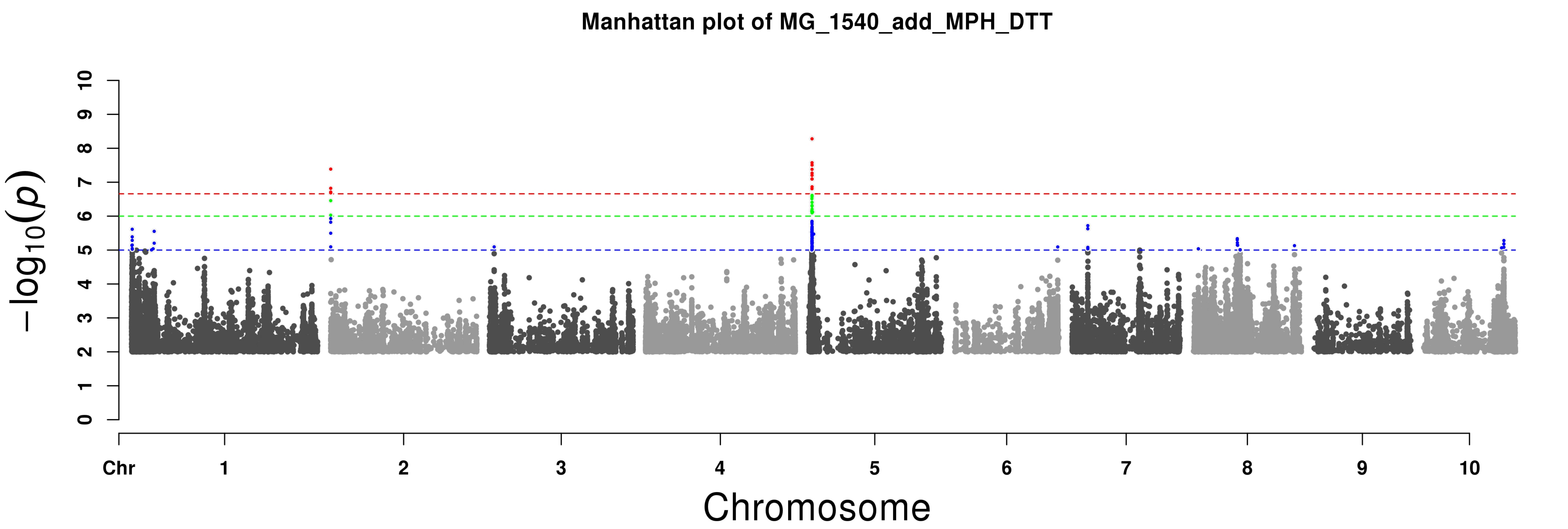
Manhattan plot of MG_1540_add_MPH_DTT
$-\log_{10}(p)$
0
1
2
3
4
5
6
7
8
9
10
Chr
1
2
3
4
5
6
7
8
9
10
Chromosome

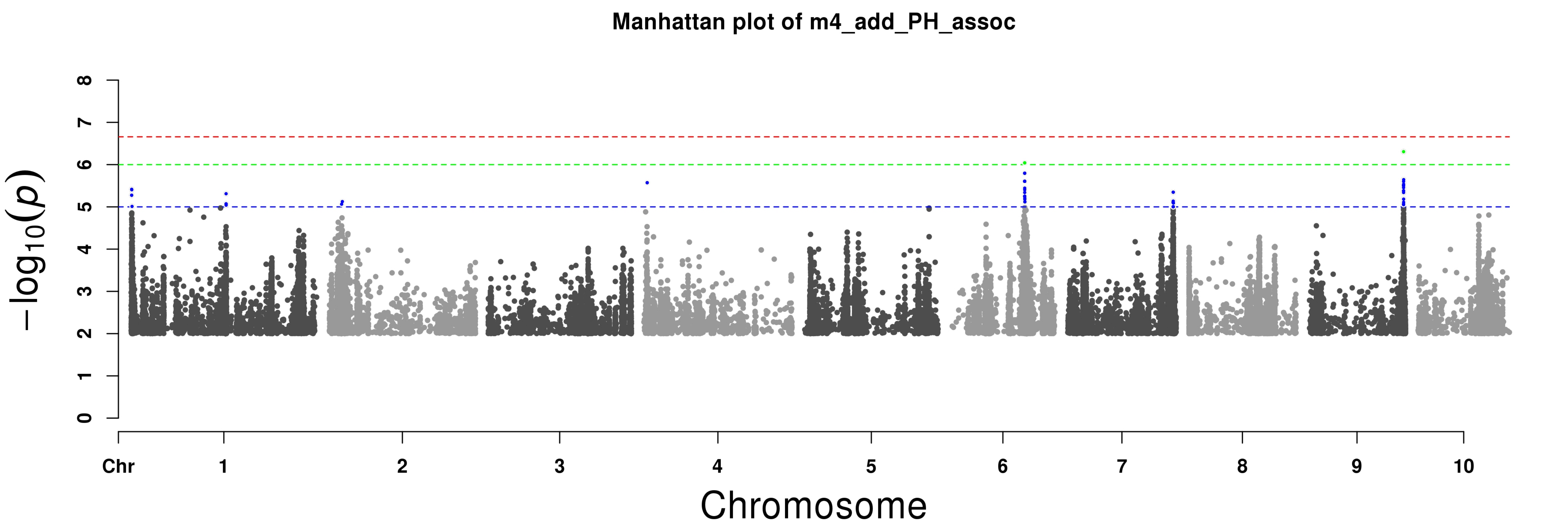

Manhattan plot of m4_add_PH_assoc
$-\log_{10}(p)$
0
1
2
3
4
5
6
7
8
Chr
1
2
3
4
5
6
7
8
9
10
Chromosome

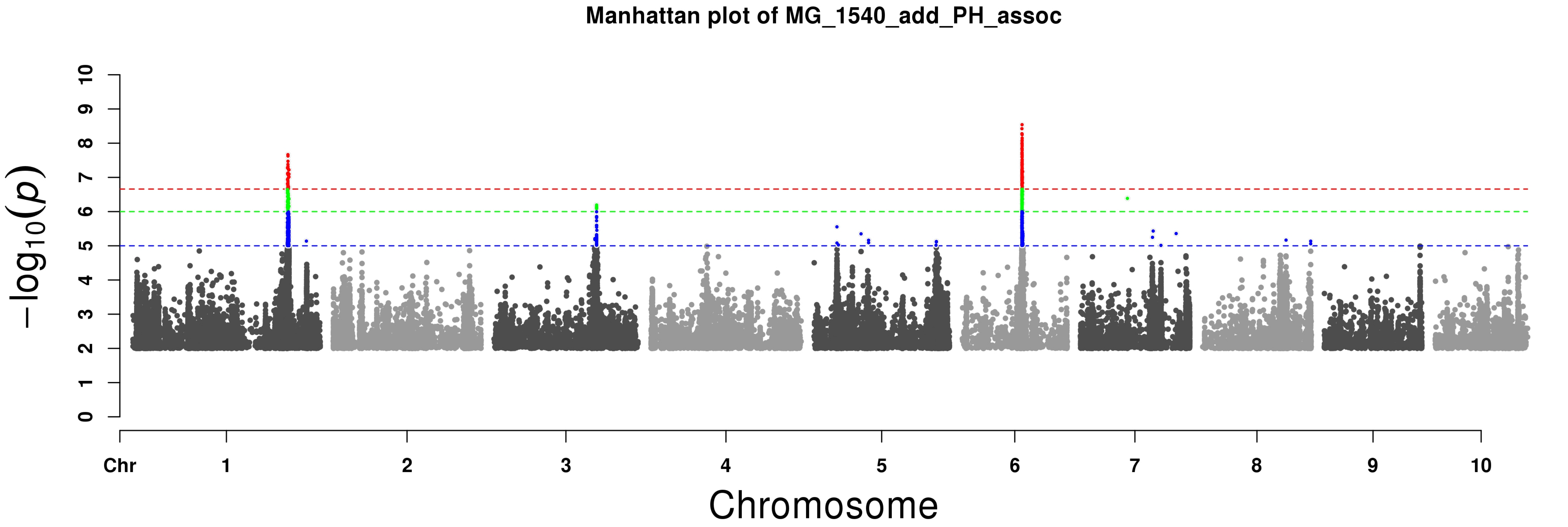
Manhattan plot of MG_1540_add_PH_assoc
$-\log_{10}(p)$
0
1
2
3
4
5
6
7
8
9
10
Chr
1
2
3
4
5
6
7
8
9
10
Chromosome

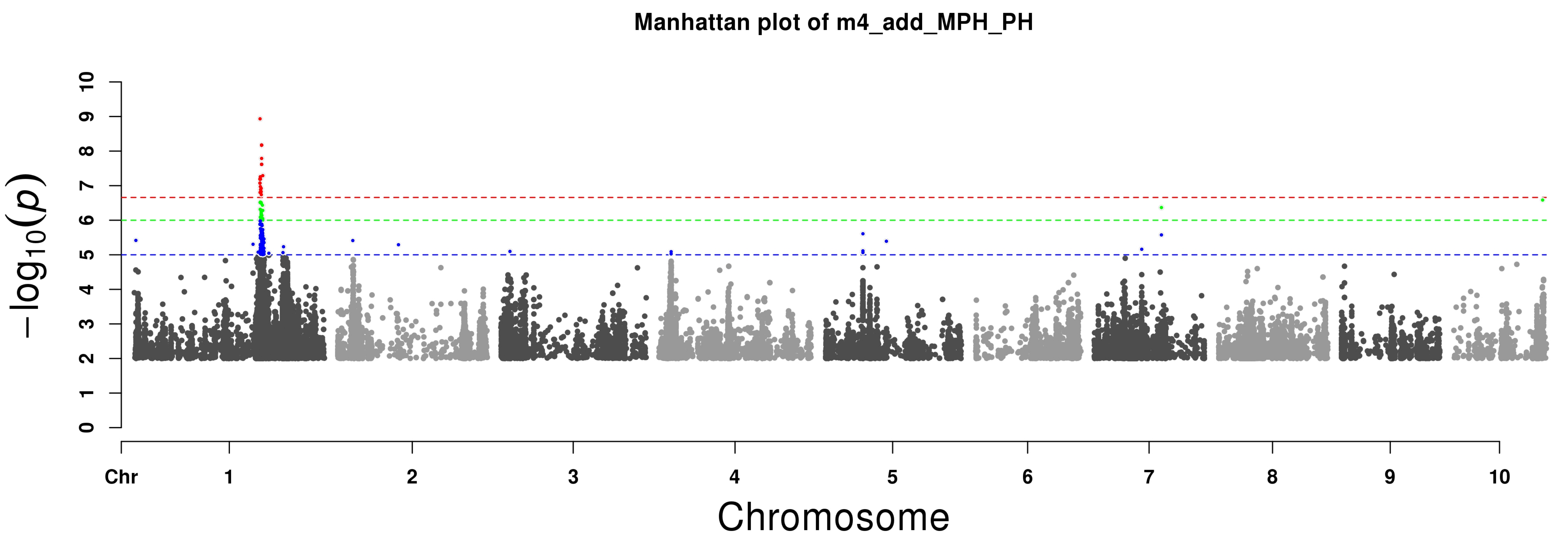
Manhattan plot of m4_add_MPH_PH
$-\log_{10}(p)$
0
1
2
3
4
5
6
7
8
9
10
Chr
1
2
3
4
5
6
7
8
9
10
Chromosome

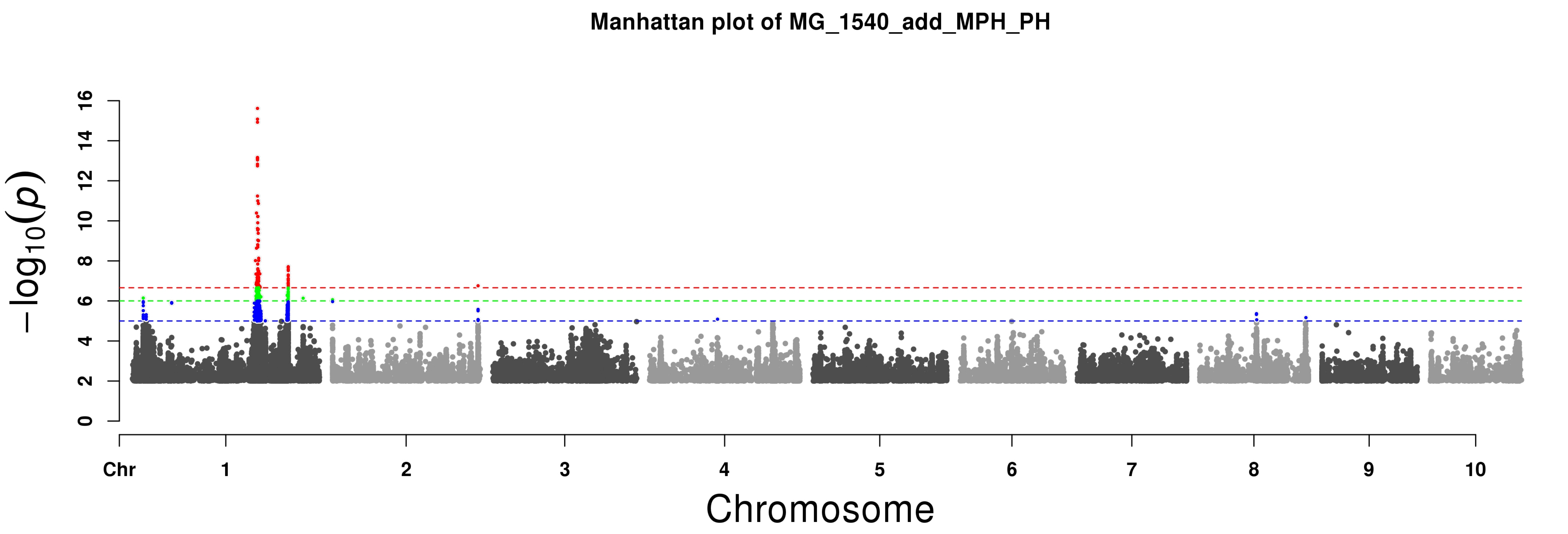

Manhattan plot of MG_1540_add_MPH_PH
$-\log_{10}(p)$
0
2
4
6
8
10
12
14
16
Chr
1
2
3
4
5
6
7
8
9
10
Chromosome

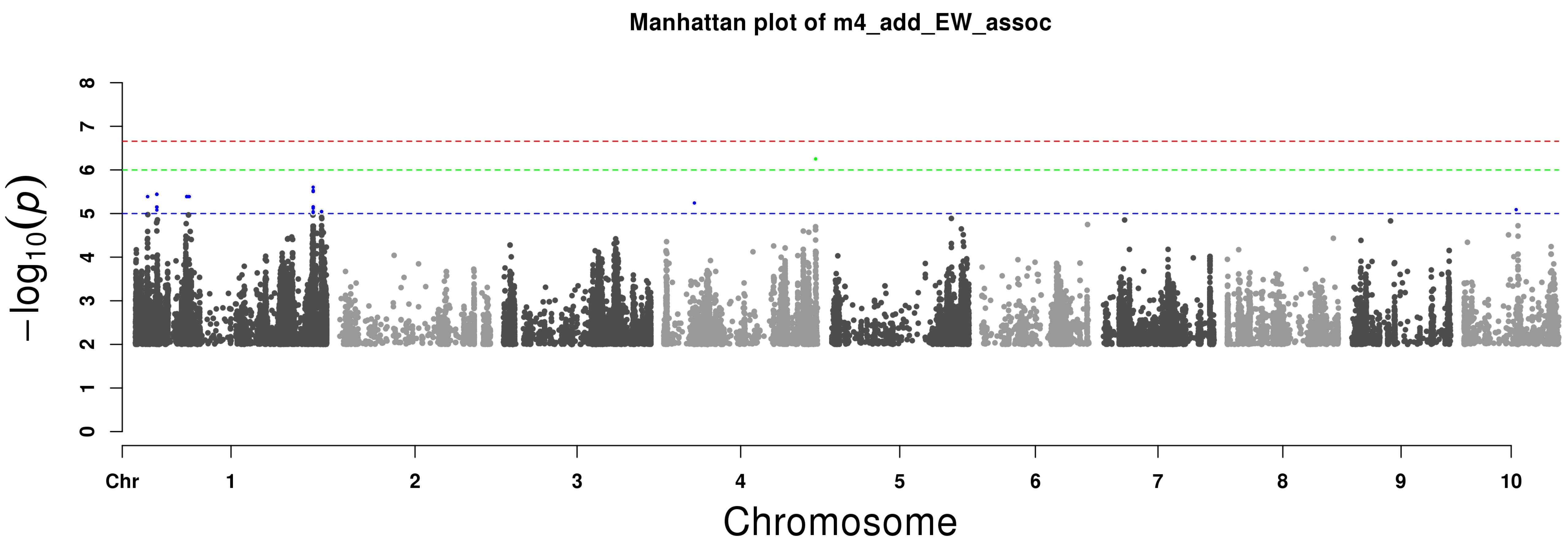
Manhattan plot of m4_add_EW_assoc
$-\log_{10}(p)$
0
1
2
3
4
5
6
7
8
Chr
1
2
3
4
5
6
7
8
9
10
Chromosome

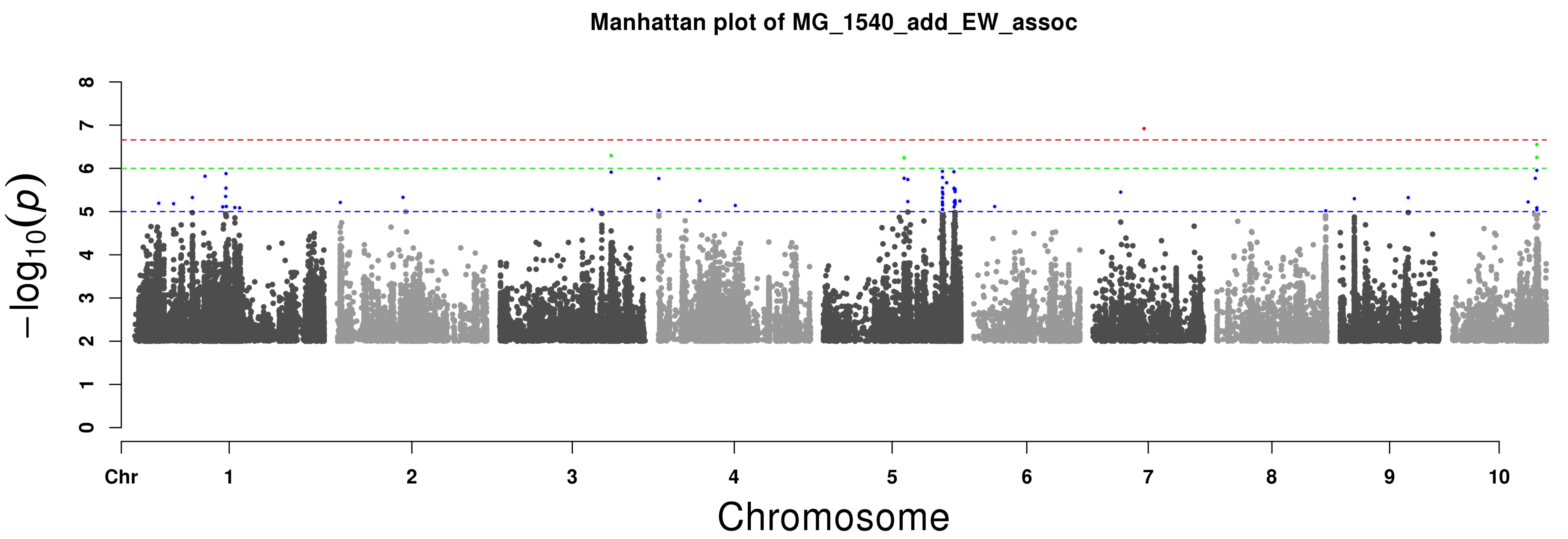

Manhattan plot of MG_1540_add_EW_assoc
$-\log_{10}(p)$
0
1
2
3
4
5
6
7
8
Chr
1
2
3
4
5
6
7
8
9
10
Chromosome

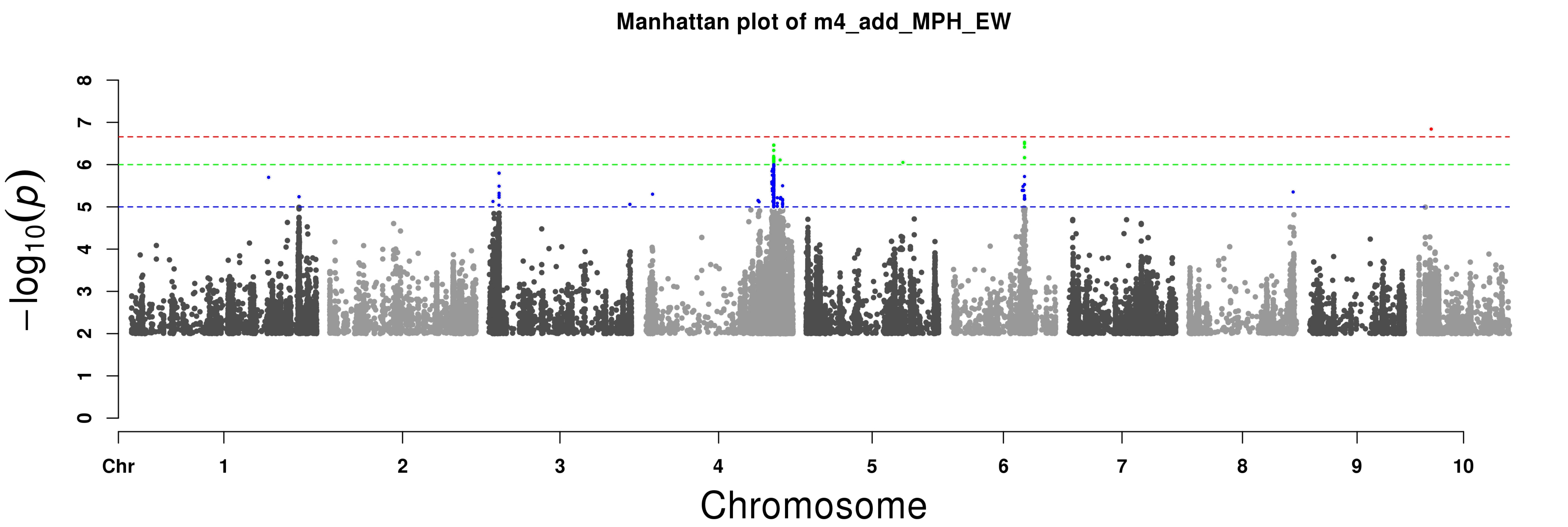

Manhattan plot of m4_add_MPH_EW
$-\log_{10}(p)$
0
1
2
3
4
5
6
7
8
Chr
1
2
3
4
5
6
7
8
9
10
Chromosome

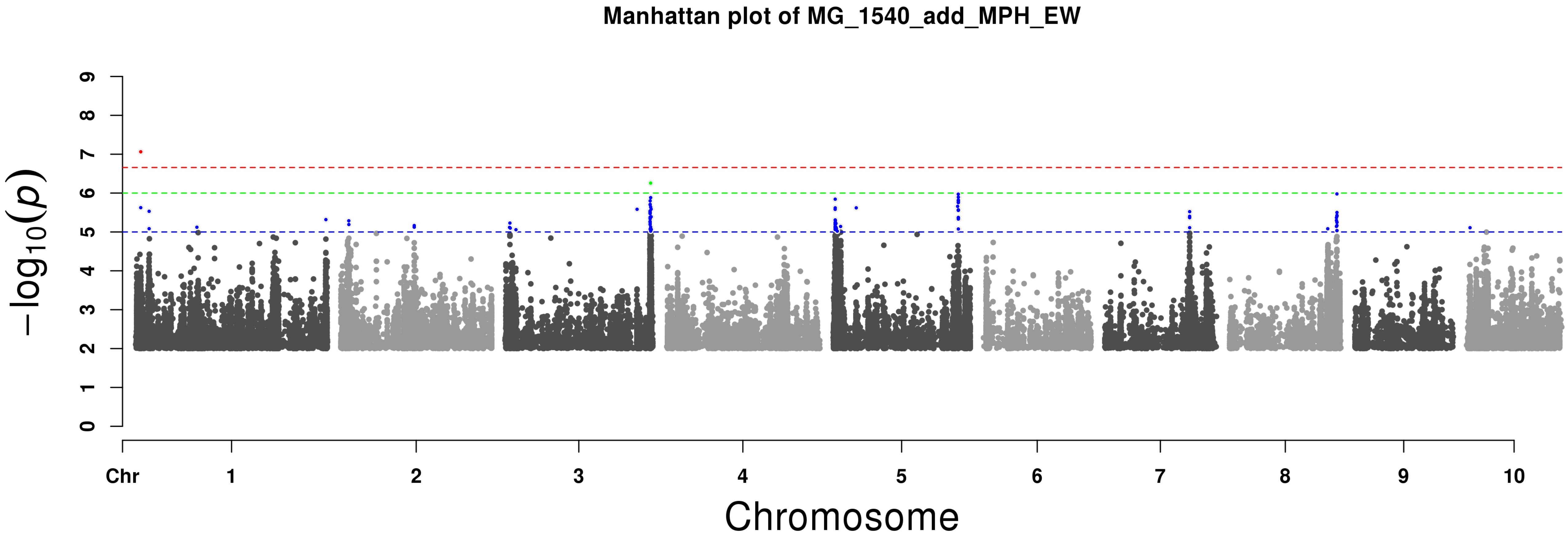

Manhattan plot of MG_1540_add_MPH_EW
$-\log_{10}(p)$
0
1
2
3
4
5
6
7
8
9
Chr
1
2
3
4
5
6
7
8
9
10
Chromosome

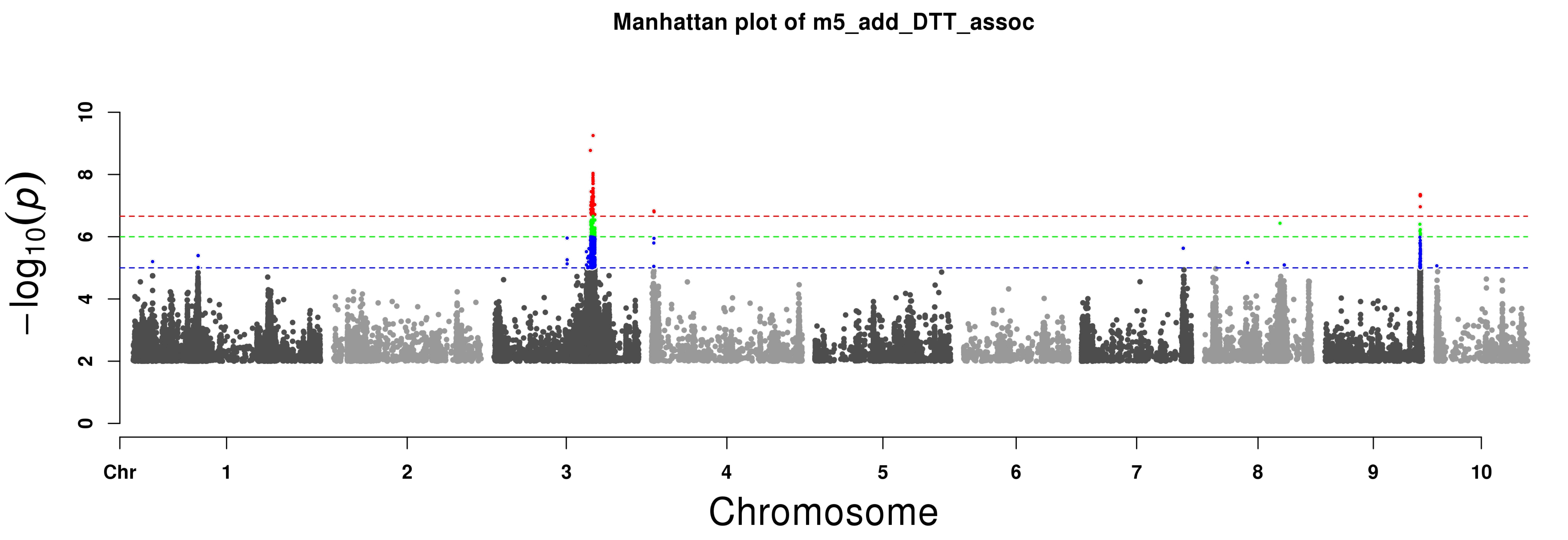

Manhattan plot of m5_add_DTT_assoc
$-\log_{10}(p)$
0
2
4
6
8
10
Chr
1
2
3
4
5
6
7
8
9
10
Chromosome

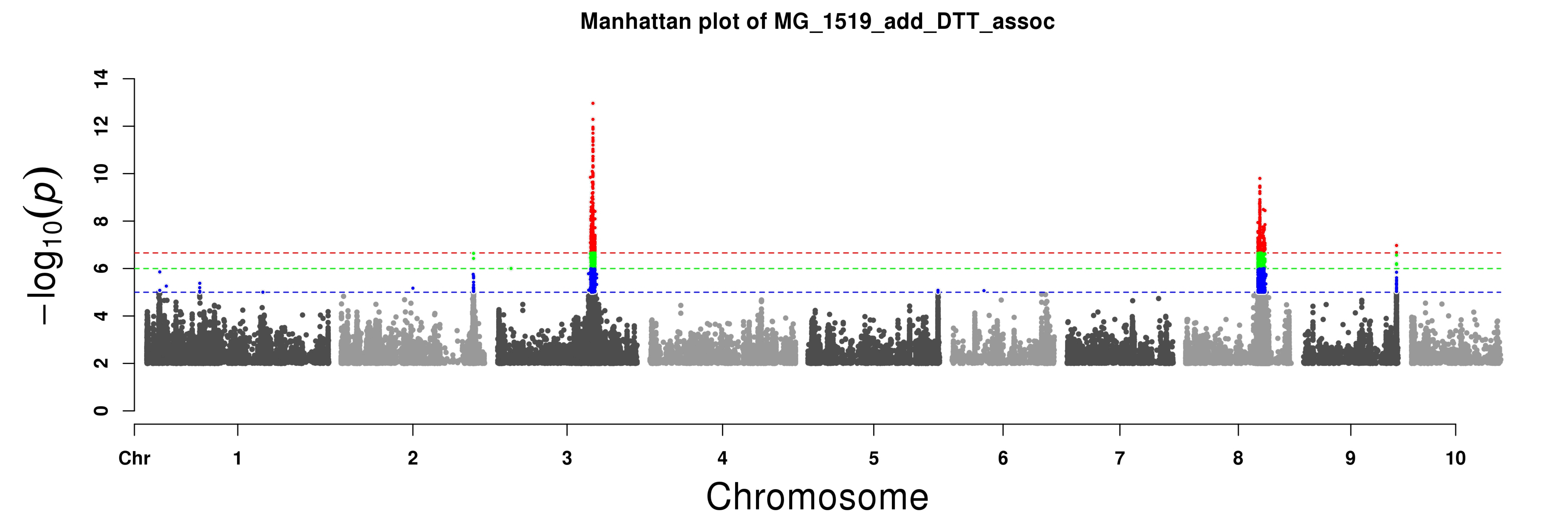
Manhattan plot of MG_1519_add_DTT_assoc
$-\log_{10}(p)$
0
2
4
6
8
10
12
14
Chr
1
2
3
4
5
6
7
8
9
10
Chromosome

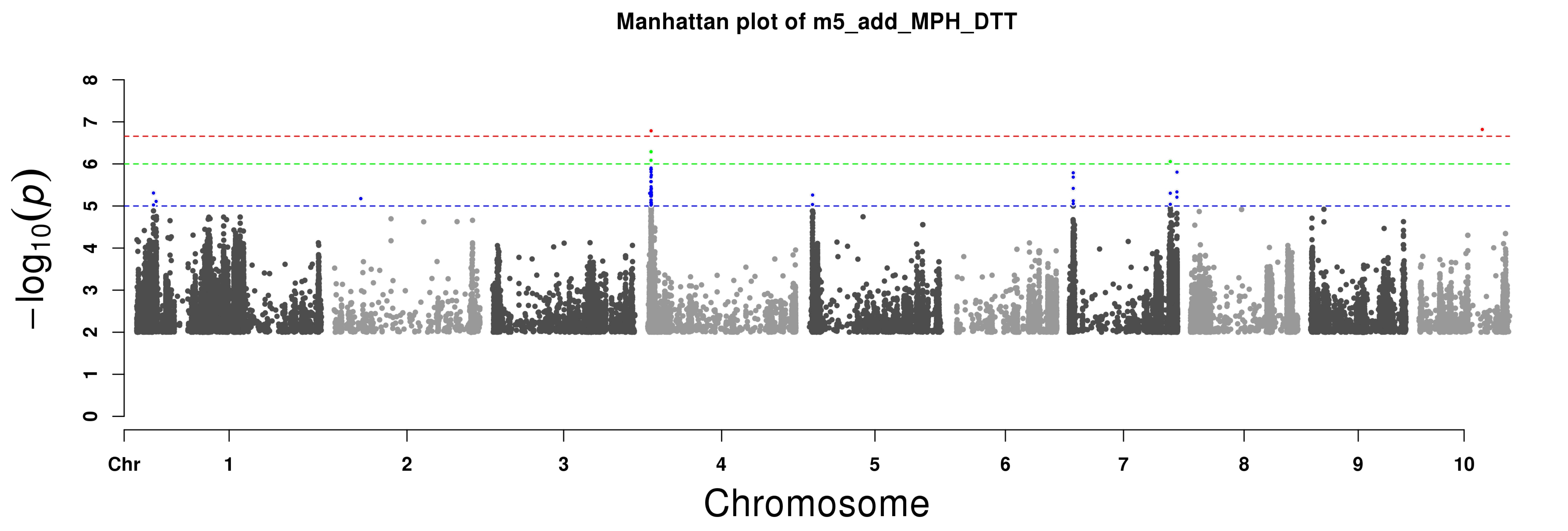

Manhattan plot of m5_add_MPH_DTT
−log10(p)
0
1
2
3
4
5
6
7
8
Chr
1
2
3
4
5
6
7
8
9
10
Chromosome

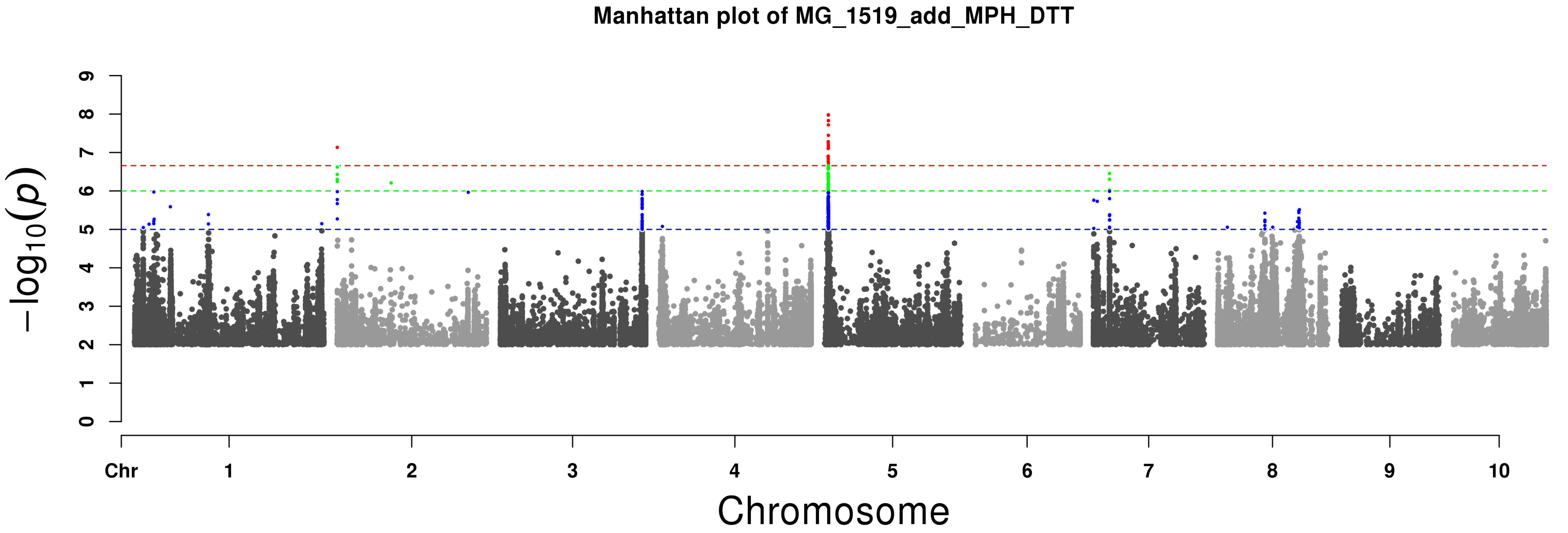
Manhattan plot of MG_1519_add_MPH_DTT
$-\log_{10}(p)$
0
1
2
3
4
5
6
7
8
9
Chr
1
2
3
4
5
6
7
8
9
10
Chromosome

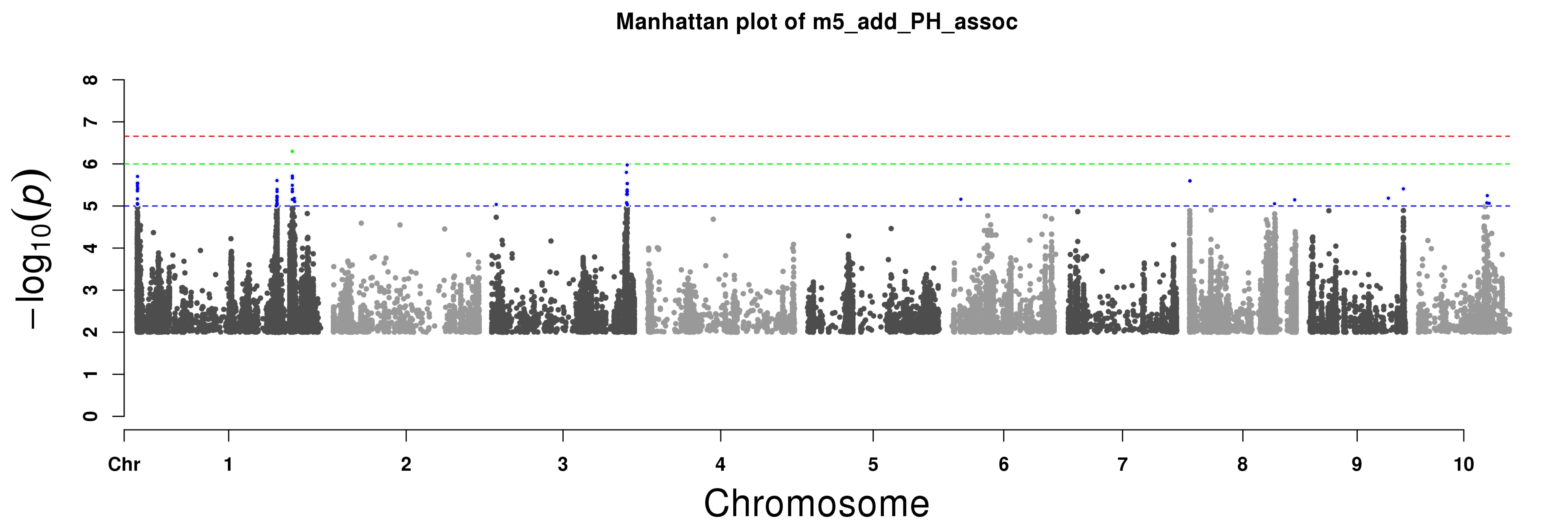

Manhattan plot of m5_add_PH_assoc
$-\log_{10}(p)$
0
1
2
3
4
5
6
7
8
Chr
1
2
3
4
5
6
7
8
9
10
Chromosome

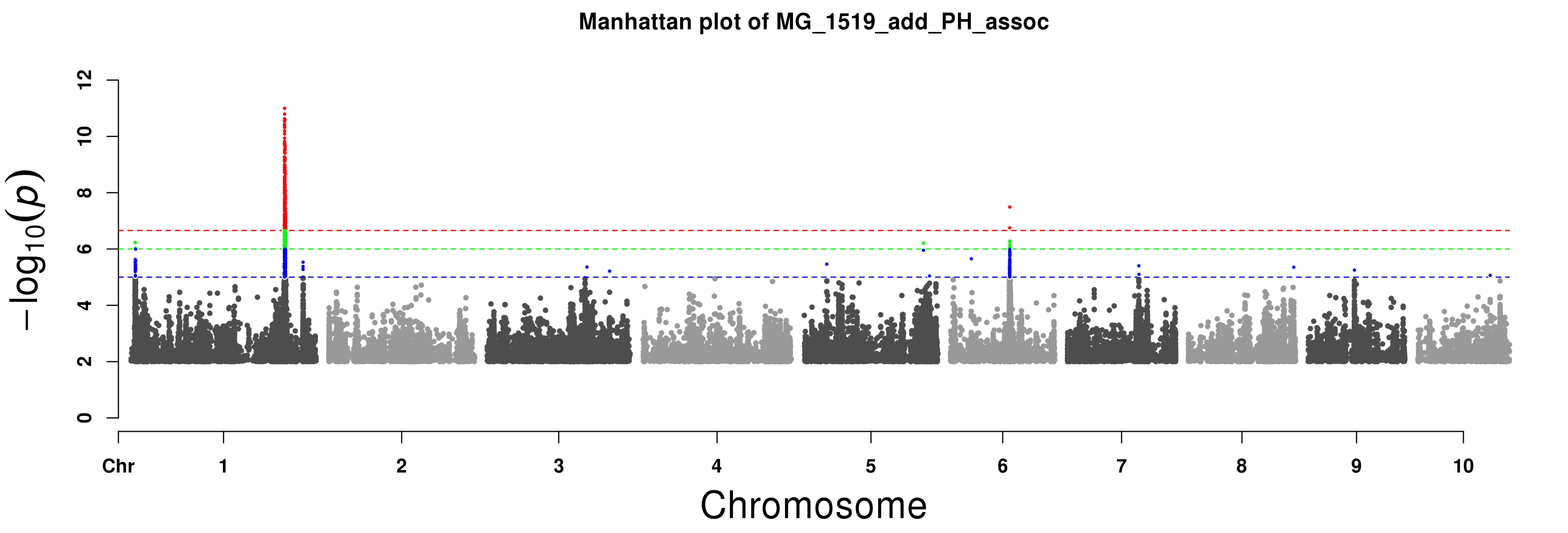
Manhattan plot of MG_1519_add_PH_assoc
−log10(p)
0
2
4
6
8
10
12
Chr
1
2
3
4
5
6
7
8
9
10
Chromosome

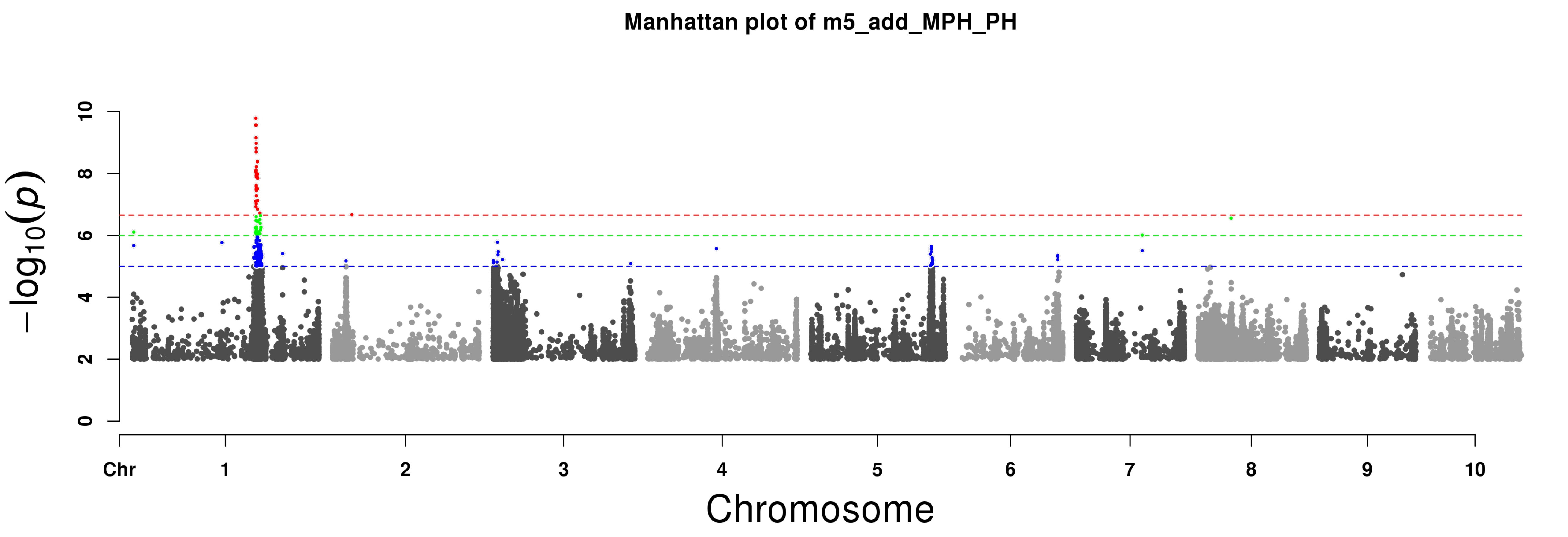

Manhattan plot of m5_add_MPH_PH
$-\log_{10}(p)$
0
2
4
6
8
10
Chr
1
2
3
4
5
6
7
8
9
10
Chromosome

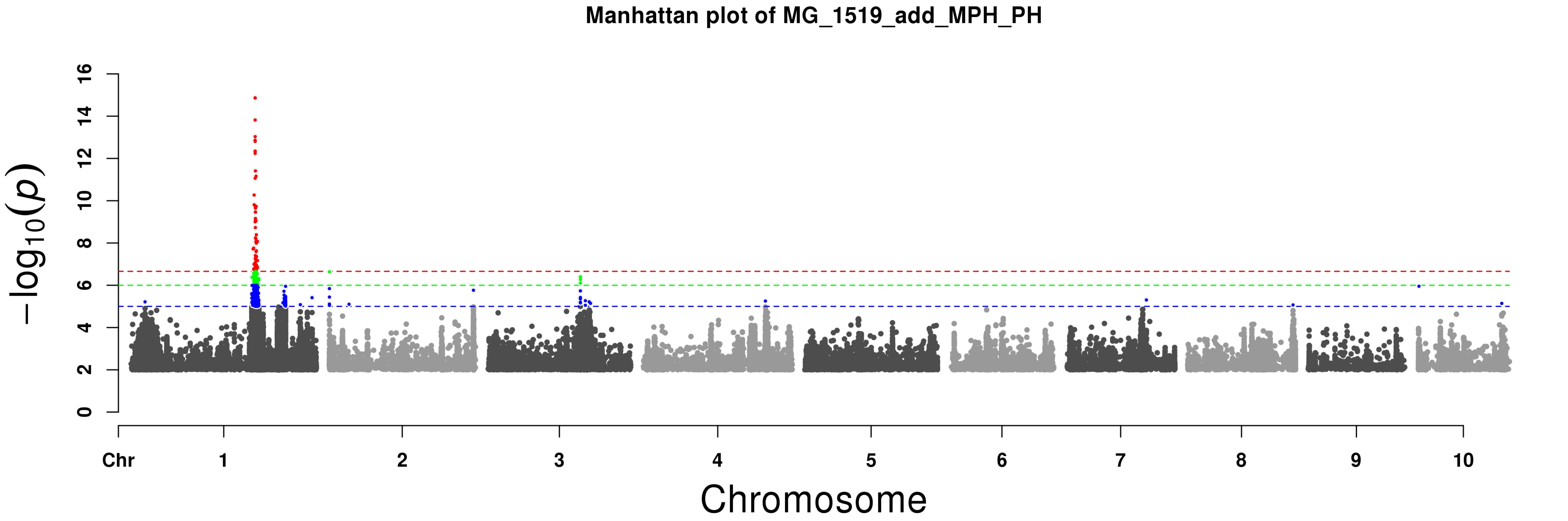

Manhattan plot of MG_1519_add_MPH_PH
$-\log_{10}(p)$
0
2
4
6
8
10
12
14
16
Chr
1
2
3
4
5
6
7
8
9
10
Chromosome

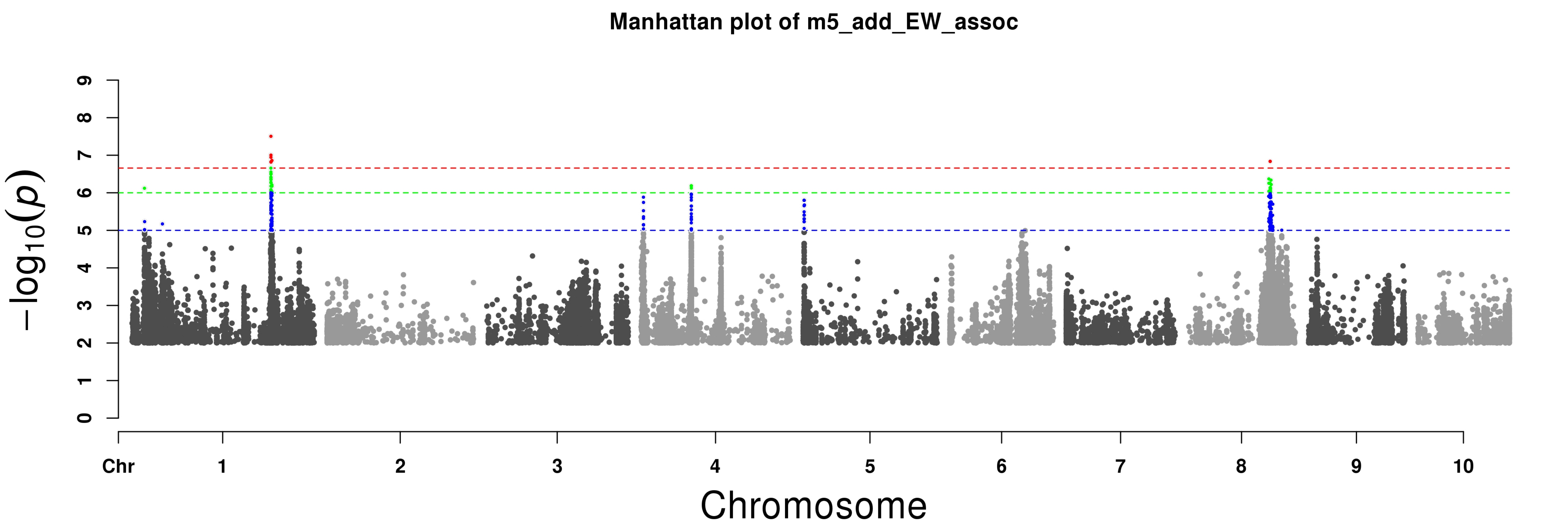

Manhattan plot of m5_add_EW_assoc
$-\log_{10}(p)$
0
1
2
3
4
5
6
7
8
9
Chr
1
2
3
4
5
6
7
8
9
10
Chromosome

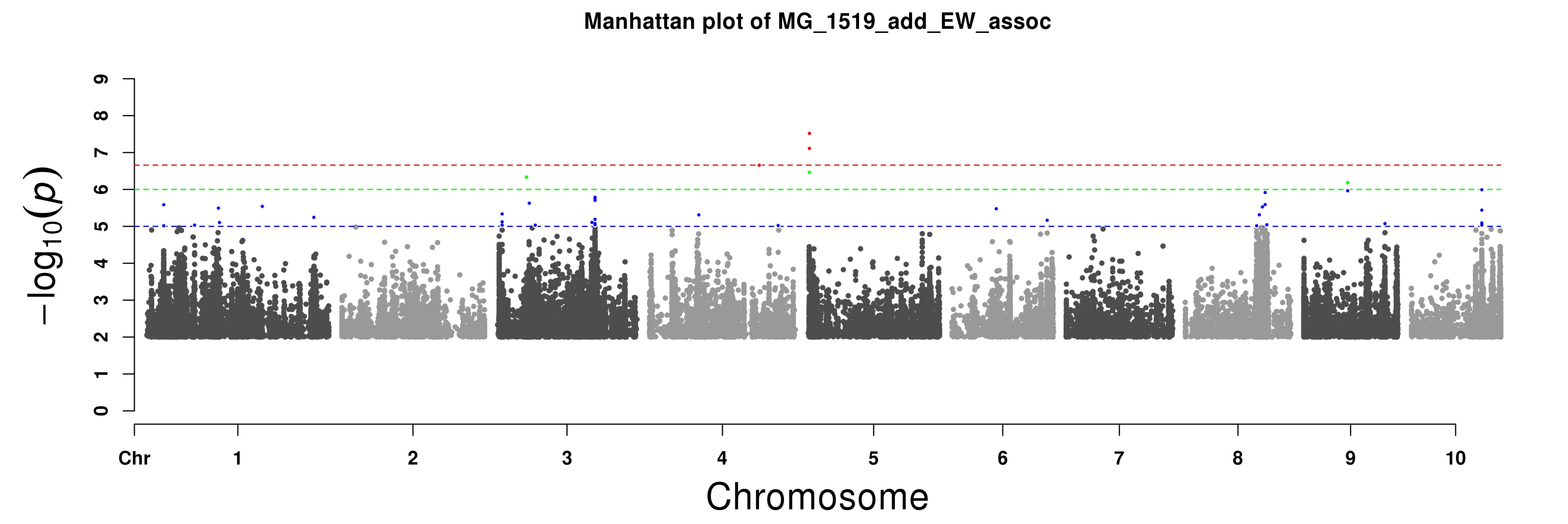
Manhattan plot of MG_1519_add_EW_assoc
$-\log_{10}(p)$
0
1
2
3
4
5
6
7
8
9
Chr
1
2
3
4
5
6
7
8
9
10
Chromosome

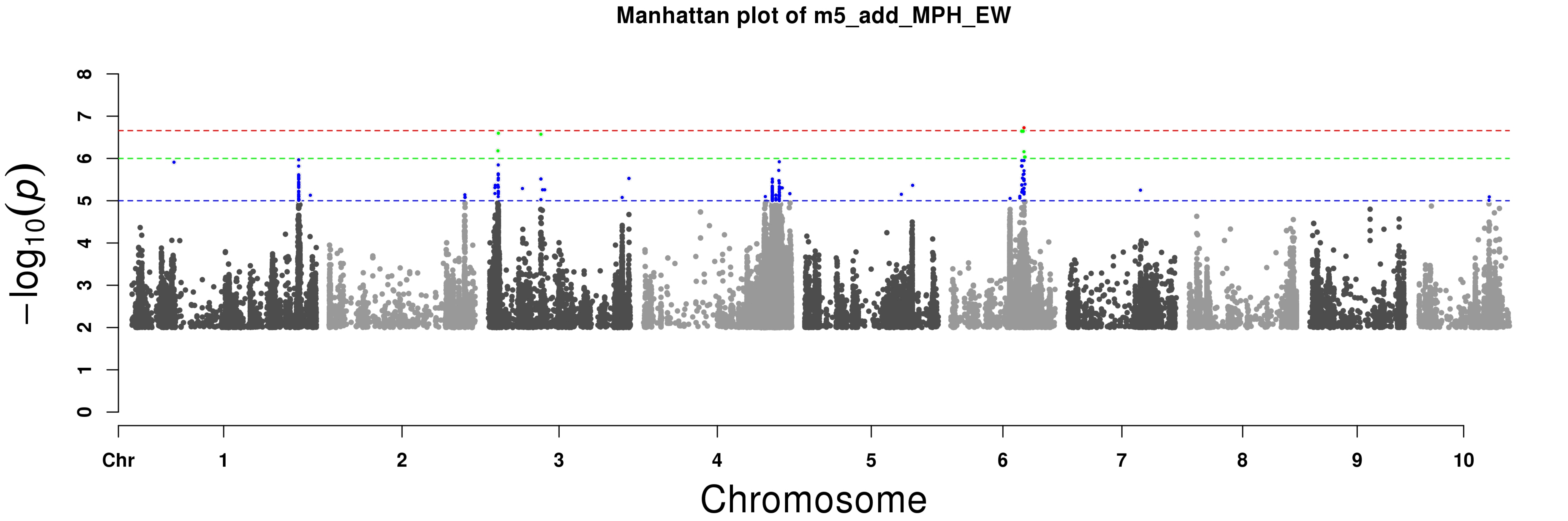

Manhattan plot of m5_add_MPH_EW
$-\log_{10}(p)$
0
1
2
3
4
5
6
7
8
Chr
1
2
3
4
5
6
7
8
9
10
Chromosome

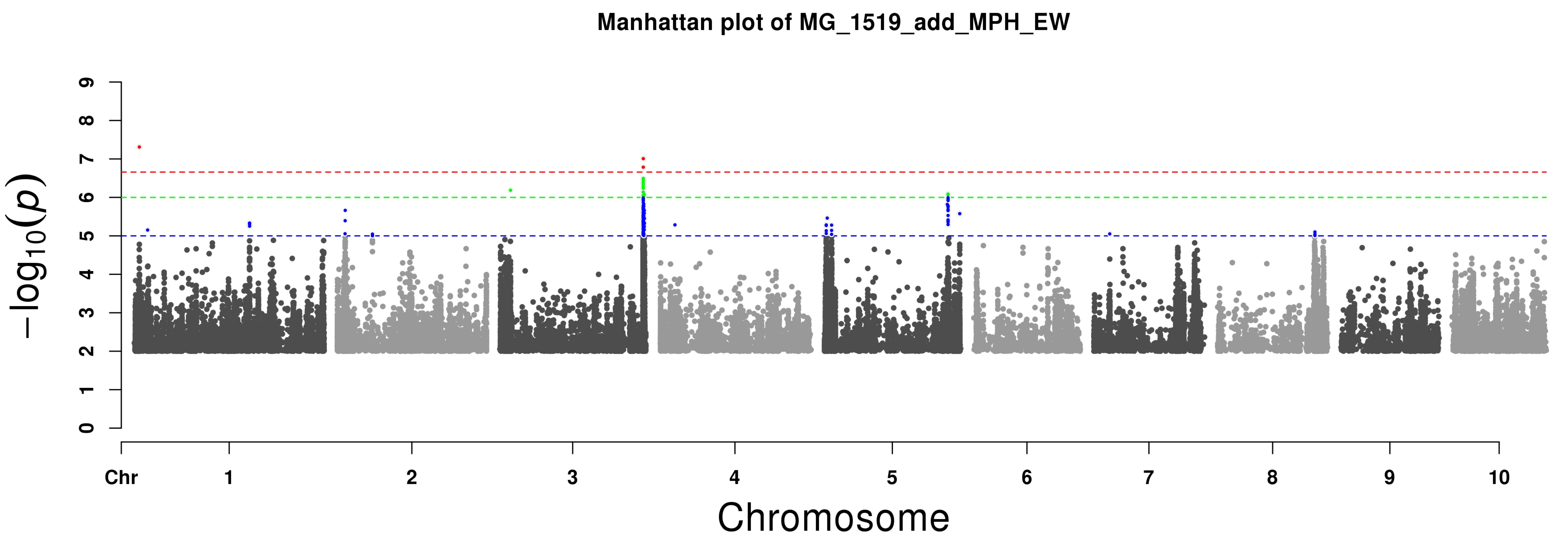

Manhattan plot of MG_1519_add_MPH_EW
$-\log_{10}(p)$
0
1
2
3
4
5
6
7
8
9
Chr
1
2
3
4
5
6
7
8
9
10
Chromosome

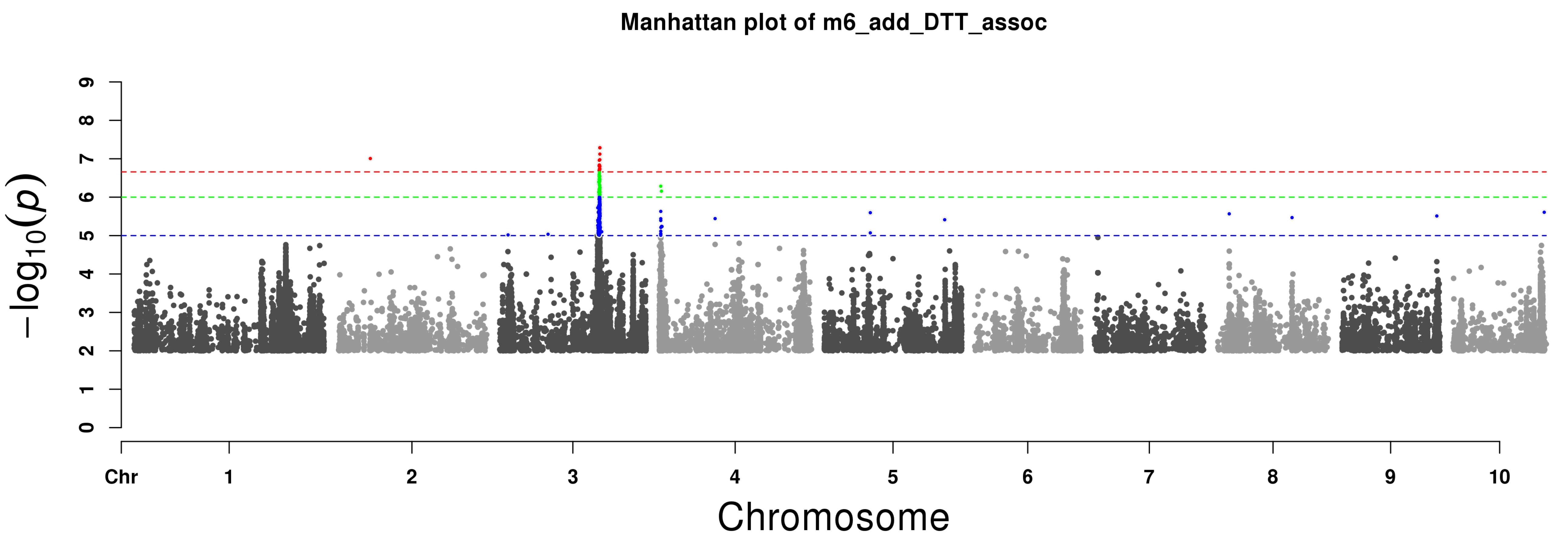

Manhattan plot of m6_add_DTT_assoc
−log10(p)
0
1
2
3
4
5
6
7
8
9
Chr
1
2
3
4
5
6
7
8
9
10
Chromosome

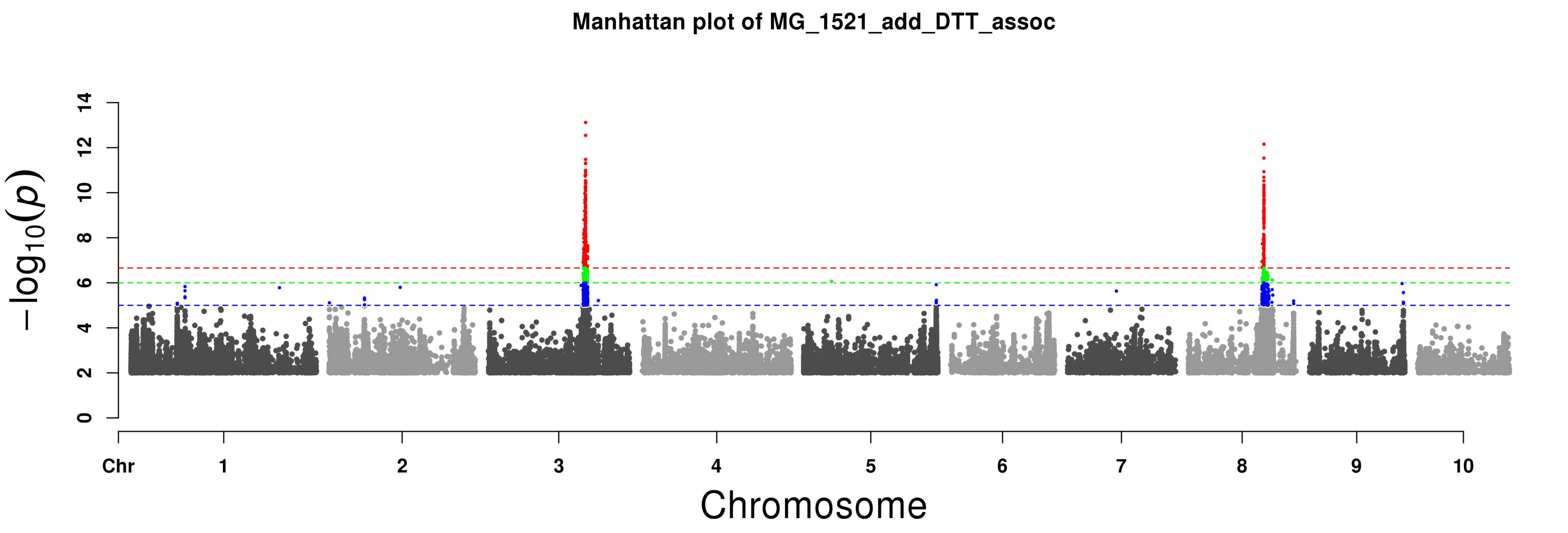

Manhattan plot of MG_1521_add_DTT_assoc
$-\log_{10}(p)$
0
2
4
6
8
10
12
14
Chr
1
2
3
4
5
6
7
8
9
10
Chromosome

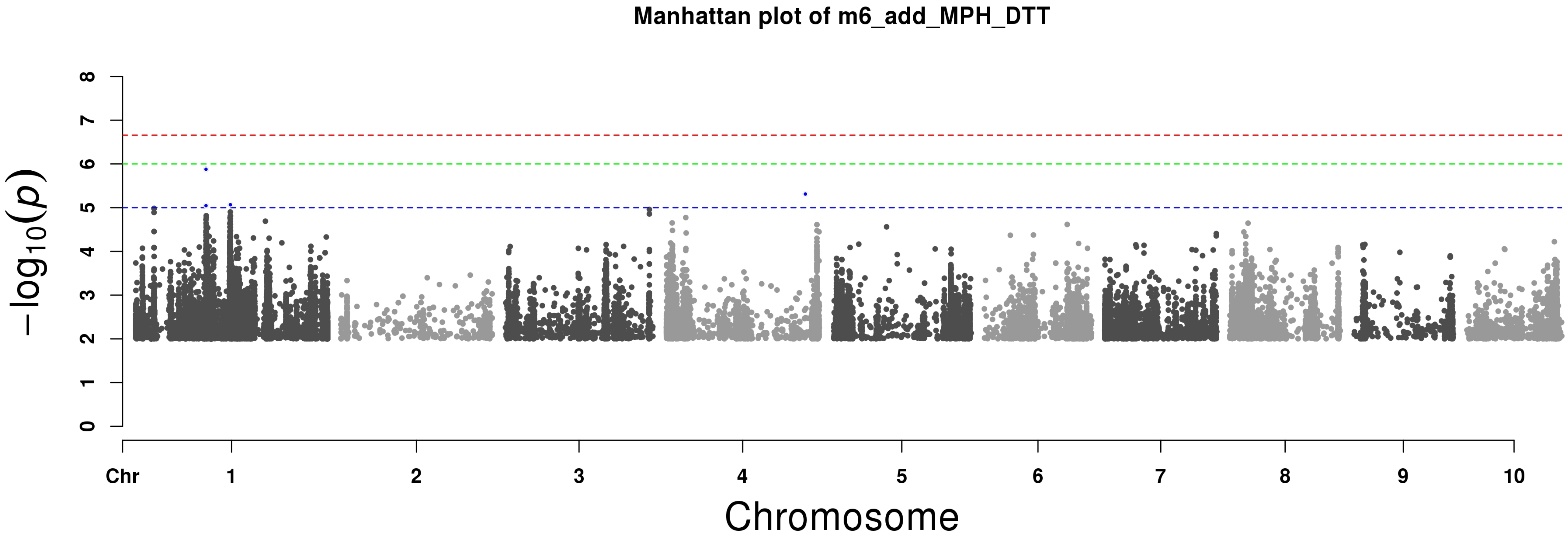
Manhattan plot of m6_add_MPH_DTT
$-\log_{10}(p)$
0
1
2
3
4
5
6
7
8
Chr
1
2
3
4
5
6
7
8
9
10
Chromosome

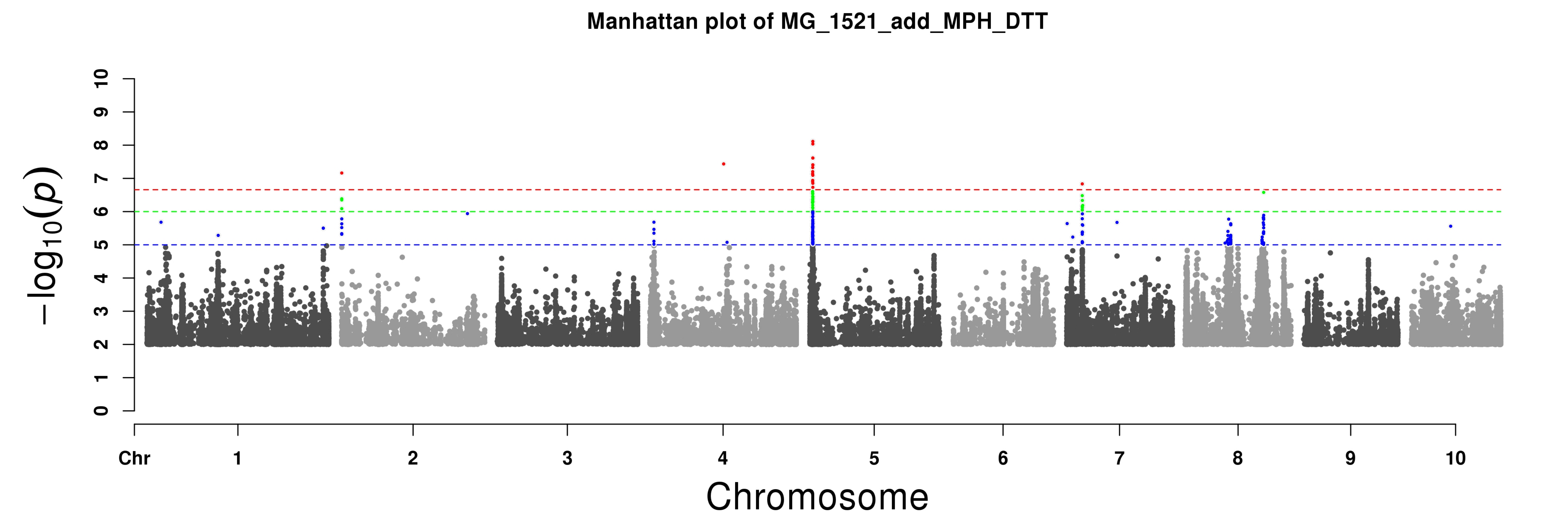
Manhattan plot of MG_1521_add_MPH_DTT
$-\log_{10}(p)$
0
1
2
3
4
5
6
7
8
9
10
Chr
1
2
3
4
5
6
7
8
9
10
Chromosome

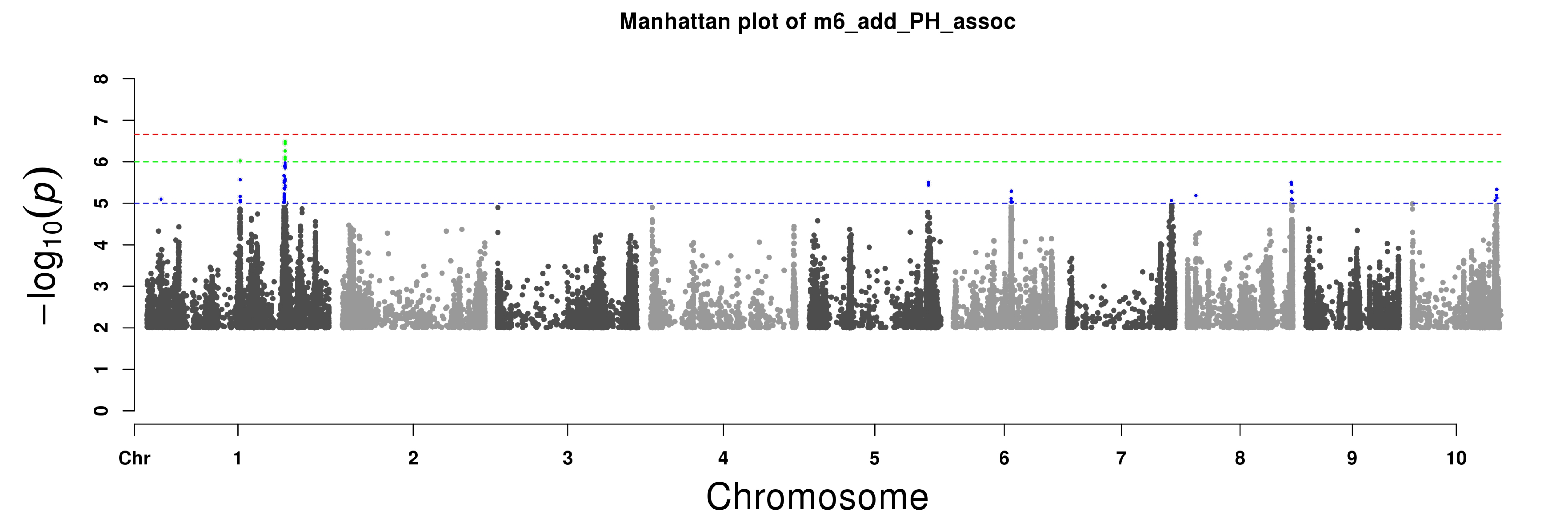

Manhattan plot of m6_add_PH_assoc
$-\log_{10}(p)$
0
1
2
3
4
5
6
7
8
Chr
1
2
3
4
5
6
7
8
9
10
Chromosome

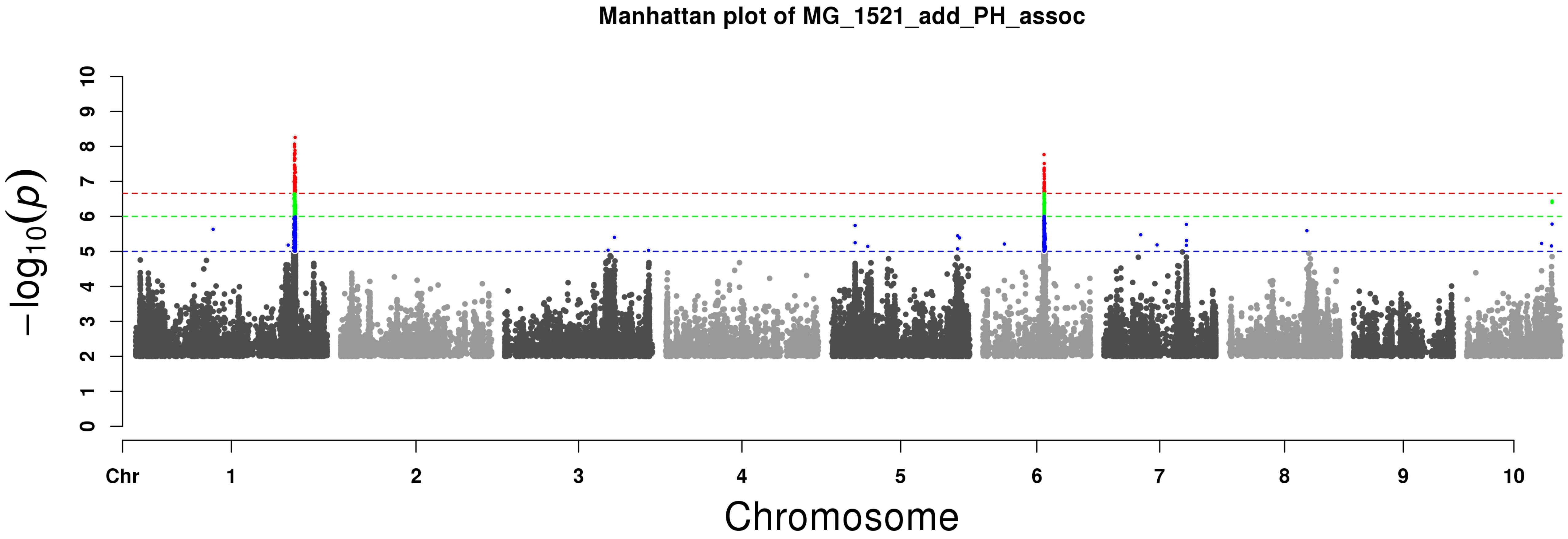

Manhattan plot of MG_1521_add_PH_assoc
$-\log_{10}(p)$
0
1
2
3
4
5
6
7
8
9
10
Chr
1
2
3
4
5
6
7
8
9
10
Chromosome

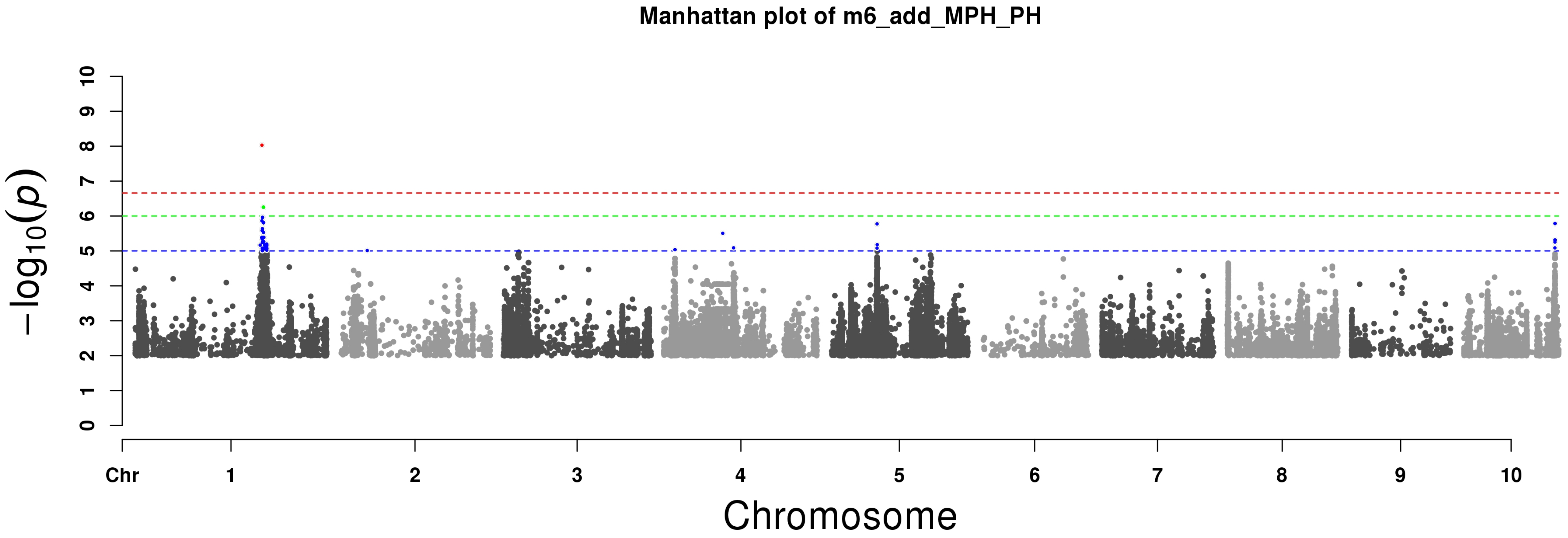

Manhattan plot of m6_add_MPH_PH
$-\log_{10}(p)$
0
1
2
3
4
5
6
7
8
9
10
Chr
1
2
3
4
5
6
7
8
9
10
Chromosome

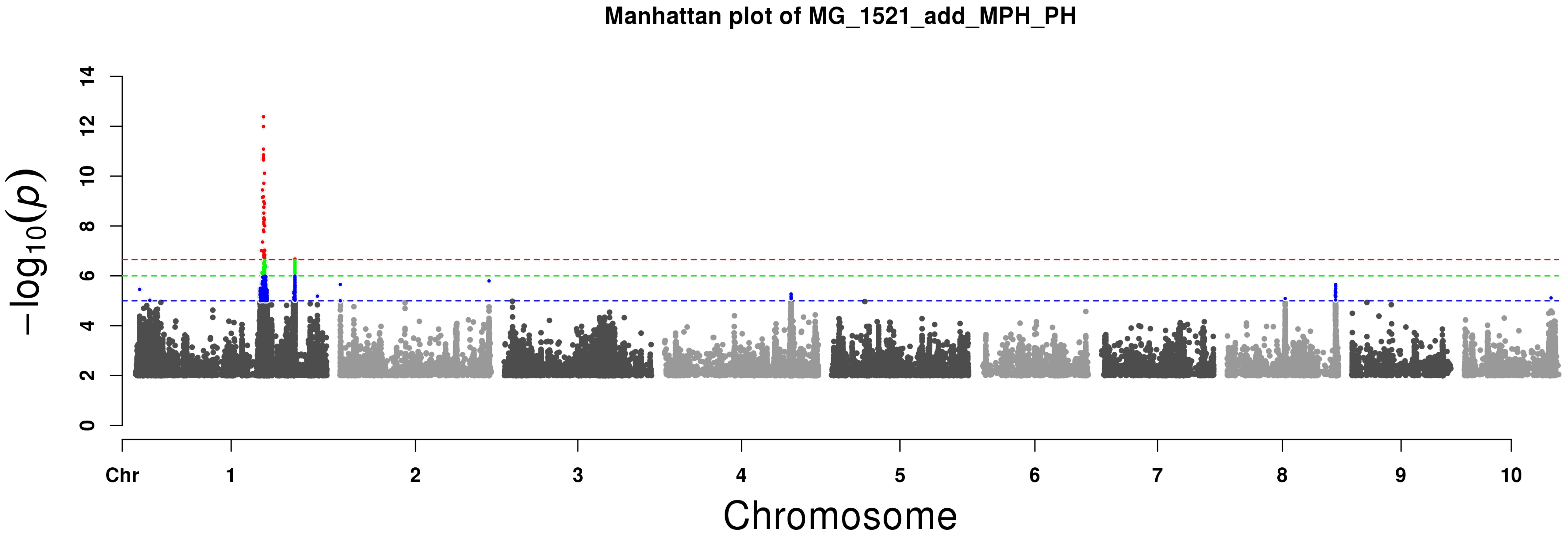

Manhattan plot of MG_1521_add_MPH_PH
−log10(p)
0
2
4
6
8
10
12
14
Chr
1
2
3
4
5
6
7
8
9
10
Chromosome

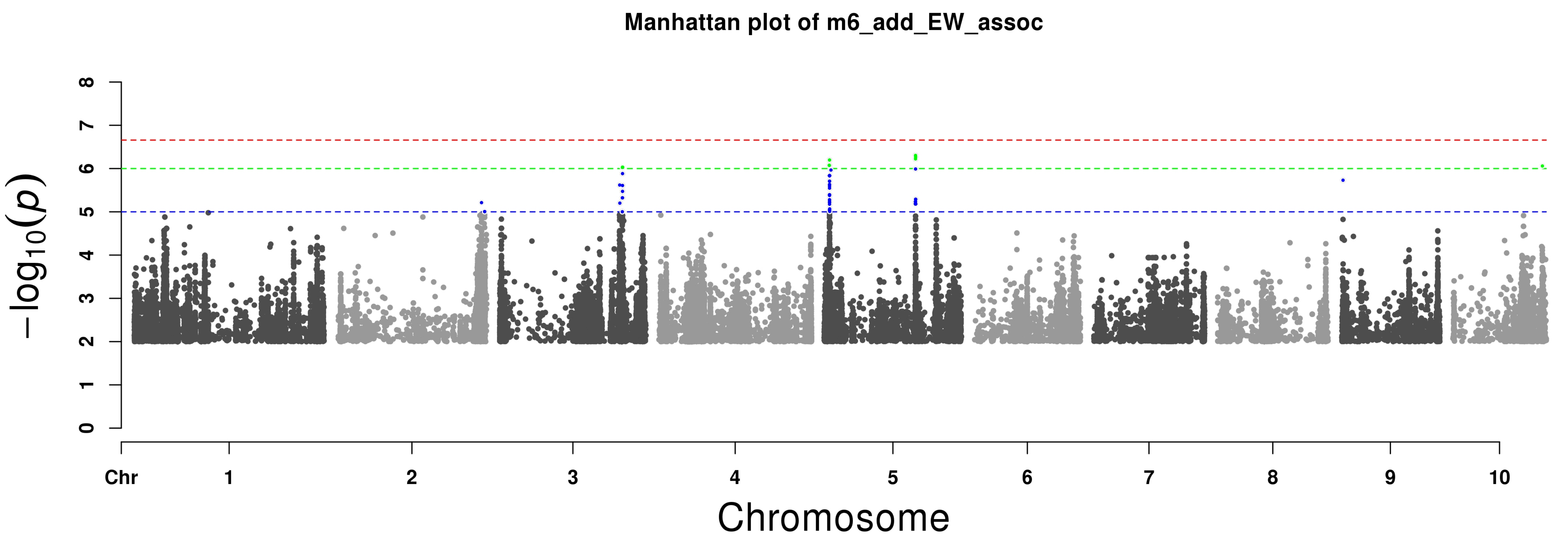

Manhattan plot of m6_add_EW_assoc
0
1
2
3
4
5
6
7
8
$-\log_{10}(p)$
Chr
1
2
3
4
5
6
7
8
9
10
Chromosome

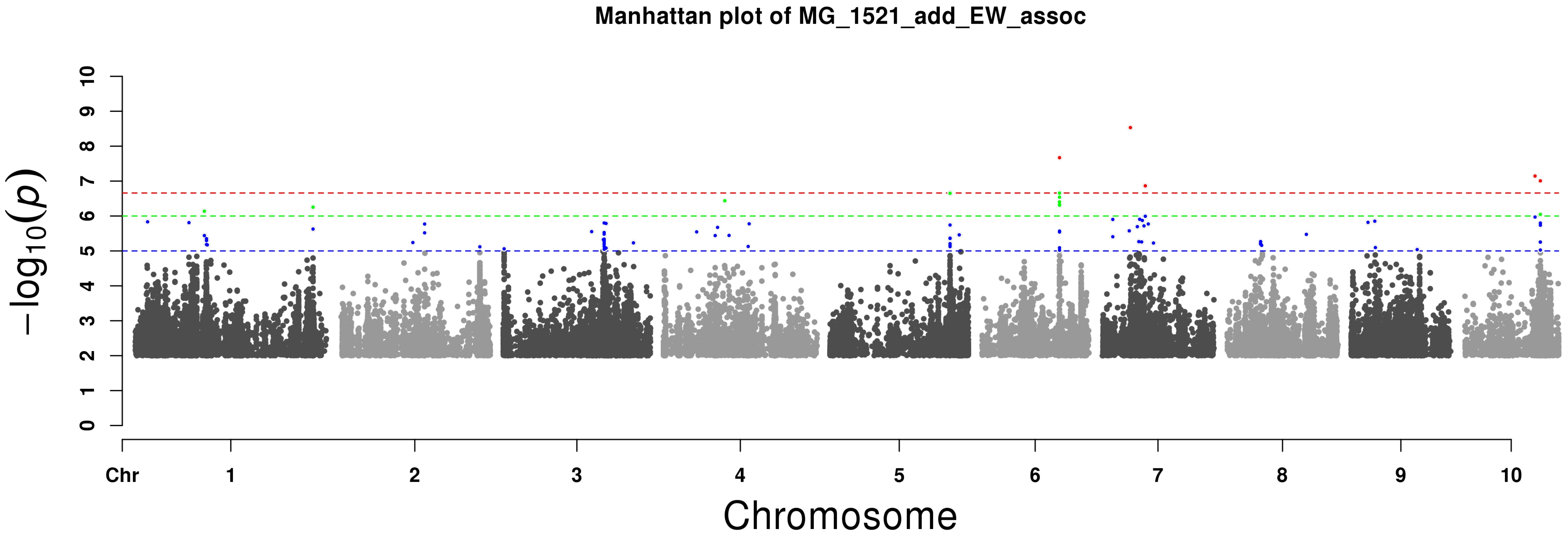
Manhattan plot of MG_1521_add_EW_assoc
$-\log_{10}(p)$
0
1
2
3
4
5
6
7
8
9
10
Chr
1
2
3
4
5
6
7
8
9
10
Chromosome

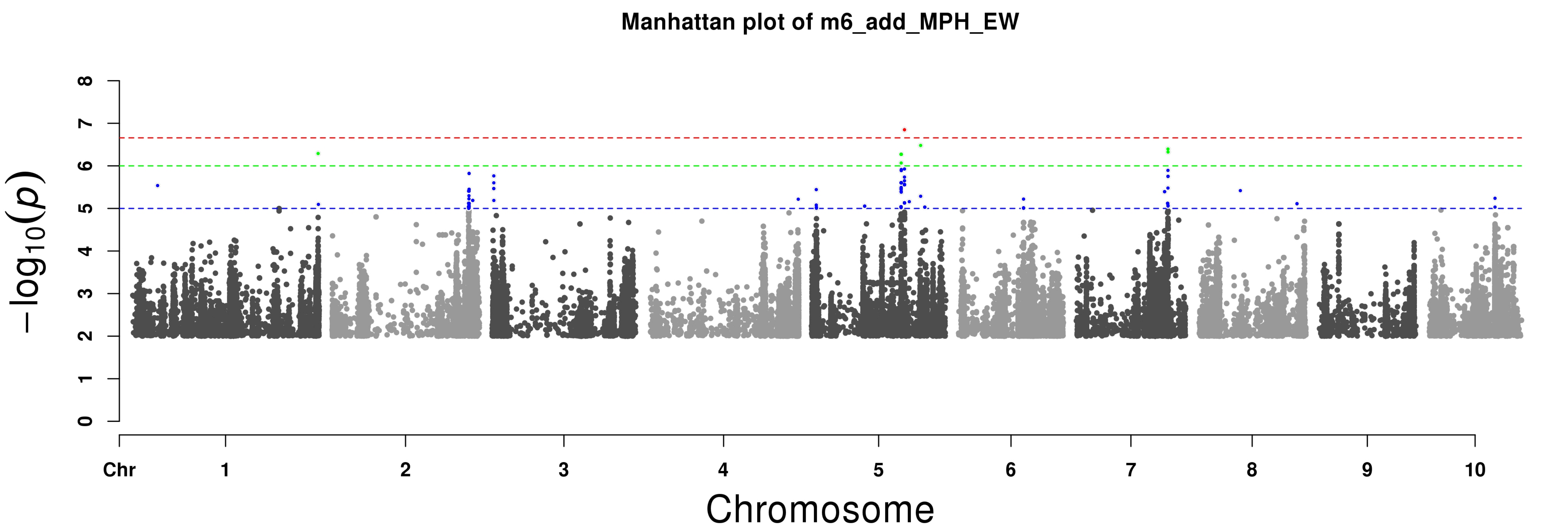

Manhattan plot of m6_add_MPH_EW
$-\log_{10}(p)$
0
1
2
3
4
5
6
7
8
Chr
1
2
3
4
5
6
7
8
9
10
Chromosome

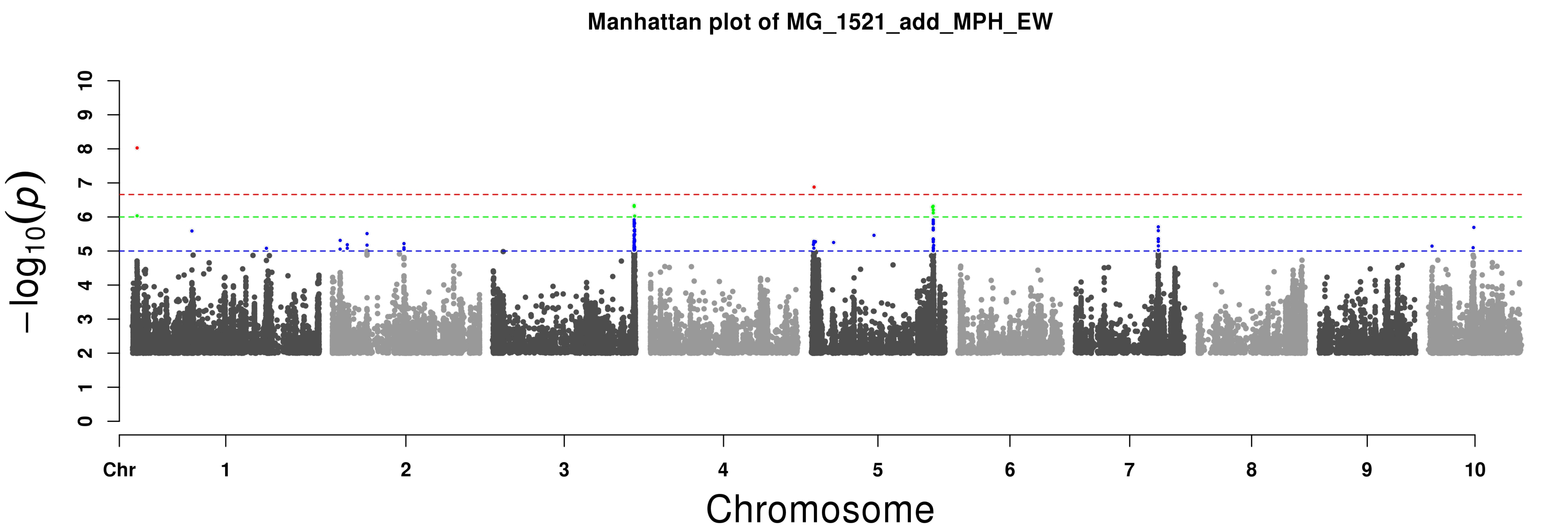

Manhattan plot of MG_1521_add_MPH_EW
$-\log_{10}(p)$
0
1
2
3
4
5
6
7
8
9
10
Chr
1
2
3
4
5
6
7
8
9
10
Chromosome

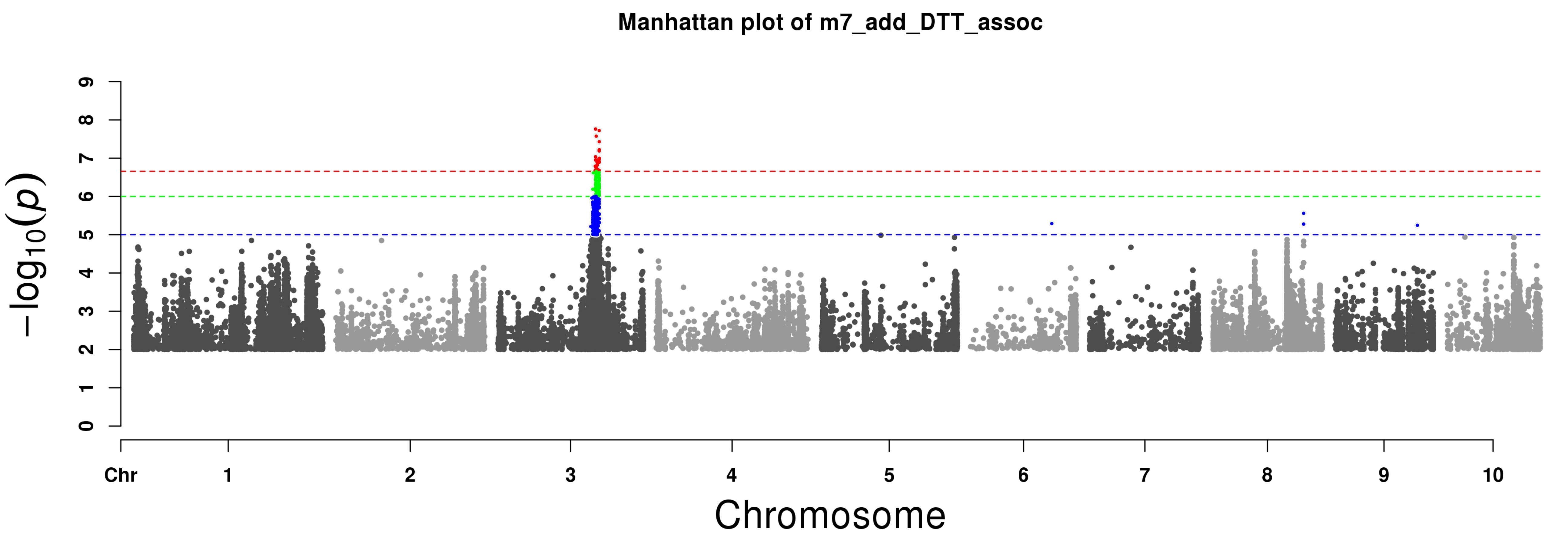
Manhattan plot of m7_add_DTT_assoc
$-\log_{10}(p)$
0
1
2
3
4
5
6
7
8
9
Chr
1
2
3
4
5
6
7
8
9
10
Chromosome

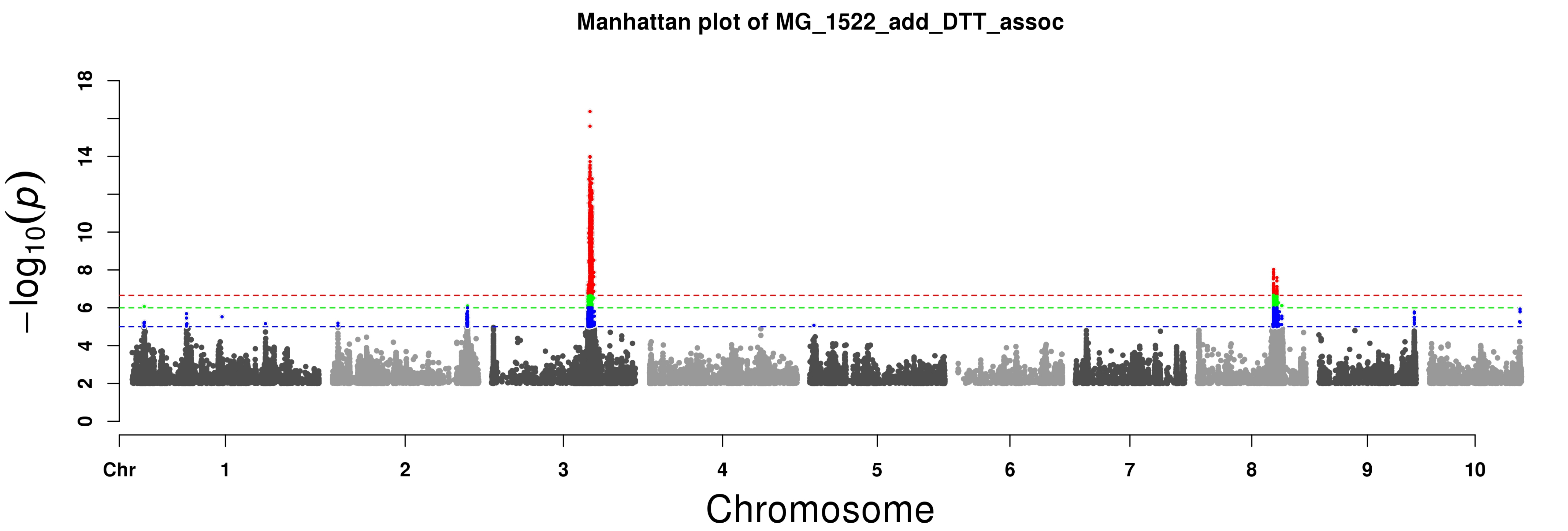

Manhattan plot of MG_1522_add_DTT_assoc
$-\log_{10}(p)$
0
2
4
6
8
10
14
18
Chr
1
2
3
4
5
6
7
8
9
10
Chromosome

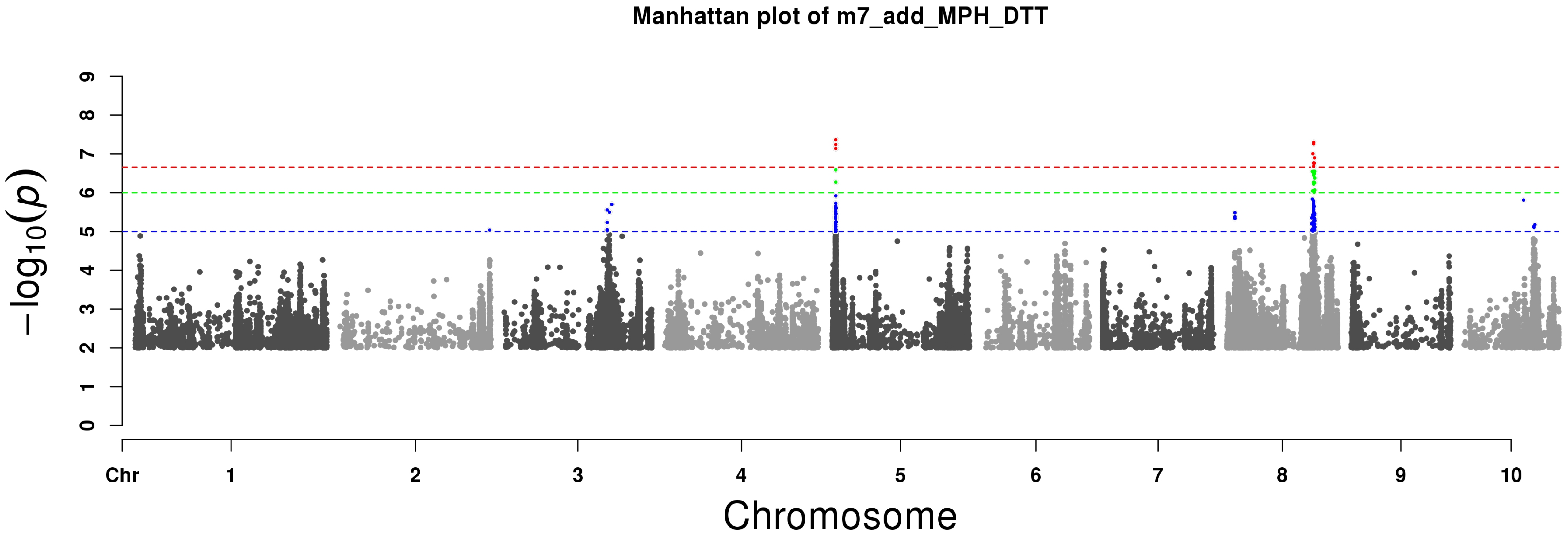

Manhattan plot of m7_add_MPH_DTT
$-\log_{10}(p)$
0
1
2
3
4
5
6
7
8
9
Chr
1
2
3
4
5
6
7
8
9
10
Chromosome

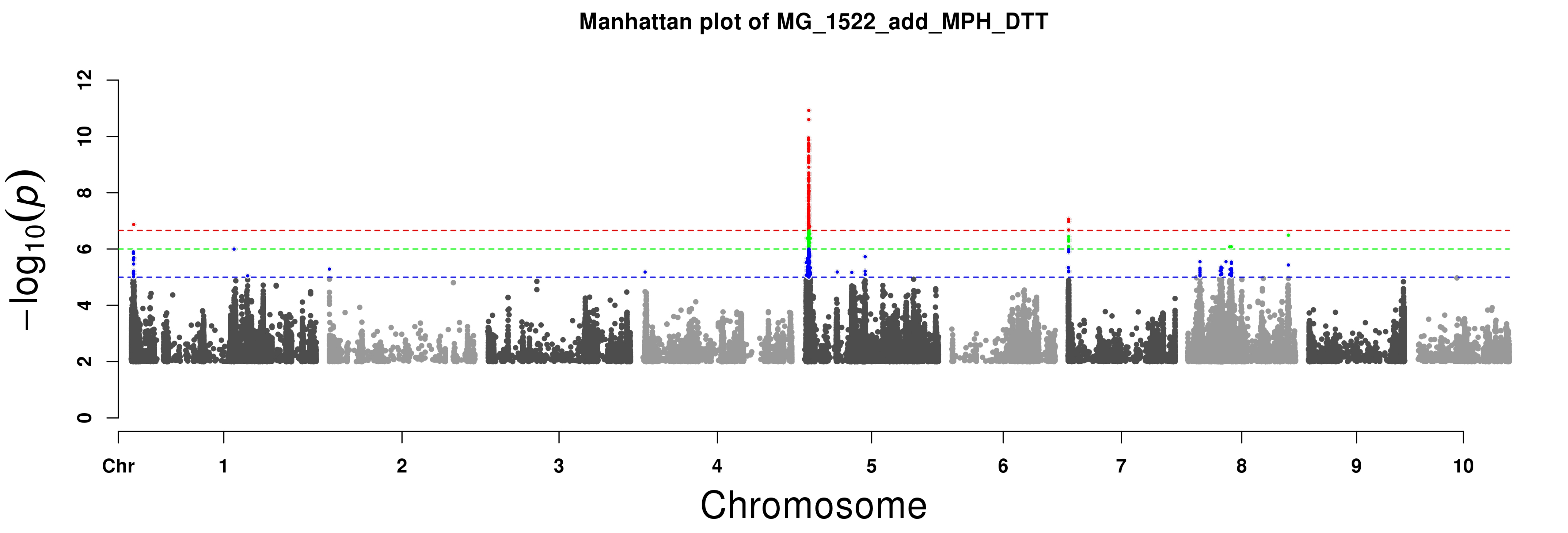

Manhattan plot of MG_1522_add_MPH_DTT
$-\log_{10}(p)$
0
2
4
6
8
10
12
Chr
1
2
3
4
5
6
7
8
9
10
Chromosome

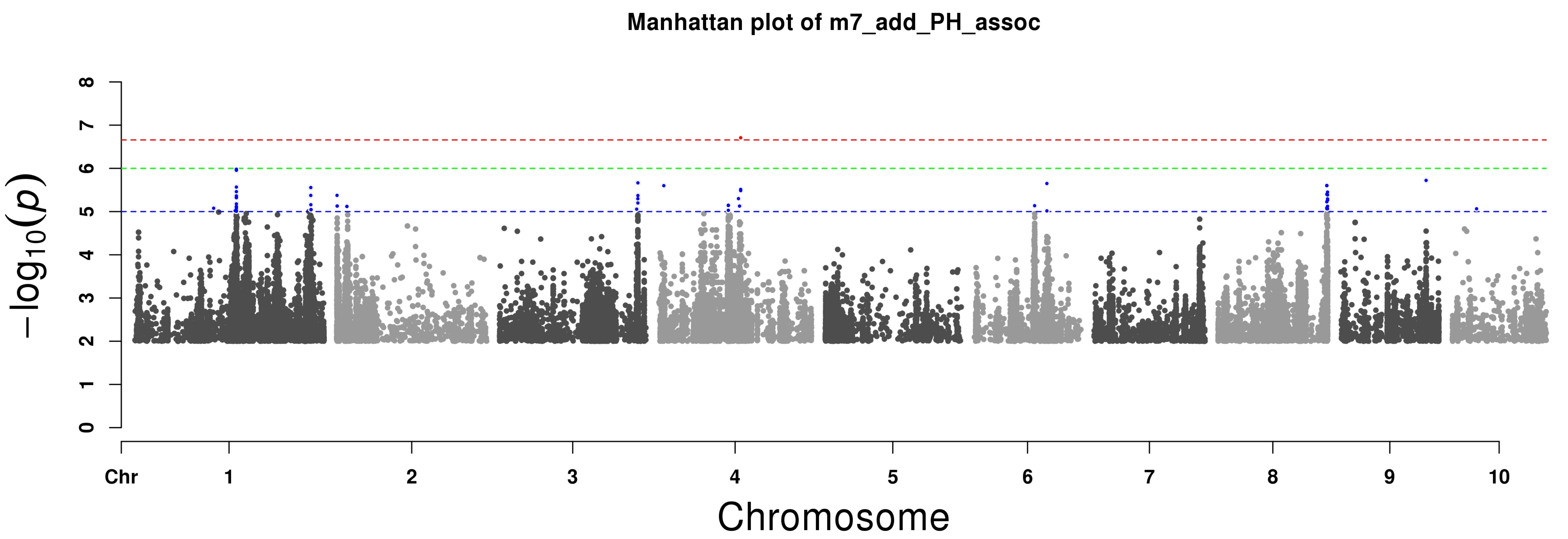
Manhattan plot of m7_add_PH_assoc
$-\log_{10}(p)$
0
1
2
3
4
5
6
7
8
Chr
1
2
3
4
5
6
7
8
9
10
Chromosome

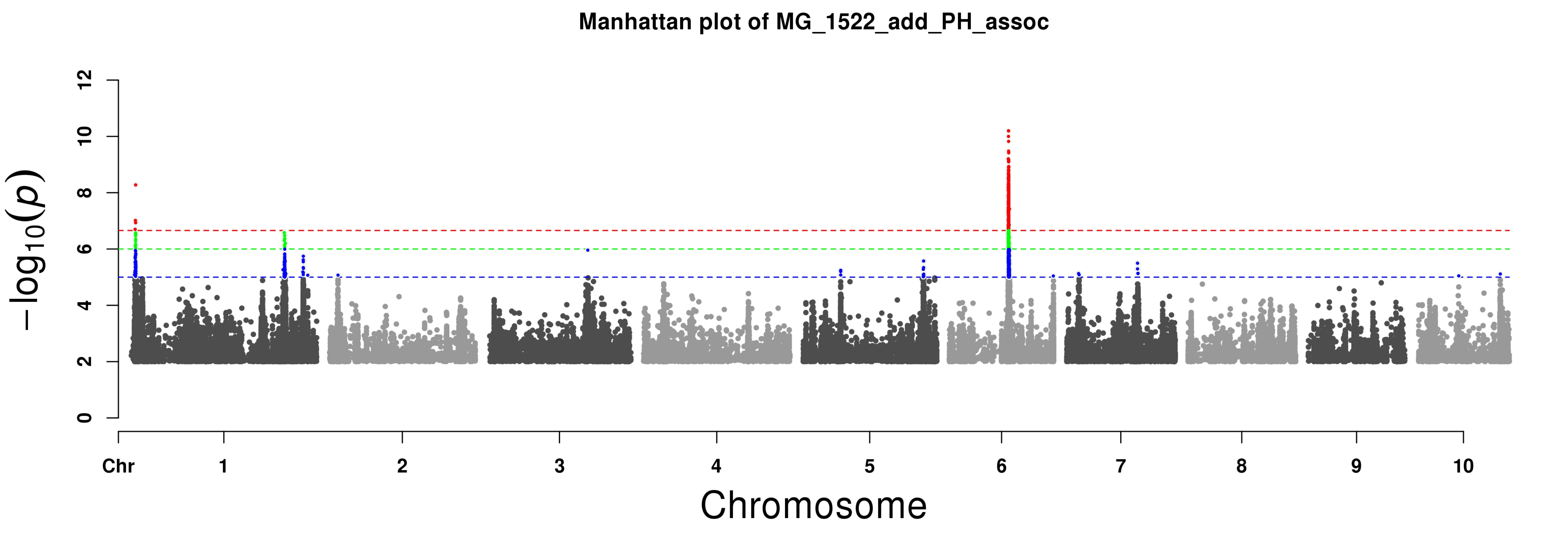
Manhattan plot of MG_1522_add_PH_assoc
$-\log_{10}(p)$
0
2
4
6
8
10
12
Chr
1
2
3
4
5
6
7
8
9
10
Chromosome

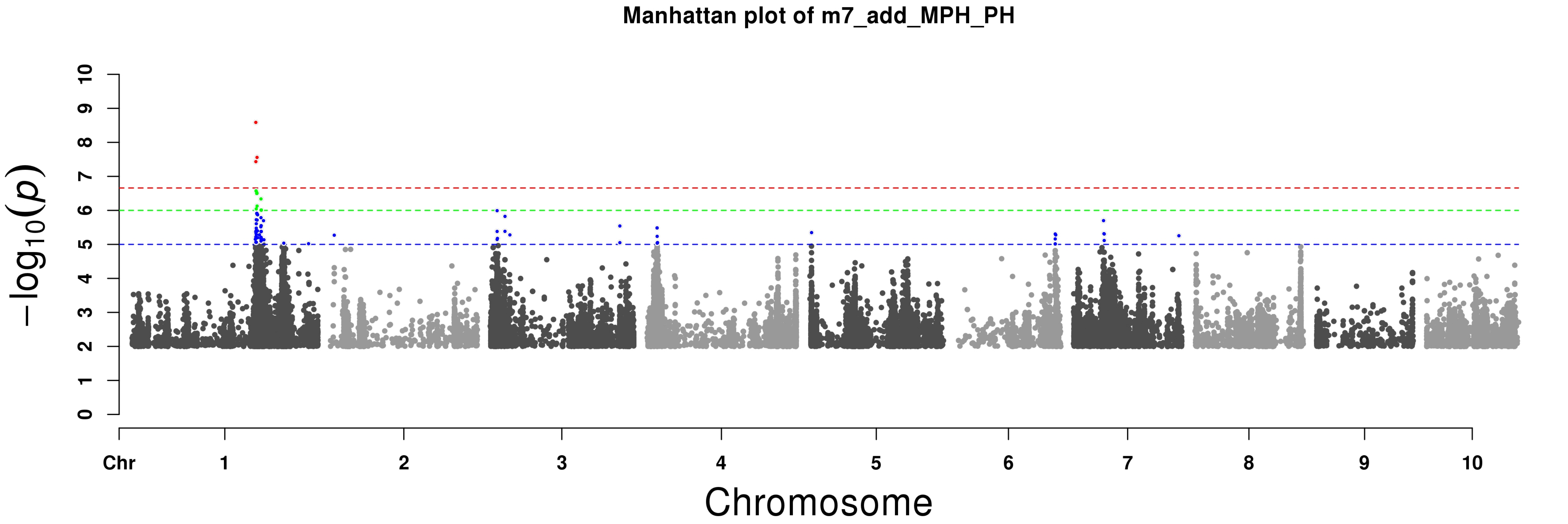

Manhattan plot of m7_add_MPH_PH
$-\log_{10}(p)$
0
1
2
3
4
5
6
7
8
9
10
Chr
1
2
3
4
5
6
7
8
9
10
Chromosome

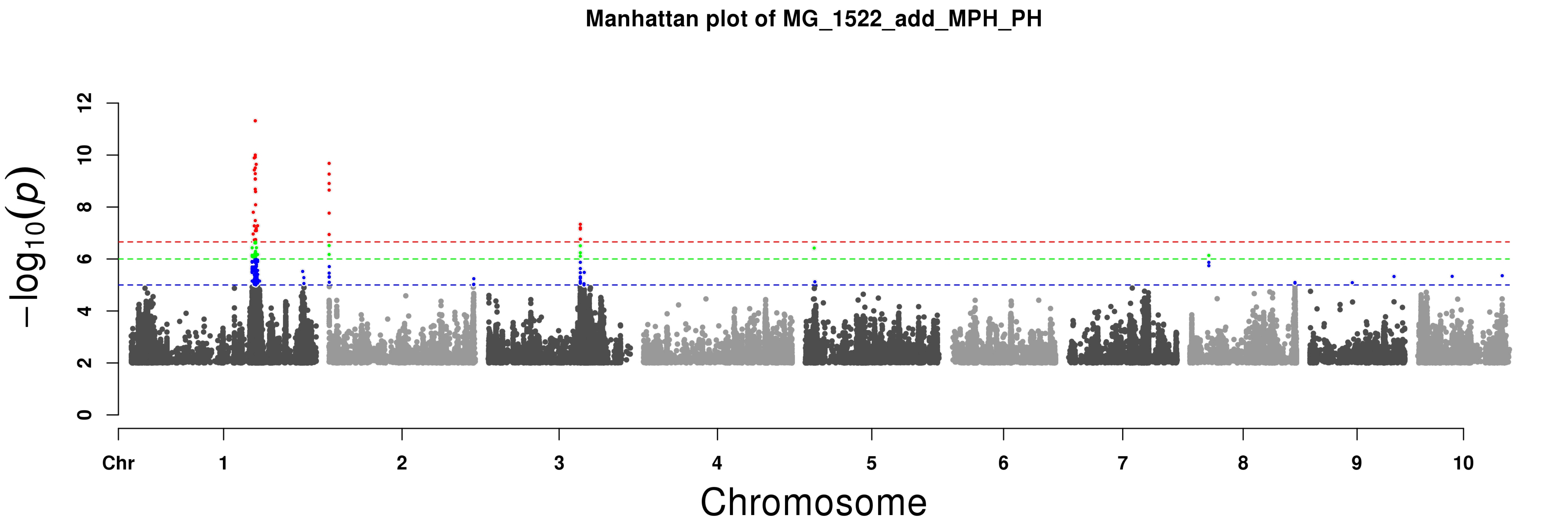
Manhattan plot of MG_1522_add_MPH_PH
$-\log_{10}(p)$
0
2
4
6
8
10
12
Chr
1
2
3
4
5
6
7
8
9
10
Chromosome

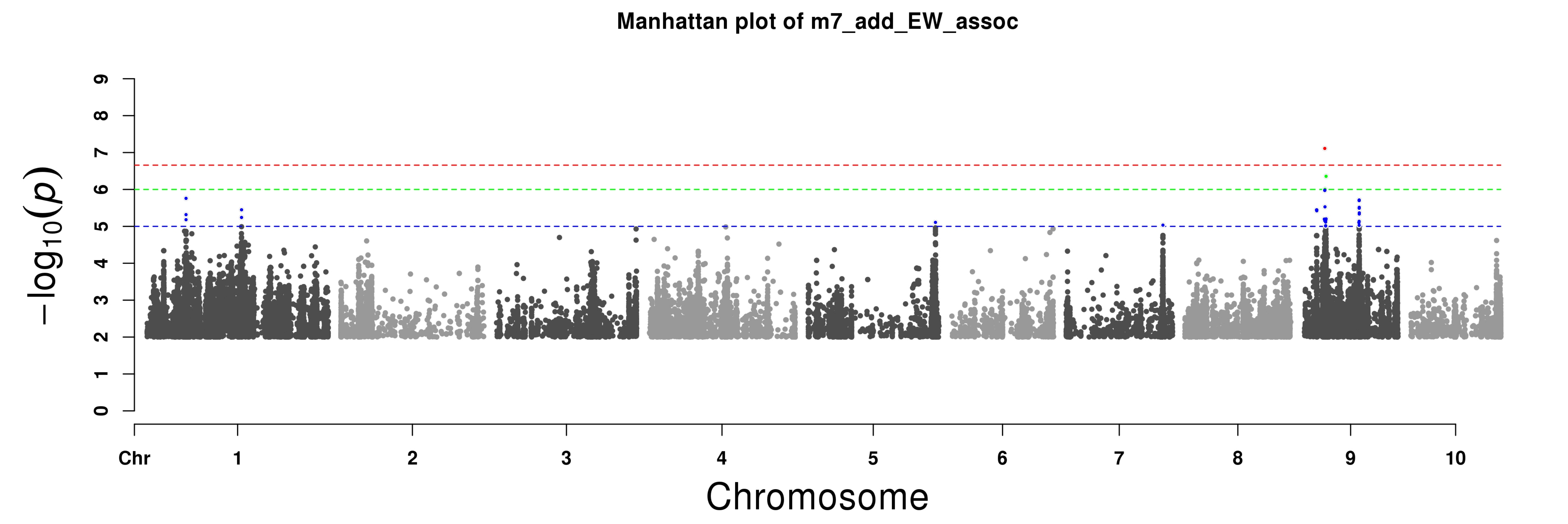

Manhattan plot of m7_add_EW_assoc
$-\log_{10}(p)$
0
1
2
3
4
5
6
7
8
9
Chr
1
2
3
4
5
6
7
8
9
10
Chromosome

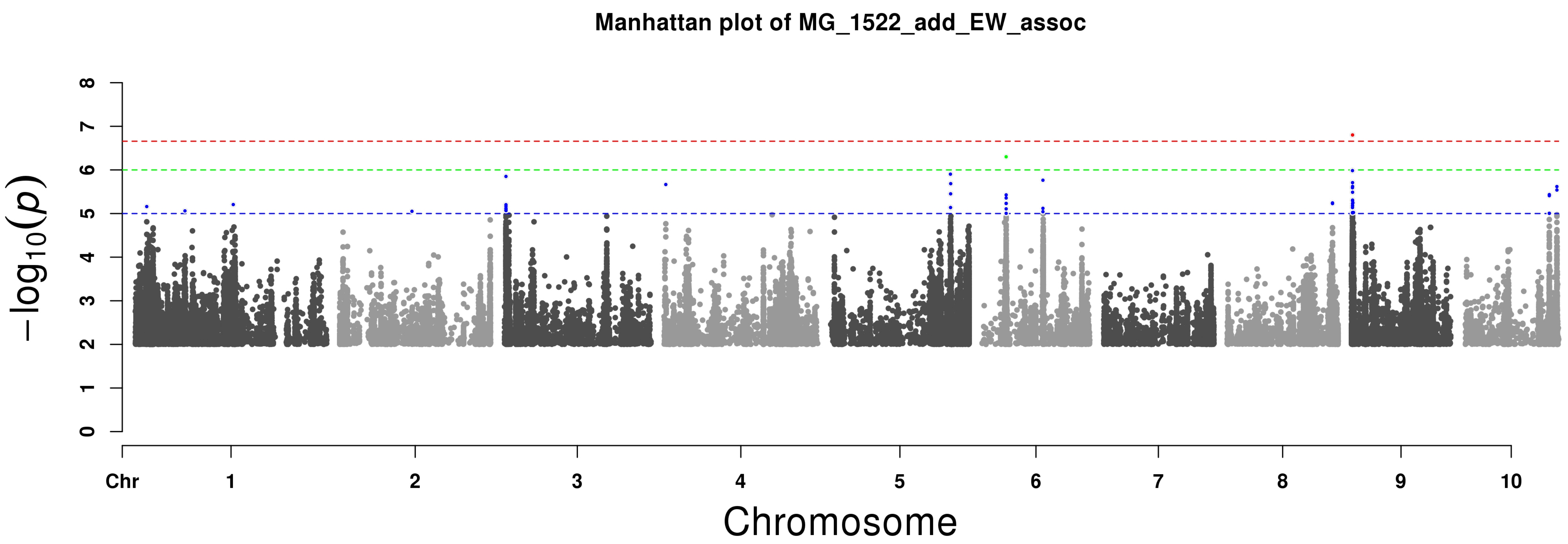

Manhattan plot of MG_1522_add_EW_assoc
−log10(p)
0
1
2
3
4
5
6
7
8
Chr
1
2
3
4
5
6
7
8
9
10
Chromosome

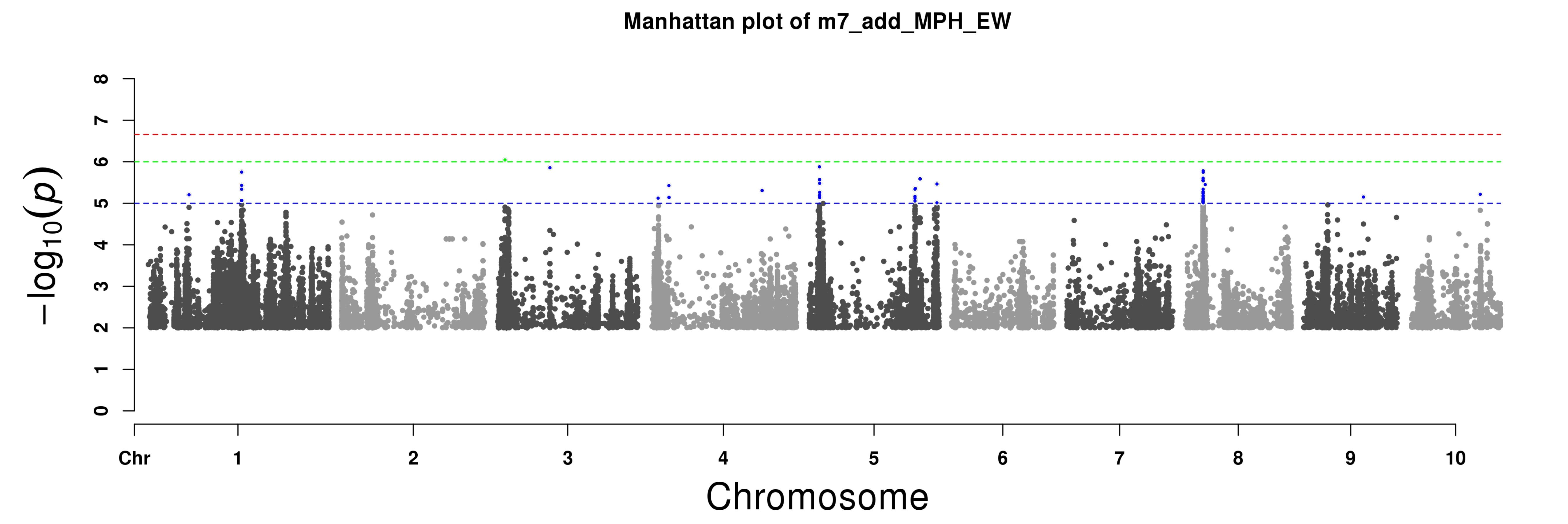

Manhattan plot of m7_add_MPH_EW
$-\log_{10}(p)$
0
1
2
3
4
5
6
7
8
Chr
1
2
3
4
5
6
7
8
9
10
Chromosome

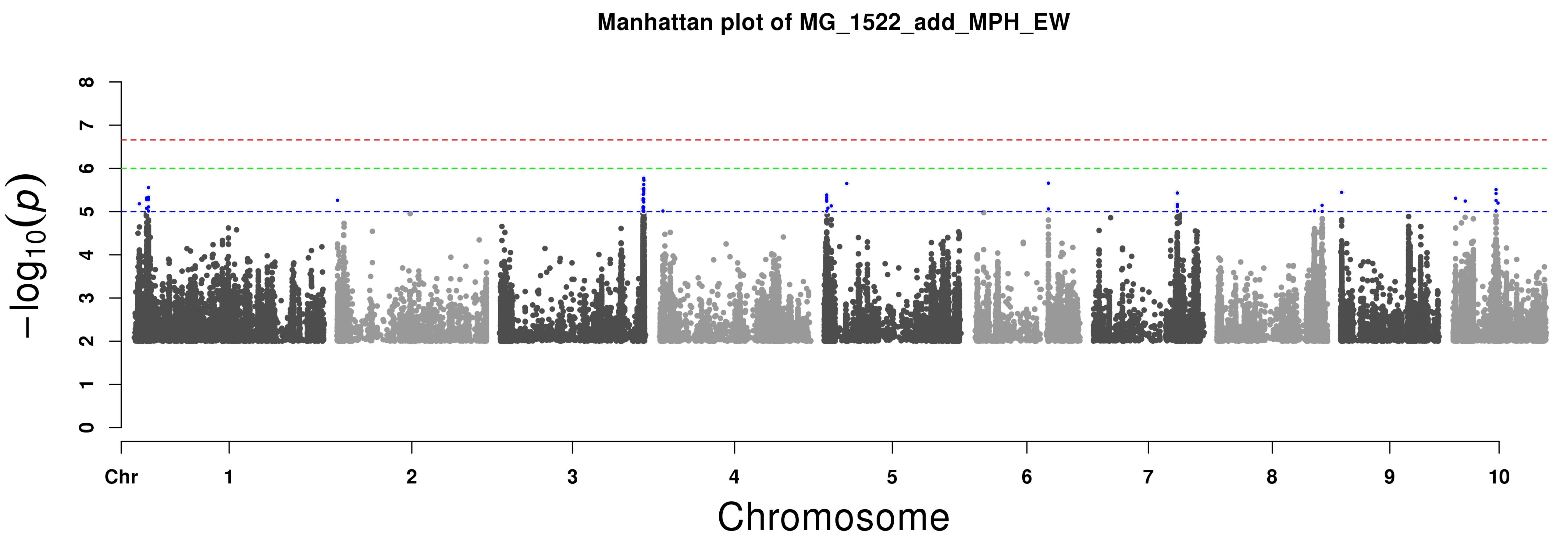

Manhattan plot of MG_1522_add_MPH_EW
$-\log_{10}(p)$
0
1
2
3
4
5
6
7
8
Chr
1
2
3
4
5
6
7
8
9
10
Chromosome

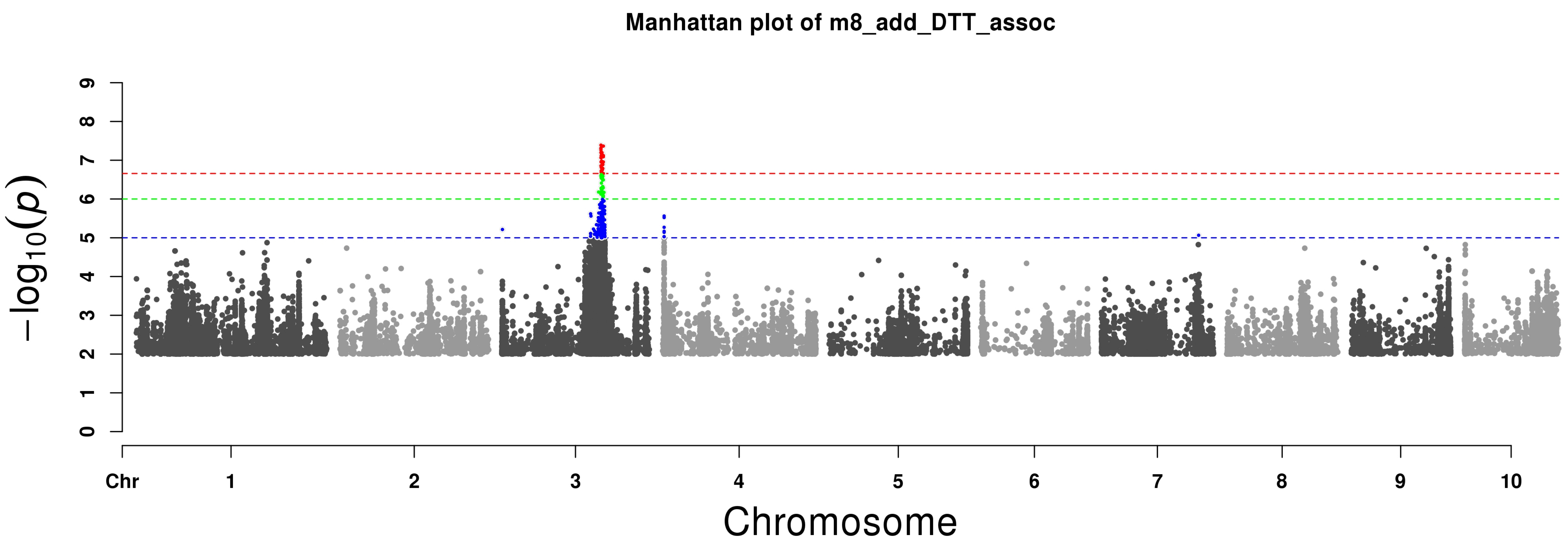

Manhattan plot of m8_add_DTT_assoc
−log10(p)
0
1
2
3
4
5
6
7
8
9
Chr
1
2
3
4
5
6
7
8
9
10
Chromosome

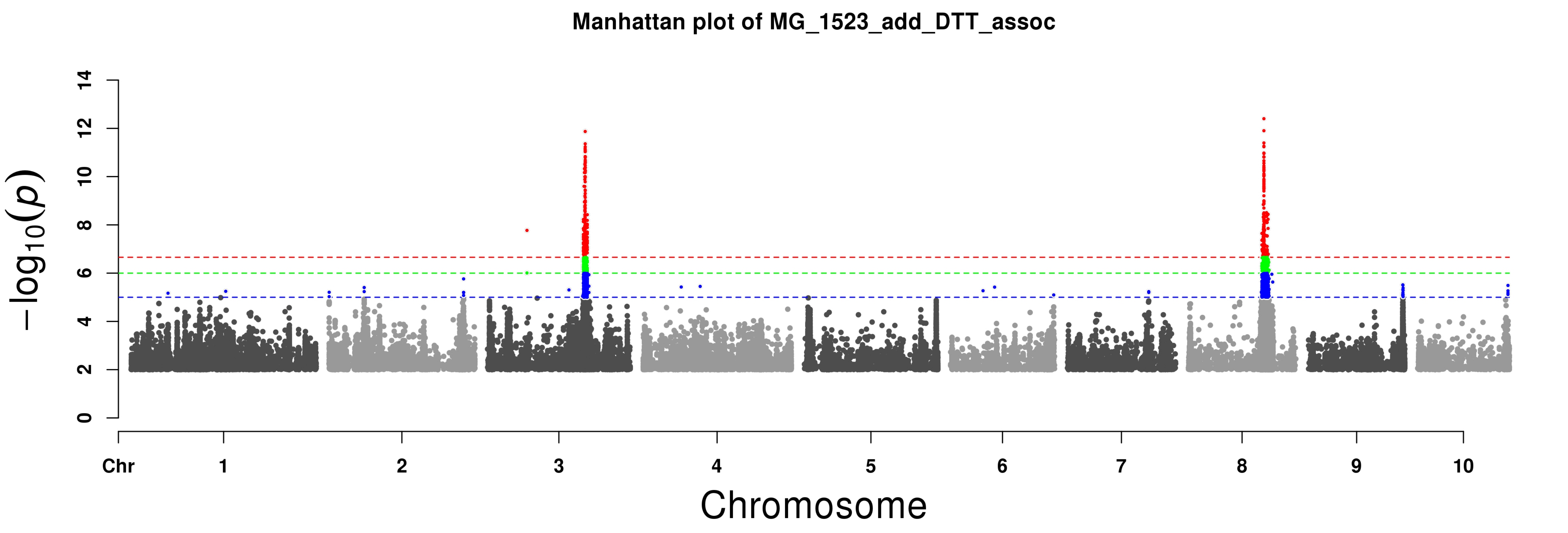

Manhattan plot of MG_1523_add_DTT_assoc
$-\log_{10}(p)$
0
2
4
6
8
10
12
14
Chr
1
2
3
4
5
6
7
8
9
10
Chromosome

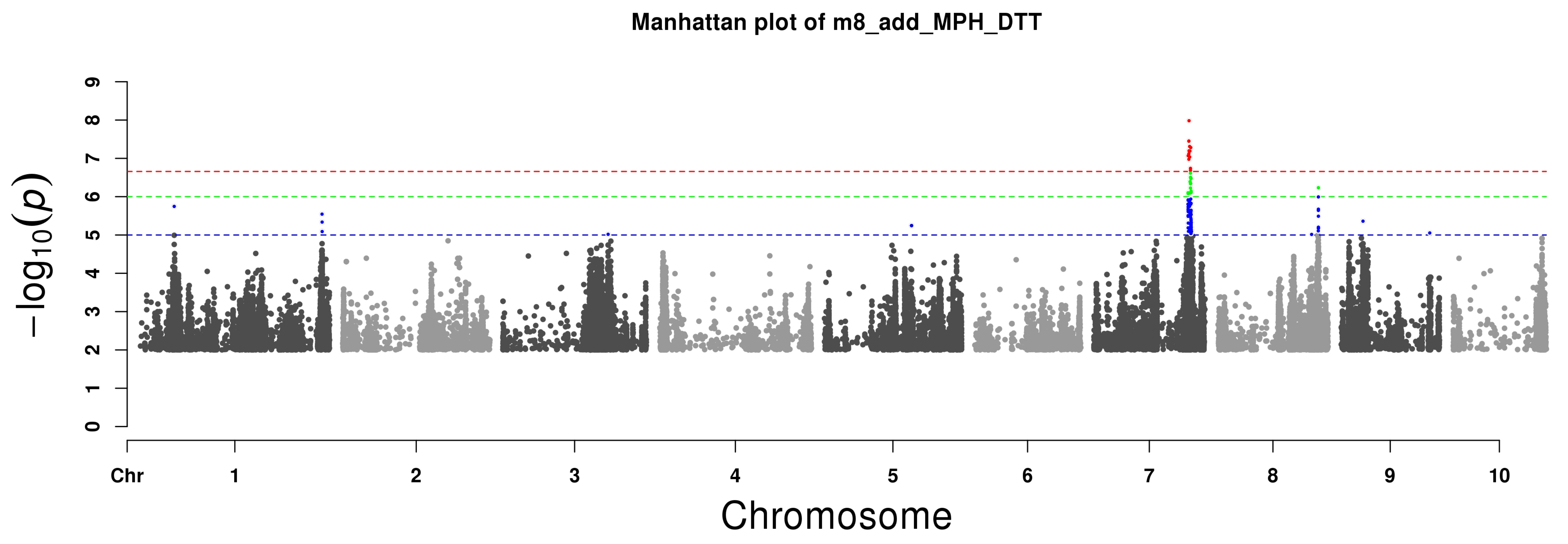

Manhattan plot of m8_add_MPH_DTT
−log10(p)
0
1
2
3
4
5
6
7
8
9
Chr
1
2
3
4
5
6
7
8
9
10
Chromosome

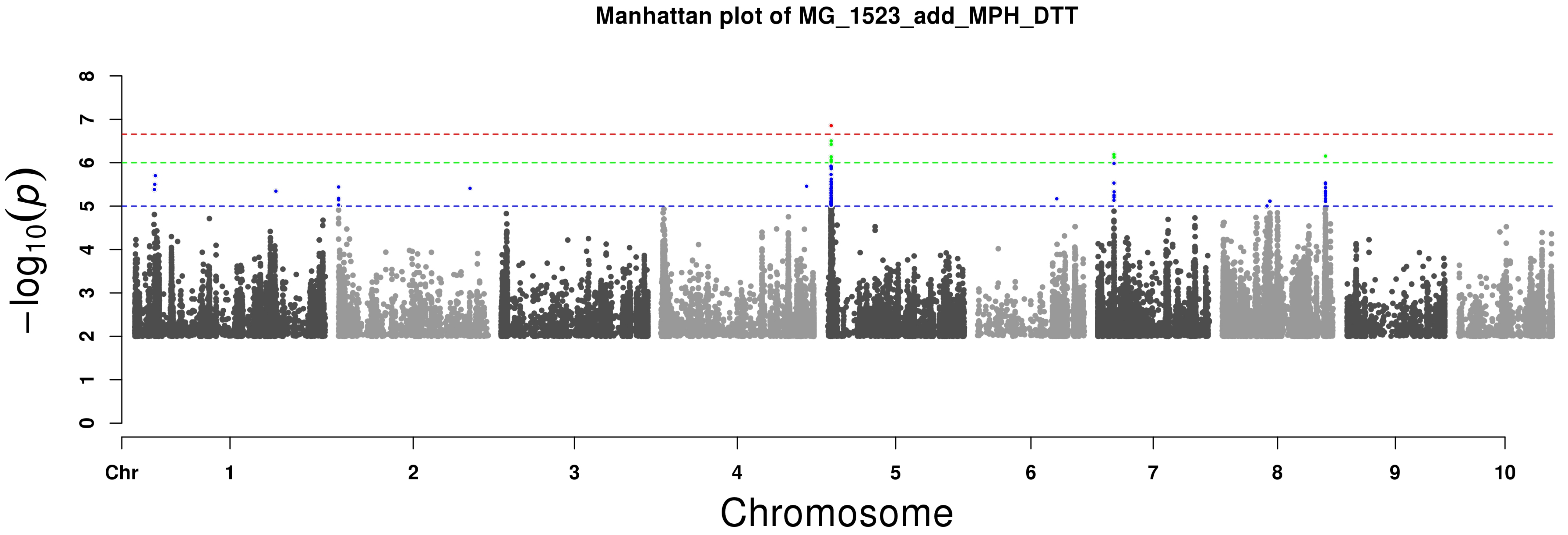

Manhattan plot of MG_1523_add_MPH_DTT
$-\log_{10}(p)$
0
1
2
3
4
5
6
7
8
Chr
1
2
3
4
5
6
7
8
9
10
Chromosome

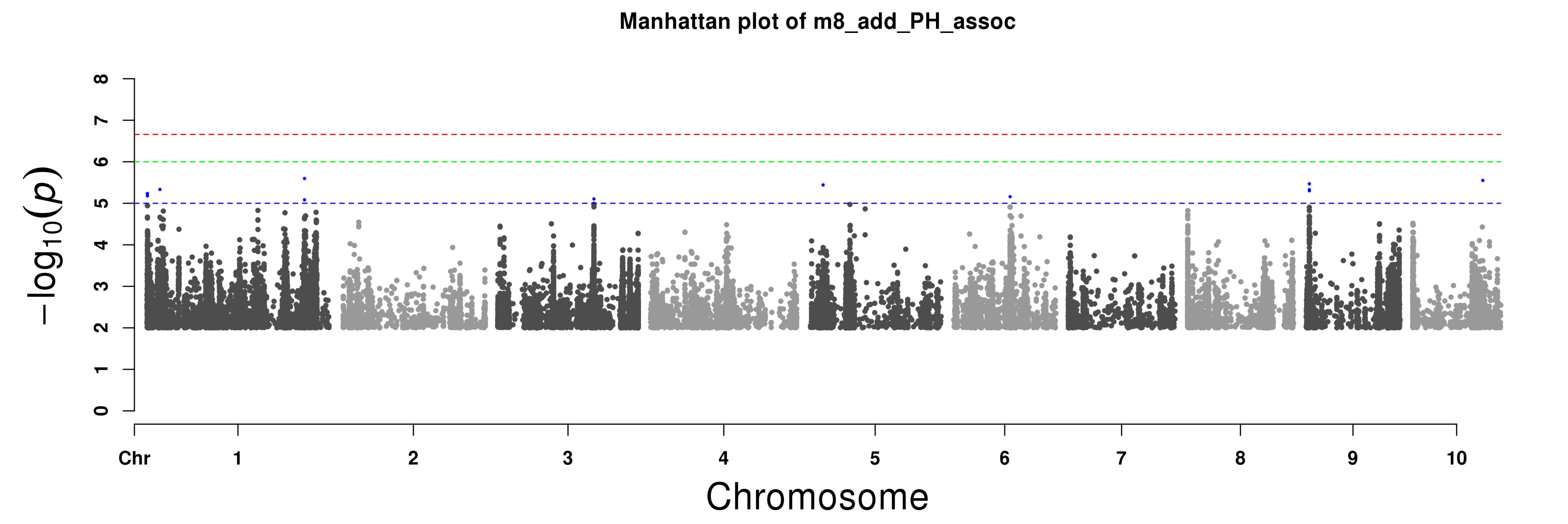

Manhattan plot of m8_add_PH_assoc
$-\log_{10}(p)$
0
1
2
3
4
5
6
7
8
Chr
1
2
3
4
5
6
7
8
9
10
Chromosome

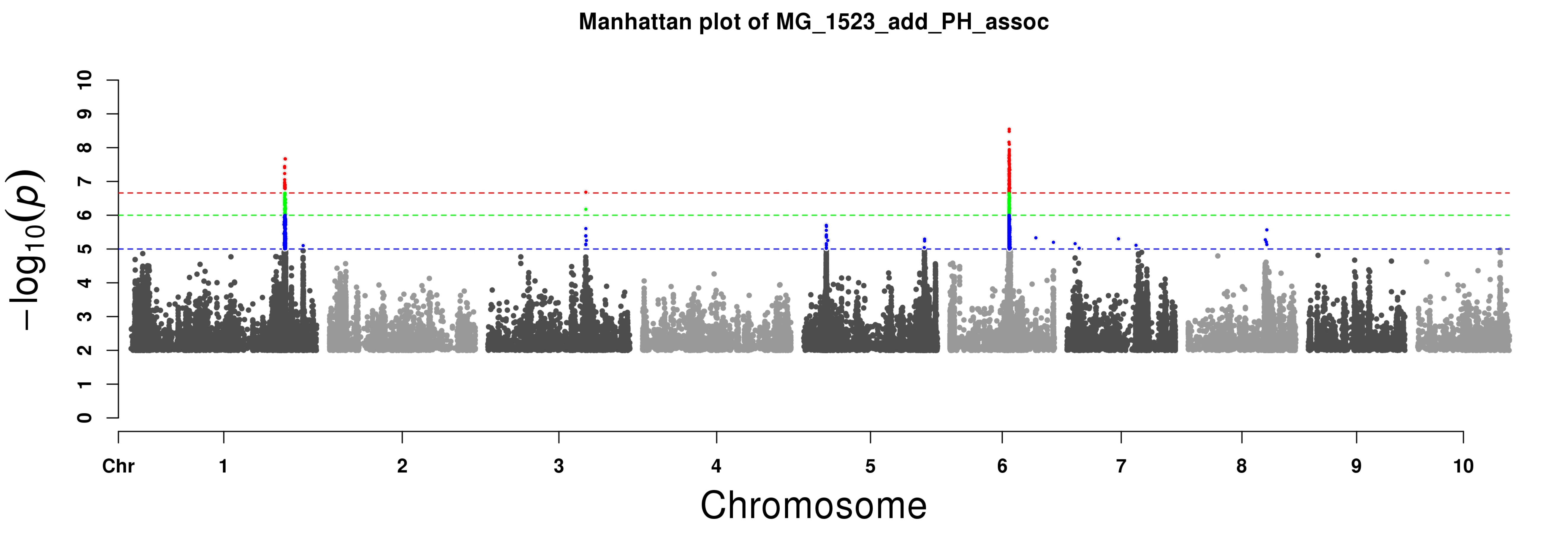

Manhattan plot of MG_1523_add_PH_assoc
$-\log_{10}(p)$
0
1
2
3
4
5
6
7
8
9
10
Chr
1
2
3
4
5
6
7
8
9
10
Chromosome

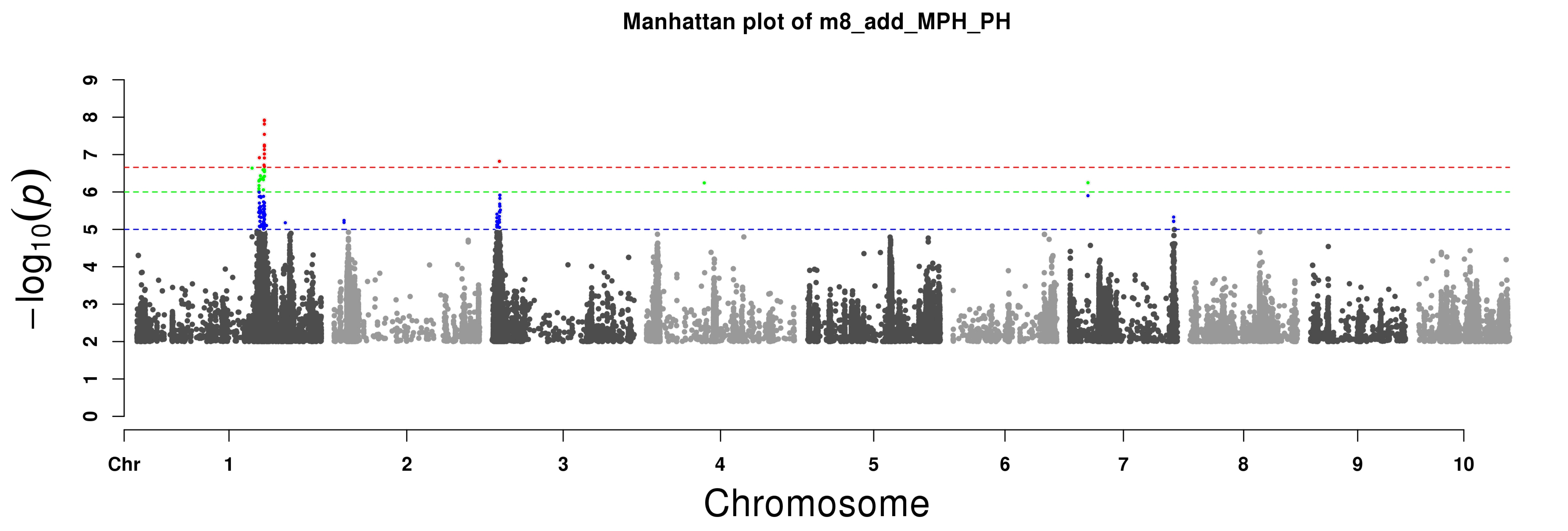

Manhattan plot of m8_add_MPH_PH
$-\log_{10}(p)$
0
1
2
3
4
5
6
7
8
9
Chr
1
2
3
4
5
6
7
8
9
10
Chromosome

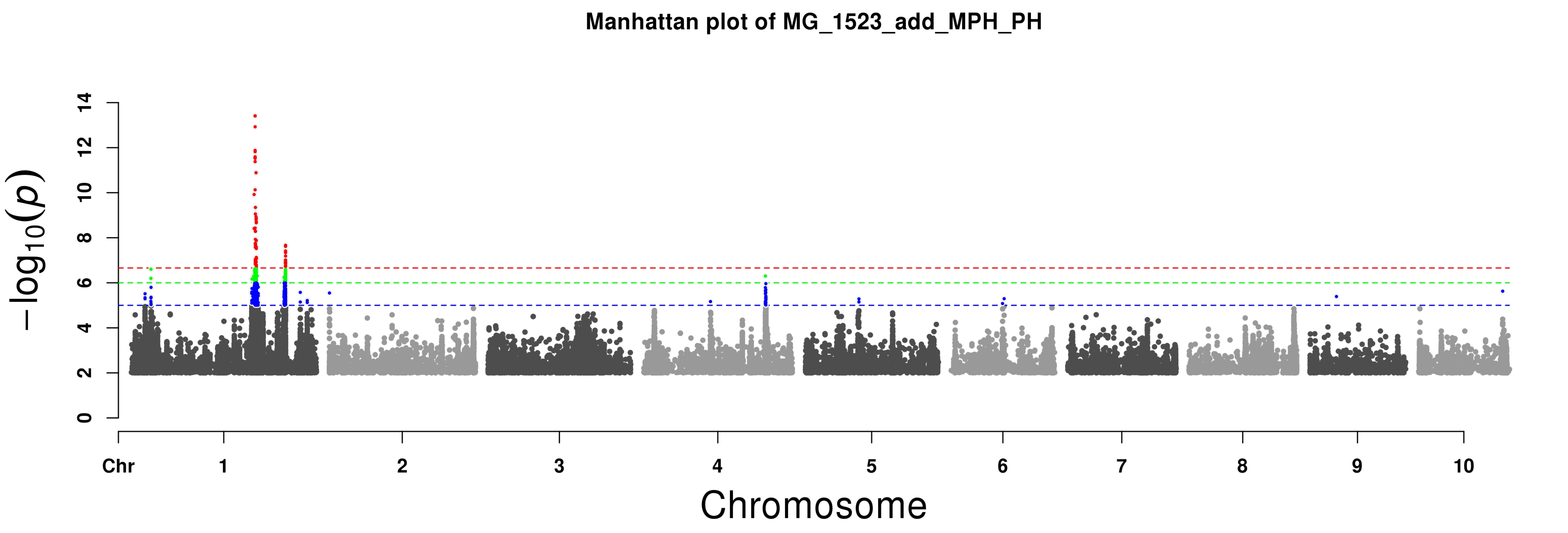
Manhattan plot of MG_1523_add_MPH_PH
$-\log_{10}(p)$
0
2
4
6
8
10
12
14
Chr
1
2
3
4
5
6
7
8
9
10
Chromosome

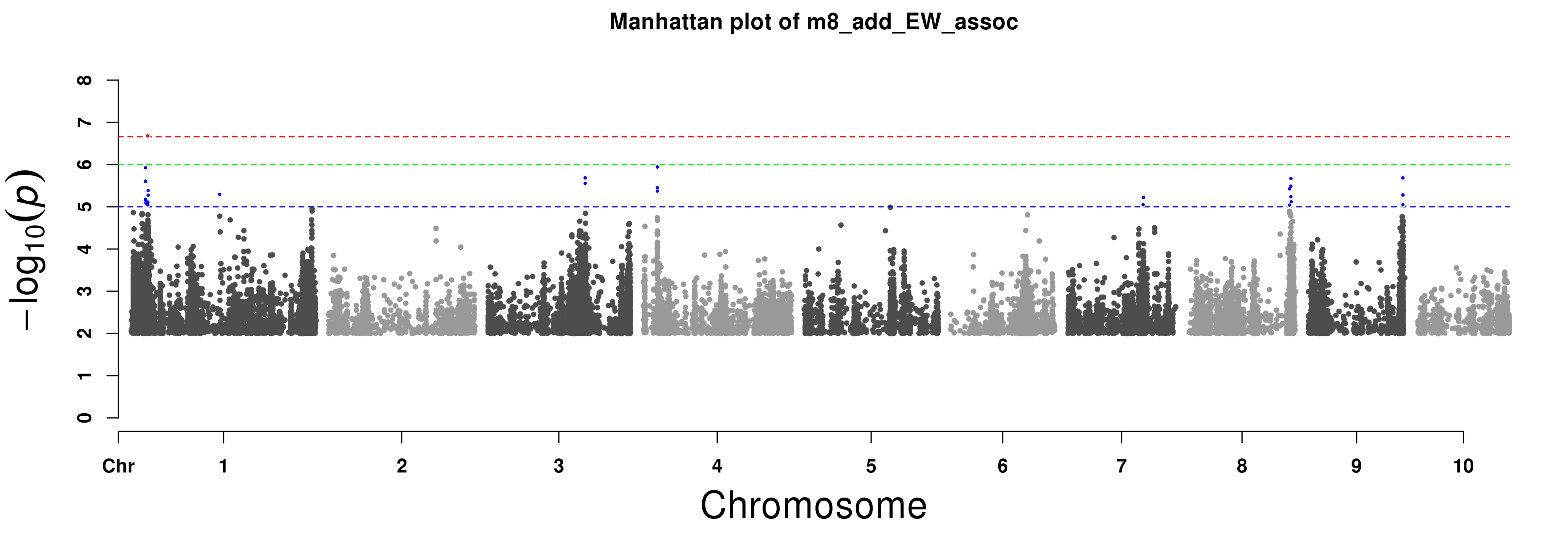

Manhattan plot of m8_add_EW_assoc
$-\log_{10}(p)$
0
1
2
3
4
5
6
7
8
Chr
1
2
3
4
5
6
7
8
9
10
Chromosome

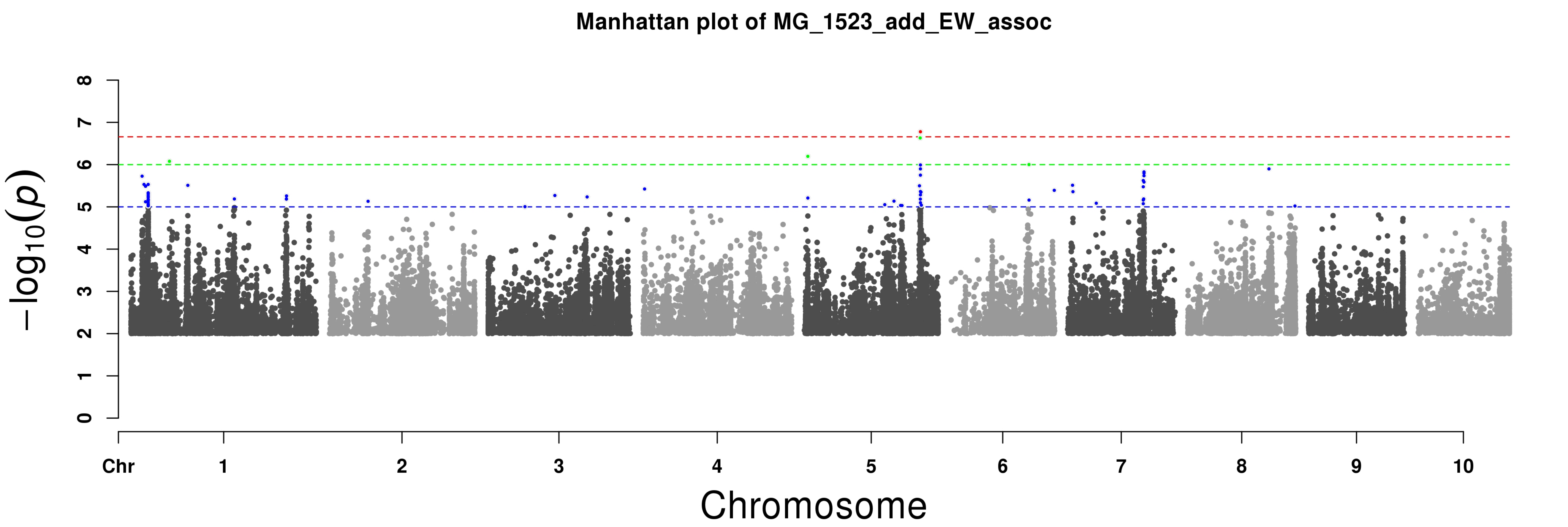
Manhattan plot of MG_1523_add_EW_assoc
−log10(p)
0
1
2
3
4
5
6
7
8
Chr
1
2
3
4
5
6
7
8
9
10
Chromosome

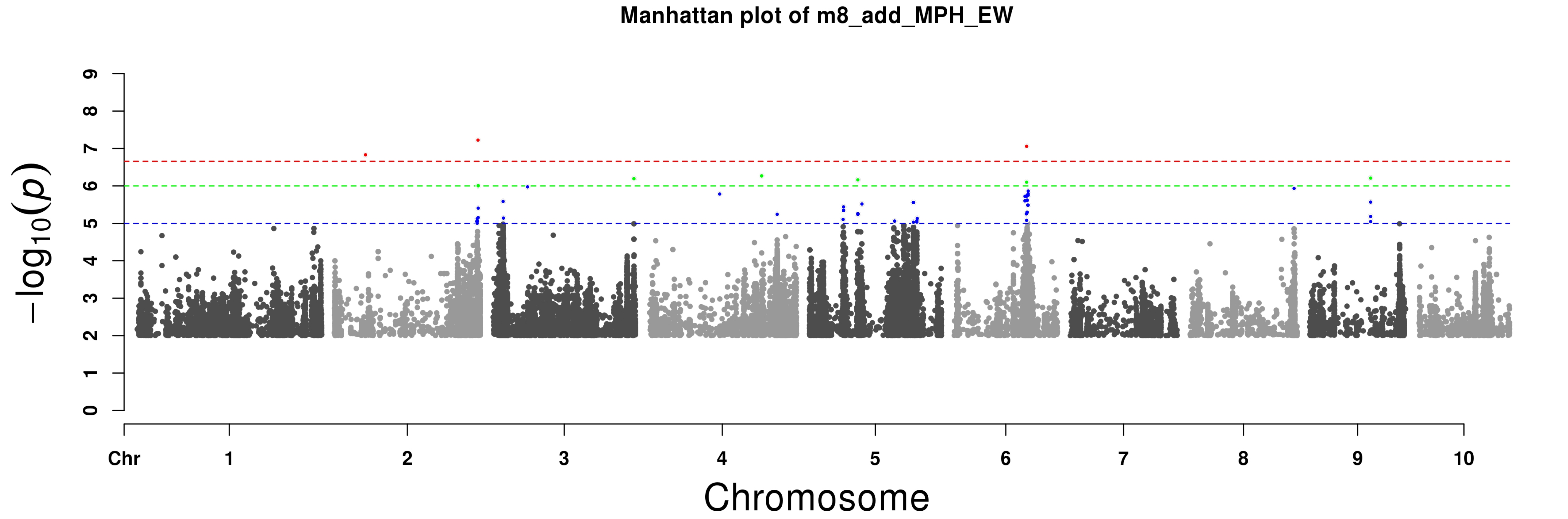

Manhattan plot of m8_add_MPH_EW
$-\log_{10}(p)$
0
1
2
3
4
5
6
7
8
9
Chr
1
2
3
4
5
6
7
8
9
10
Chromosome

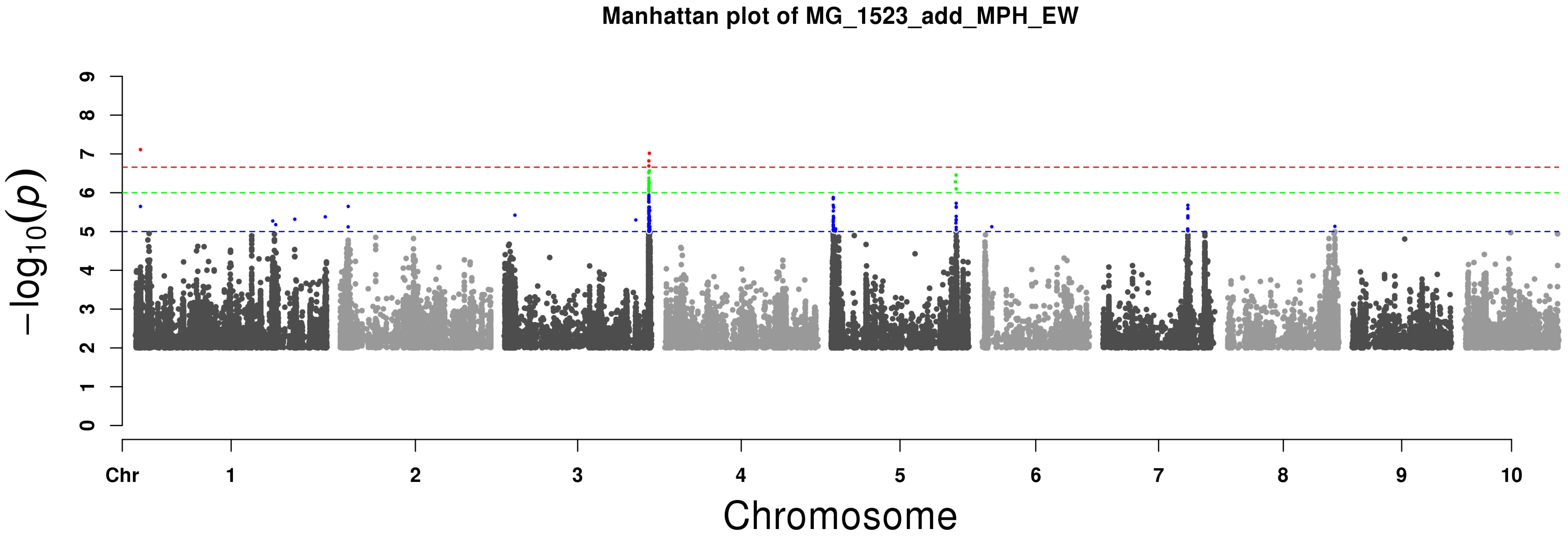
Manhattan plot of MG_1523_add_MPH_EW
$-\log_{10}(p)$
0
1
2
3
4
5
6
7
8
9
Chr
1
2
3
4
5
6
7
8
9
10
Chromosome

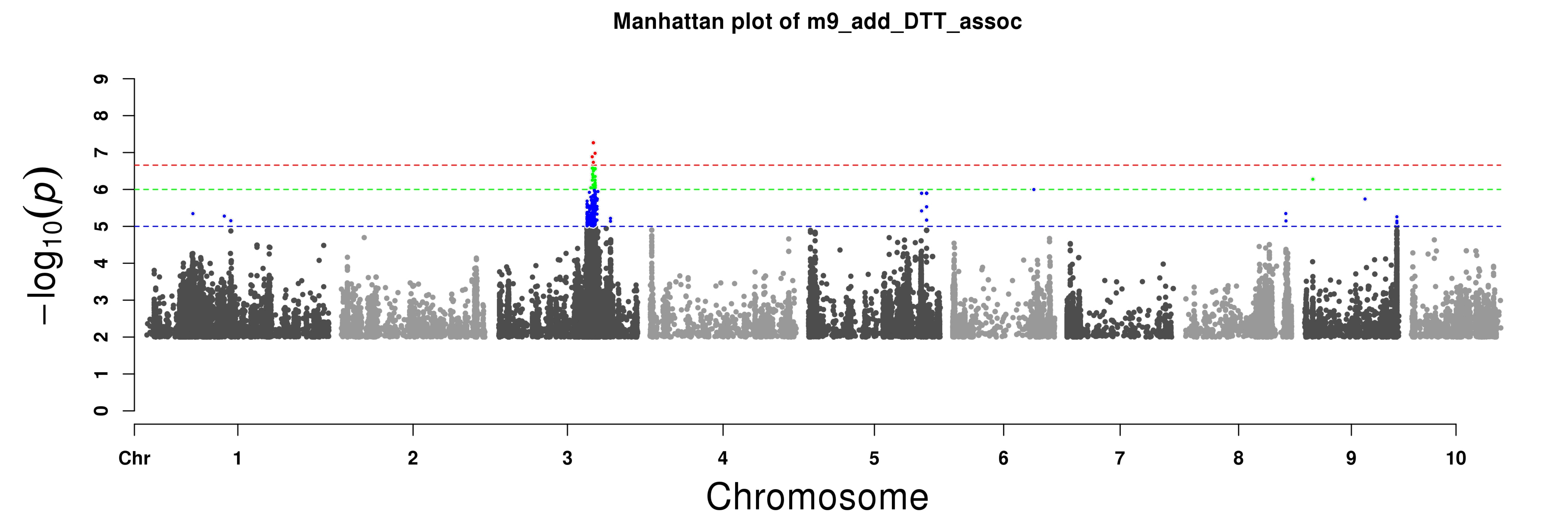

Manhattan plot of m9_add_DTT_assoc
$-\log_{10}(p)$
0
1
2
3
4
5
6
7
8
9
Chr
1
2
3
4
5
6
7
8
9
10
Chromosome

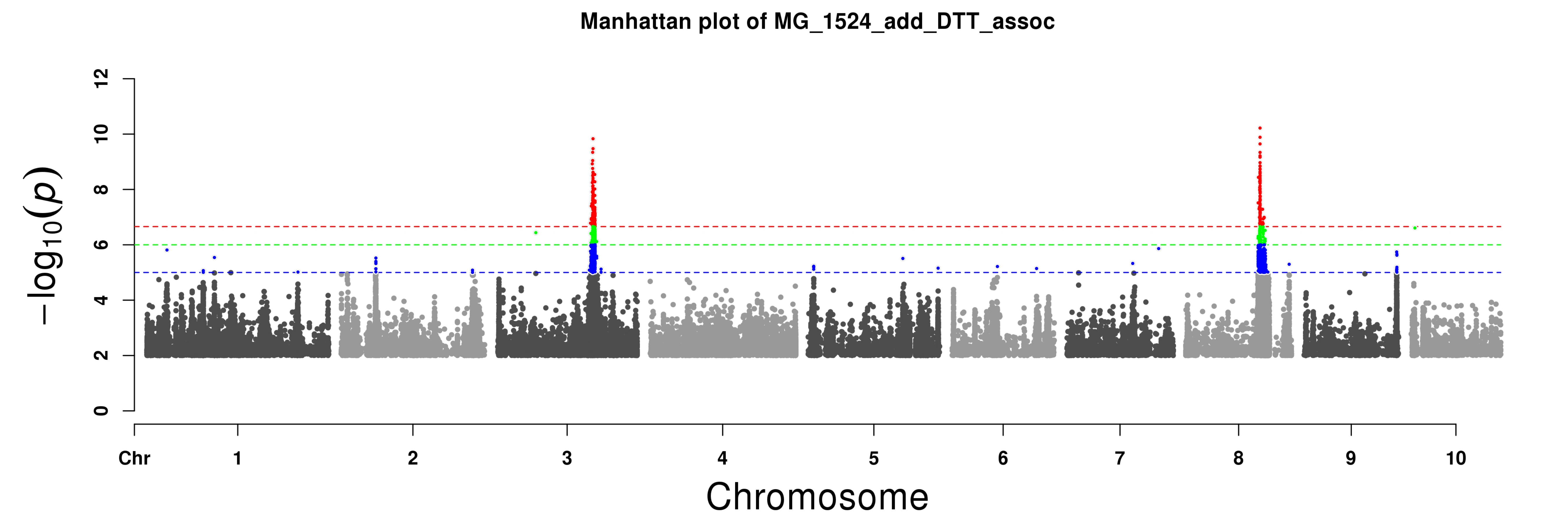
Manhattan plot of MG_1524_add_DTT_assoc
$-\log_{10}(p)$
0
2
4
6
8
10
12
Chr
1
2
3
4
5
6
7
8
9
10
Chromosome

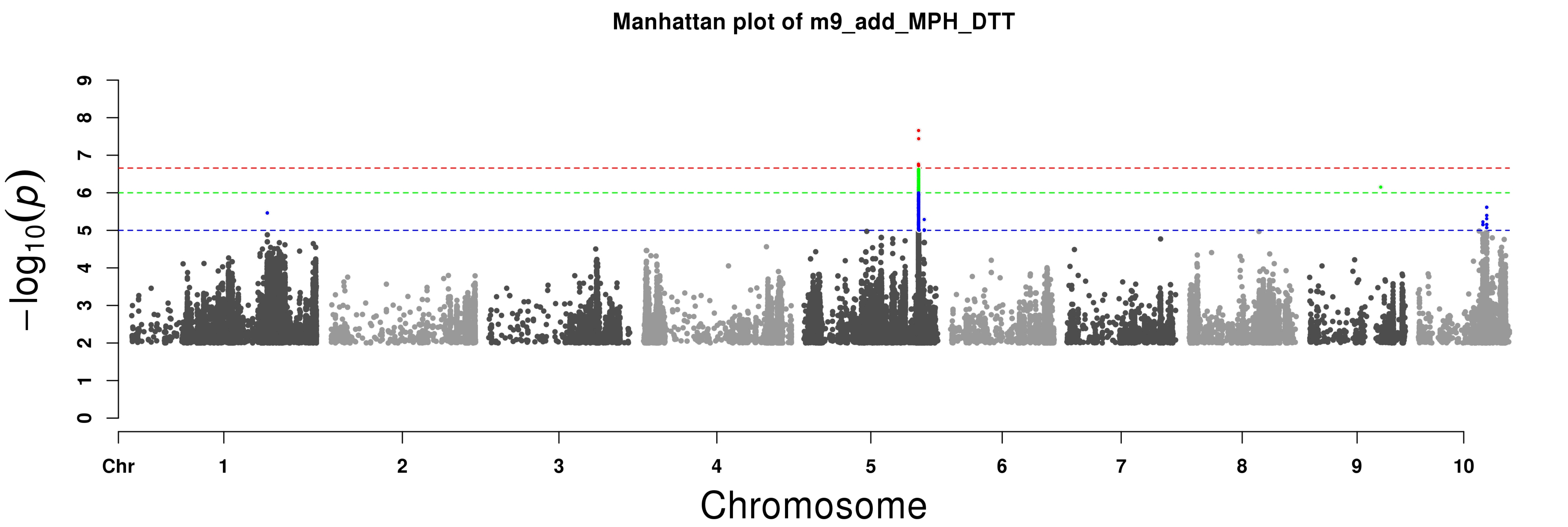

Manhattan plot of m9_add_MPH_DTT
$-\log_{10}(p)$
0
1
2
3
4
5
6
7
8
9
Chr
1
2
3
4
5
6
7
8
9
10
Chromosome

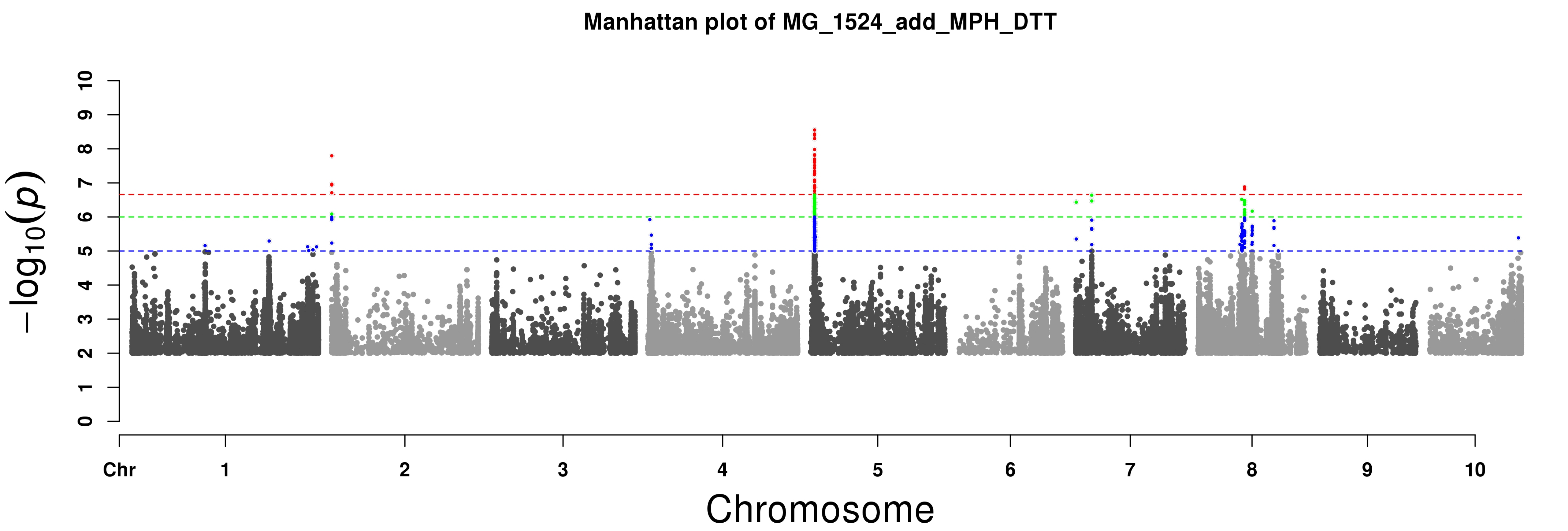

Manhattan plot of MG_1524_add_MPH_DTT
$-\log_{10}(p)$
0
1
2
3
4
5
6
7
8
9
10
Chr
1
2
3
4
5
6
7
8
9
10
Chromosome

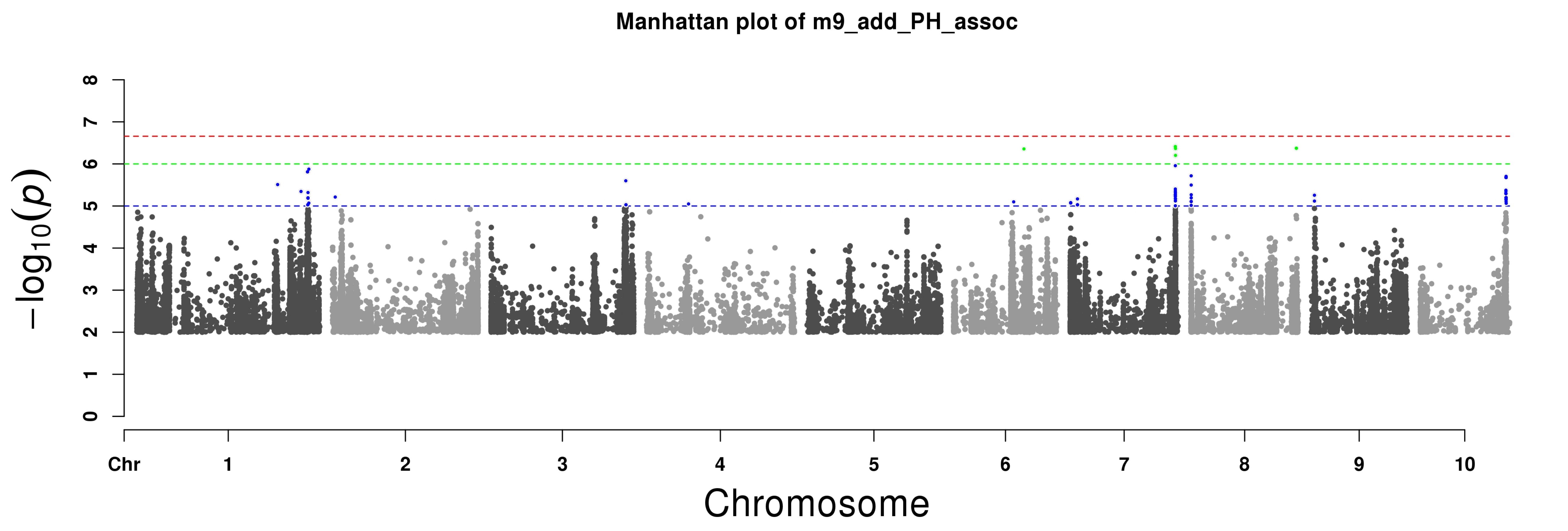

Manhattan plot of m9_add_PH_assoc
$-\log_{10}(p)$
0
1
2
3
4
5
6
7
8
Chr
1
2
3
4
5
6
7
8
9
10
Chromosome

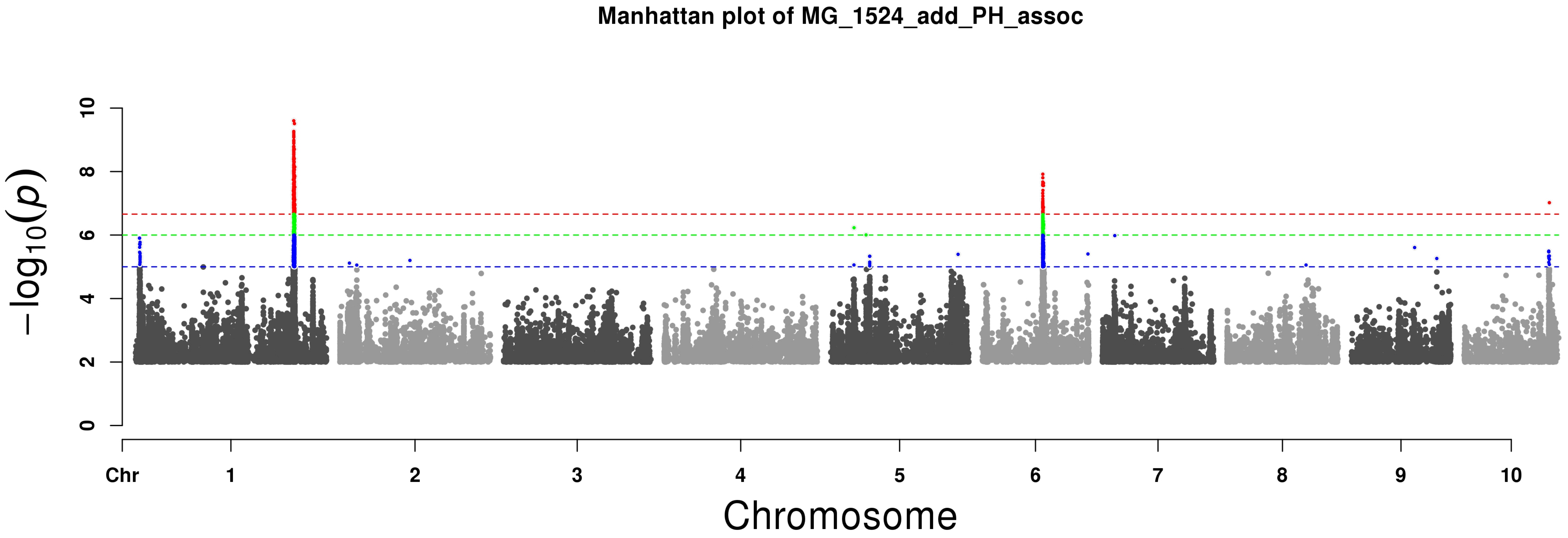
Manhattan plot of MG_1524_add_PH_assoc
$-\log_{10}(p)$
0
2
4
6
8
10
Chr
1
2
3
4
5
6
7
8
9
10
Chromosome

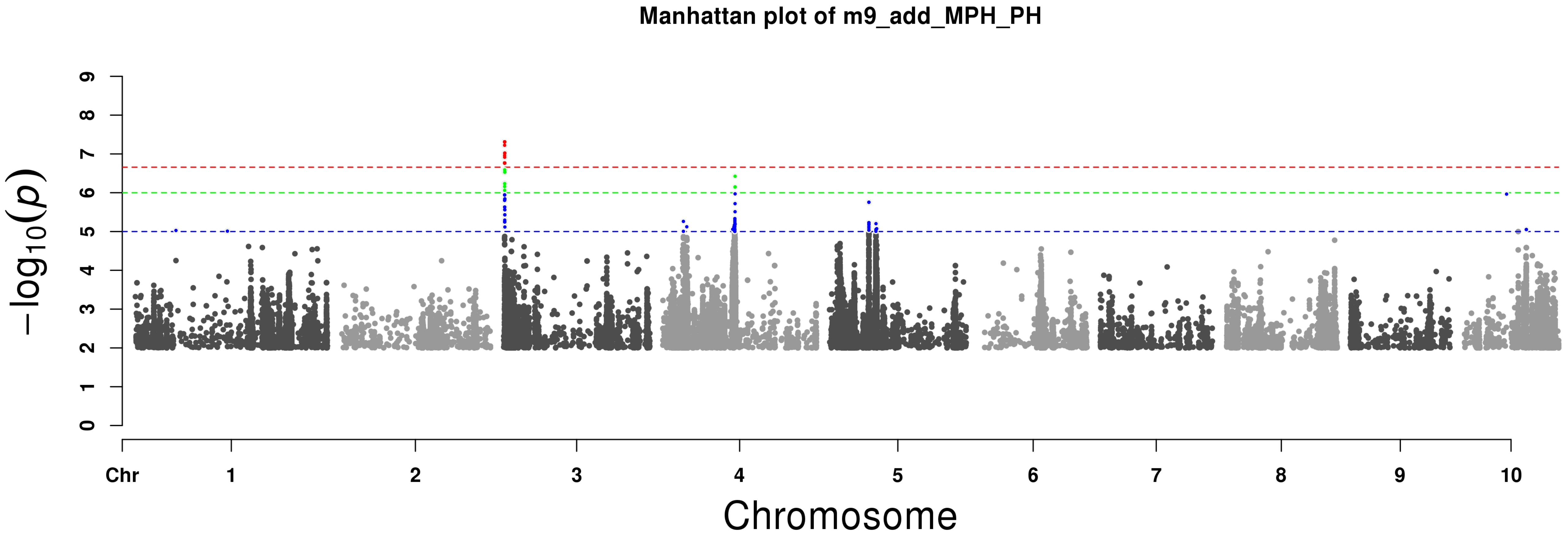

Manhattan plot of m9_add_MPH_PH
$-\log_{10}(p)$
0
1
2
3
4
5
6
7
8
9
Chr
1
2
3
4
5
6
7
8
9
10
Chromosome

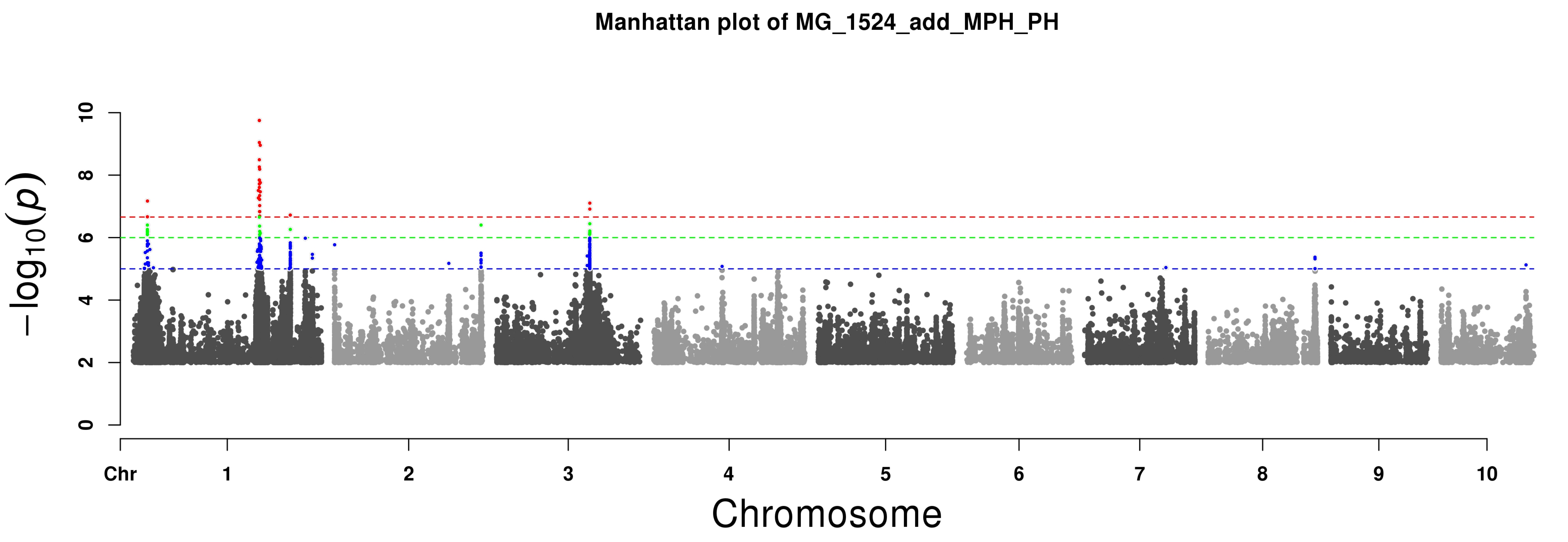
Manhattan plot of MG_1524_add_MPH_PH
$-\log_{10}(p)$
0
2
4
6
8
10
Chr
1
2
3
4
5
6
7
8
9
10
Chromosome

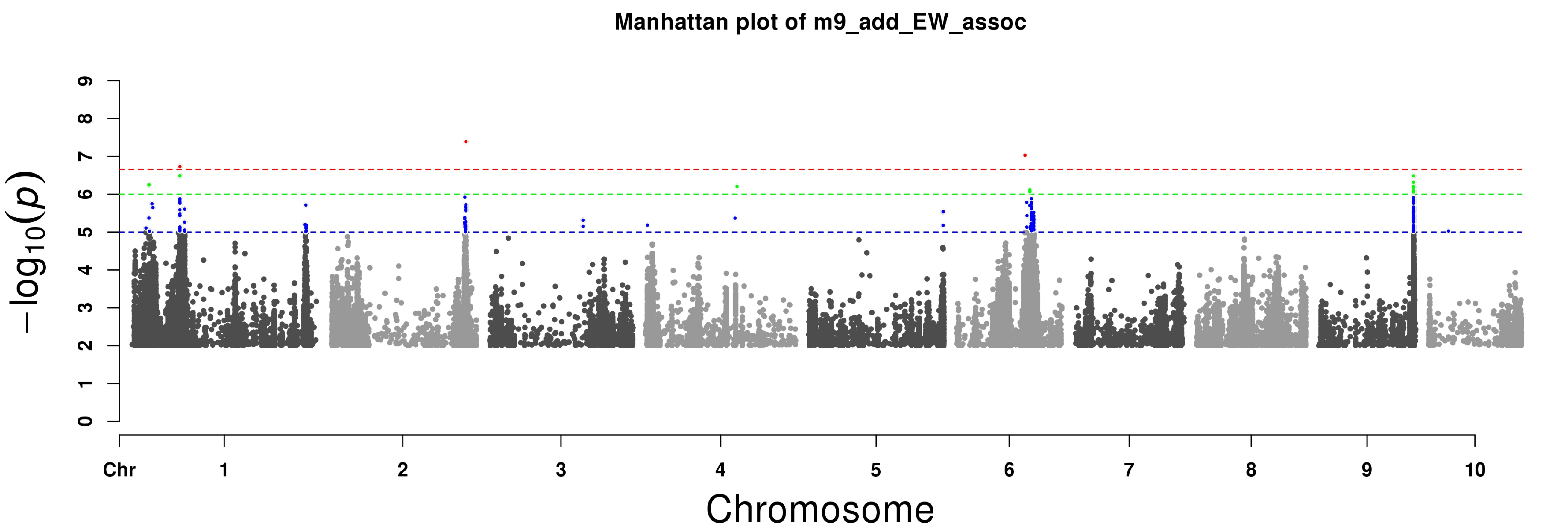

Manhattan plot of m9_add_EW_assoc
$-\log_{10}(p)$
0
1
2
3
4
5
6
7
8
9
Chr
1
2
3
4
5
6
7
8
9
10
Chromosome

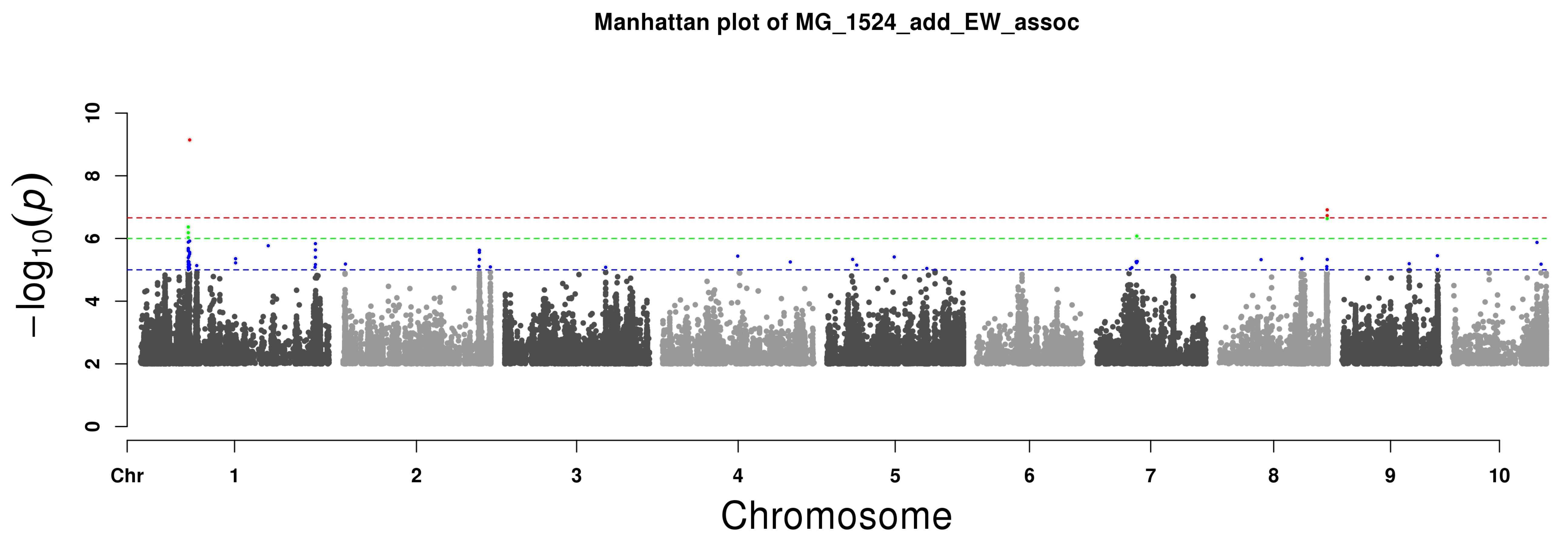

Manhattan plot of MG_1524_add_EW_assoc
$-\log_{10}(p)$
0
2
4
6
8
10
Chr
1
2
3
4
5
6
7
8
9
10
Chromosome

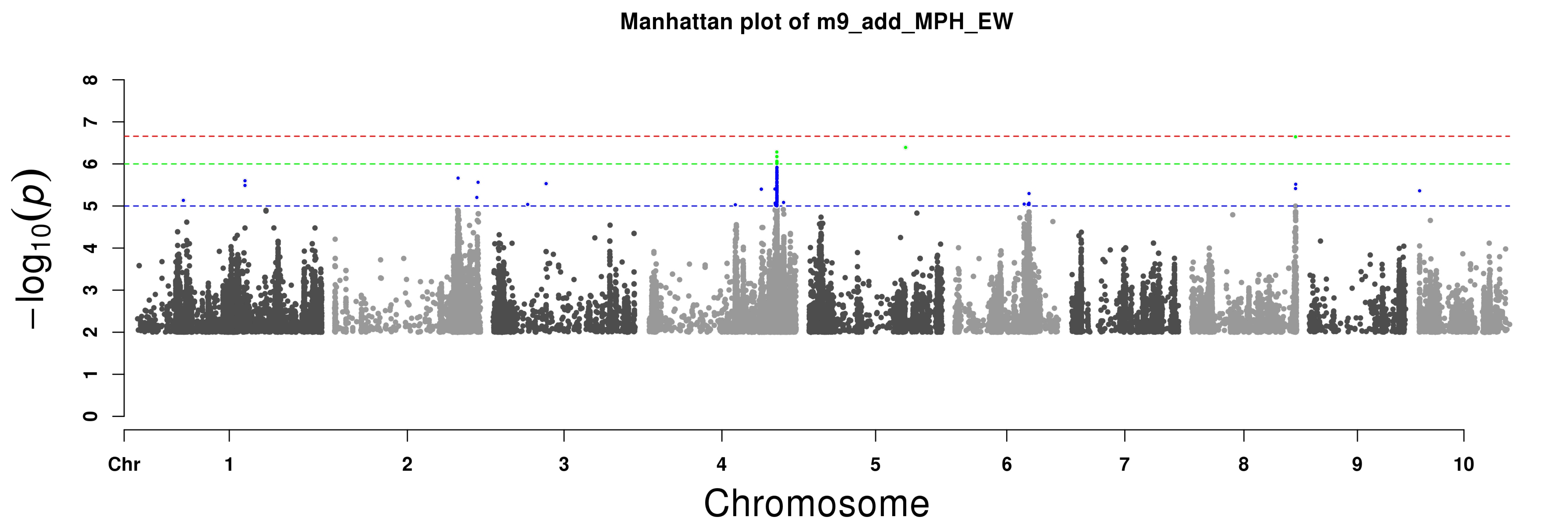

Manhattan plot of m9_add_MPH_EW
$-\log_{10}(p)$
0
1
2
3
4
5
6
7
8
Chr
1
2
3
4
5
6
7
8
9
10
Chromosome

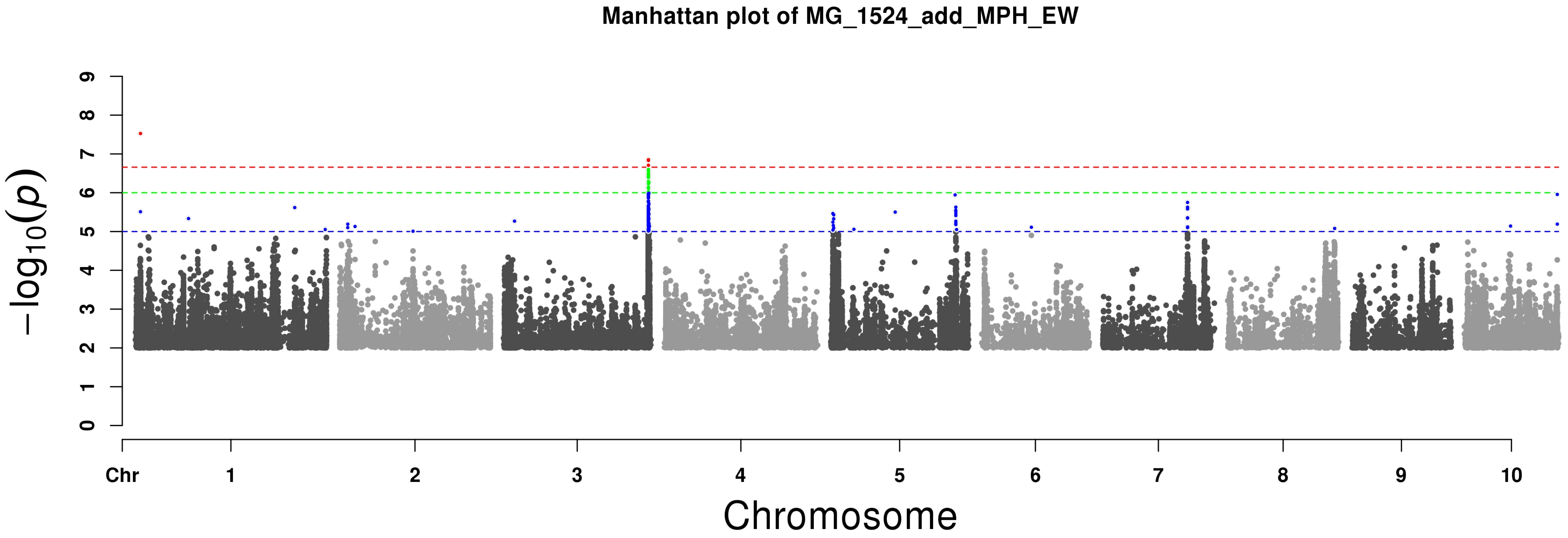

Manhattan plot of MG_1524_add_MPH_EW
$-\log_{10}(p)$
0
1
2
3
4
5
6
7
8
9
Chr
1
2
3
4
5
6
7
8
9
10
Chromosome

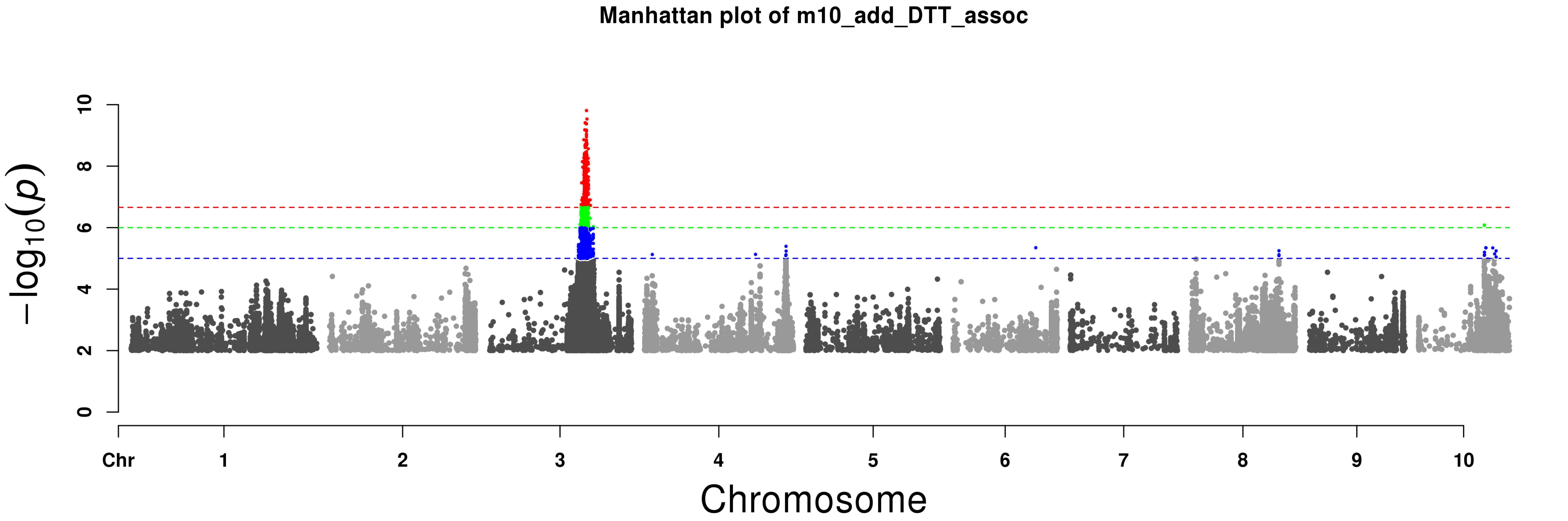

Manhattan plot of m10_add_DTT_assoc
$-\log_{10}(p)$
0
2
4
6
8
10
Chr
1
2
3
4
5
6
7
8
9
10
Chromosome

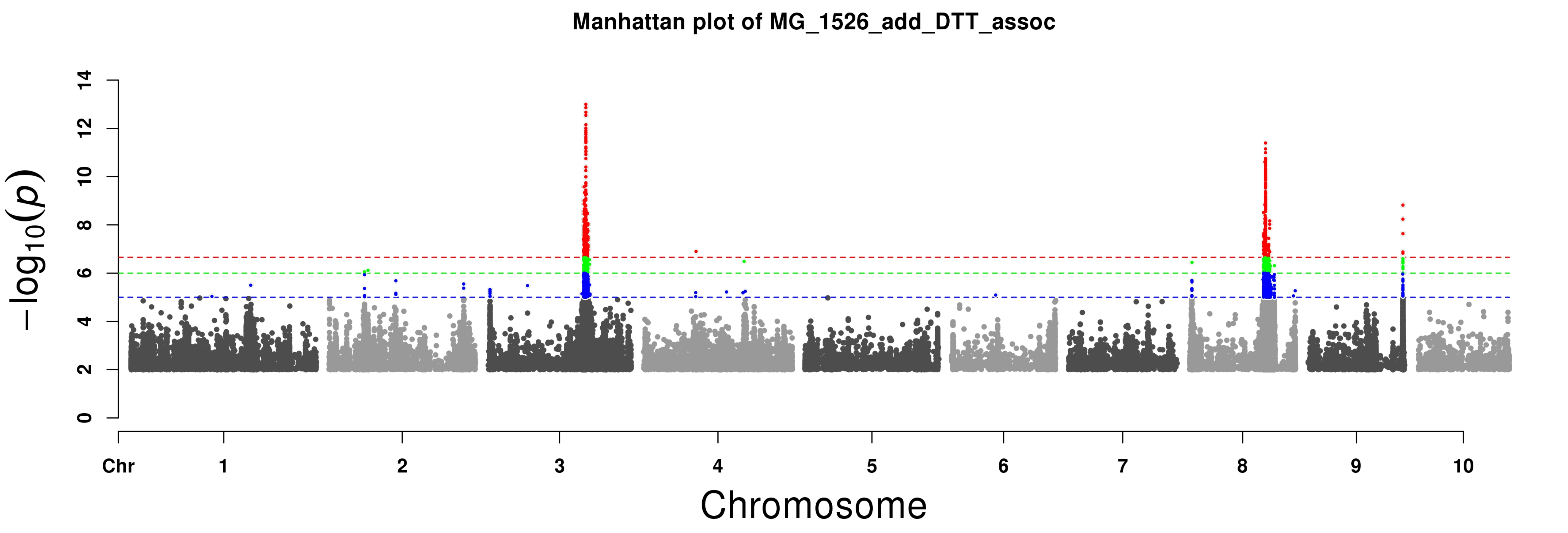
Manhattan plot of MG_1526_add_DTT_assoc
−log10(p)
0
2
4
6
8
10
12
14
Chr
1
2
3
4
5
6
7
8
9
10
Chromosome

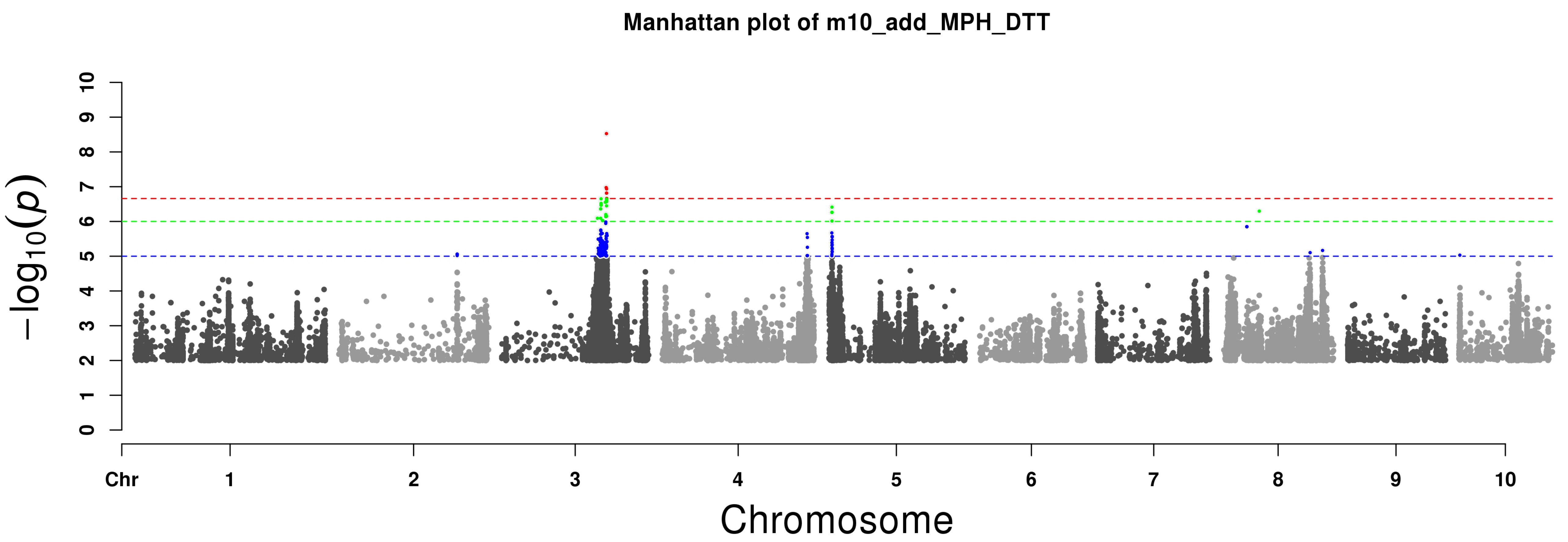

Manhattan plot of m10_add_MPH_DTT
$-\log_{10}(p)$
0
1
2
3
4
5
6
7
8
9
10
Chr
1
2
3
4
5
6
7
8
9
10
Chromosome

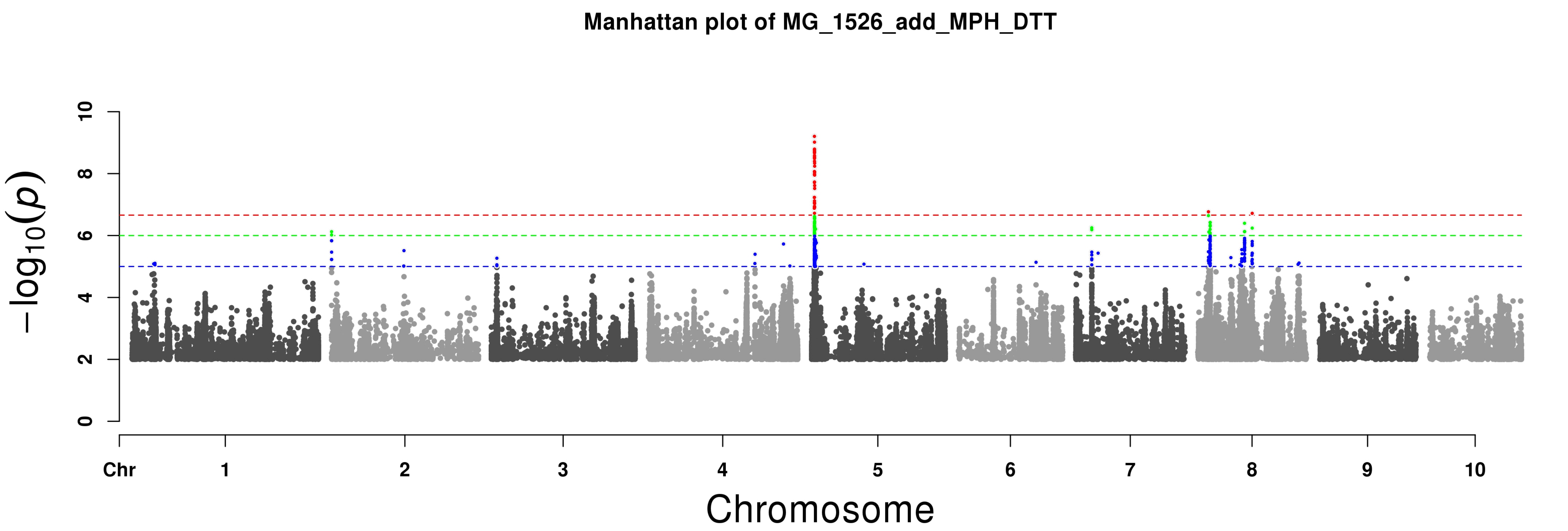

Manhattan plot of MG_1526_add_MPH_DTT
$-\log_{10}(p)$
0
2
4
6
8
10
Chr
1
2
3
4
5
6
7
8
9
10
Chromosome

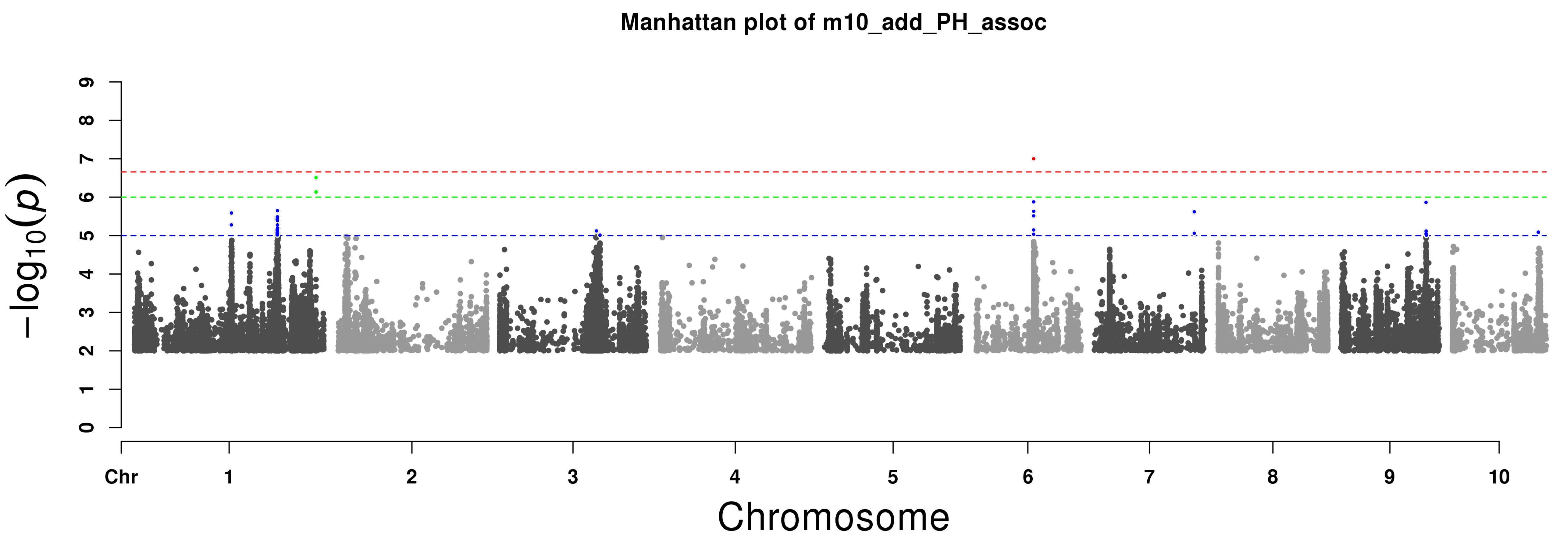

Manhattan plot of m10_add_PH_assoc
$-\log_{10}(p)$
0
1
2
3
4
5
6
7
8
9
Chr
1
2
3
4
5
6
7
8
9
10
Chromosome

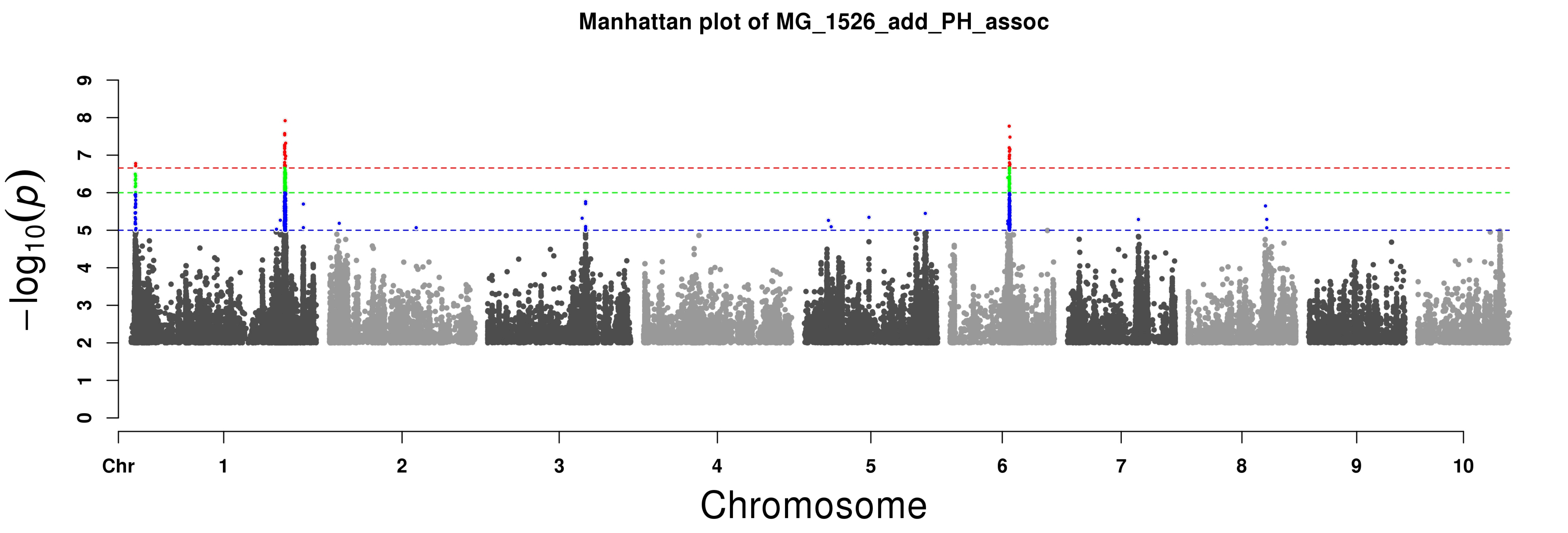

Manhattan plot of MG_1526_add_PH_assoc
$-\log_{10}(p)$
0
1
2
3
4
5
6
7
8
9
Chr
1
2
3
4
5
6
7
8
9
10
Chromosome

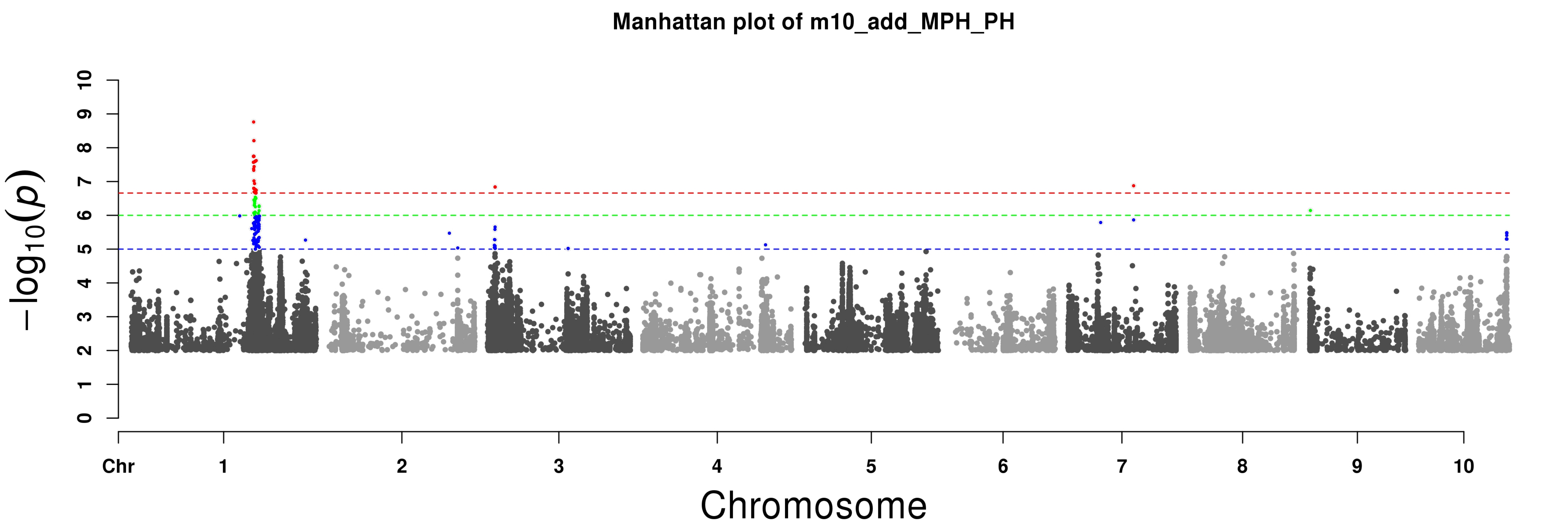

Manhattan plot of m10_add_MPH_PH
$-\log_{10}(p)$
0
1
2
3
4
5
6
7
8
9
10
Chr
1
2
3
4
5
6
7
8
9
10
Chromosome

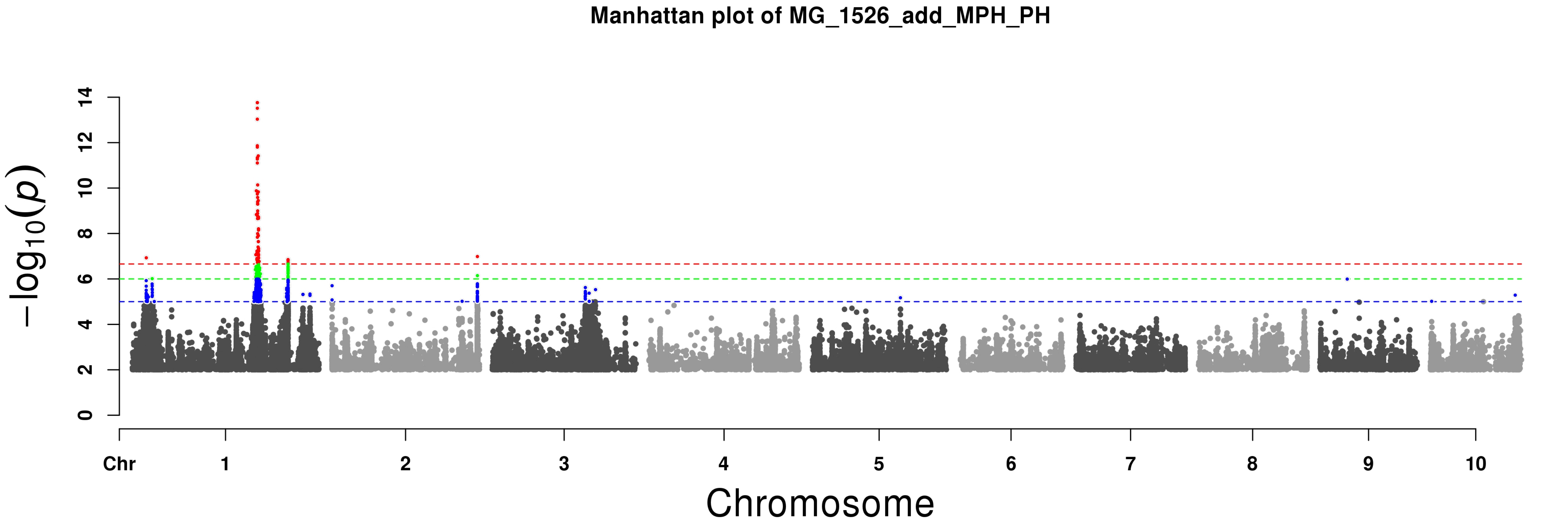

Manhattan plot of MG_1526_add_MPH_PH
14
12
10
8
6
4
2
0
$-\log_{10}(p)$
Chr
1
2
3
4
5
6
7
8
9
10
Chromosome

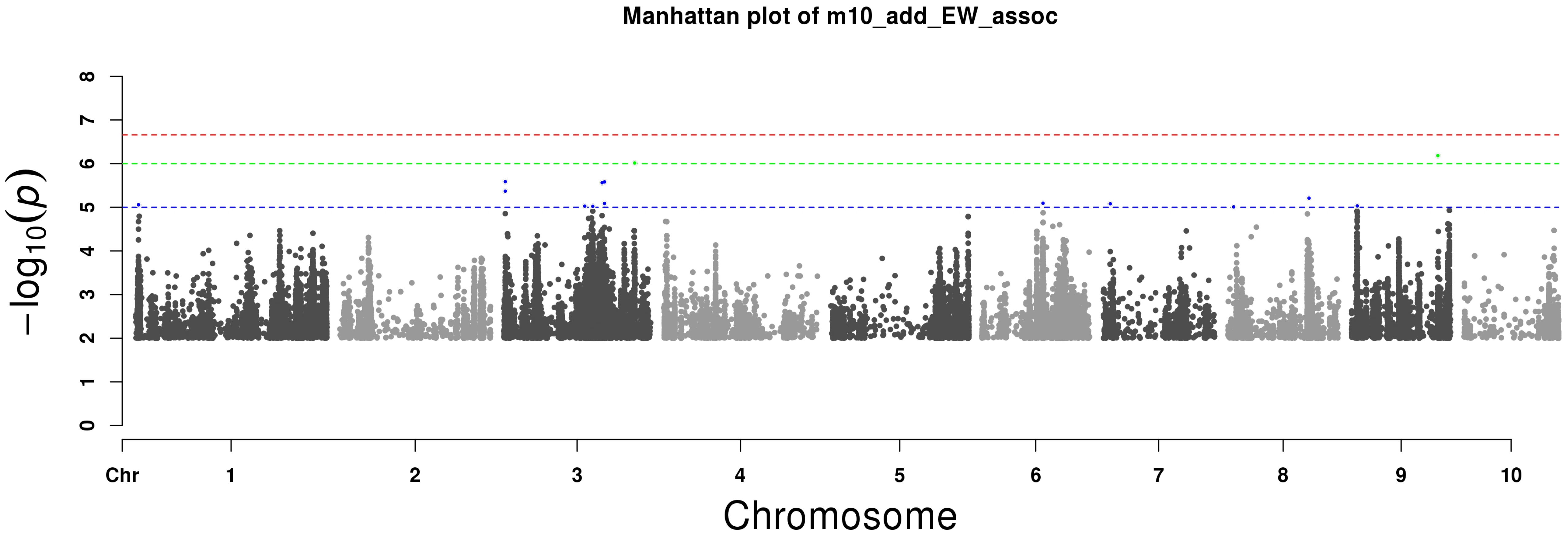
Manhattan plot of m10_add_EW_assoc
$-\log_{10}(p)$
0
1
2
3
4
5
6
7
8
Chr
1
2
3
4
5
6
7
8
9
10
Chromosome

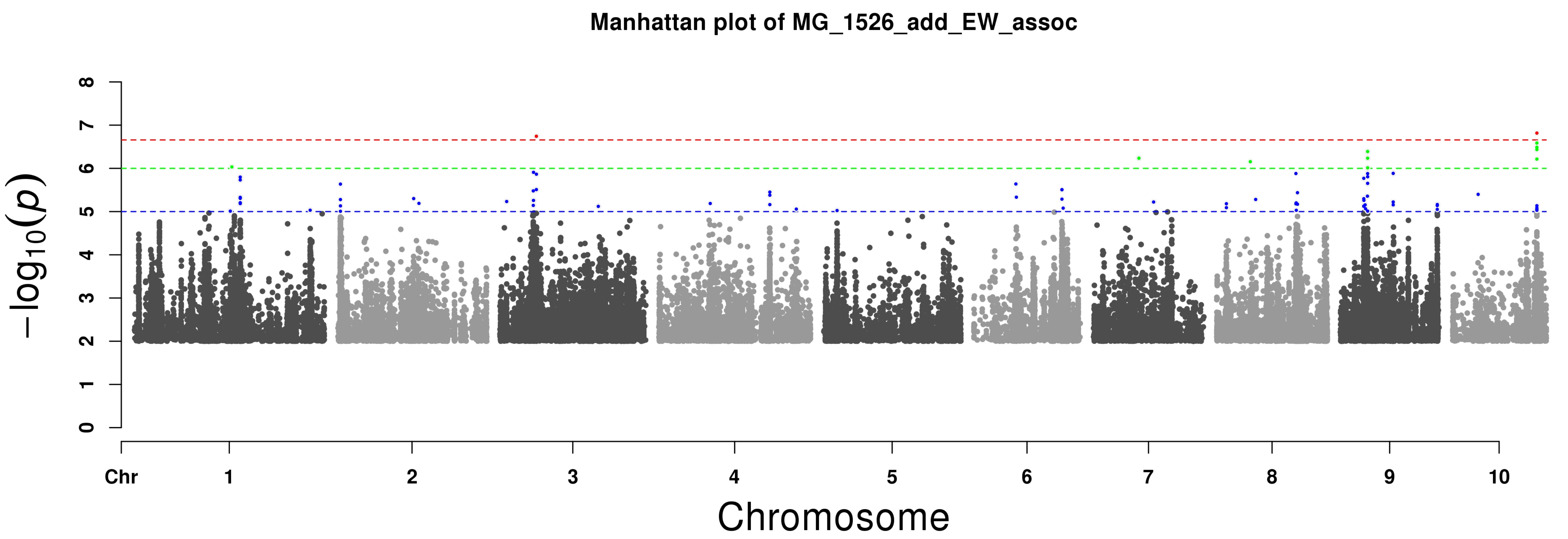
Manhattan plot of MG_1526_add_EW_assoc
$-\log_{10}(p)$
0
1
2
3
4
5
6
7
8
Chr
1
2
3
4
5
6
7
8
9
10
Chromosome

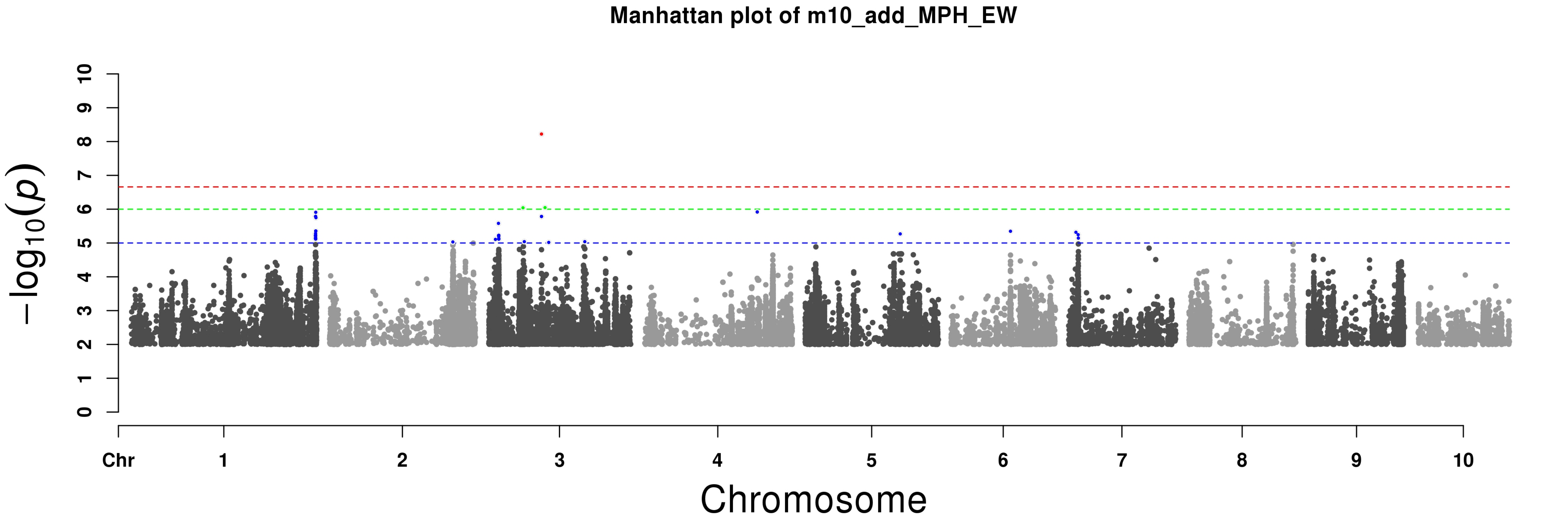

Manhattan plot of m10_add_MPH_EW
$-\log_{10}(p)$
0
1
2
3
4
5
6
7
8
9
10
Chr
1
2
3
4
5
6
7
8
9
10
Chromosome

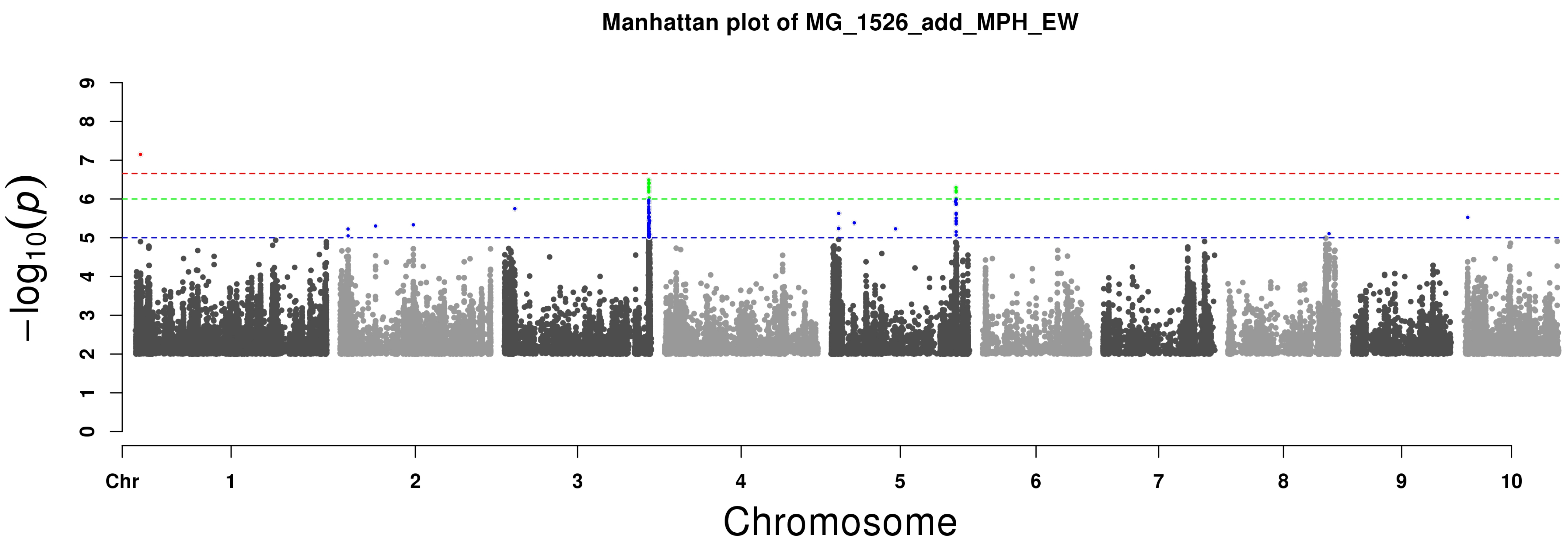

Manhattan plot of MG_1526_add_MPH_EW
−log10(p)
0
1
2
3
4
5
6
7
8
9
Chr
1
2
3
4
5
6
7
8
9
10
Chromosome

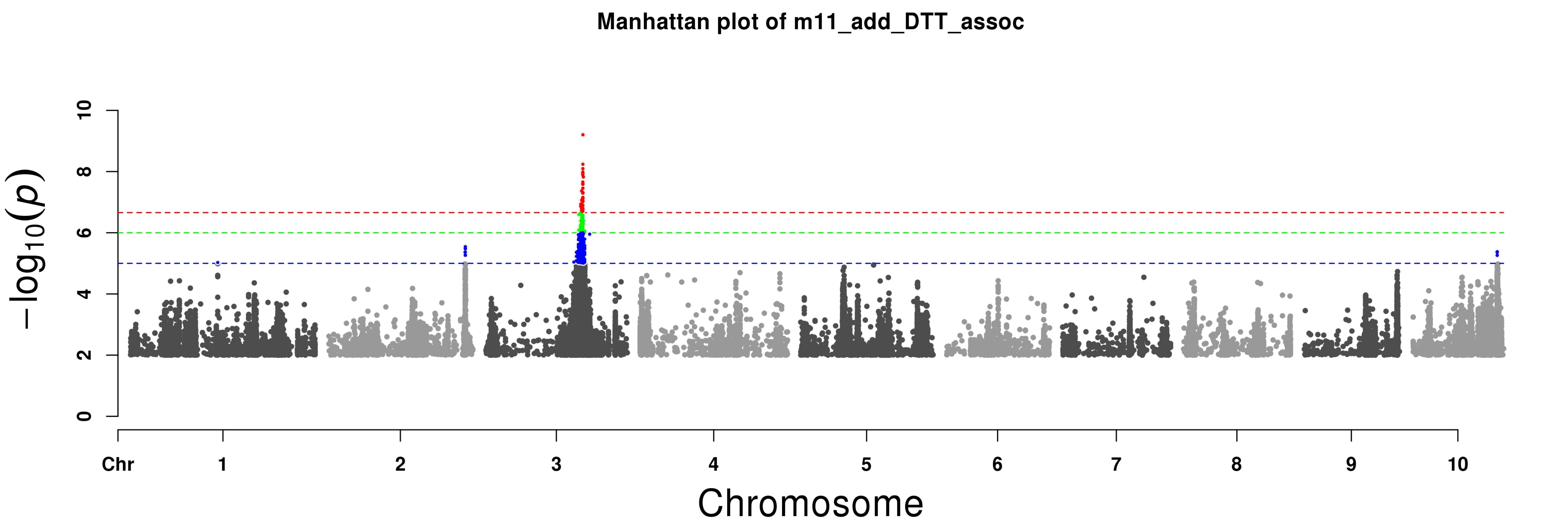

Manhattan plot of m11_add_DTT_assoc
$-\log_{10}(p)$
0
2
4
6
8
10
Chr
1
2
3
4
5
6
7
8
9
10
Chromosome

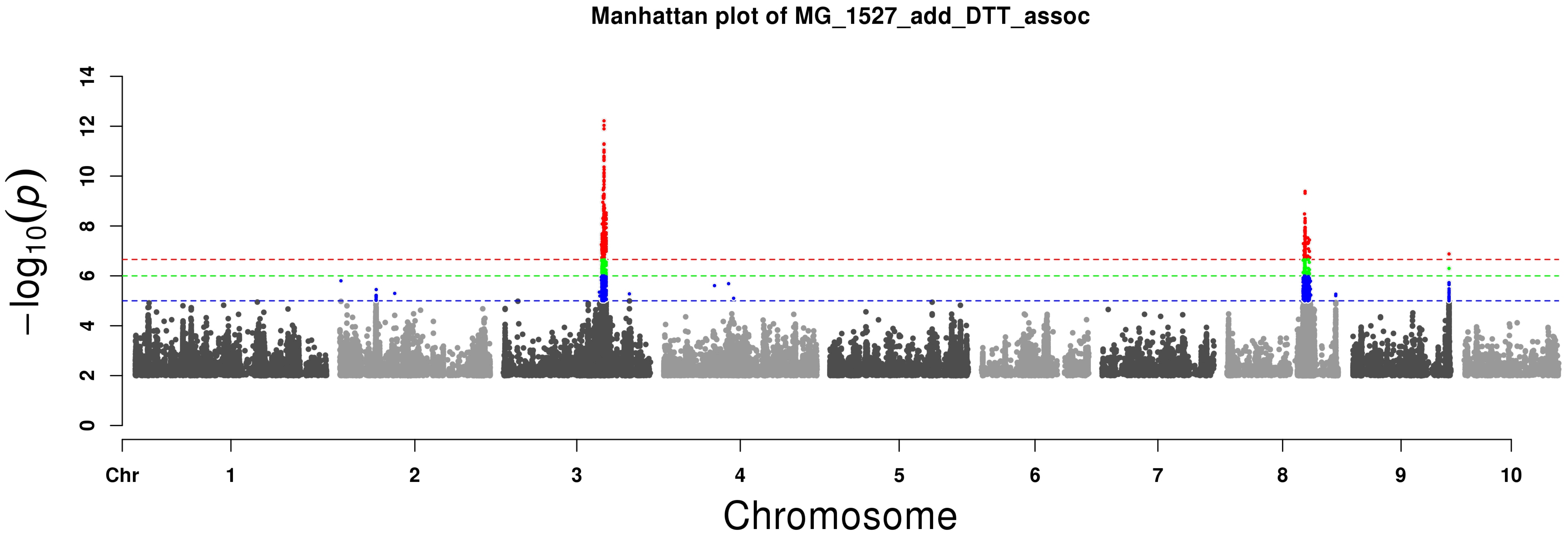
Manhattan plot of MG_1527_add_DTT_assoc
0
2
4
6
8
10
12
14
$-\log_{10}(p)$
Chr
1
2
3
4
5
6
7
8
9
10
Chromosome

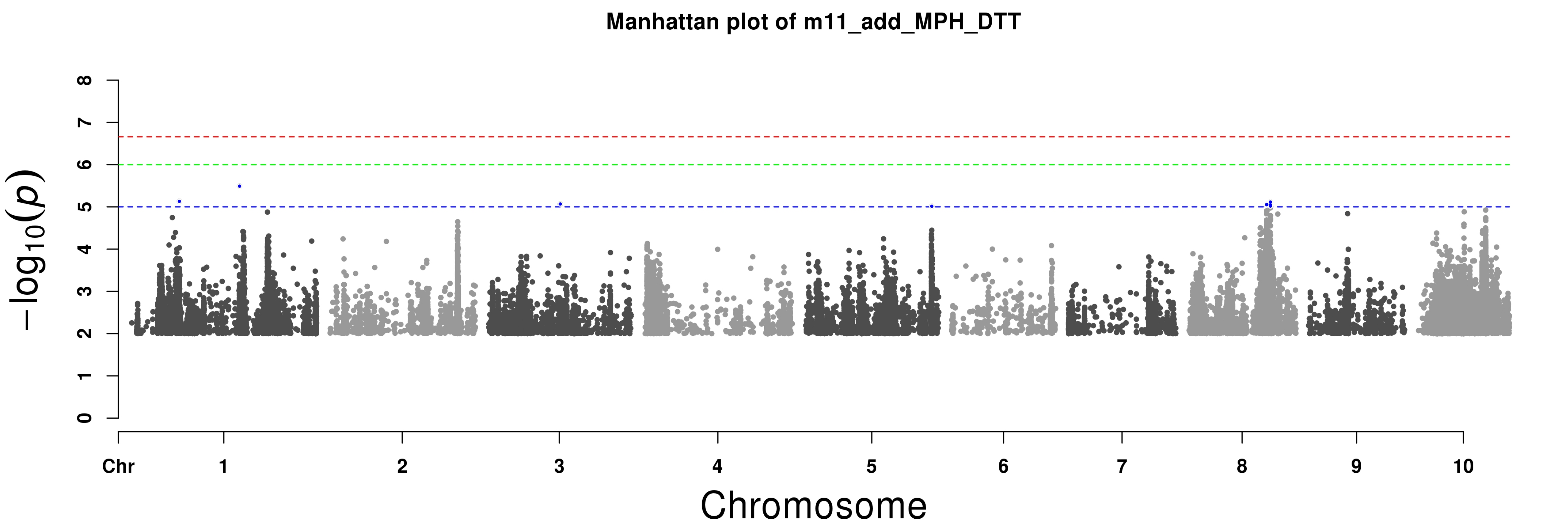
Manhattan plot of m11_add_MPH_DTT
$-\log_{10}(p)$
0
1
2
3
4
5
6
7
8
Chr
1
2
3
4
5
6
7
8
9
10
Chromosome

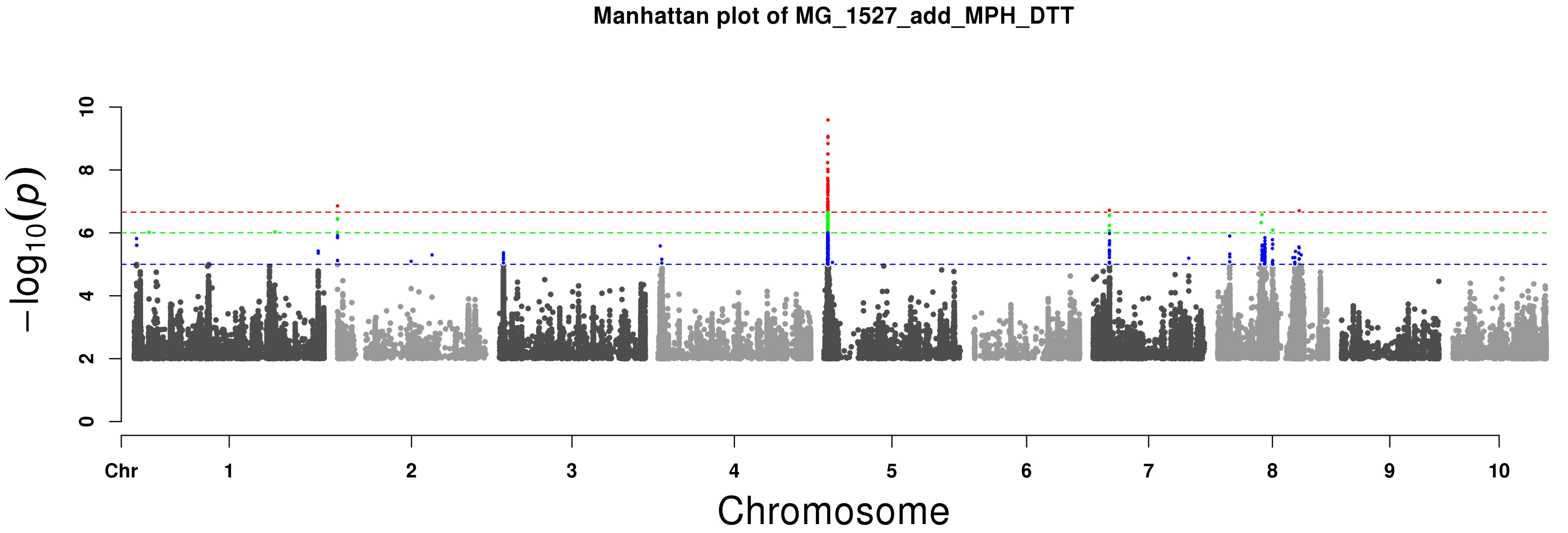
Manhattan plot of MG_1527_add_MPH_DTT
$-\log_{10}(p)$
0
2
4
6
8
10
Chr
1
2
3
4
5
6
7
8
9
10
Chromosome

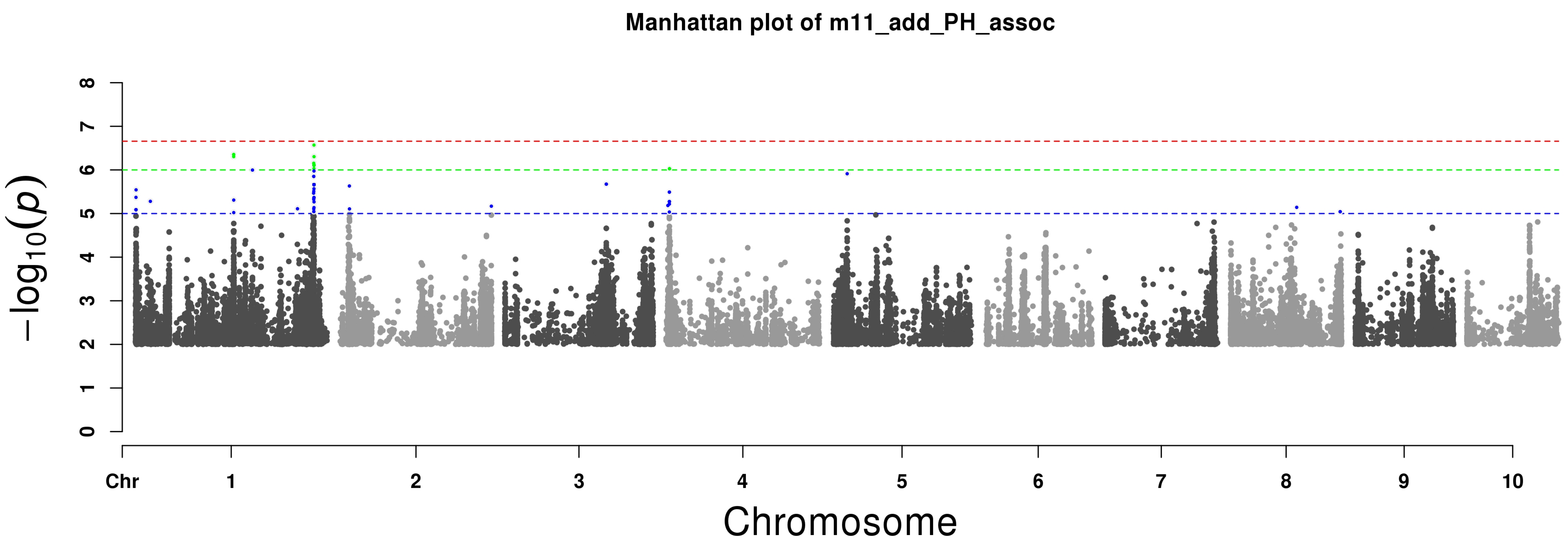

Manhattan plot of m11_add_PH_assoc
0
1
2
3
4
5
6
7
8
$-\log_{10}(p)$
Chr
1
2
3
4
5
6
7
8
9
10
Chromosome

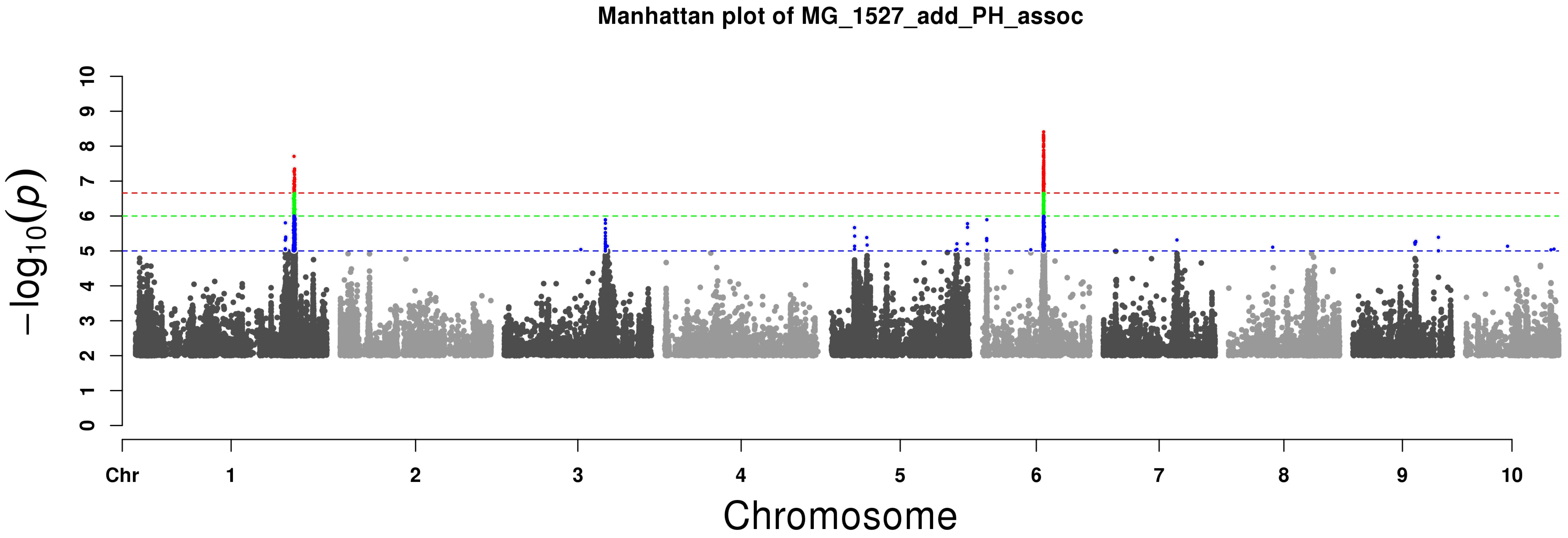

Manhattan plot of MG_1527_add_PH_assoc
$-\log_{10}(p)$
0
1
2
3
4
5
6
7
8
9
10
Chr
1
2
3
4
5
6
7
8
9
10
Chromosome

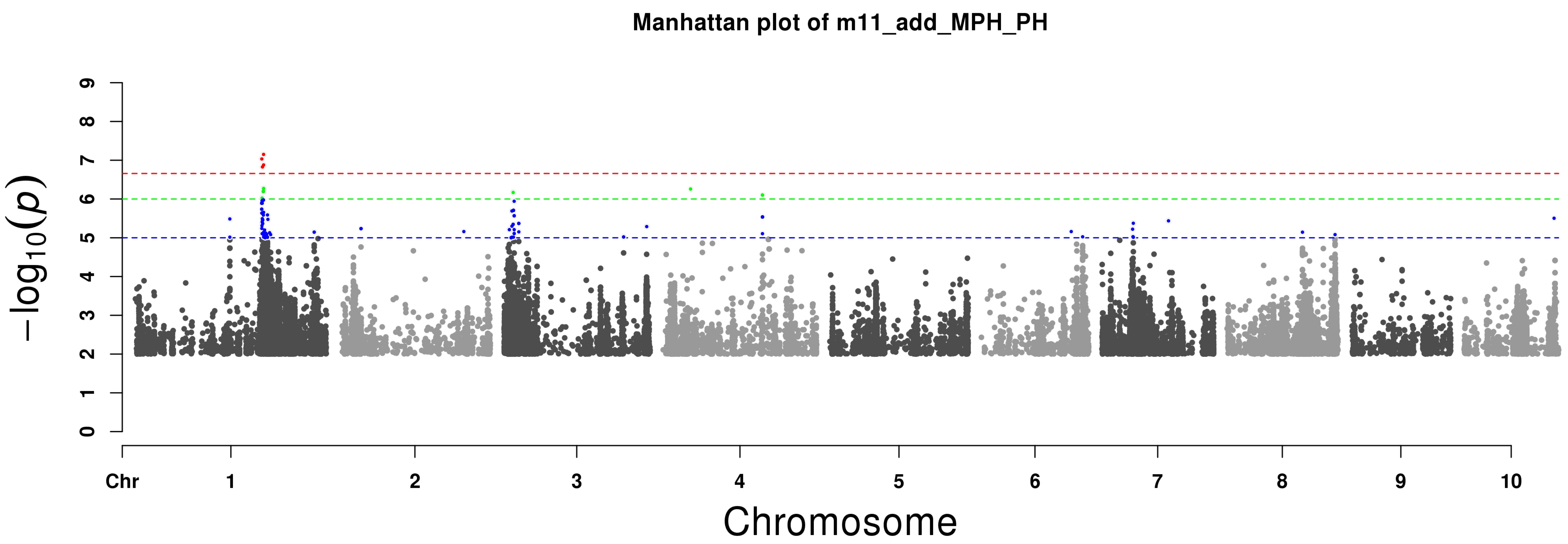

Manhattan plot of m11_add_MPH_PH
$-\log_{10}(p)$
0
1
2
3
4
5
6
7
8
9
Chr
1
2
3
4
5
6
7
8
9
10
Chromosome

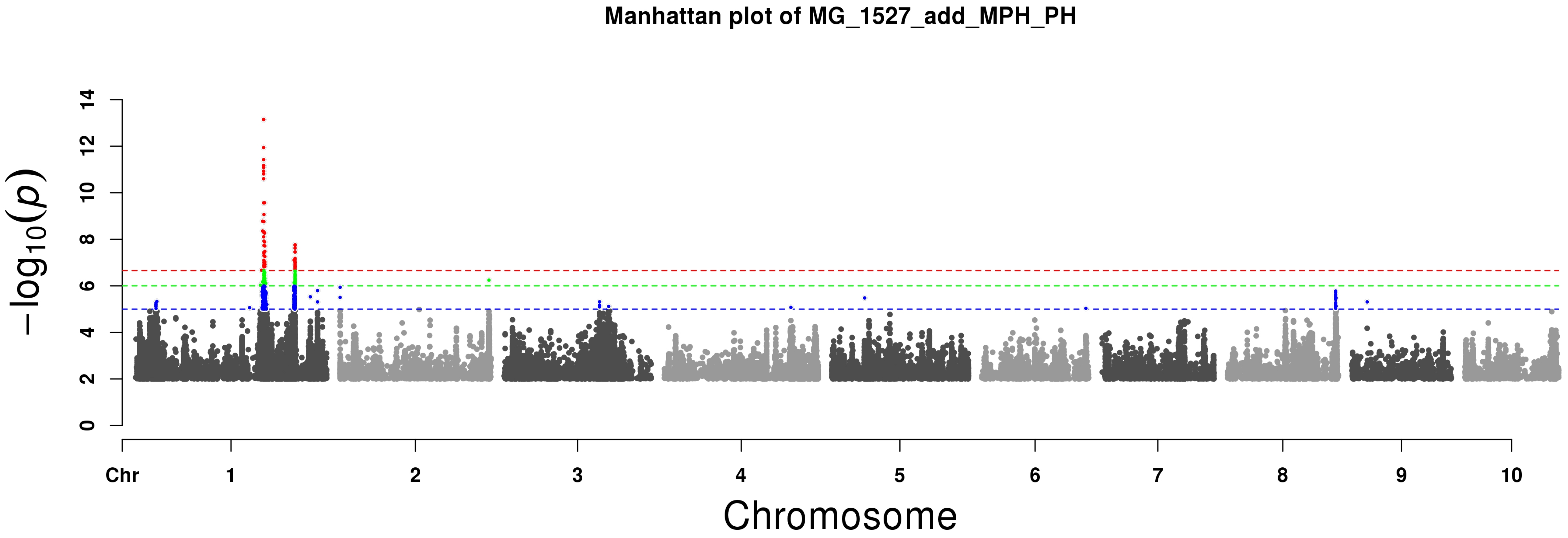

Manhattan plot of MG_1527_add_MPH_PH
$-\log_{10}(p)$
0
2
4
6
8
10
12
14
Chr
1
2
3
4
5
6
7
8
9
10
Chromosome

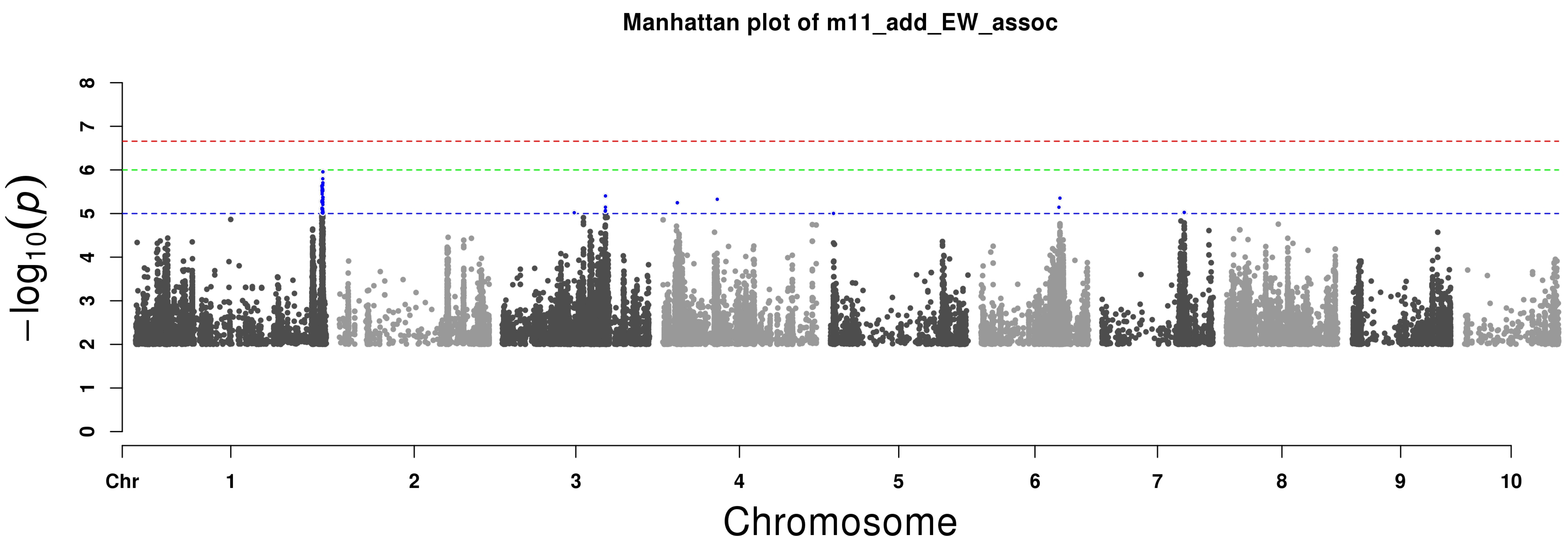

Manhattan plot of m11_add_EW_assoc
$-\log_{10}(p)$
0
1
2
3
4
5
6
7
8
Chr
1
2
3
4
5
6
7
8
9
10
Chromosome

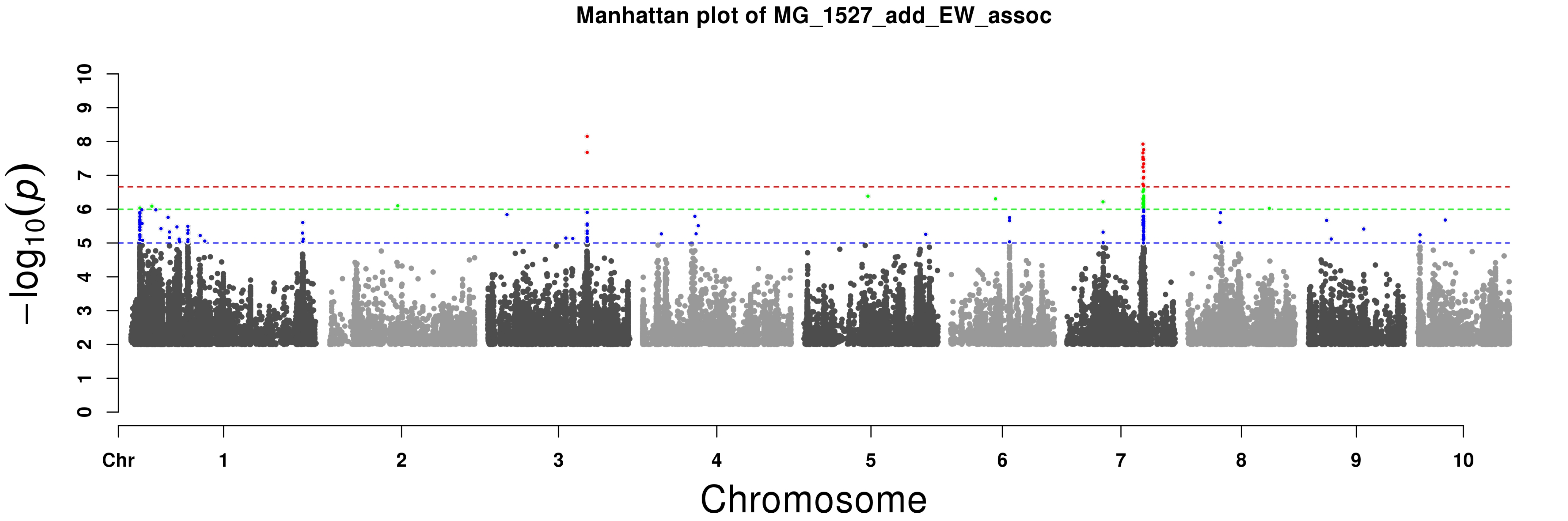
Manhattan plot of MG_1527_add_EW_assoc
$-\log_{10}(p)$
0
1
2
3
4
5
6
7
8
9
10
Chr
1
2
3
4
5
6
7
8
9
10
Chromosome

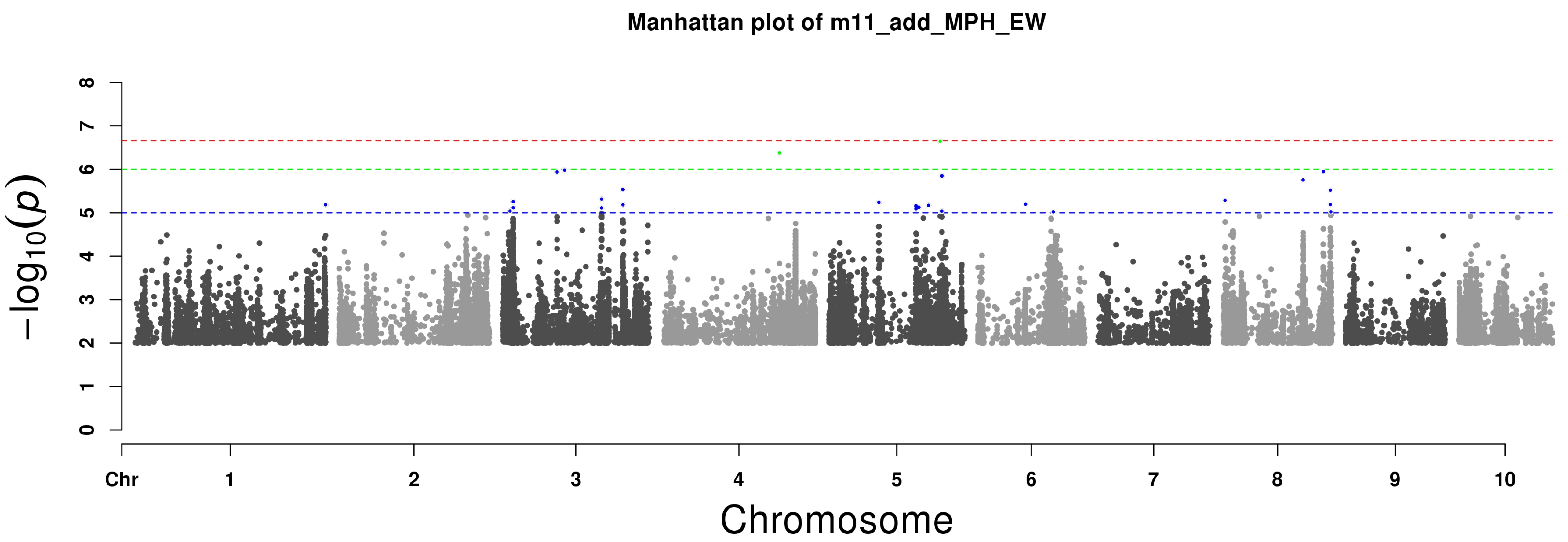

Manhattan plot of m11_add_MPH_EW
$-\log_{10}(p)$
0
1
2
3
4
5
6
7
8
Chr
1
2
3
4
5
6
7
8
9
10
Chromosome

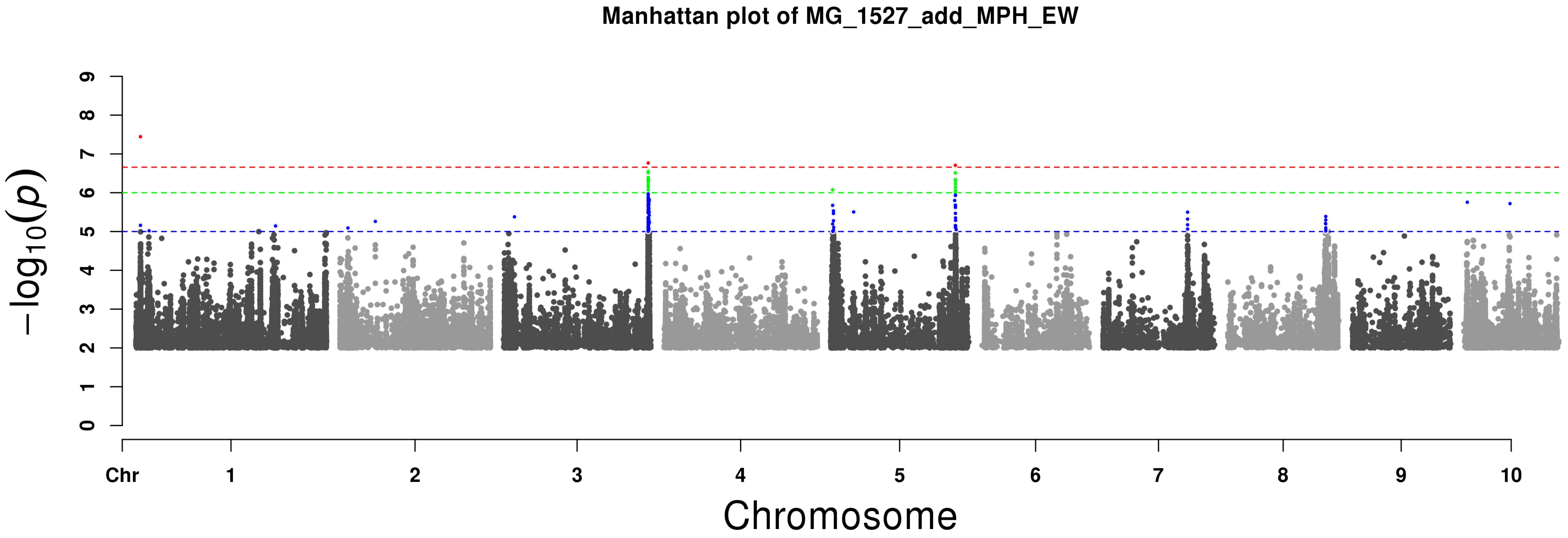

Manhattan plot of MG_1527_add_MPH_EW
$-\log_{10}(p)$
0
1
2
3
4
5
6
7
8
9
Chr
1
2
3
4
5
6
7
8
9
10
Chromosome

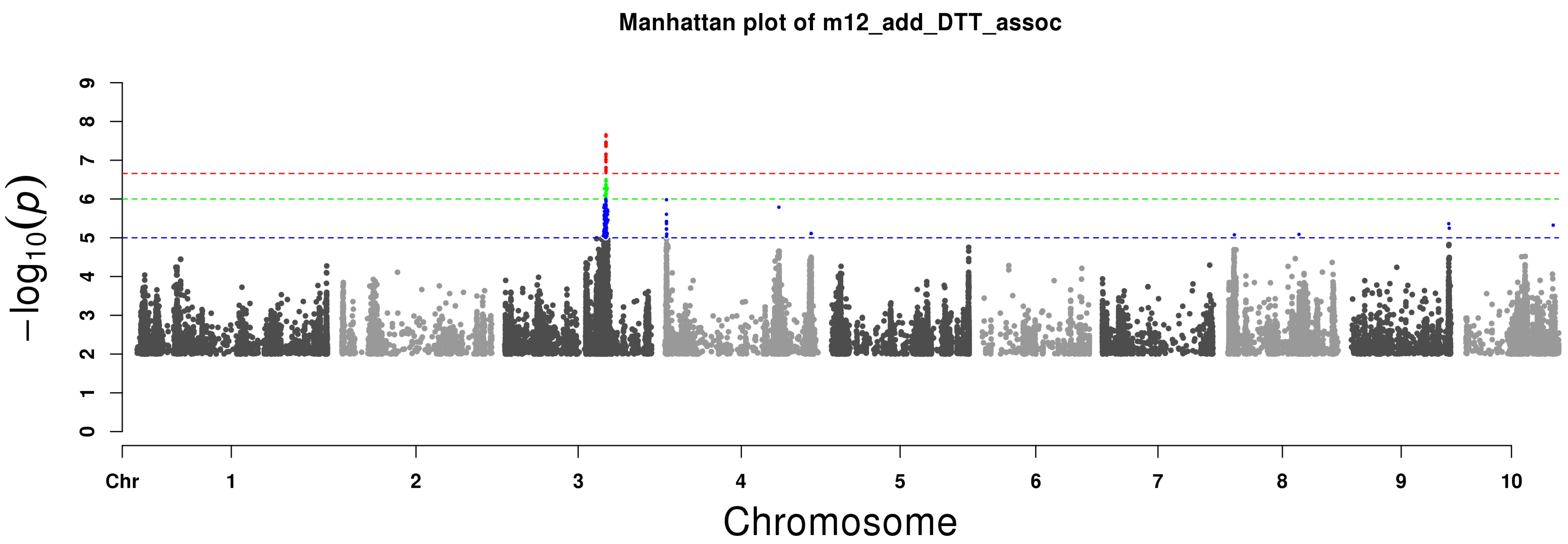

Manhattan plot of m12_add_DTT_assoc
$-\log_{10}(p)$
0
1
2
3
4
5
6
7
8
9
Chr
1
2
3
4
5
6
7
8
9
10
Chromosome

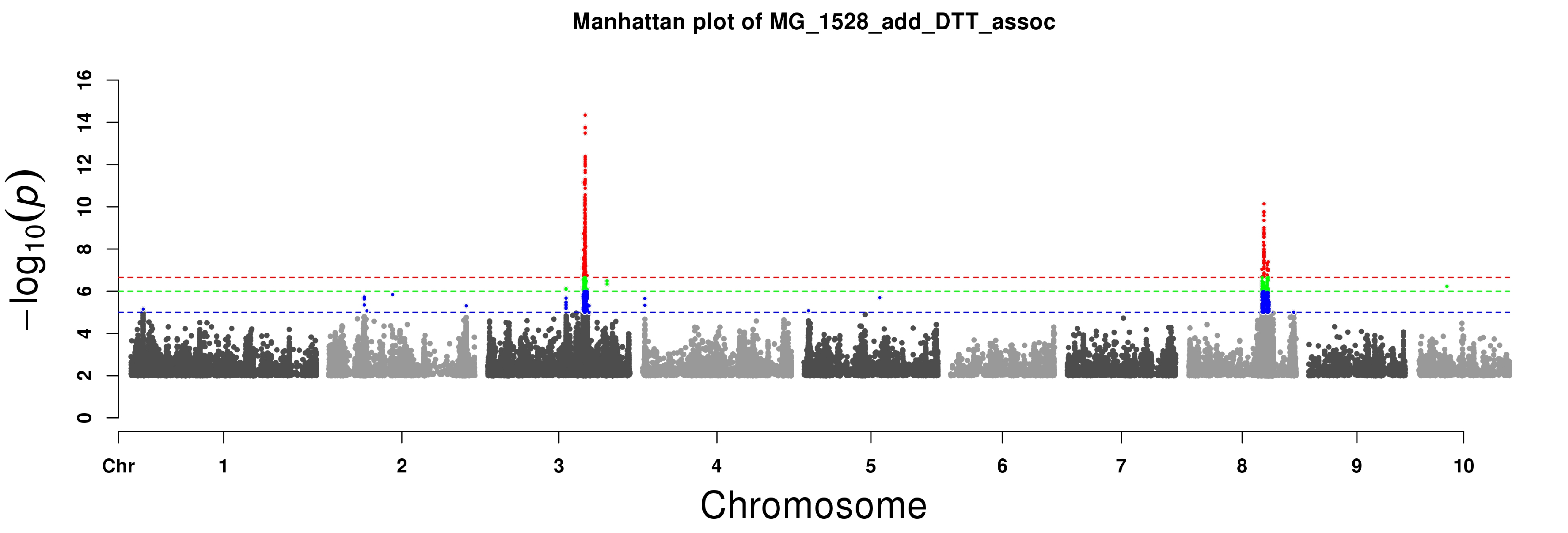

Manhattan plot of MG_1528_add_DTT_assoc
$-\log_{10}(p)$
0
2
4
6
8
10
12
14
16
Chr
1
2
3
4
5
6
7
8
9
10
Chromosome

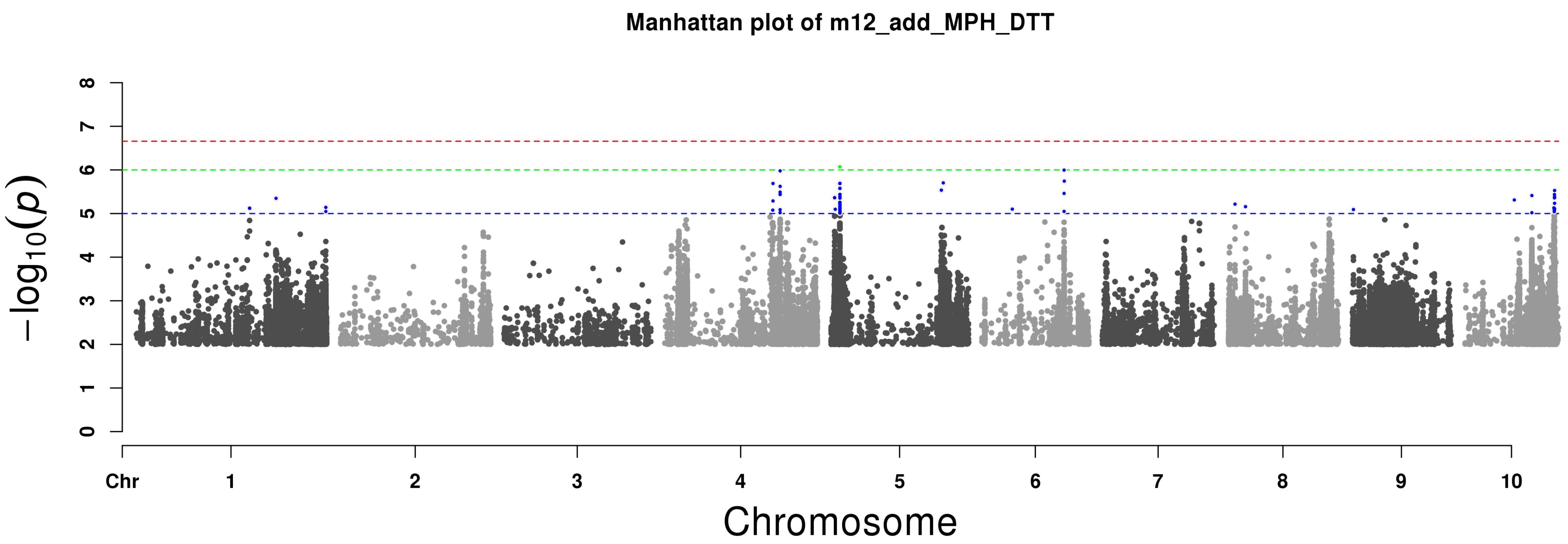

Manhattan plot of m12_add_MPH_DTT
$-\log_{10}(p)$
0
1
2
3
4
5
6
7
8
Chr
1
2
3
4
5
6
7
8
9
10
Chromosome

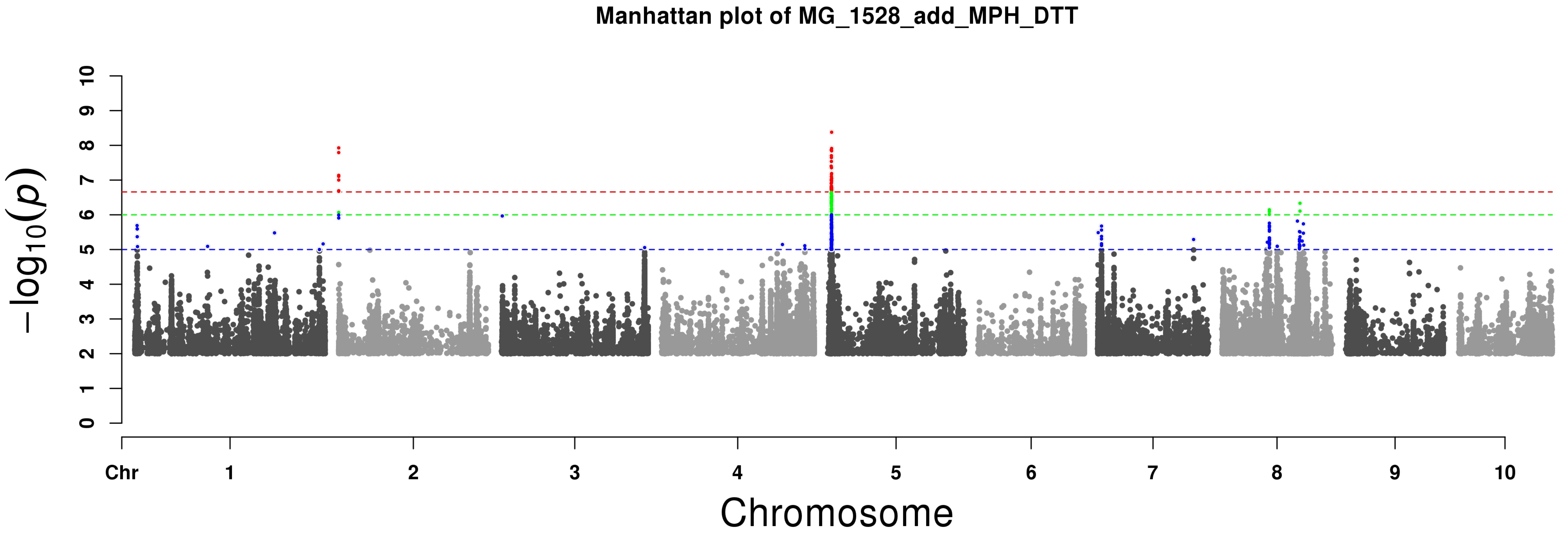

Manhattan plot of MG_1528_add_MPH_DTT
$-\log_{10}(p)$
0
1
2
3
4
5
6
7
8
9
10
Chr
1
2
3
4
5
6
7
8
9
10
Chromosome

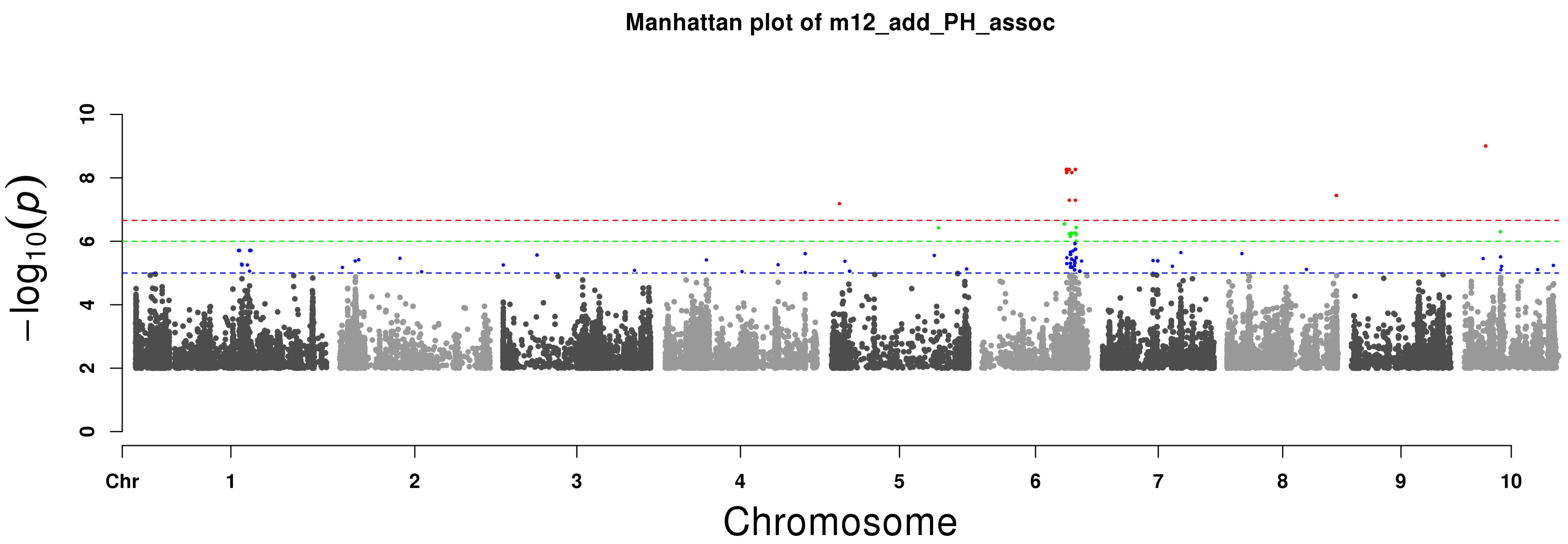

Manhattan plot of m12_add_PH_assoc
$-\log_{10}(p)$
0
2
4
6
8
10
Chr
1
2
3
4
5
6
7
8
9
10
Chromosome

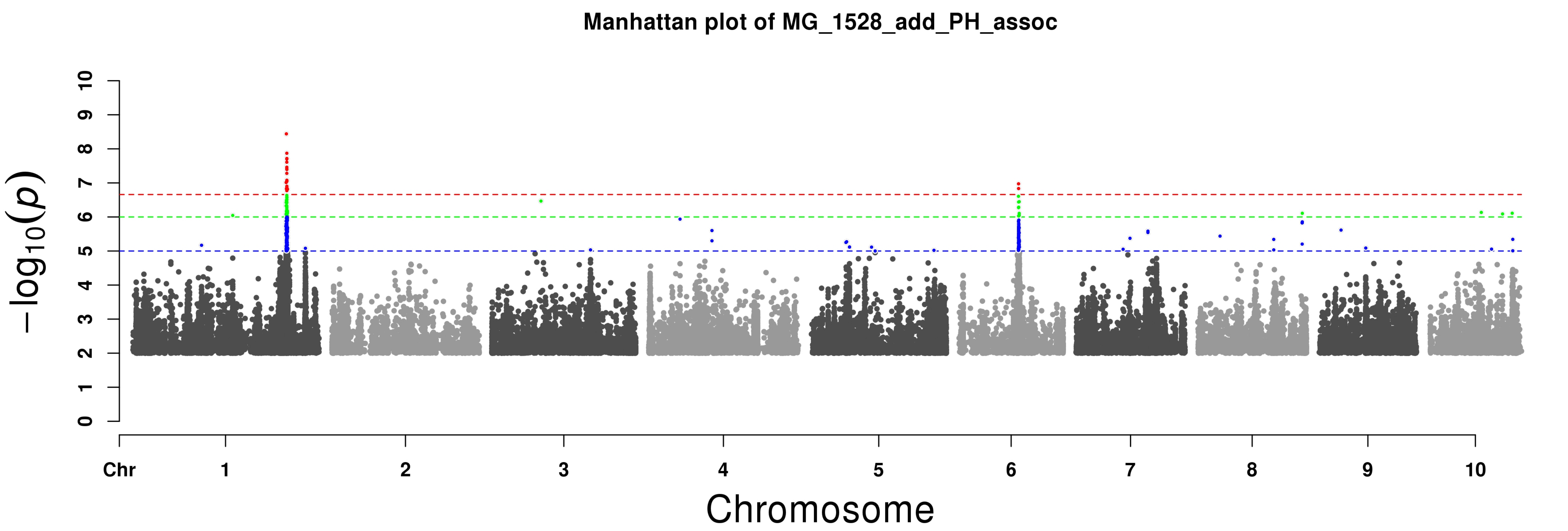

Manhattan plot of MG_1528_add_PH_assoc
$-\log_{10}(p)$
0
1
2
3
4
5
6
7
8
9
10
Chr
1
2
3
4
5
6
7
8
9
10
Chromosome

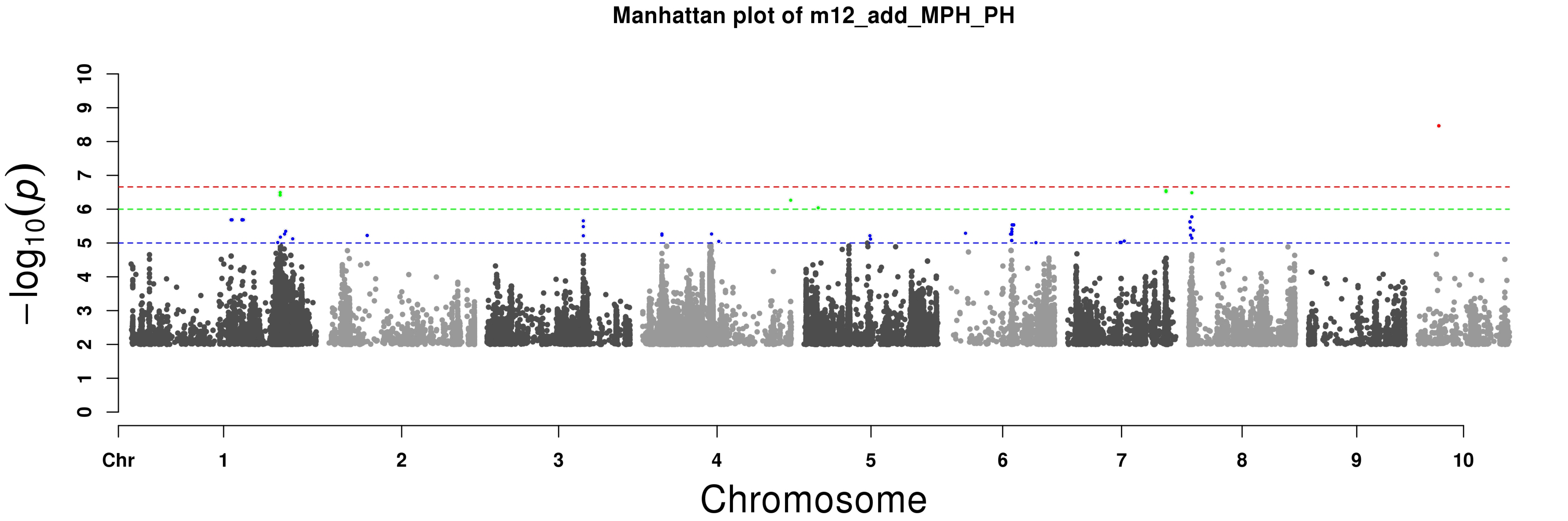

Manhattan plot of m12_add_MPH_PH
$-\log_{10}(p)$
0
1
2
3
4
5
6
7
8
9
10
Chr
1
2
3
4
5
6
7
8
9
10
Chromosome

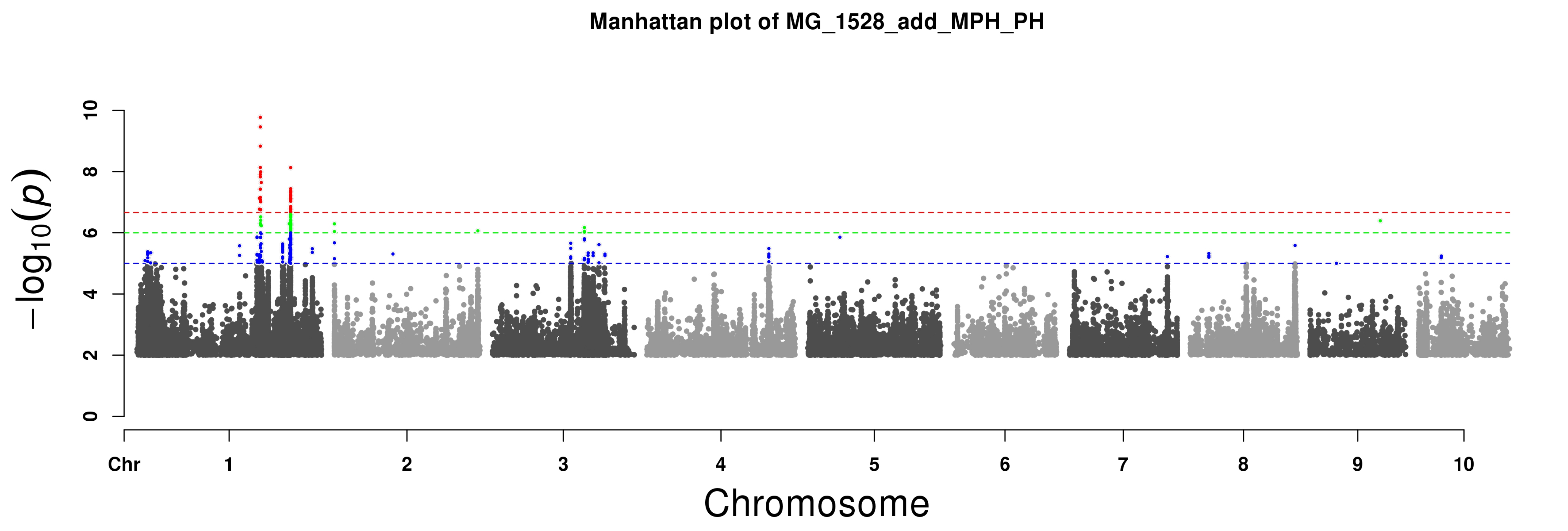

Manhattan plot of MG_1528_add_MPH_PH
−log10(p)
0
2
4
6
8
10
Chr
1
2
3
4
5
6
7
8
9
10
Chromosome

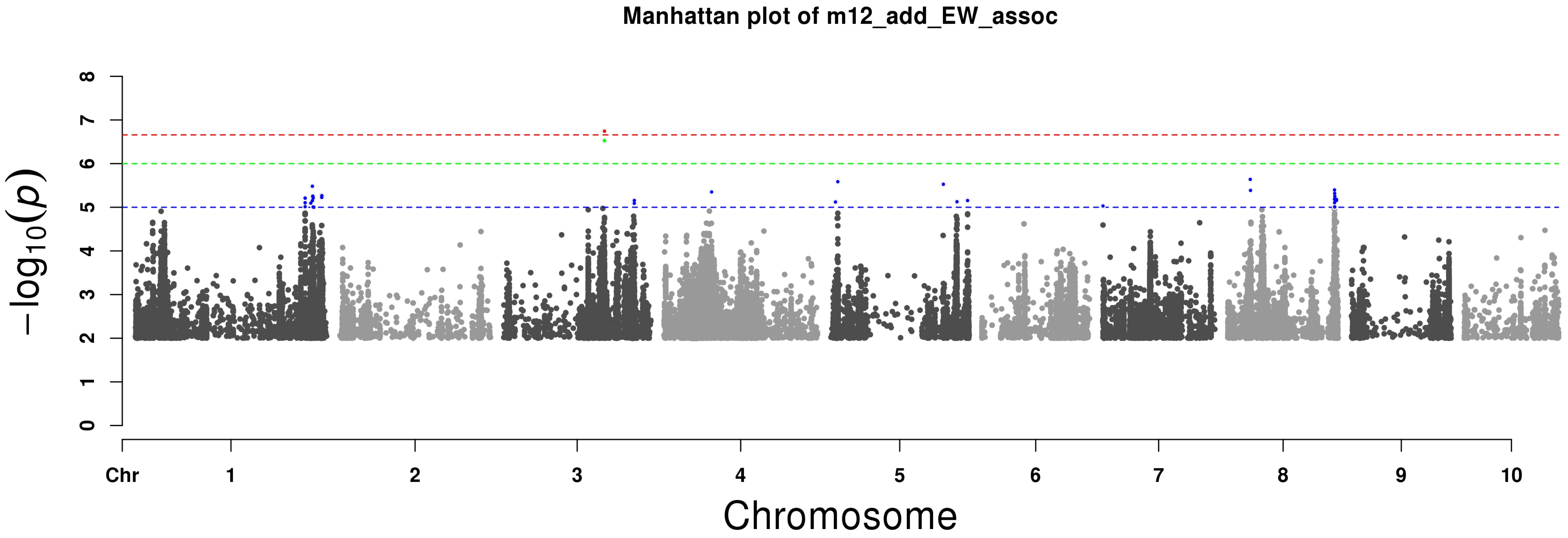
Manhattan plot of m12_add_EW_assoc
$-\log_{10}(p)$
0
1
2
3
4
5
6
7
8
Chr
1
2
3
4
5
6
7
8
9
10
Chromosome

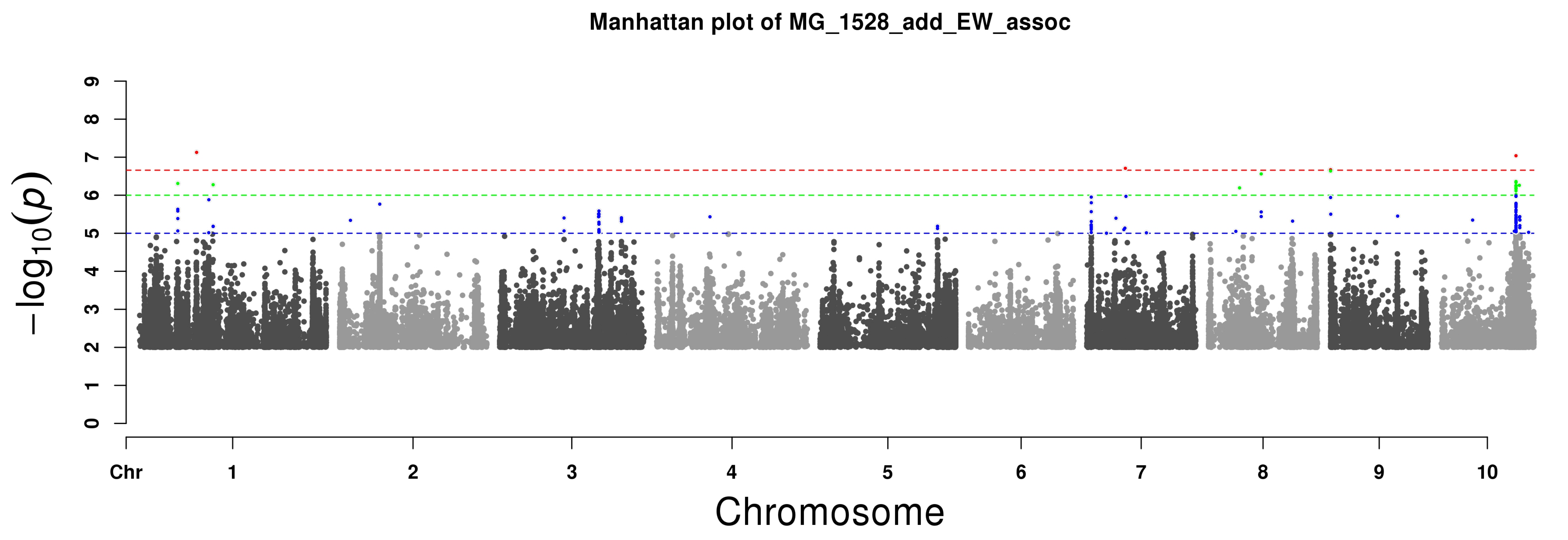

Manhattan plot of MG_1528_add_EW_assoc
$-\log_{10}(p)$
0
1
2
3
4
5
6
7
8
9
Chr
1
2
3
4
5
6
7
8
9
10
Chromosome

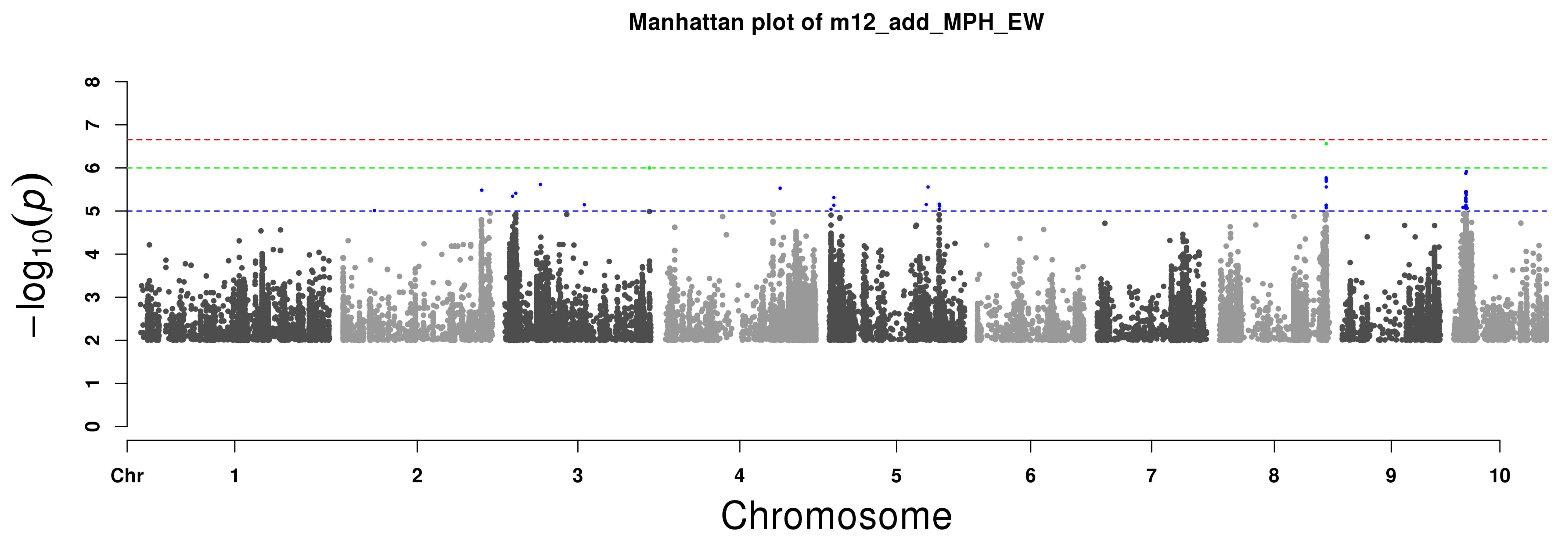

Manhattan plot of m12_add_MPH_EW
$-\log_{10}(p)$
0
1
2
3
4
5
6
7
8
Chr
1
2
3
4
5
6
7
8
9
10
Chromosome

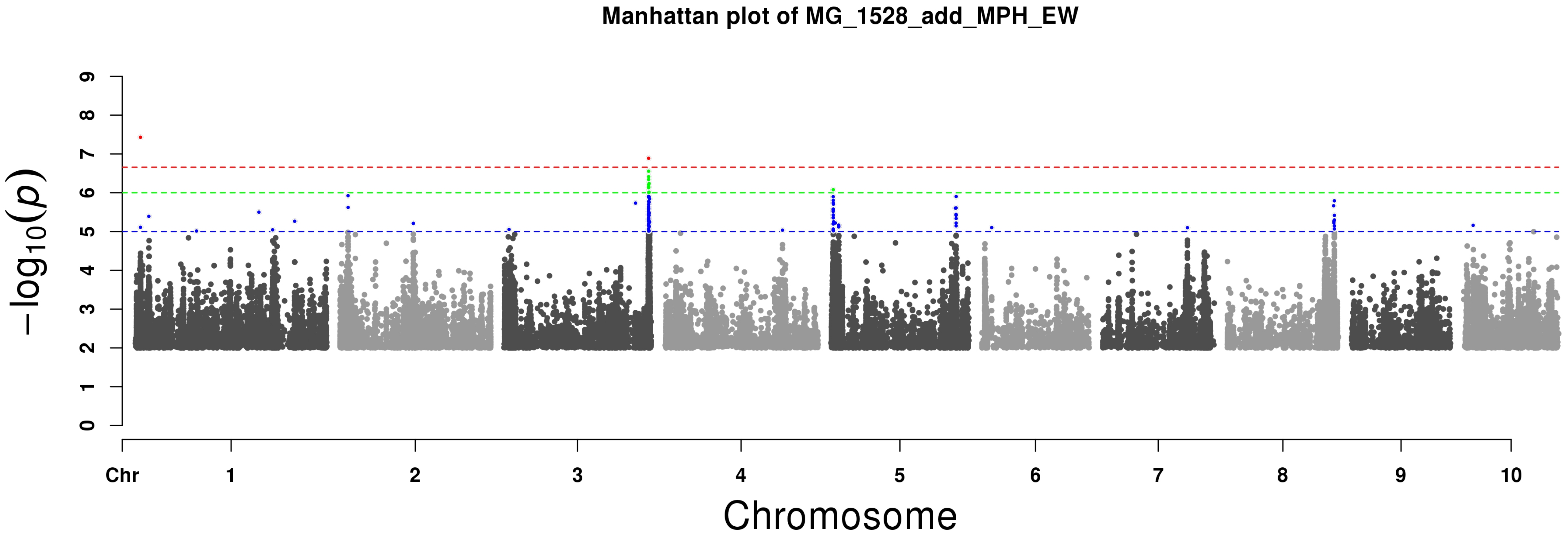

Manhattan plot of MG_1528_add_MPH_EW
$-\log_{10}(p)$
0
1
2
3
4
5
6
7
8
9
Chr
1
2
3
4
5
6
7
8
9
10
Chromosome

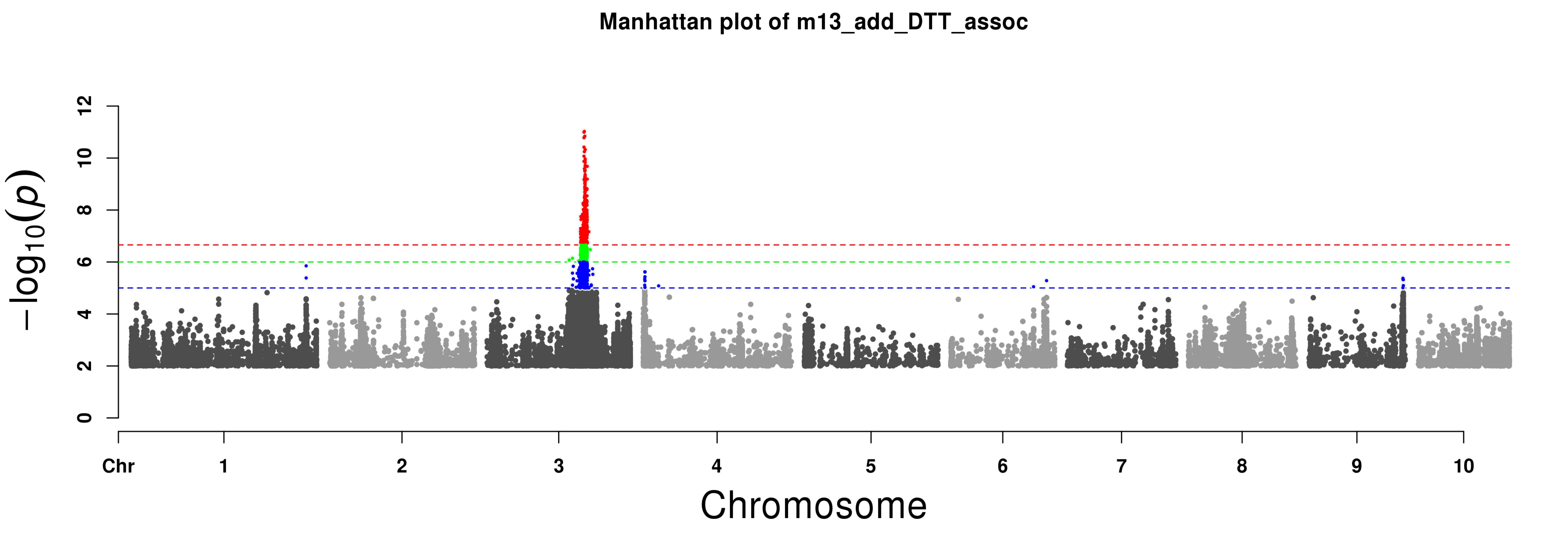
Manhattan plot of m13_add_DTT_assoc
$-\log_{10}(p)$
0
2
4
6
8
10
12
Chr
1
2
3
4
5
6
7
8
9
10
Chromosome

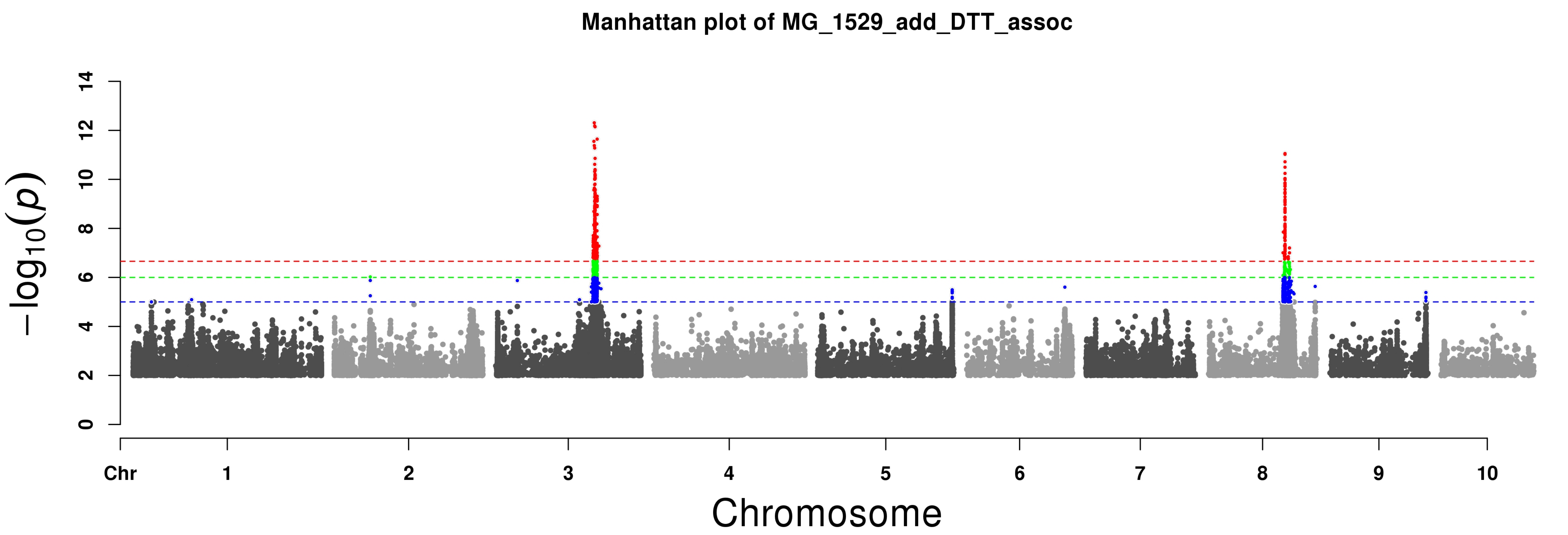

Manhattan plot of MG_1529_add_DTT_assoc
$-\log_{10}(p)$
0
2
4
6
8
10
12
14
Chr
1
2
3
4
5
6
7
8
9
10
Chromosome

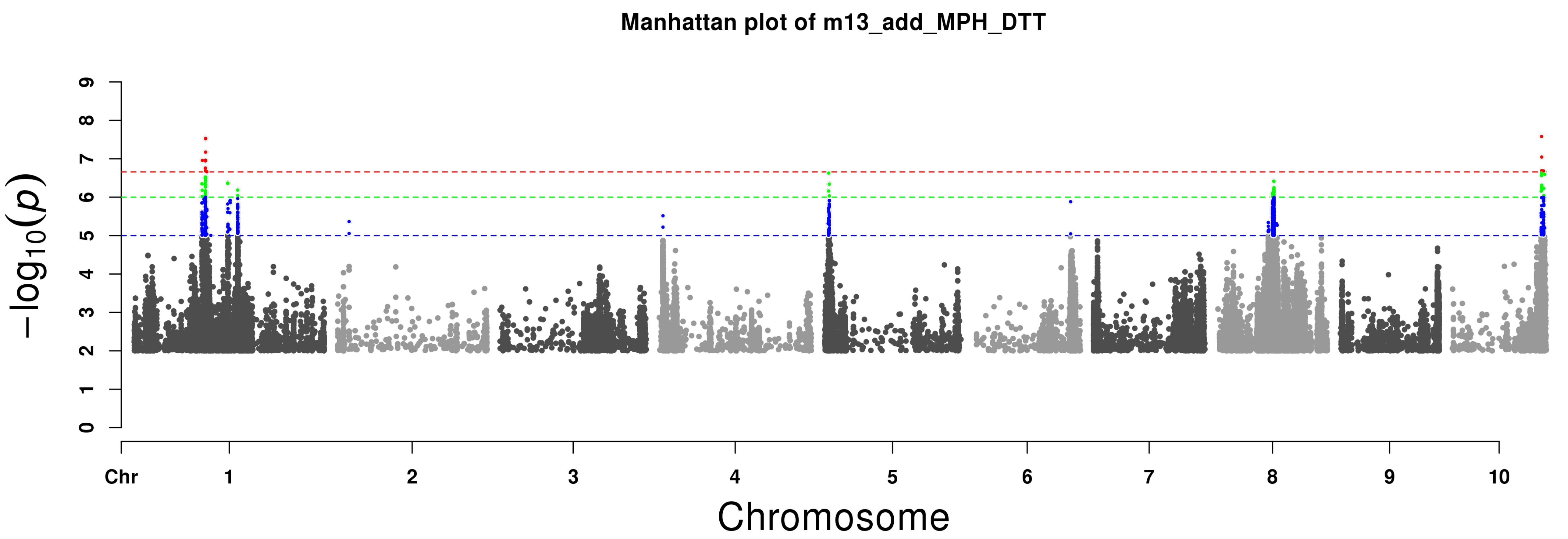

Manhattan plot of m13_add_MPH_DTT
$-\log_{10}(p)$
0
1
2
3
4
5
6
7
8
9
Chr
1
2
3
4
5
6
7
8
9
10
Chromosome

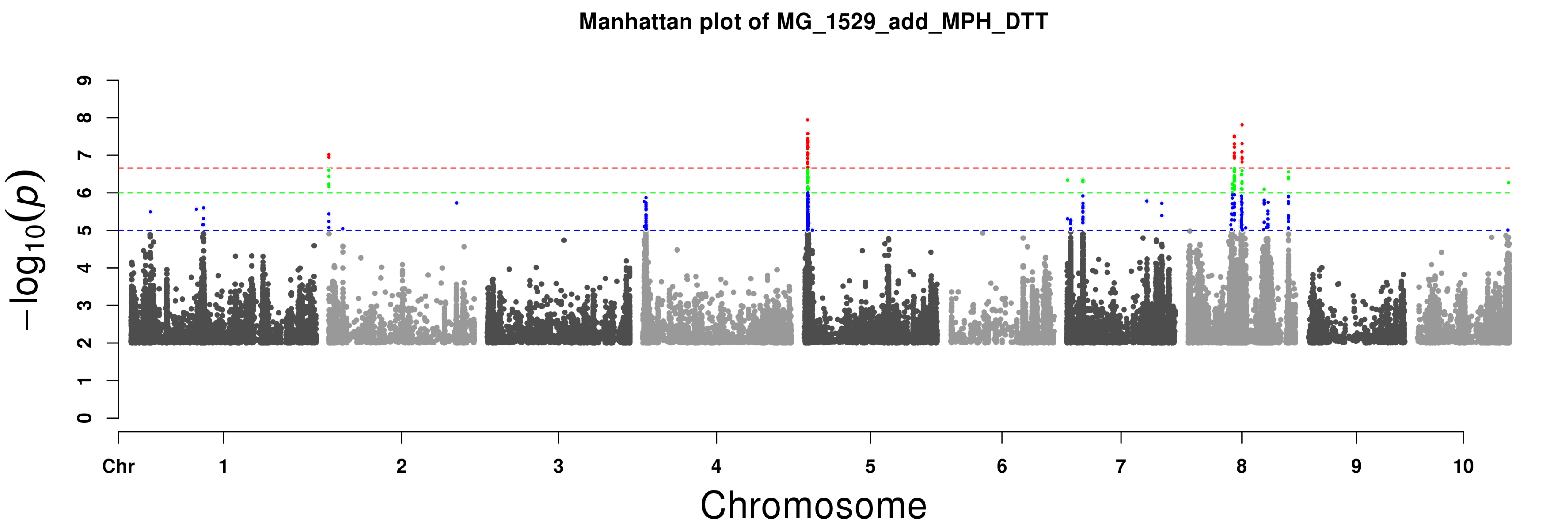

Manhattan plot of MG_1529_add_MPH_DTT
$-\log_{10}(p)$
0
1
2
3
4
5
6
7
8
9
Chr
1
2
3
4
5
6
7
8
9
10
Chromosome

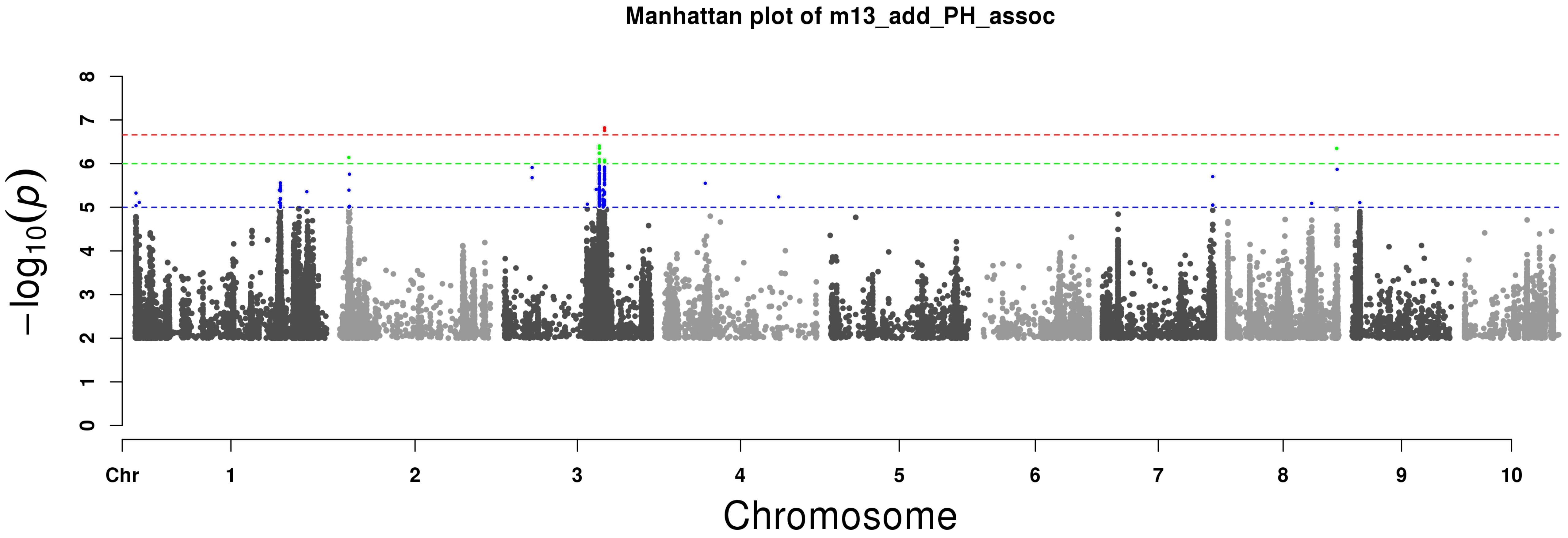
Manhattan plot of m13_add_PH_assoc
$-\log_{10}(p)$
0
1
2
3
4
5
6
7
8
Chr
1
2
3
4
5
6
7
8
9
10
Chromosome

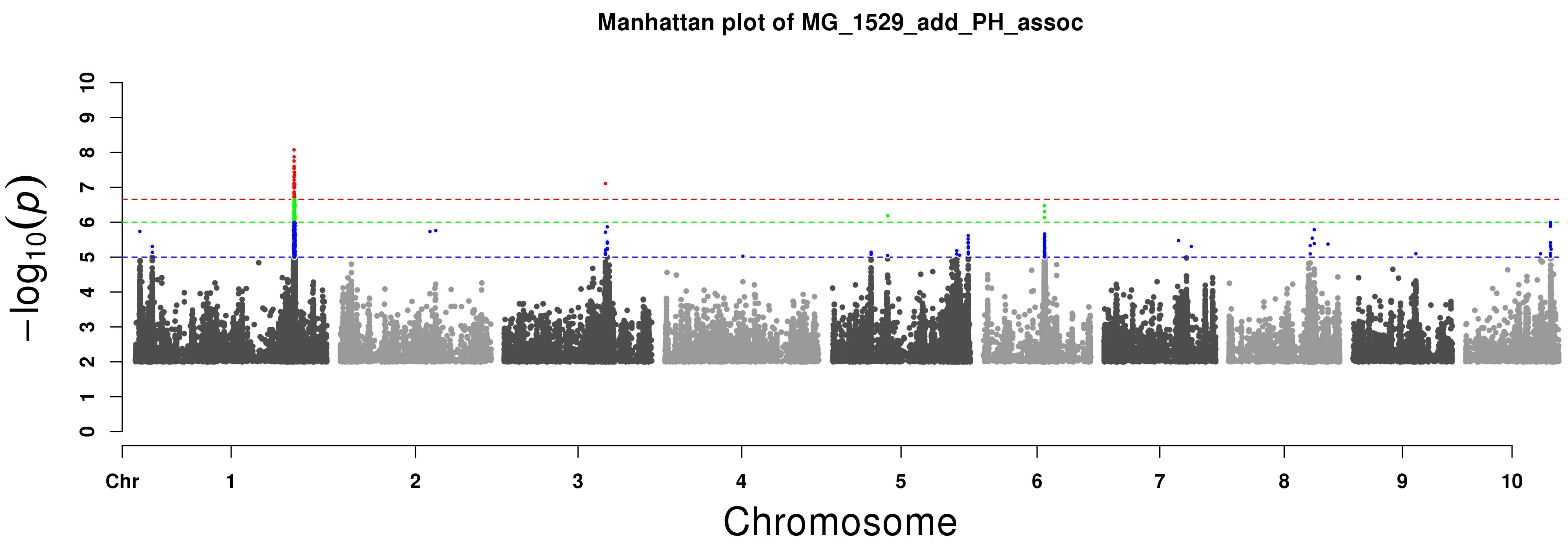
Manhattan plot of MG_1529_add_PH_assoc
$-\log_{10}(p)$
0
1
2
3
4
5
6
7
8
9
10
Chr
1
2
3
4
5
6
7
8
9
10
Chromosome

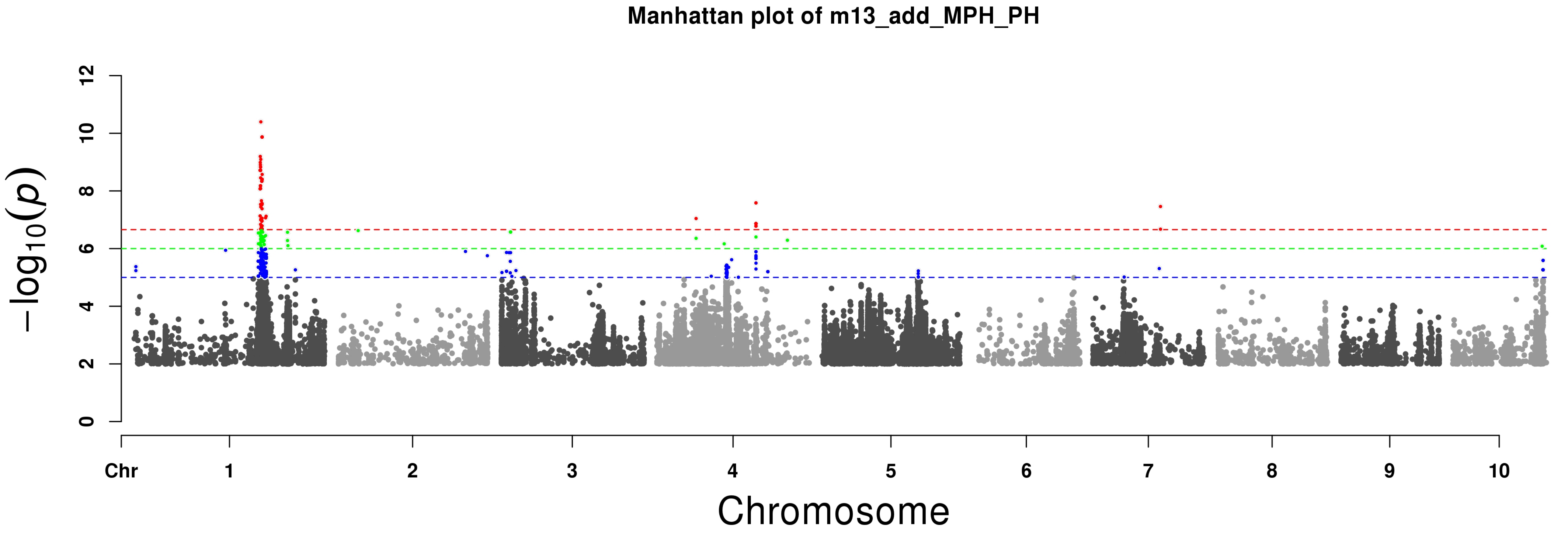

Manhattan plot of m13_add_MPH_PH
$-\log_{10}(p)$
0
2
4
6
8
10
12
Chr
1
2
3
4
5
6
7
8
9
10
Chromosome

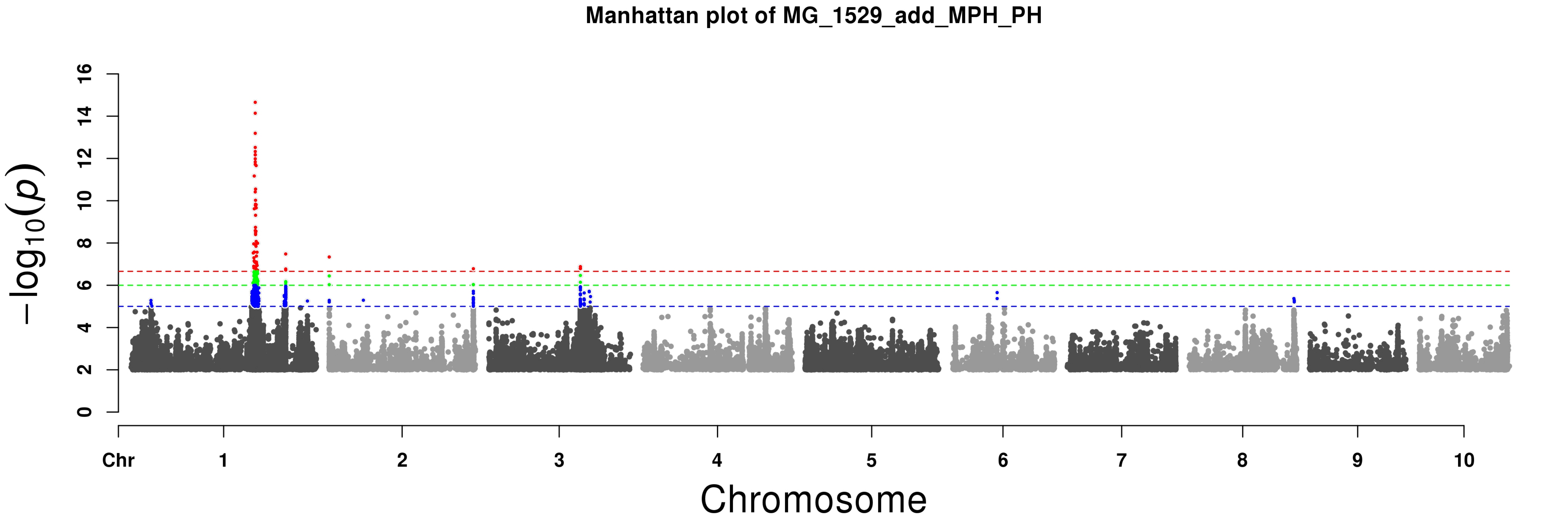

Manhattan plot of MG_1529_add_MPH_PH
$-\log_{10}(p)$
0
2
4
6
8
10
12
14
16
Chr
1
2
3
4
5
6
7
8
9
10
Chromosome

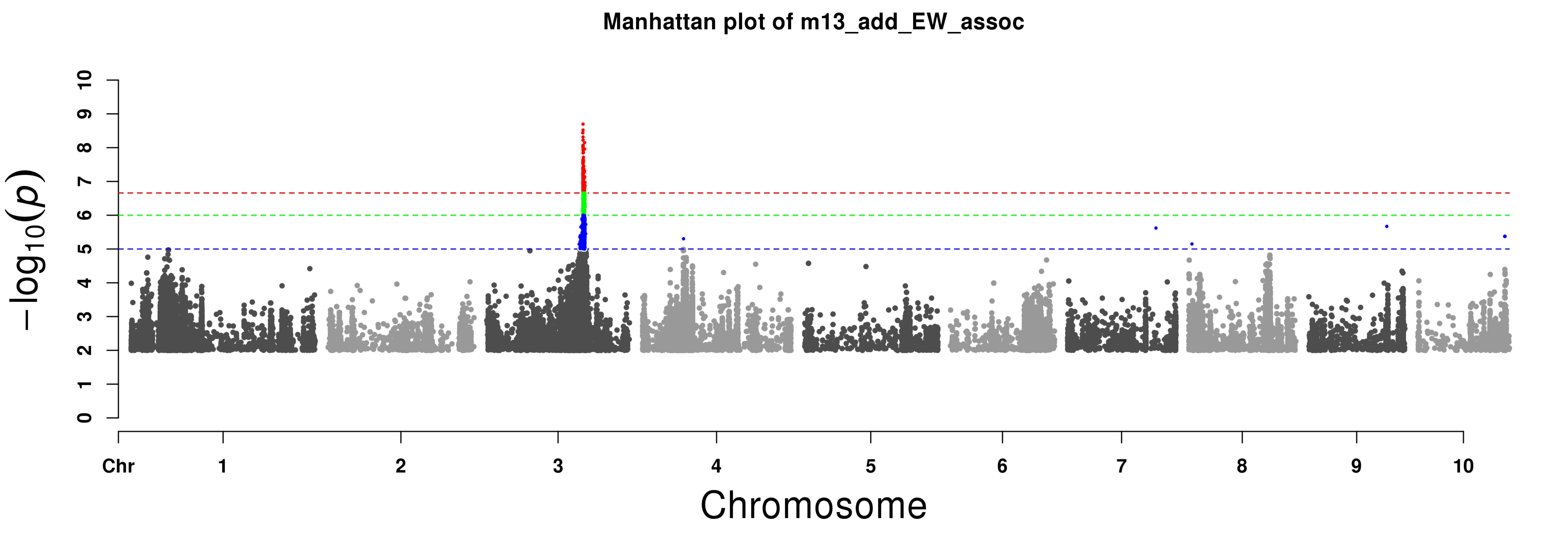

Manhattan plot of m13_add_EW_assoc
$-\log_{10}(p)$
0
1
2
3
4
5
6
7
8
9
10
Chr
1
2
3
4
5
6
7
8
9
10
Chromosome

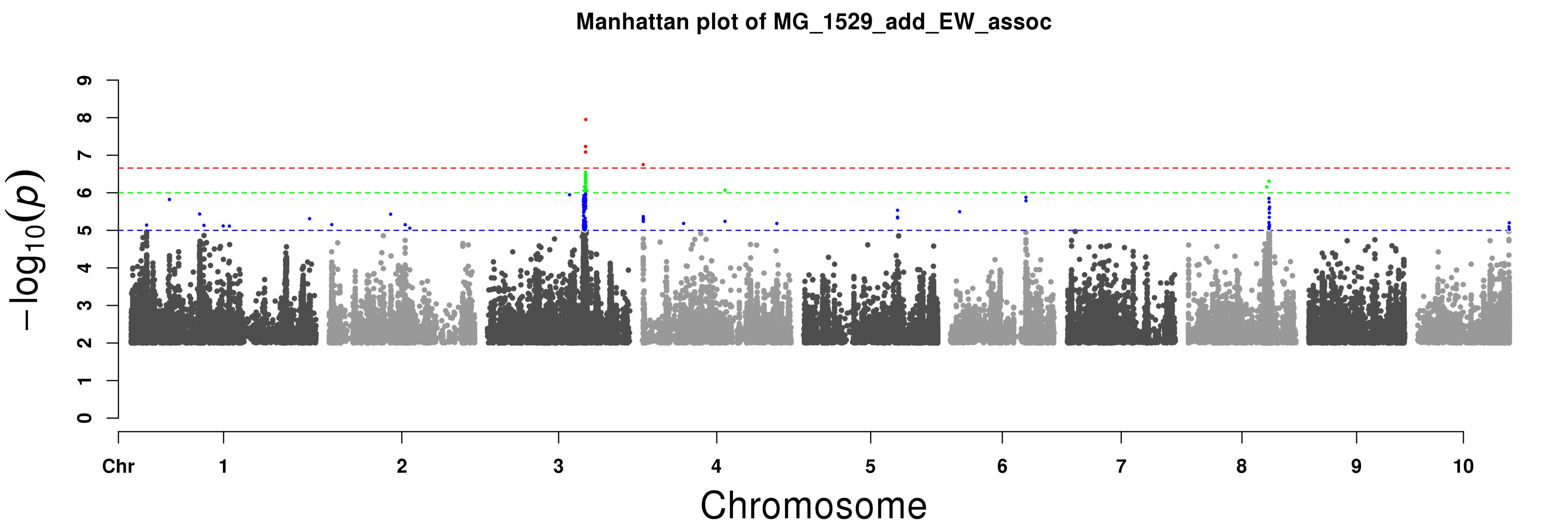

Manhattan plot of MG_1529_add_EW_assoc
$-\log_{10}(p)$
0
1
2
3
4
5
6
7
8
9
Chr
1
2
3
4
5
6
7
8
9
10
Chromosome

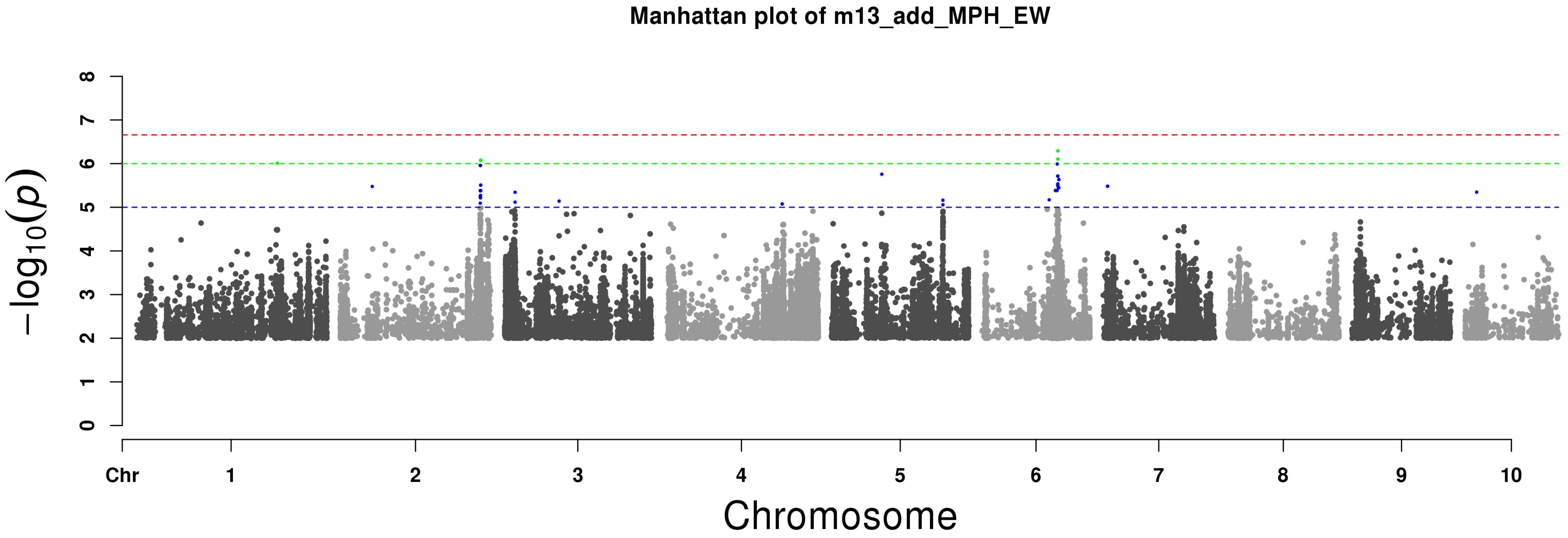
Manhattan plot of m13_add_MPH_EW
$-\log_{10}(p)$
0
1
2
3
4
5
6
7
8
Chr
1
2
3
4
5
6
7
8
9
10
Chromosome

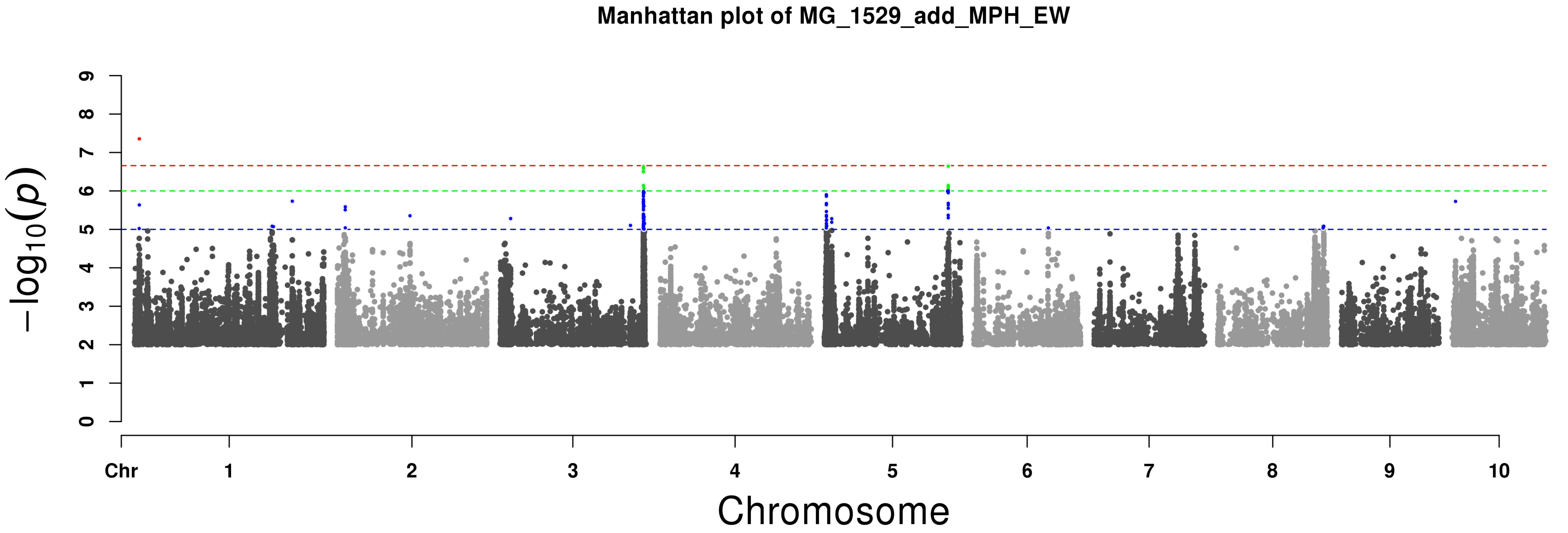
Manhattan plot of MG_1529_add_MPH_EW
$-\log_{10}(p)$
0
1
2
3
4
5
6
7
8
9
Chr
1
2
3
4
5
6
7
8
9
10
Chromosome

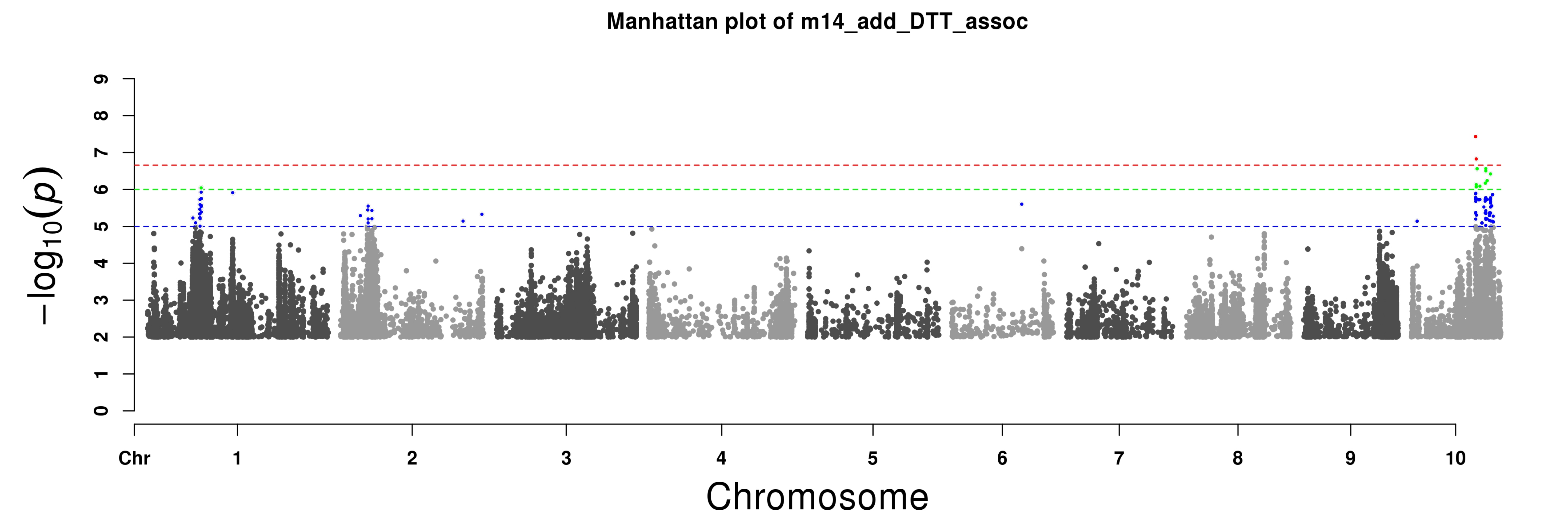
Manhattan plot of m14_add_DTT_assoc
$-\log_{10}(p)$
0
1
2
3
4
5
6
7
8
9
Chr
1
2
3
4
5
6
7
8
9
10
Chromosome

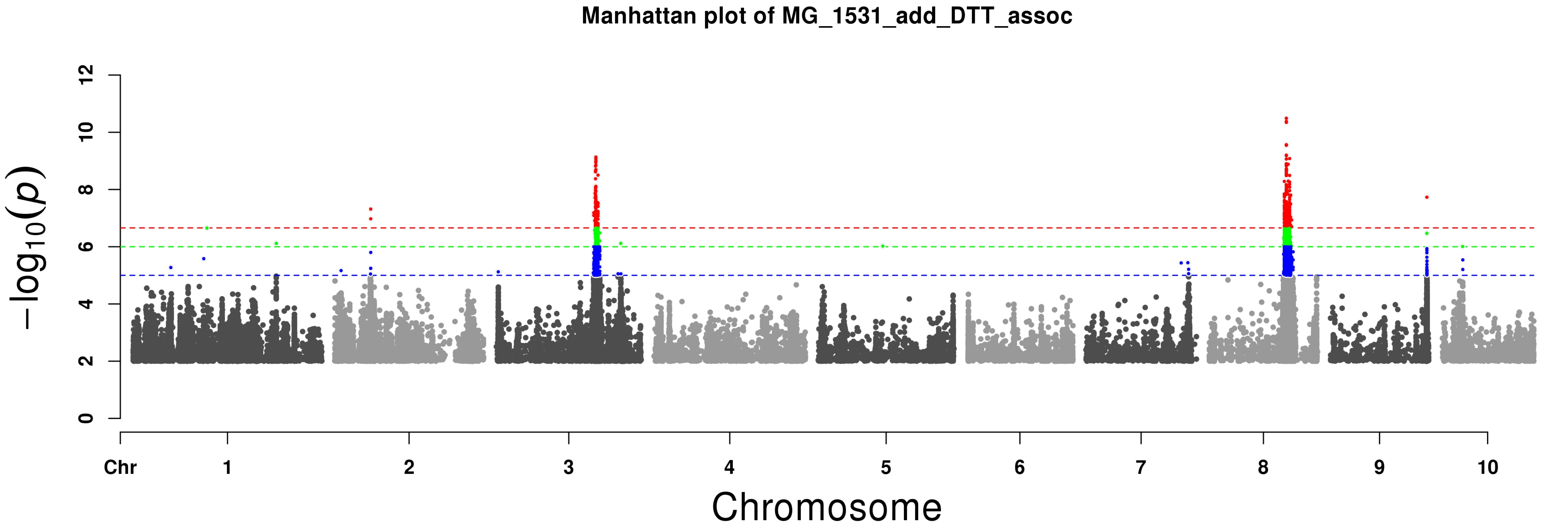

Manhattan plot of MG_1531_add_DTT_assoc
$-\log_{10}(p)$
0
2
4
6
8
10
12
Chr
1
2
3
4
5
6
7
8
9
10
Chromosome

Manhattan plot of m14_add_MPH_DTT

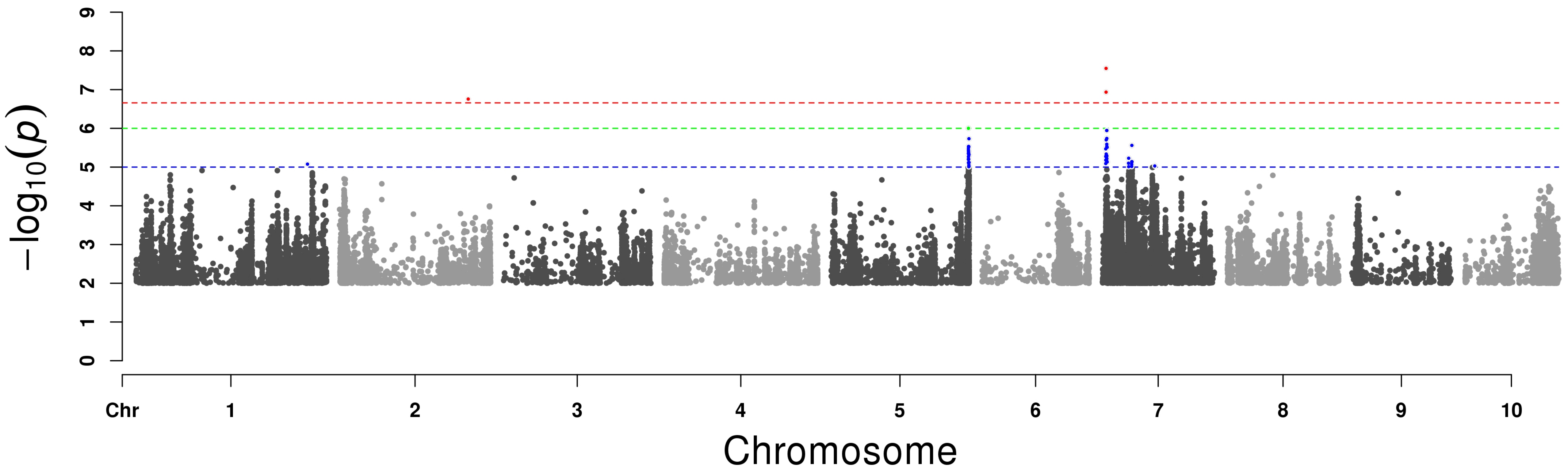

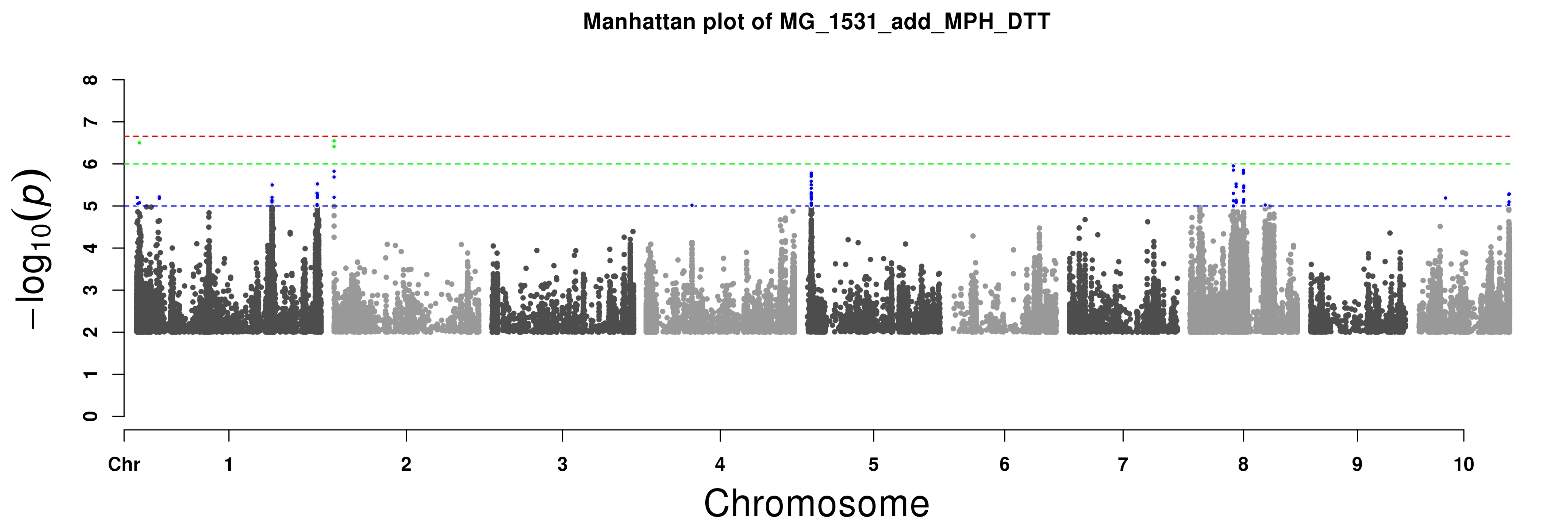

Manhattan plot of MG_1531_add_MPH_DTT
$-\log_{10}(p)$
0
1
2
3
4
5
6
7
8
Chr
1
2
3
4
5
6
7
8
9
10
Chromosome

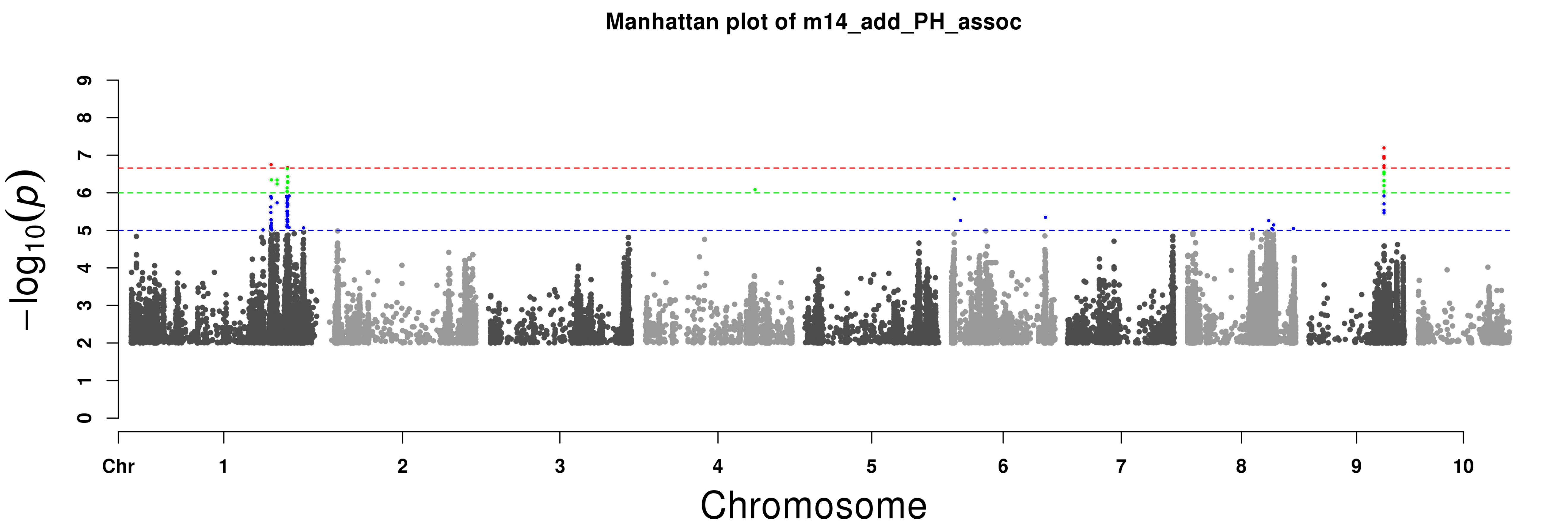
Manhattan plot of m14_add_PH_assoc
$-\log_{10}(p)$
0
1
2
3
4
5
6
7
8
9
Chr
1
2
3
4
5
6
7
8
9
10
Chromosome

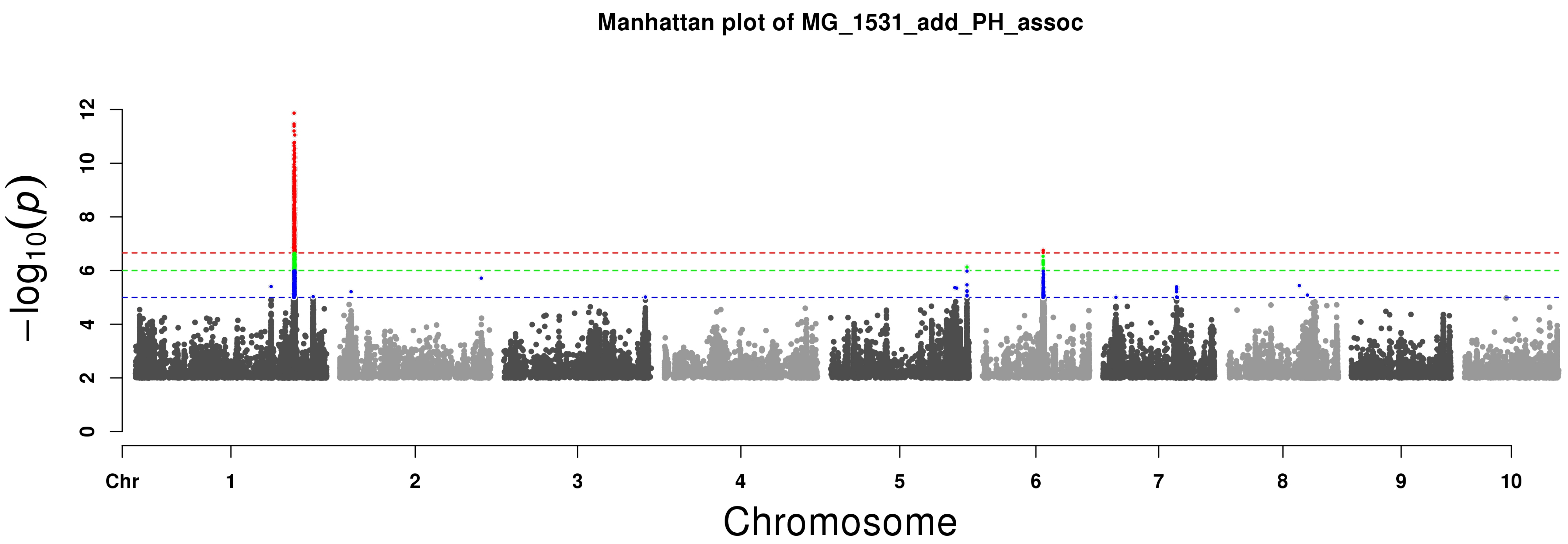
Manhattan plot of MG_1531_add_PH_assoc
$-\log_{10}(p)$
0
2
4
6
8
10
12
Chr
1
2
3
4
5
6
7
8
9
10
Chromosome

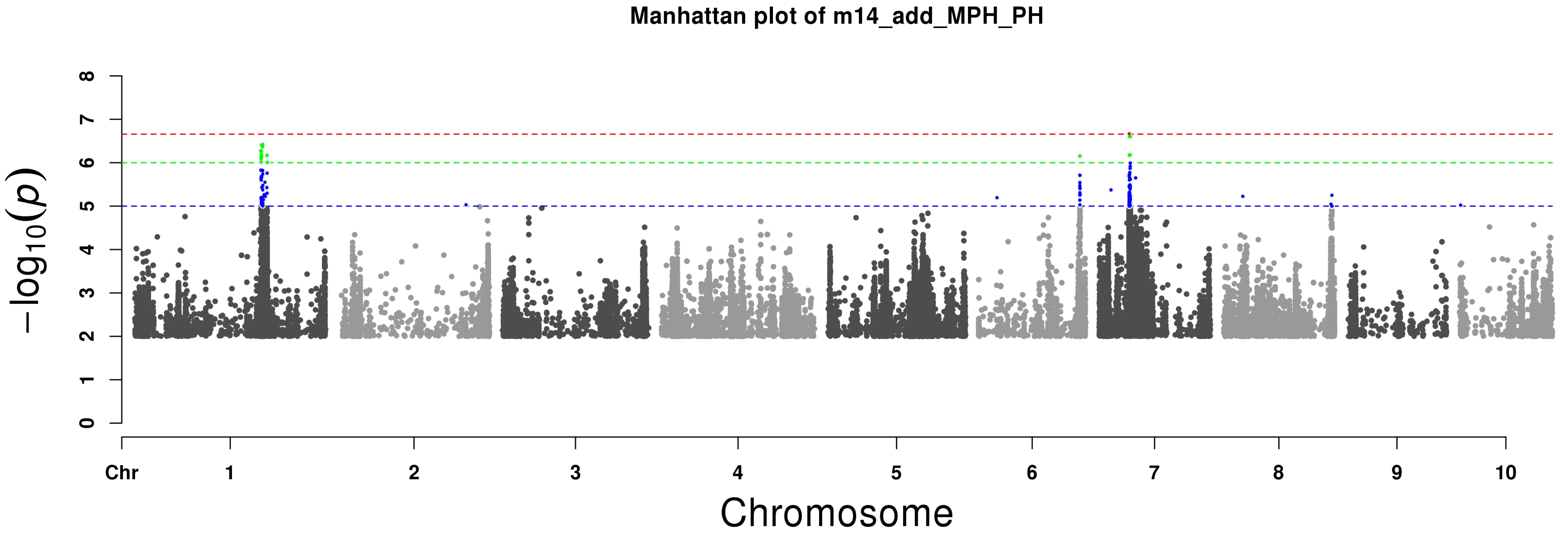
Manhattan plot of m14_add_MPH_PH
$-\log_{10}(p)$
0
1
2
3
4
5
6
7
8
Chr
1
2
3
4
5
6
7
8
9
10
Chromosome

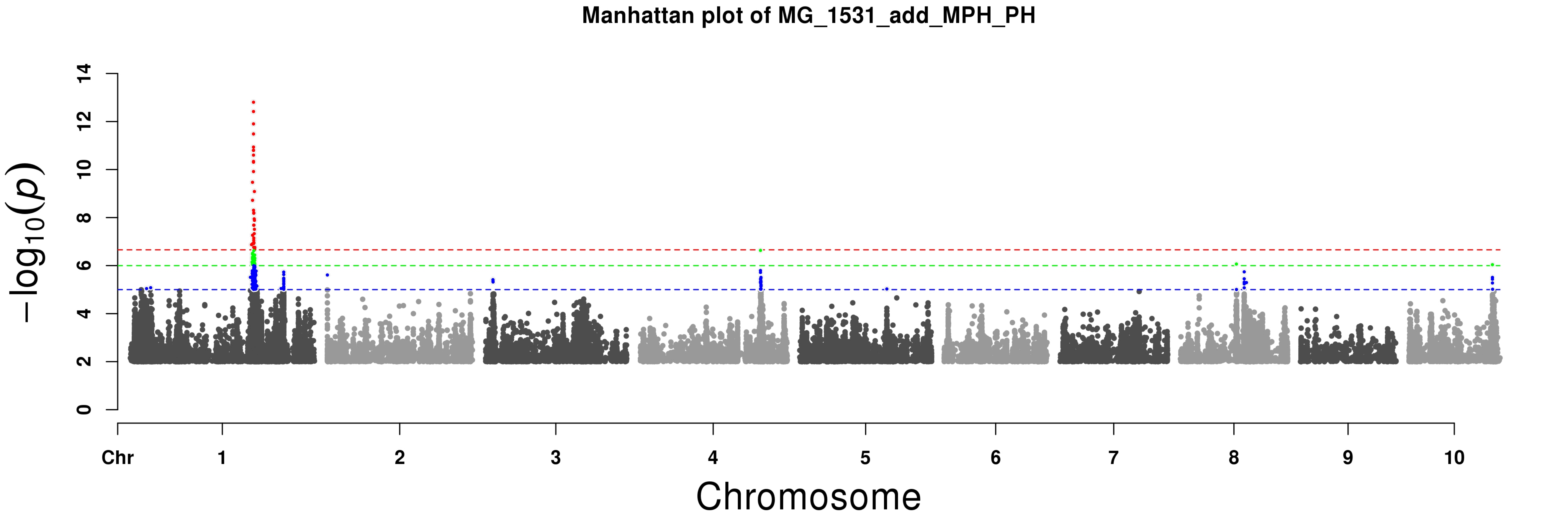
Manhattan plot of MG_1531_add_MPH_PH
$-\log_{10}(p)$
0
2
4
6
8
10
12
14
Chr
1
2
3
4
5
6
7
8
9
10
Chromosome

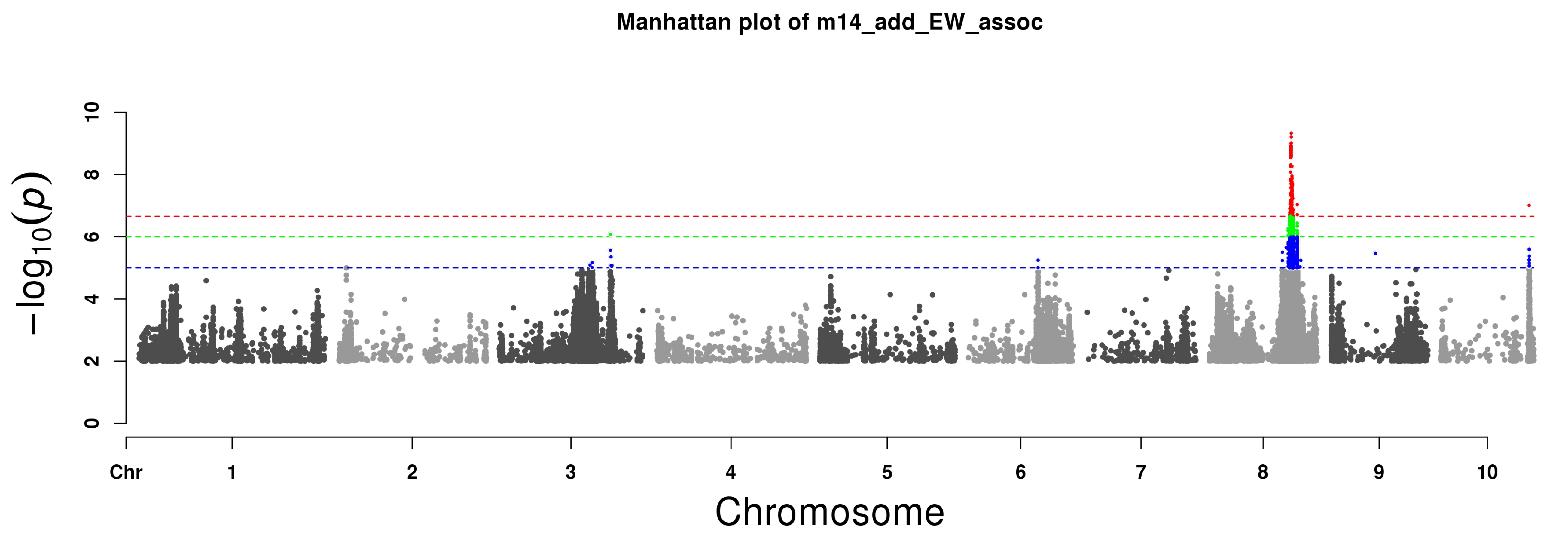

Manhattan plot of m14_add_EW_assoc
$-\log_{10}(p)$
0
2
4
6
8
10
Chr
1
2
3
4
5
6
7
8
9
10
Chromosome

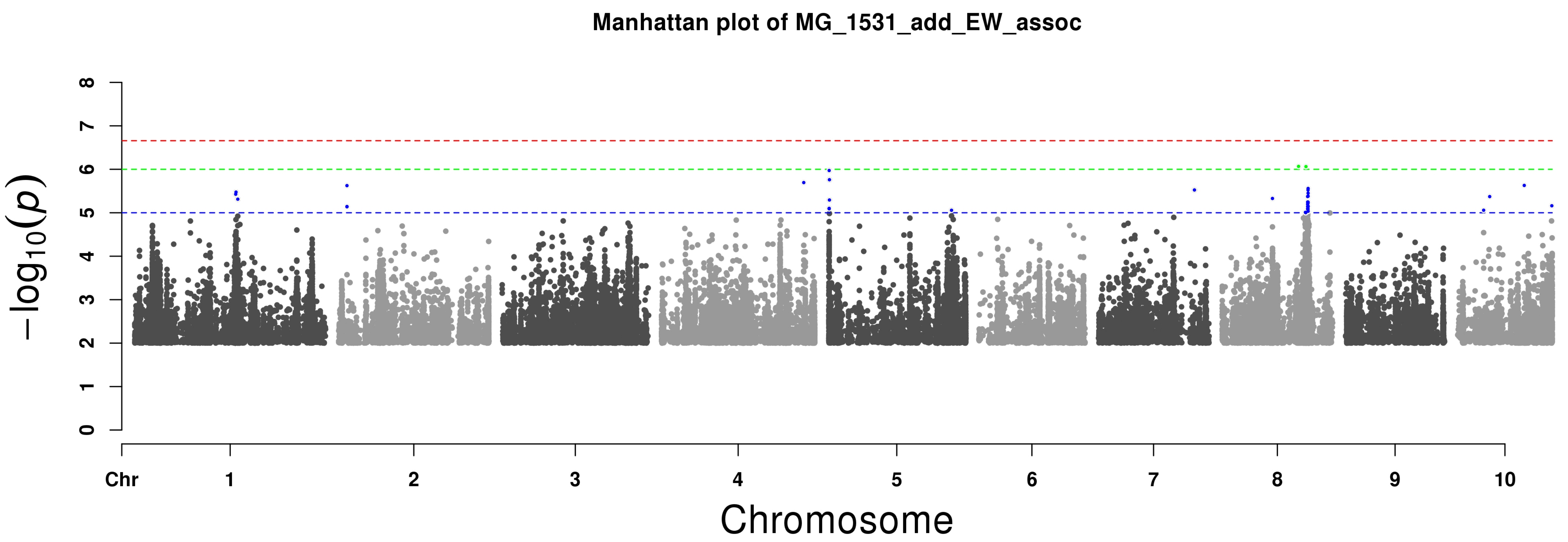

Manhattan plot of MG_1531_add_EW_assoc
$-\log_{10}(p)$
0
1
2
3
4
5
6
7
8
Chr
1
2
3
4
5
6
7
8
9
10
Chromosome

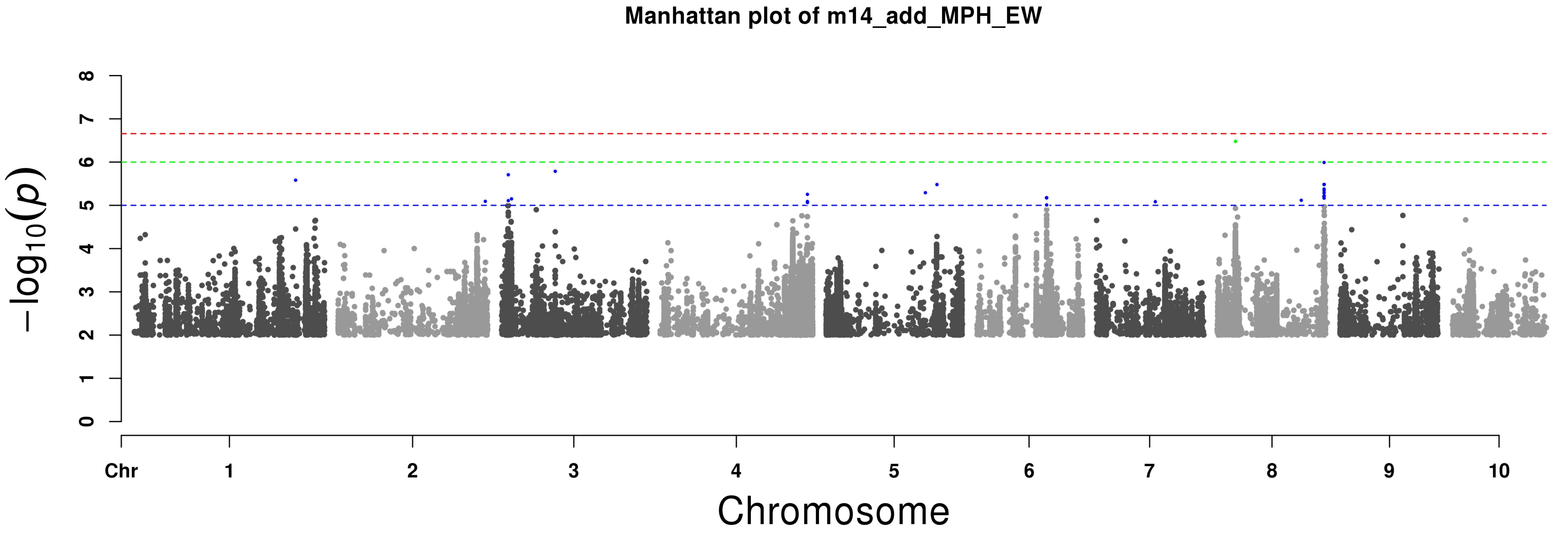

Manhattan plot of m14_add_MPH_EW
$-\log_{10}(p)$
0
1
2
3
4
5
6
7
8
Chr
1
2
3
4
5
6
7
8
9
10
Chromosome

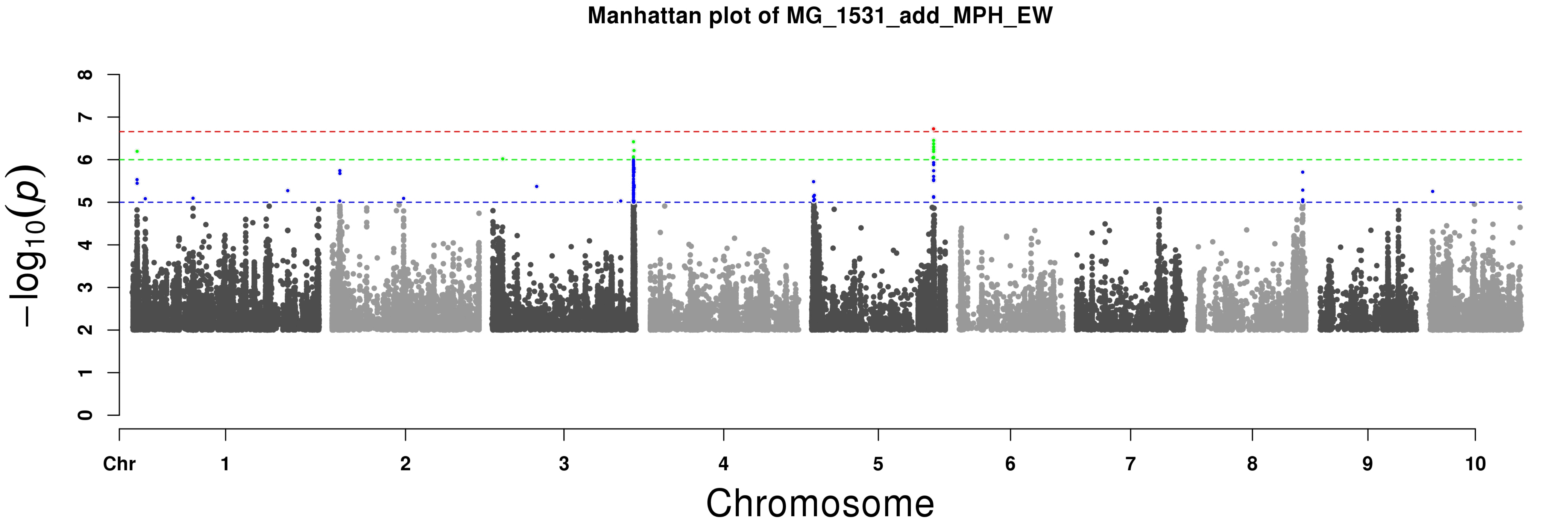

Manhattan plot of MG_1531_add_MPH_EW
$-\log_{10}(p)$
0
1
2
3
4
5
6
7
8
Chr
1
2
3
4
5
6
7
8
9
10
Chromosome

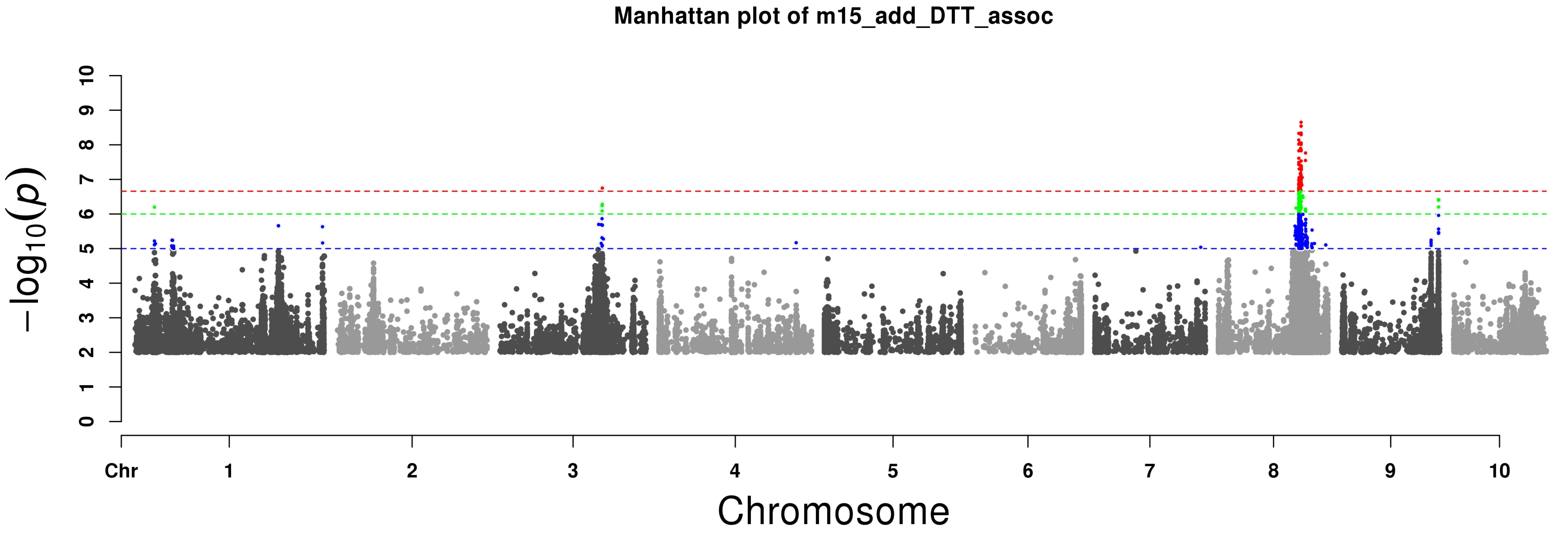

Manhattan plot of m15_add_DTT_assoc
$-\log_{10}(p)$
0
1
2
3
4
5
6
7
8
9
10
Chr
1
2
3
4
5
6
7
8
9
10
Chromosome

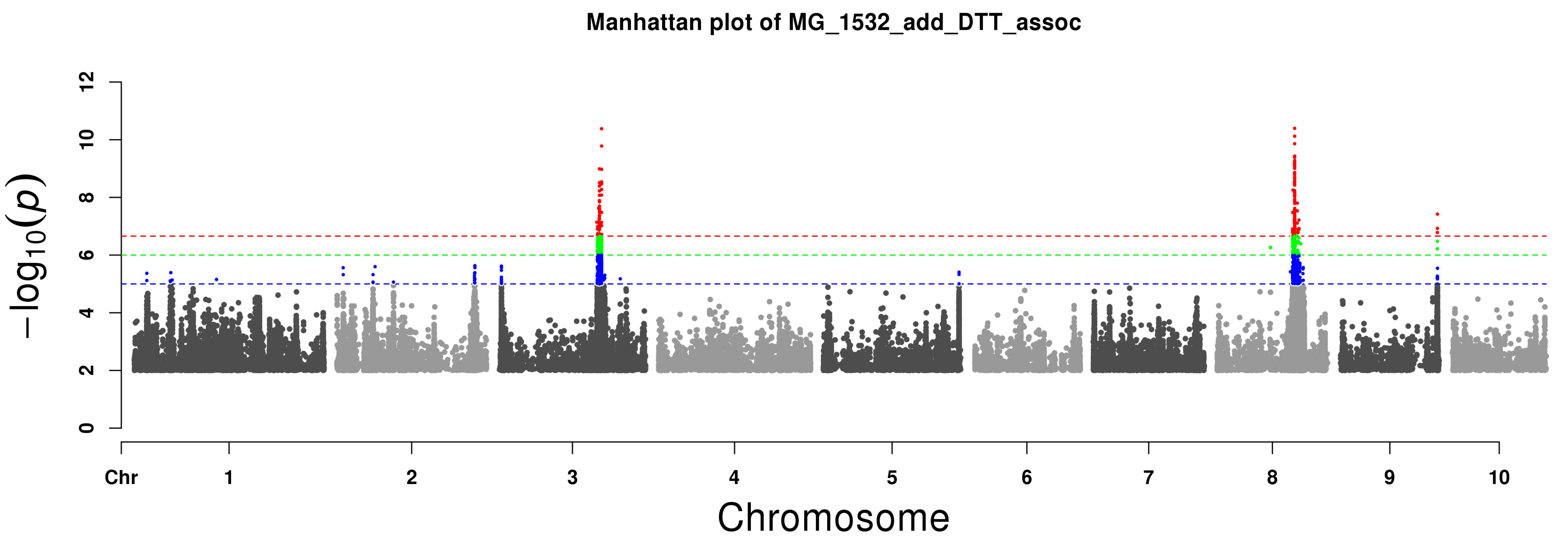

Manhattan plot of MG_1532_add_DTT_assoc
$-\log_{10}(p)$
0
2
4
6
8
10
12
Chr
1
2
3
4
5
6
7
8
9
10
Chromosome

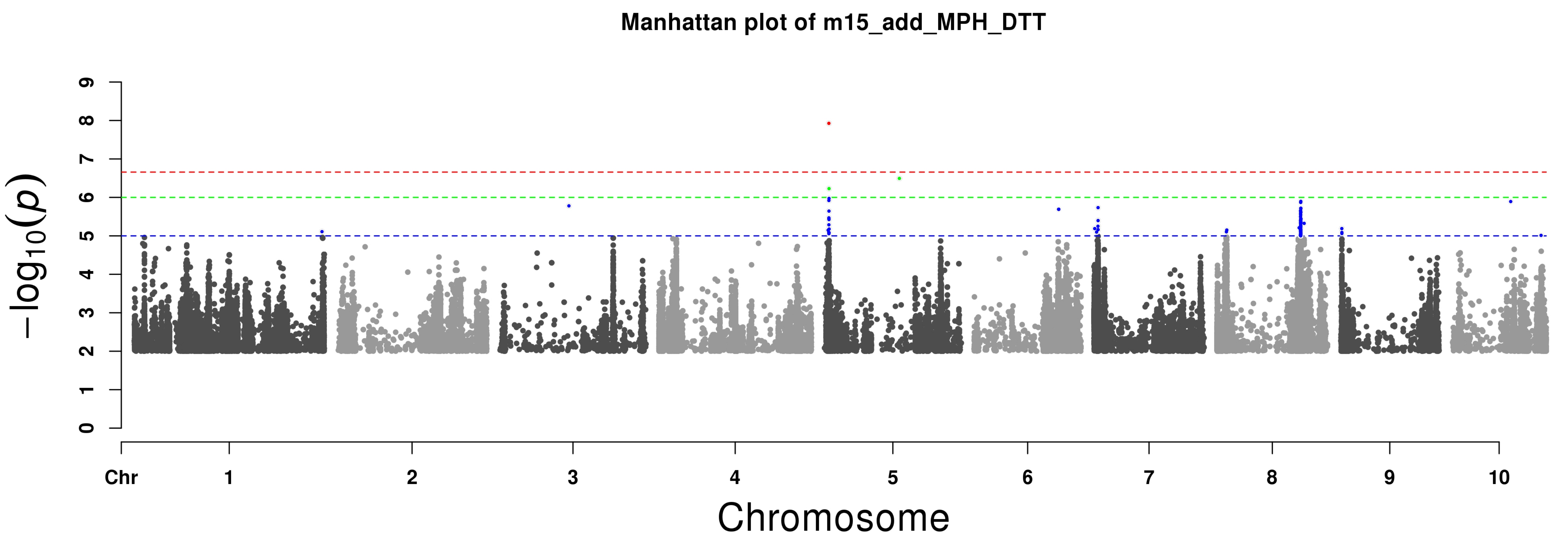
Manhattan plot of m15_add_MPH_DTT
$-\log_{10}(p)$
0
1
2
3
4
5
6
7
8
9
Chr
1
2
3
4
5
6
7
8
9
10
Chromosome

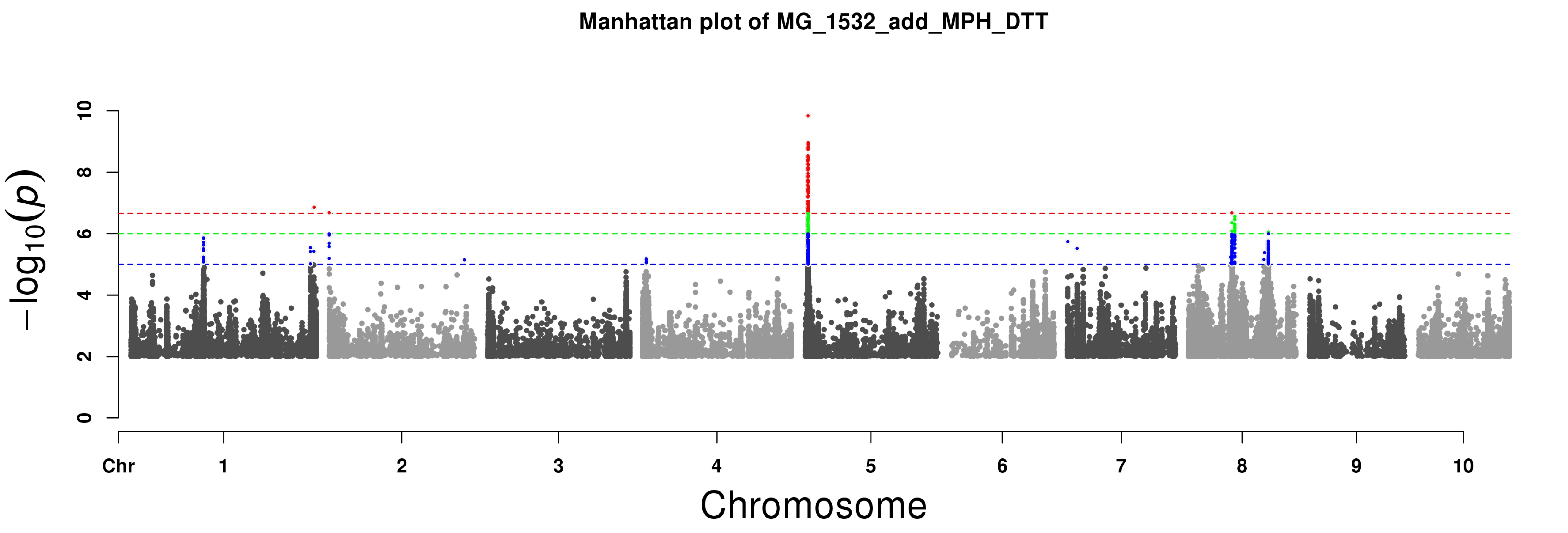
Manhattan plot of MG_1532_add_MPH_DTT
$-\log_{10}(p)$
0
2
4
6
8
10
Chr
1
2
3
4
5
6
7
8
9
10
Chromosome

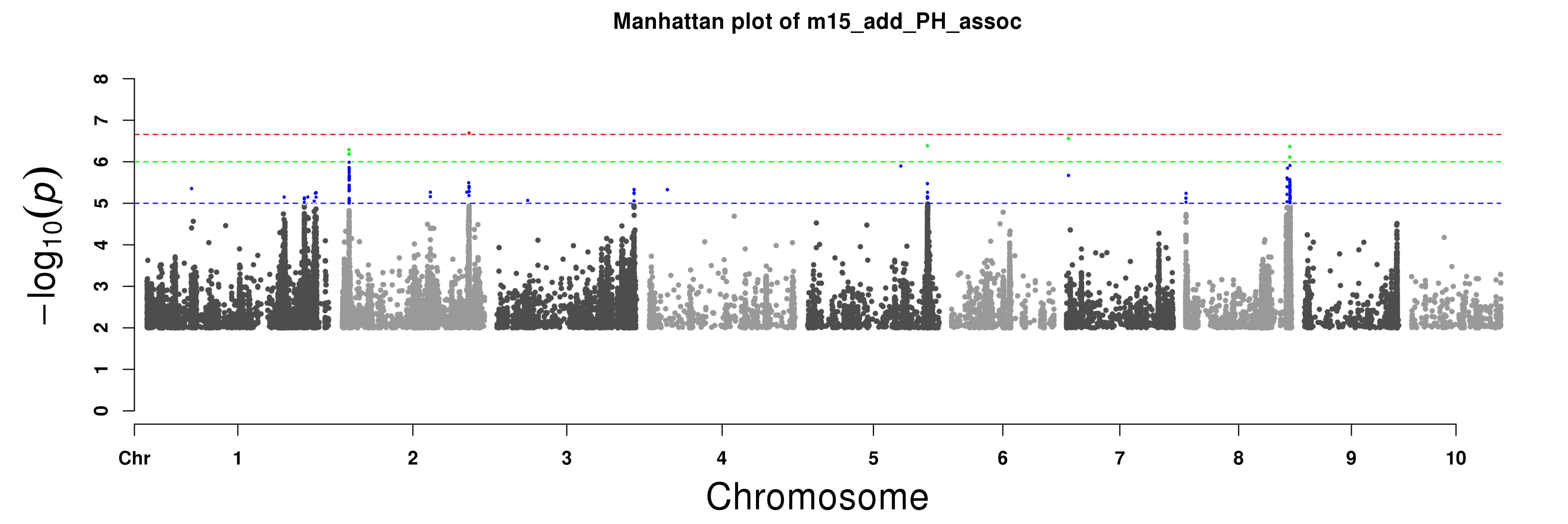

Manhattan plot of m15_add_PH_assoc
$-\log_{10}(p)$
0
1
2
3
4
5
6
7
8
Chr
1
2
3
4
5
6
7
8
9
10
Chromosome

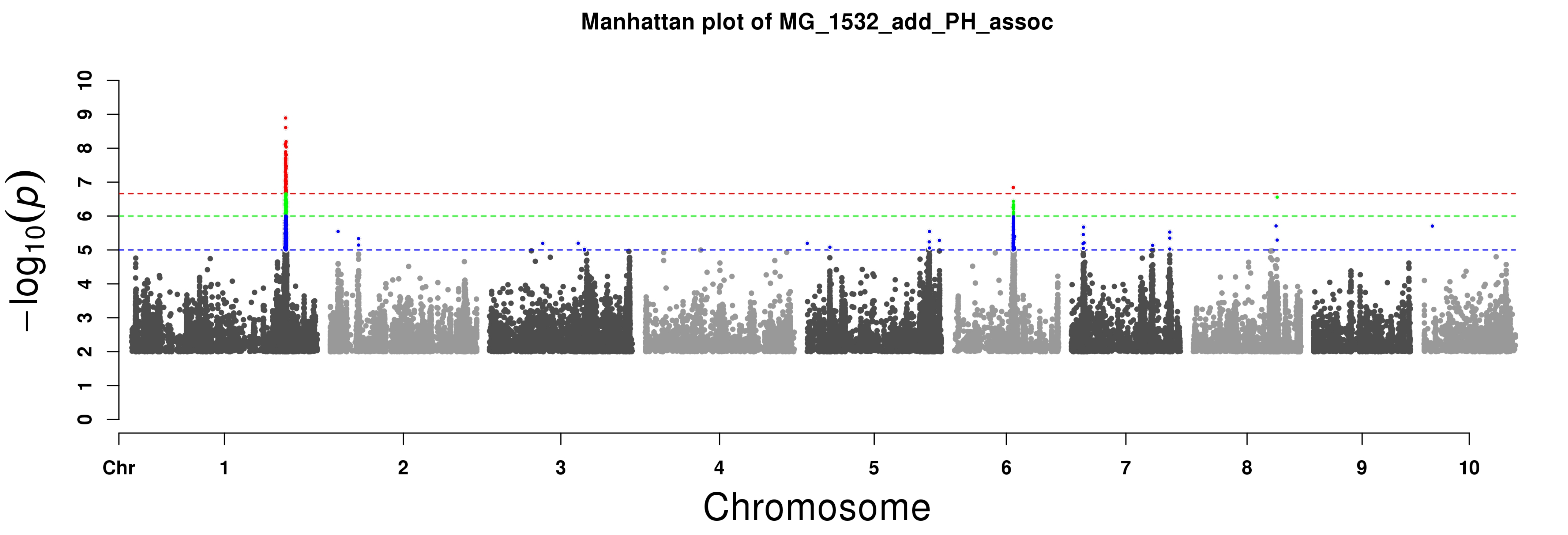

Manhattan plot of MG_1532_add_PH_assoc
$-\log_{10}(p)$
0
1
2
3
4
5
6
7
8
9
10
Chr
1
2
3
4
5
6
7
8
9
10
Chromosome

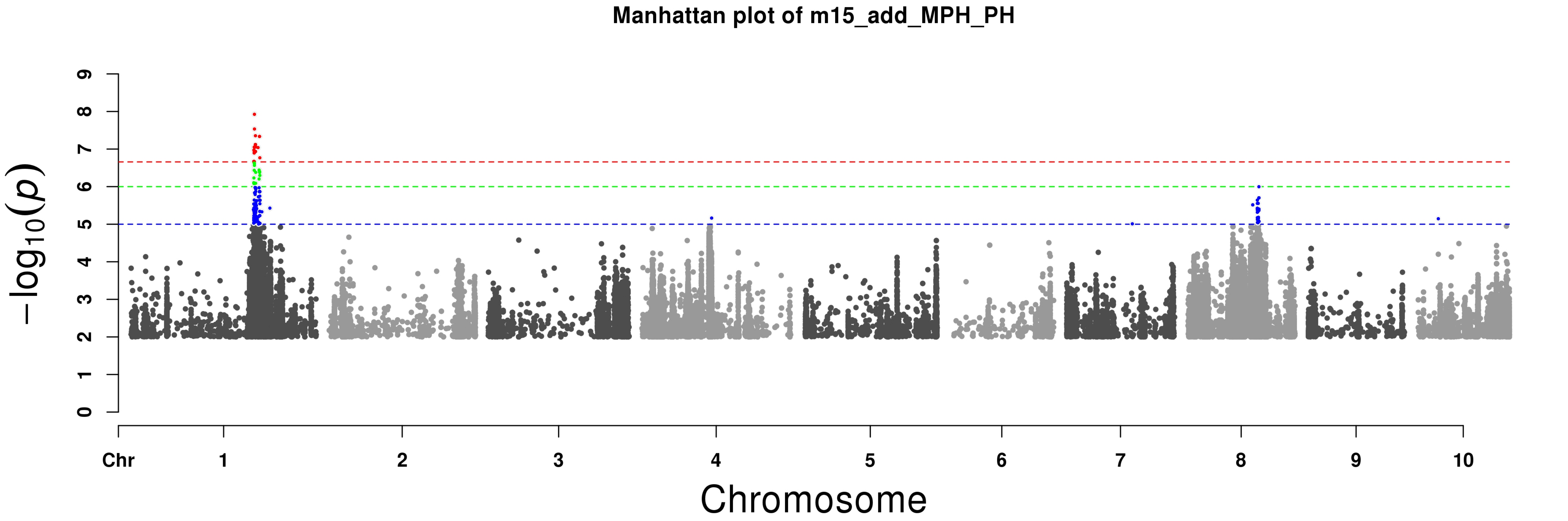

Manhattan plot of m15_add_MPH_PH
$-\log_{10}(p)$
0
1
2
3
4
5
6
7
8
9
Chr
1
2
3
4
5
6
7
8
9
10
Chromosome

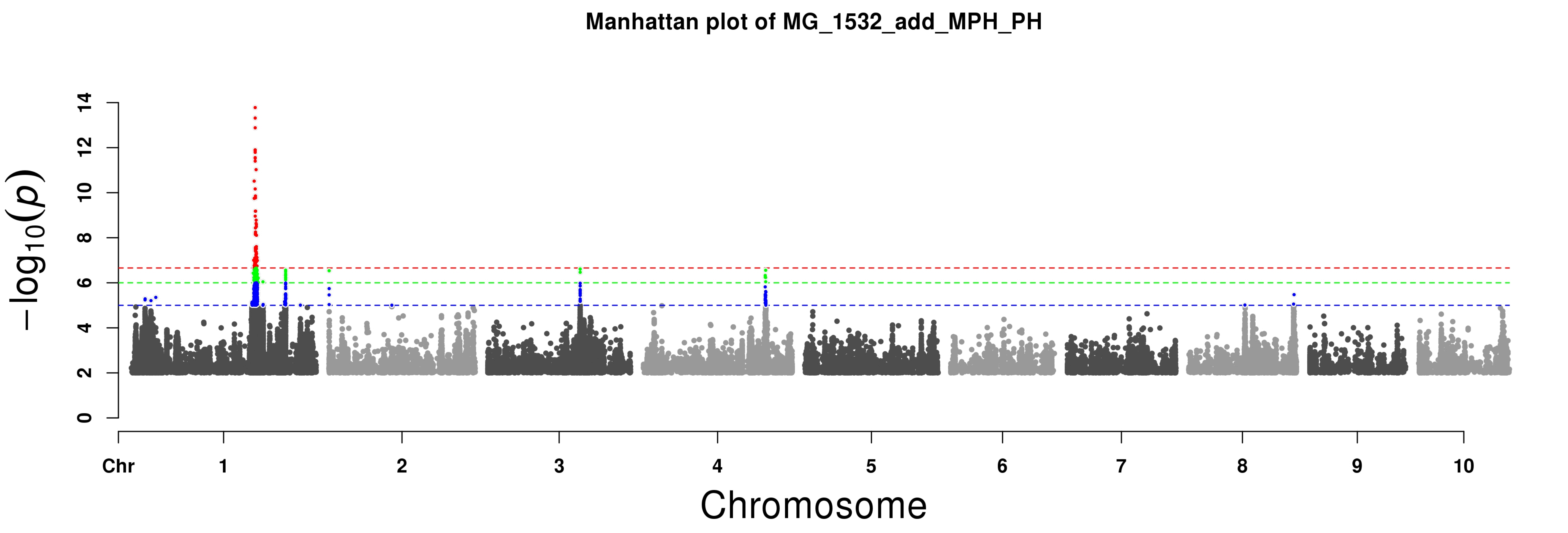

Manhattan plot of MG_1532_add_MPH_PH
$-\log_{10}(p)$
0
2
4
6
8
10
12
14
Chr
1
2
3
4
5
6
7
8
9
10
Chromosome

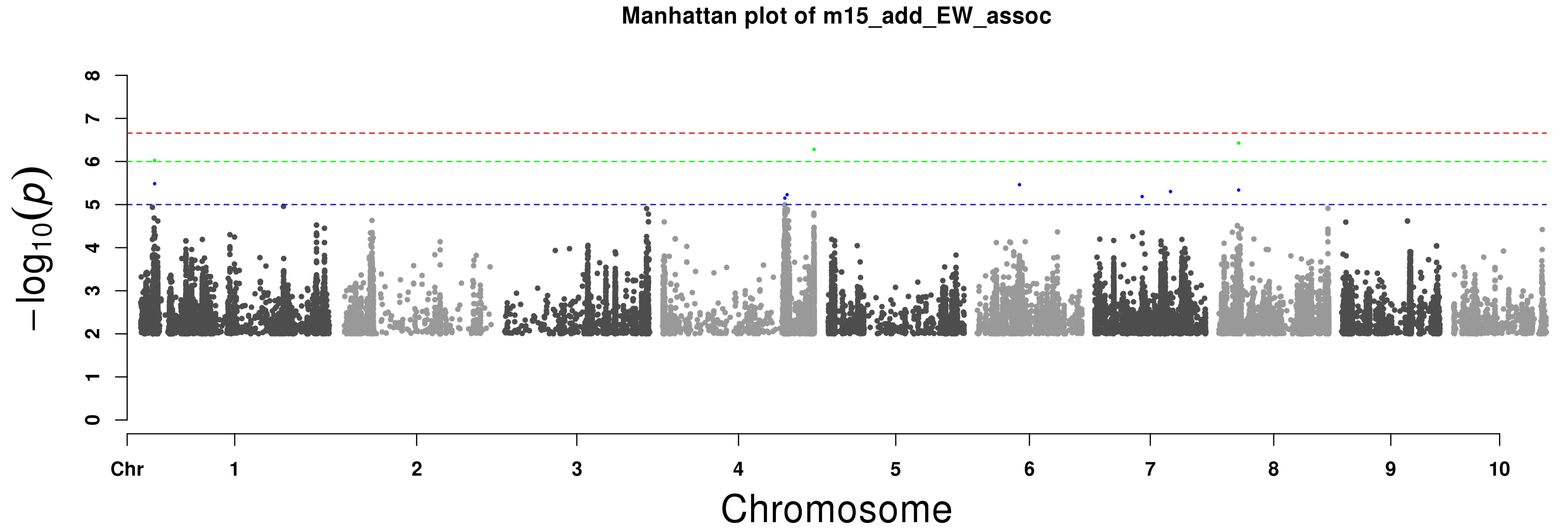
Manhattan plot of m15_add_EW_assoc
$-\log_{10}(p)$
0
1
2
3
4
5
6
7
8
Chr
1
2
3
4
5
6
7
8
9
10
Chromosome

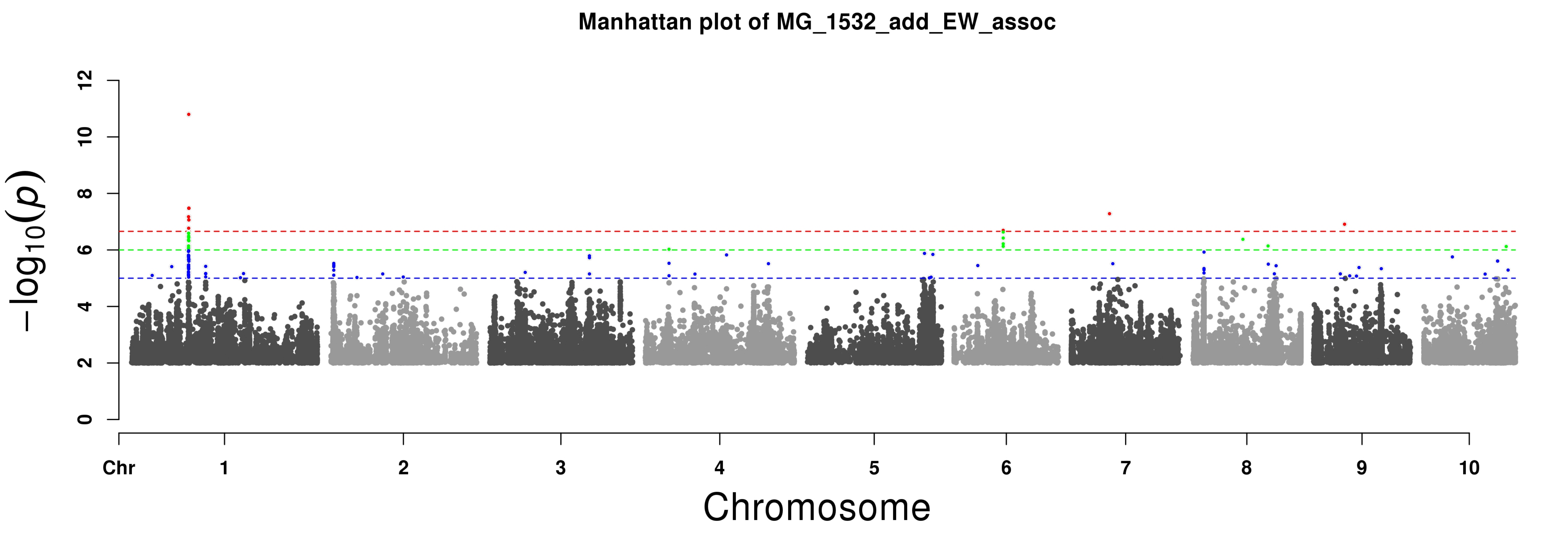
Manhattan plot of MG_1532_add_EW_assoc
$-\log_{10}(p)$
0
2
4
6
8
10
12
Chr
1
2
3
4
5
6
7
8
9
10
Chromosome

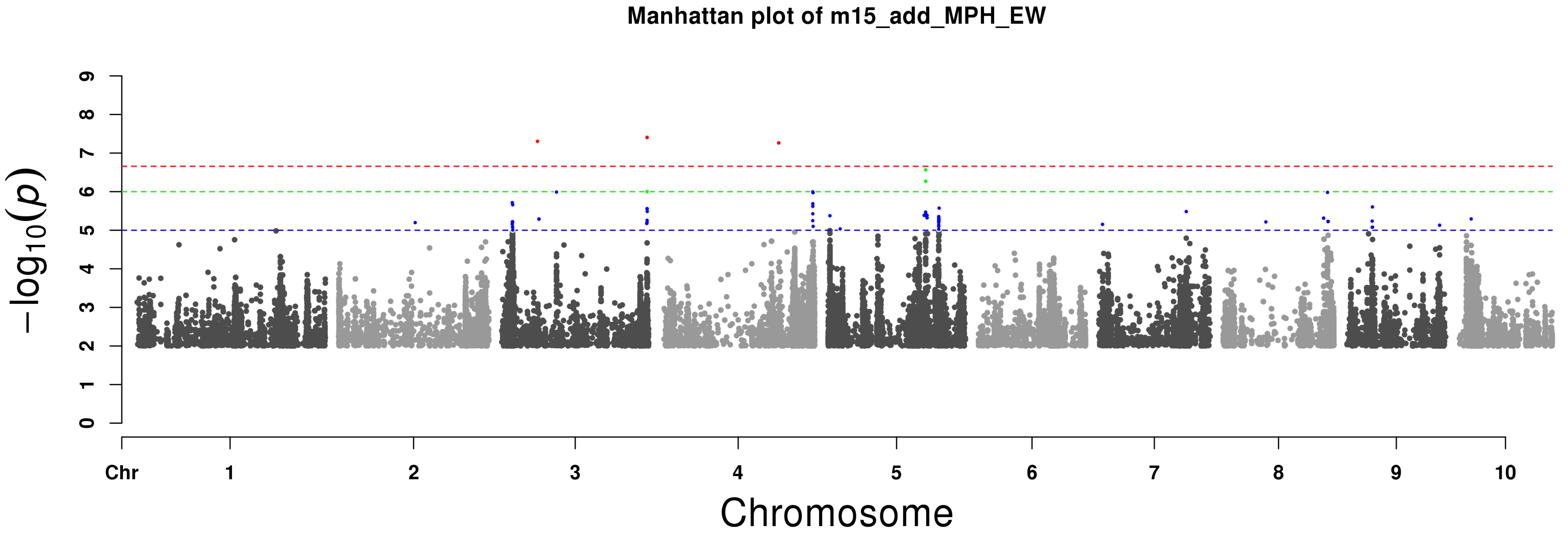

Manhattan plot of m15_add_MPH_EW
−log10(p)
0
1
2
3
4
5
6
7
8
9
Chr
1
2
3
4
5
6
7
8
9
10
Chromosome

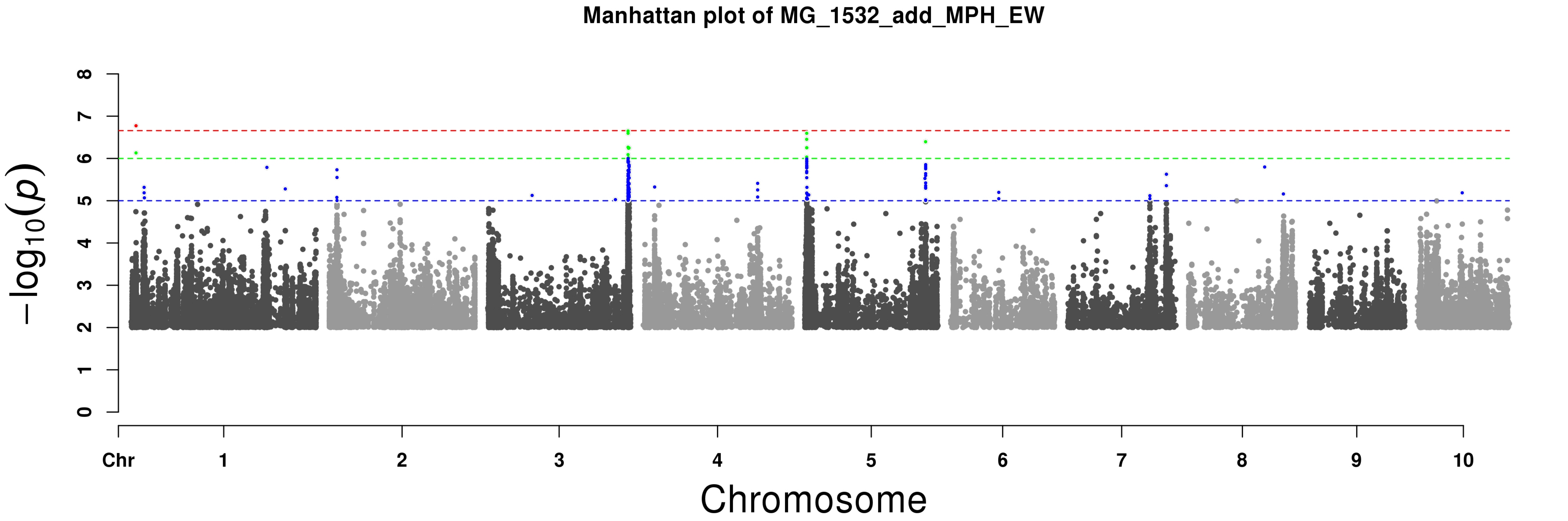

Manhattan plot of MG_1532_add_MPH_EW
$-\log_{10}(p)$
0
1
2
3
4
5
6
7
8
Chr
1
2
3
4
5
6
7
8
9
10
Chromosome

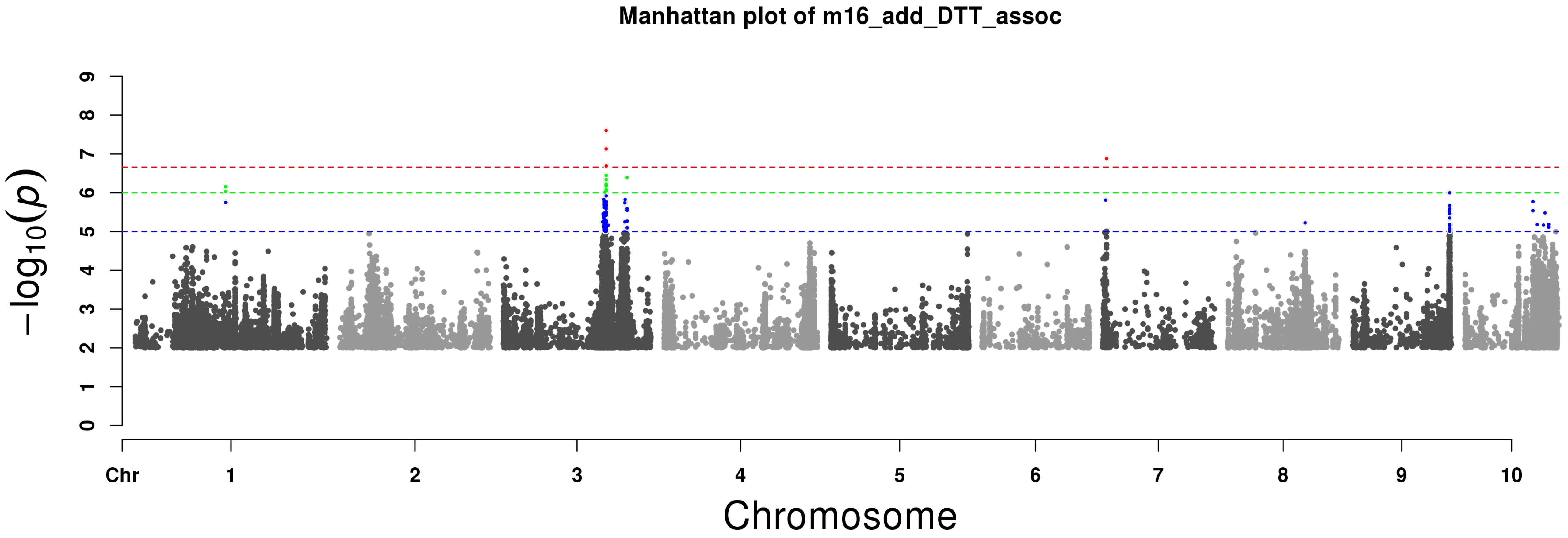

Manhattan plot of m16_add_DTT_assoc
$-\log_{10}(p)$
0
1
2
3
4
5
6
7
8
9
Chr
1
2
3
4
5
6
7
8
9
10
Chromosome

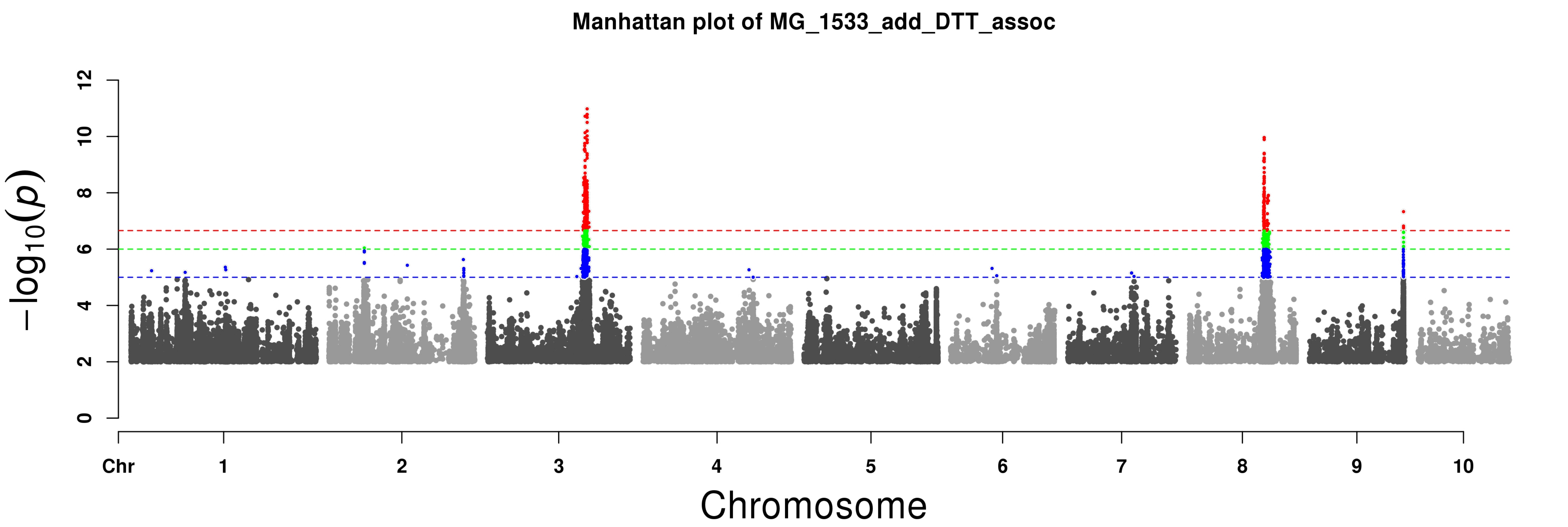

Manhattan plot of MG_1533_add_DTT_assoc
$-\log_{10}(p)$
0
2
4
6
8
10
12
Chr
1
2
3
4
5
6
7
8
9
10
Chromosome

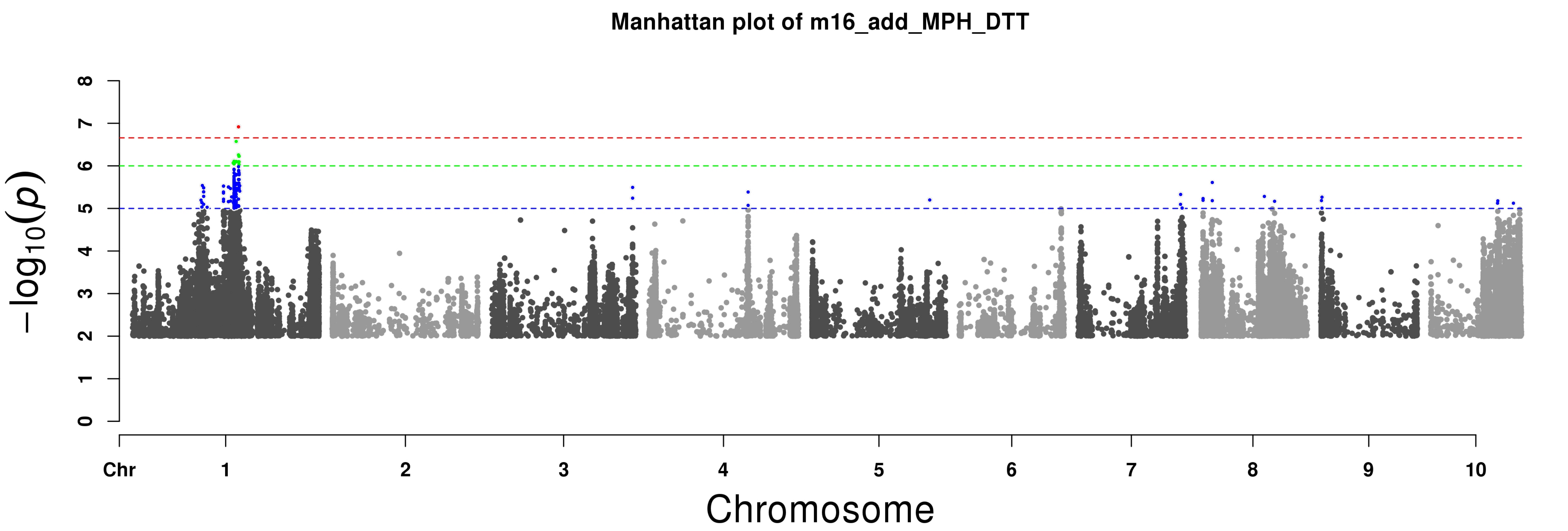

Manhattan plot of m16_add_MPH_DTT
$-\log_{10}(p)$
0
1
2
3
4
5
6
7
8
Chr
1
2
3
4
5
6
7
8
9
10
Chromosome

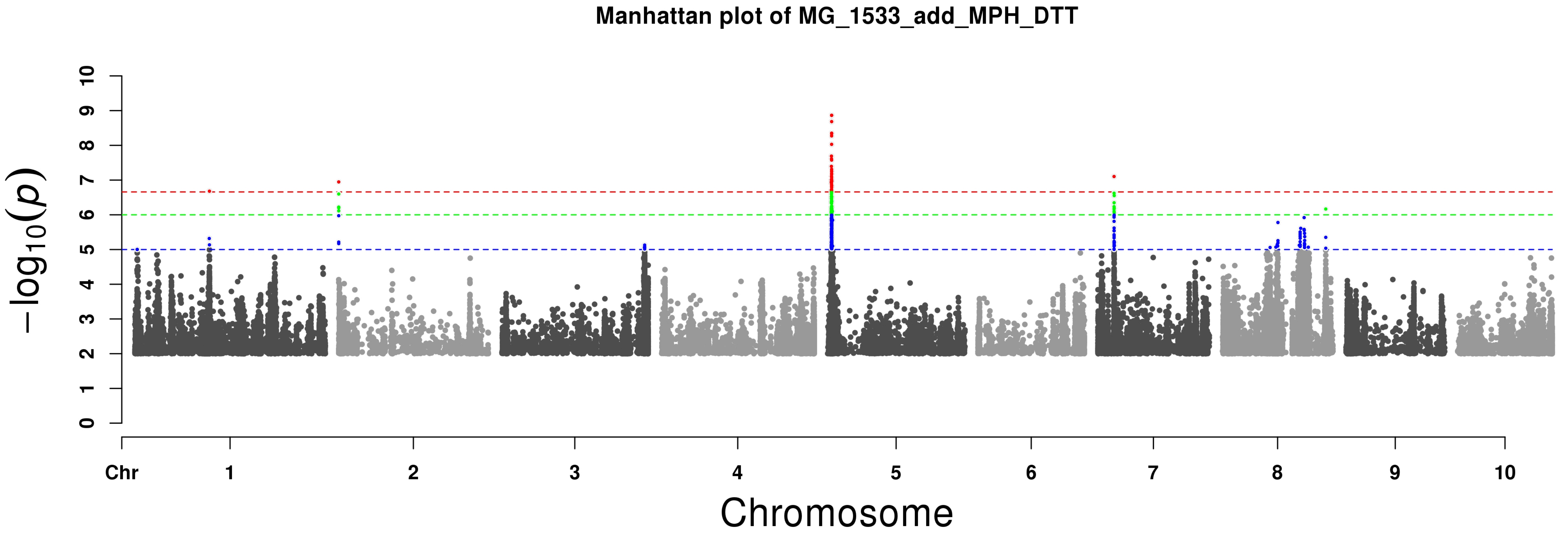

Manhattan plot of MG_1533_add_MPH_DTT
$-\log_{10}(p)$
0
1
2
3
4
5
6
7
8
9
10
Chr
1
2
3
4
5
6
7
8
9
10
Chromosome

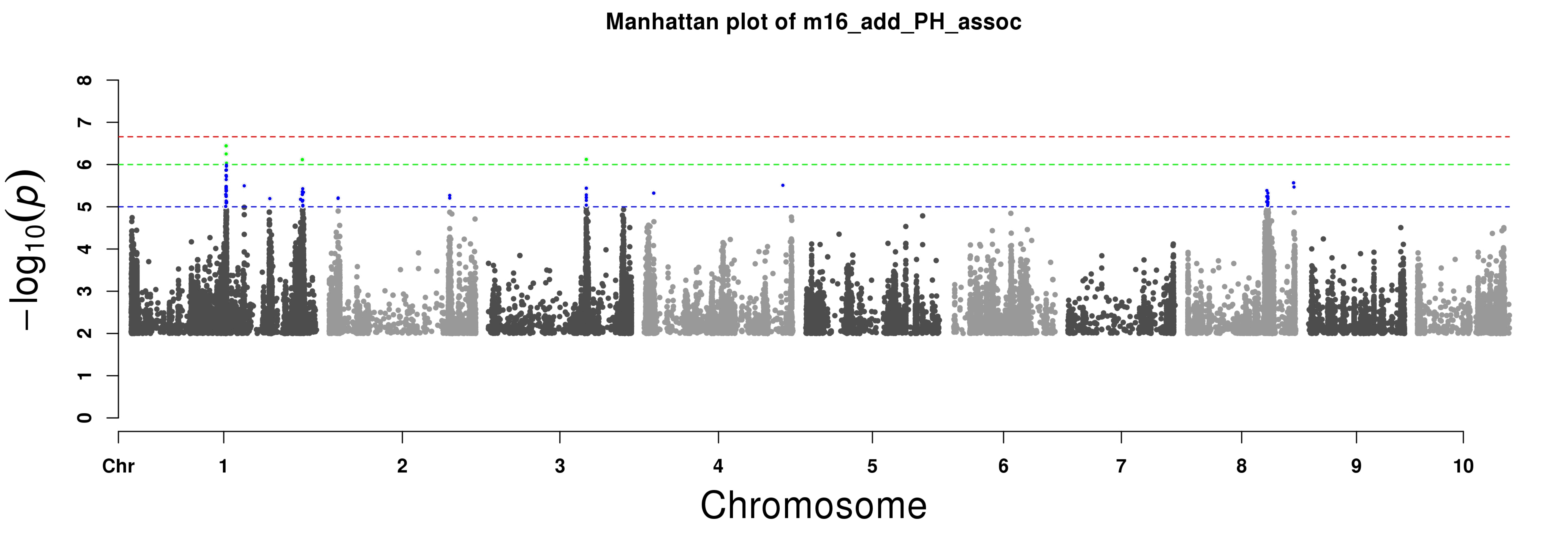
Manhattan plot of m16_add_PH_assoc
$-\log_{10}(p)$
0
1
2
3
4
5
6
7
8
Chr
1
2
3
4
5
6
7
8
9
10
Chromosome

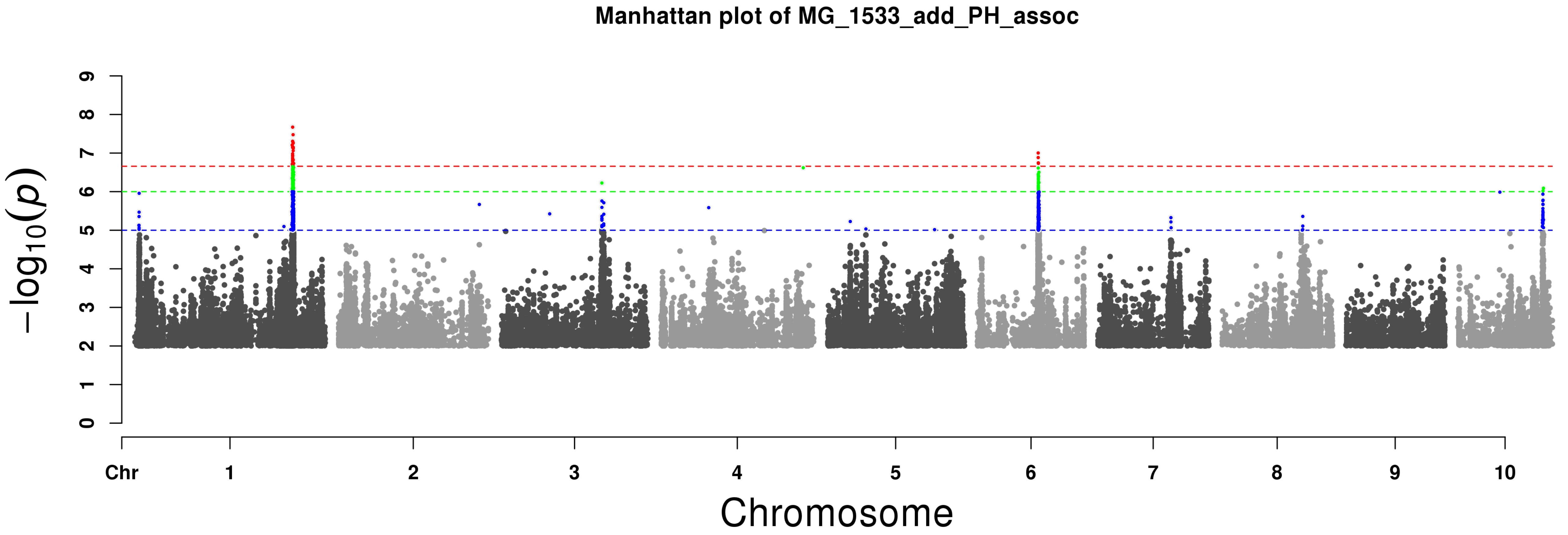

Manhattan plot of MG_1533_add_PH_assoc
$-\log_{10}(p)$
0
1
2
3
4
5
6
7
8
9
Chr
1
2
3
4
5
6
7
8
9
10
Chromosome

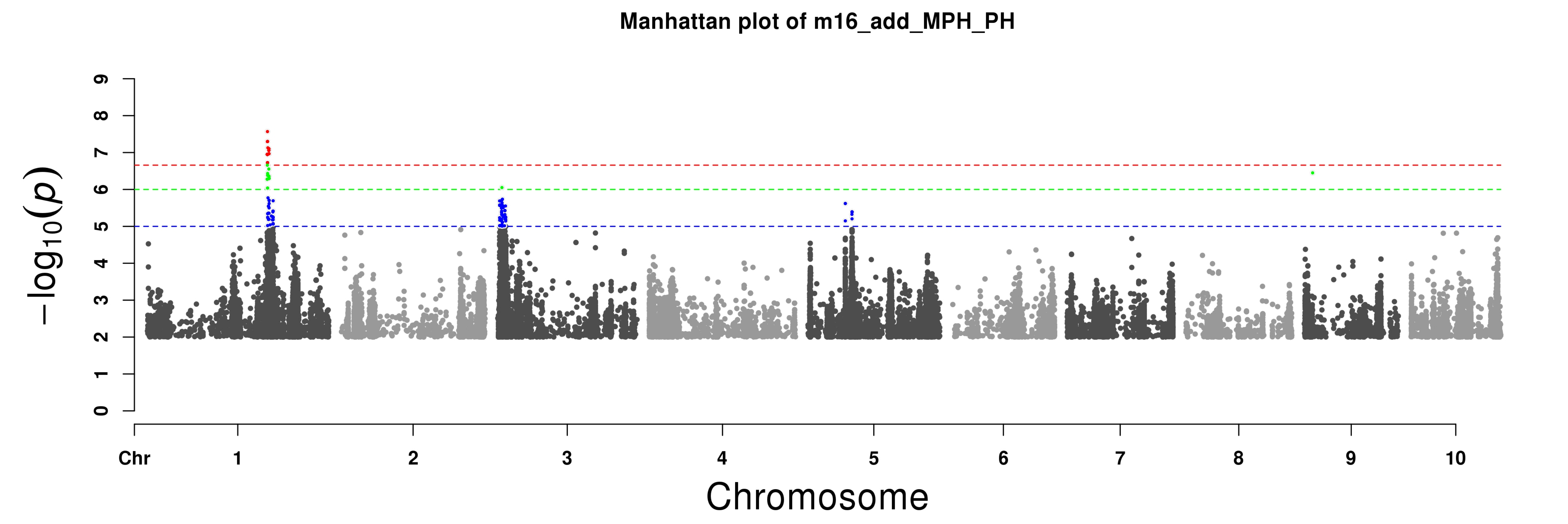

Manhattan plot of m16_add_MPH_PH
$-\log_{10}(p)$
0
1
2
3
4
5
6
7
8
9
Chr
1
2
3
4
5
6
7
8
9
10
Chromosome

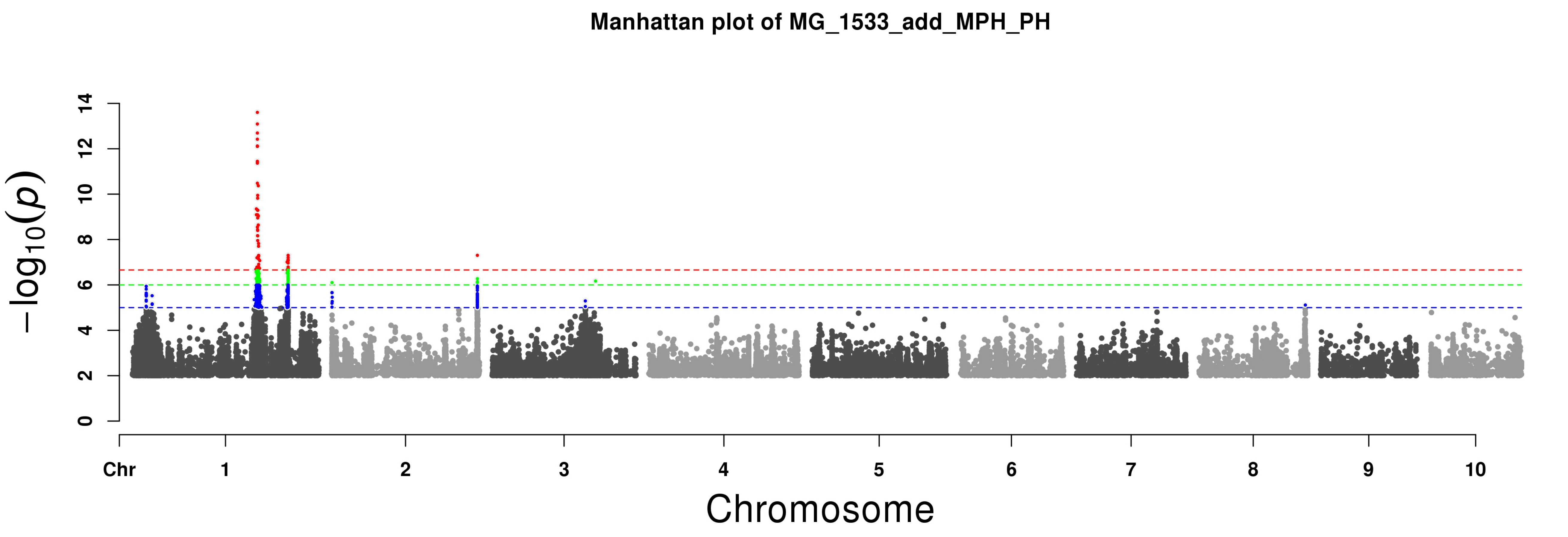
Manhattan plot of MG_1533_add_MPH_PH
−log10(p)
0
2
4
6
8
10
12
14
Chr
1
2
3
4
5
6
7
8
9
10
Chromosome

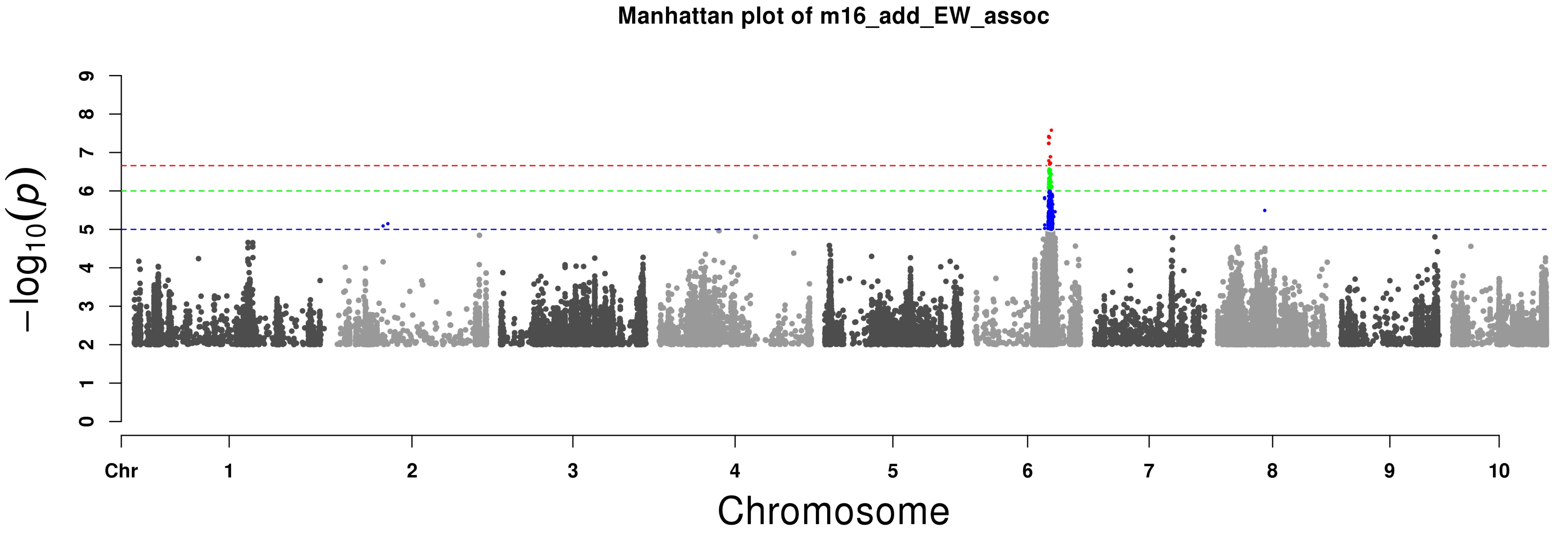

Manhattan plot of m16_add_EW_assoc
$-\log_{10}(p)$
0
1
2
3
4
5
6
7
8
9
Chr
1
2
3
4
5
6
7
8
9
10
Chromosome

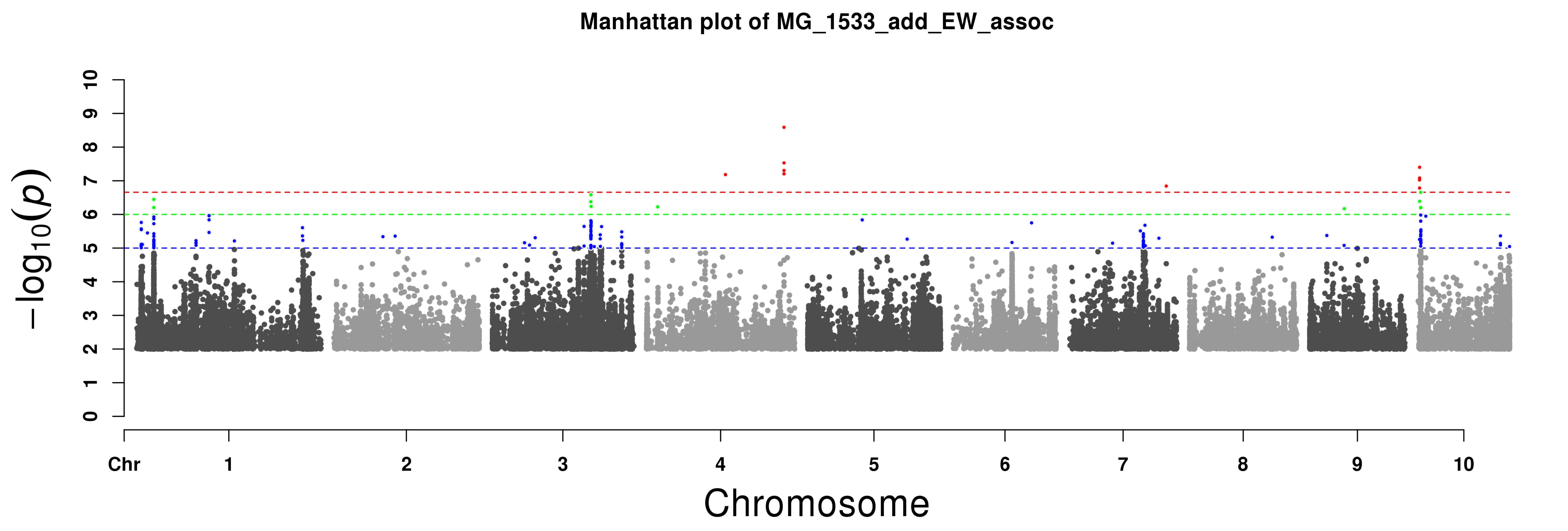

Manhattan plot of MG_1533_add_EW_assoc
$-\log_{10}(p)$
0
1
2
3
4
5
6
7
8
9
10
Chr
1
2
3
4
5
6
7
8
9
10
Chromosome

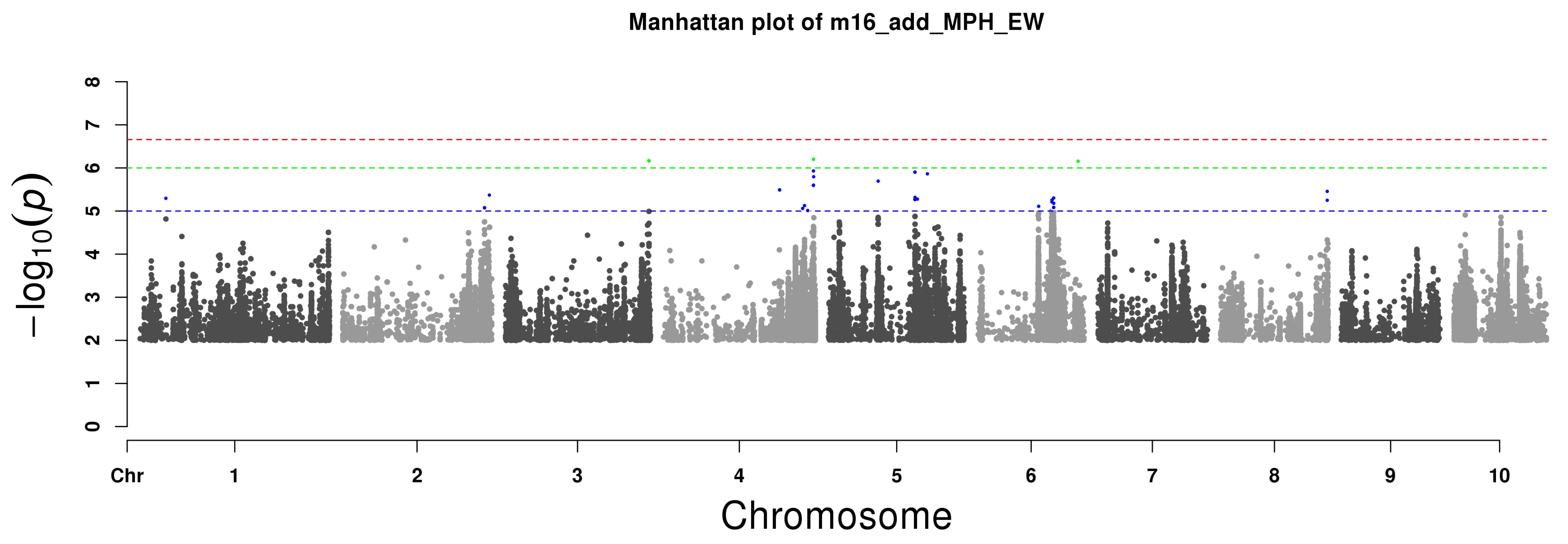

Manhattan plot of m16_add_MPH_EW
$-\log_{10}(p)$
0
1
2
3
4
5
6
7
8
Chr
1
2
3
4
5
6
7
8
9
10
Chromosome

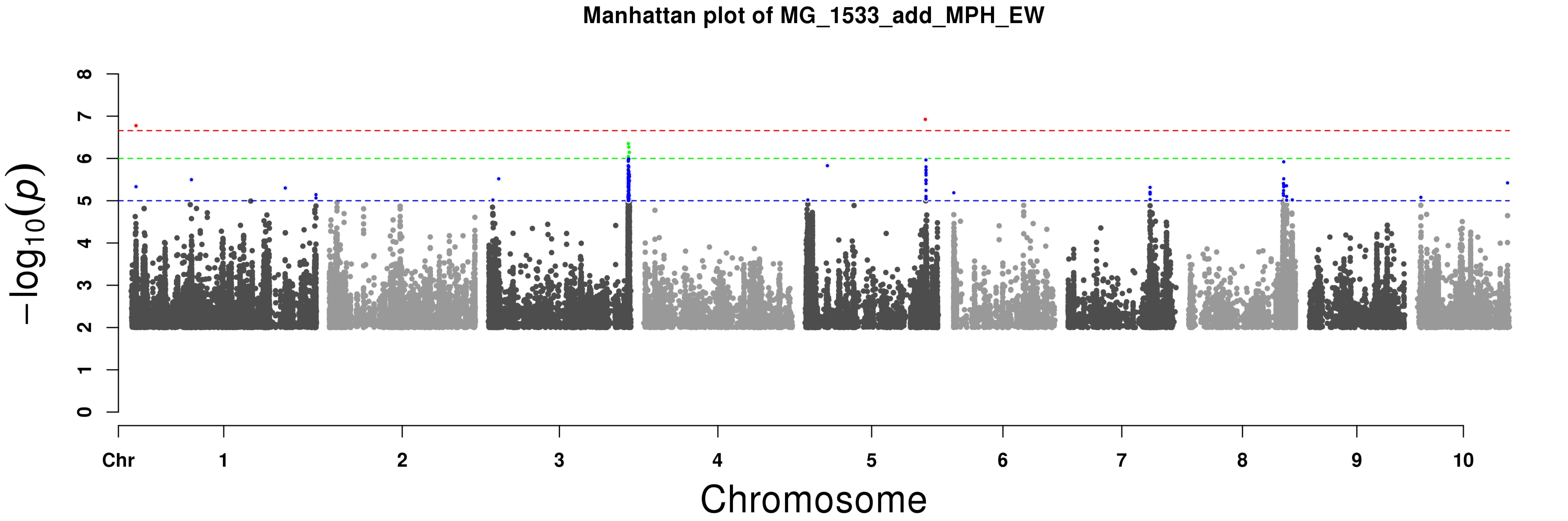

Manhattan plot of MG_1533_add_MPH_EW
$-\log_{10}(p)$
0
1
2
3
4
5
6
7
8
Chr
1
2
3
4
5
6
7
8
9
10
Chromosome

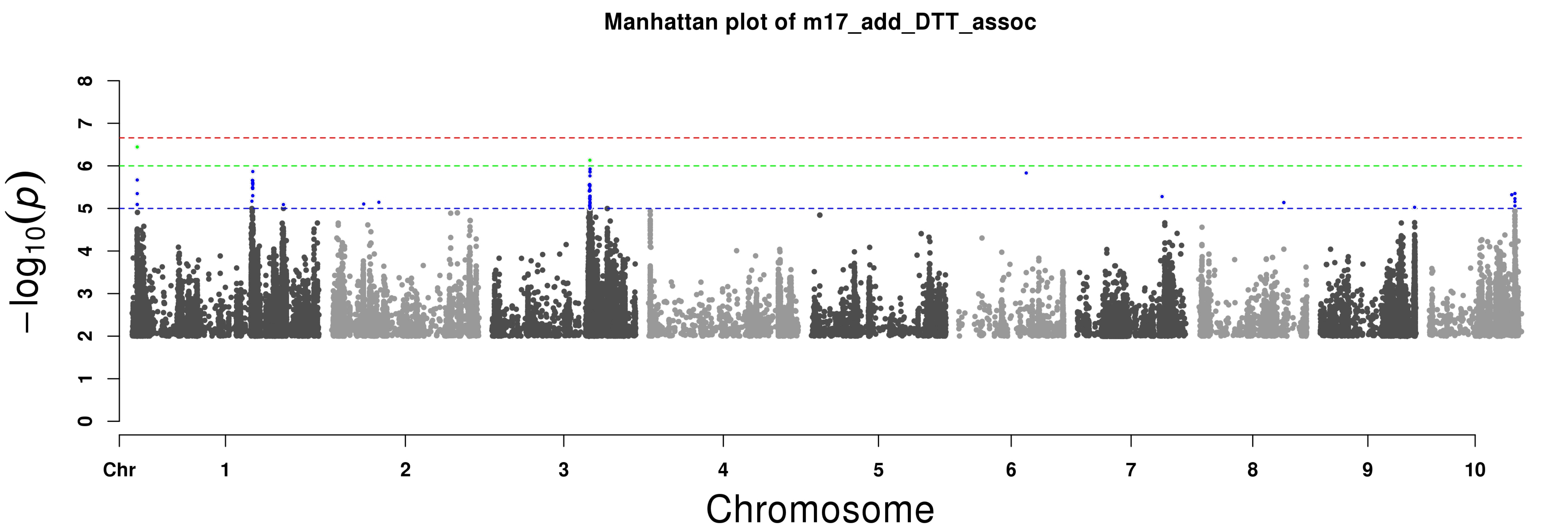

Manhattan plot of m17_add_DTT_assoc
$-\log_{10}(p)$
0
1
2
3
4
5
6
7
8
Chr
1
2
3
4
5
6
7
8
9
10
Chromosome

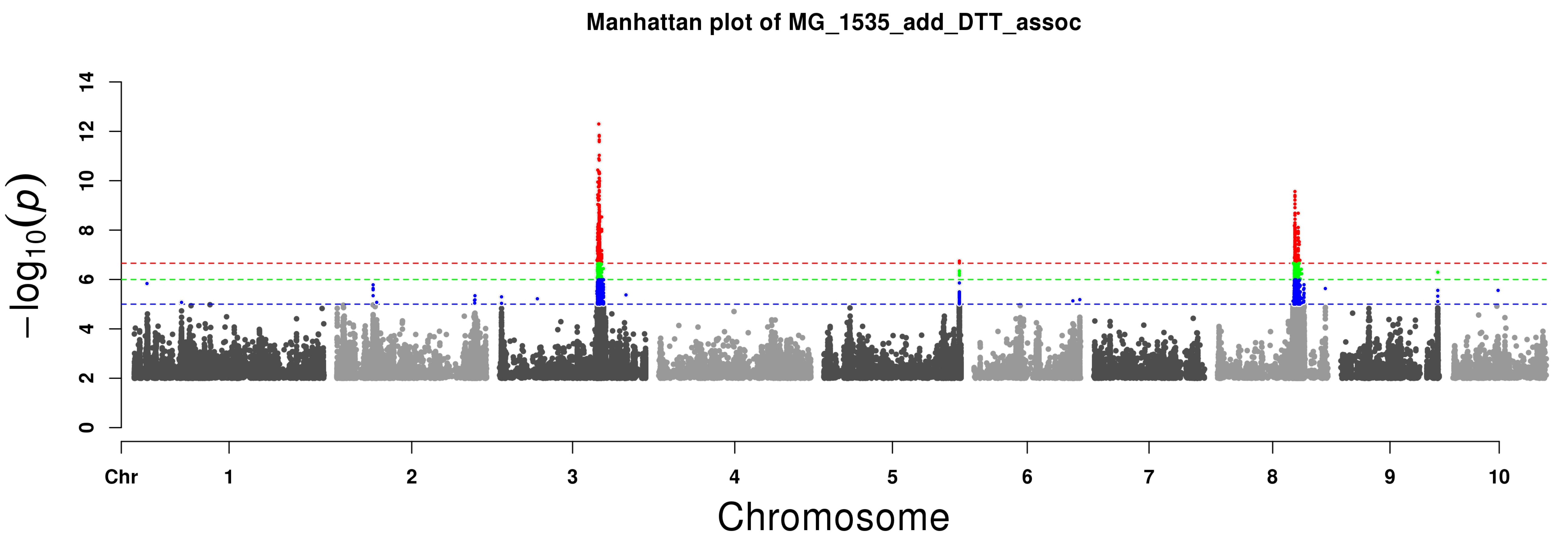

Manhattan plot of MG_1535_add_DTT_assoc
−log10(p)
0
2
4
6
8
10
12
14
Chr
1
2
3
4
5
6
7
8
9
10
Chromosome

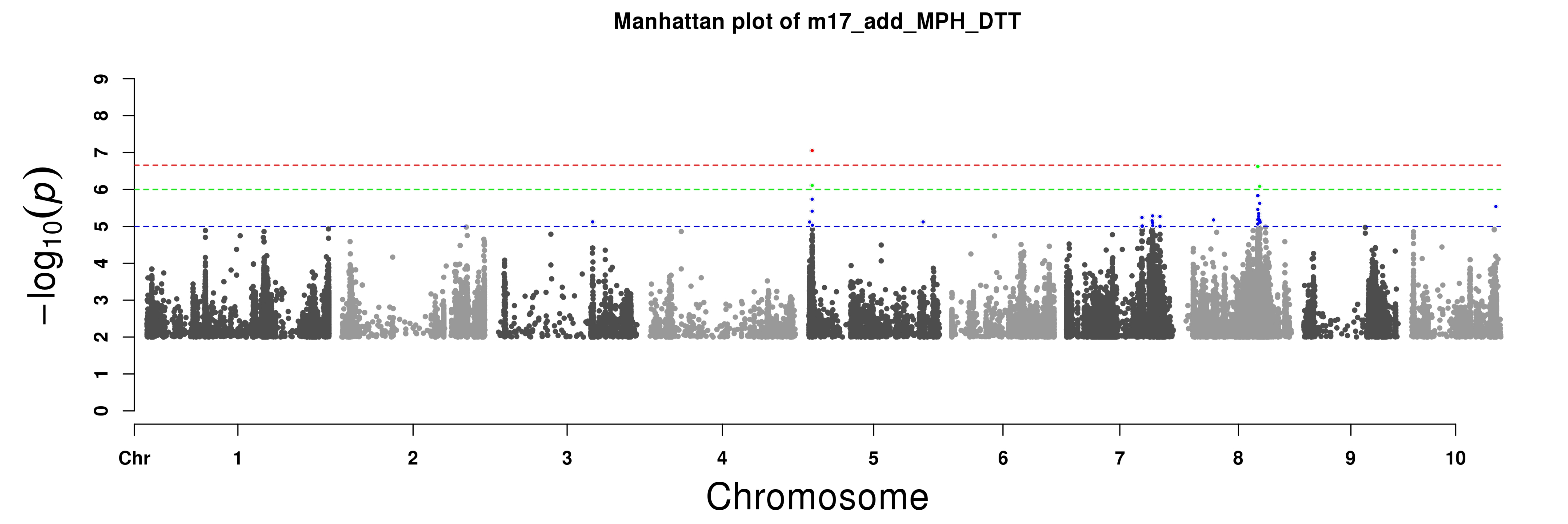

Manhattan plot of m17_add_MPH_DTT
$-\log_{10}(p)$
0
1
2
3
4
5
6
7
8
9
Chr
1
2
3
4
5
6
7
8
9
10
Chromosome

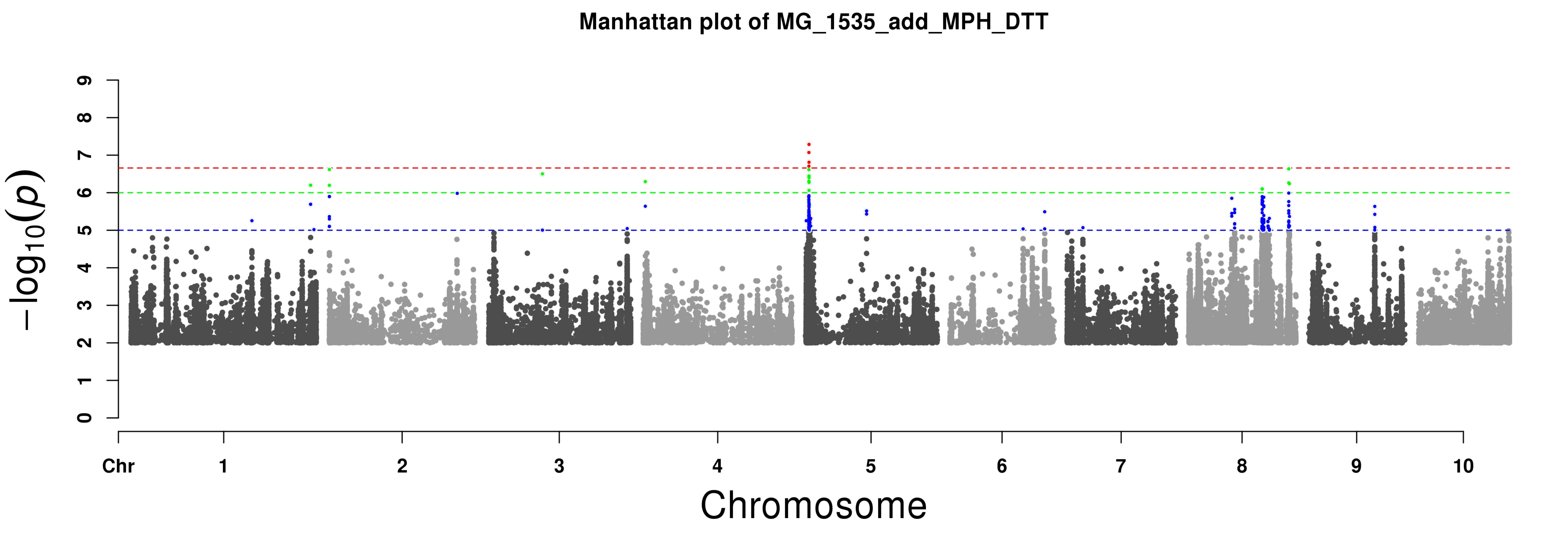

Manhattan plot of MG_1535_add_MPH_DTT
$-\log_{10}(p)$
0
1
2
3
4
5
6
7
8
9
Chr
1
2
3
4
5
6
7
8
9
10
Chromosome

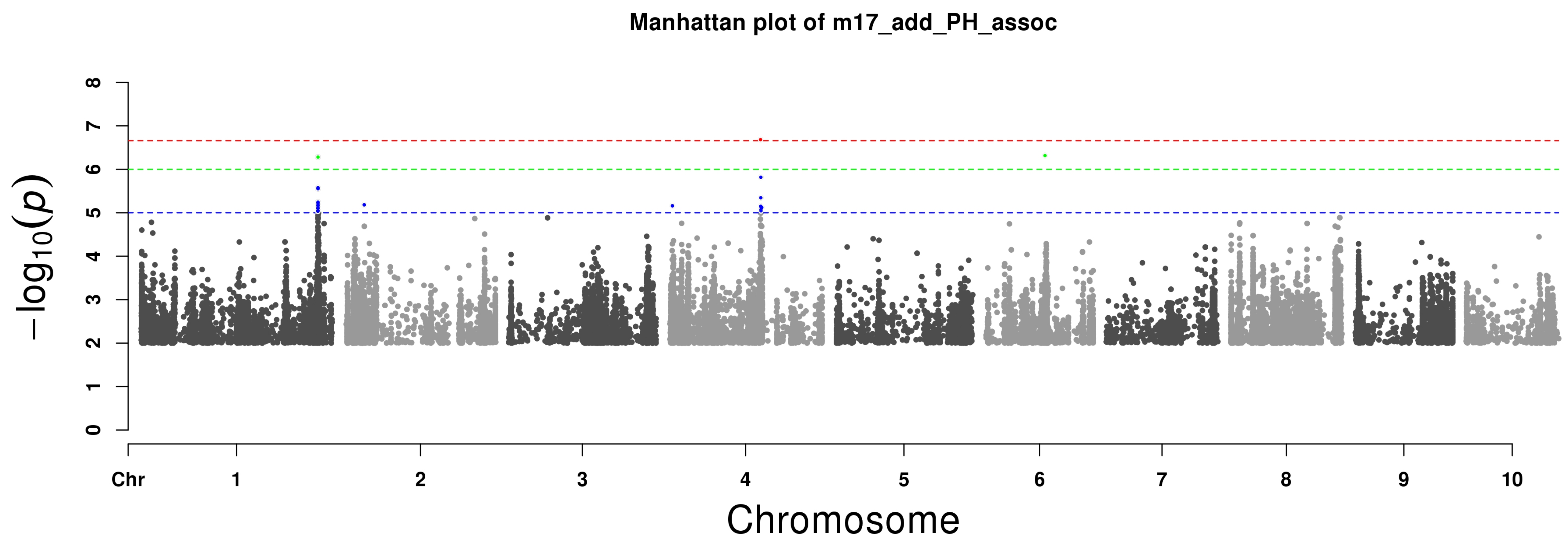

Manhattan plot of m17_add_PH_assoc
$-\log_{10}(p)$
0
1
2
3
4
5
6
7
8
Chr
1
2
3
4
5
6
7
8
9
10
Chromosome

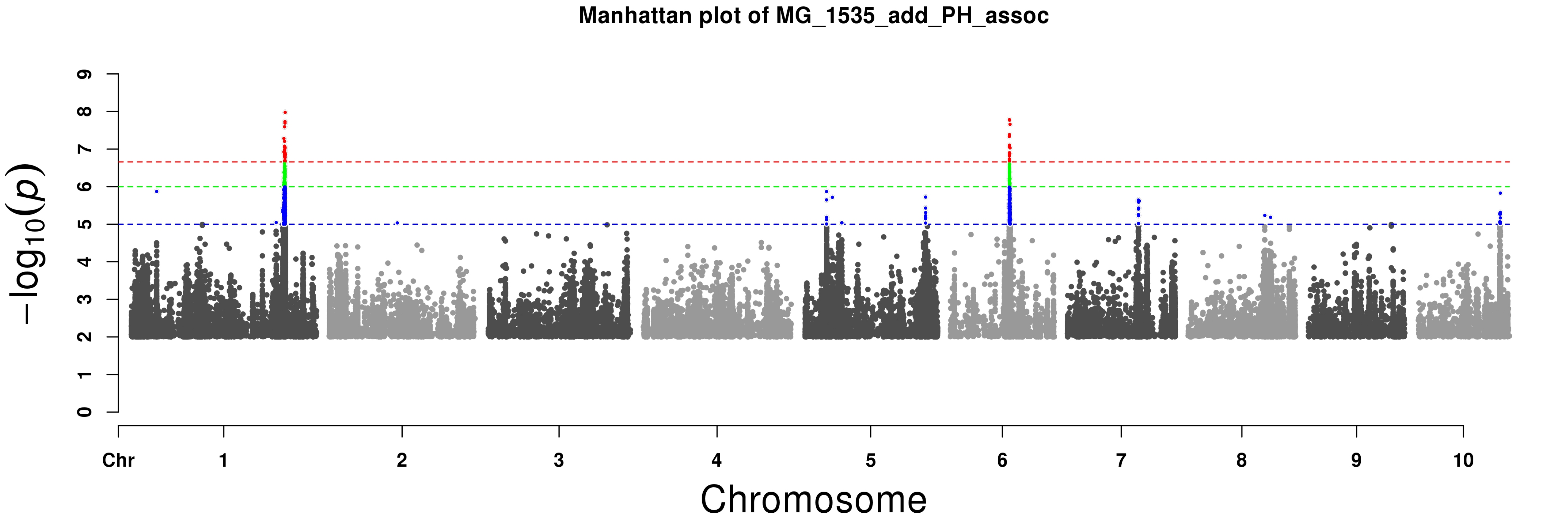

Manhattan plot of MG_1535_add_PH_assoc
$-\log_{10}(p)$
0
1
2
3
4
5
6
7
8
9
Chr
1
2
3
4
5
6
7
8
9
10
Chromosome

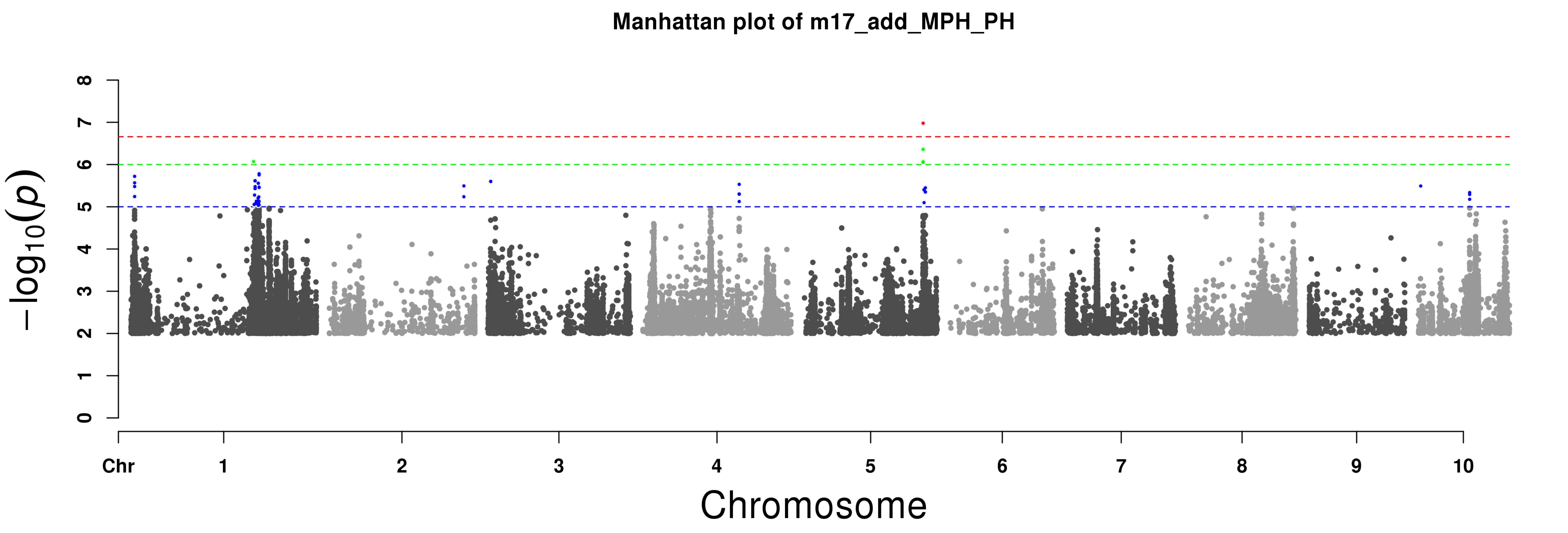
Manhattan plot of m17_add_MPH_PH
$-\log_{10}(p)$
0
1
2
3
4
5
6
7
8
Chr
1
2
3
4
5
6
7
8
9
10
Chromosome

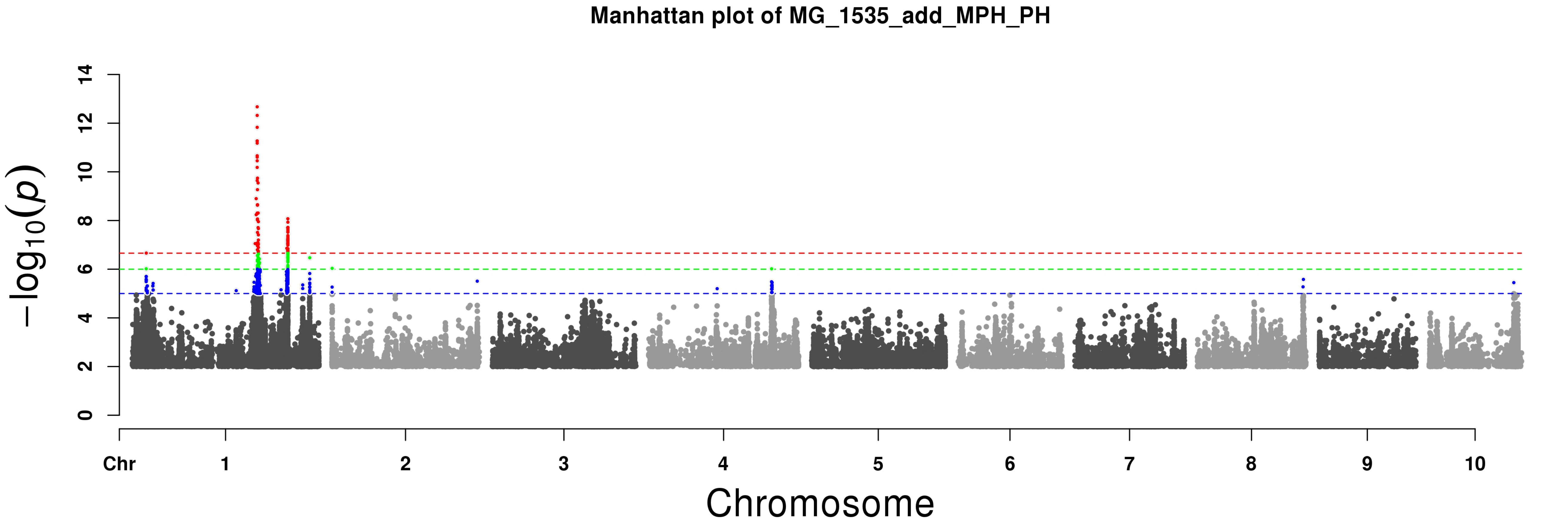

Manhattan plot of MG_1535_add_MPH_PH
$-\log_{10}(p)$
0
2
4
6
8
10
12
14
Chr
1
2
3
4
5
6
7
8
9
10
Chromosome

Manhattan plot of m17_add_EW_assoc

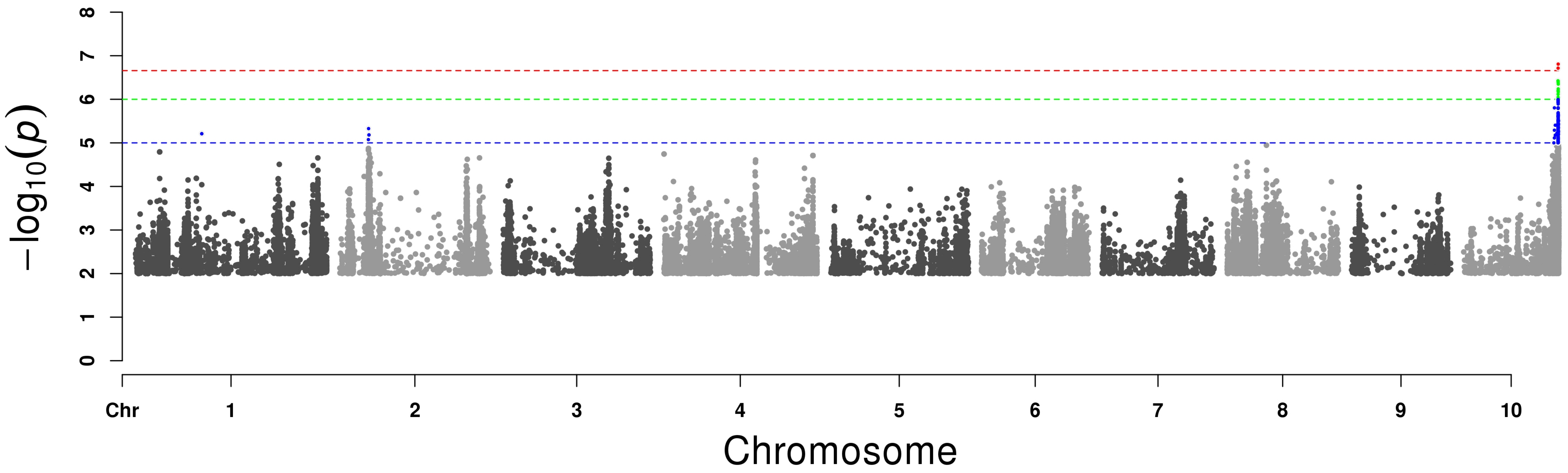

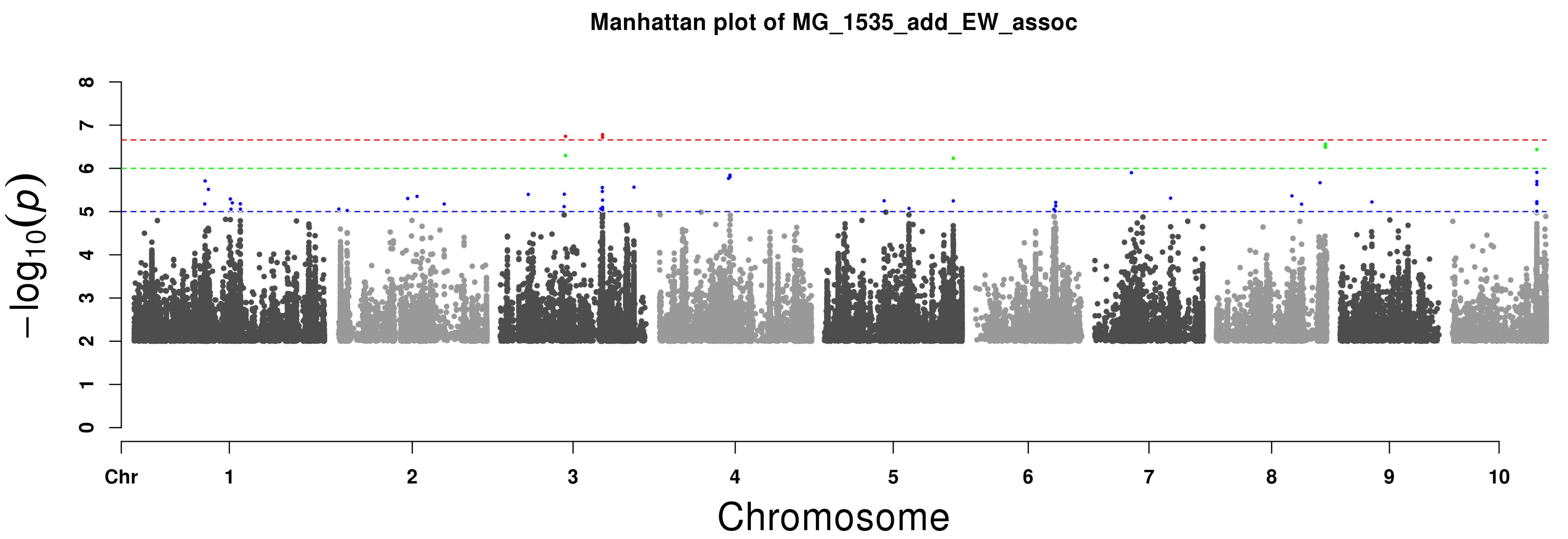

Manhattan plot of MG_1535_add_EW_assoc
0
1
2
3
4
5
6
7
8
$-\log_{10}(p)$
Chr
1
2
3
4
5
6
7
8
9
10
Chromosome

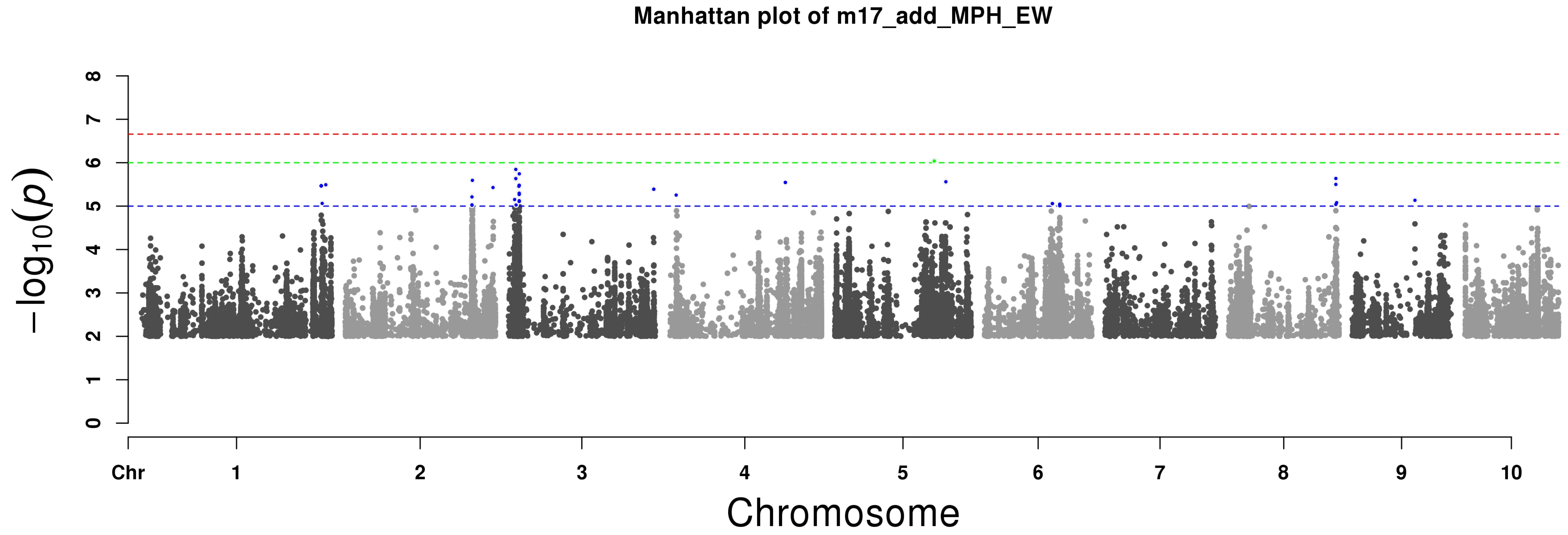

Manhattan plot of m17_add_MPH_EW
$-\log_{10}(p)$
0
1
2
3
4
5
6
7
8
Chr
1
2
3
4
5
6
7
8
9
10
Chromosome

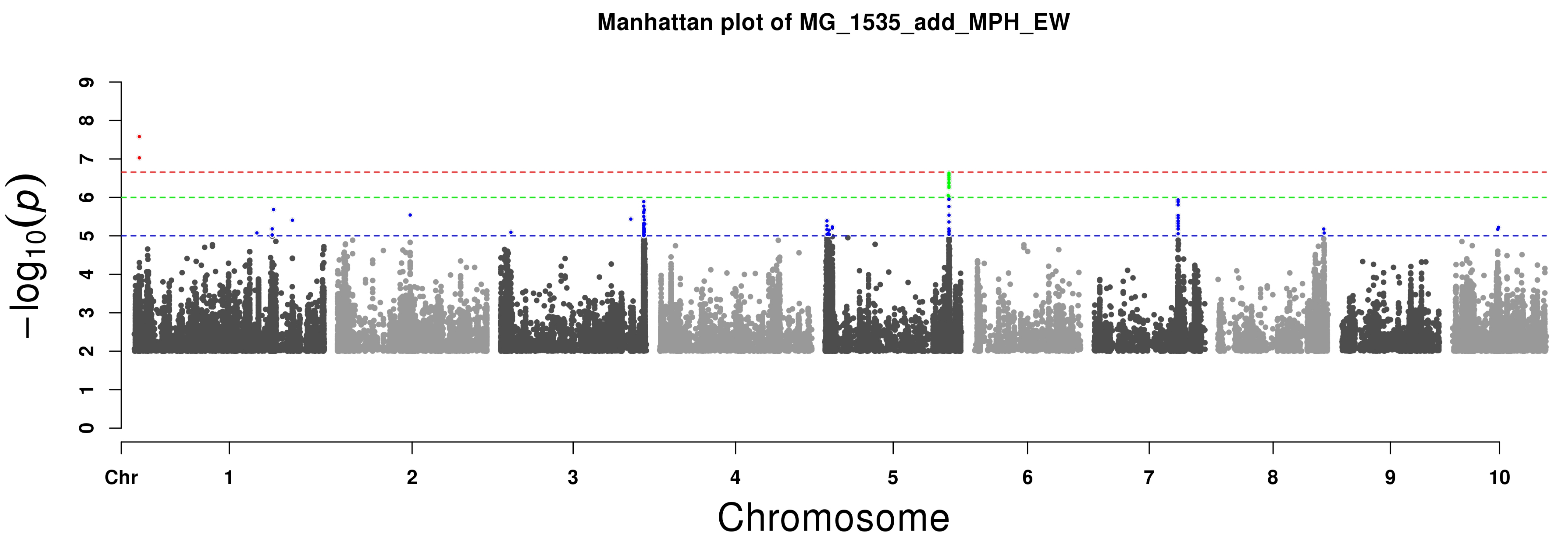

Manhattan plot of MG_1535_add_MPH_EW
$-\log_{10}(p)$
0
1
2
3
4
5
6
7
8
9
Chr
1
2
3
4
5
6
7
8
9
10
Chromosome

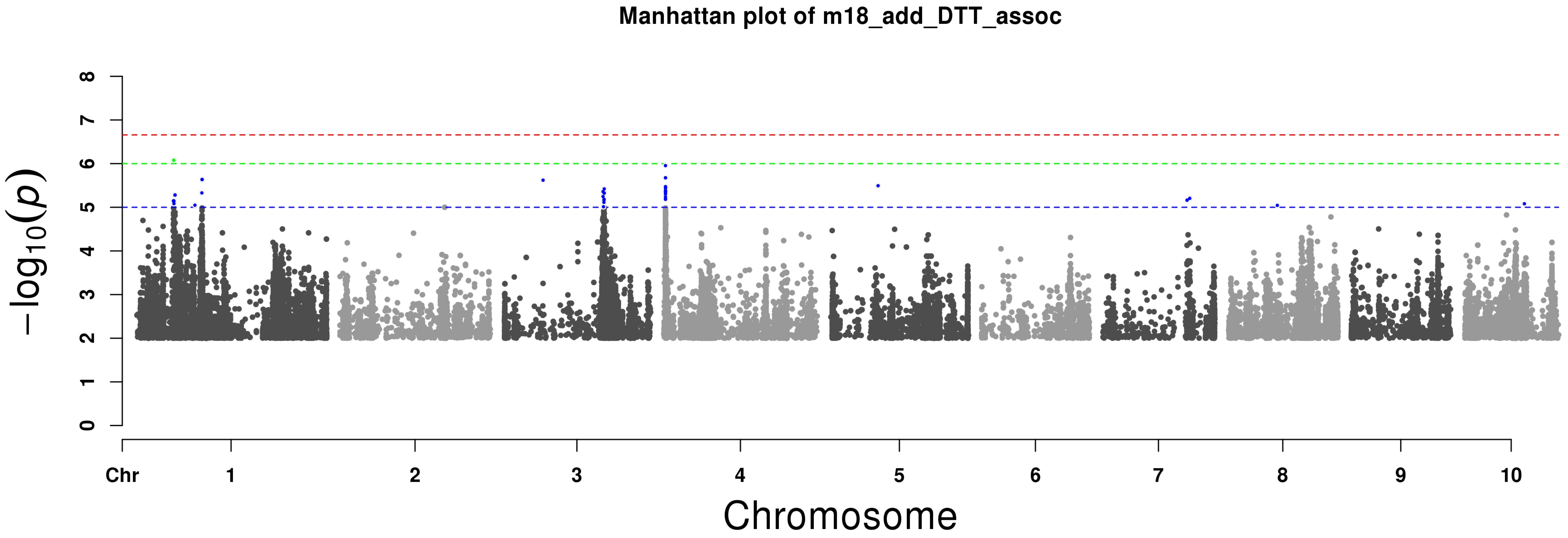

Manhattan plot of m18_add_DTT_assoc
$-\log_{10}(p)$
0
1
2
3
4
5
6
7
8
Chr
1
2
3
4
5
6
7
8
9
10
Chromosome

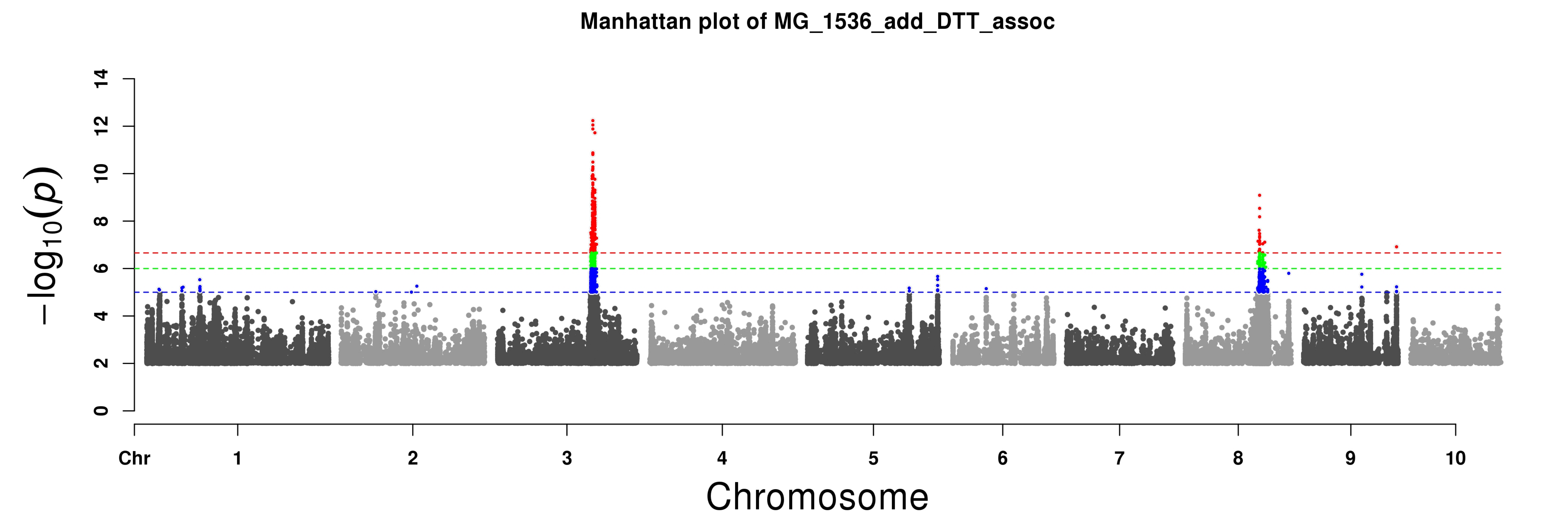
Manhattan plot of MG_1536_add_DTT_assoc
−log10(p)
0
2
4
6
8
10
12
14
Chr
1
2
3
4
5
6
7
8
9
10
Chromosome

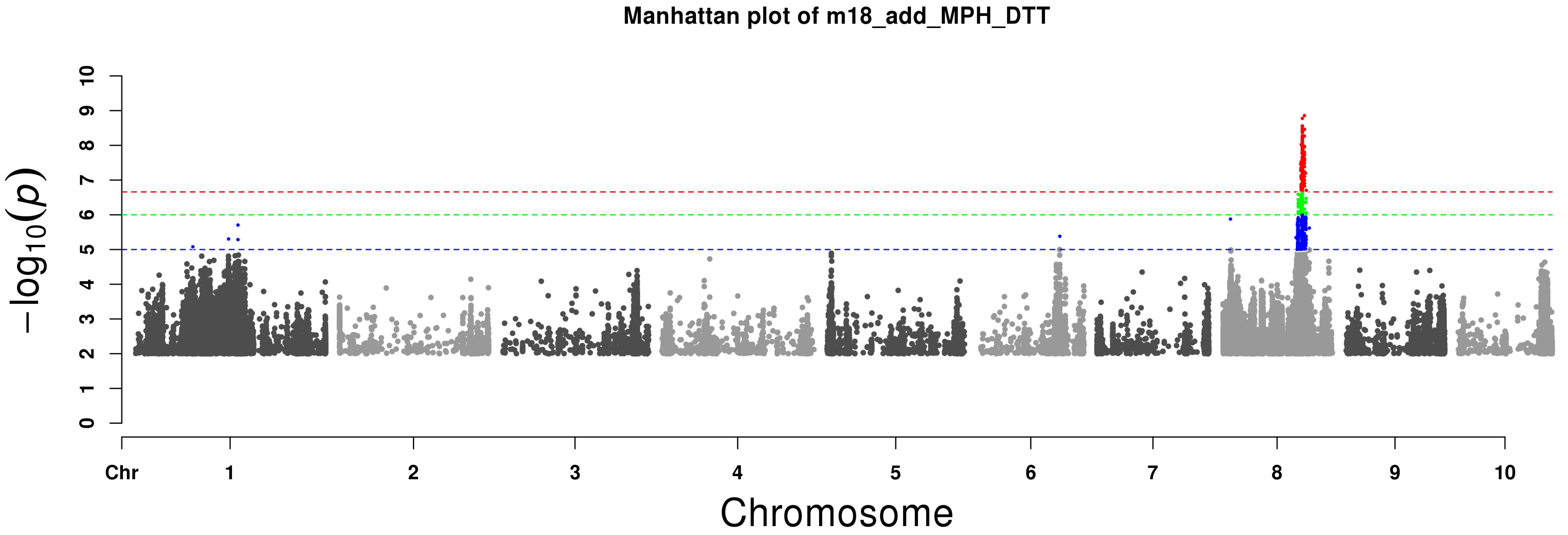

Manhattan plot of m18_add_MPH_DTT
$-\log_{10}(p)$
0
1
2
3
4
5
6
7
8
9
10
Chr
1
2
3
4
5
6
7
8
9
10
Chromosome

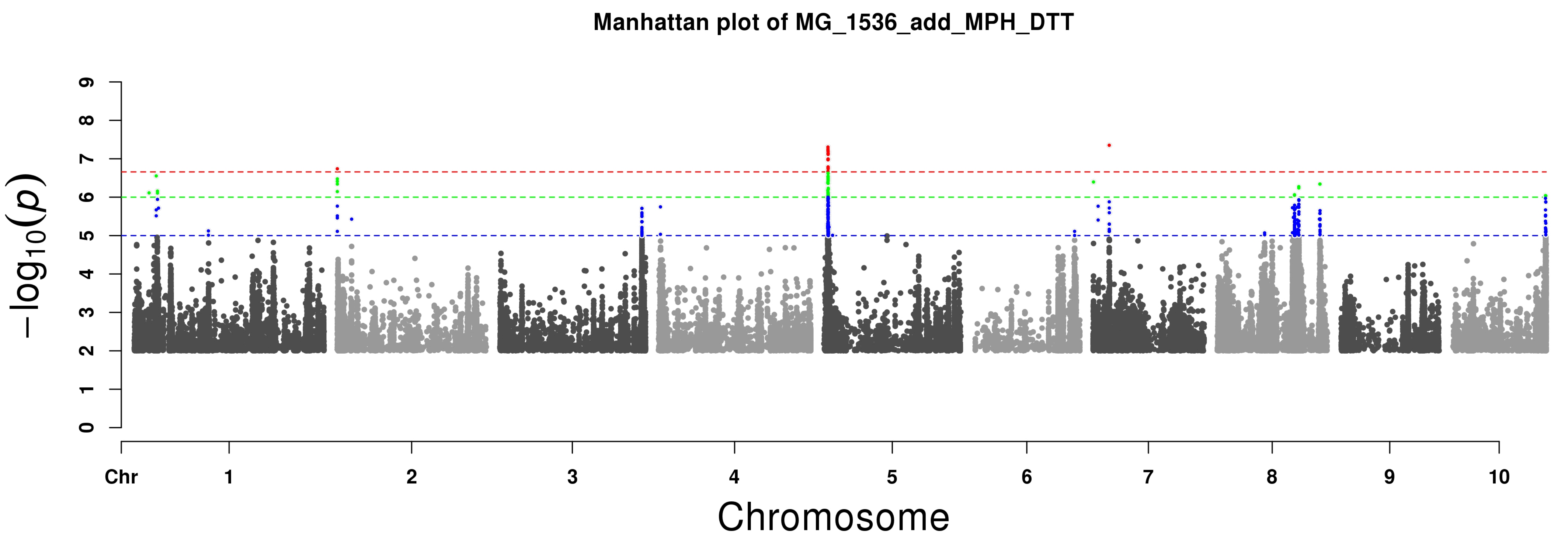

Manhattan plot of MG_1536_add_MPH_DTT
$-\log_{10}(p)$
0
1
2
3
4
5
6
7
8
9
Chr
1
2
3
4
5
6
7
8
9
10
Chromosome

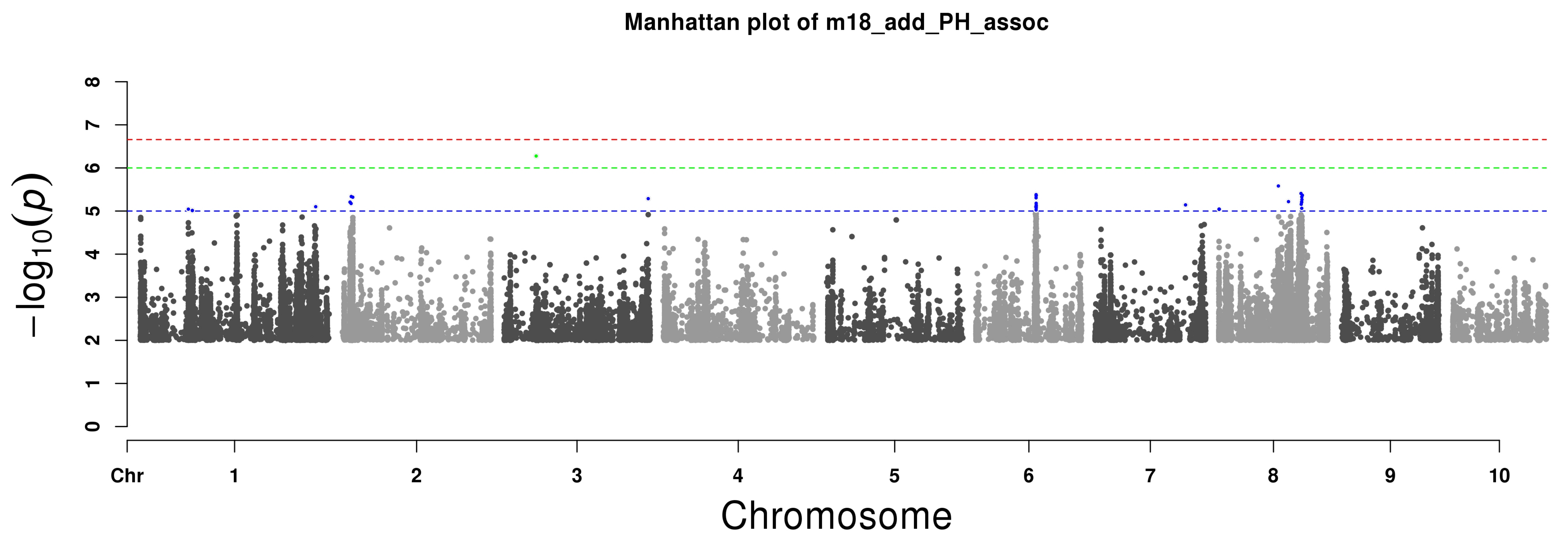
Manhattan plot of m18_add_PH_assoc
$-\log_{10}(p)$
0
1
2
3
4
5
6
7
8
Chr
1
2
3
4
5
6
7
8
9
10
Chromosome

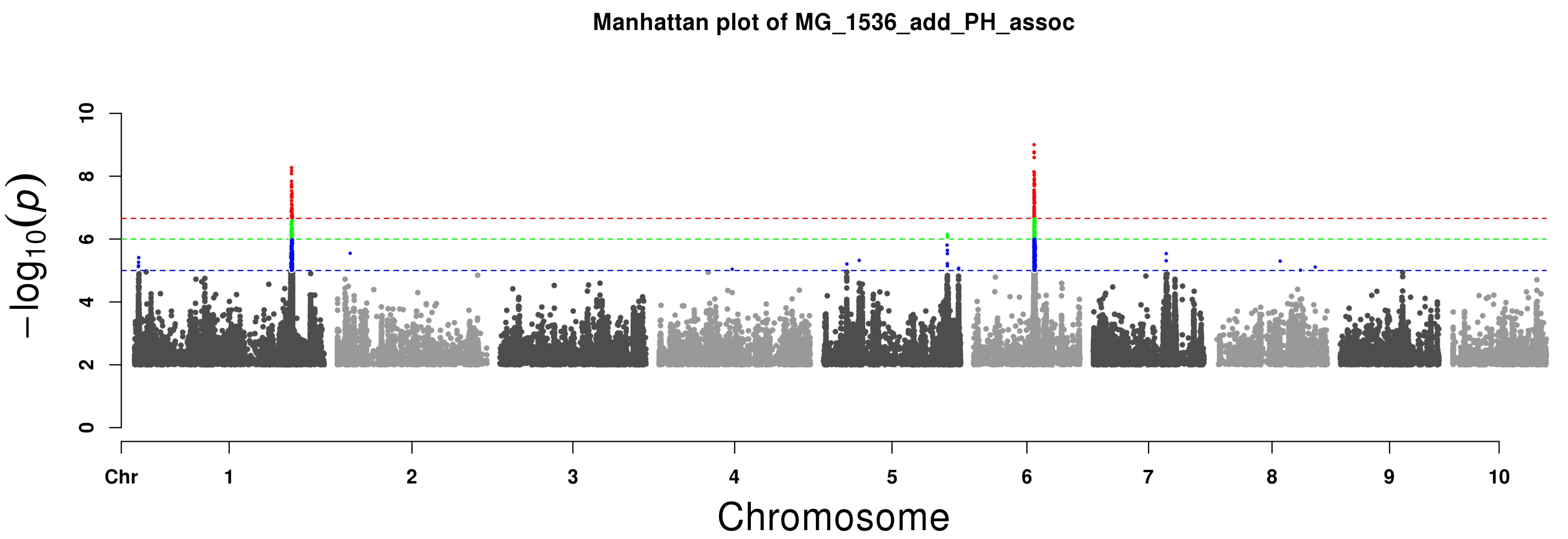

Manhattan plot of MG_1536_add_PH_assoc
$-\log_{10}(p)$
0
2
4
6
8
10
Chr
1
2
3
4
5
6
7
8
9
10
Chromosome

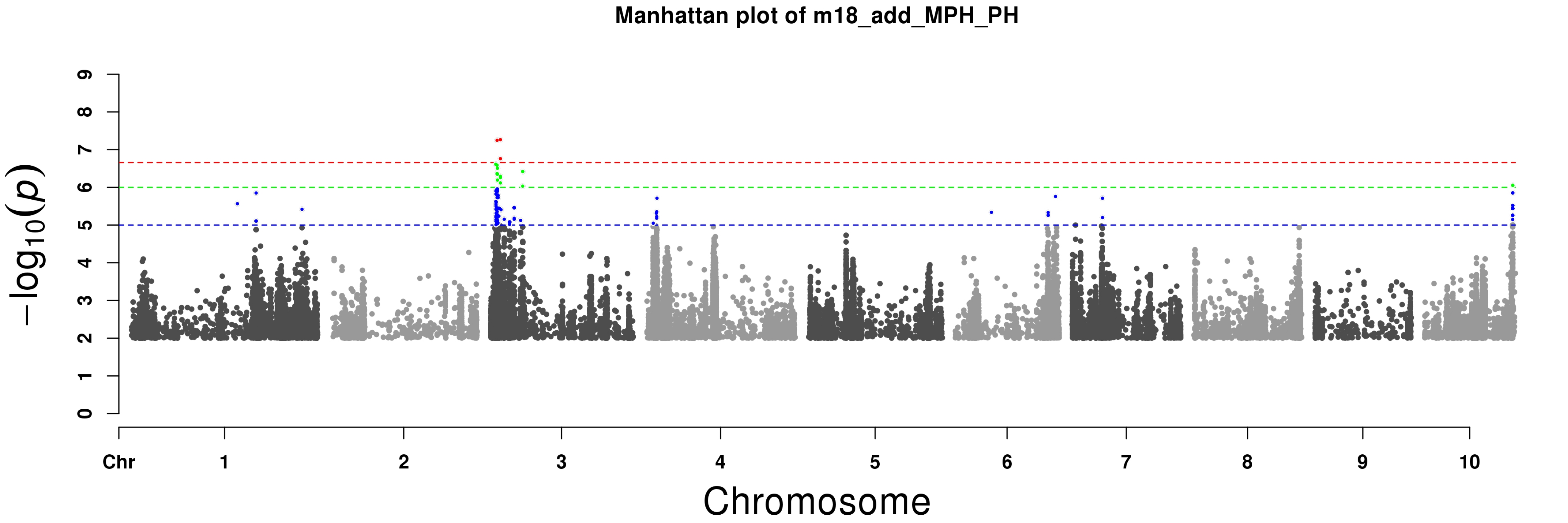

Manhattan plot of m18_add_MPH_PH
$-\log_{10}(p)$
0
1
2
3
4
5
6
7
8
9
Chr
1
2
3
4
5
6
7
8
9
10
Chromosome

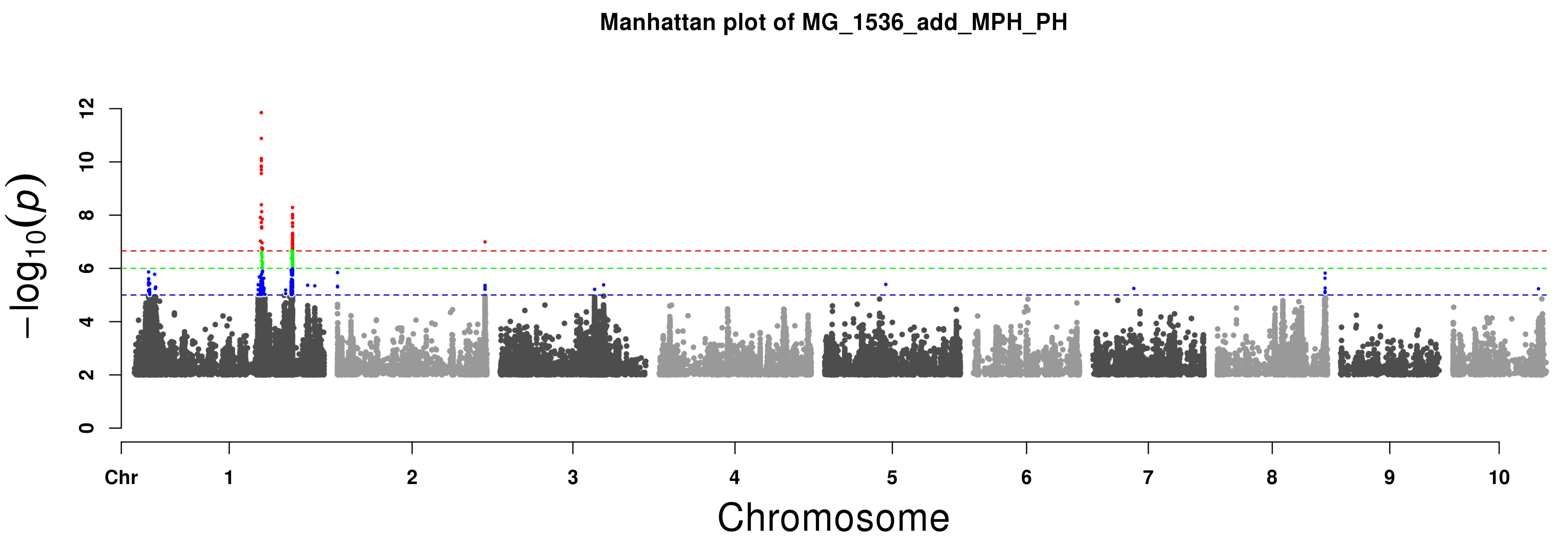

Manhattan plot of MG_1536_add_MPH_PH
$-\log_{10}(p)$
0
2
4
6
8
10
12
Chr
1
2
3
4
5
6
7
8
9
10
Chromosome

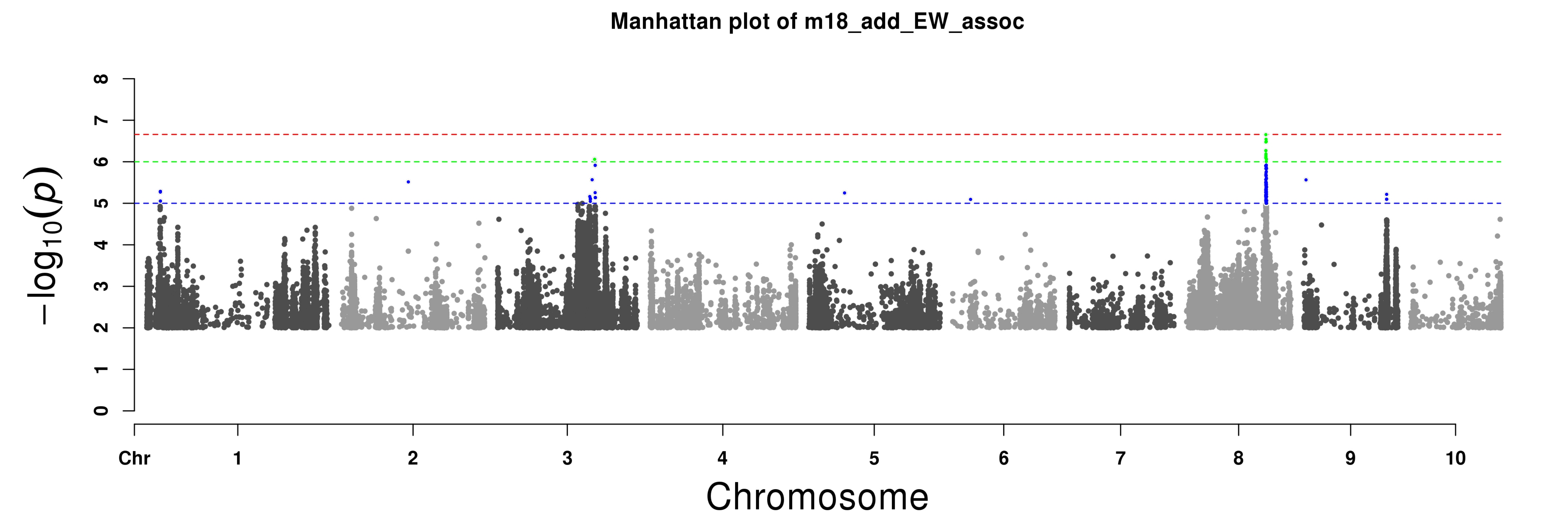
Manhattan plot of m18_add_EW_assoc
$-\log_{10}(p)$
0
1
2
3
4
5
6
7
8
Chr
1
2
3
4
5
6
7
8
9
10
Chromosome

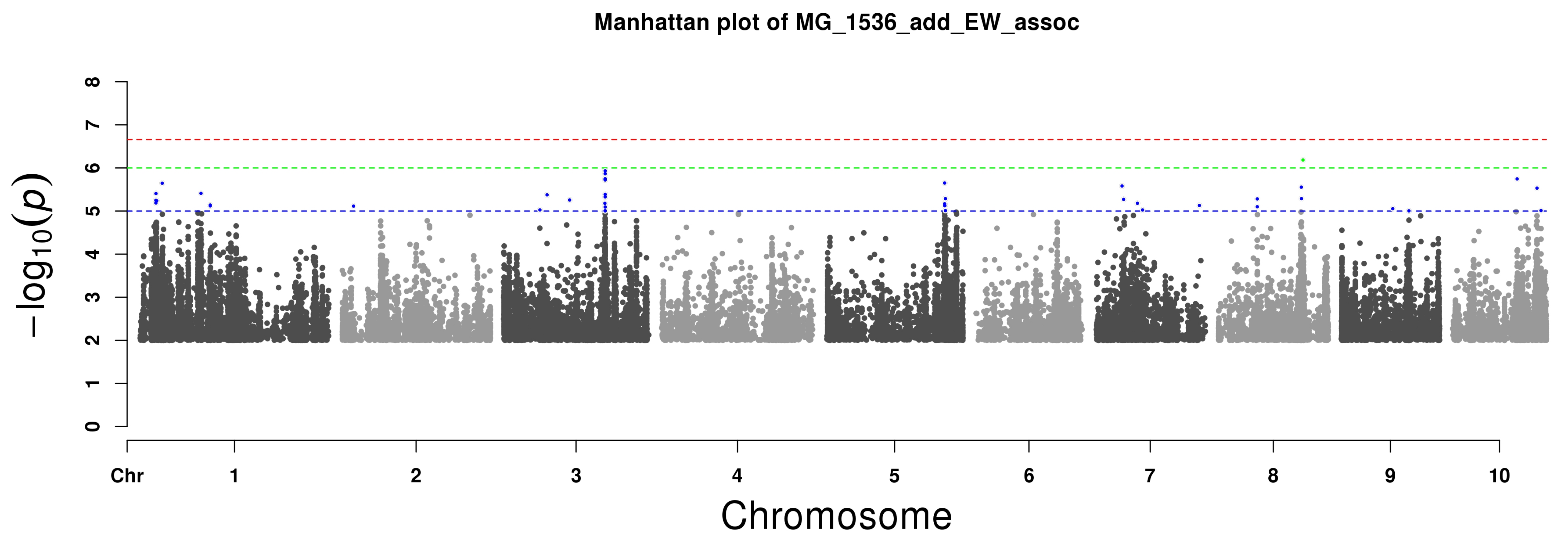
Manhattan plot of MG_1536_add_EW_assoc
−log10(p)
0
1
2
3
4
5
6
7
8
Chr
1
2
3
4
5
6
7
8
9
10
Chromosome

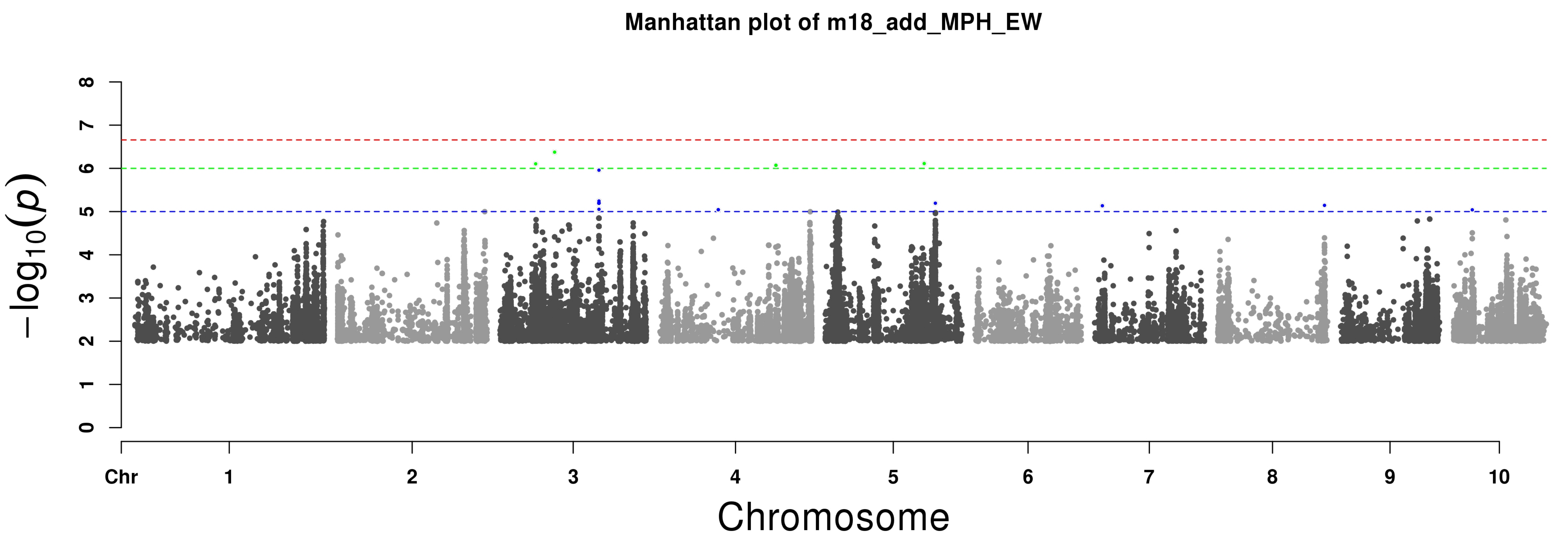

Manhattan plot of m18_add_MPH_EW
$-\log_{10}(p)$
0
1
2
3
4
5
6
7
8
Chr
1
2
3
4
5
6
7
8
9
10
Chromosome

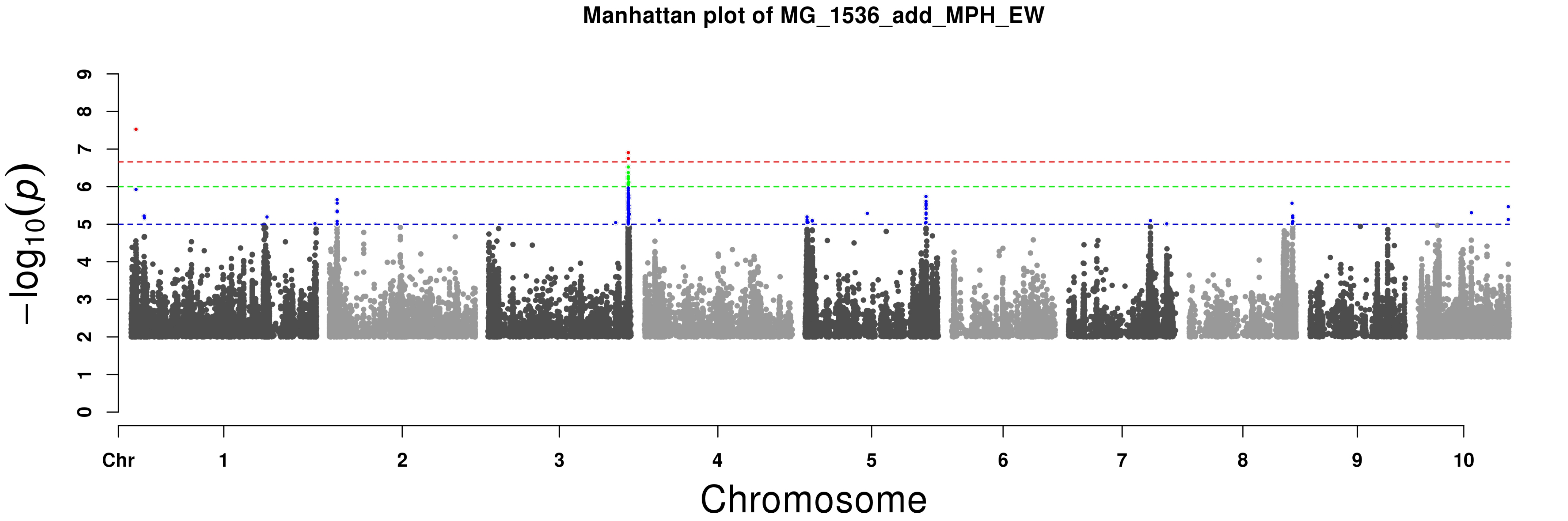

Manhattan plot of MG_1536_add_MPH_EW
$-\log_{10}(p)$
0
1
2
3
4
5
6
7
8
9
Chr
1
2
3
4
5
6
7
8
9
10
Chromosome

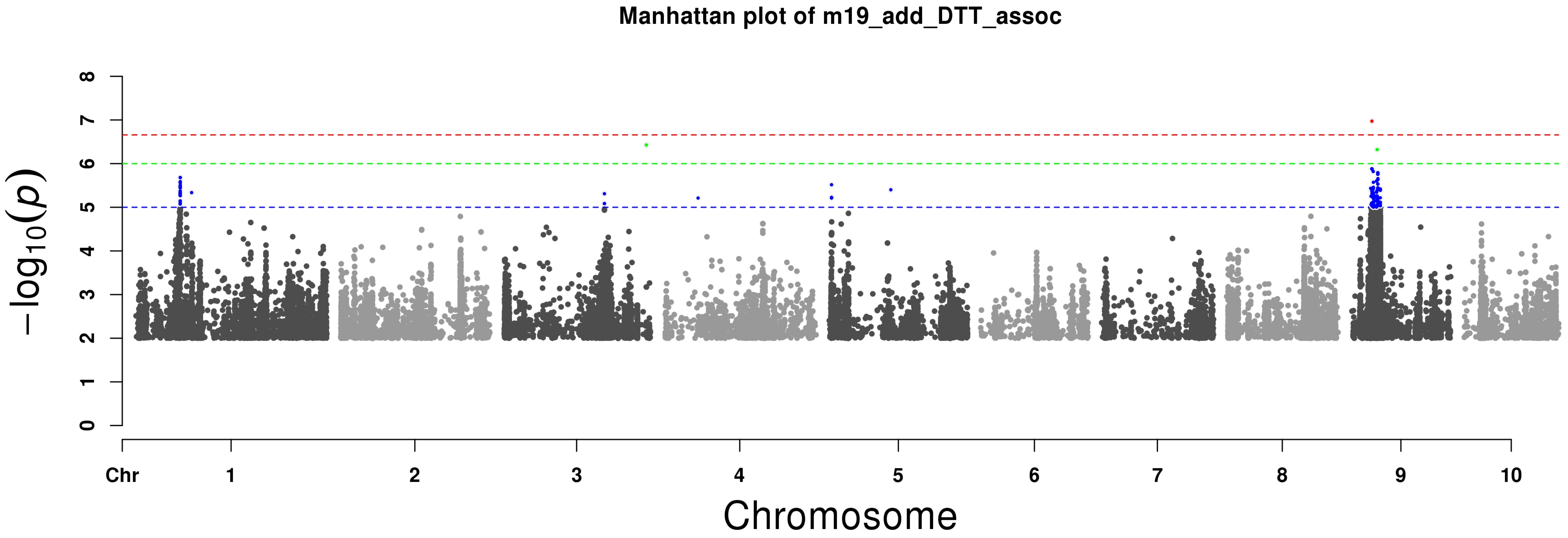

Manhattan plot of m19_add_DTT_assoc
$-\log_{10}(p)$
0
1
2
3
4
5
6
7
8
Chr
1
2
3
4
5
6
7
8
9
10
Chromosome

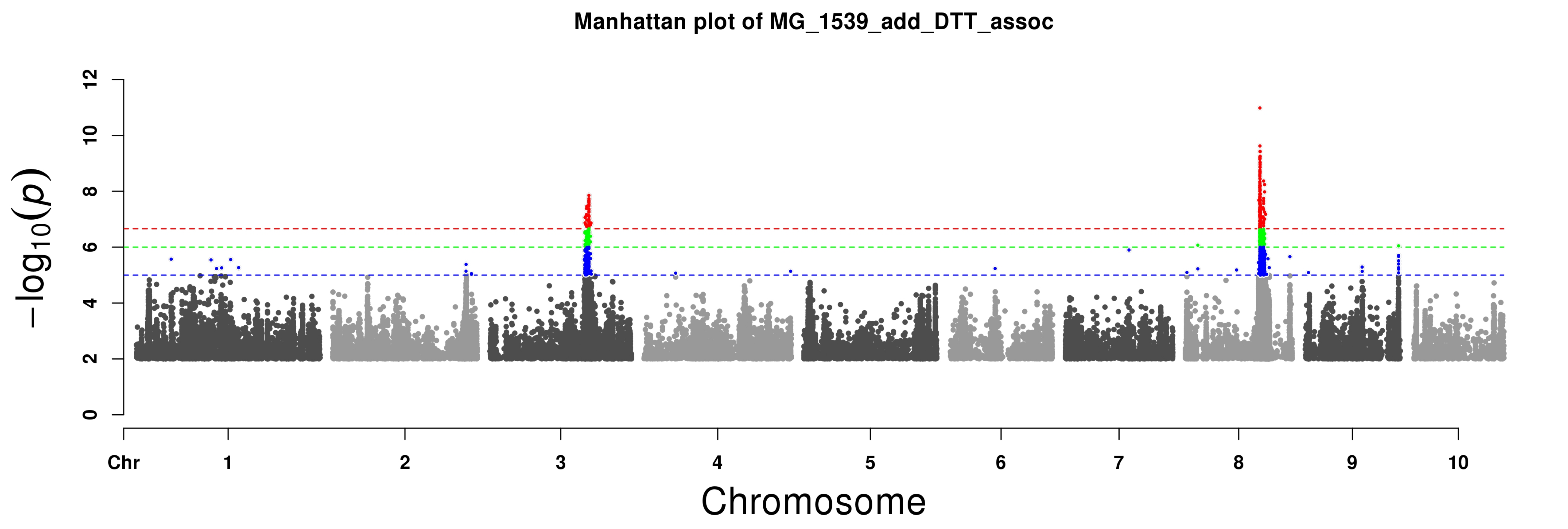

Manhattan plot of MG_1539_add_DTT_assoc
−log10(p)
0
2
4
6
8
10
12
Chr
1
2
3
4
5
6
7
8
9
10
Chromosome

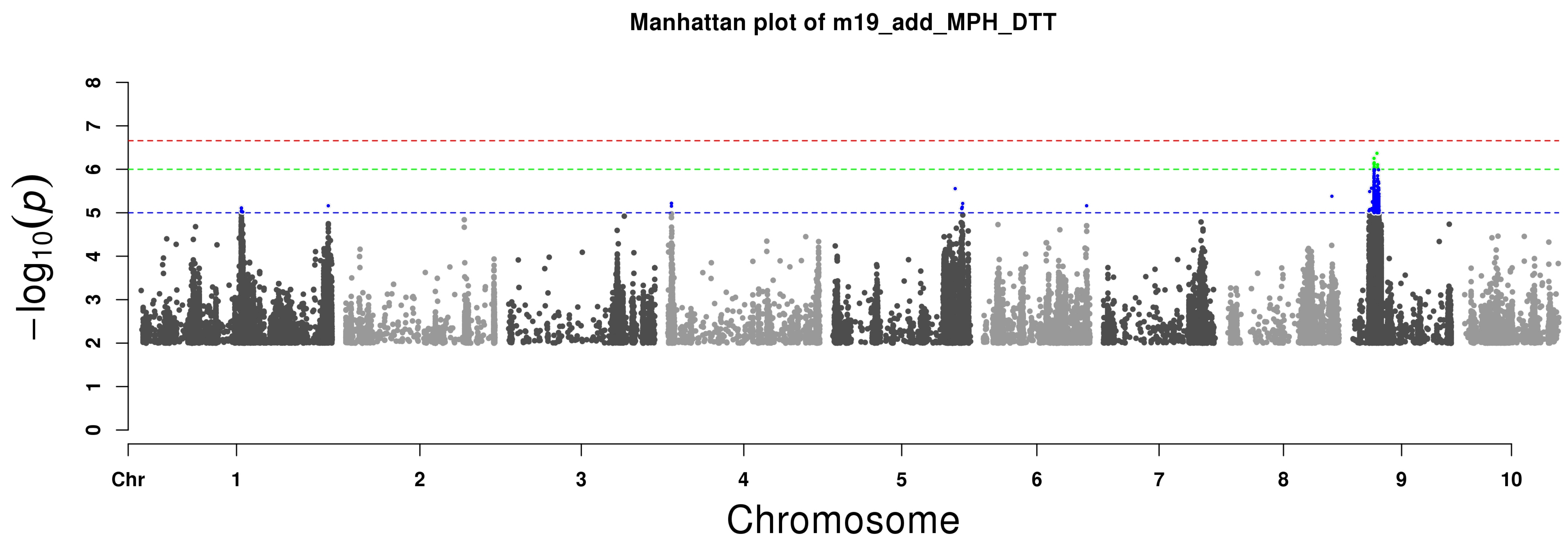

Manhattan plot of m19_add_MPH_DTT
0
1
2
3
4
5
6
7
8
$-\log_{10}(p)$
Chr
1
2
3
4
5
6
7
8
9
10
Chromosome

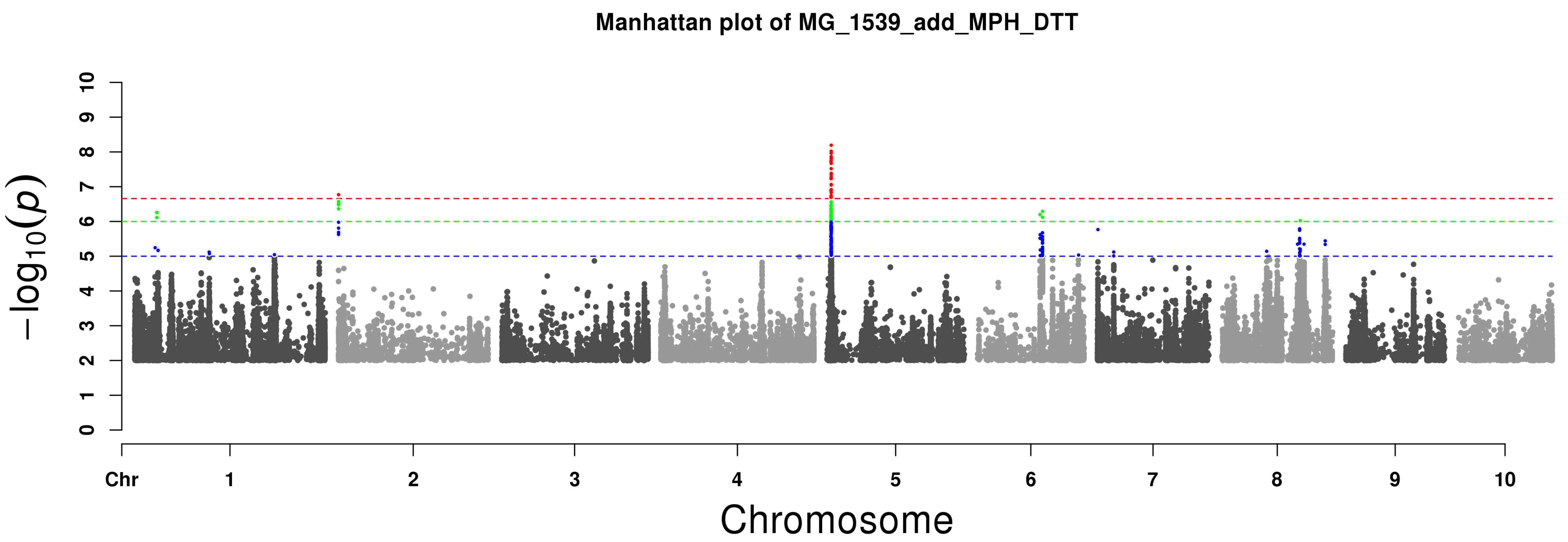

Manhattan plot of MG_1539_add_MPH_DTT
$-\log_{10}(p)$
0
1
2
3
4
5
6
7
8
9
10
Chr
1
2
3
4
5
6
7
8
9
10
Chromosome

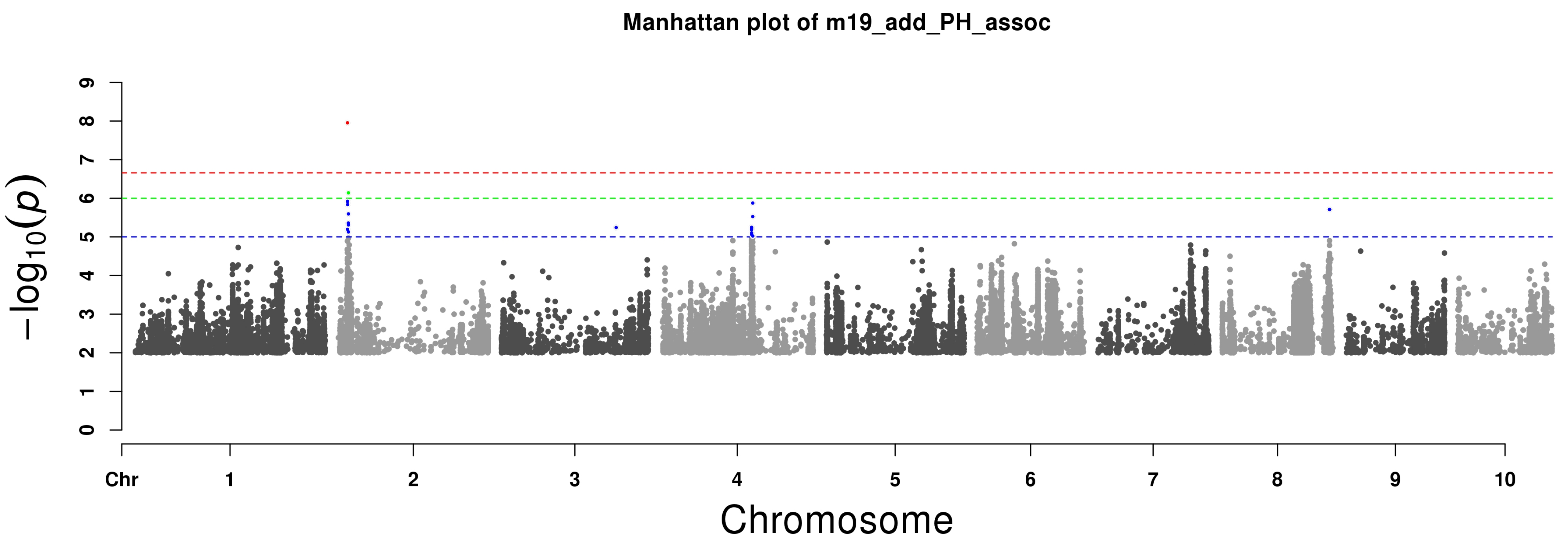

Manhattan plot of m19_add_PH_assoc
$-\log_{10}(p)$
0
1
2
3
4
5
6
7
8
9
Chr
1
2
3
4
5
6
7
8
9
10
Chromosome

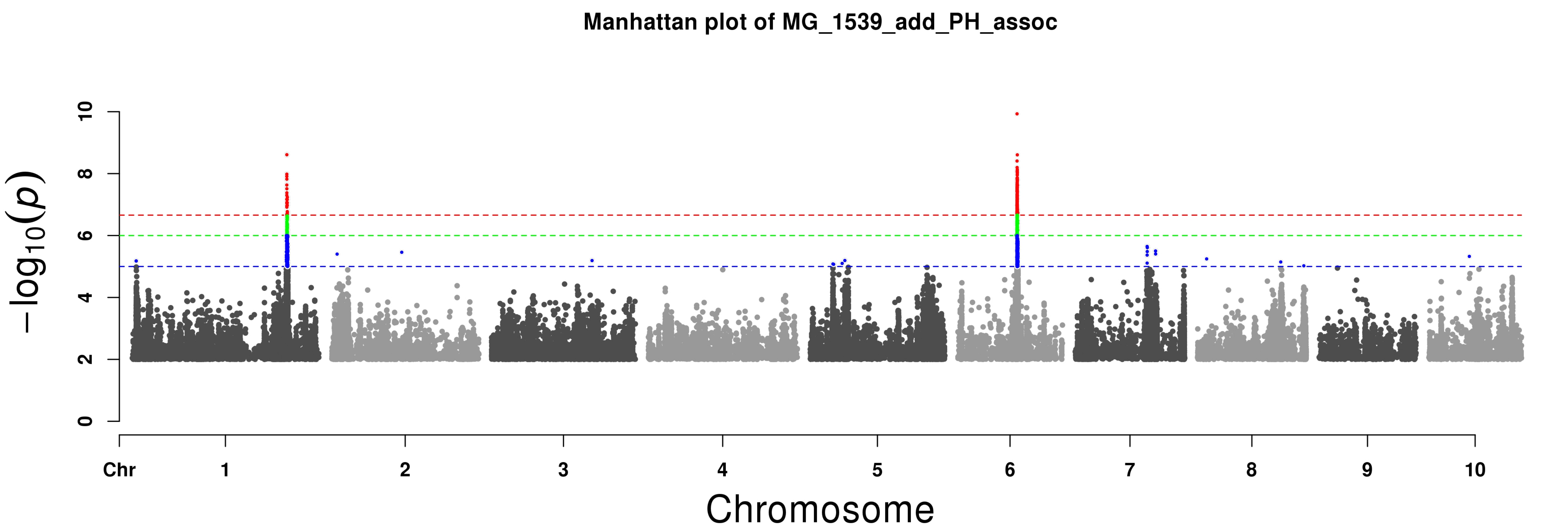

Manhattan plot of MG_1539_add_PH_assoc
$-\log_{10}(p)$
0
2
4
6
8
10
Chr
1
2
3
4
5
6
7
8
9
10
Chromosome

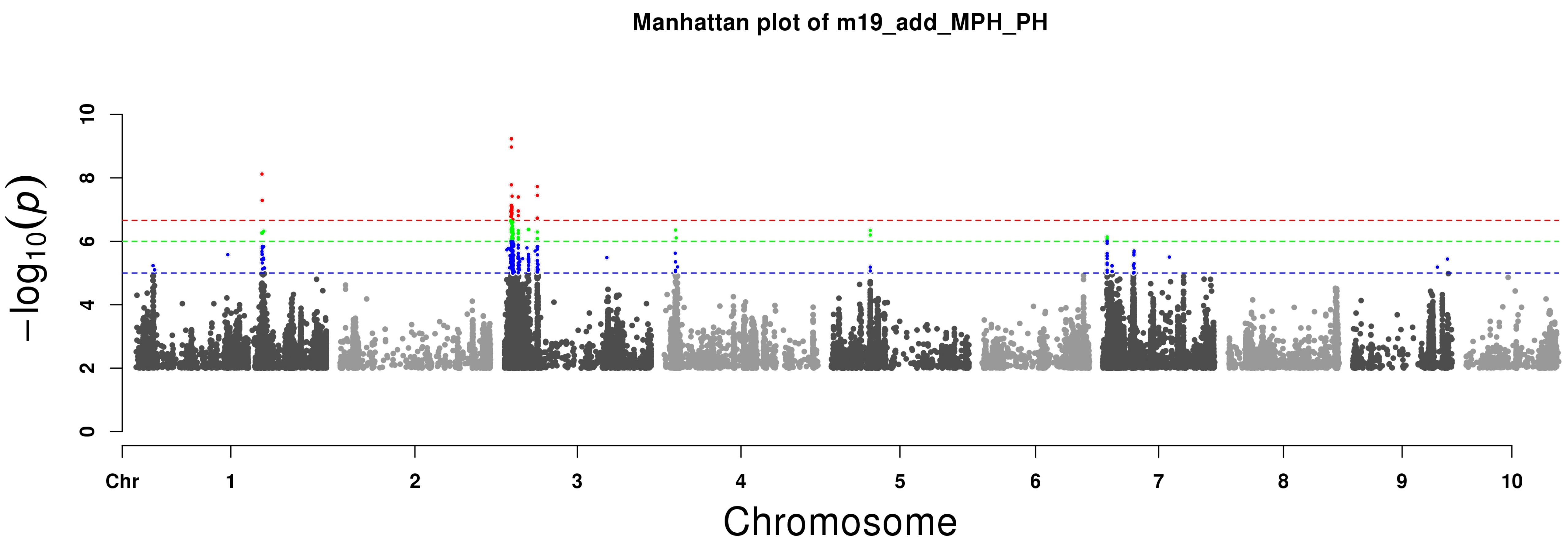

Manhattan plot of m19_add_MPH_PH
−log10(p)
0
2
4
6
8
10
Chr
1
2
3
4
5
6
7
8
9
10
Chromosome

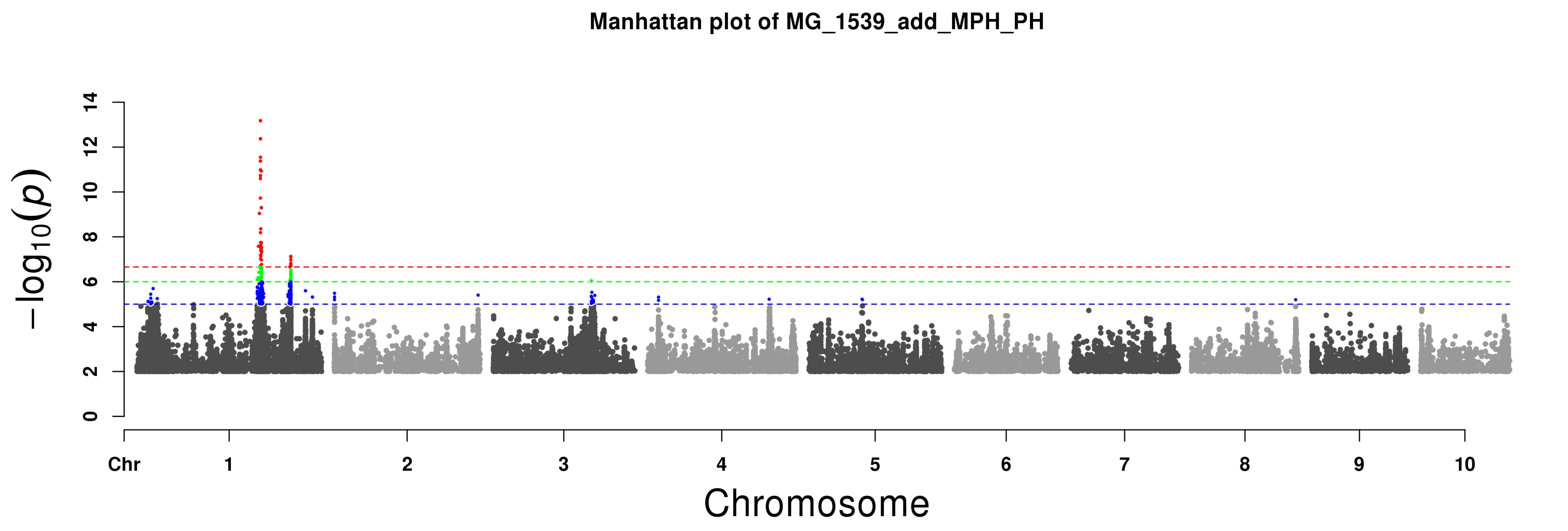

Manhattan plot of MG_1539_add_MPH_PH
$-\log_{10}(p)$
0
2
4
6
8
10
12
14
Chr
1
2
3
4
5
6
7
8
9
10
Chromosome

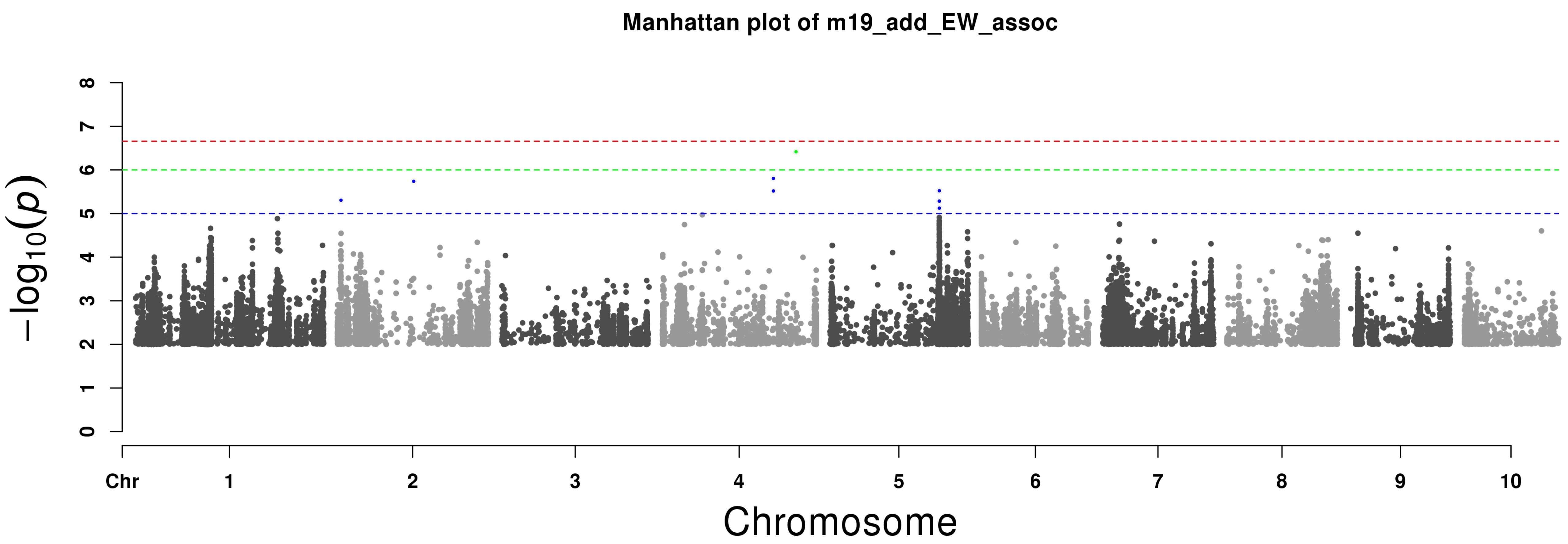

Manhattan plot of m19_add_EW_assoc
$-\log_{10}(p)$
0
1
2
3
4
5
6
7
8
Chr
1
2
3
4
5
6
7
8
9
10
Chromosome

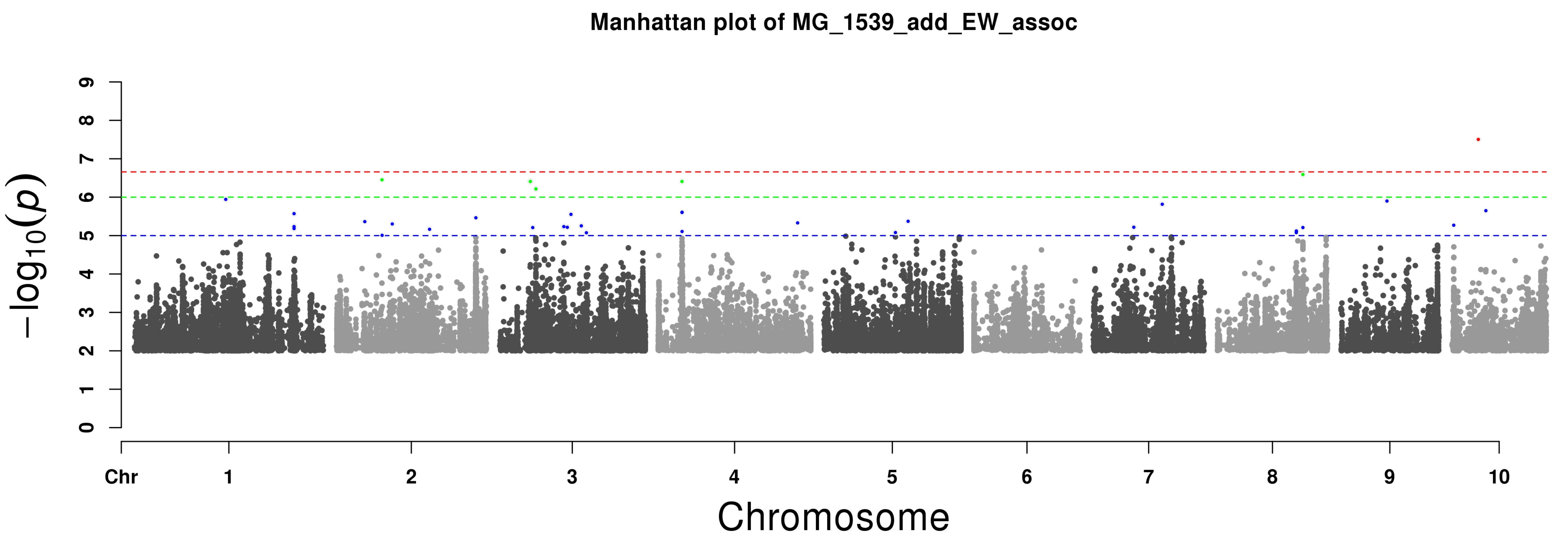

Manhattan plot of MG_1539_add_EW_assoc
−log10(p)
0
1
2
3
4
5
6
7
8
9
Chr
1
2
3
4
5
6
7
8
9
10
Chromosome

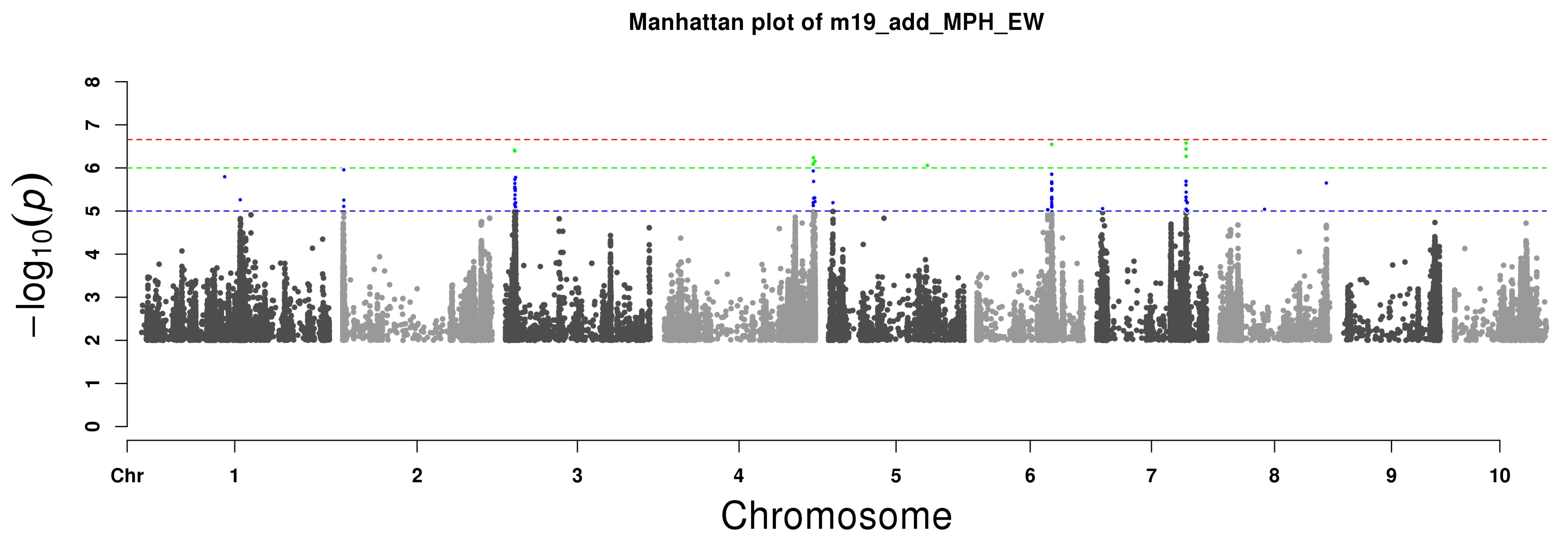

Manhattan plot of m19_add_MPH_EW
$-\log_{10}(p)$
0
1
2
3
4
5
6
7
8
Chr
1
2
3
4
5
6
7
8
9
10
Chromosome

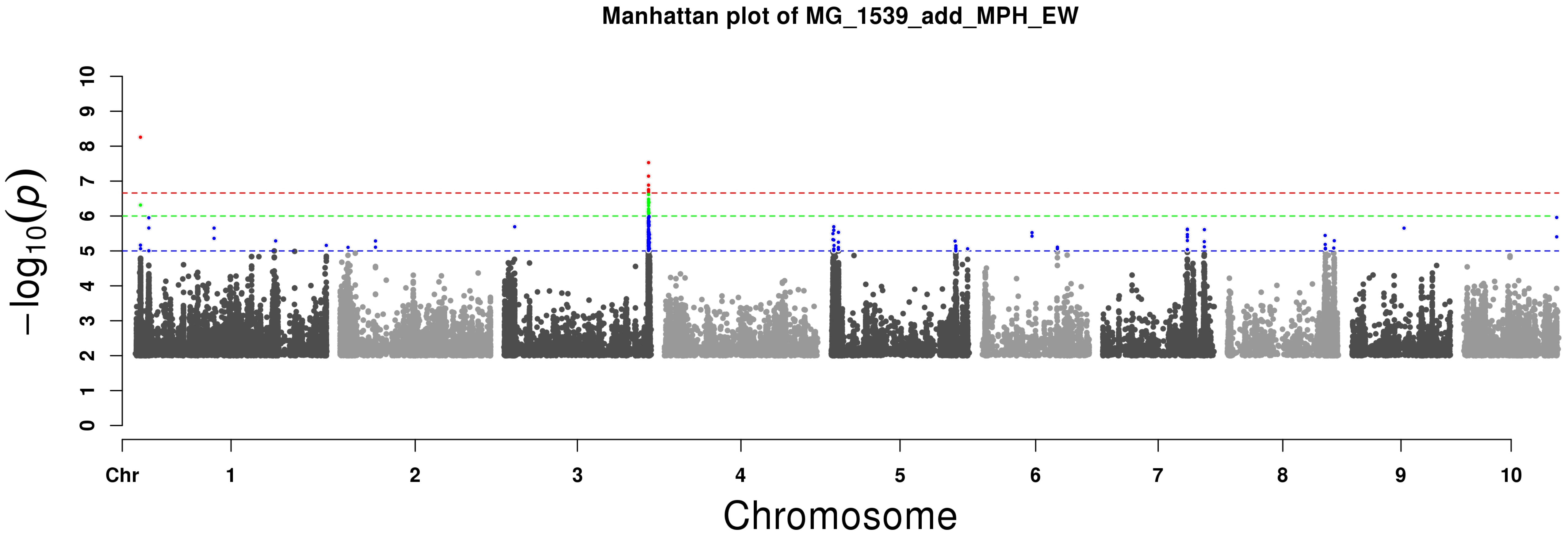

Manhattan plot of MG_1539_add_MPH_EW
$-\log_{10}(p)$
0
1
2
3
4
5
6
7
8
9
10
Chr
1
2
3
4
5
6
7
8
9
10
Chromosome

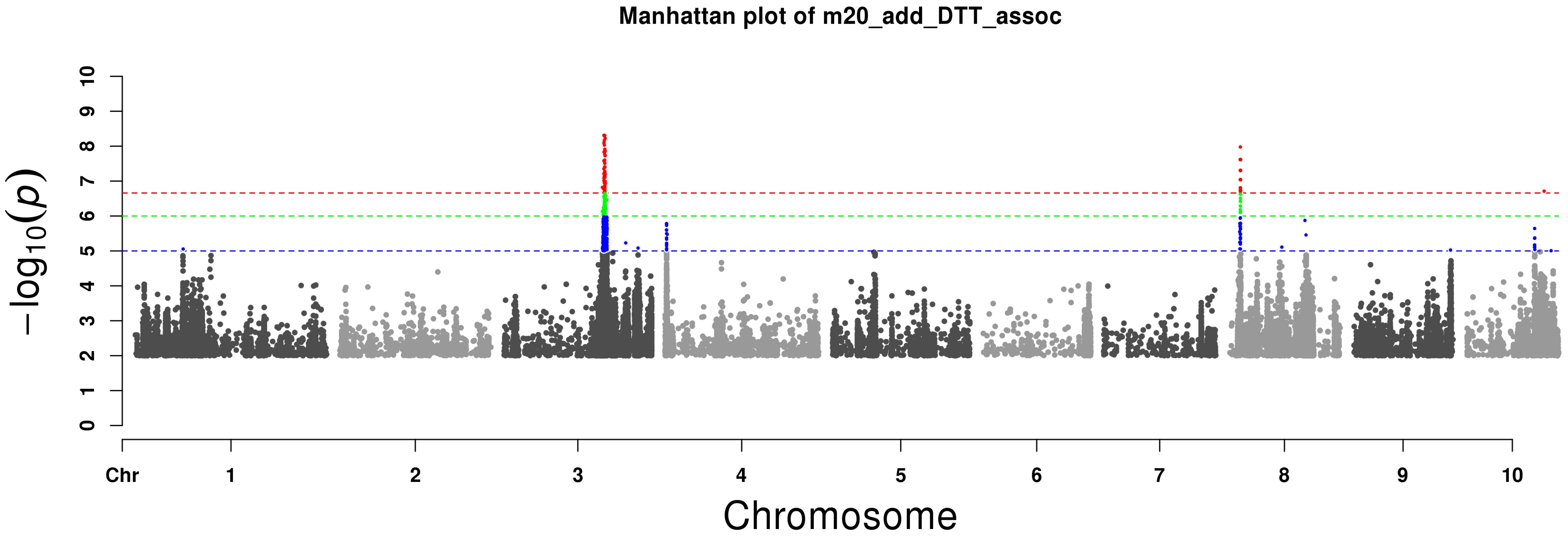

Manhattan plot of m20_add_DTT_assoc
$-\log_{10}(p)$
0
1
2
3
4
5
6
7
8
9
10
Chr
1
2
3
4
5
6
7
8
9
10
Chromosome

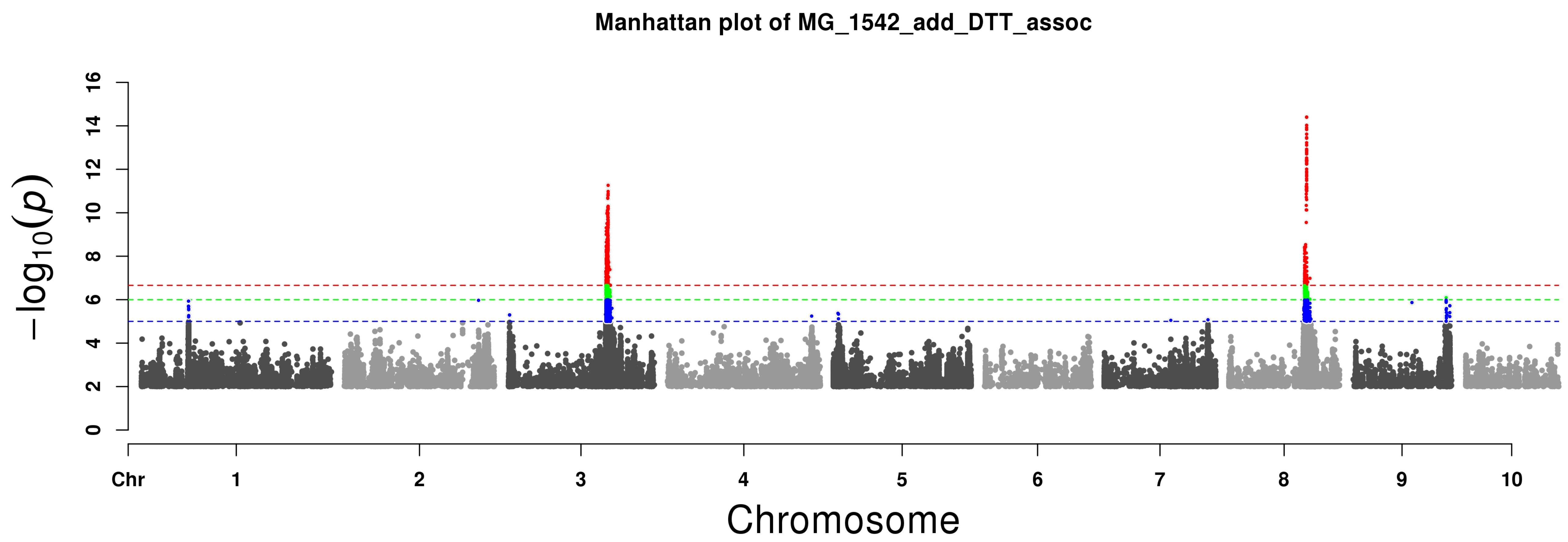

Manhattan plot of MG_1542_add_DTT_assoc
$-\log_{10}(p)$
0
2
4
6
8
10
12
14
16
Chr
1
2
3
4
5
6
7
8
9
10
Chromosome

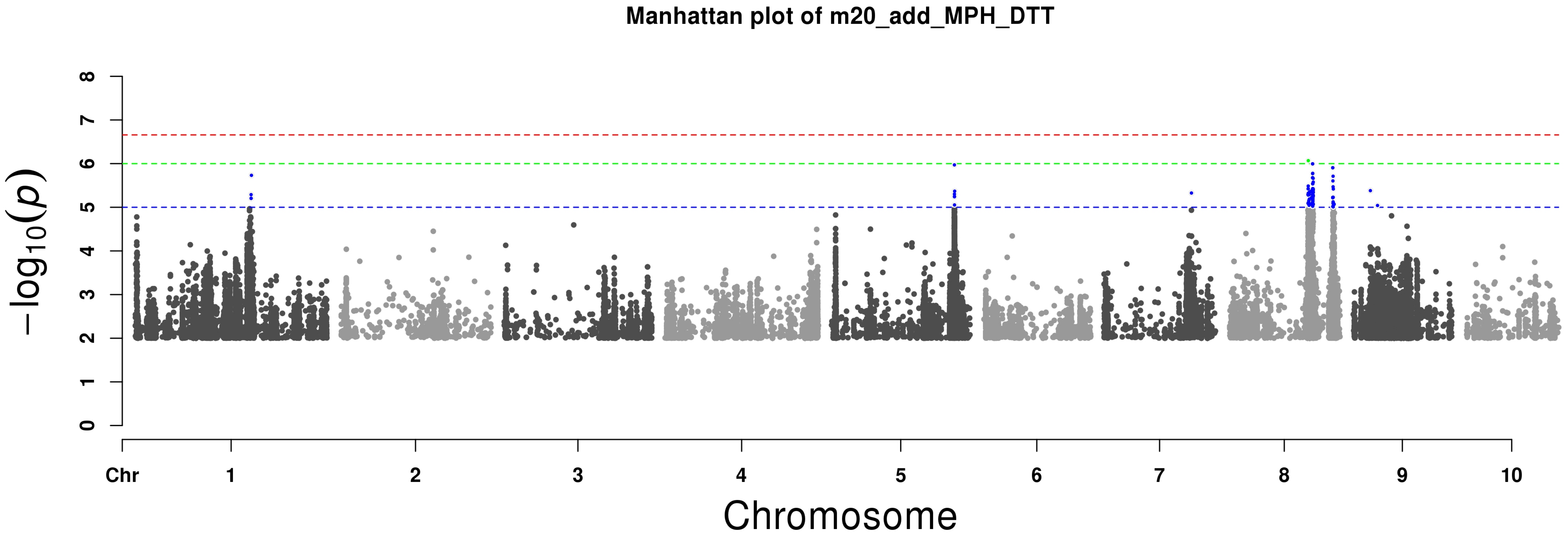

Manhattan plot of m20_add_MPH_DTT
$-\log_{10}(p)$
0
1
2
3
4
5
6
7
8
Chr
1
2
3
4
5
6
7
8
9
10
Chromosome

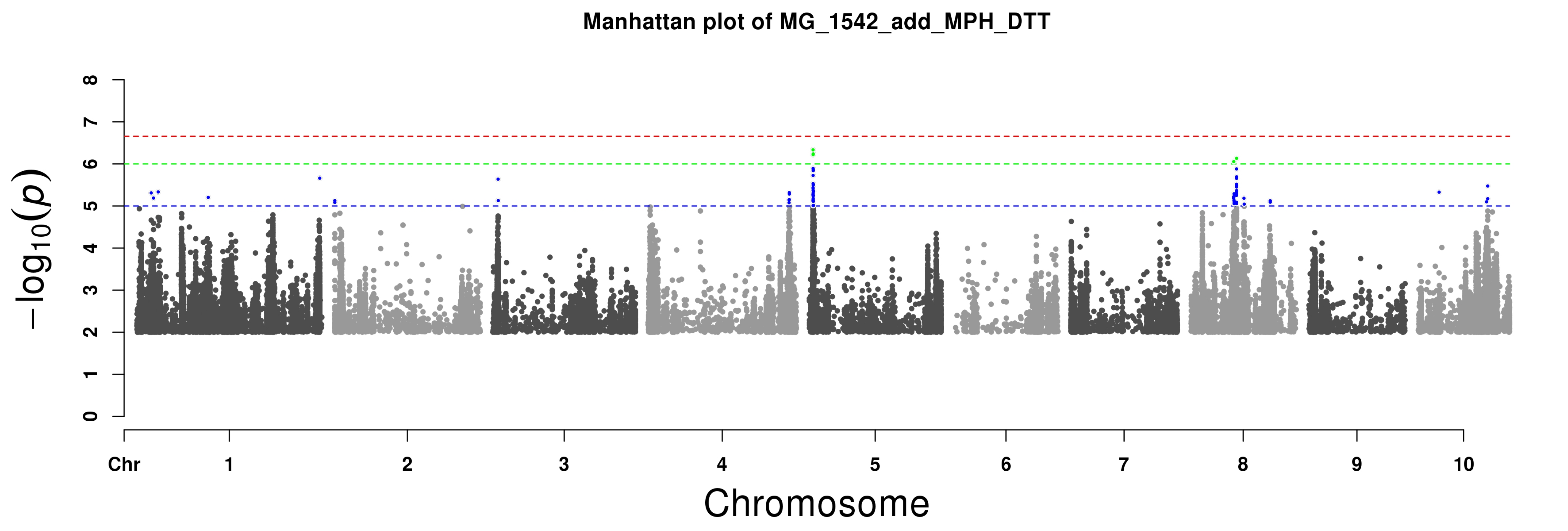

Manhattan plot of MG_1542_add_MPH_DTT
$-\log_{10}(p)$
0
1
2
3
4
5
6
7
8
Chr
1
2
3
4
5
6
7
8
9
10
Chromosome

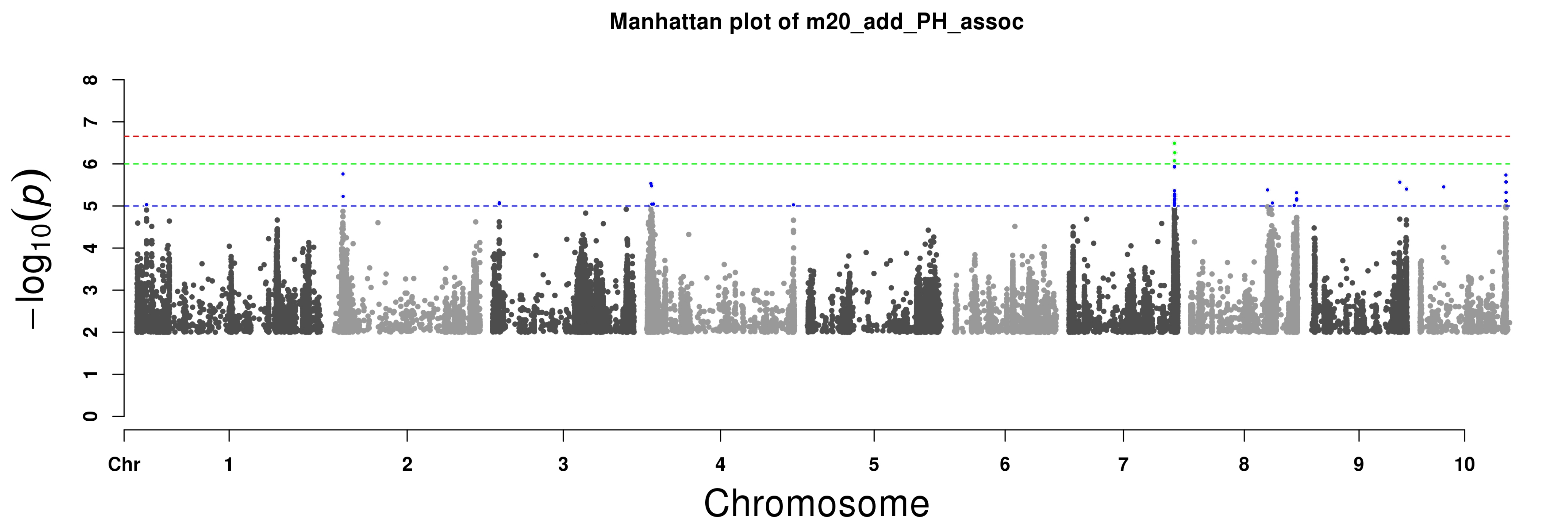
Manhattan plot of m20_add_PH_assoc
$-\log_{10}(p)$
0
1
2
3
4
5
6
7
8
Chr
1
2
3
4
5
6
7
8
9
10
Chromosome

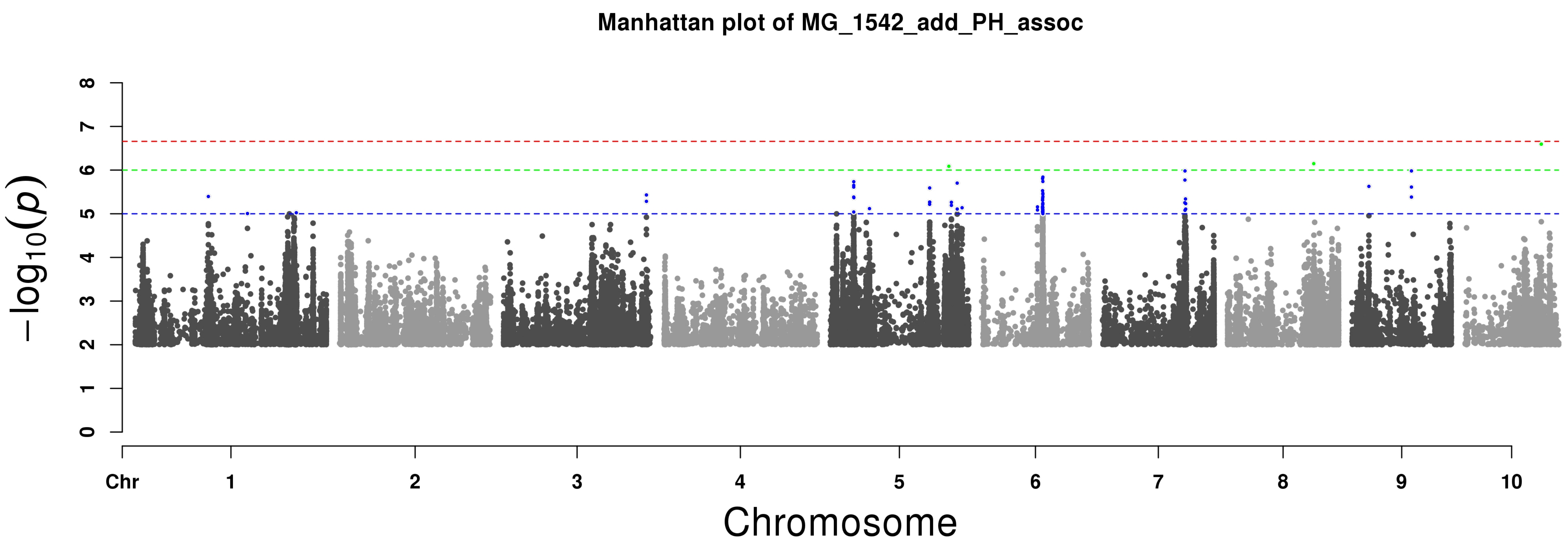

Manhattan plot of MG_1542_add_PH_assoc
$-\log_{10}(p)$
0
1
2
3
4
5
6
7
8
Chr
1
2
3
4
5
6
7
8
9
10
Chromosome

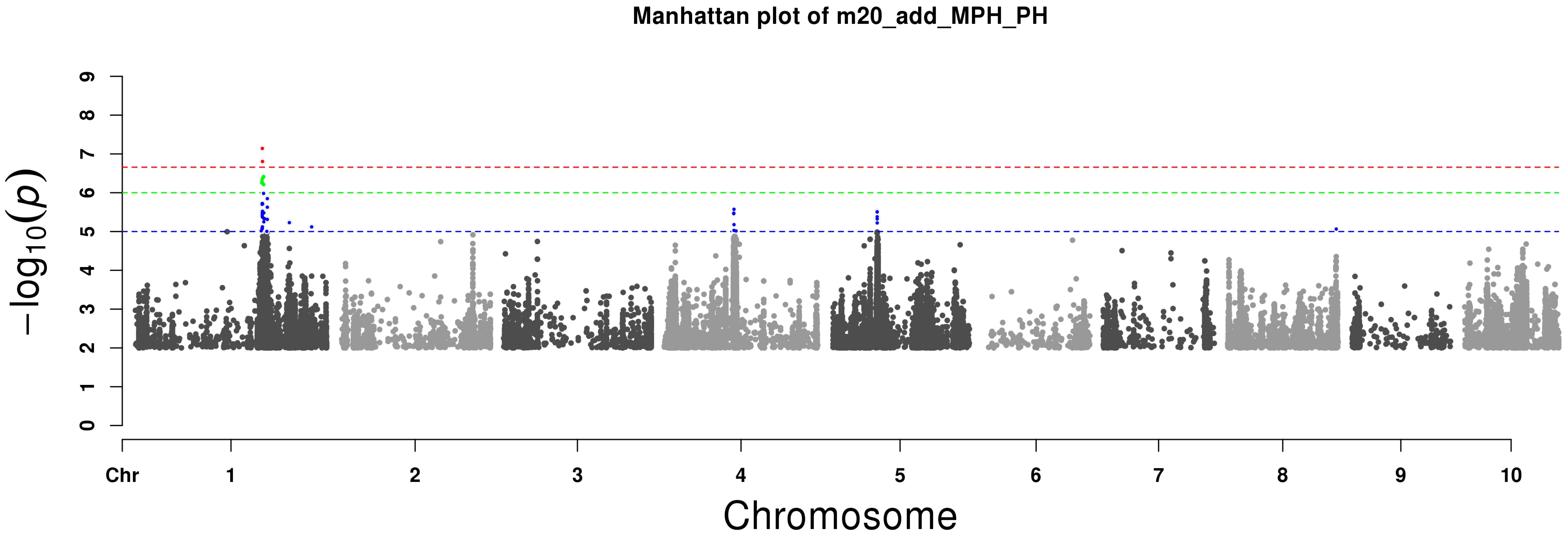

Manhattan plot of m20_add_MPH_PH
$-\log_{10}(p)$
0
1
2
3
4
5
6
7
8
9
Chr
1
2
3
4
5
6
7
8
9
10
Chromosome

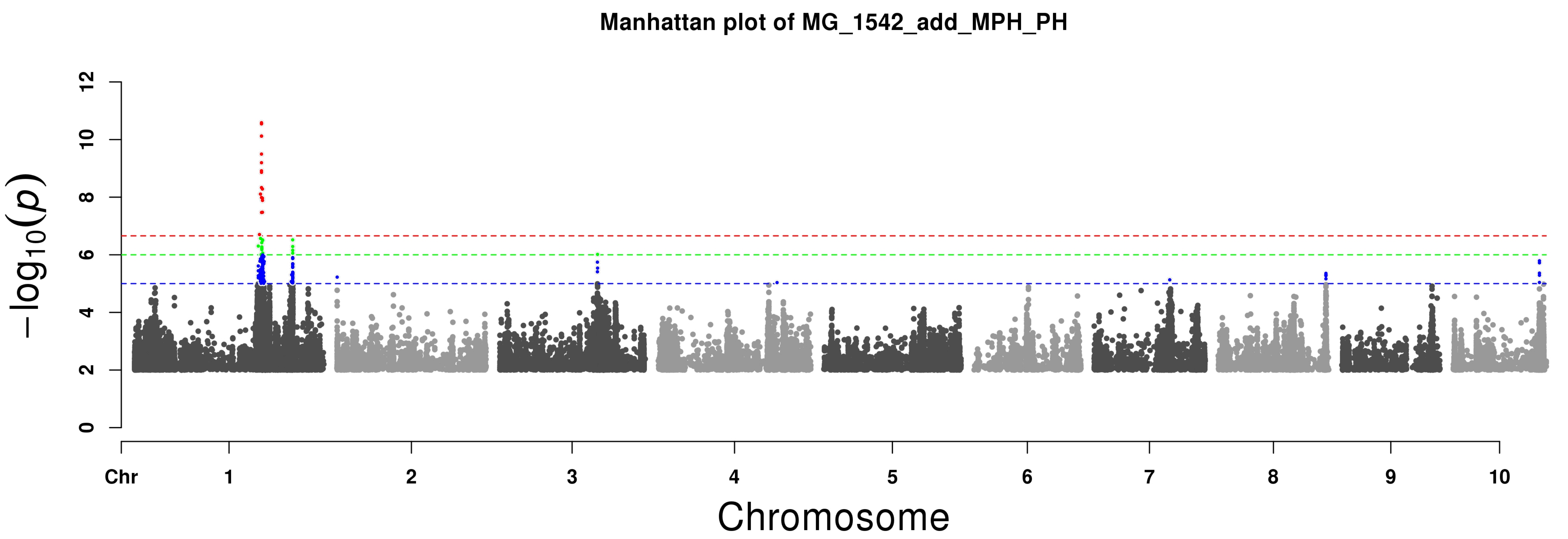
Manhattan plot of MG_1542_add_MPH_PH
$-\log_{10}(p)$
0
2
4
6
8
10
12
Chr
1
2
3
4
5
6
7
8
9
10
Chromosome

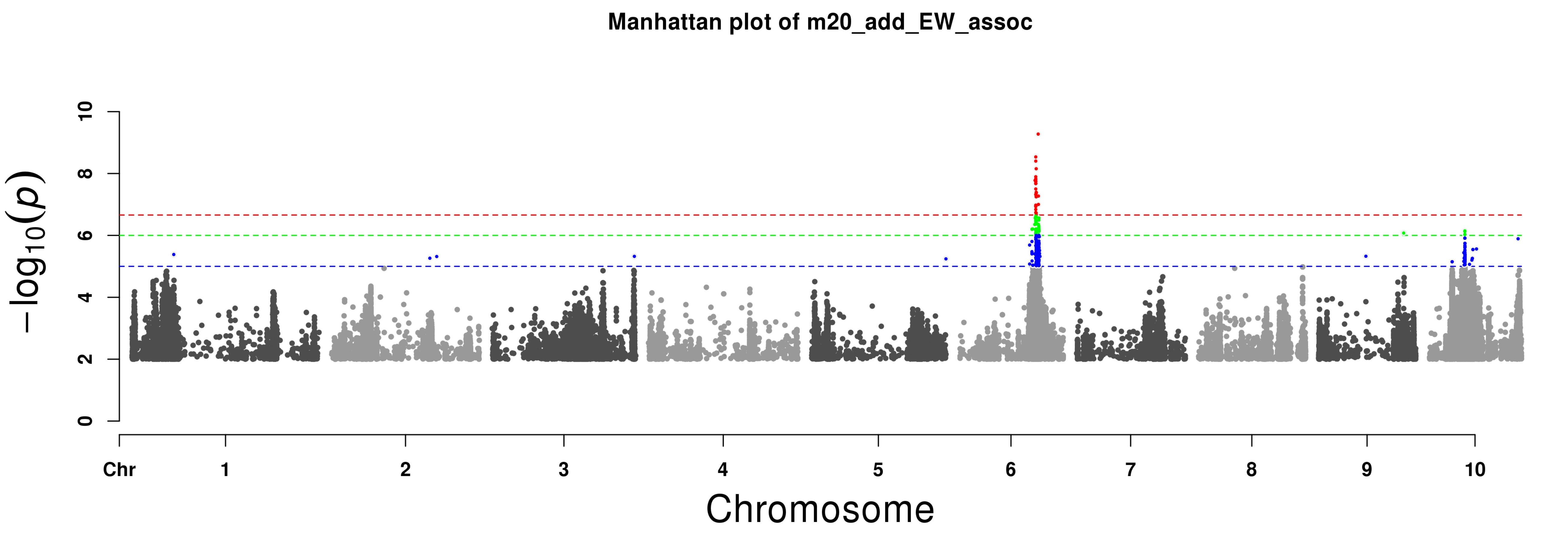

Manhattan plot of m20_add_EW_assoc
$-\log_{10}(p)$
0
2
4
6
8
10
Chr
1
2
3
4
5
6
7
8
9
10
Chromosome

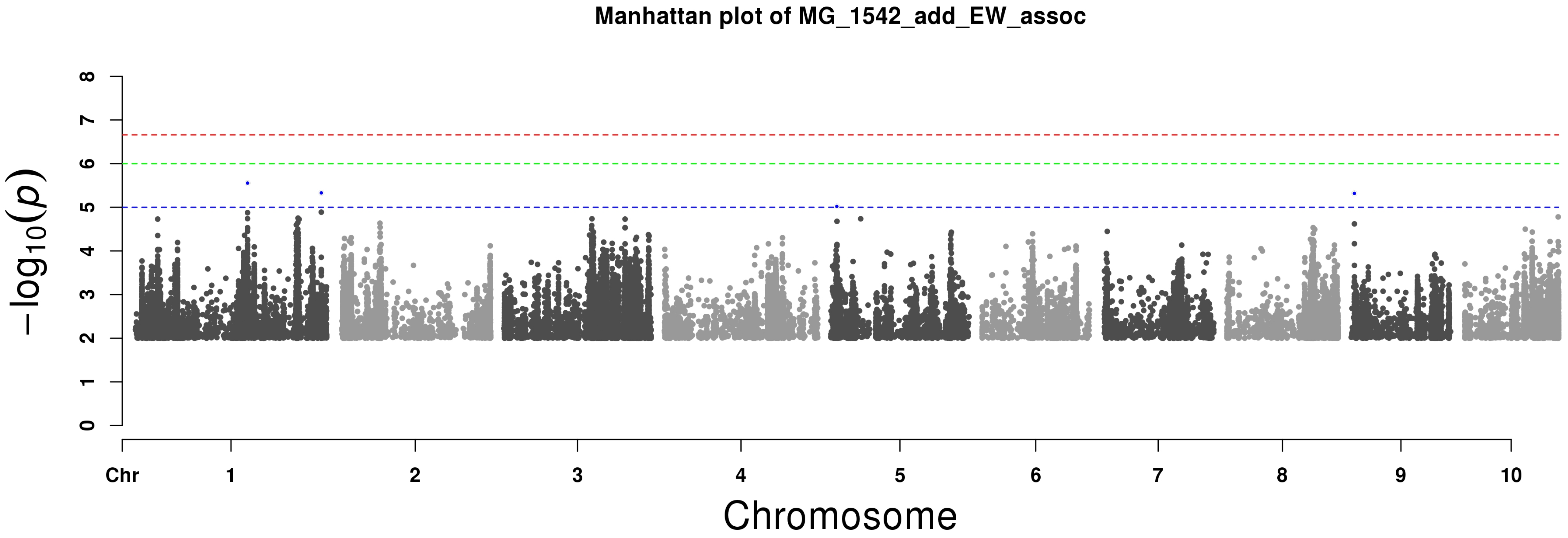

Manhattan plot of MG_1542_add_EW_assoc
$-\log_{10}(p)$
0
1
2
3
4
5
6
7
8
Chr
1
2
3
4
5
6
7
8
9
10
Chromosome

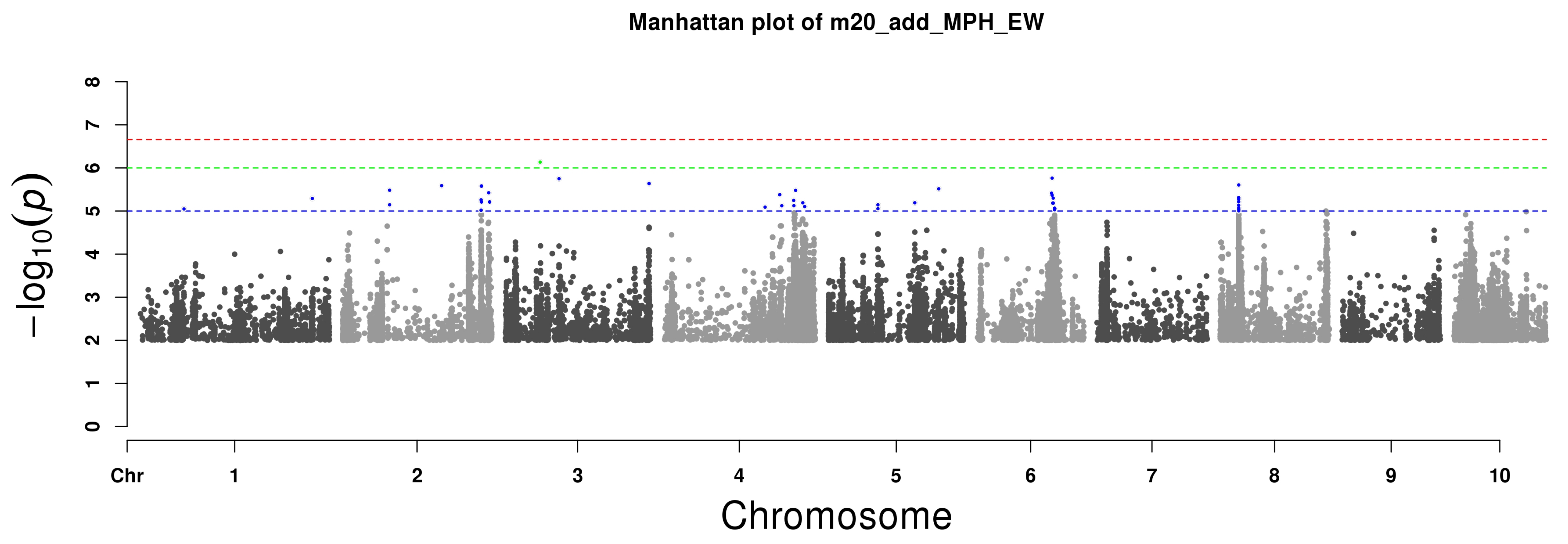

Manhattan plot of m20_add_MPH_EW
$-\log_{10}(p)$
0
1
2
3
4
5
6
7
8
Chr
1
2
3
4
5
6
7
8
9
10
Chromosome

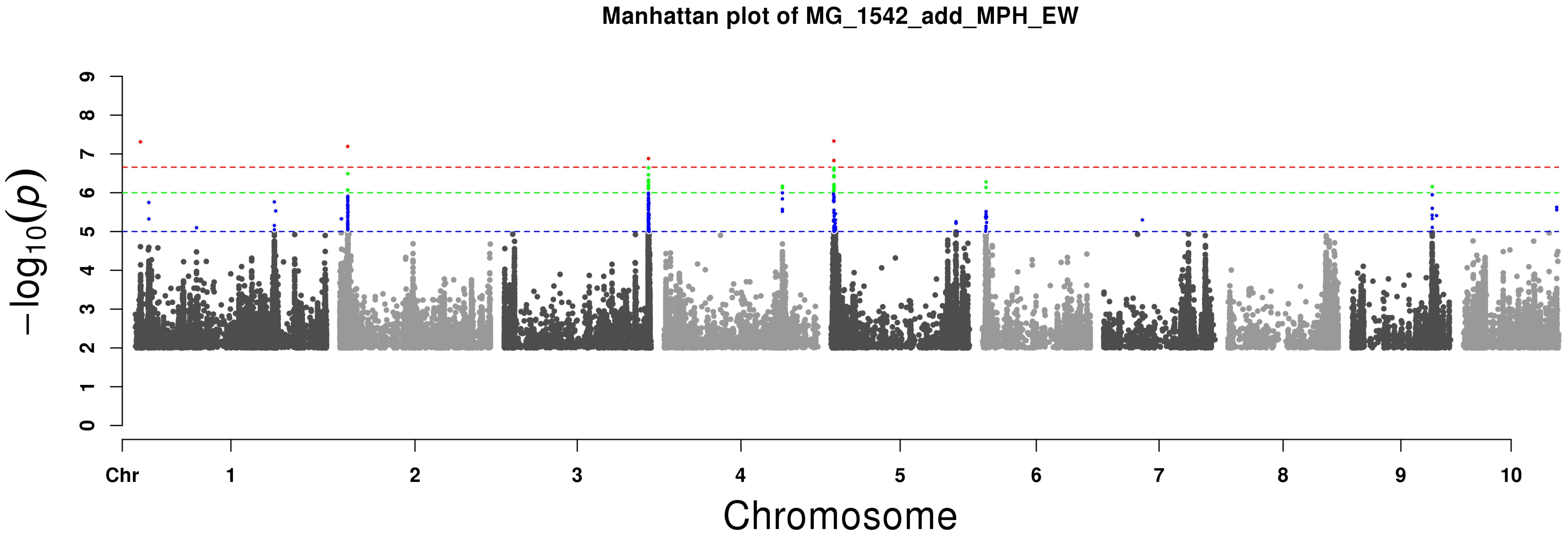
Manhattan plot of MG_1542_add_MPH_EW
$-\log_{10}(p)$
0
1
2
3
4
5
6
7
8
9
Chr
1
2
3
4
5
6
7
8
9
10
Chromosome

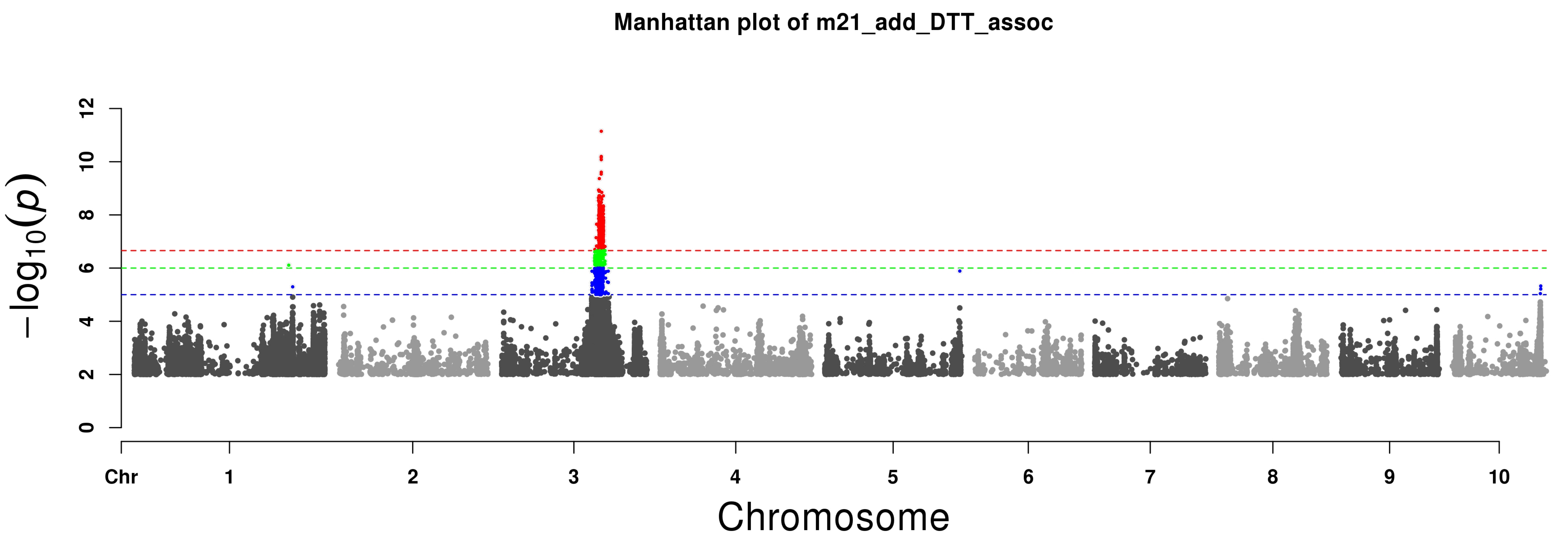

Manhattan plot of m21_add_DTT_assoc
$-\log_{10}(p)$
0
2
4
6
8
10
12
Chr
1
2
3
4
5
6
7
8
9
10
Chromosome

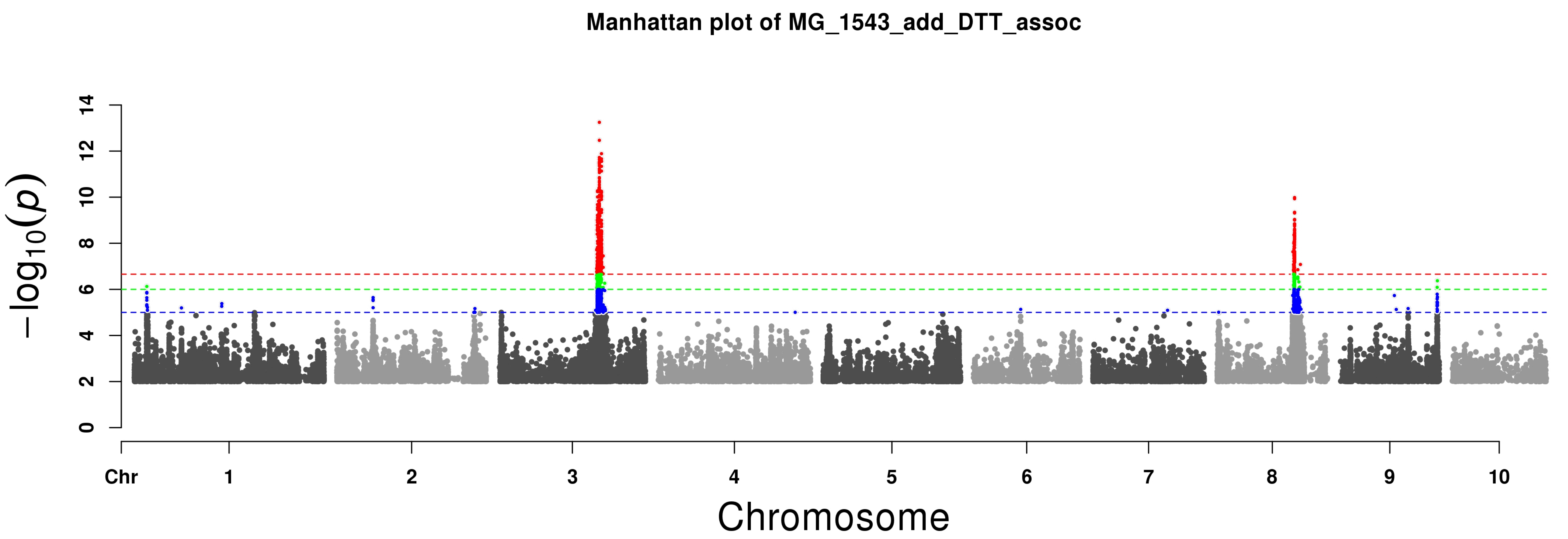

Manhattan plot of MG_1543_add_DTT_assoc
$-\log_{10}(p)$
0
2
4
6
8
10
12
14
Chr
1
2
3
4
5
6
7
8
9
10
Chromosome

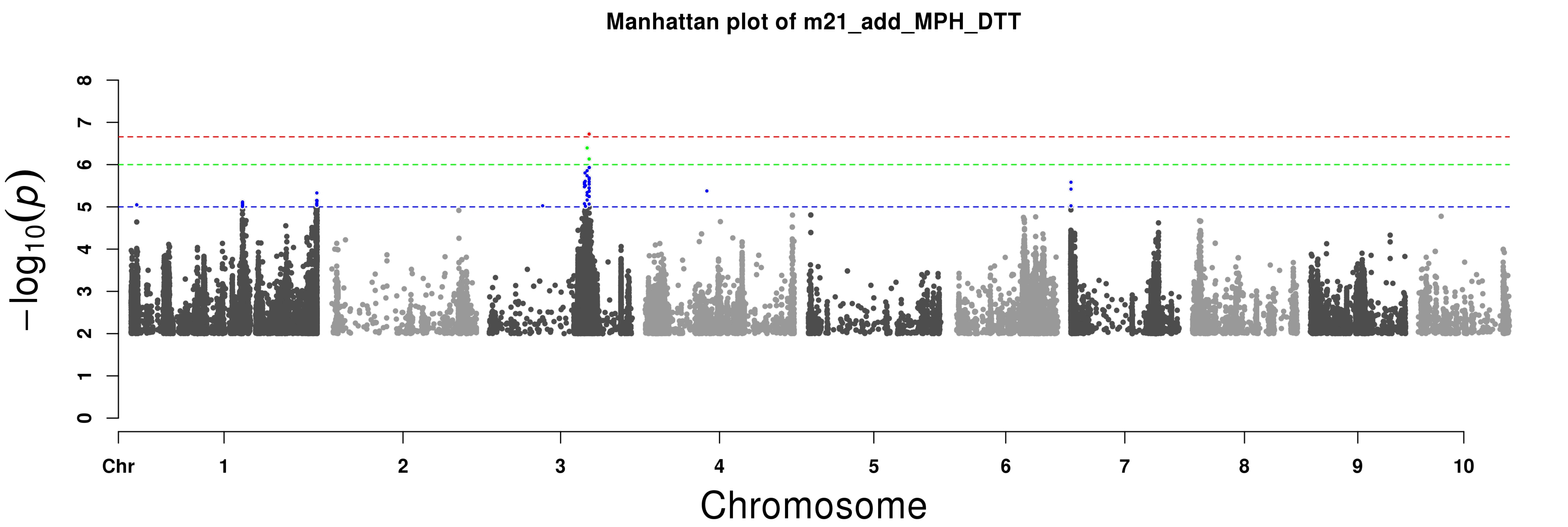

Manhattan plot of m21_add_MPH_DTT
$-\log_{10}(p)$
0
1
2
3
4
5
6
7
8
Chr
1
2
3
4
5
6
7
8
9
10
Chromosome

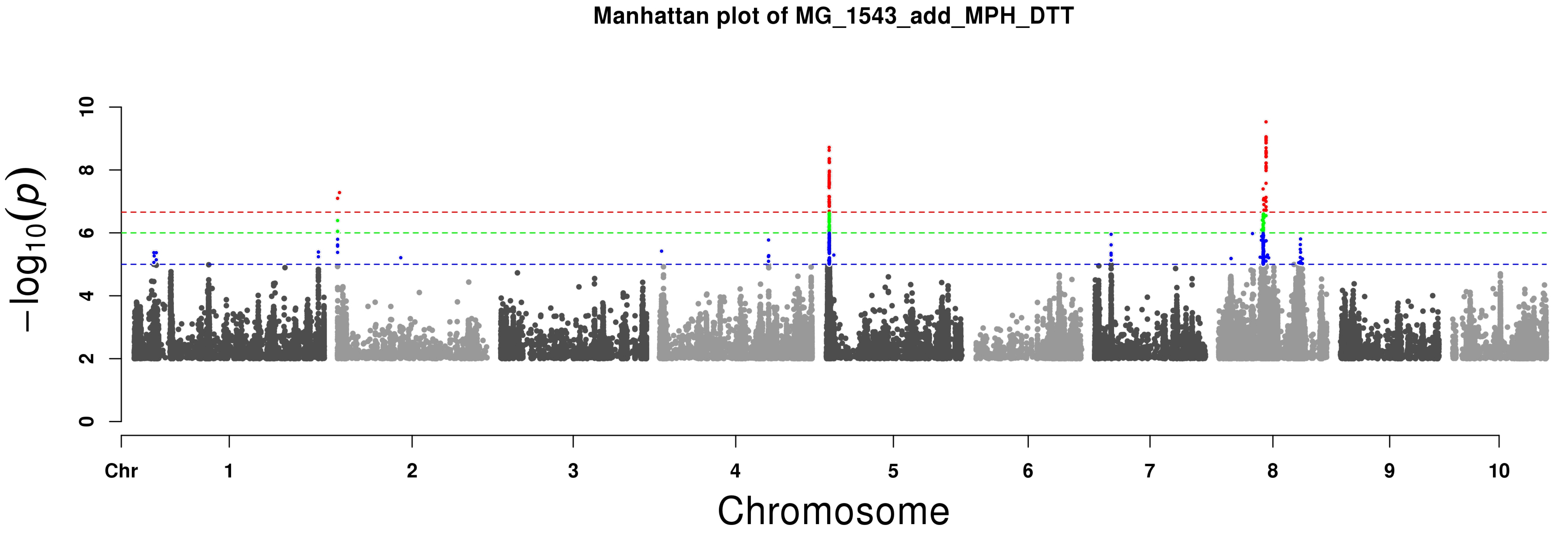

Manhattan plot of MG_1543_add_MPH_DTT
$-\log_{10}(p)$
0
2
4
6
8
10
Chr
1
2
3
4
5
6
7
8
9
10
Chromosome

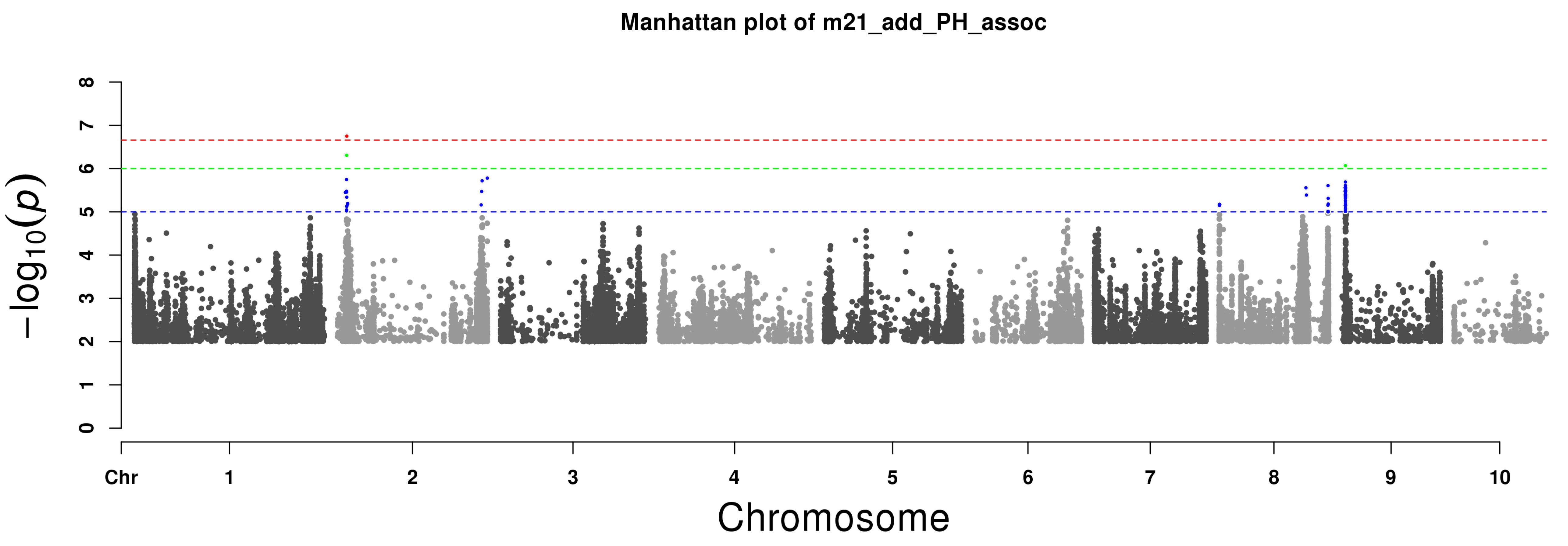
Manhattan plot of m21_add_PH_assoc
$-\log_{10}(p)$
0
1
2
3
4
5
6
7
8
Chr
1
2
3
4
5
6
7
8
9
10
Chromosome

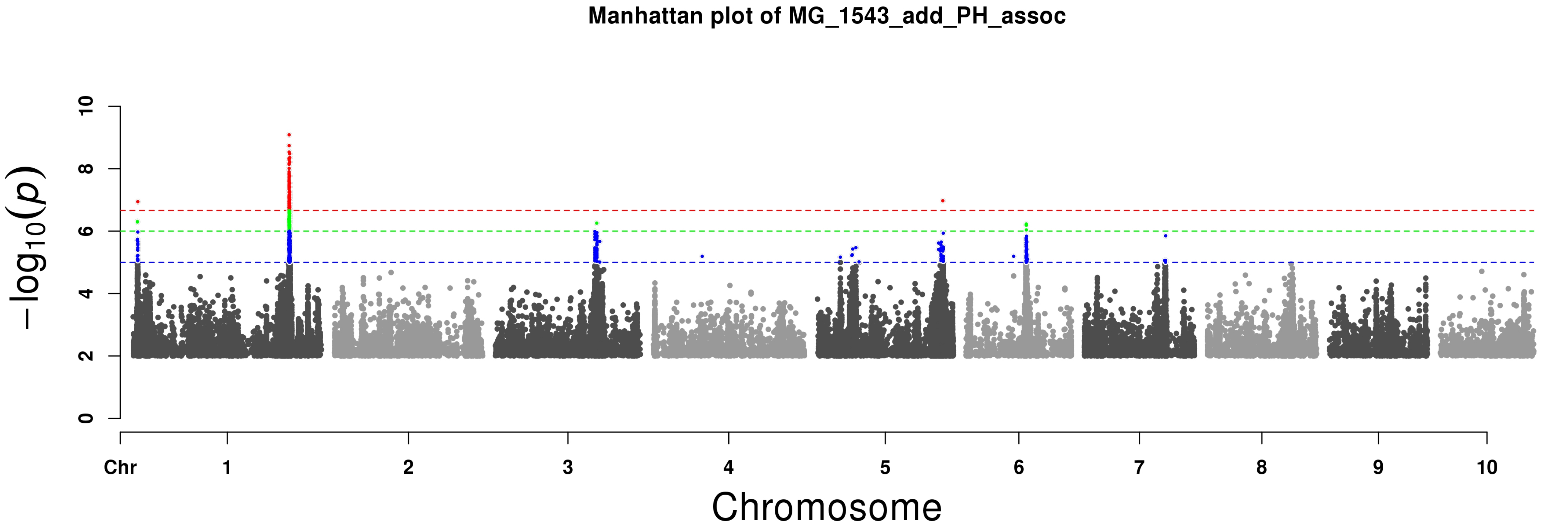

Manhattan plot of MG_1543_add_PH_assoc
$-\log_{10}(p)$
0
2
4
6
8
10
Chr
1
2
3
4
5
6
7
8
9
10
Chromosome

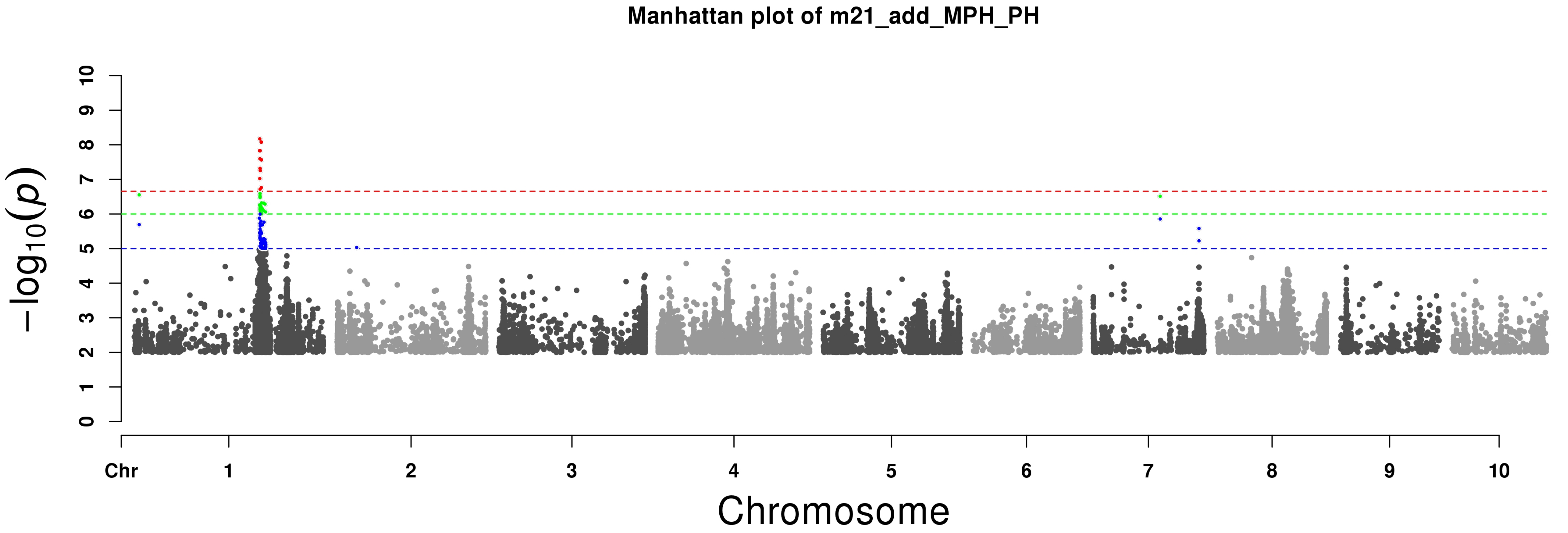

Manhattan plot of m21_add_MPH_PH
$-\log_{10}(p)$
0
1
2
3
4
5
6
7
8
9
10
Chr
1
2
3
4
5
6
7
8
9
10
Chromosome

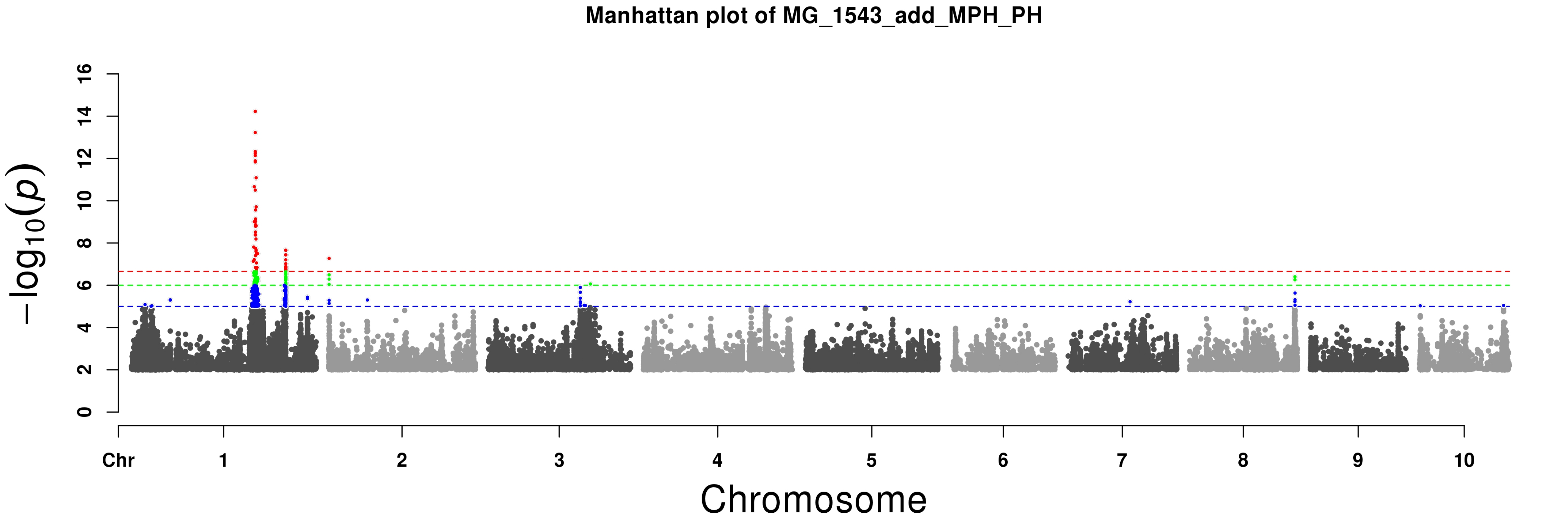
Manhattan plot of MG_1543_add_MPH_PH
$-\log_{10}(p)$
0
2
4
6
8
10
12
14
16
Chr
1
2
3
4
5
6
7
8
9
10
Chromosome

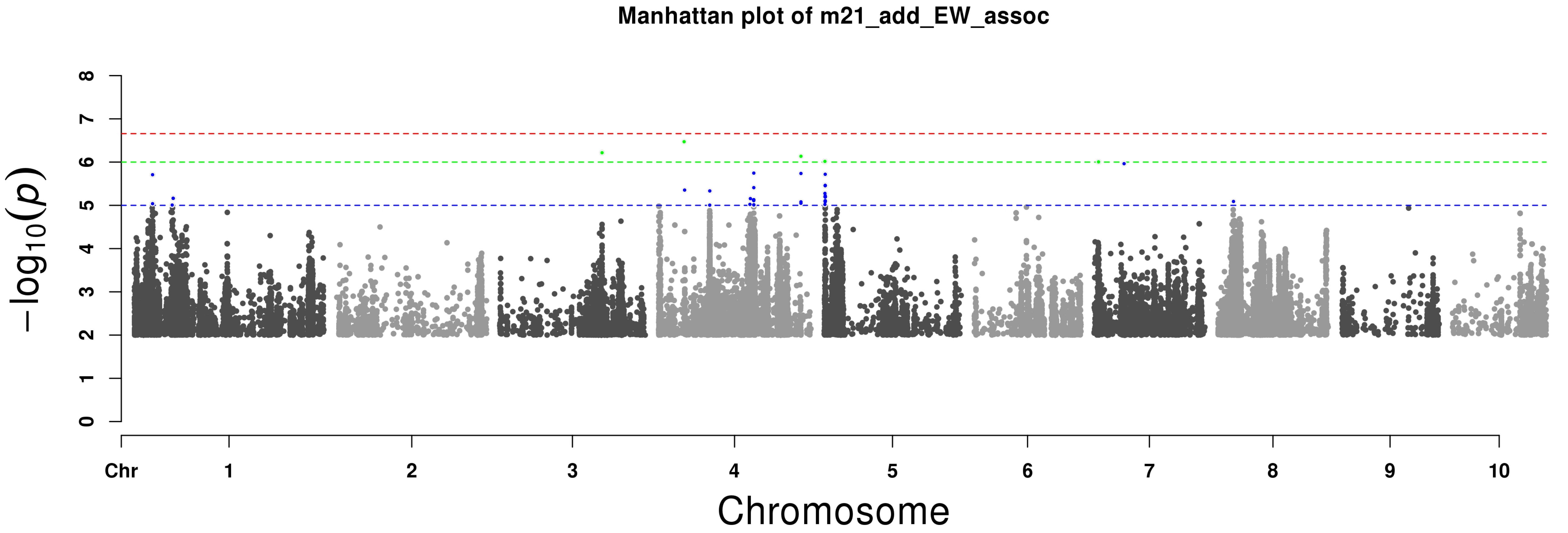
Manhattan plot of m21_add_EW_assoc
$-\log_{10}(p)$
0
1
2
3
4
5
6
7
8
Chr
1
2
3
4
5
6
7
8
9
10
Chromosome

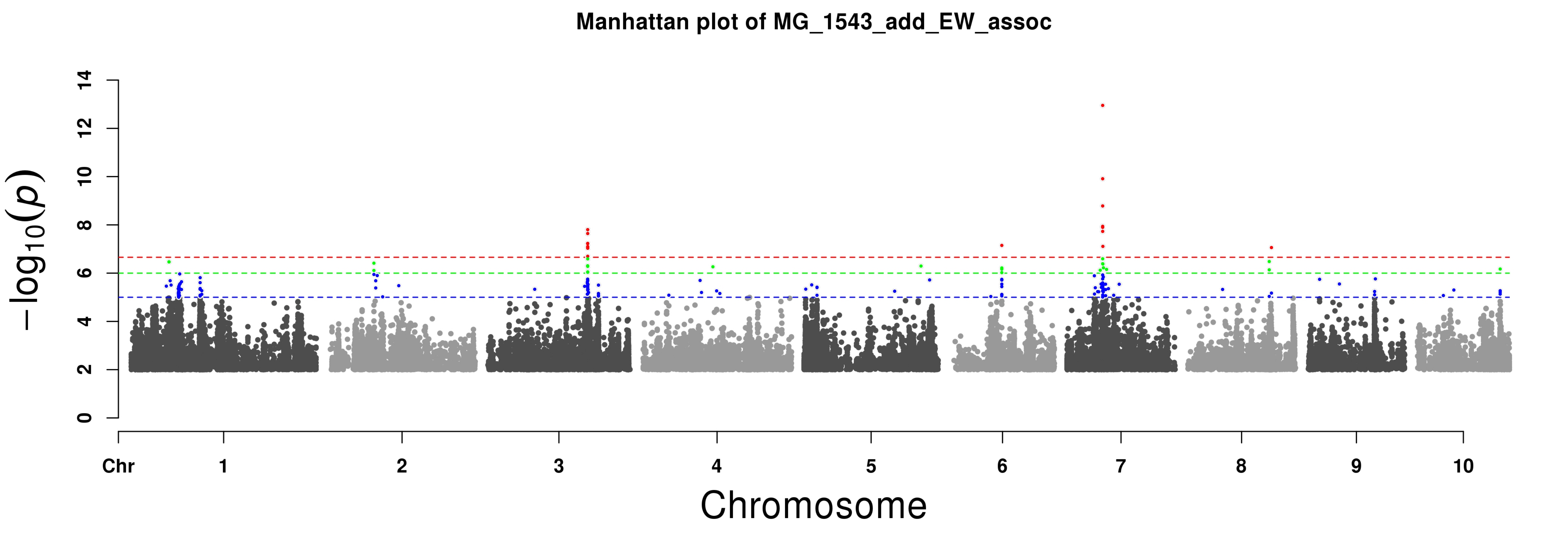

Manhattan plot of MG_1543_add_EW_assoc
$-\log_{10}(p)$
0
2
4
6
8
10
12
14
Chr
1
2
3
4
5
6
7
8
9
10
Chromosome

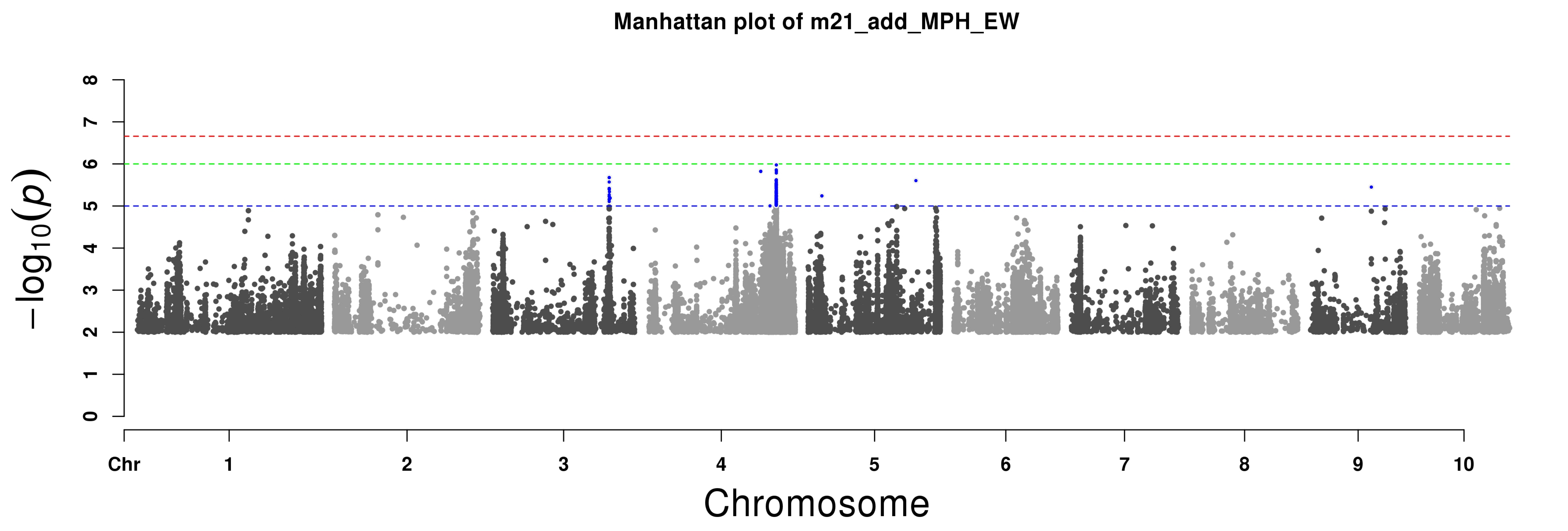

Manhattan plot of m21_add_MPH_EW
$-\log_{10}(p)$
0
1
2
3
4
5
6
7
8
Chr
1
2
3
4
5
6
7
8
9
10
Chromosome

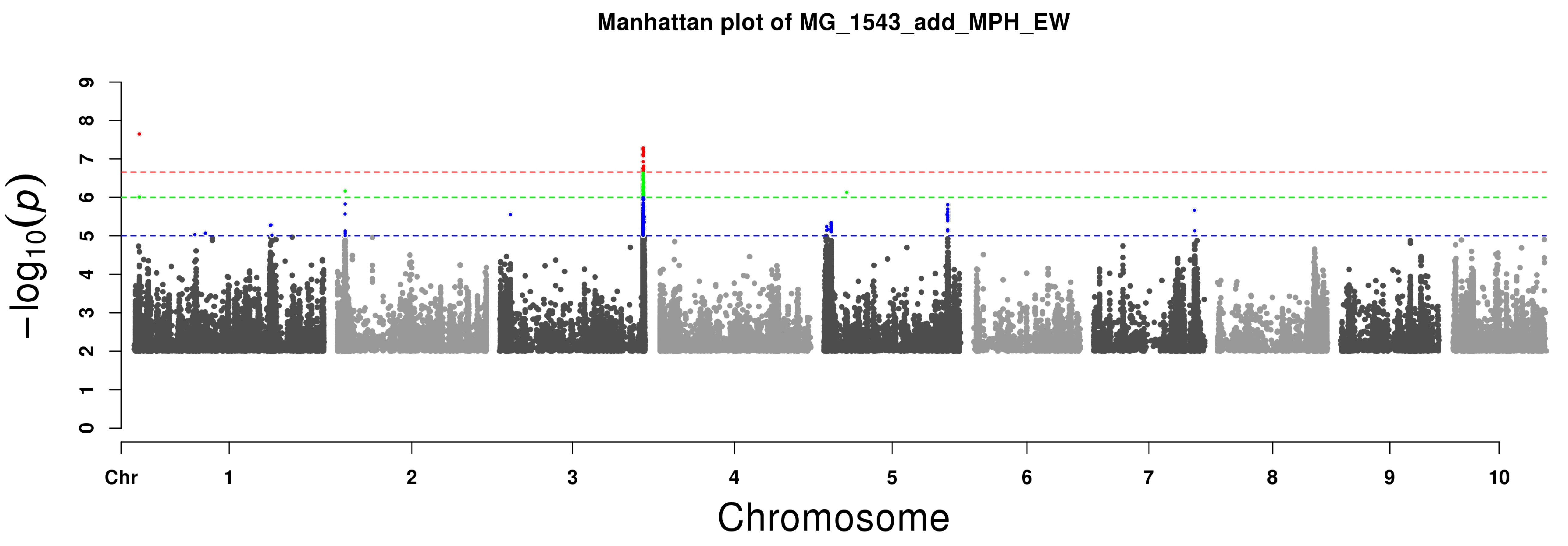

Manhattan plot of MG_1543_add_MPH_EW
$-\log_{10}(p)$
0
1
2
3
4
5
6
7
8
9
Chr
1
2
3
4
5
6
7
8
9
10
Chromosome

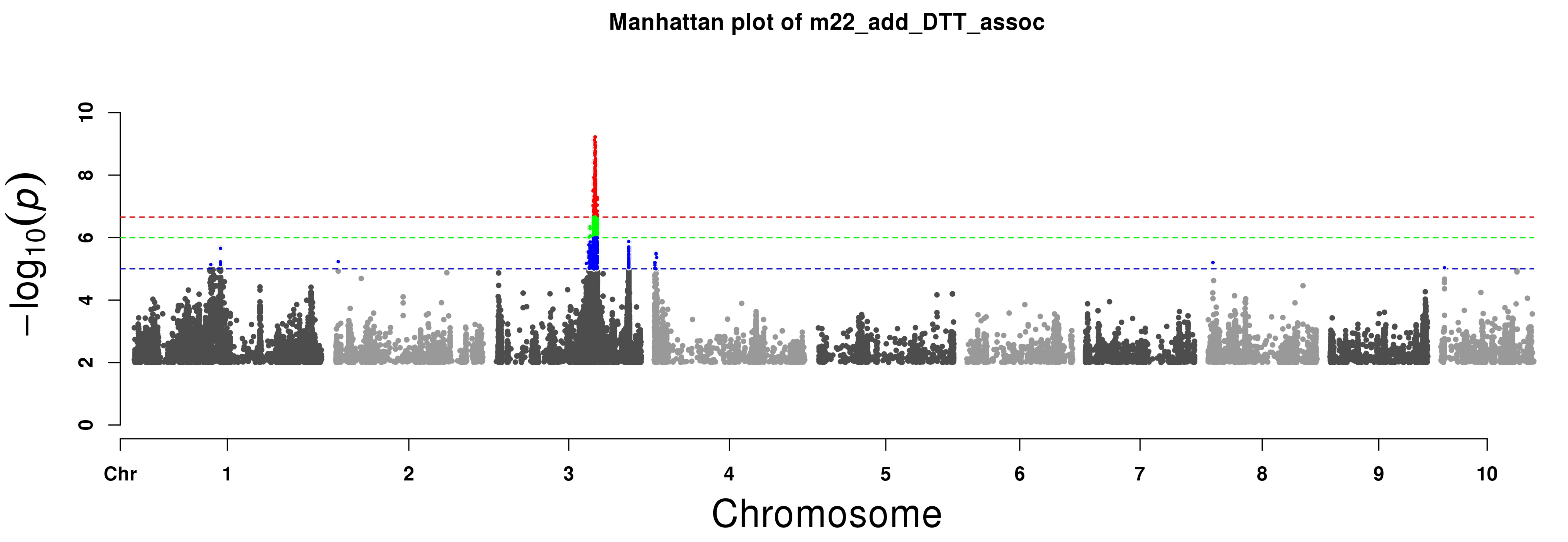

Manhattan plot of m22_add_DTT_assoc
$-\log_{10}(p)$
0
2
4
6
8
10
Chr
1
2
3
4
5
6
7
8
9
10
Chromosome

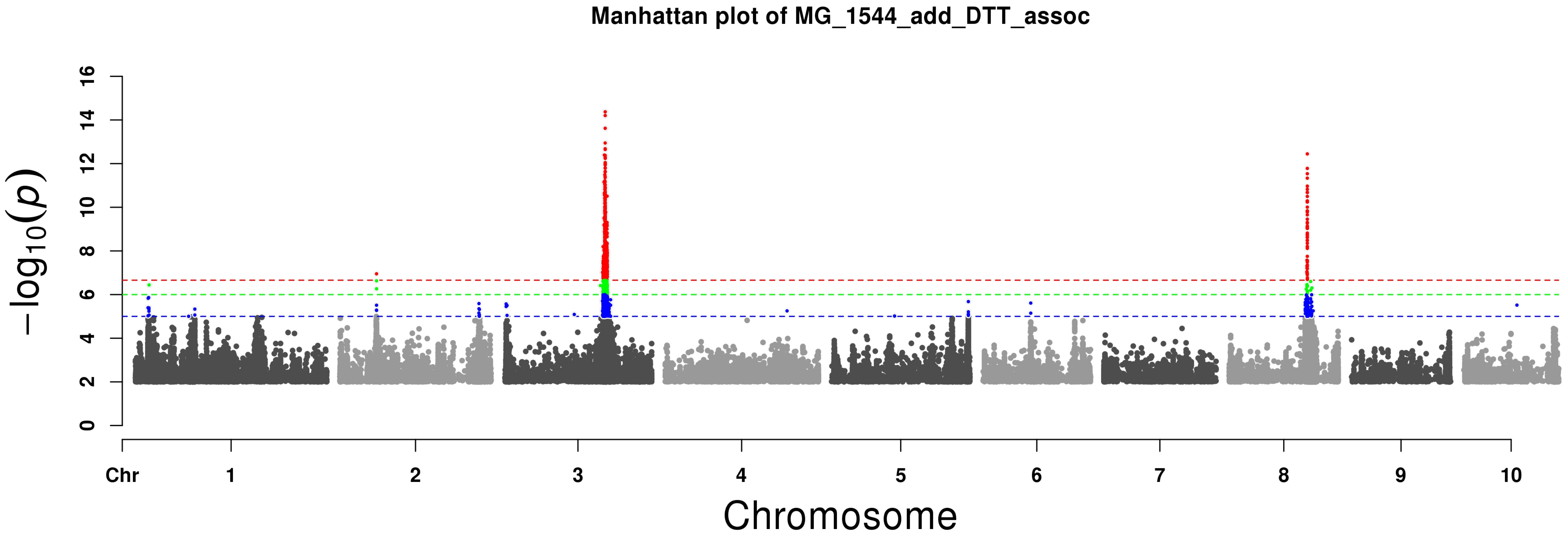

Manhattan plot of MG_1544_add_DTT_assoc
$-\log_{10}(p)$
0
2
4
6
8
10
12
14
16
Chr
1
2
3
4
5
6
7
8
9
10
Chromosome

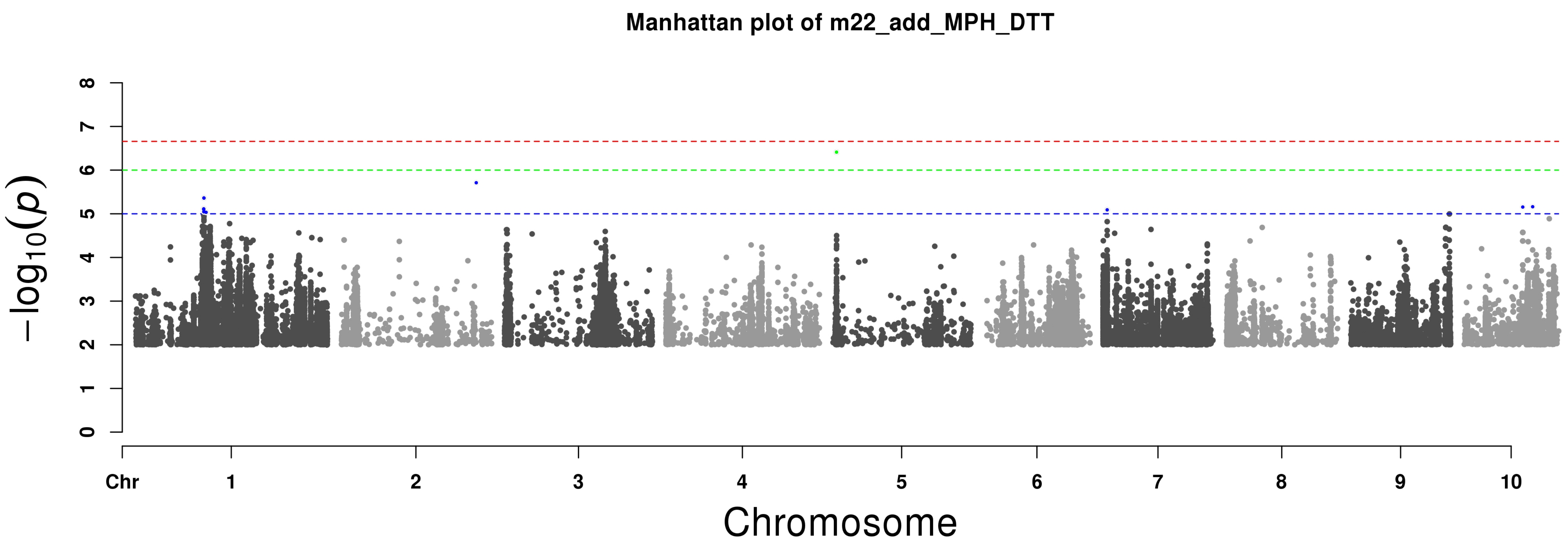

Manhattan plot of m22_add_MPH_DTT
$-\log_{10}(p)$
0
1
2
3
4
5
6
7
8
Chr
1
2
3
4
5
6
7
8
9
10
Chromosome

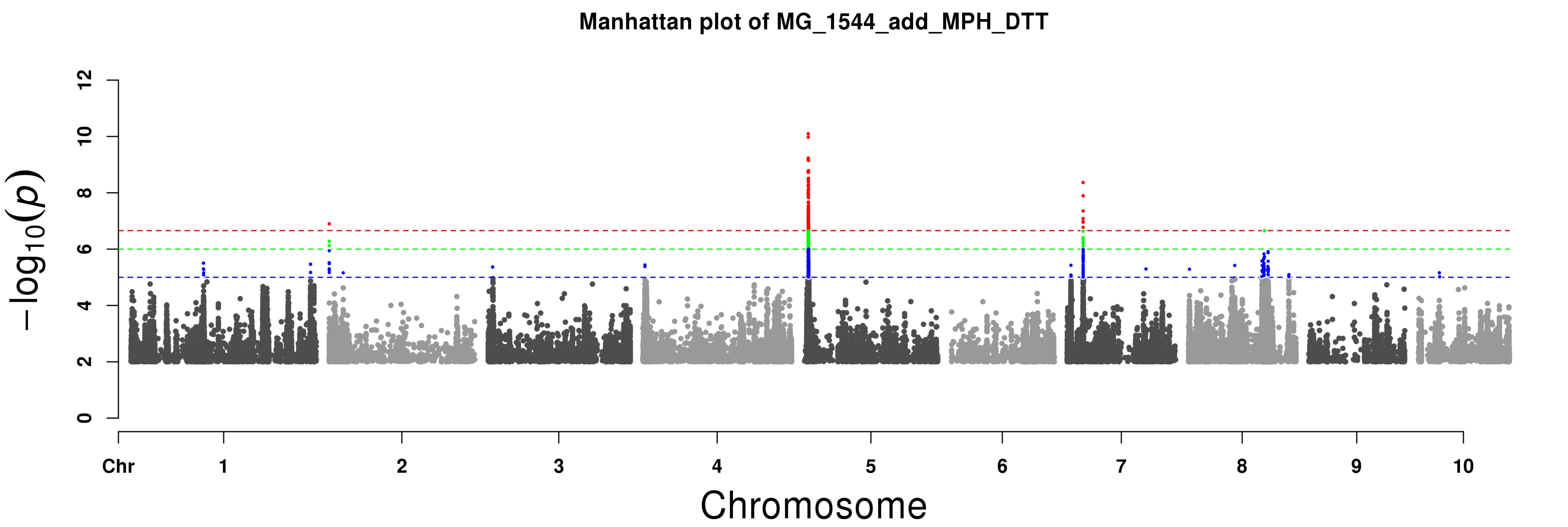
Manhattan plot of MG_1544_add_MPH_DTT
$-\log_{10}(p)$
0
2
4
6
8
10
12
Chr
1
2
3
4
5
6
7
8
9
10
Chromosome

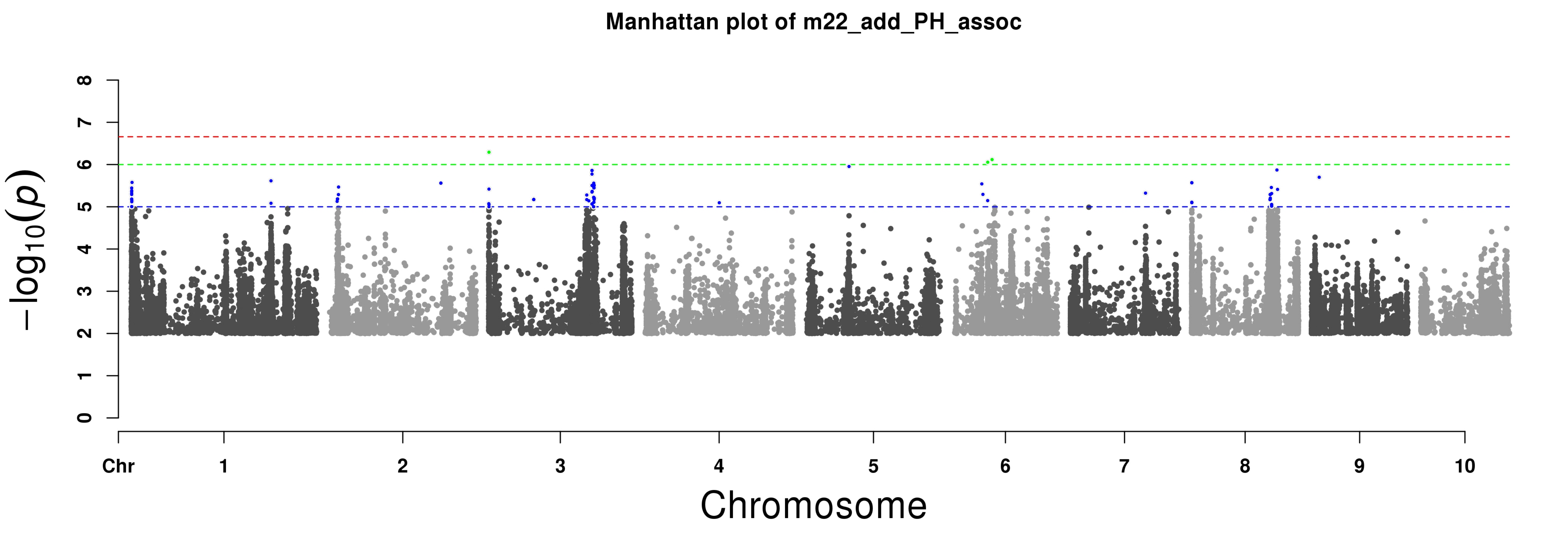
Manhattan plot of m22_add_PH_assoc
$-\log_{10}(p)$
0
1
2
3
4
5
6
7
8
Chr
1
2
3
4
5
6
7
8
9
10
Chromosome

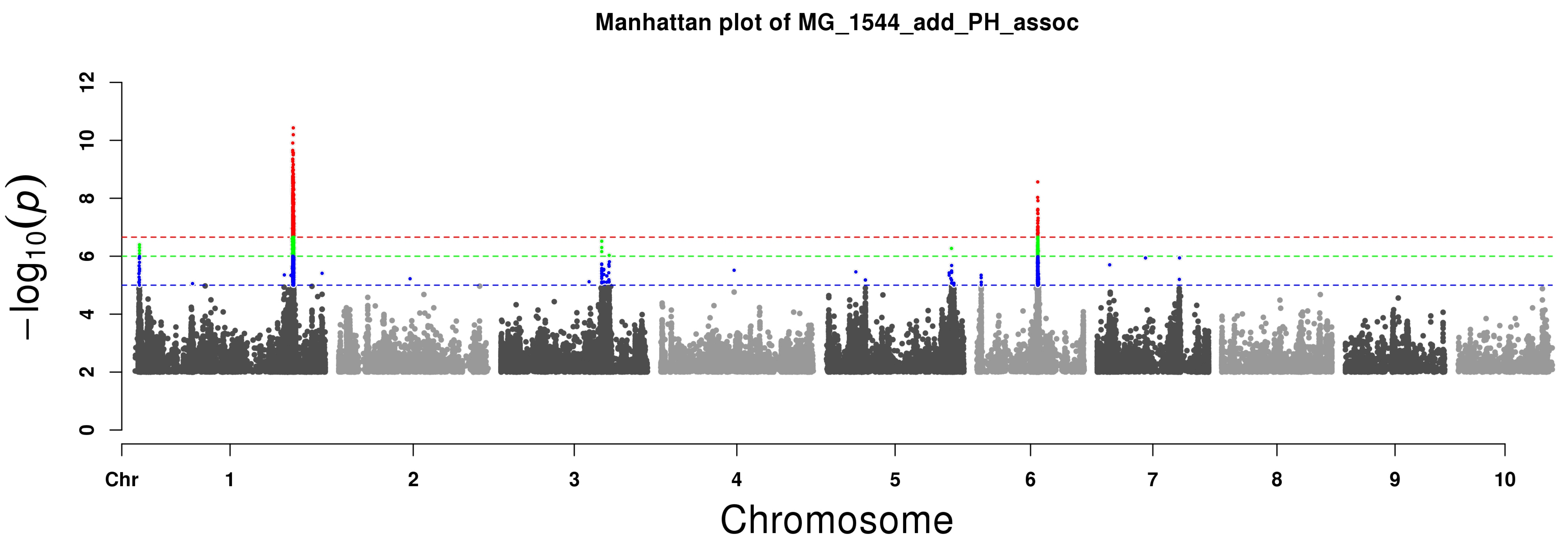
Manhattan plot of MG_1544_add_PH_assoc
$-\log_{10}(p)$
0
2
4
6
8
10
12
Chr
1
2
3
4
5
6
7
8
9
10
Chromosome

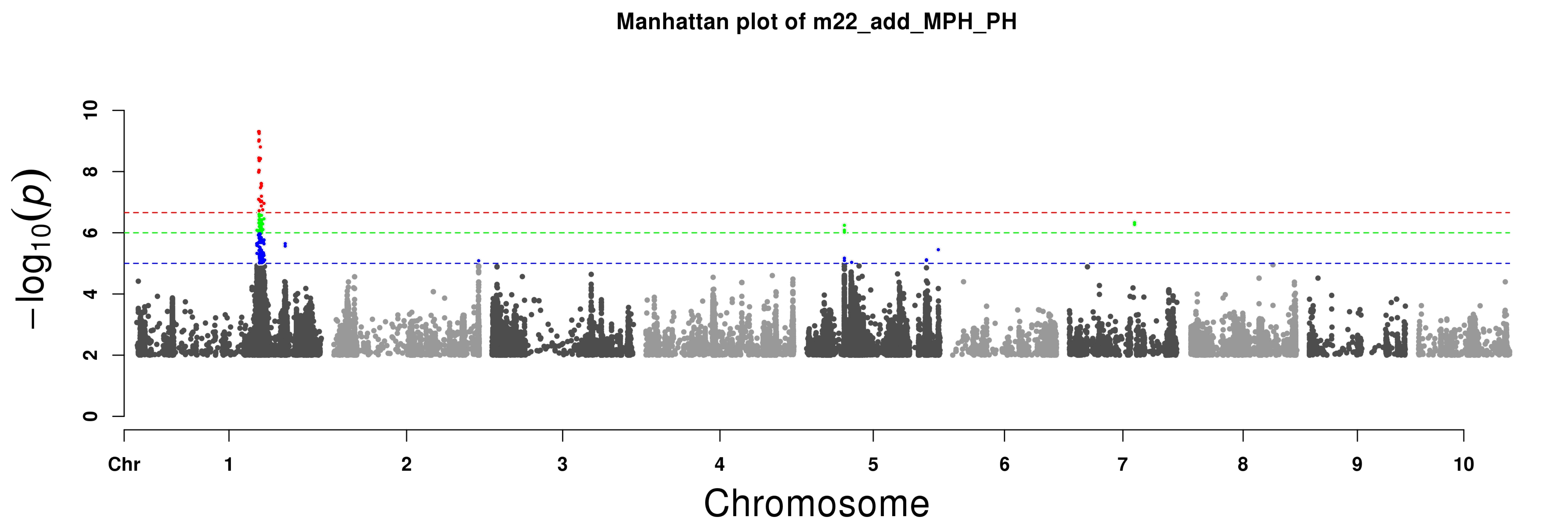

Manhattan plot of m22_add_MPH_PH
10
8
6
4
2
0
−log10(p)
Chr
1
2
3
4
5
6
7
8
9
10
Chromosome

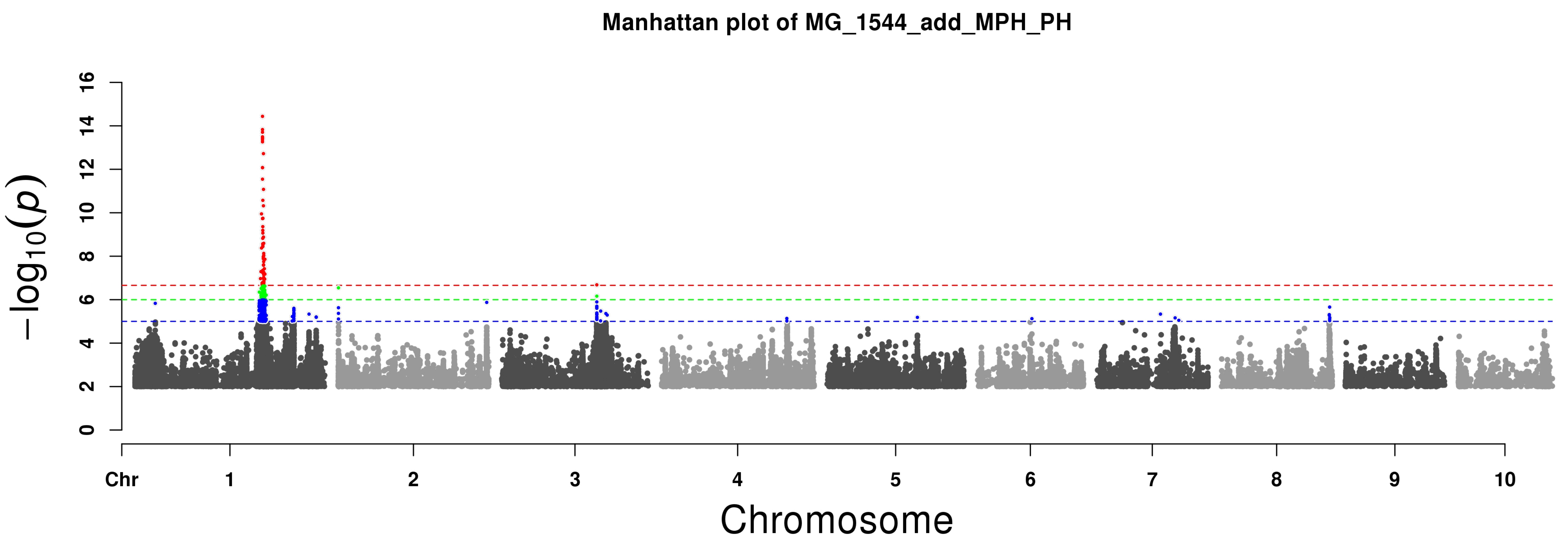
Manhattan plot of MG_1544_add_MPH_PH
$-\log_{10}(p)$
0
2
4
6
8
10
12
14
16
Chr
1
2
3
4
5
6
7
8
9
10
Chromosome

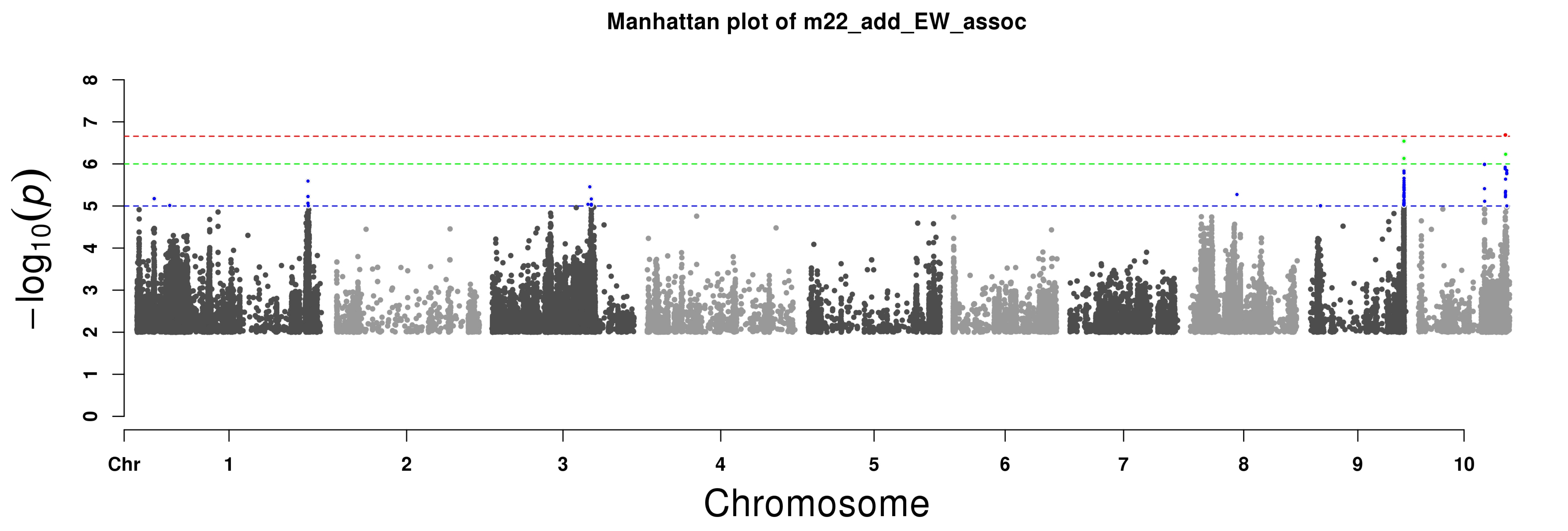
Manhattan plot of m22_add_EW_assoc
$-\log_{10}(p)$
0
1
2
3
4
5
6
7
8
Chr
1
2
3
4
5
6
7
8
9
10
Chromosome

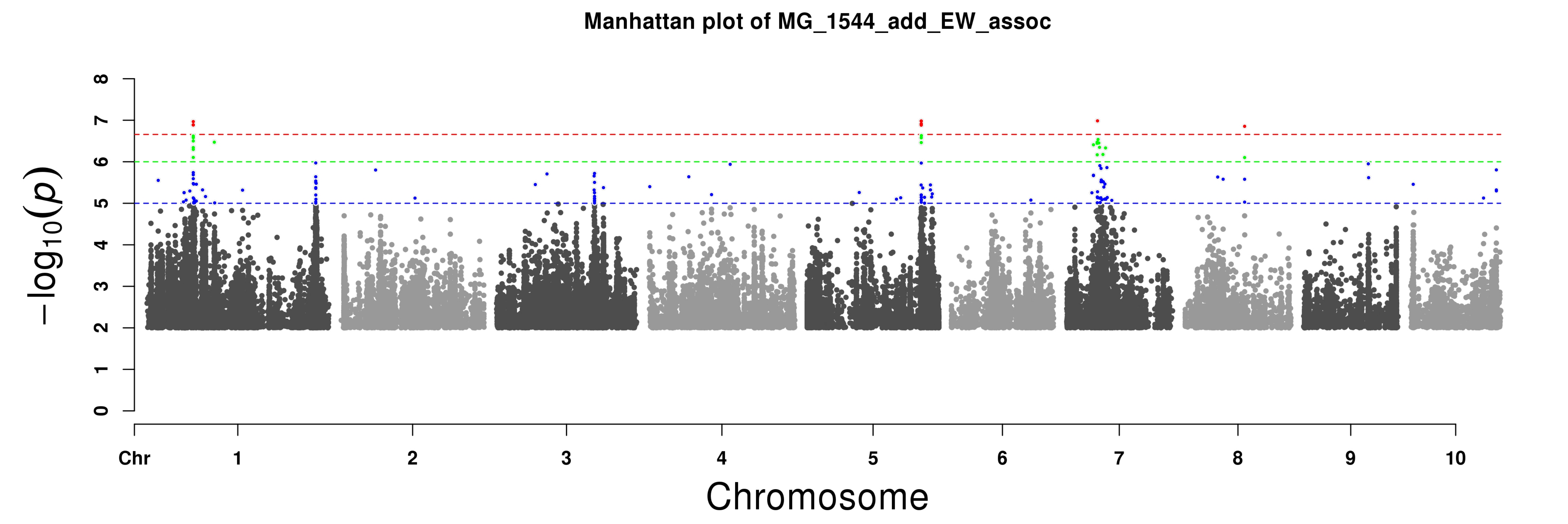
Manhattan plot of MG_1544_add_EW_assoc
$-\log_{10}(p)$
0
1
2
3
4
5
6
7
8
Chr
1
2
3
4
5
6
7
8
9
10
Chromosome

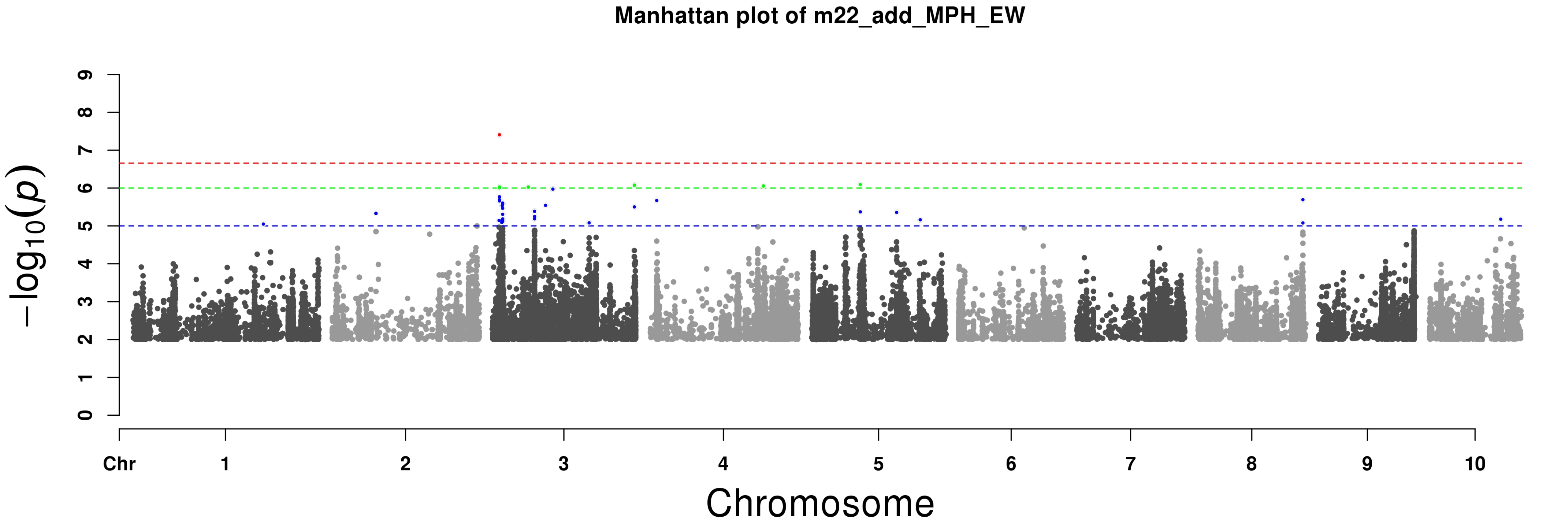
Manhattan plot of m22_add_MPH_EW
$-\log_{10}(p)$
0
1
2
3
4
5
6
7
8
9
Chr
1
2
3
4
5
6
7
8
9
10
Chromosome

Manhattan plot of MG_1544_add_MPH_EW

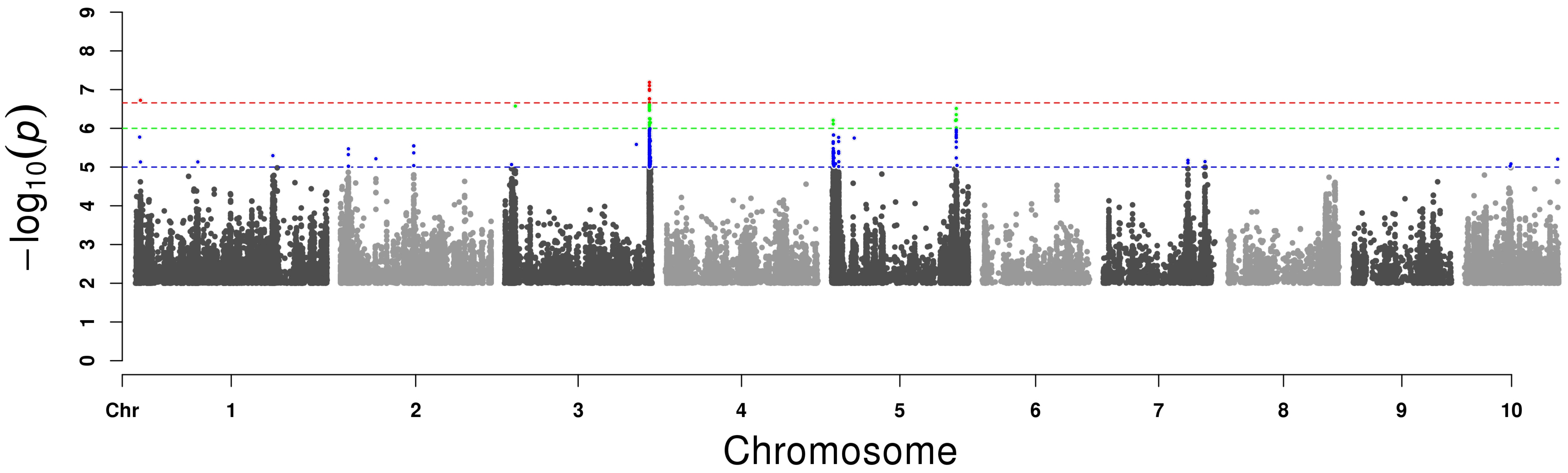

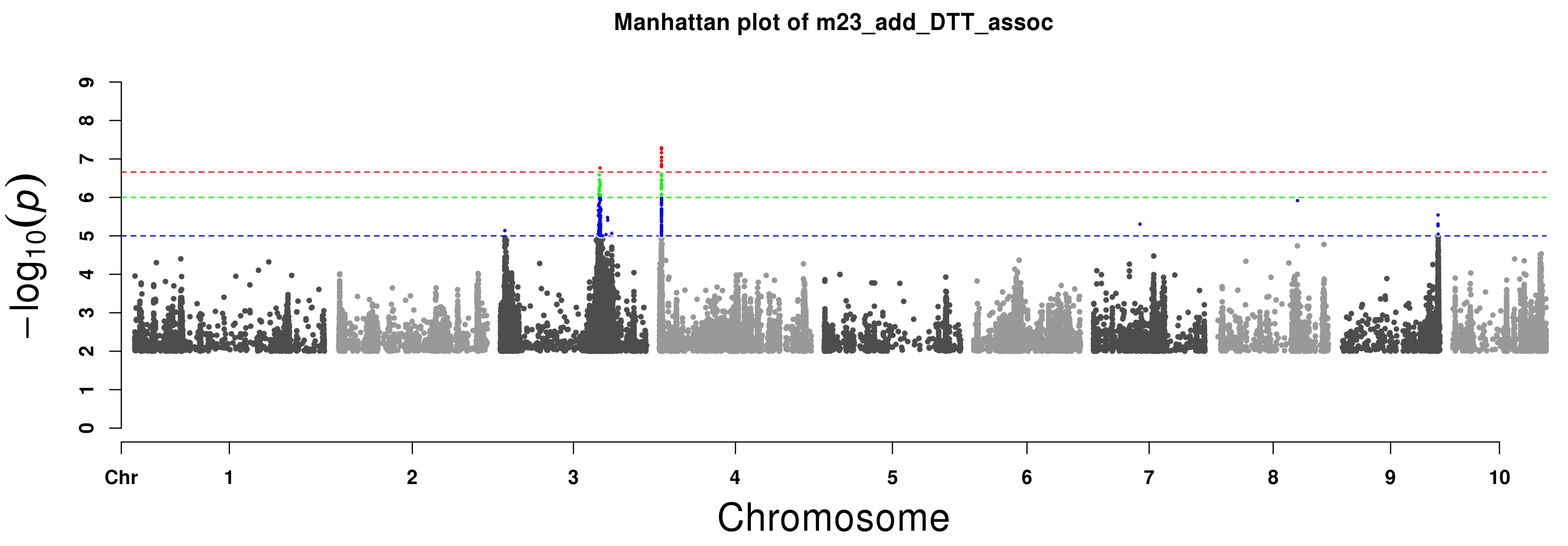

Manhattan plot of m23_add_DTT_assoc
$-\log_{10}(p)$
0
1
2
3
4
5
6
7
8
9
Chr
1
2
3
4
5
6
7
8
9
10
Chromosome

Manhattan plot of MG_1546_add_DTT_assoc

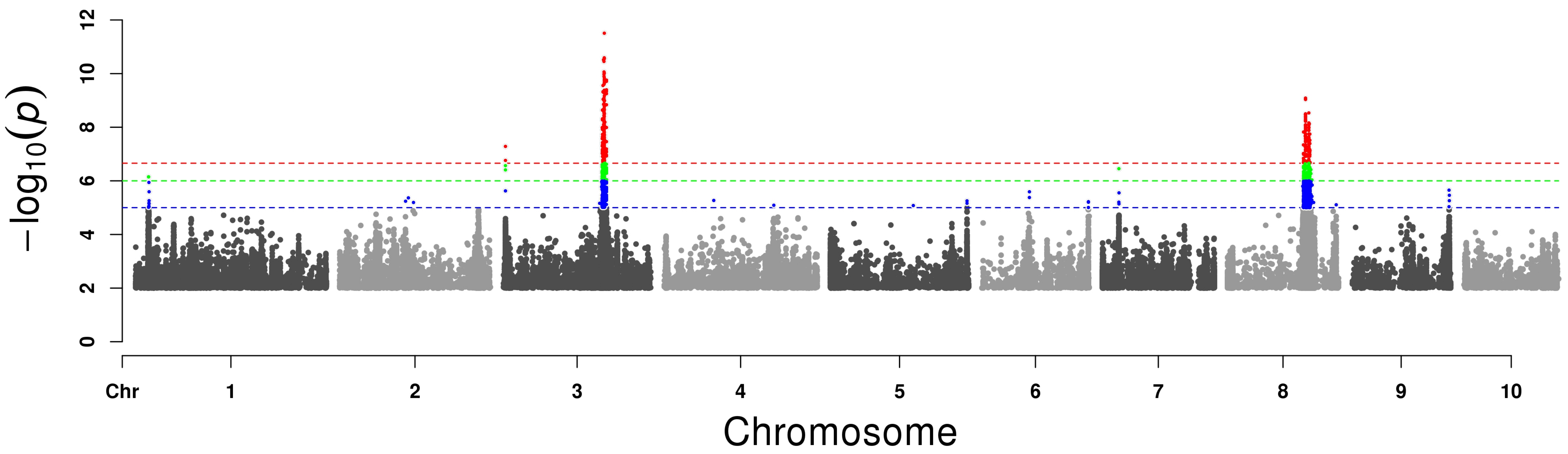

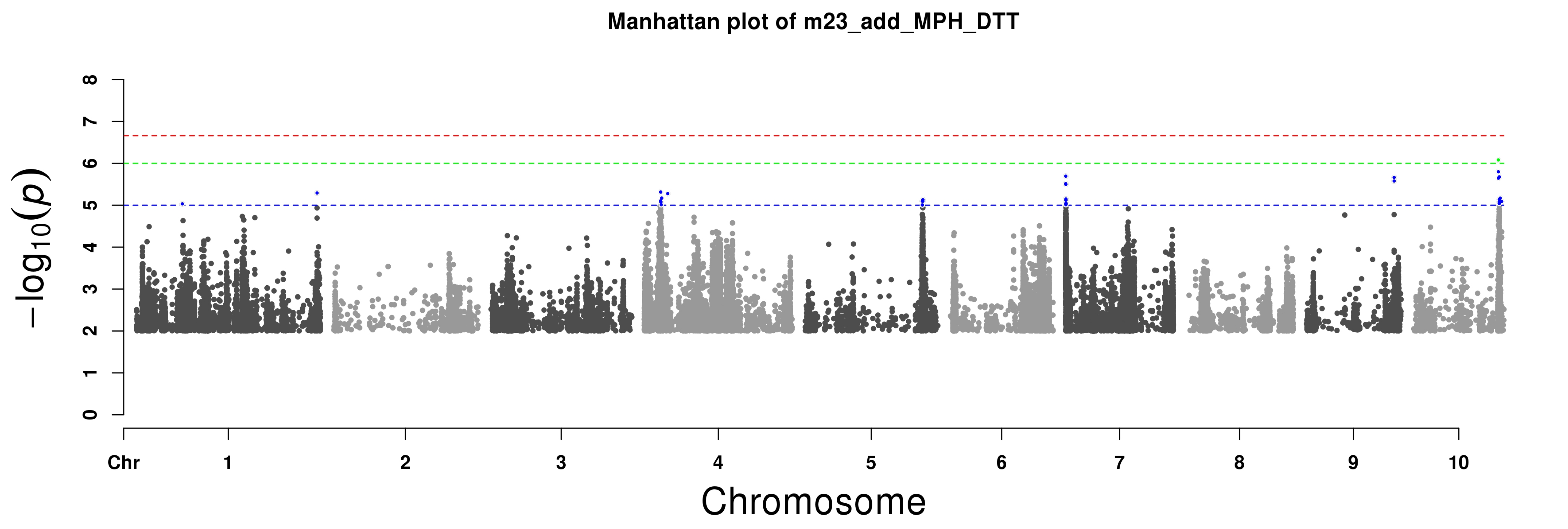
Manhattan plot of m23_add_MPH_DTT
$-\log_{10}(p)$
0
1
2
3
4
5
6
7
8
Chr
1
2
3
4
5
6
7
8
9
10
Chromosome

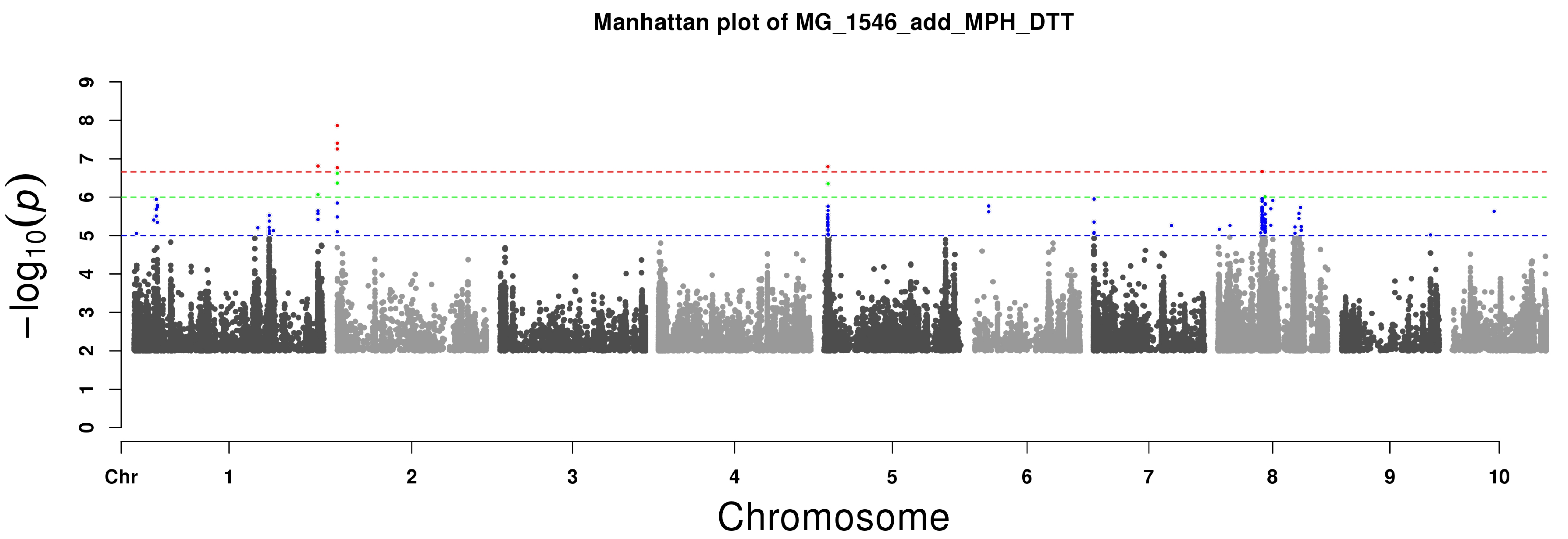
Manhattan plot of MG_1546_add_MPH_DTT
$-\log_{10}(p)$
0
1
2
3
4
5
6
7
8
9
Chr
1
2
3
4
5
6
7
8
9
10
Chromosome

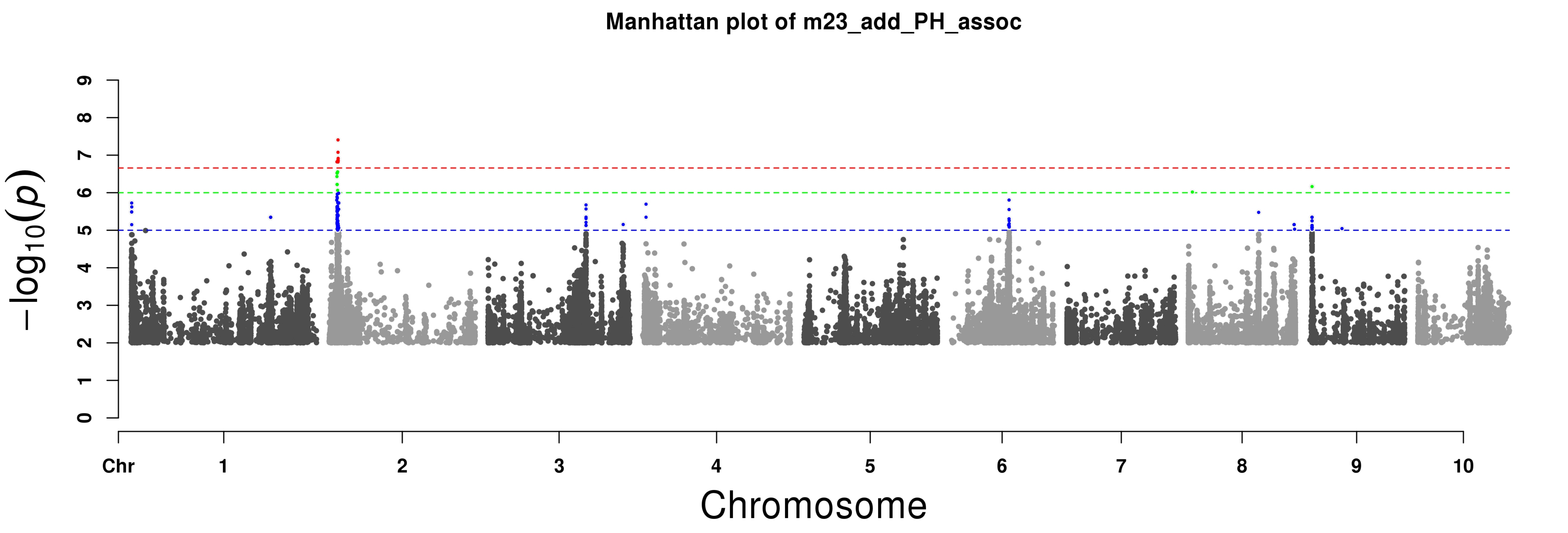

Manhattan plot of m23_add_PH_assoc
$-\log_{10}(p)$
0
1
2
3
4
5
6
7
8
9
Chr
1
2
3
4
5
6
7
8
9
10
Chromosome

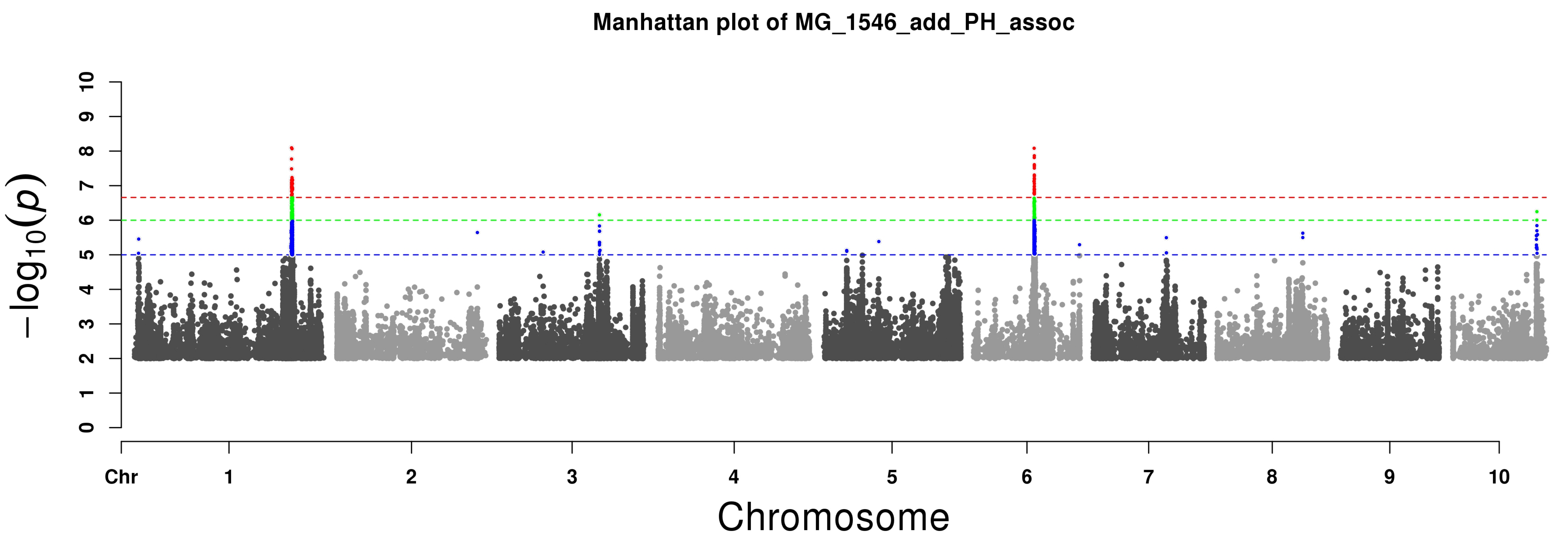

Manhattan plot of MG_1546_add_PH_assoc
$-\log_{10}(p)$
0
1
2
3
4
5
6
7
8
9
10
Chr
1
2
3
4
5
6
7
8
9
10
Chromosome

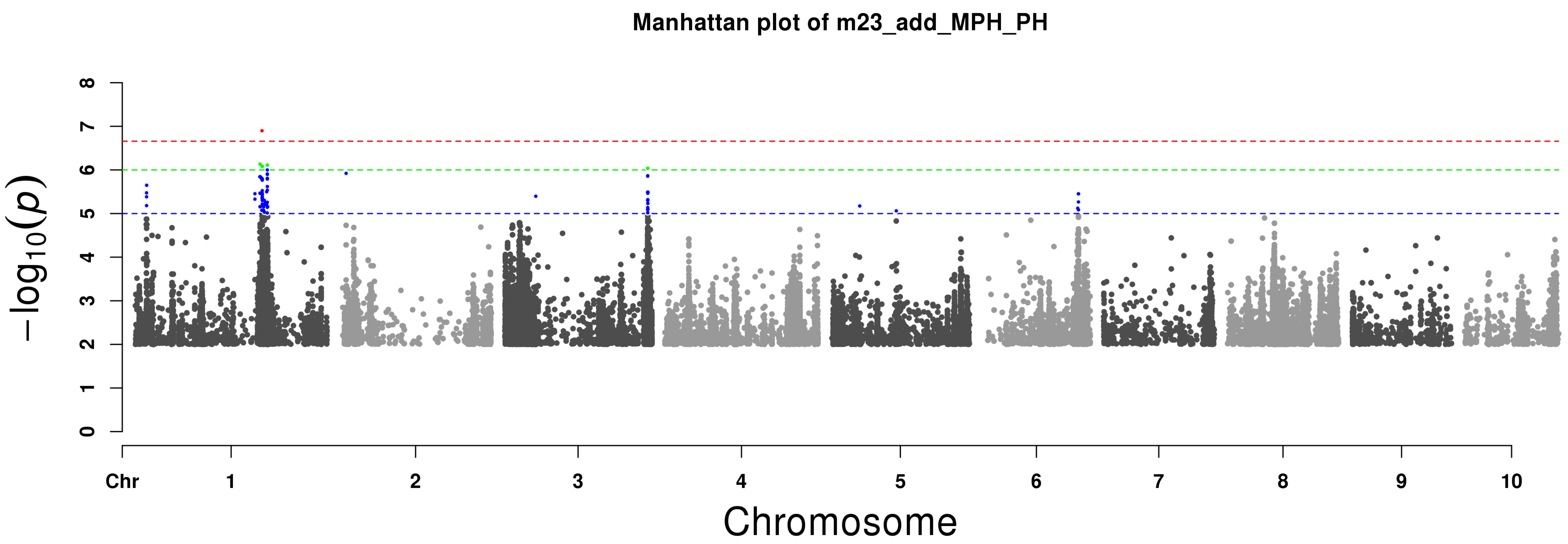

Manhattan plot of m23_add_MPH_PH
$-\log_{10}(p)$
0
1
2
3
4
5
6
7
8
Chr
1
2
3
4
5
6
7
8
9
10
Chromosome

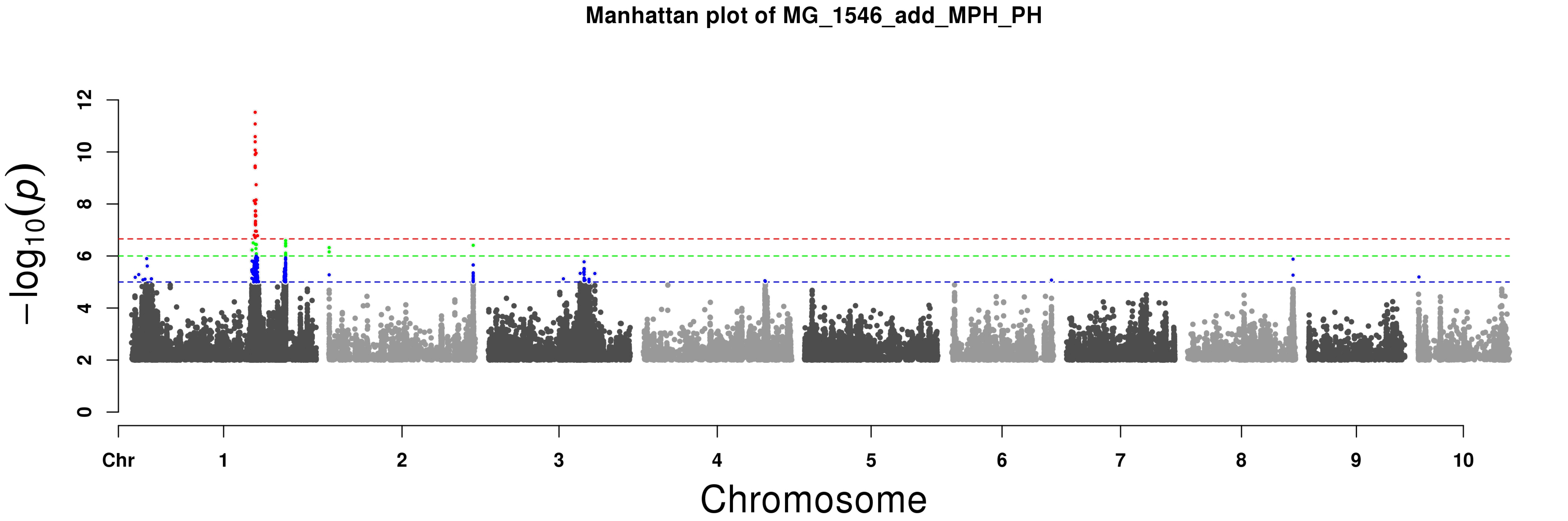

Manhattan plot of MG_1546_add_MPH_PH
−log10(p)
0
2
4
6
8
10
12
Chr
1
2
3
4
5
6
7
8
9
10
Chromosome

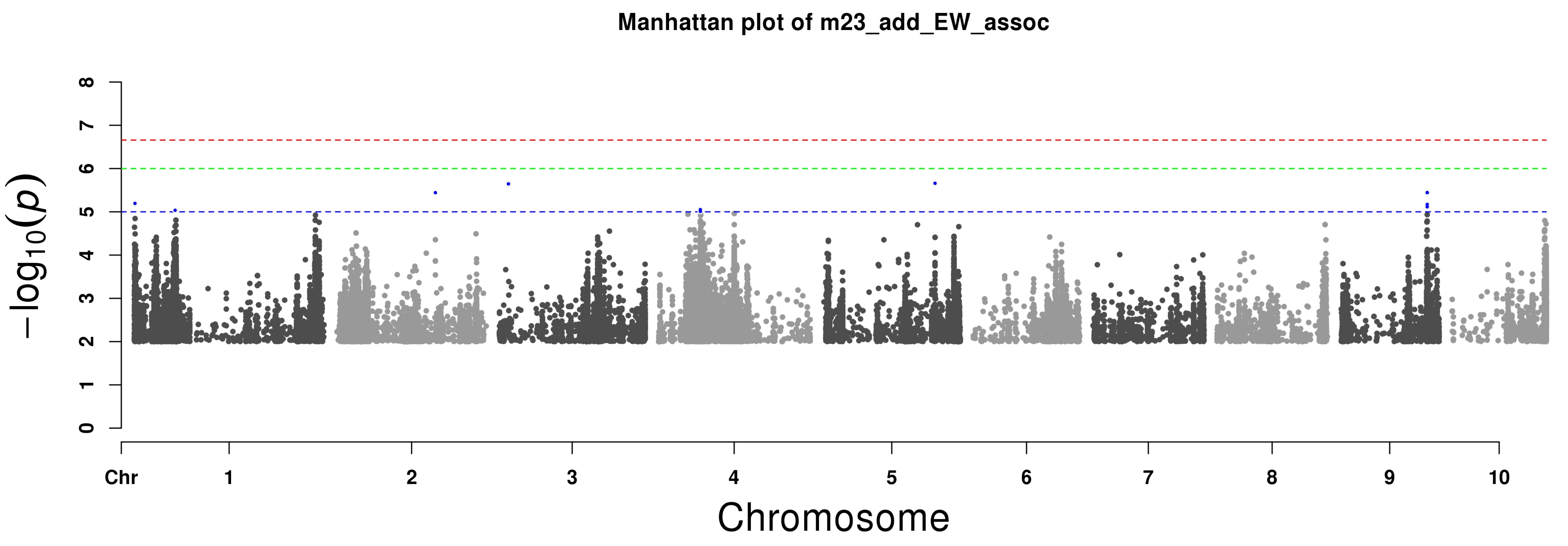

Manhattan plot of m23_add_EW_assoc
$-\log_{10}(p)$
0
1
2
3
4
5
6
7
8
Chr
1
2
3
4
5
6
7
8
9
10
Chromosome

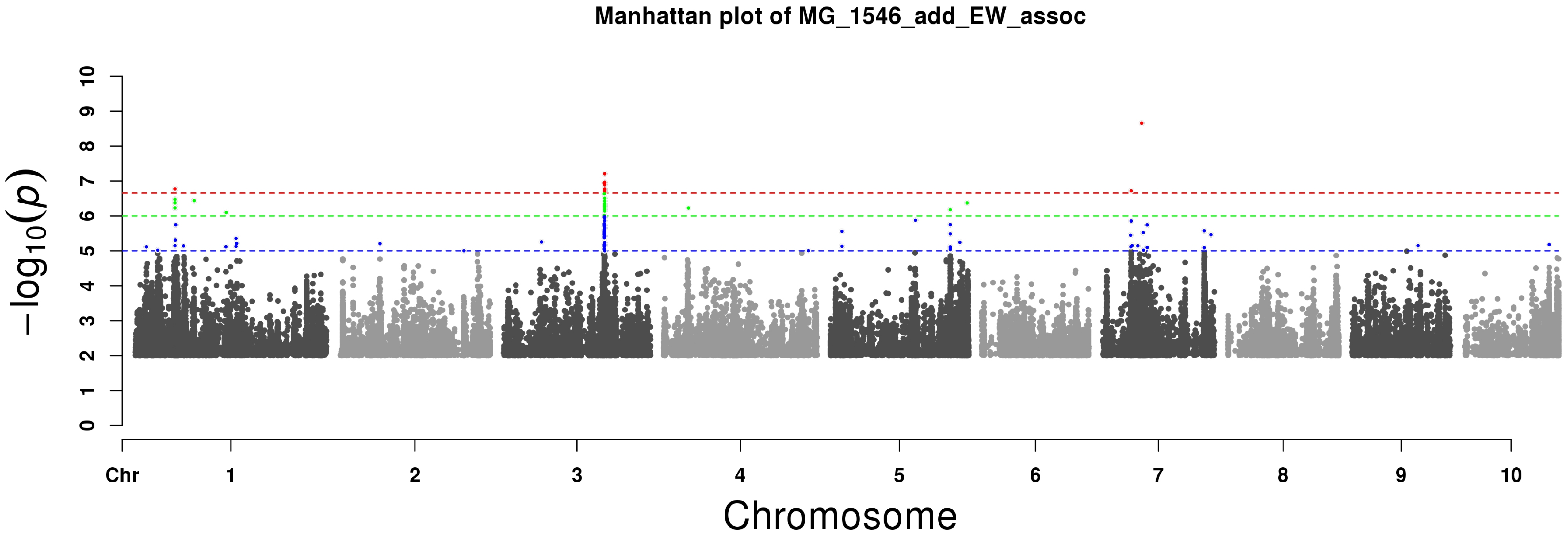

Manhattan plot of MG_1546_add_EW_assoc
$-\log_{10}(p)$
0
1
2
3
4
5
6
7
8
9
10
Chr
1
2
3
4
5
6
7
8
9
10
Chromosome

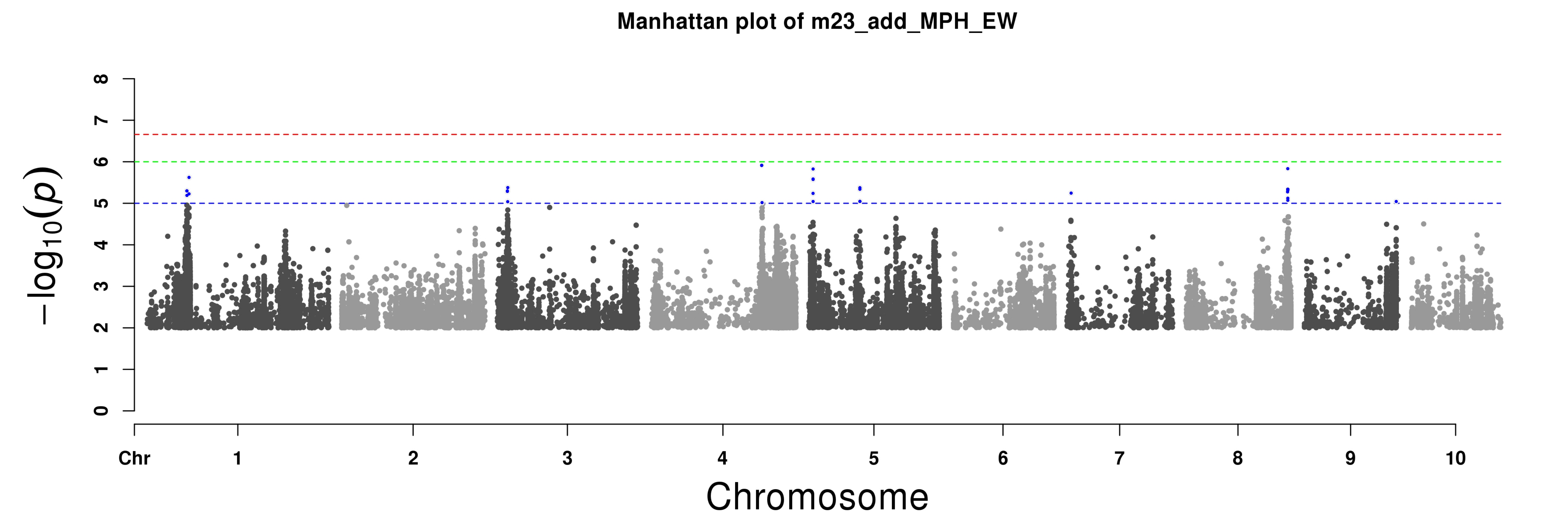

Manhattan plot of m23_add_MPH_EW
$-\log_{10}(p)$
0
1
2
3
4
5
6
7
8
Chr
1
2
3
4
5
6
7
8
9
10
Chromosome

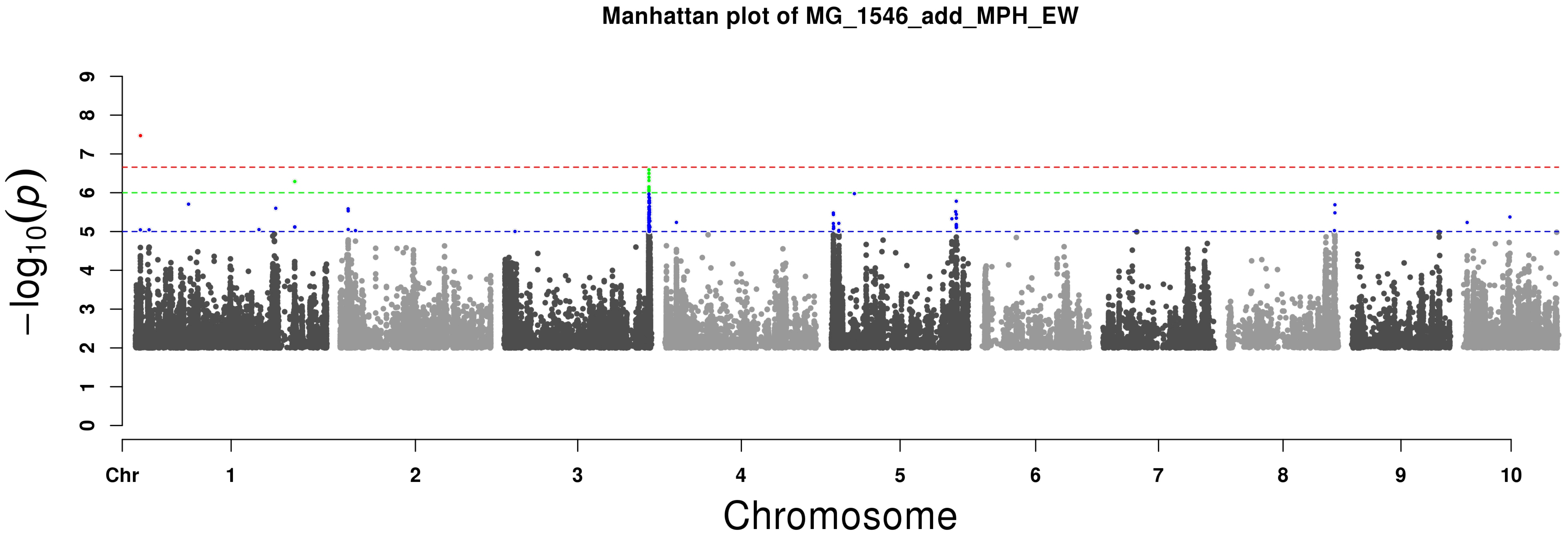

Manhattan plot of MG_1546_add_MPH_EW
$-\log_{10}(p)$
0
1
2
3
4
5
6
7
8
9
Chr
1
2
3
4
5
6
7
8
9
10
Chromosome

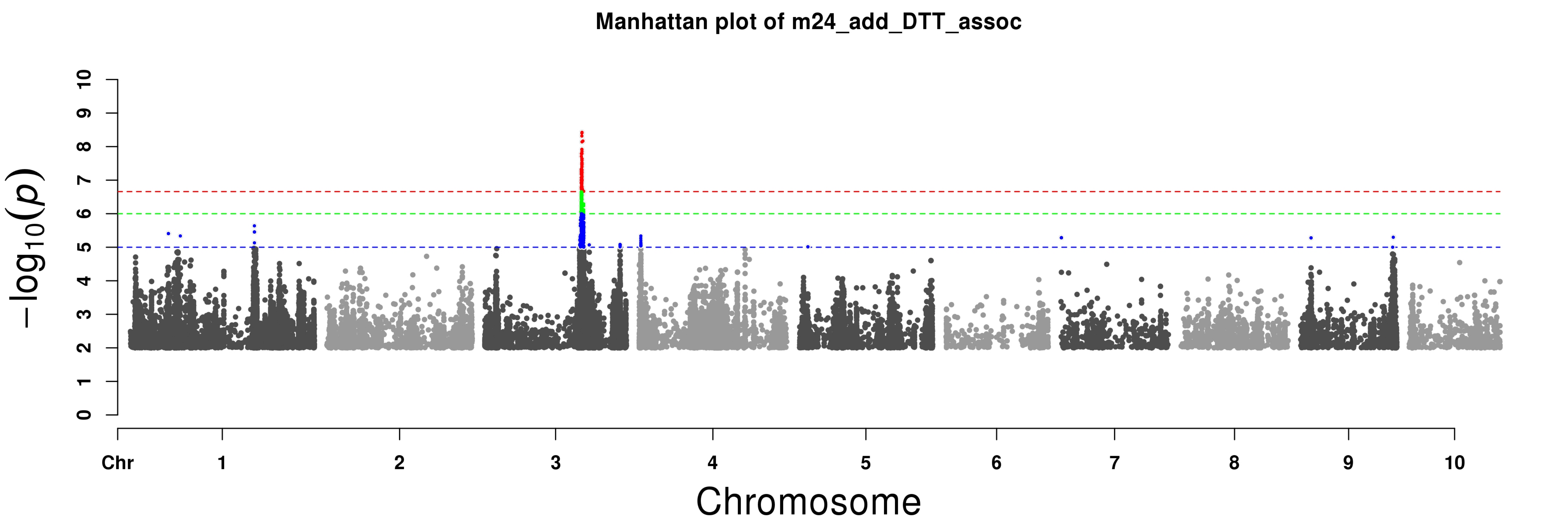

Manhattan plot of m24_add_DTT_assoc
$-\log_{10}(p)$
0
1
2
3
4
5
6
7
8
9
10
Chr
1
2
3
4
5
6
7
8
9
10
Chromosome

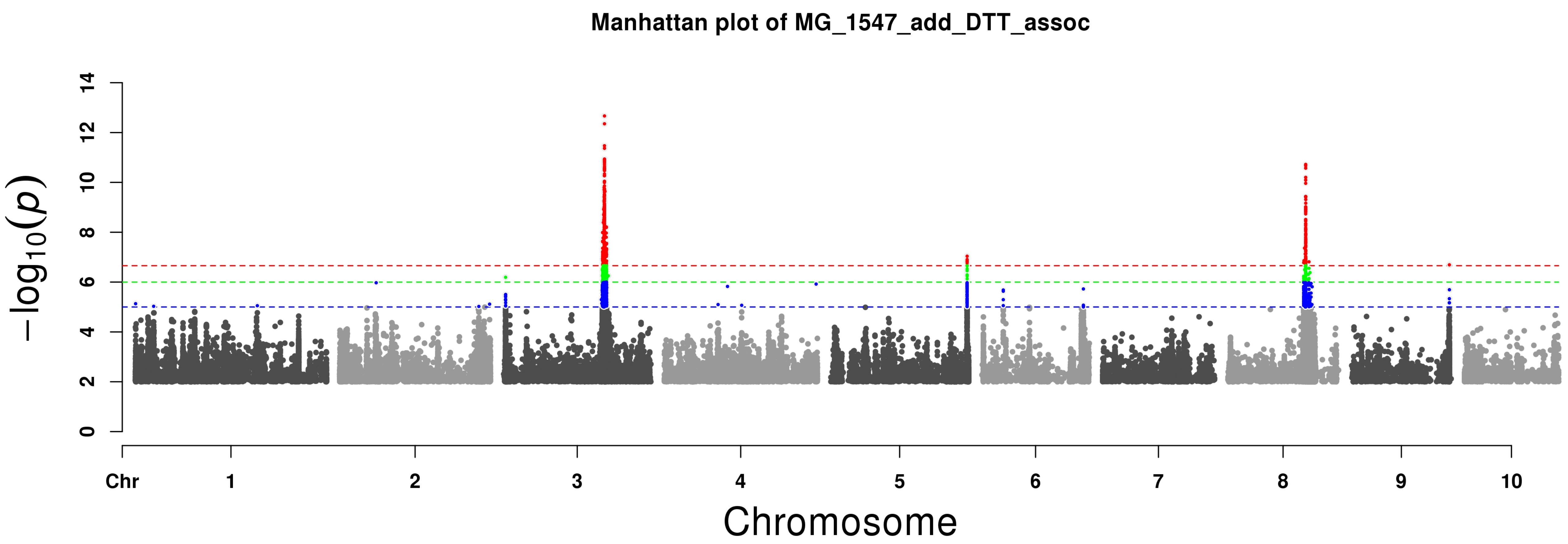
Manhattan plot of MG_1547_add_DTT_assoc
$-\log_{10}(p)$
0
2
4
6
8
10
12
14
Chr
1
2
3
4
5
6
7
8
9
10
Chromosome

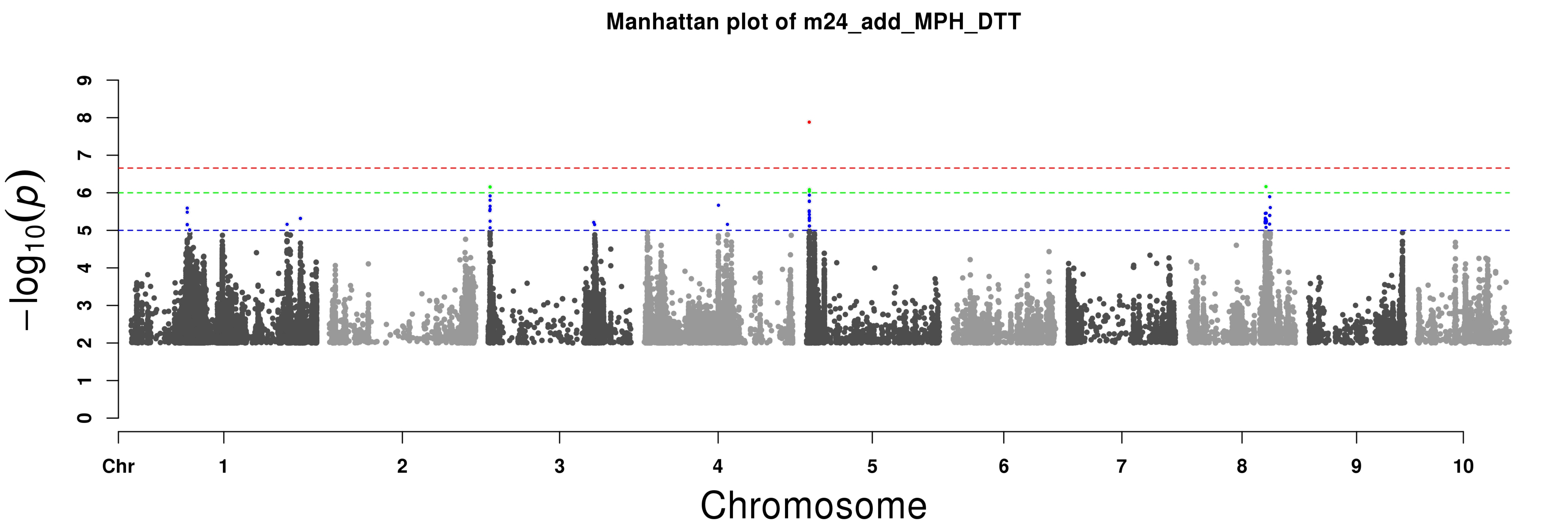
Manhattan plot of m24_add_MPH_DTT
$-\log_{10}(p)$
0
1
2
3
4
5
6
7
8
9
Chr
1
2
3
4
5
6
7
8
9
10
Chromosome

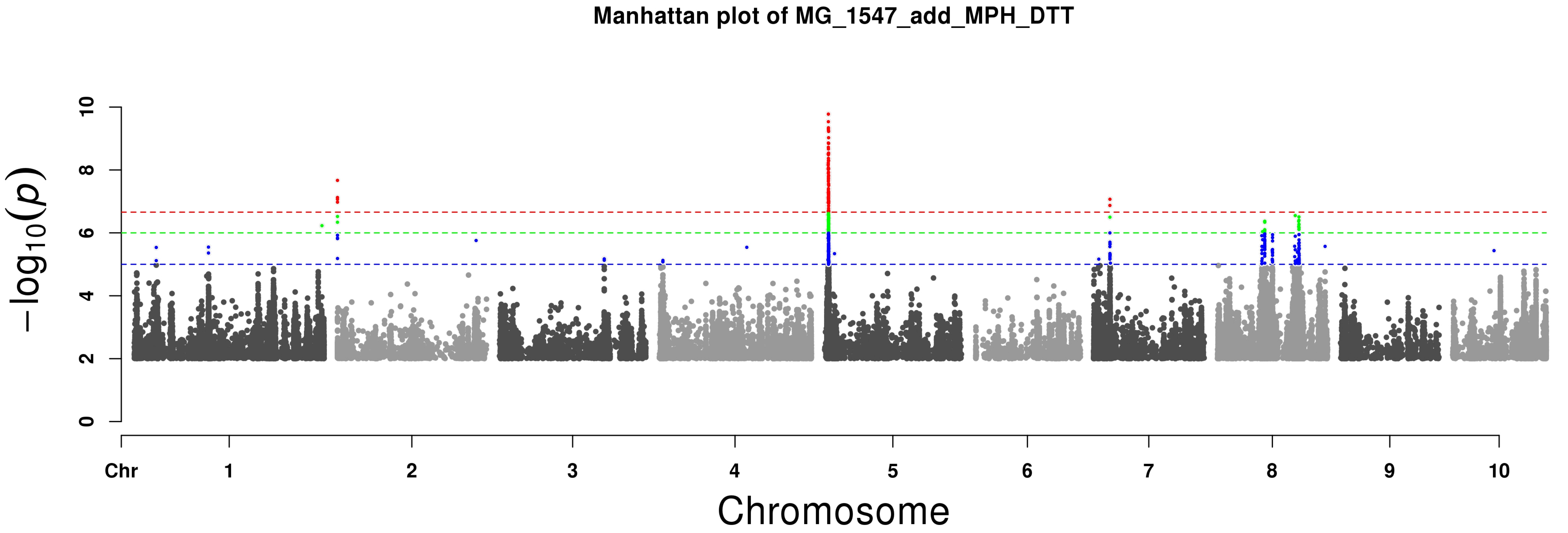

Manhattan plot of MG_1547_add_MPH_DTT
$-\log_{10}(p)$
0
2
4
6
8
10
Chr
1
2
3
4
5
6
7
8
9
10
Chromosome

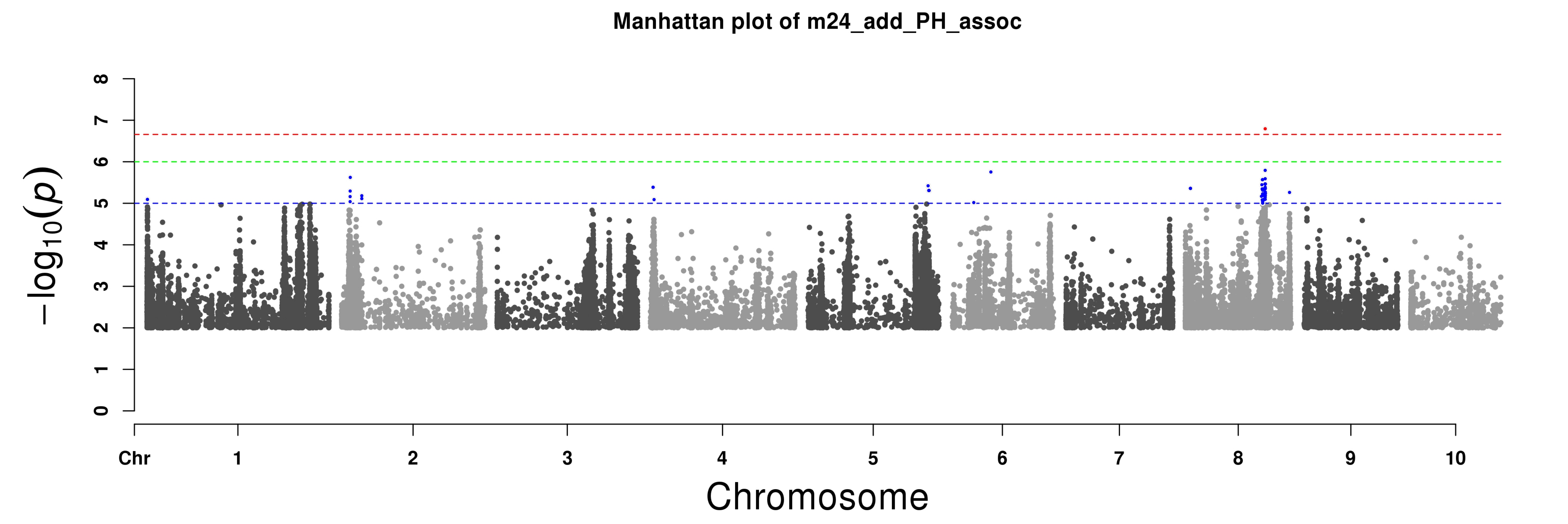

Manhattan plot of m24_add_PH_assoc
$-\log_{10}(p)$
0
1
2
3
4
5
6
7
8
Chr
1
2
3
4
5
6
7
8
9
10
Chromosome

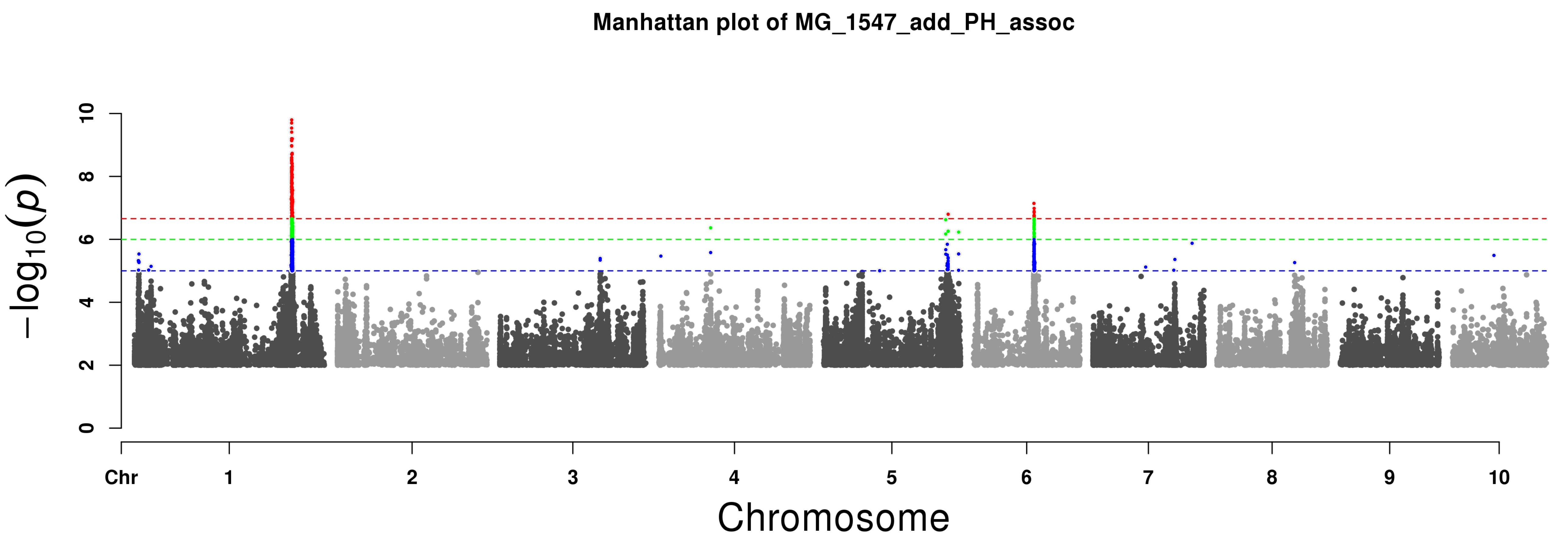

Manhattan plot of MG_1547_add_PH_assoc
$-\log_{10}(p)$
0
2
4
6
8
10
Chr
1
2
3
4
5
6
7
8
9
10
Chromosome

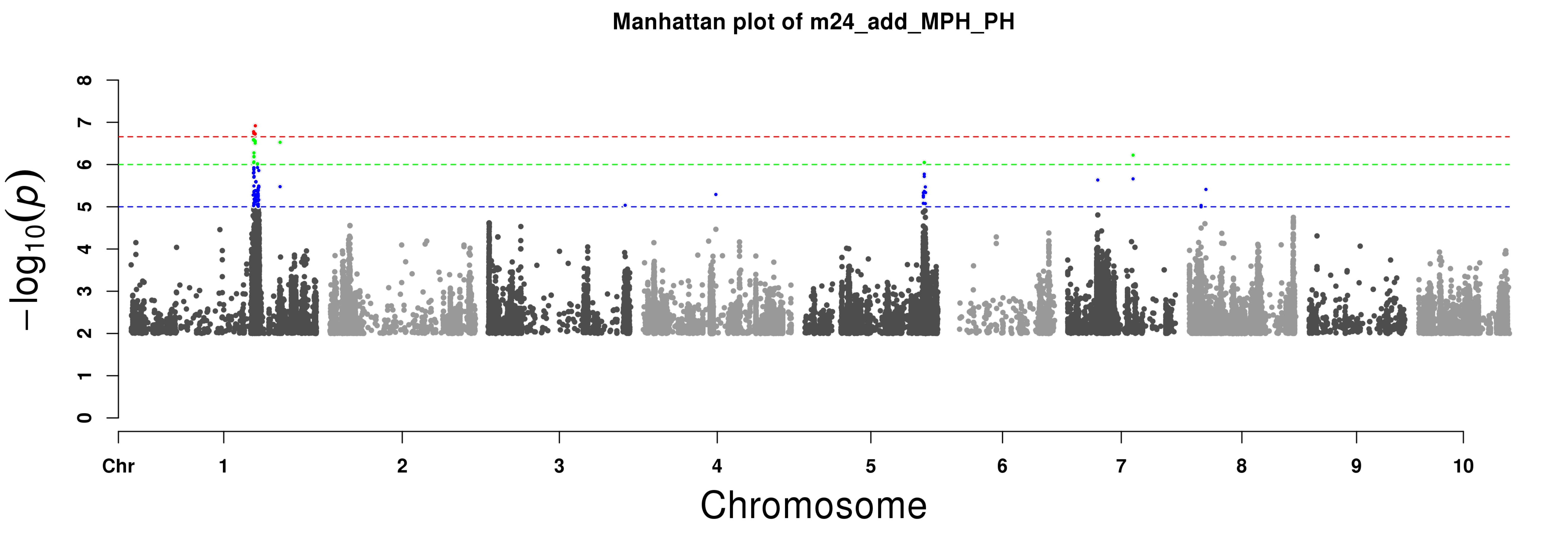
Manhattan plot of m24_add_MPH_PH
$-\log_{10}(p)$
0
1
2
3
4
5
6
7
8
Chr
1
2
3
4
5
6
7
8
9
10
Chromosome

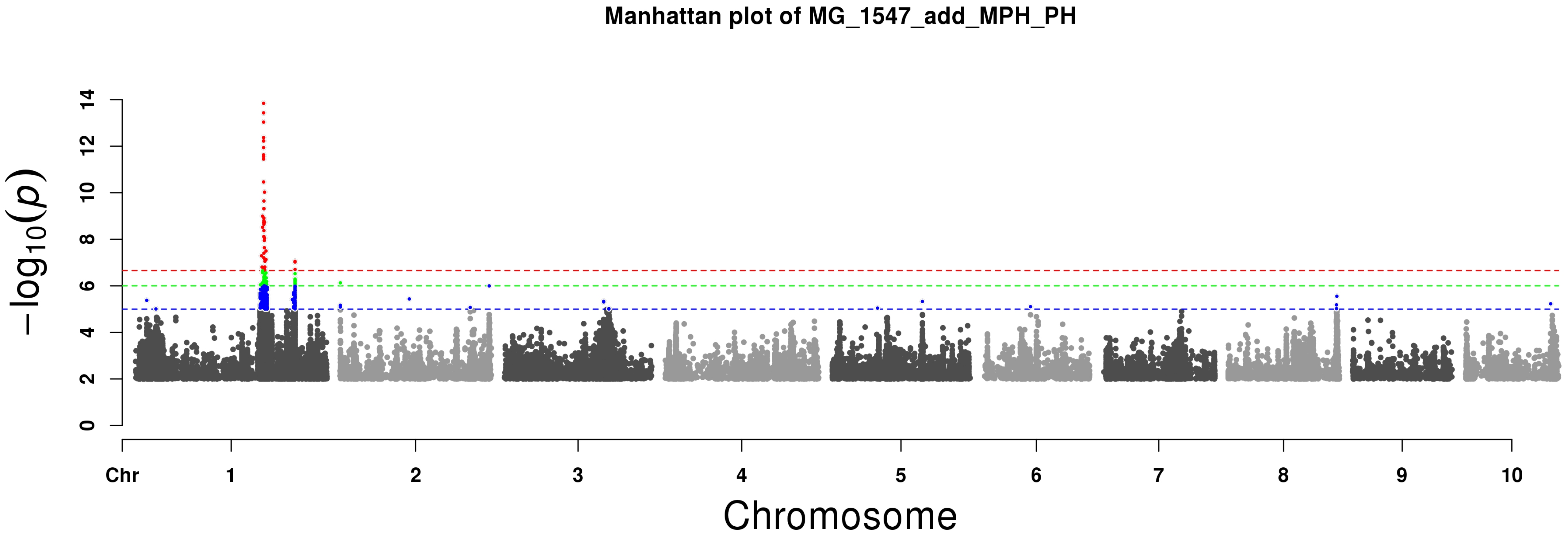

Manhattan plot of MG_1547_add_MPH_PH
$-\log_{10}(p)$
0
2
4
6
8
10
12
14
Chr
1
2
3
4
5
6
7
8
9
10
Chromosome

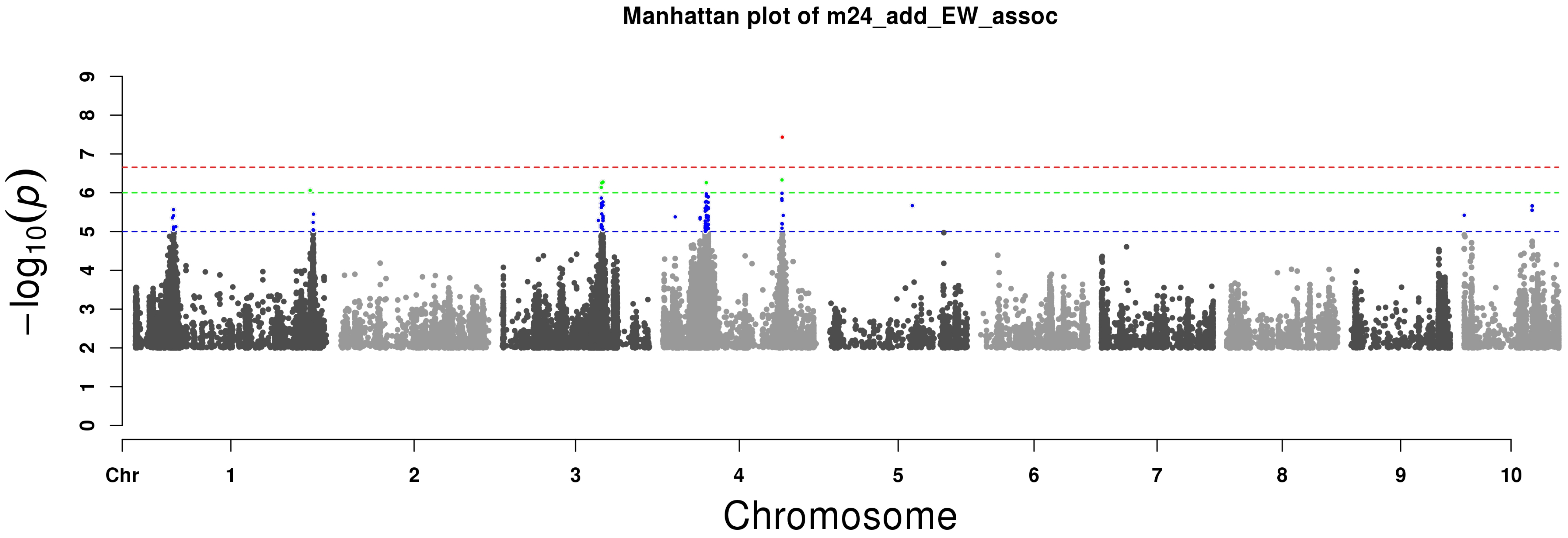

Manhattan plot of m24_add_EW_assoc
$-\log_{10}(p)$
0
1
2
3
4
5
6
7
8
9
Chr
1
2
3
4
5
6
7
8
9
10
Chromosome

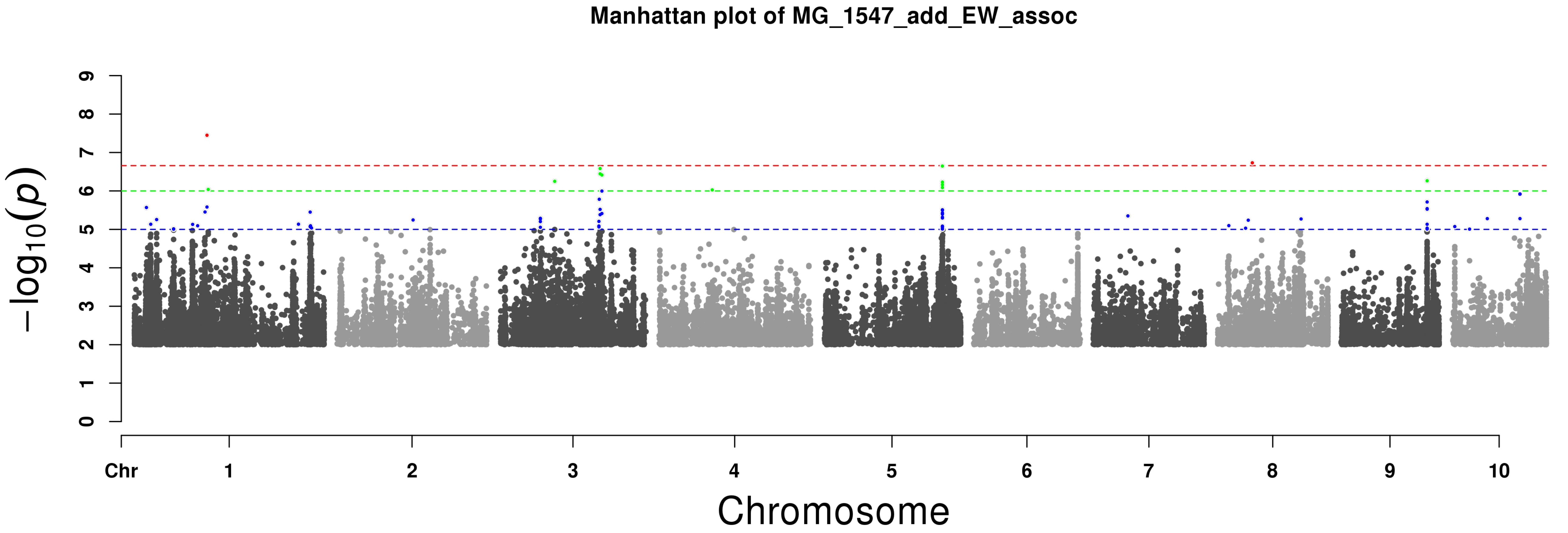
Manhattan plot of MG_1547_add_EW_assoc
$-\log_{10}(p)$
0
1
2
3
4
5
6
7
8
9
Chr
1
2
3
4
5
6
7
8
9
10
Chromosome

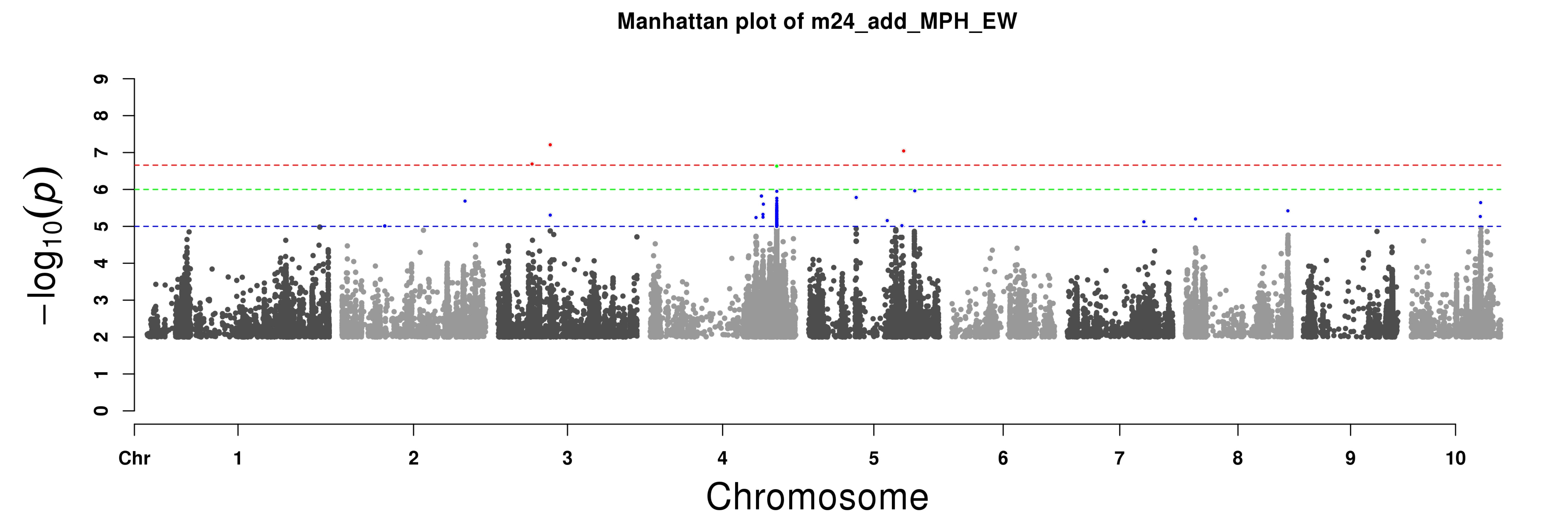

Manhattan plot of m24_add_MPH_EW
$-\log_{10}(p)$
0
1
2
3
4
5
6
7
8
9
Chr
1
2
3
4
5
6
7
8
9
10
Chromosome

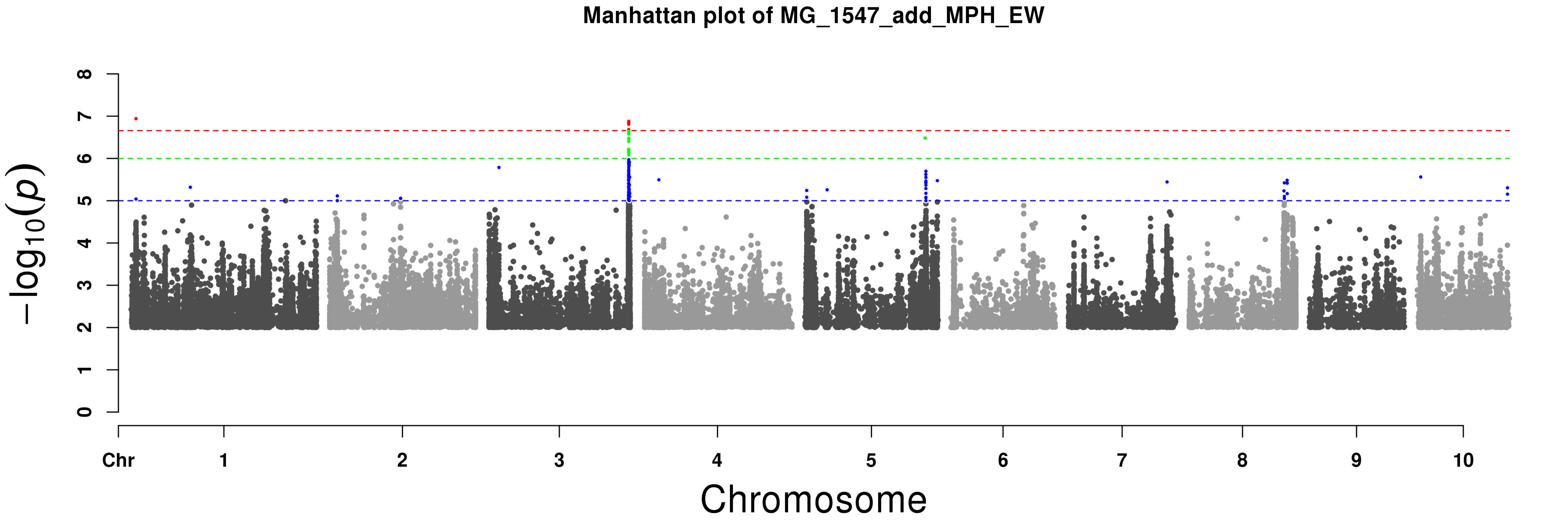

Manhattan plot of MG_1547_add_MPH_EW
$-\log_{10}(p)$
0
1
2
3
4
5
6
7
8
Chr
1
2
3
4
5
6
7
8
9
10
Chromosome

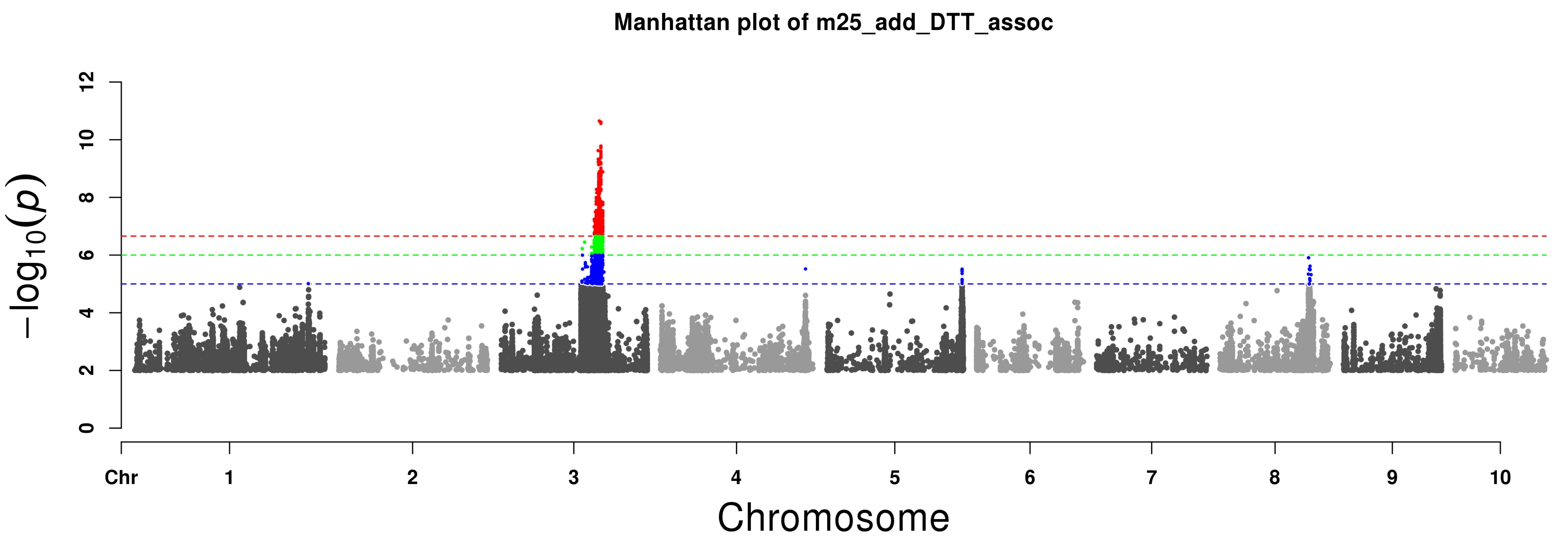

Manhattan plot of m25_add_DTT_assoc
$-\log_{10}(p)$
0
2
4
6
8
10
12
Chr
1
2
3
4
5
6
7
8
9
10
Chromosome

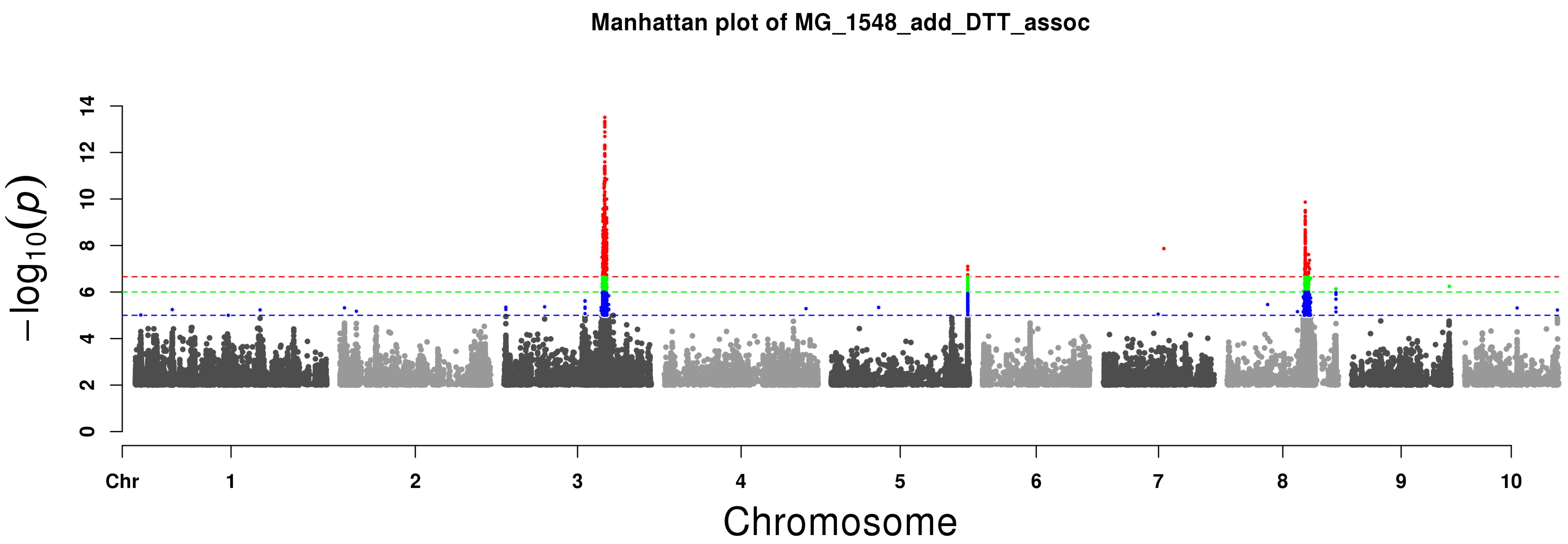

Manhattan plot of MG_1548_add_DTT_assoc
$-\log_{10}(p)$
0
2
4
6
8
10
12
14
Chr
1
2
3
4
5
6
7
8
9
10
Chromosome

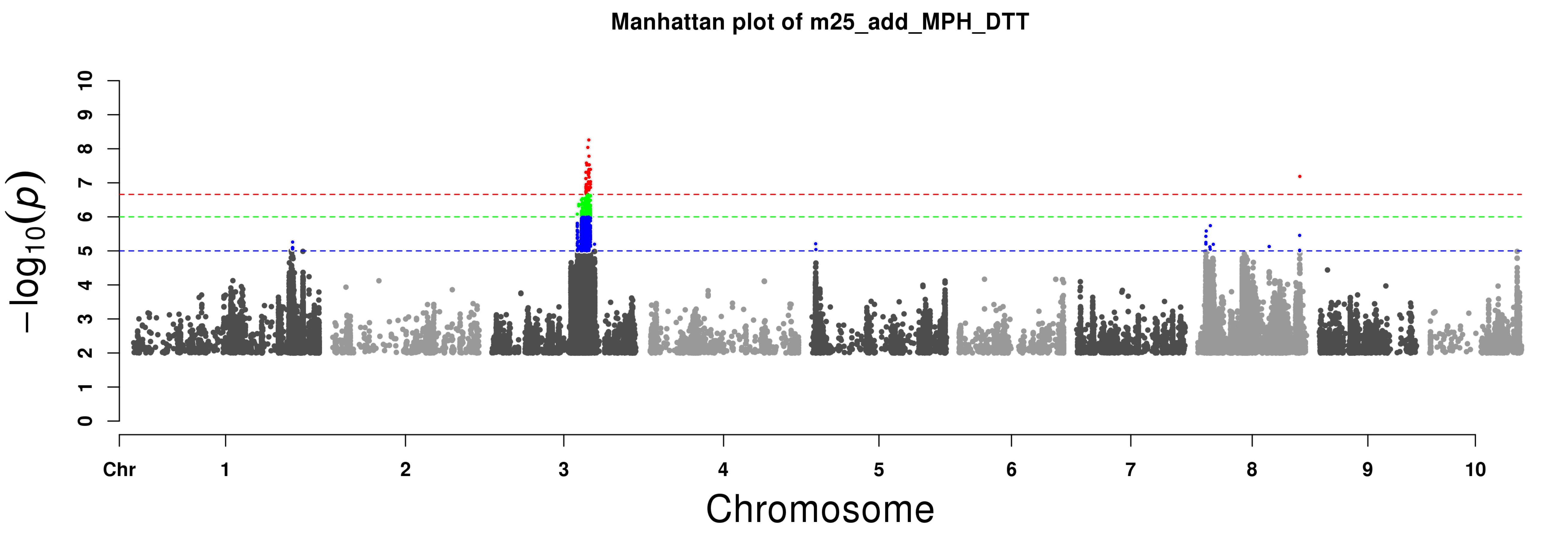
Manhattan plot of m25_add_MPH_DTT
0
1
2
3
4
5
6
7
8
9
10
$-\log_{10}(p)$
Chr
1
2
3
4
5
6
7
8
9
10
Chromosome

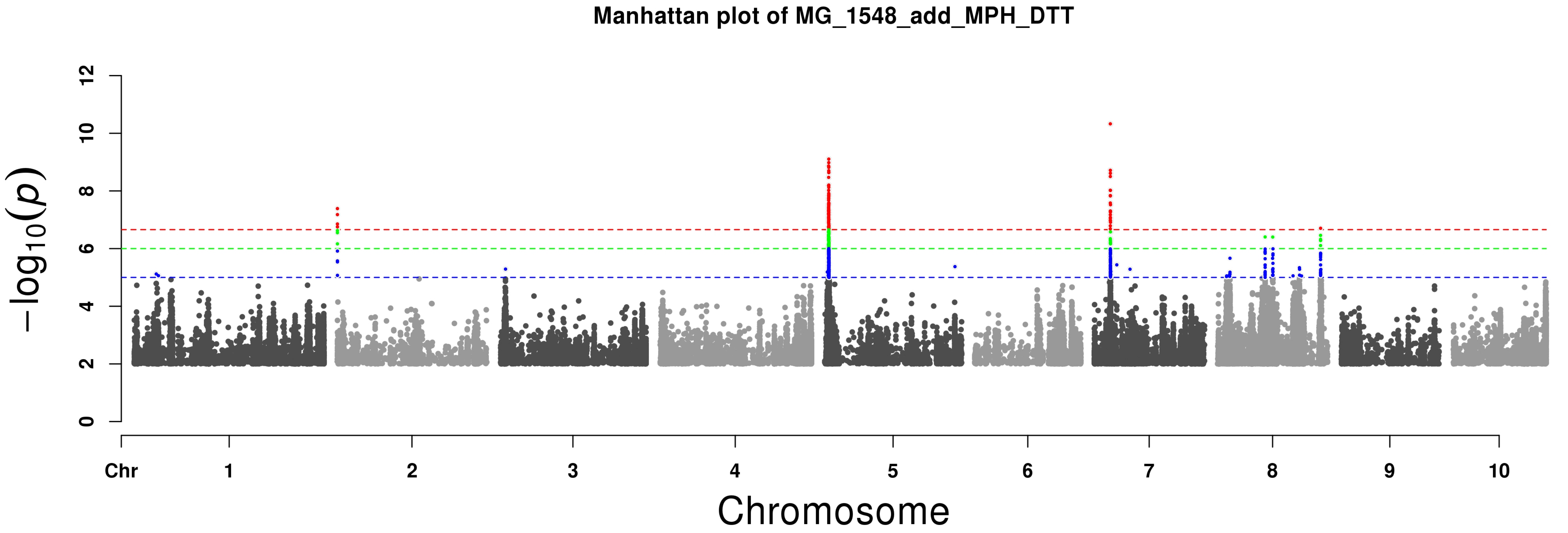
Manhattan plot of MG_1548_add_MPH_DTT
$-\log_{10}(p)$
0
2
4
6
8
10
12
Chr
1
2
3
4
5
6
7
8
9
10
Chromosome

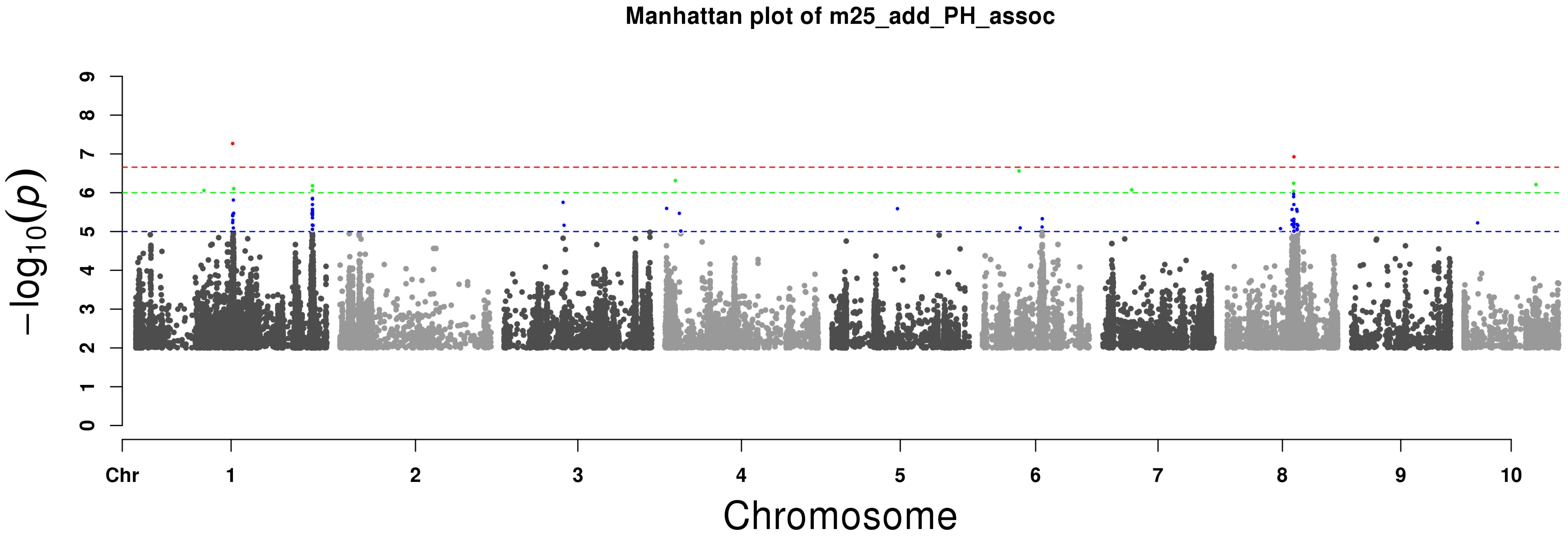
Manhattan plot of m25_add_PH_assoc
$-\log_{10}(p)$
0
1
2
3
4
5
6
7
8
9
Chr
1
2
3
4
5
6
7
8
9
10
Chromosome

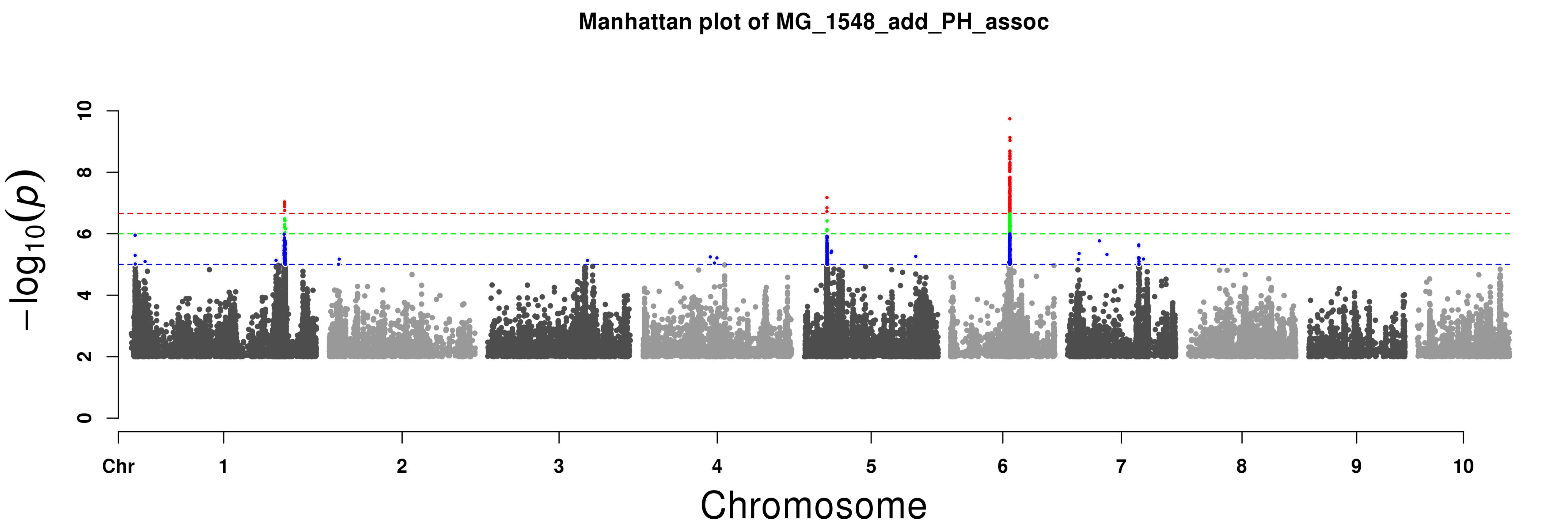
Manhattan plot of MG_1548_add_PH_assoc
$-\log_{10}(p)$
0
2
4
6
8
10
Chr
1
2
3
4
5
6
7
8
9
10
Chromosome

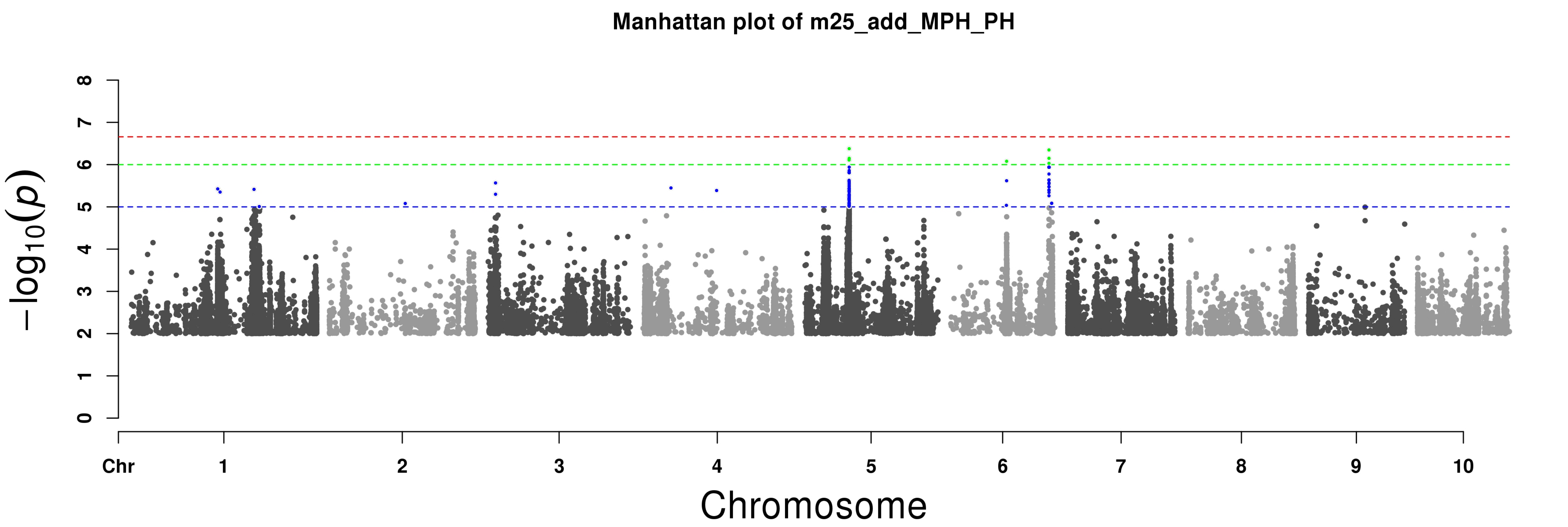

Manhattan plot of m25_add_MPH_PH
$-\log_{10}(p)$
0
1
2
3
4
5
6
7
8
Chr
1
2
3
4
5
6
7
8
9
10
Chromosome

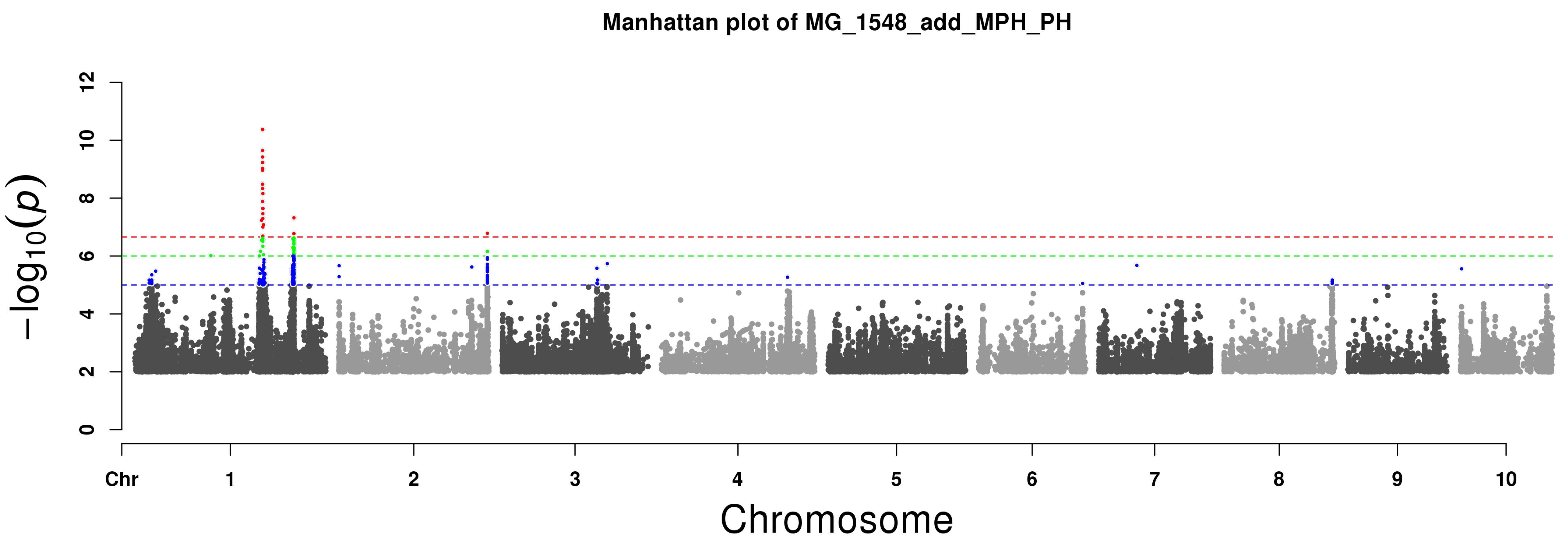
Manhattan plot of MG_1548_add_MPH_PH
−log10(p)
0
2
4
6
8
10
12
Chr
1
2
3
4
5
6
7
8
9
10
Chromosome

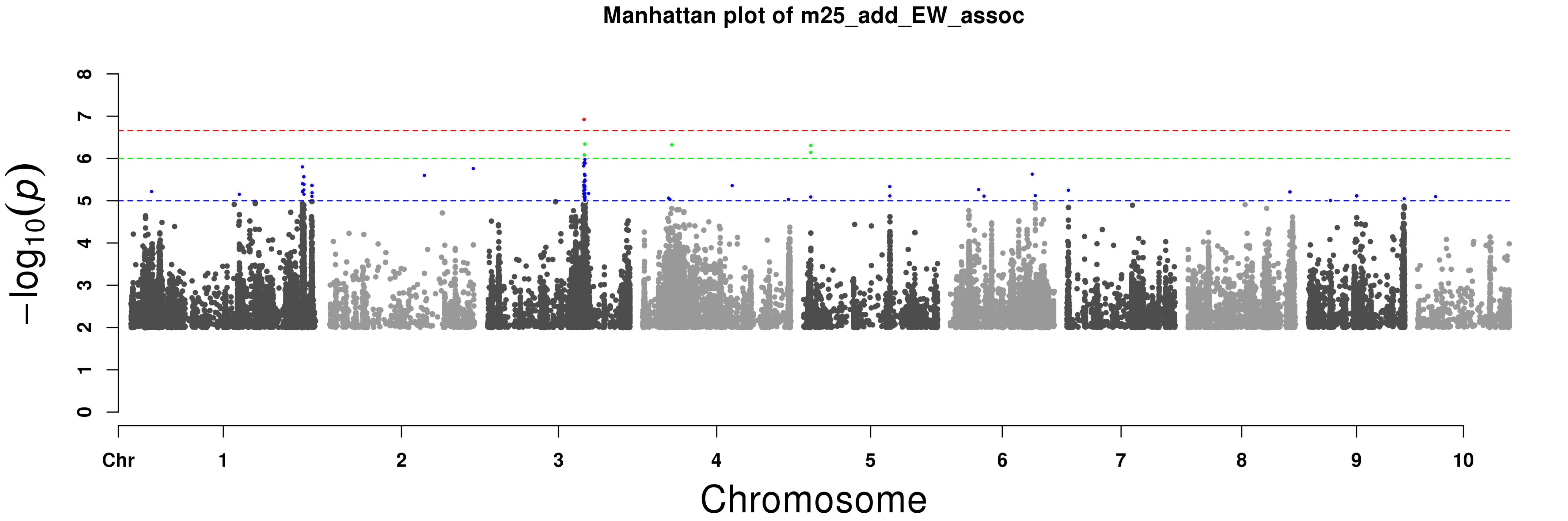
Manhattan plot of m25_add_EW_assoc
$-\log_{10}(p)$
0
1
2
3
4
5
6
7
8
Chr
1
2
3
4
5
6
7
8
9
10
Chromosome

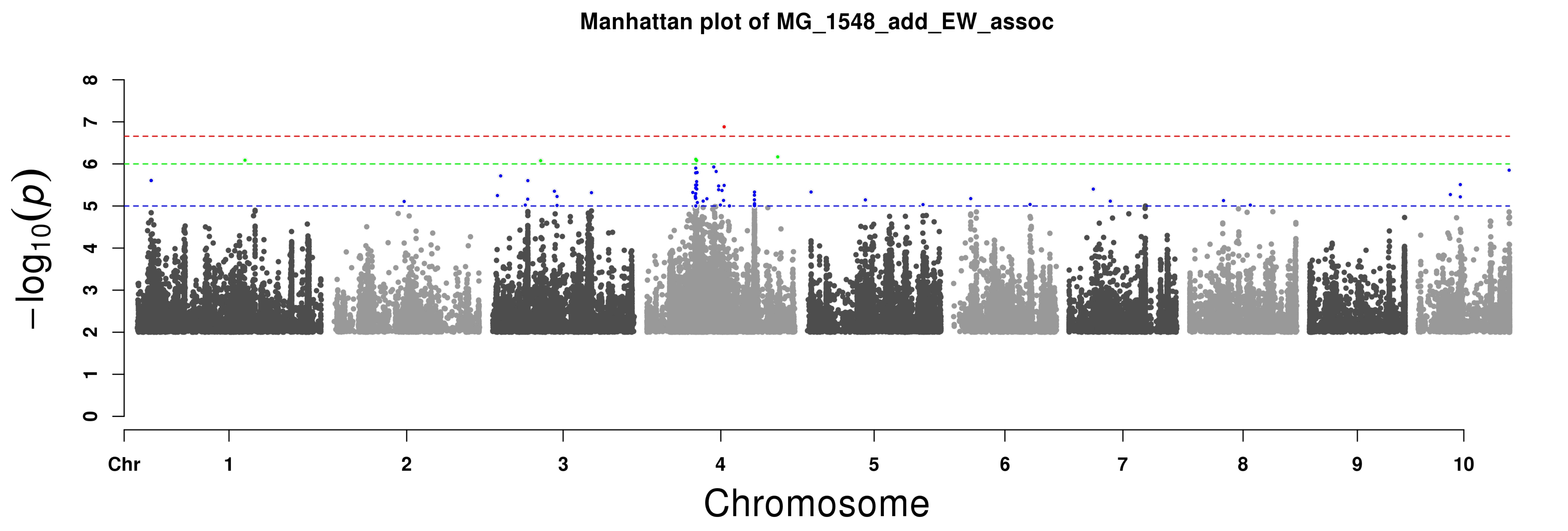
Manhattan plot of MG_1548_add_EW_assoc
$-\log_{10}(p)$
0
1
2
3
4
5
6
7
8
Chr
1
2
3
4
5
6
7
8
9
10
Chromosome

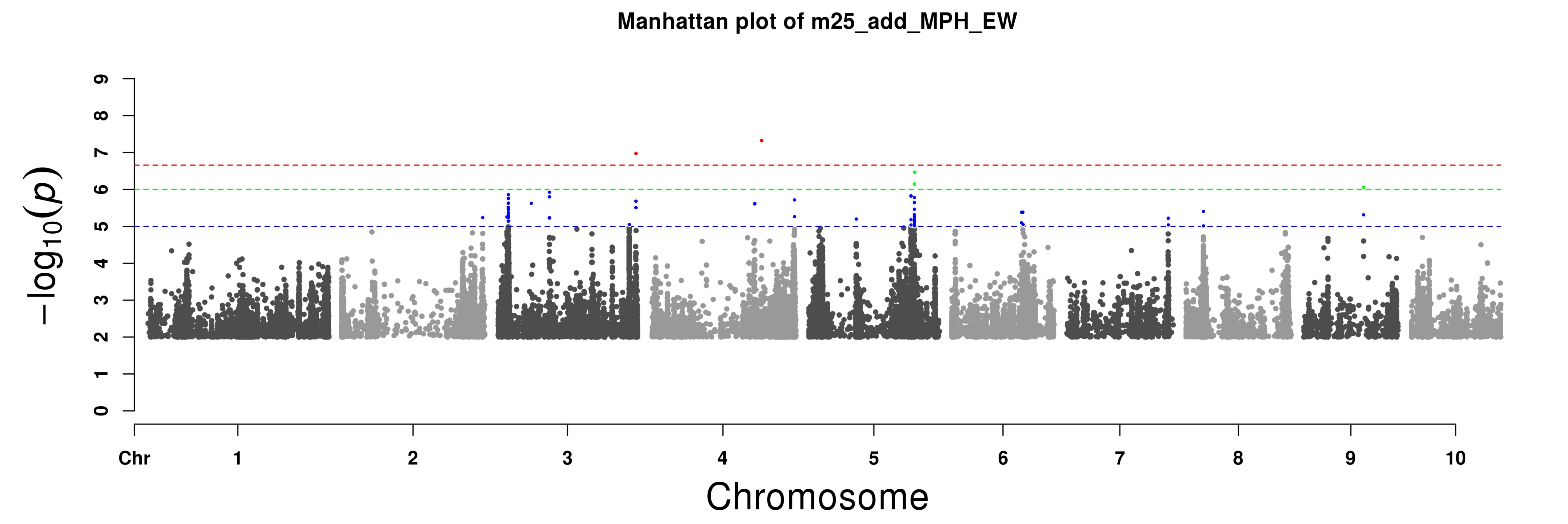

Manhattan plot of m25_add_MPH_EW
$-\log_{10}(p)$
0
1
2
3
4
5
6
7
8
9
Chr
1
2
3
4
5
6
7
8
9
10
Chromosome

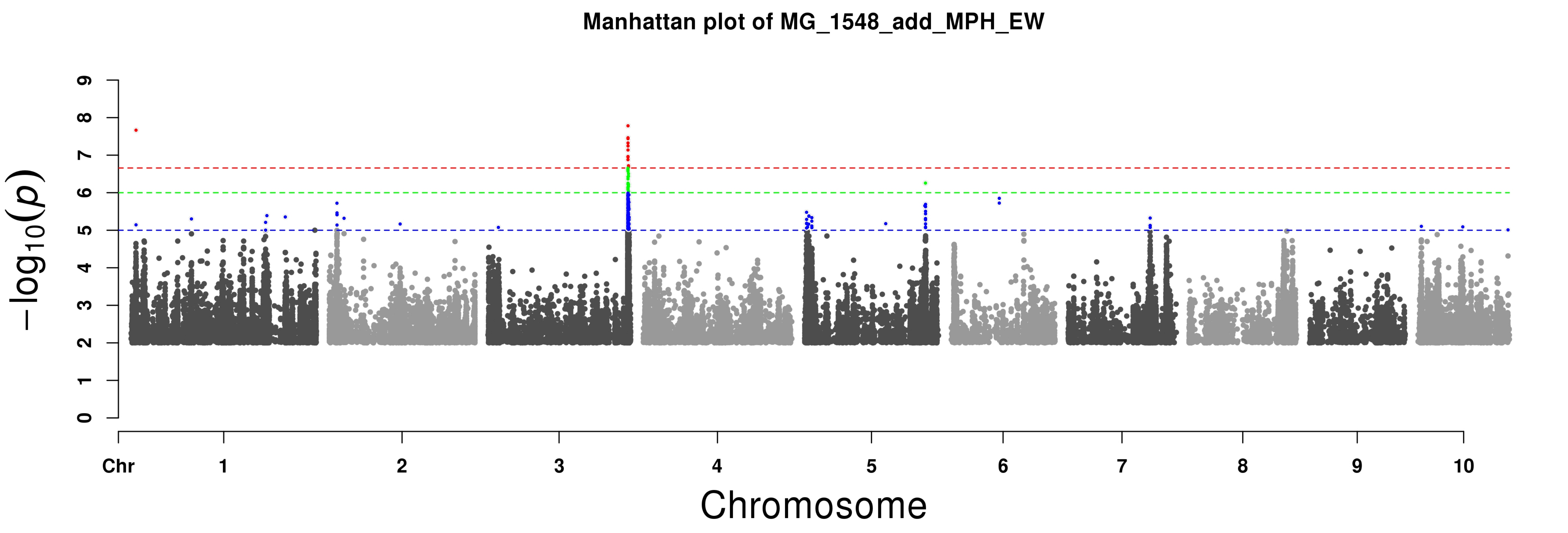

Manhattan plot of MG_1548_add_MPH_EW
$-\log_{10}(p)$
0
1
2
3
4
5
6
7
8
9
Chr
1
2
3
4
5
6
7
8
9
10
Chromosome

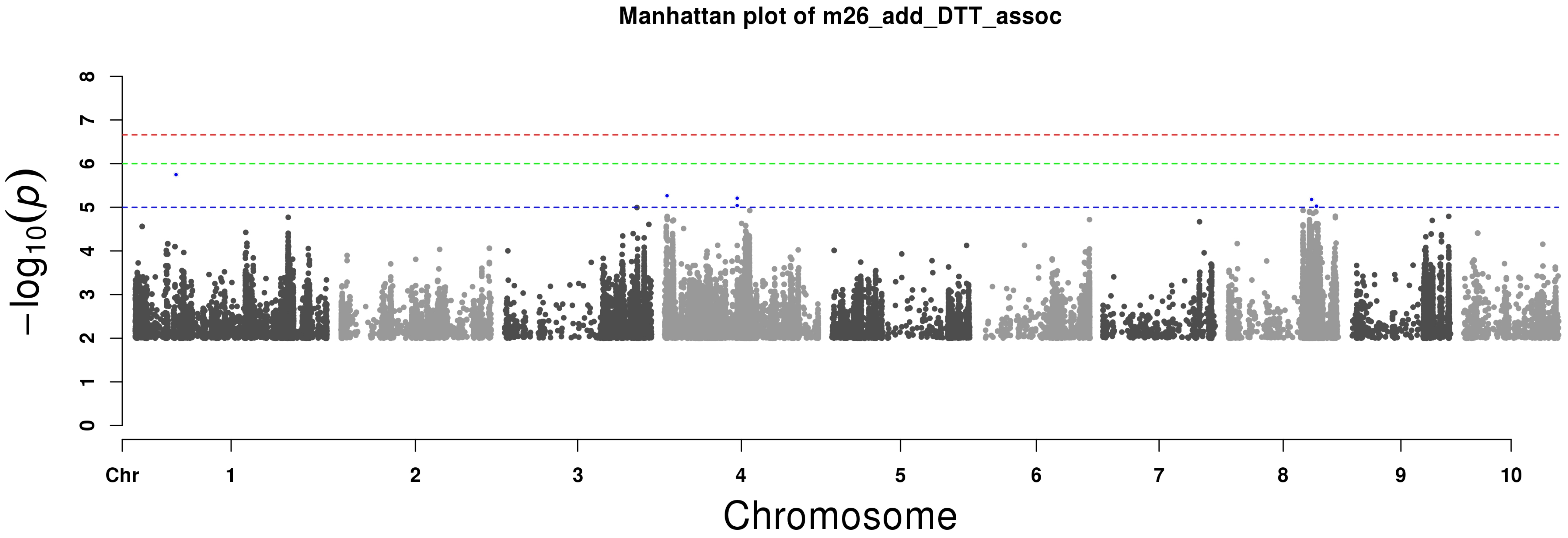

Manhattan plot of m26_add_DTT_assoc
$-\log_{10}(p)$
0
1
2
3
4
5
6
7
8
Chr
1
2
3
4
5
6
7
8
9
10
Chromosome

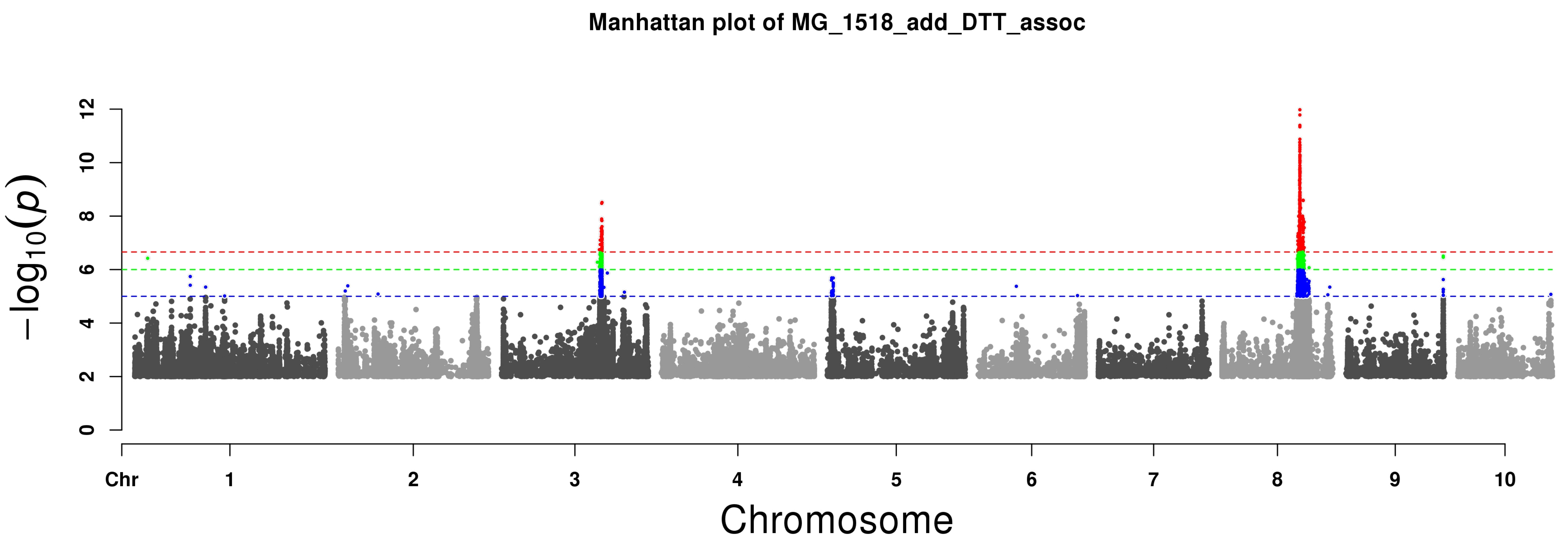
Manhattan plot of MG_1518_add_DTT_assoc
$-\log_{10}(p)$
0
2
4
6
8
10
12
Chr
1
2
3
4
5
6
7
8
9
10
Chromosome

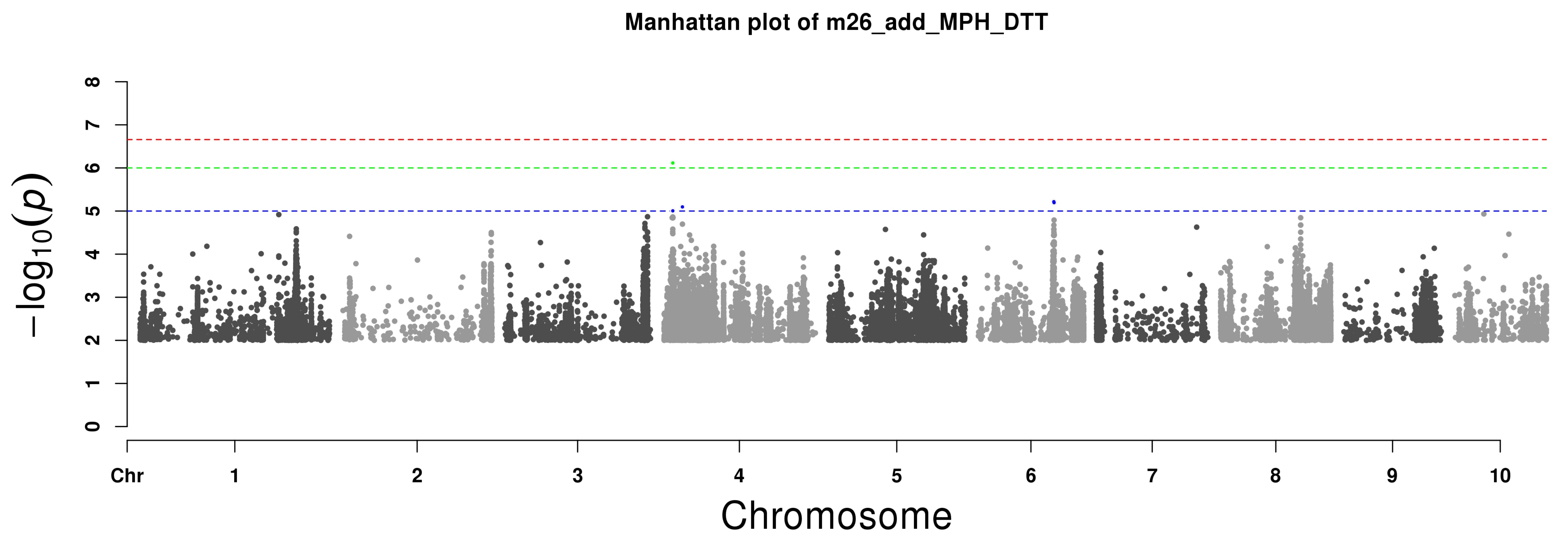

Manhattan plot of m26_add_MPH_DTT
$-\log_{10}(p)$
0
1
2
3
4
5
6
7
8
Chr
1
2
3
4
5
6
7
8
9
10
Chromosome

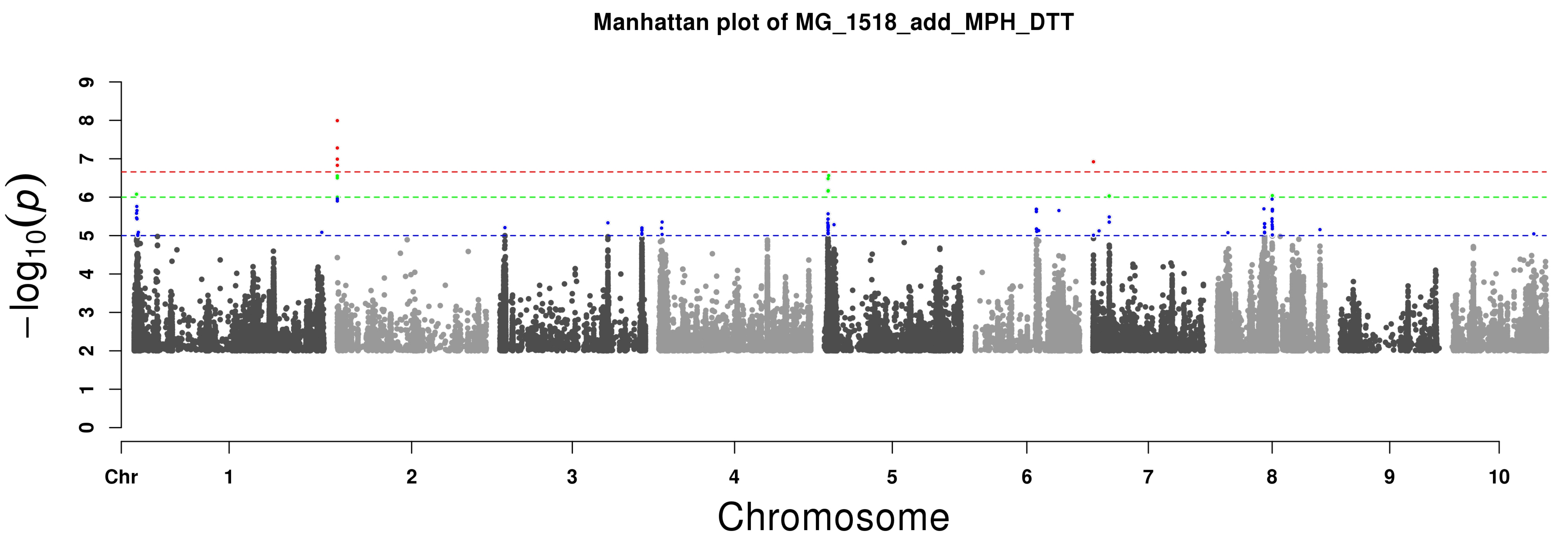
Manhattan plot of MG_1518_add_MPH_DTT
$-\log_{10}(p)$
0
1
2
3
4
5
6
7
8
9
Chr
1
2
3
4
5
6
7
8
9
10
Chromosome

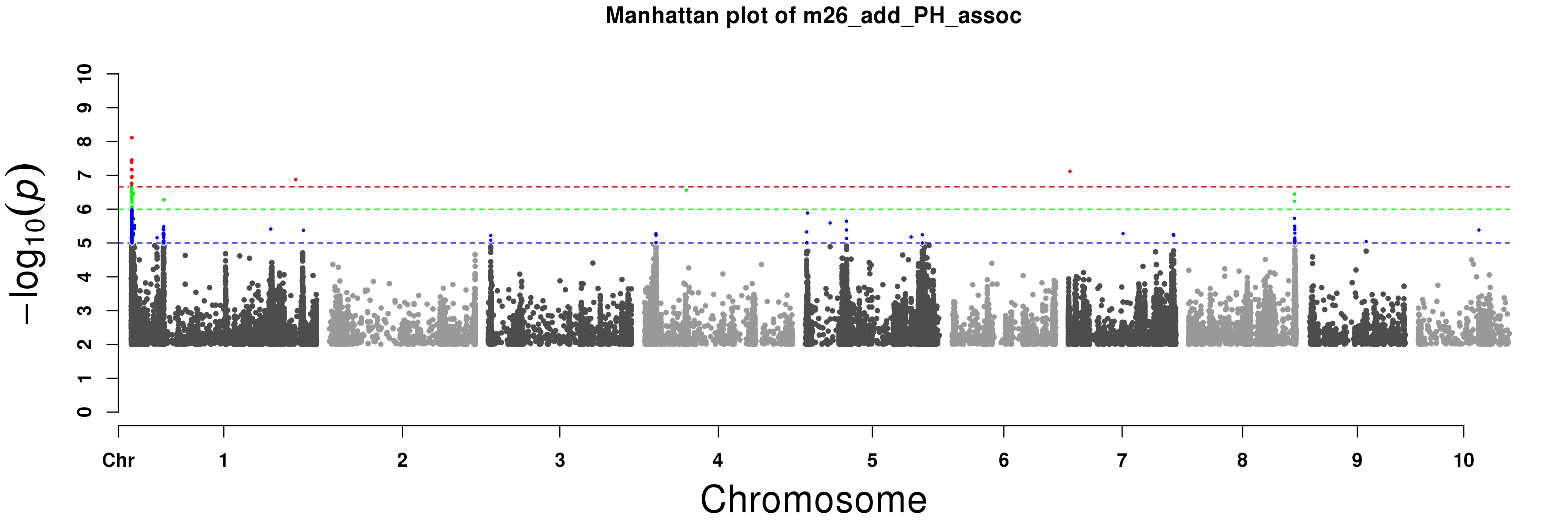

Manhattan plot of m26_add_PH_assoc
$-\log_{10}(p)$
0
1
2
3
4
5
6
7
8
9
10
Chr
1
2
3
4
5
6
7
8
9
10
Chromosome

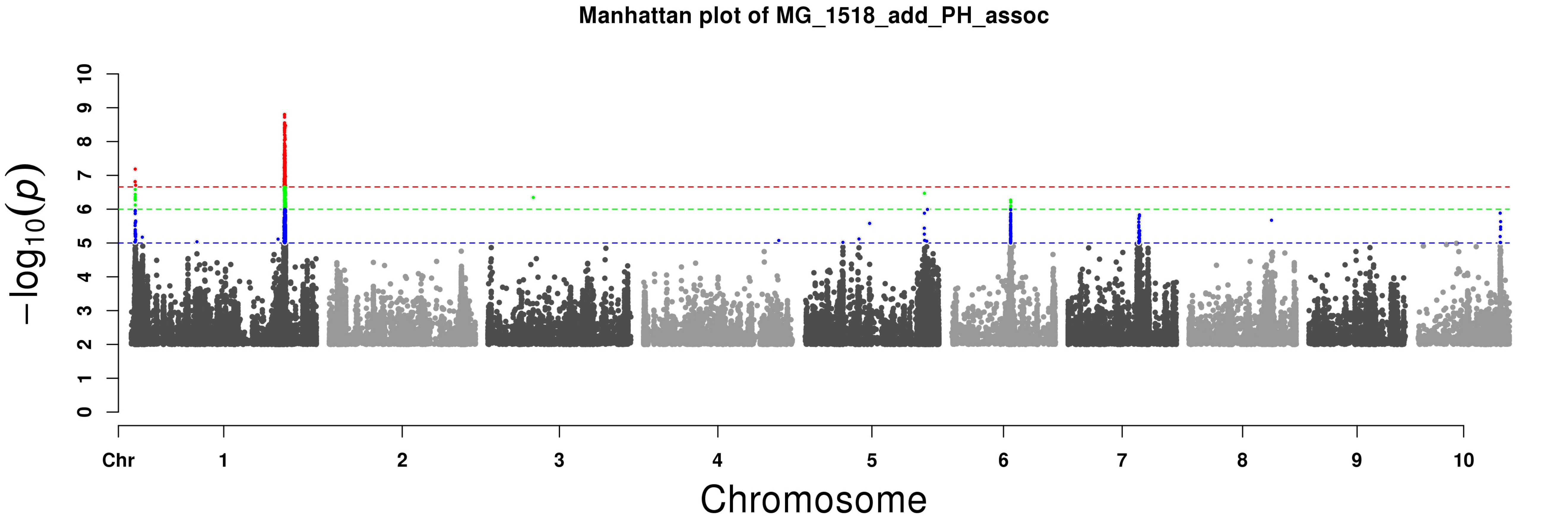

Manhattan plot of MG_1518_add_PH_assoc
$-\log_{10}(p)$
0
1
2
3
4
5
6
7
8
9
10
Chr
1
2
3
4
5
6
7
8
9
10
Chromosome

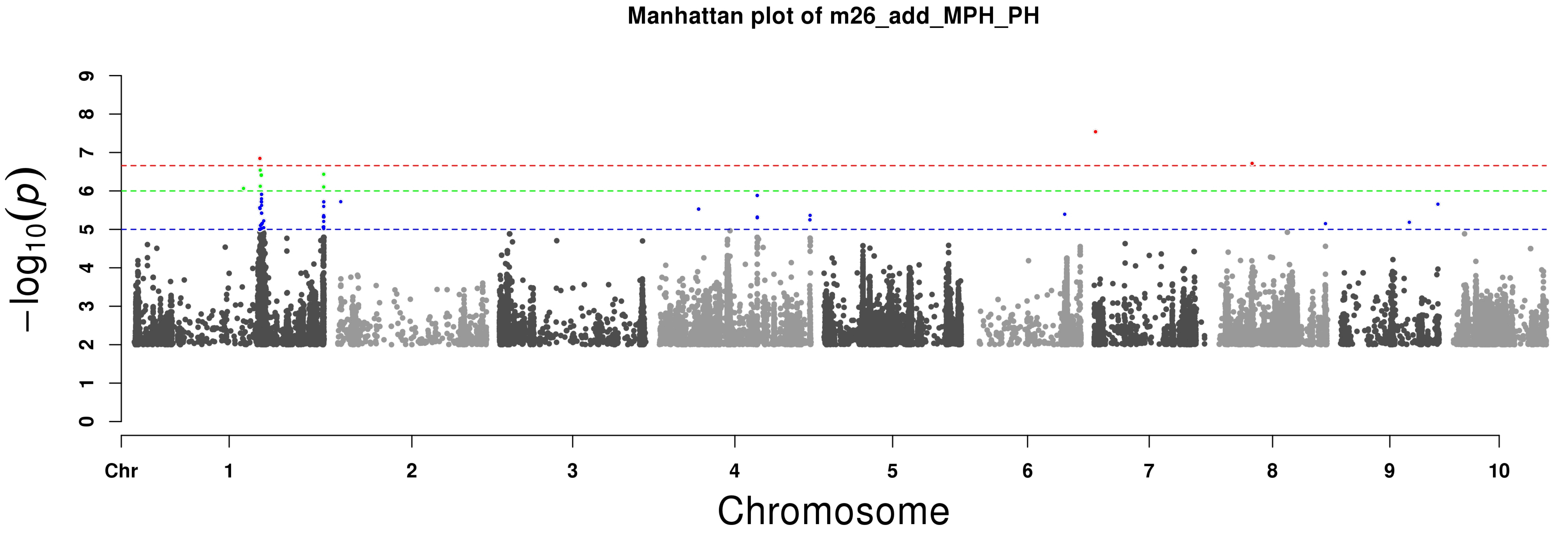
Manhattan plot of m26_add_MPH_PH
$-\log_{10}(p)$
0
1
2
3
4
5
6
7
8
9
Chr
1
2
3
4
5
6
7
8
9
10
Chromosome

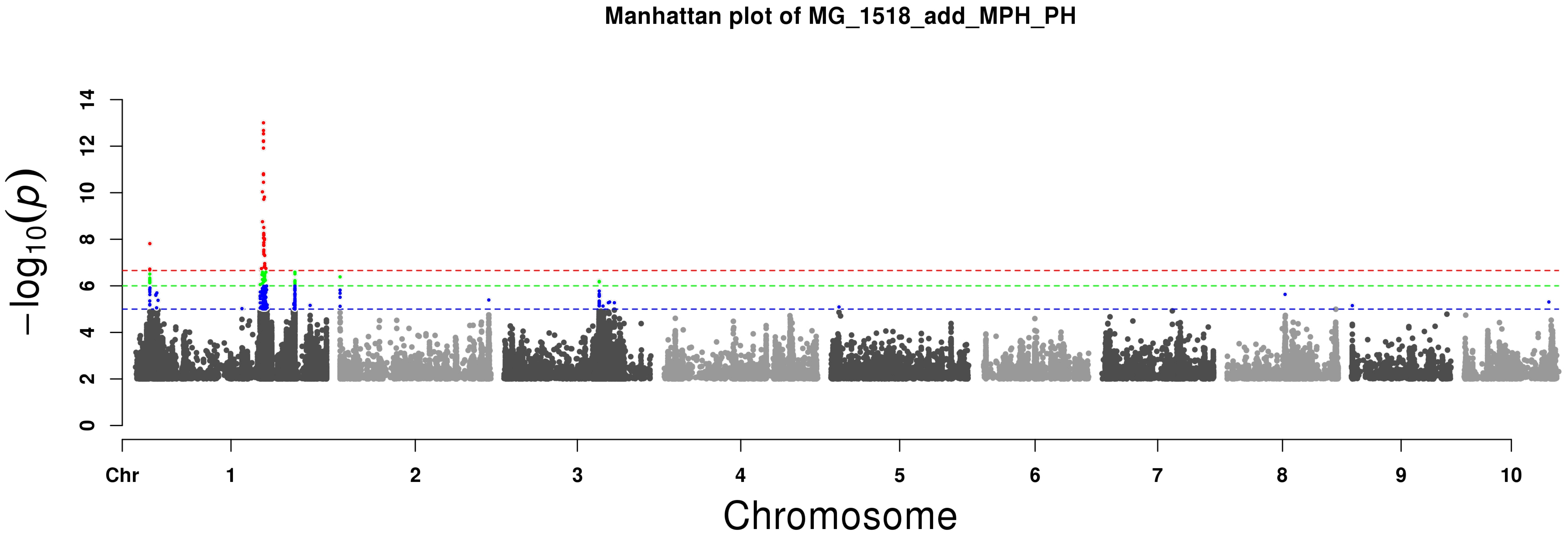

Manhattan plot of MG_1518_add_MPH_PH
$-\log_{10}(p)$
0
2
4
6
8
10
12
14
Chr
1
2
3
4
5
6
7
8
9
10
Chromosome

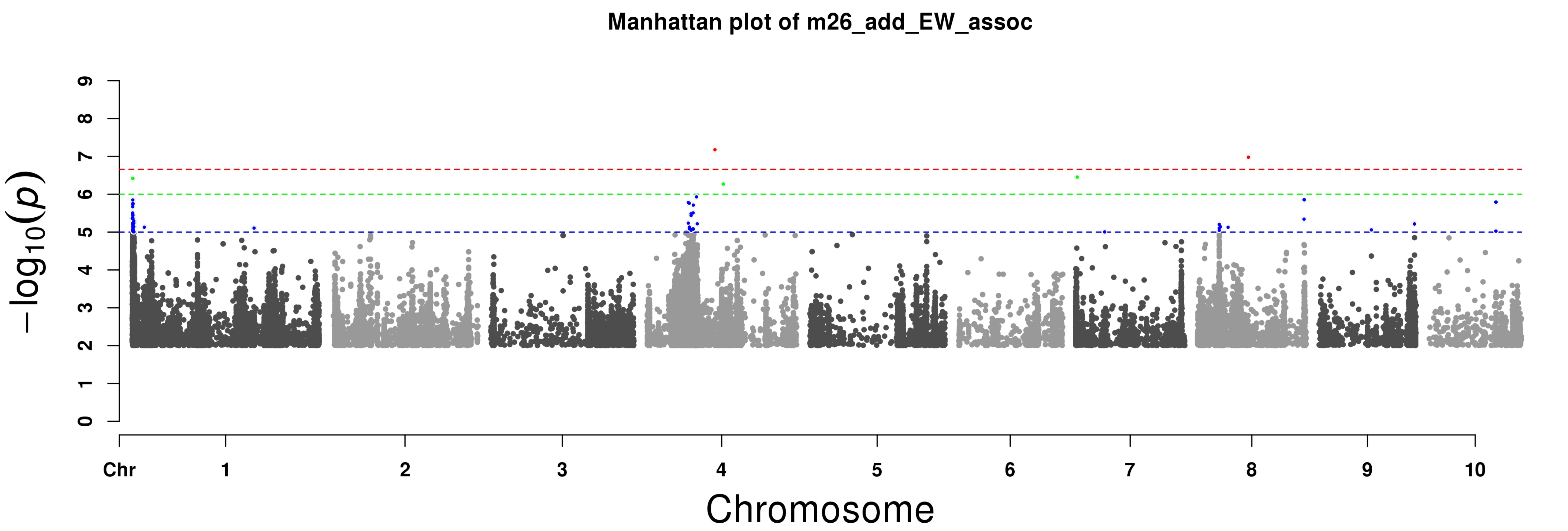
Manhattan plot of m26_add_EW_assoc
$-\log_{10}(p)$
0
1
2
3
4
5
6
7
8
9
Chr
1
2
3
4
5
6
7
8
9
10
Chromosome

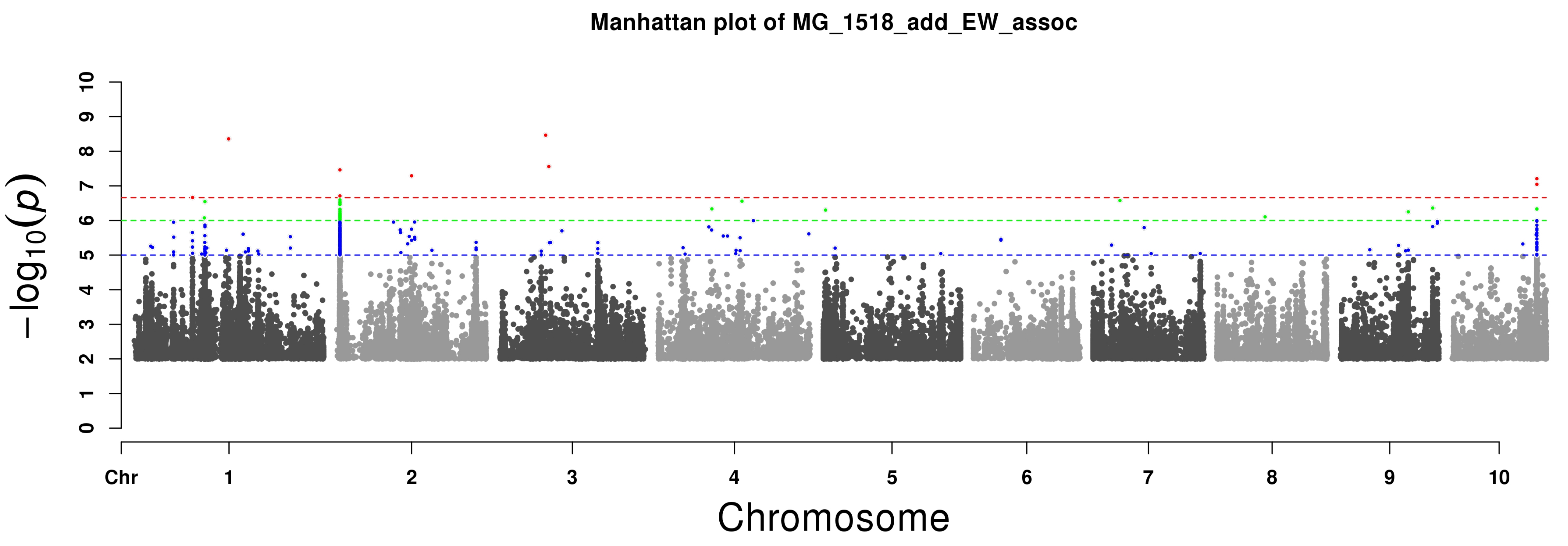

Manhattan plot of MG_1518_add_EW_assoc
$-\log_{10}(p)$
0
1
2
3
4
5
6
7
8
9
10
Chr
1
2
3
4
5
6
7
8
9
10
Chromosome

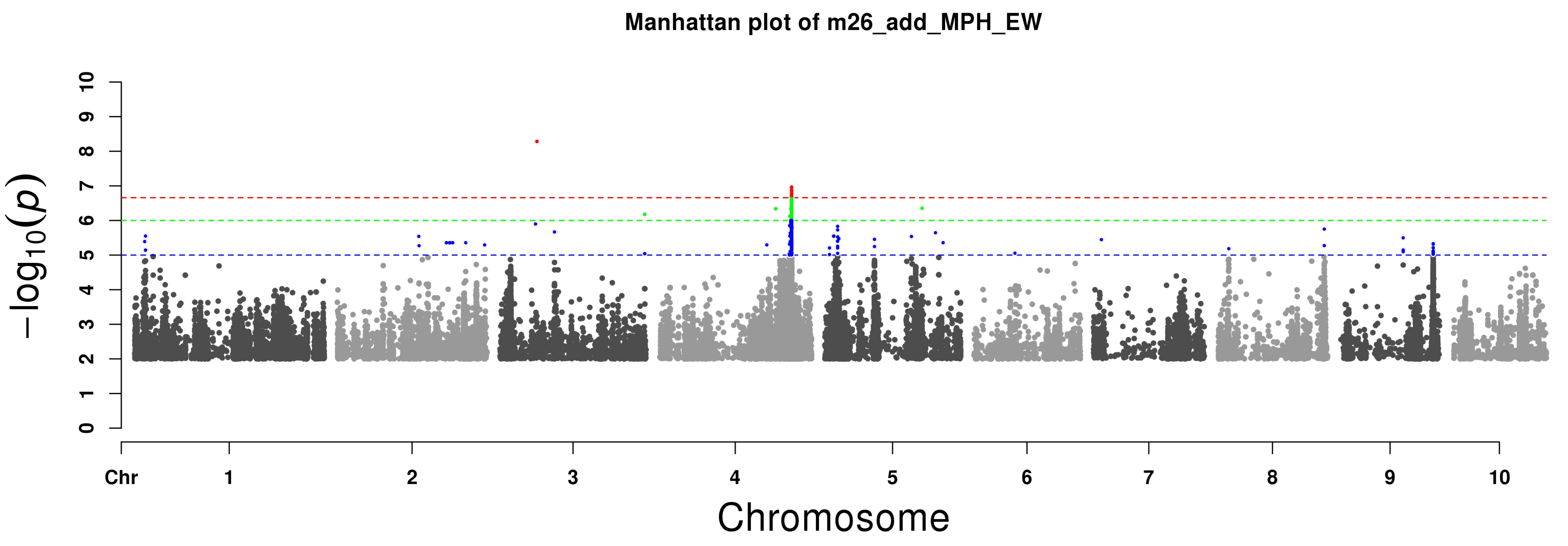
Manhattan plot of m26_add_MPH_EW
$-\log_{10}(p)$
0
1
2
3
4
5
6
7
8
9
10
Chr
1
2
3
4
5
6
7
8
9
10
Chromosome

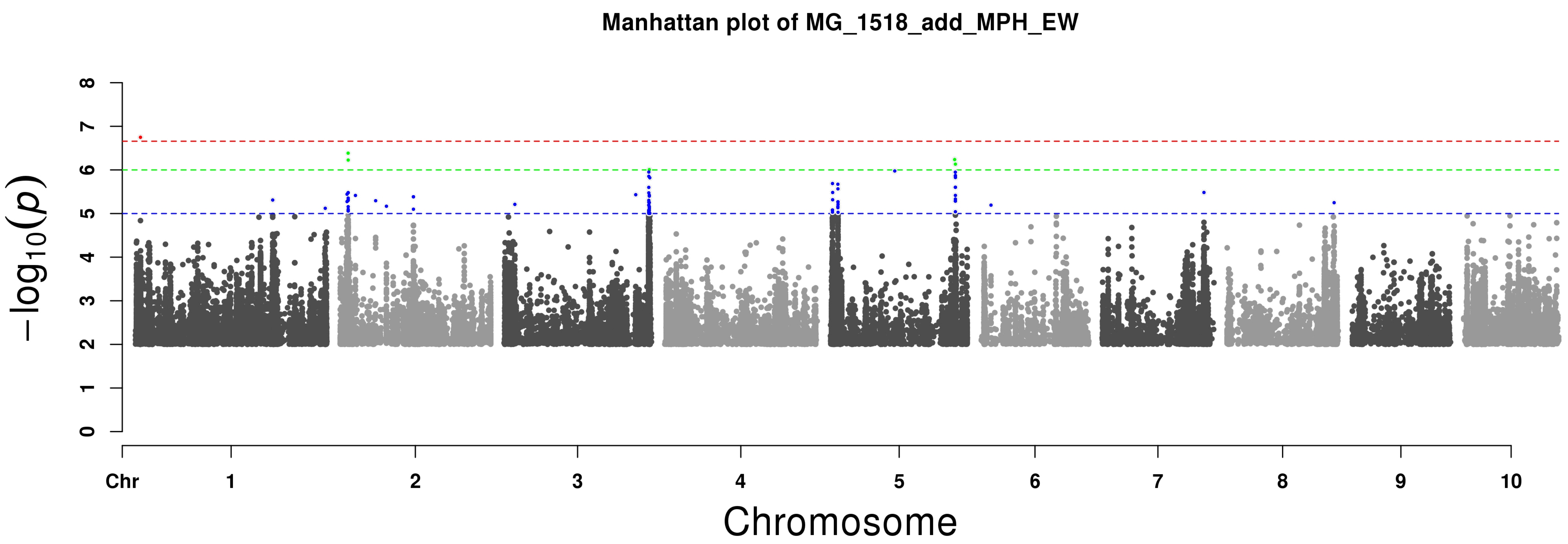

Manhattan plot of MG_1518_add_MPH_EW
$-\log_{10}(p)$
0
1
2
3
4
5
6
7
8
Chr
1
2
3
4
5
6
7
8
9
10
Chromosome

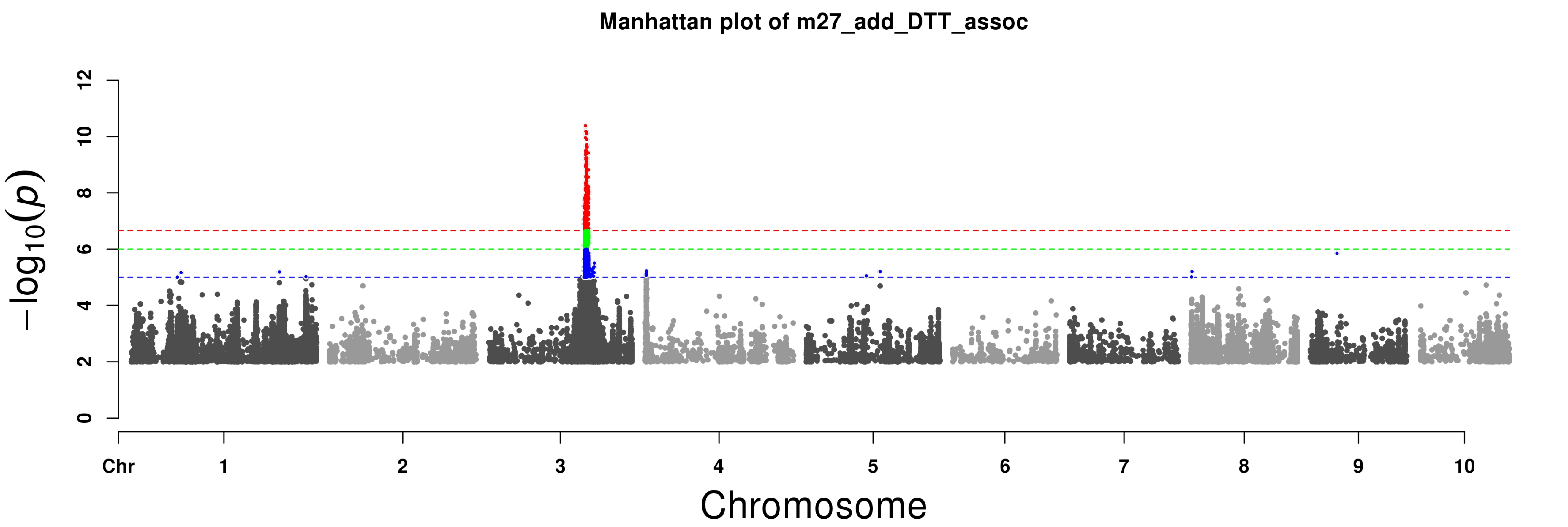

Manhattan plot of m27_add_DTT_assoc
$-\log_{10}(p)$
0
2
4
6
8
10
12
Chr
1
2
3
4
5
6
7
8
9
10
Chromosome

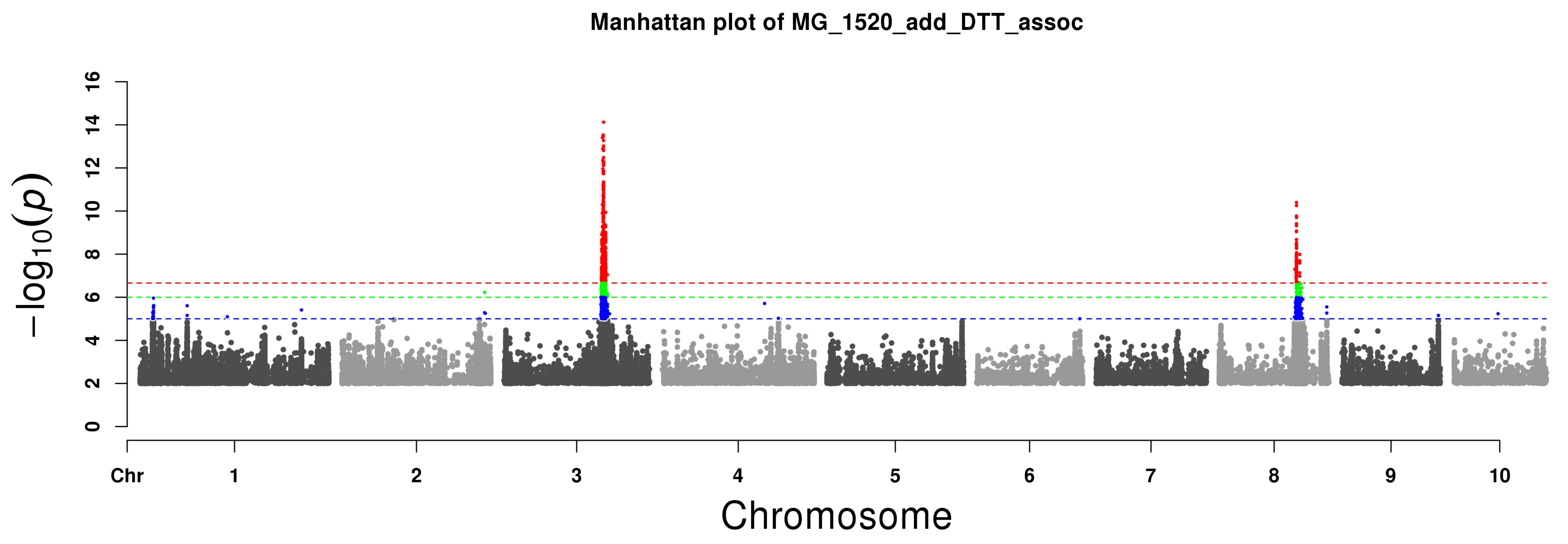

Manhattan plot of MG_1520_add_DTT_assoc
$-\log_{10}(p)$
0
2
4
6
8
10
12
14
16
Chr
1
2
3
4
5
6
7
8
9
10
Chromosome

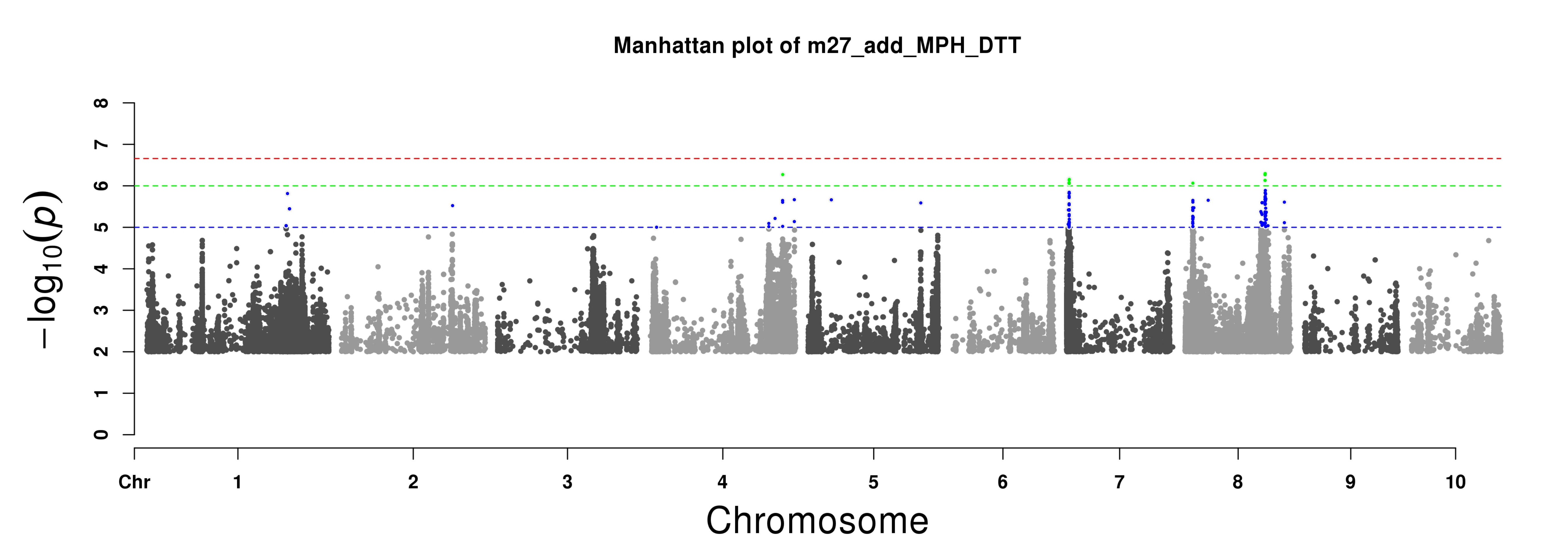
Manhattan plot of m27_add_MPH_DTT
$-\log_{10}(p)$
0
1
2
3
4
5
6
7
8
Chr
1
2
3
4
5
6
7
8
9
10
Chromosome

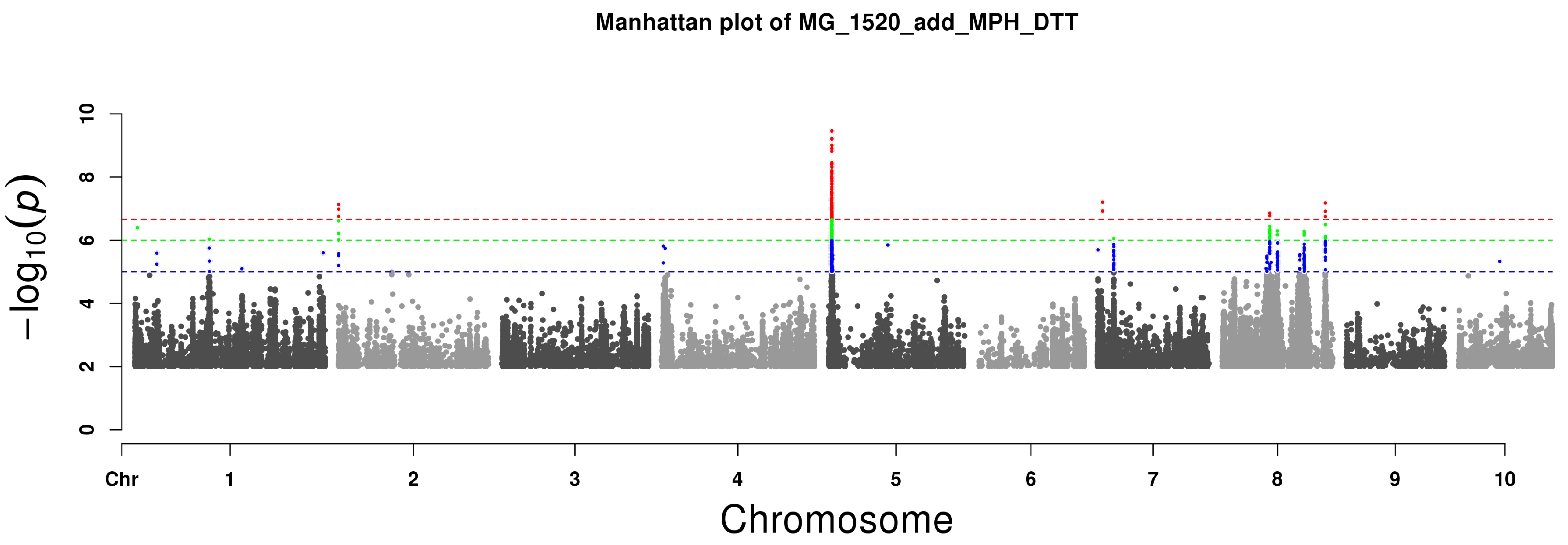

Manhattan plot of MG_1520_add_MPH_DTT
−log10(p)
0
2
4
6
8
10
Chr
1
2
3
4
5
6
7
8
9
10
Chromosome

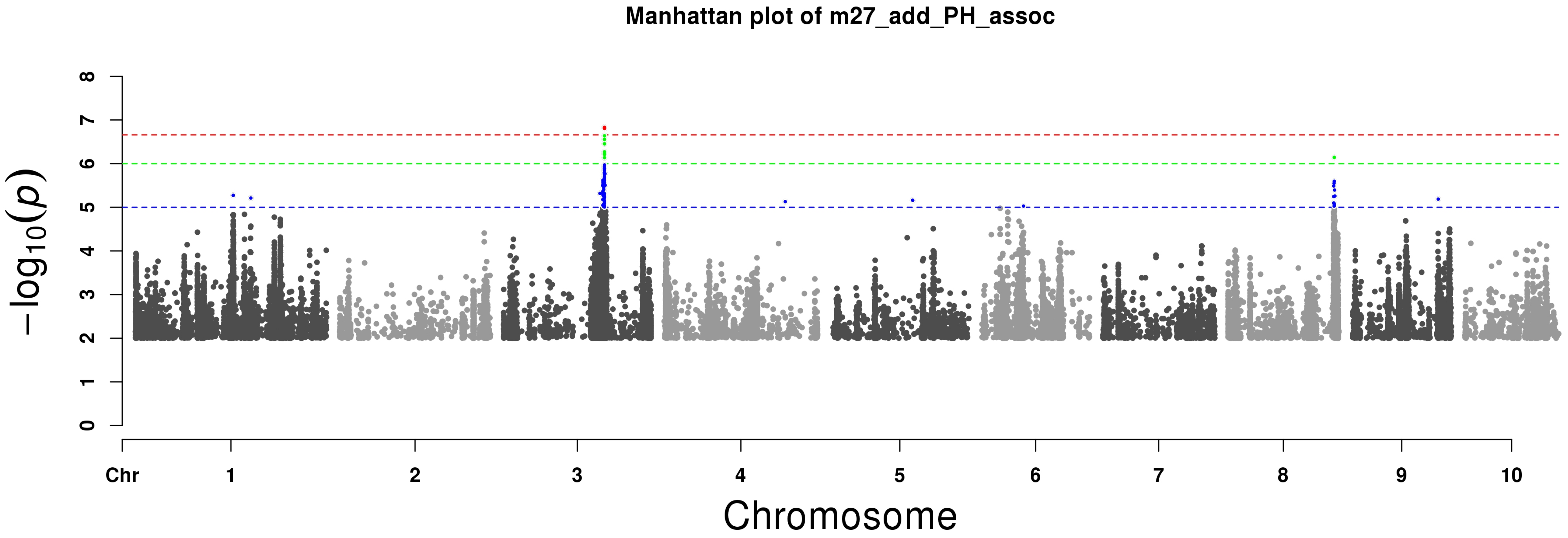

Manhattan plot of m27_add_PH_assoc
$-\log_{10}(p)$
0
1
2
3
4
5
6
7
8
Chr
1
2
3
4
5
6
7
8
9
10
Chromosome

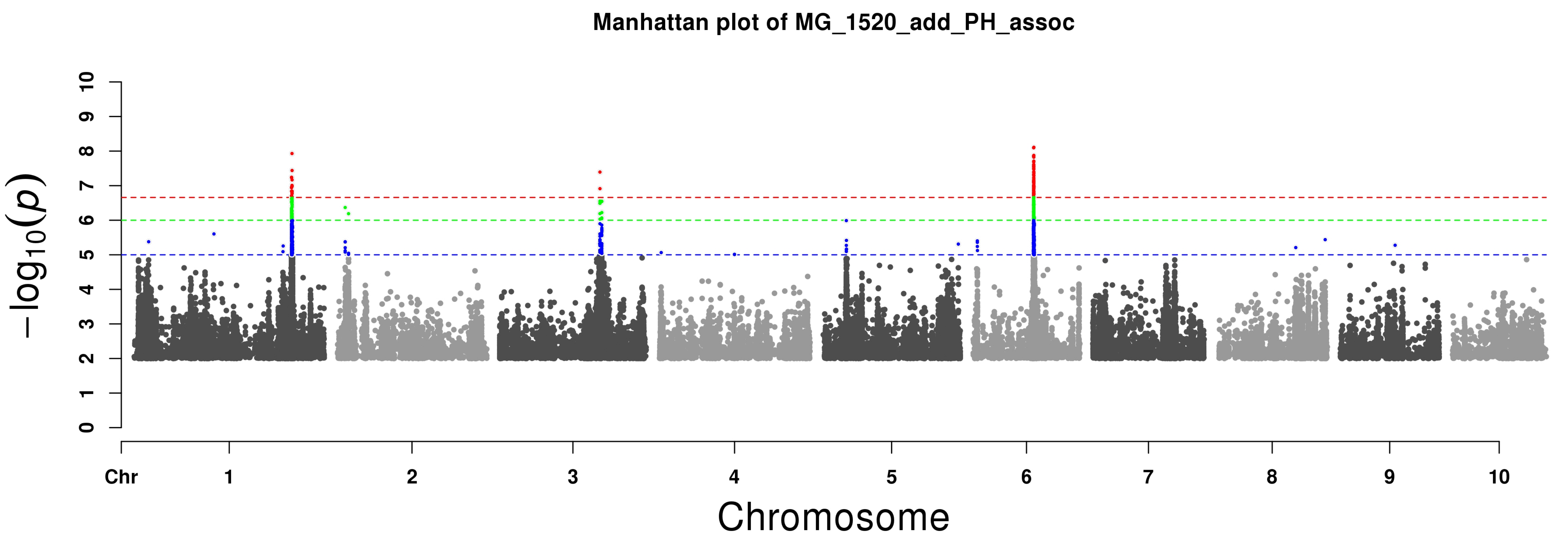
Manhattan plot of MG_1520_add_PH_assoc
$-\log_{10}(p)$
0
1
2
3
4
5
6
7
8
9
10
Chr
1
2
3
4
5
6
7
8
9
10
Chromosome

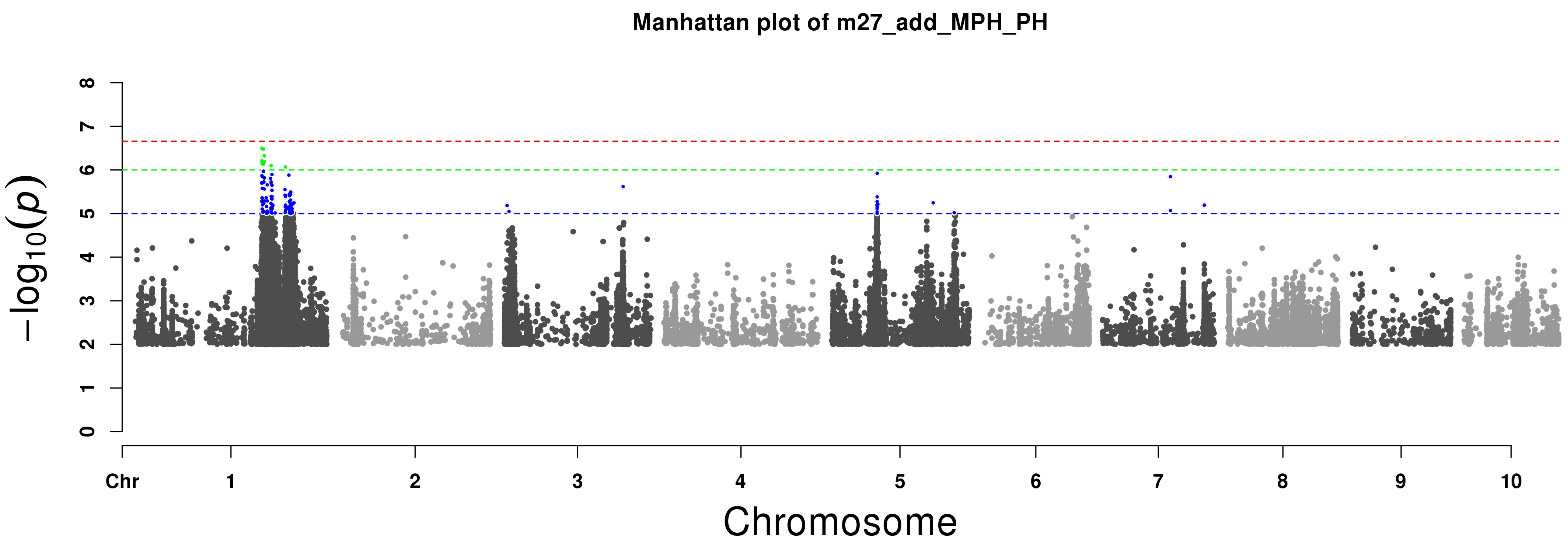

Manhattan plot of m27_add_MPH_PH
$-\log_{10}(p)$
0
1
2
3
4
5
6
7
8
Chr
1
2
3
4
5
6
7
8
9
10
Chromosome

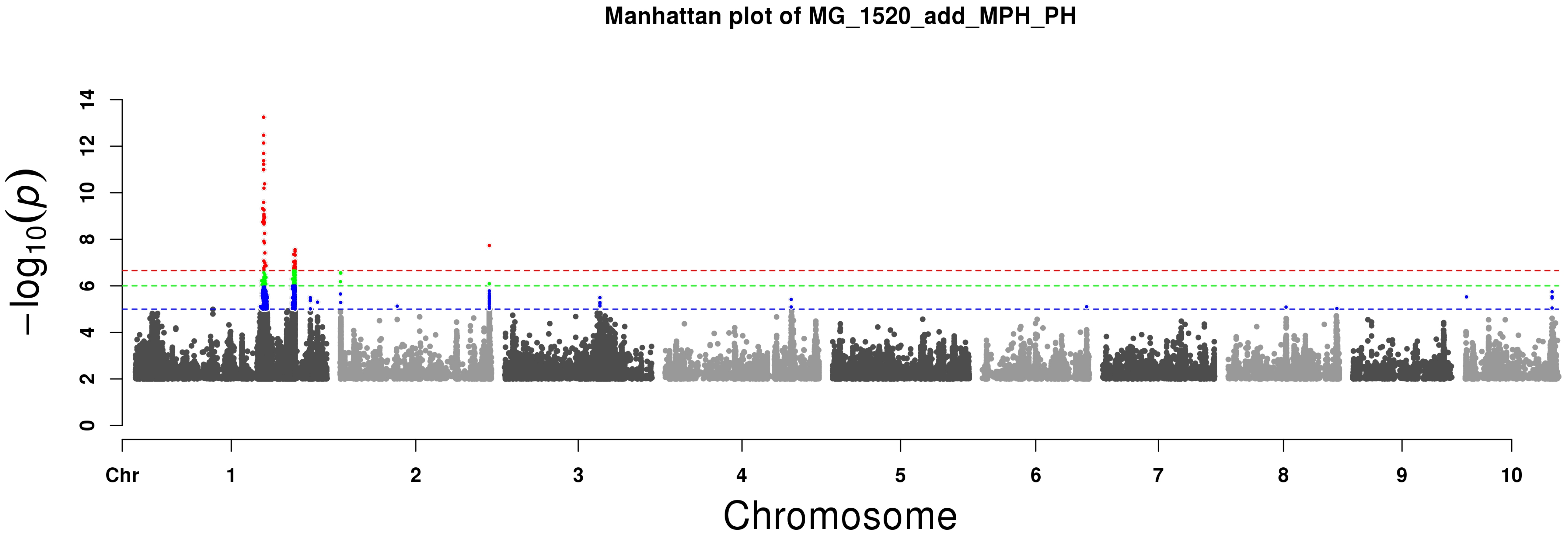

Manhattan plot of MG_1520_add_MPH_PH
$-\log_{10}(p)$
0
2
4
6
8
10
12
14
Chr
1
2
3
4
5
6
7
8
9
10
Chromosome

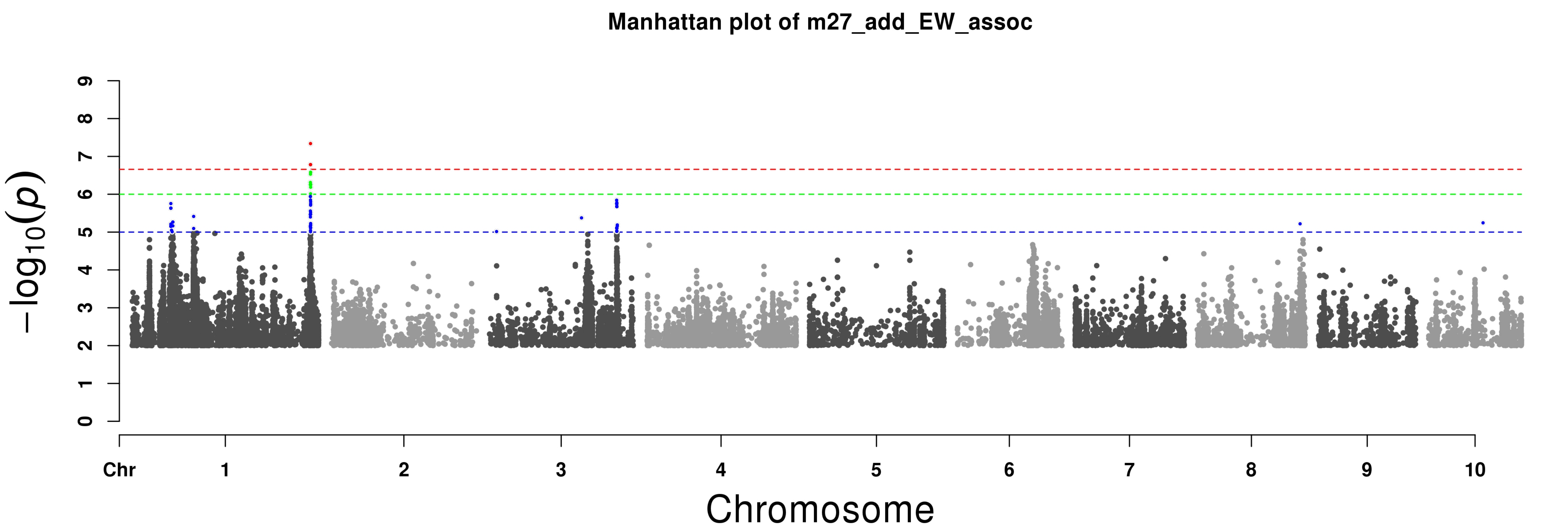

Manhattan plot of m27_add_EW_assoc
$-\log_{10}(p)$
0
1
2
3
4
5
6
7
8
9
Chr
1
2
3
4
5
6
7
8
9
10
Chromosome

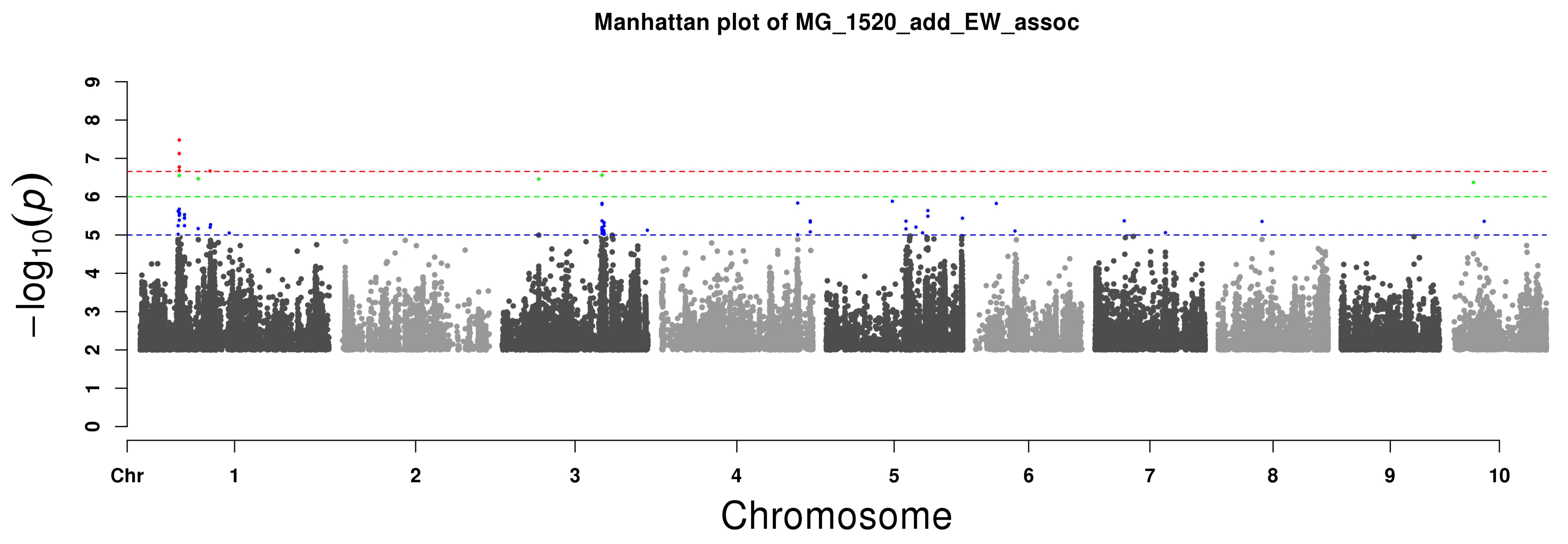
Manhattan plot of MG_1520_add_EW_assoc
$-\log_{10}(p)$
0
1
2
3
4
5
6
7
8
9
Chr
1
2
3
4
5
6
7
8
9
10
Chromosome

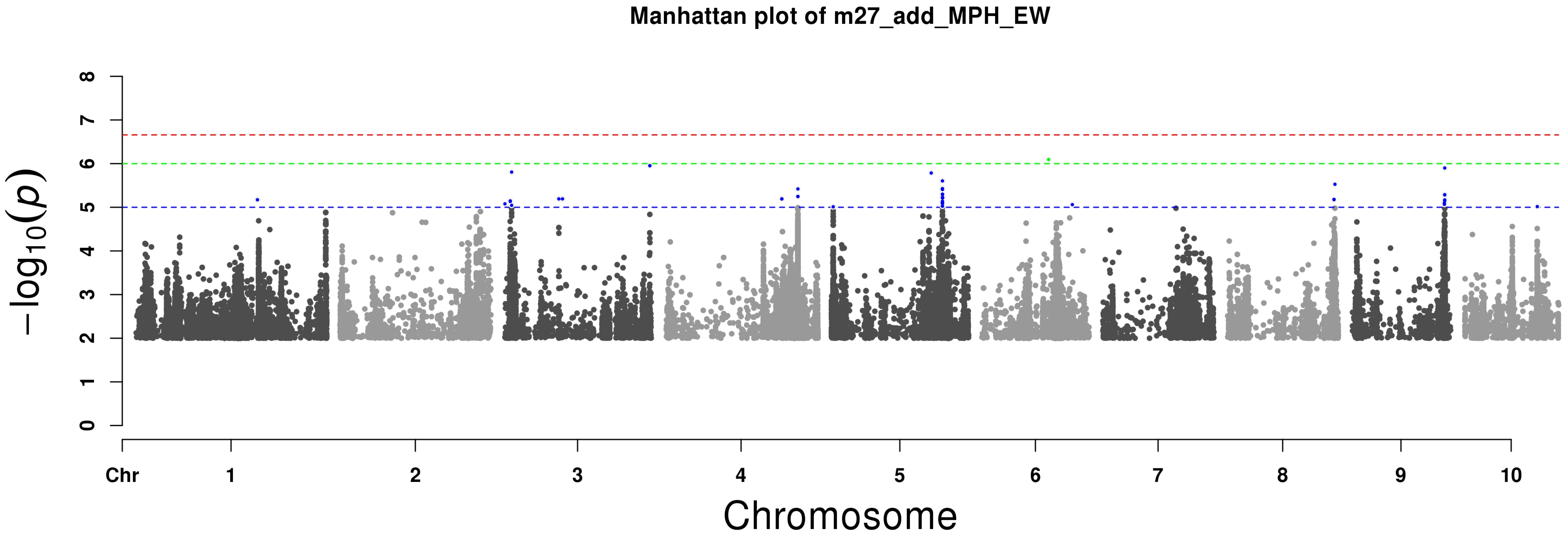

Manhattan plot of m27_add_MPH_EW
$-\log_{10}(p)$
0
1
2
3
4
5
6
7
8
Chr
1
2
3
4
5
6
7
8
9
10
Chromosome

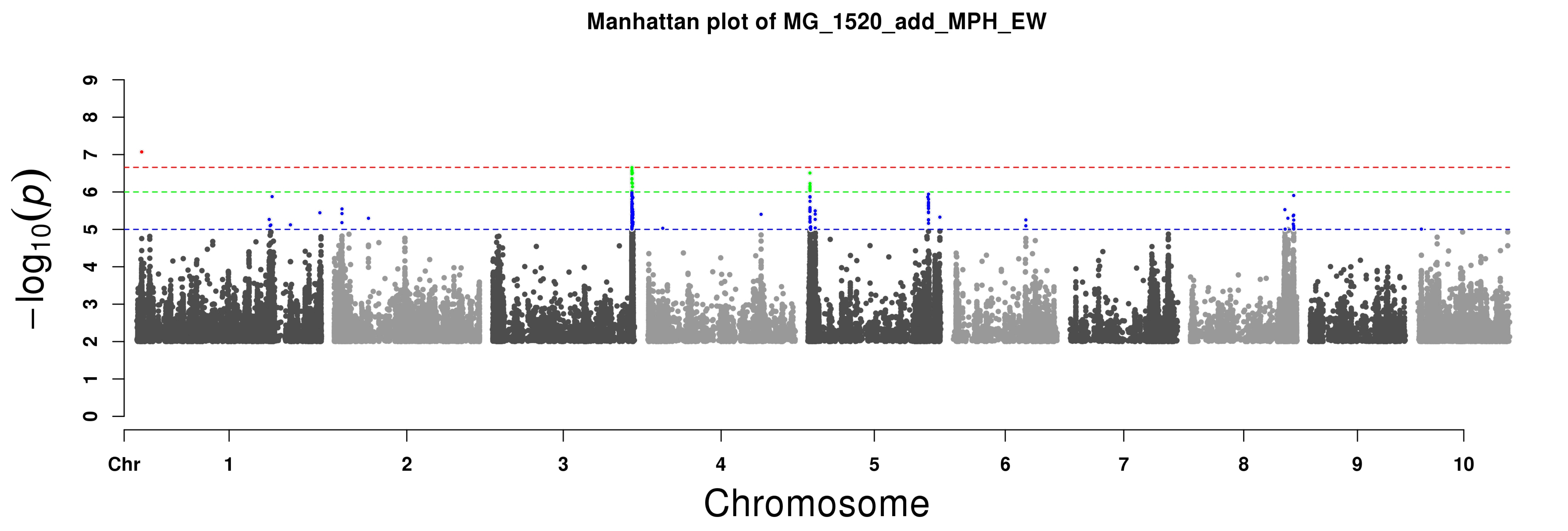

Manhattan plot of MG_1520_add_MPH_EW
$-\log_{10}(p)$
0
1
2
3
4
5
6
7
8
9
Chr
1
2
3
4
5
6
7
8
9
10
Chromosome

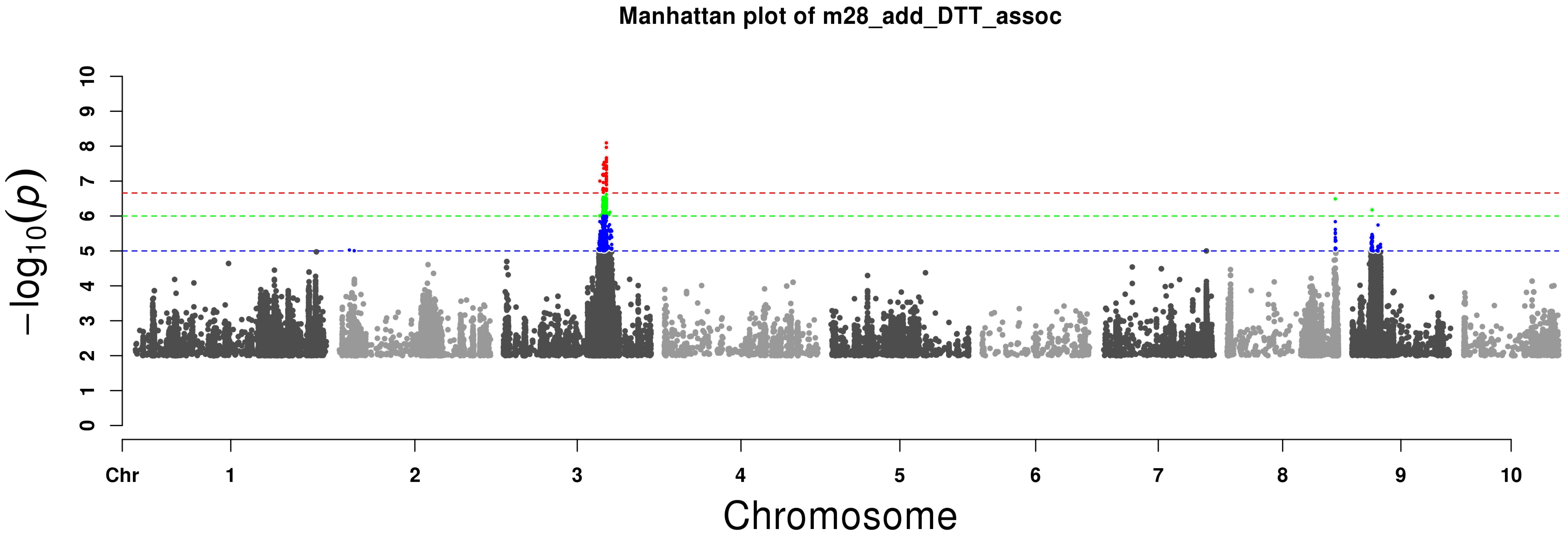
Manhattan plot of m28_add_DTT_assoc
$-\log_{10}(p)$
0
1
2
3
4
5
6
7
8
9
10
Chr
1
2
3
4
5
6
7
8
9
10
Chromosome

Manhattan plot of MG_1525_add_DTT_assoc

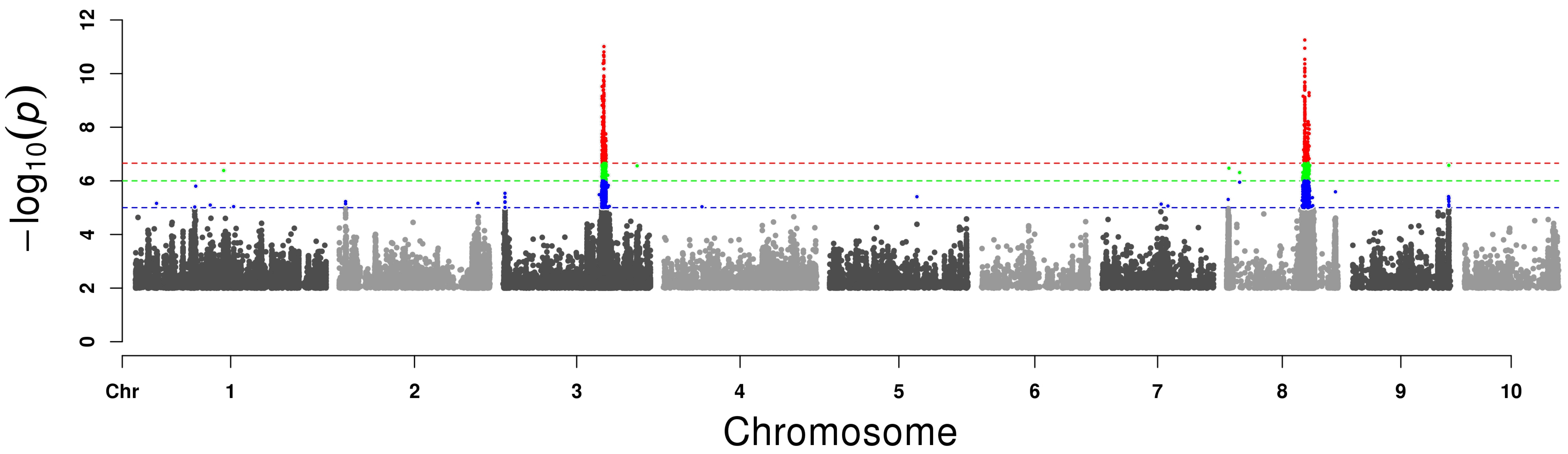

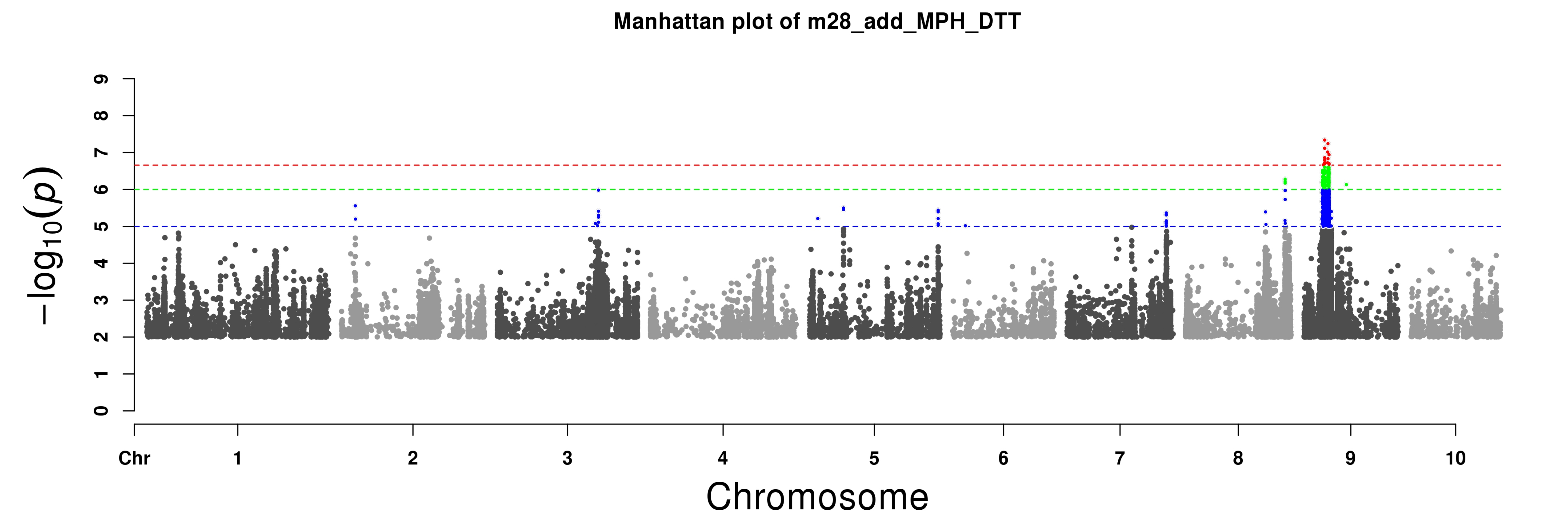

Manhattan plot of m28_add_MPH_DTT
$-\log_{10}(p)$
0
1
2
3
4
5
6
7
8
9
Chr
1
2
3
4
5
6
7
8
9
10
Chromosome

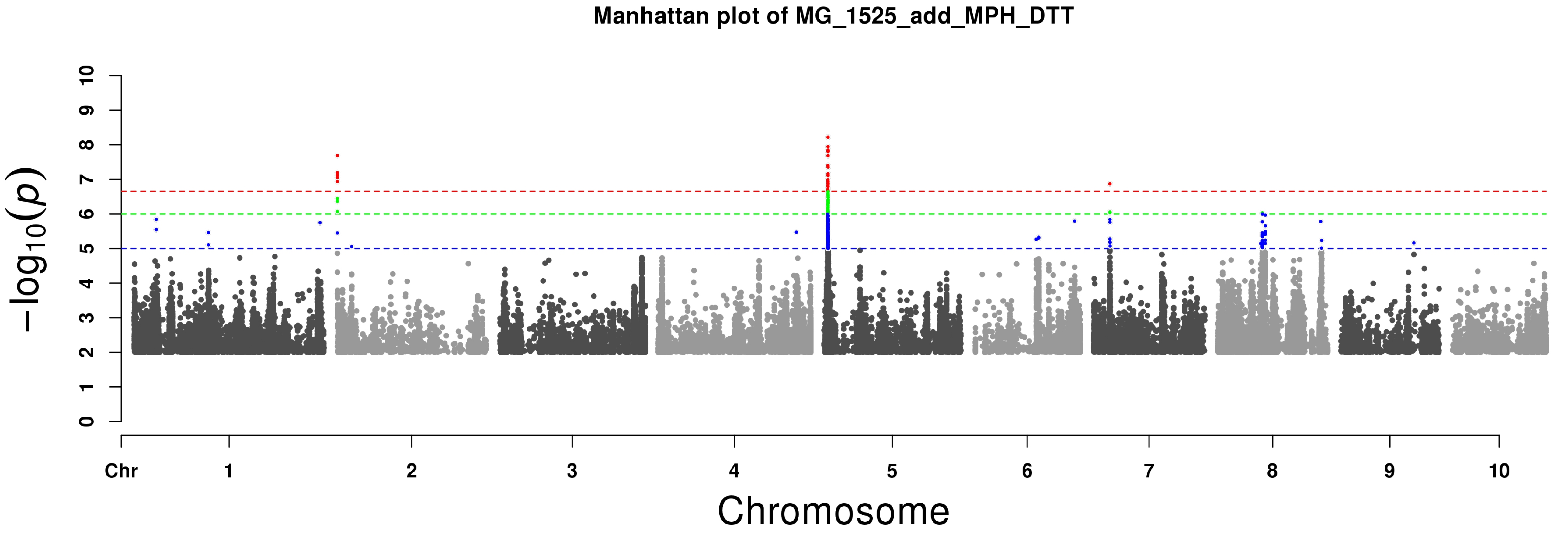

Manhattan plot of MG_1525_add_MPH_DTT
$-\log_{10}(p)$
0
1
2
3
4
5
6
7
8
9
10
Chr
1
2
3
4
5
6
7
8
9
10
Chromosome

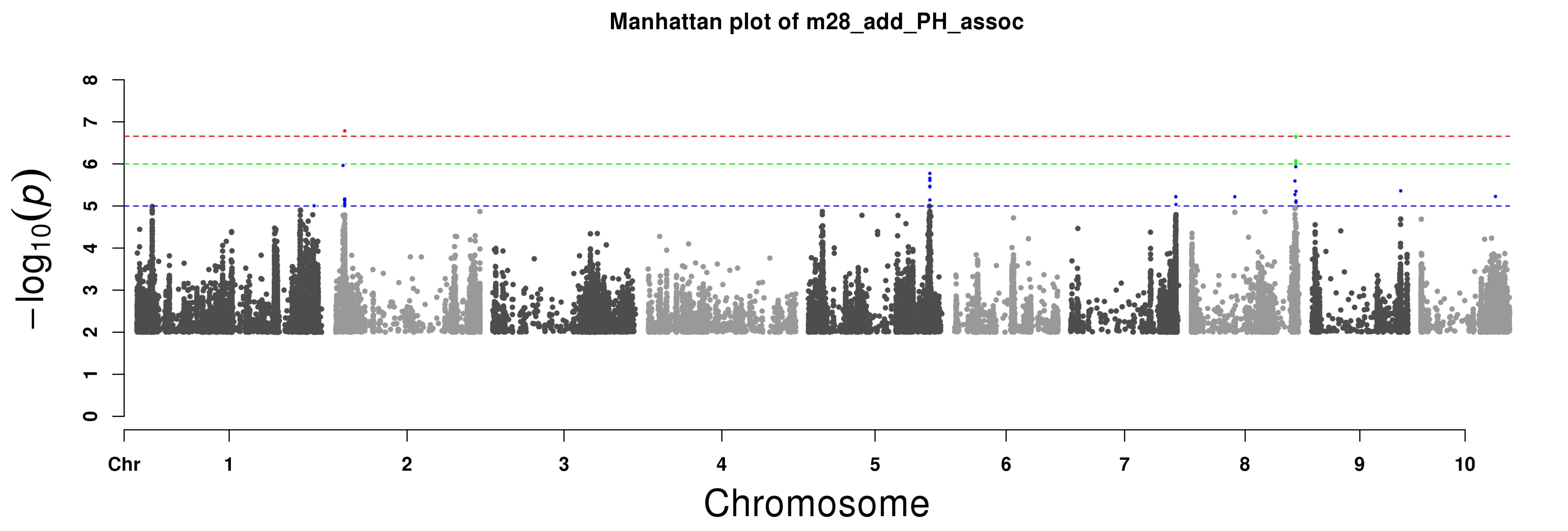
Manhattan plot of m28_add_PH_assoc
$-\log_{10}(p)$
0
1
2
3
4
5
6
7
8
Chr
1
2
3
4
5
6
7
8
9
10
Chromosome

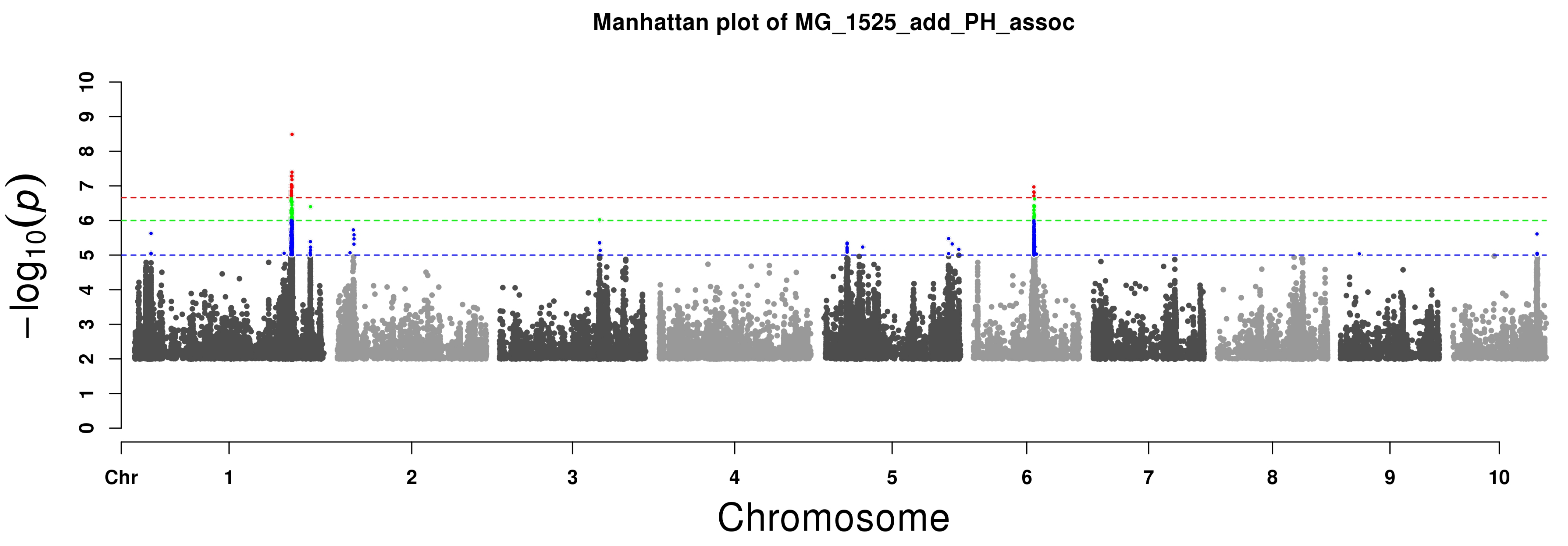

Manhattan plot of MG_1525_add_PH_assoc
$-\log_{10}(p)$
0
1
2
3
4
5
6
7
8
9
10
Chr
1
2
3
4
5
6
7
8
9
10
Chromosome

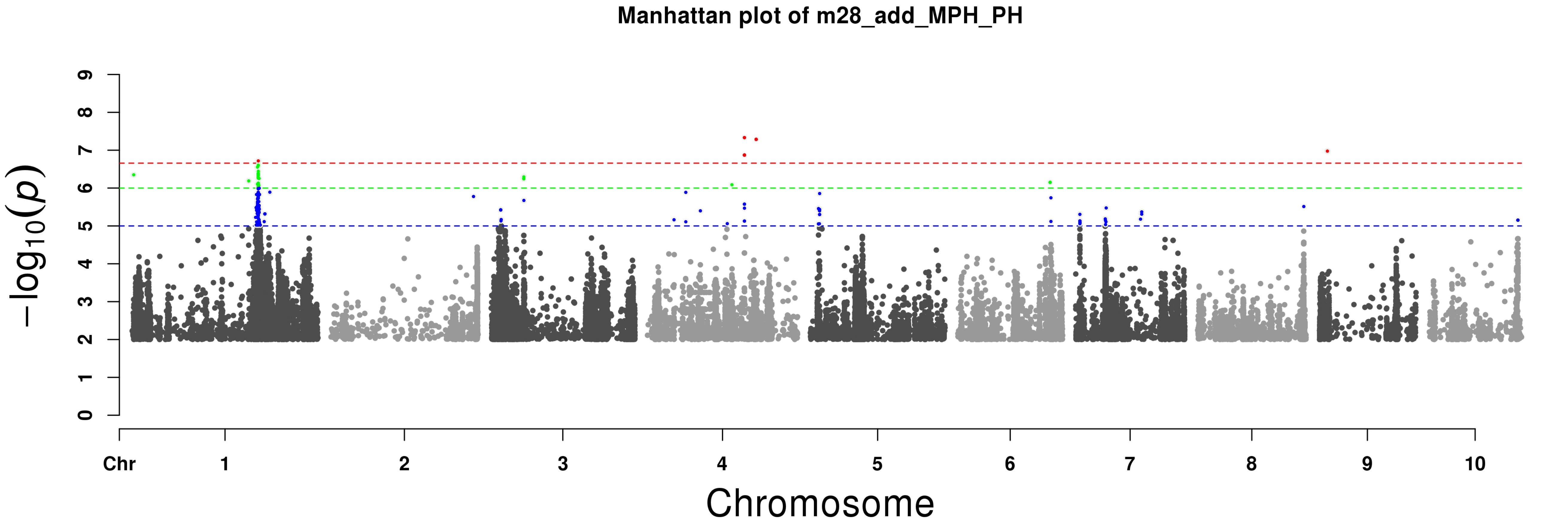

Manhattan plot of m28_add_MPH_PH
$-\log_{10}(p)$
0
1
2
3
4
5
6
7
8
9
Chr
1
2
3
4
5
6
7
8
9
10
Chromosome

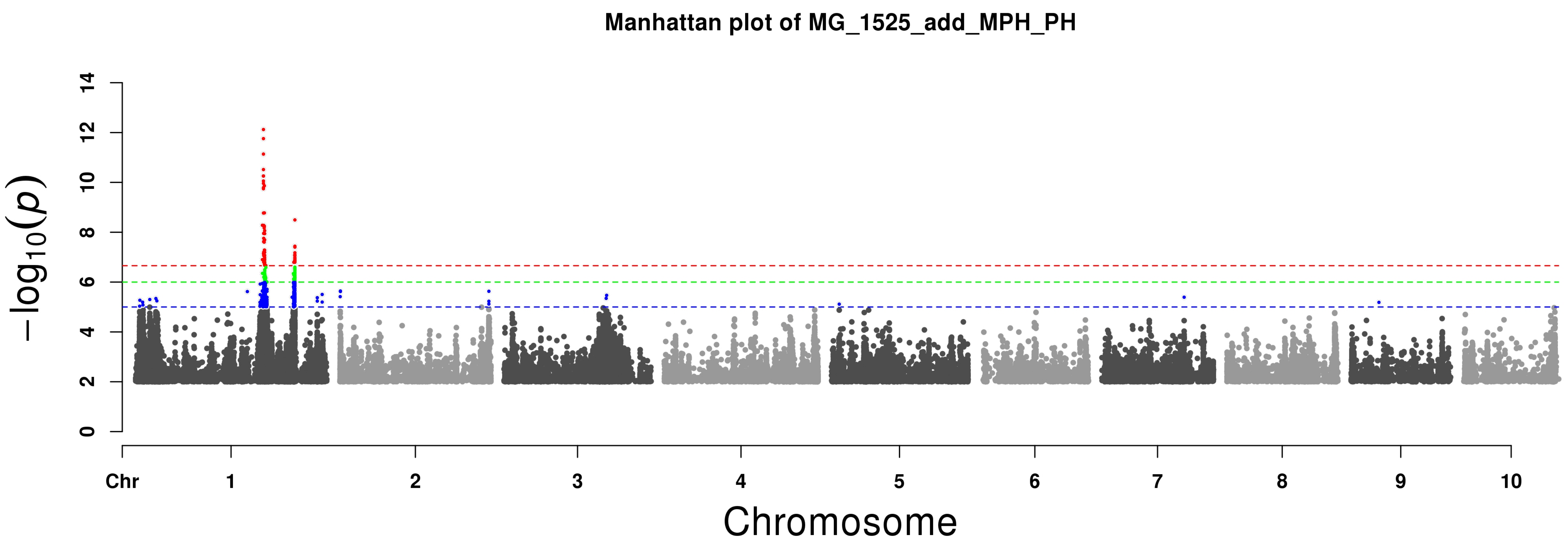

Manhattan plot of MG_1525_add_MPH_PH
$-\log_{10}(p)$
0
2
4
6
8
10
12
14
Chr
1
2
3
4
5
6
7
8
9
10
Chromosome

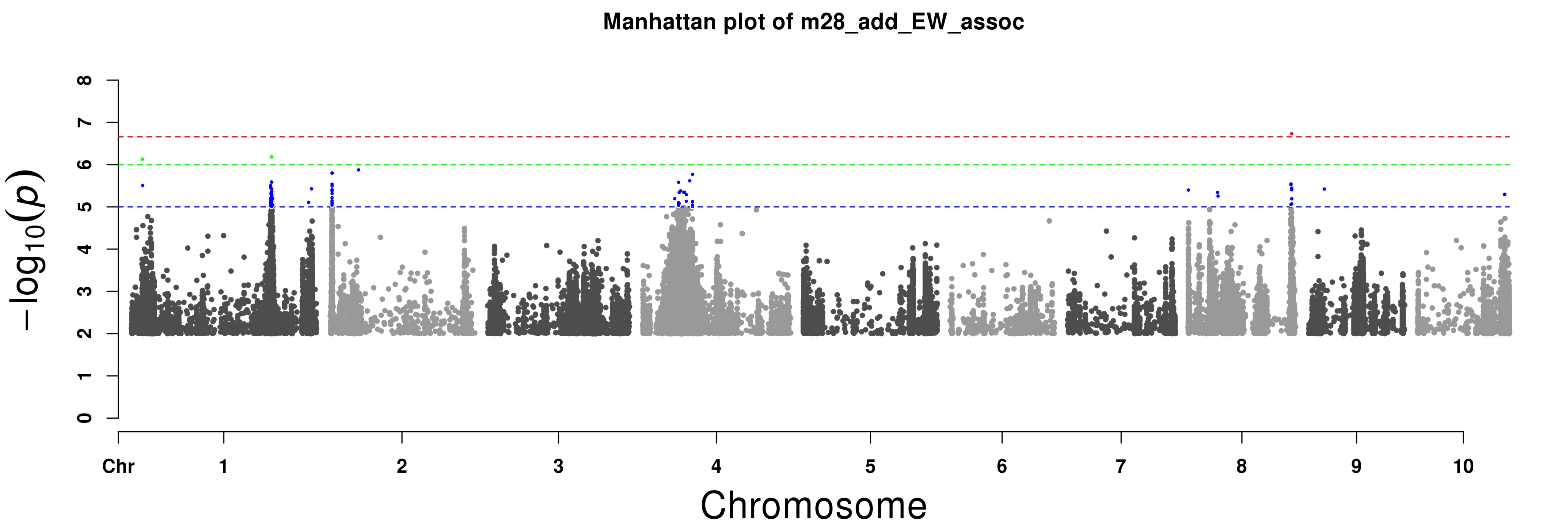

Manhattan plot of m28_add_EW_assoc
$-\log_{10}(p)$
0
1
2
3
4
5
6
7
8
Chr
1
2
3
4
5
6
7
8
9
10
Chromosome

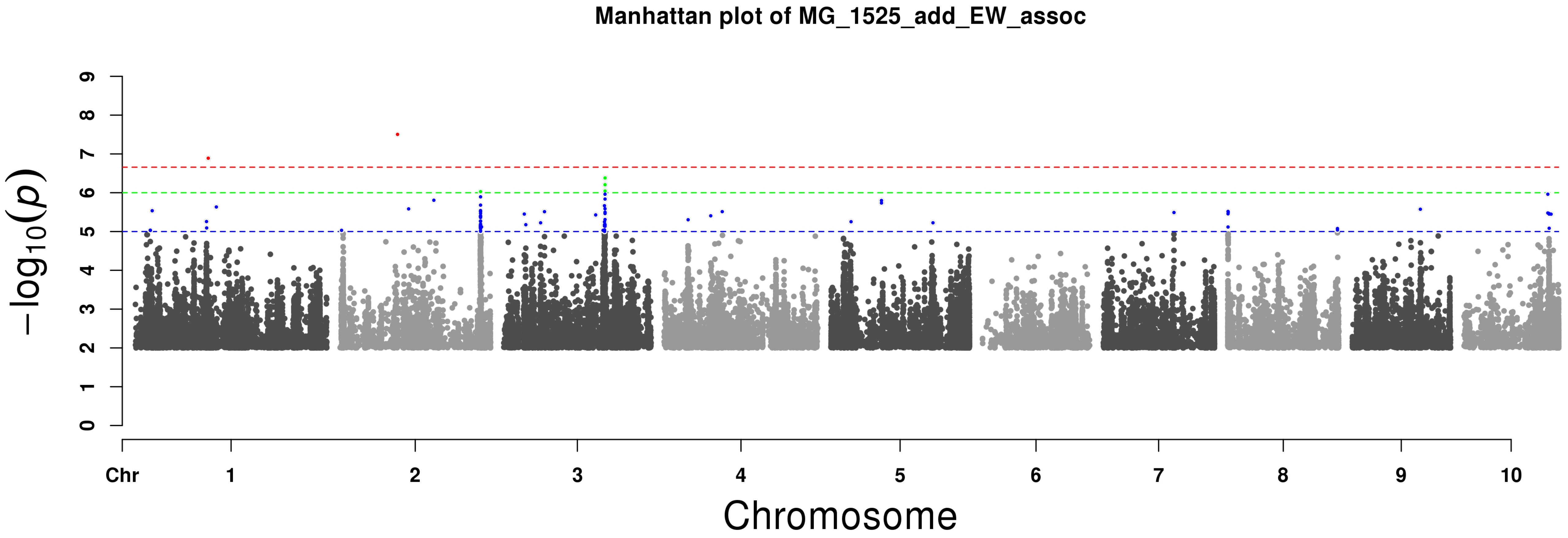

Manhattan plot of MG_1525_add_EW_assoc
$-\log_{10}(p)$
0
1
2
3
4
5
6
7
8
9
Chr
1
2
3
4
5
6
7
8
9
10
Chromosome

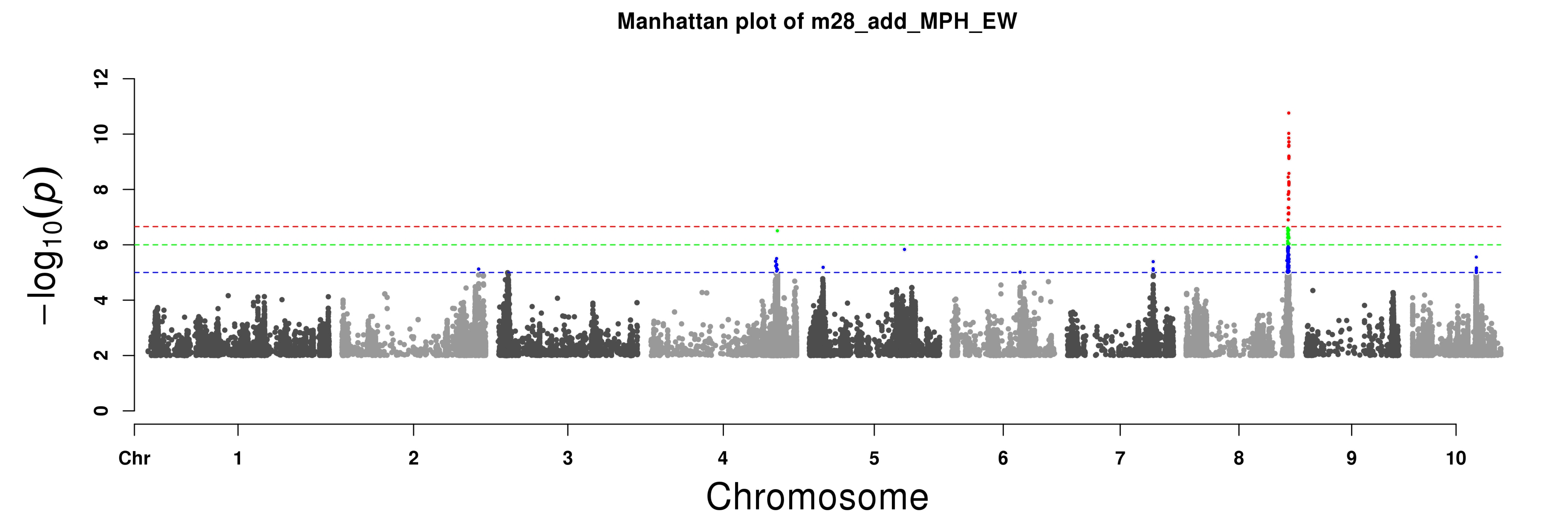

Manhattan plot of m28_add_MPH_EW
$-\log_{10}(p)$
0
2
4
6
8
10
12
Chr
1
2
3
4
5
6
7
8
9
10
Chromosome

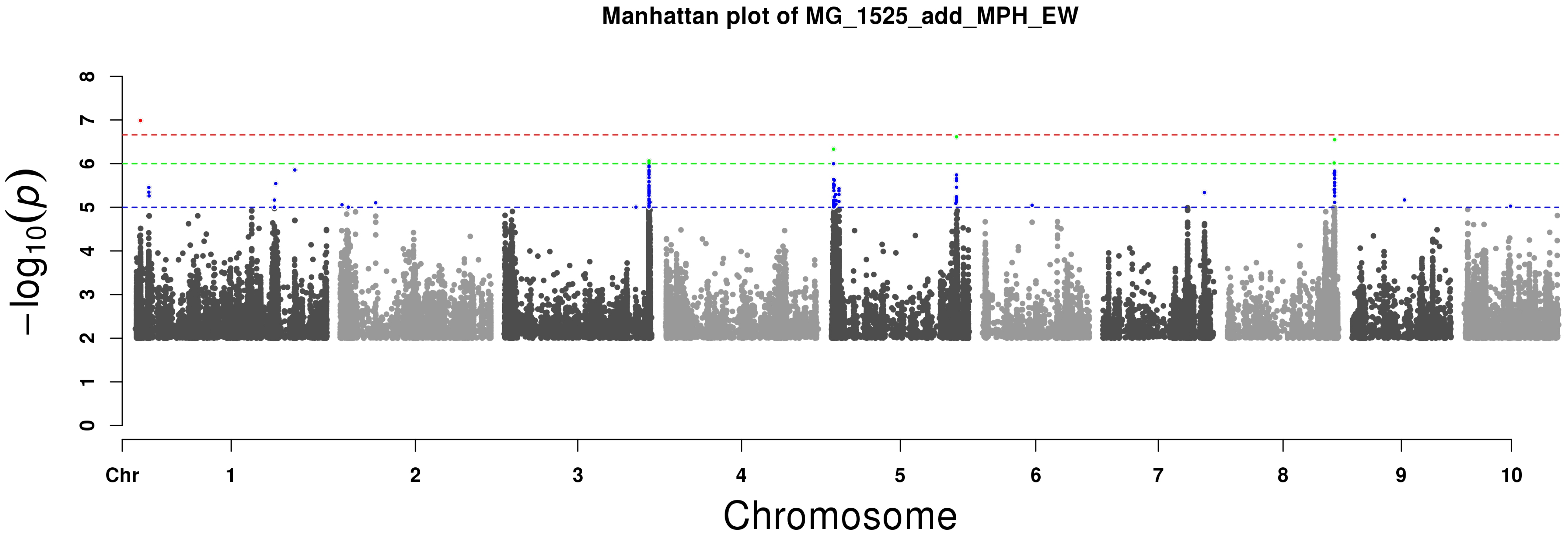
Manhattan plot of MG_1525_add_MPH_EW
$-\log_{10}(p)$
0
1
2
3
4
5
6
7
8
Chr
1
2
3
4
5
6
7
8
9
10
Chromosome

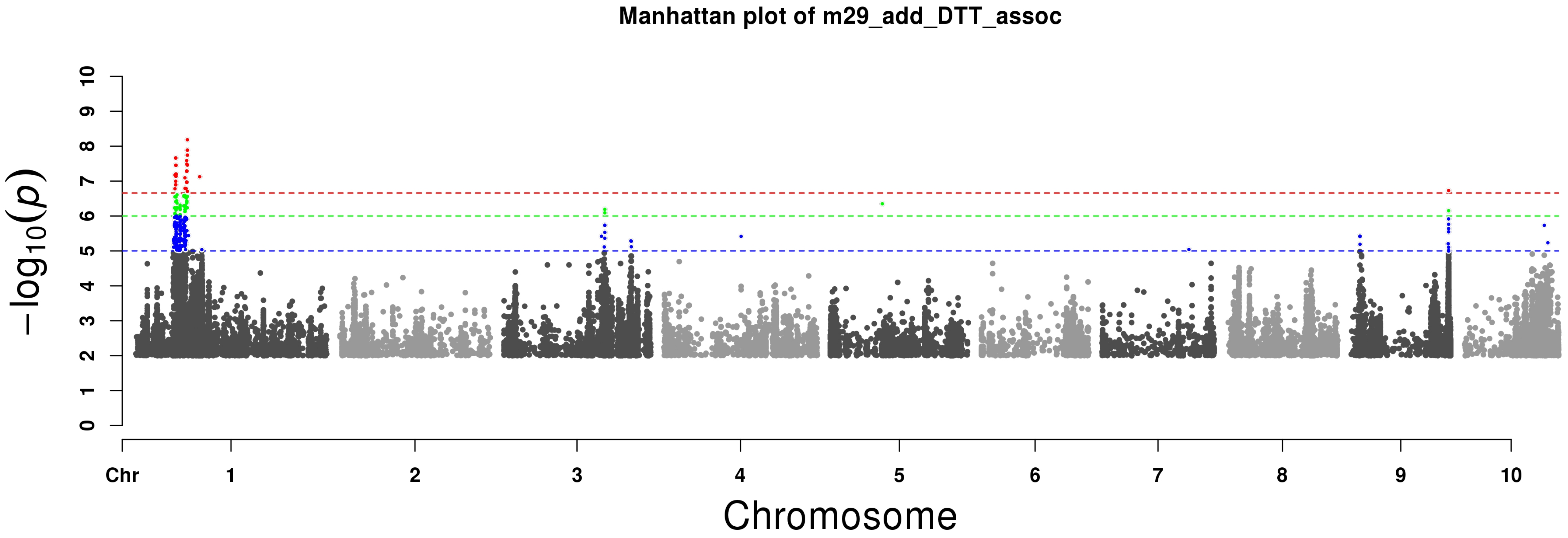
Manhattan plot of m29_add_DTT_assoc
$-\log_{10}(p)$
0
1
2
3
4
5
6
7
8
9
10
Chr
1
2
3
4
5
6
7
8
9
10
Chromosome

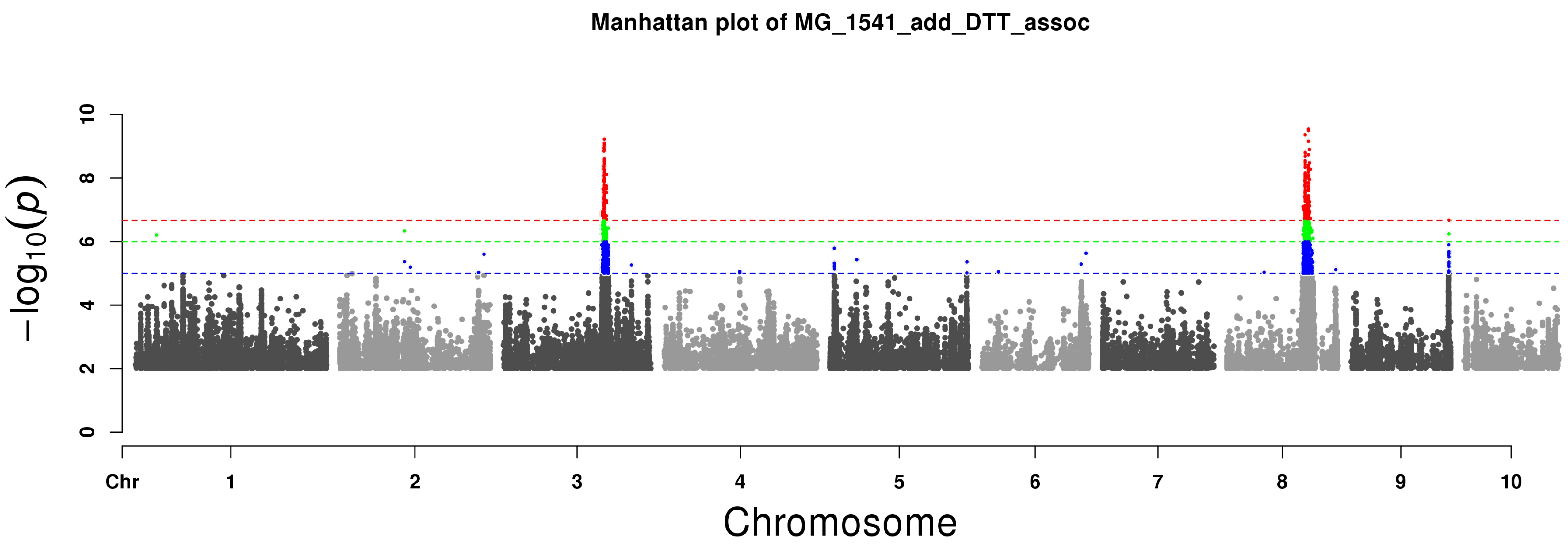

Manhattan plot of MG_1541_add_DTT_assoc
$-\log_{10}(p)$
0
2
4
6
8
10
Chr
1
2
3
4
5
6
7
8
9
10
Chromosome

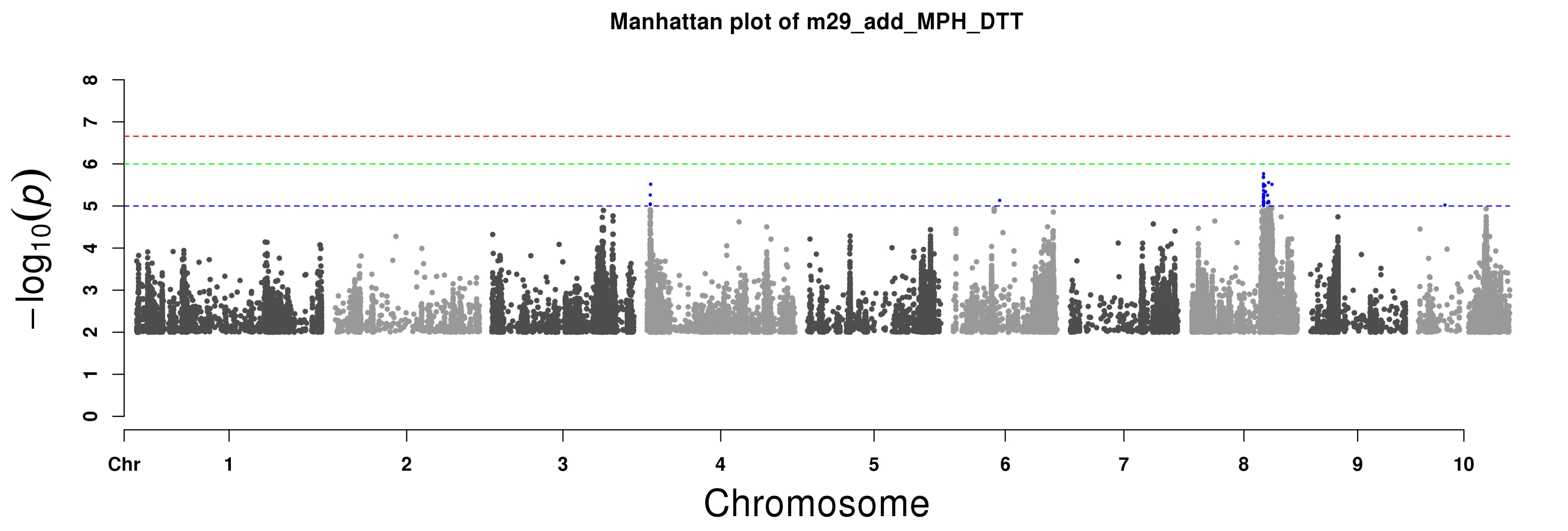
Manhattan plot of m29_add_MPH_DTT
$-\log_{10}(p)$
0
1
2
3
4
5
6
7
8
Chr
1
2
3
4
5
6
7
8
9
10
Chromosome

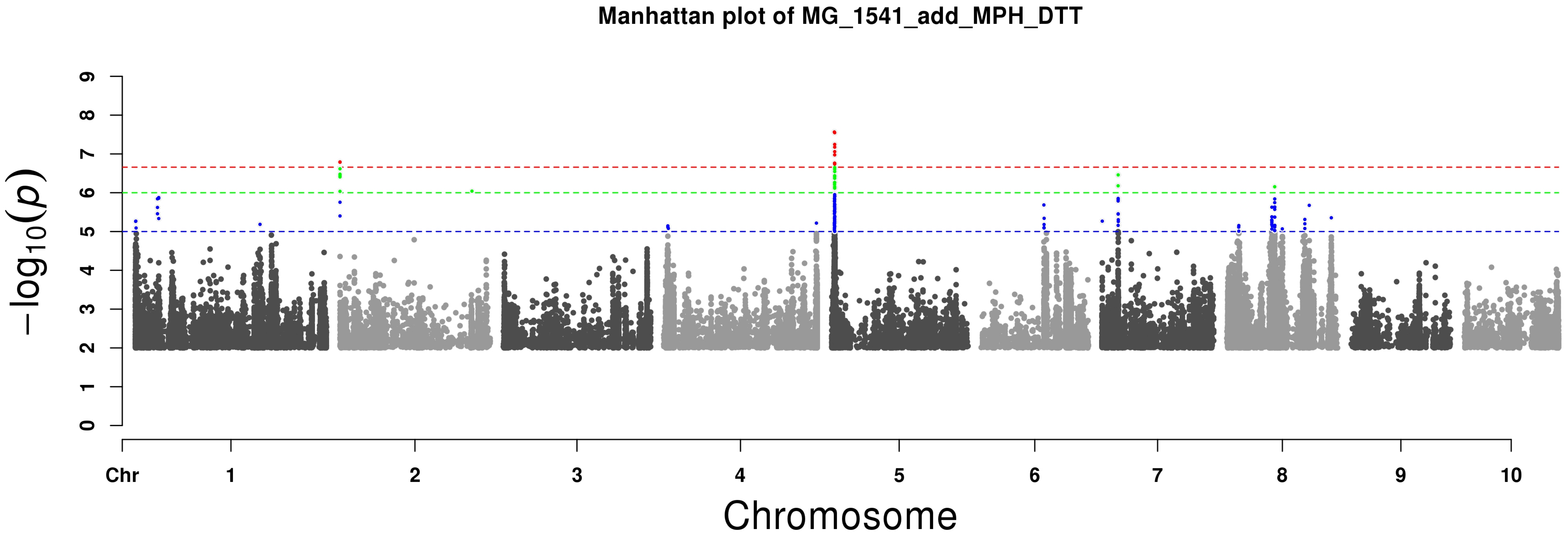

Manhattan plot of MG_1541_add_MPH_DTT
$-\log_{10}(p)$
0
1
2
3
4
5
6
7
8
9
Chr
1
2
3
4
5
6
7
8
9
10
Chromosome

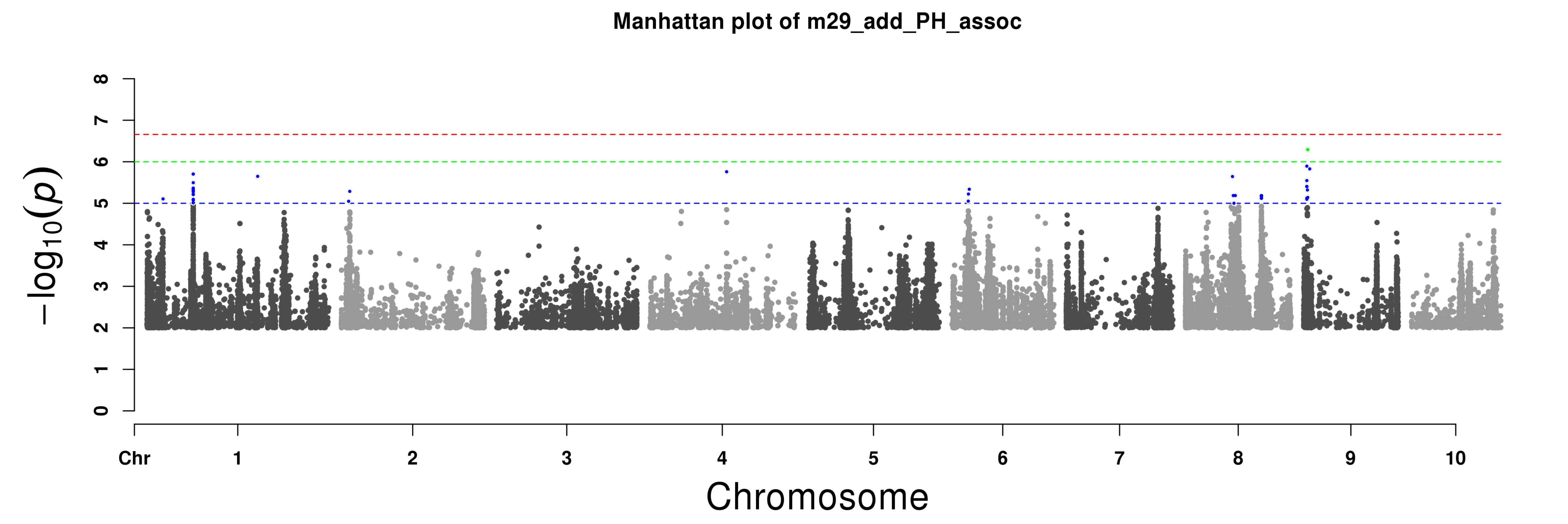

Manhattan plot of m29_add_PH_assoc
$-\log_{10}(p)$
0
1
2
3
4
5
6
7
8
Chr
1
2
3
4
5
6
7
8
9
10
Chromosome

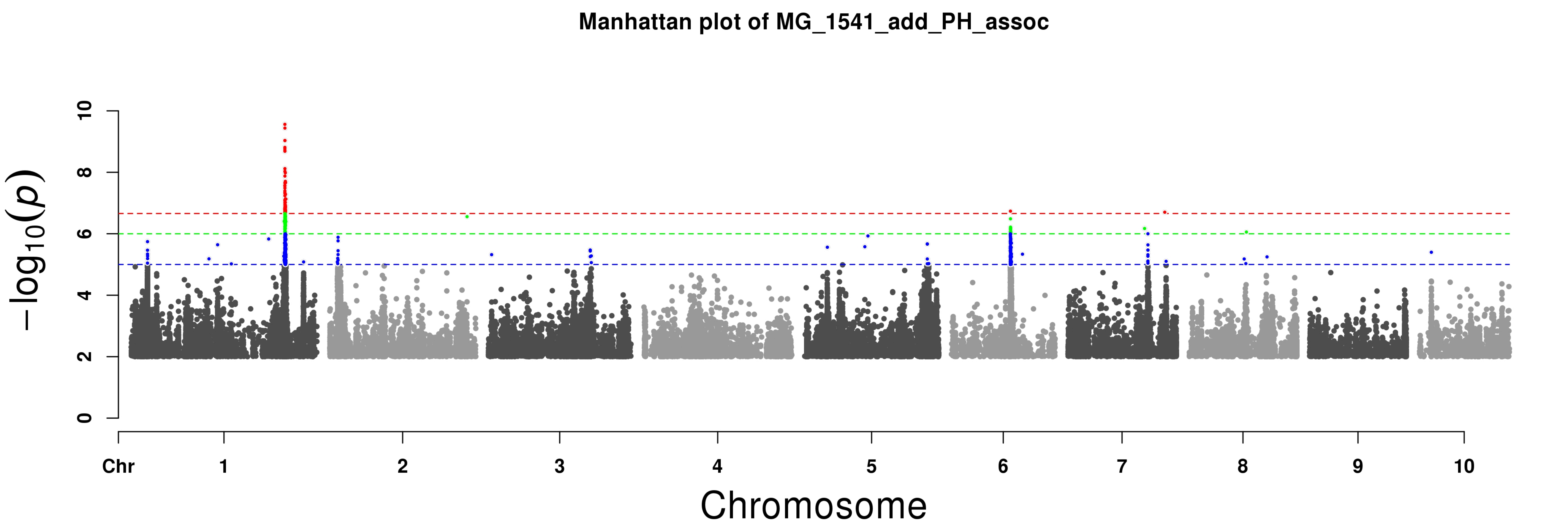

Manhattan plot of MG_1541_add_PH_assoc
10
8
6
4
2
0
$-\log_{10}(p)$
Chr
1
2
3
4
5
6
7
8
9
10
Chromosome

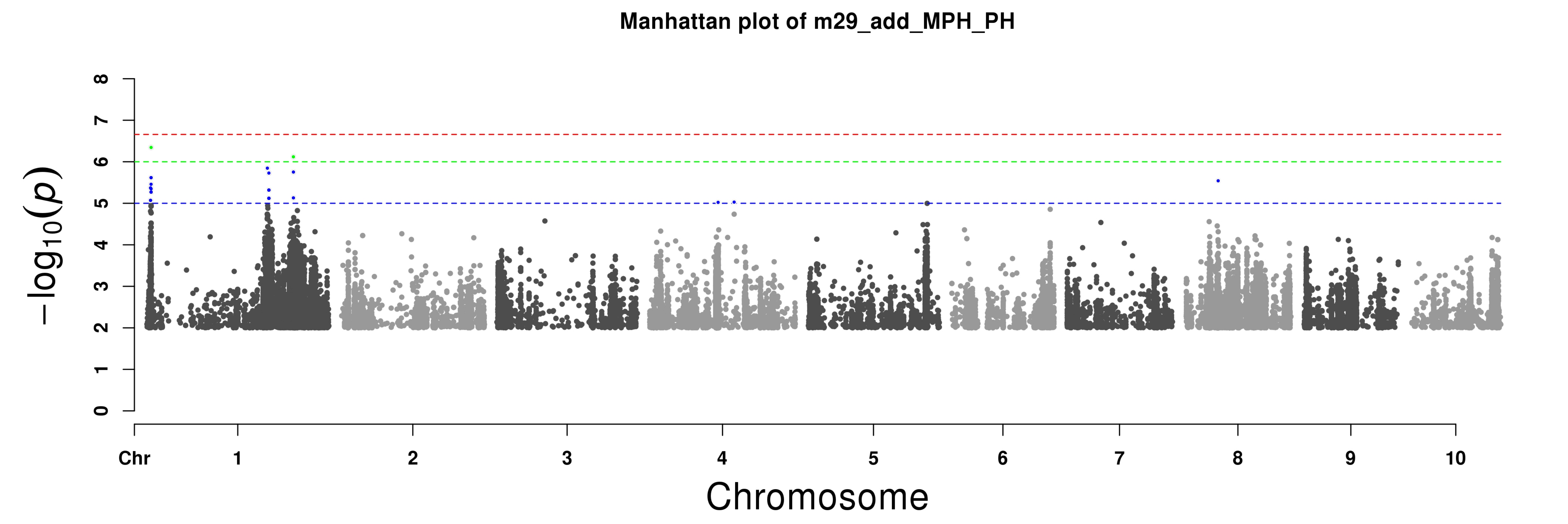

Manhattan plot of m29_add_MPH_PH
$-\log_{10}(p)$
0
1
2
3
4
5
6
7
8
Chr
1
2
3
4
5
6
7
8
9
10
Chromosome

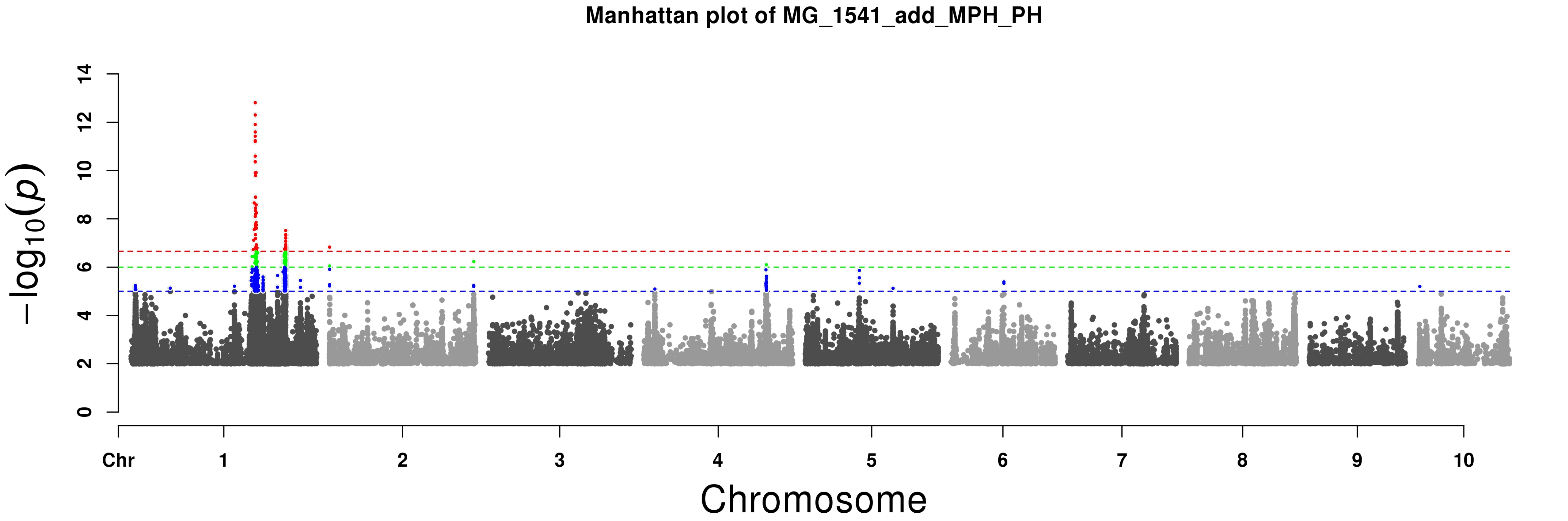
Manhattan plot of MG_1541_add_MPH_PH
$-\log_{10}(p)$
0
2
4
6
8
10
12
14
Chr
1
2
3
4
5
6
7
8
9
10
Chromosome

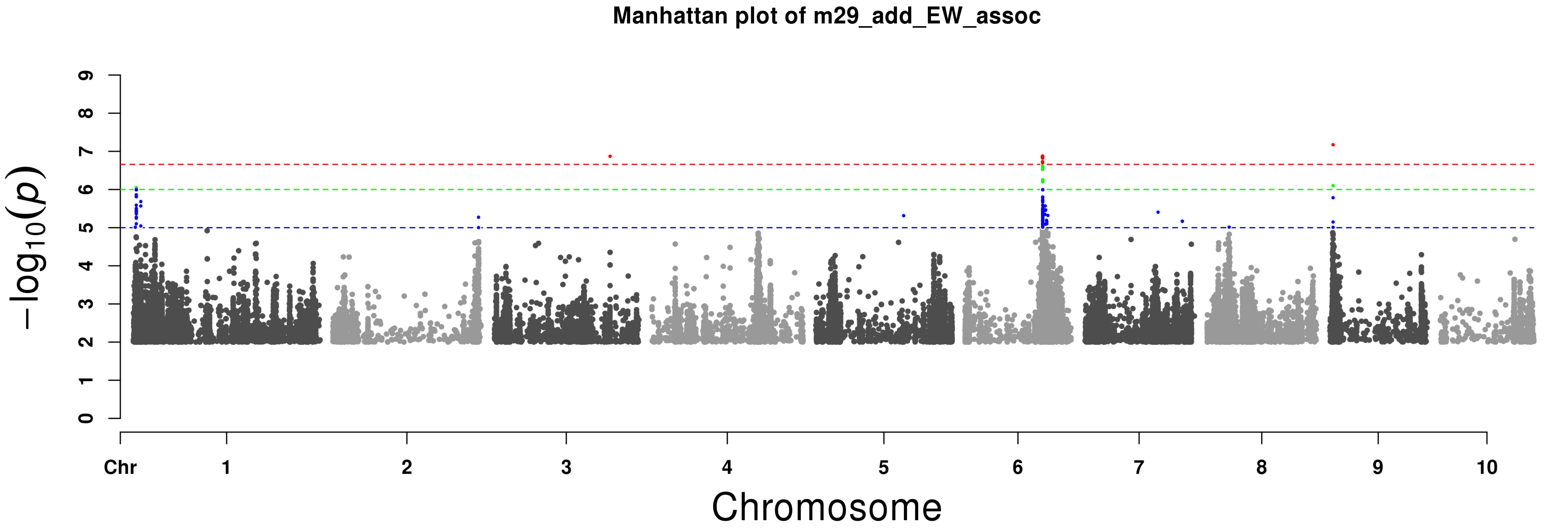
Manhattan plot of m29_add_EW_assoc
9
8
7
6
5
4
3
2
1
0
$-\log_{10}(p)$
Chr
1
2
3
4
5
6
7
8
9
10
Chromosome

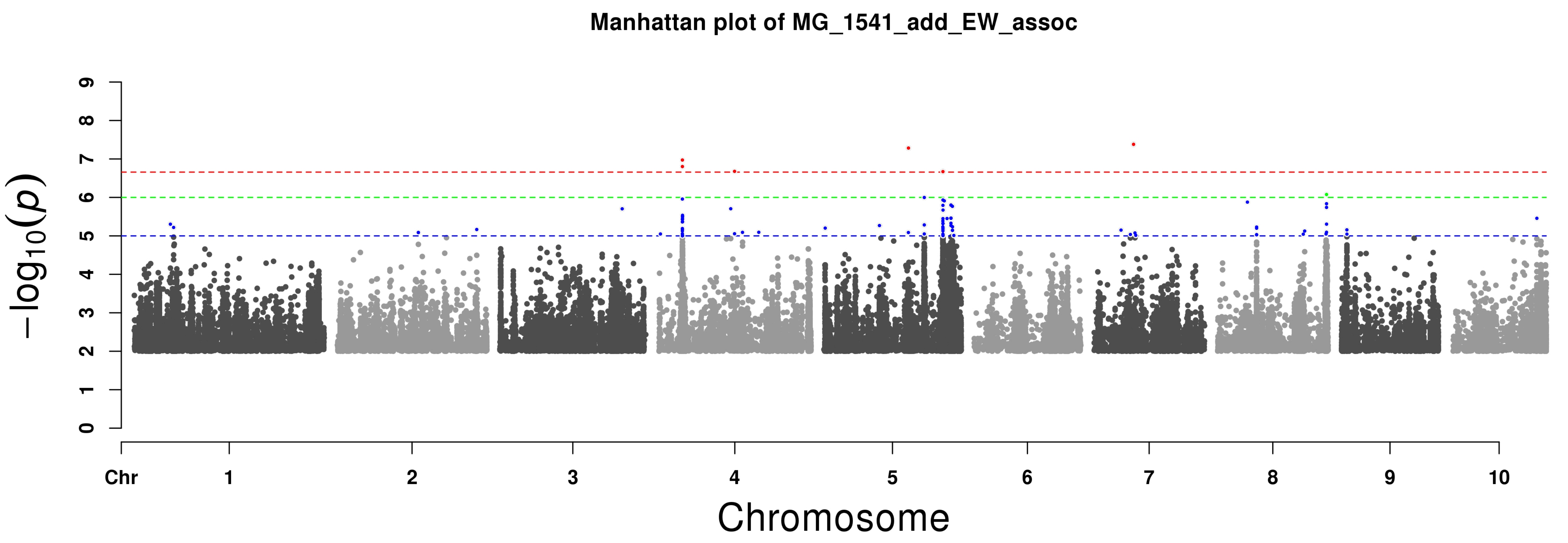

Manhattan plot of MG_1541_add_EW_assoc
−log10(p)
0
1
2
3
4
5
6
7
8
9
Chr
1
2
3
4
5
6
7
8
9
10
Chromosome

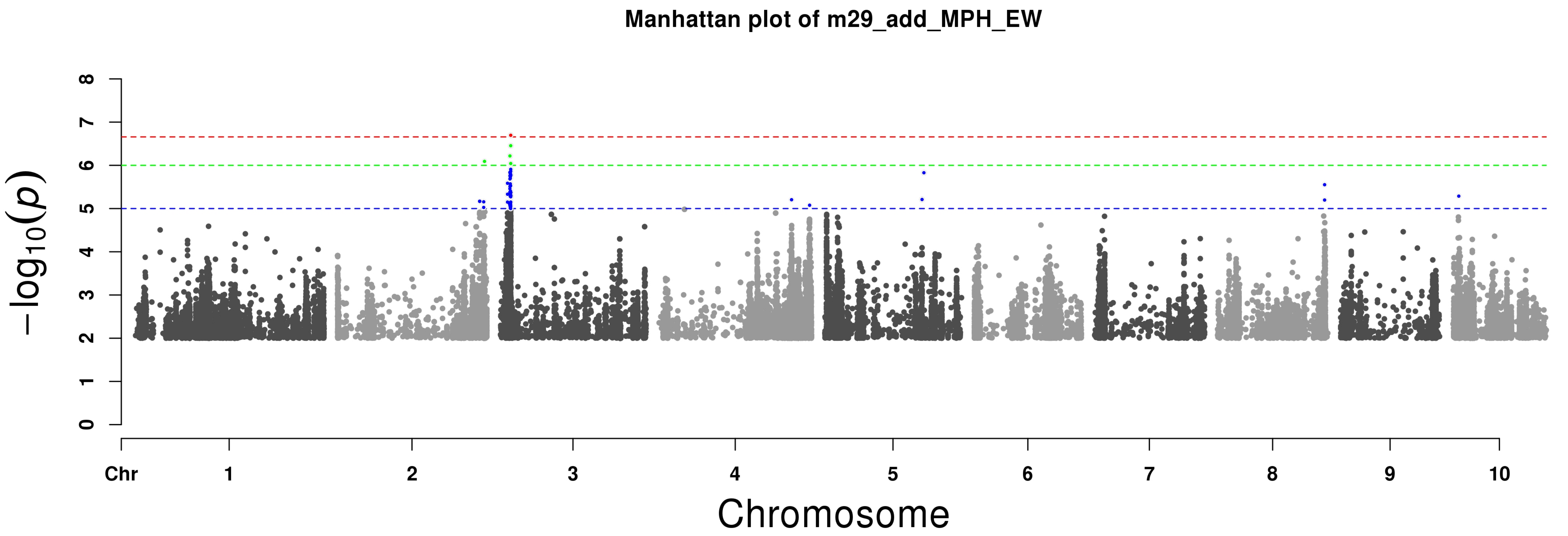

Manhattan plot of m29_add_MPH_EW
−log10(p)
0
1
2
3
4
5
6
7
8
Chr
1
2
3
4
5
6
7
8
9
10
Chromosome

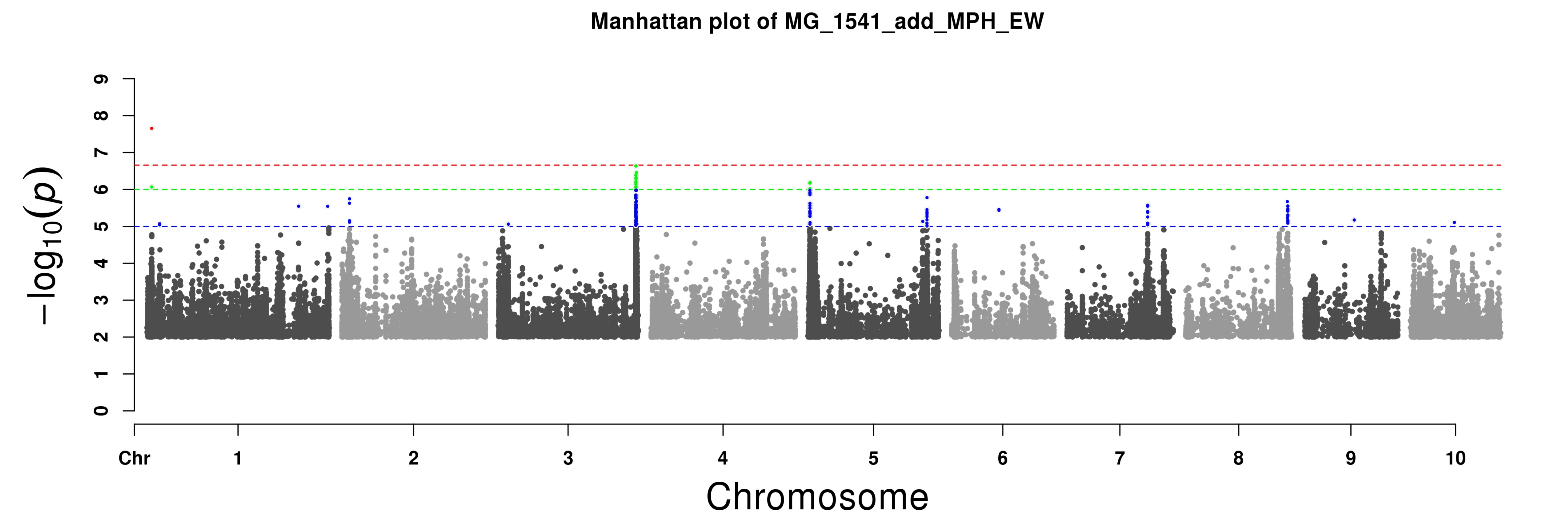
Manhattan plot of MG_1541_add_MPH_EW
$-\log_{10}(p)$
0
1
2
3
4
5
6
7
8
9
Chr
1
2
3
4
5
6
7
8
9
10
Chromosome

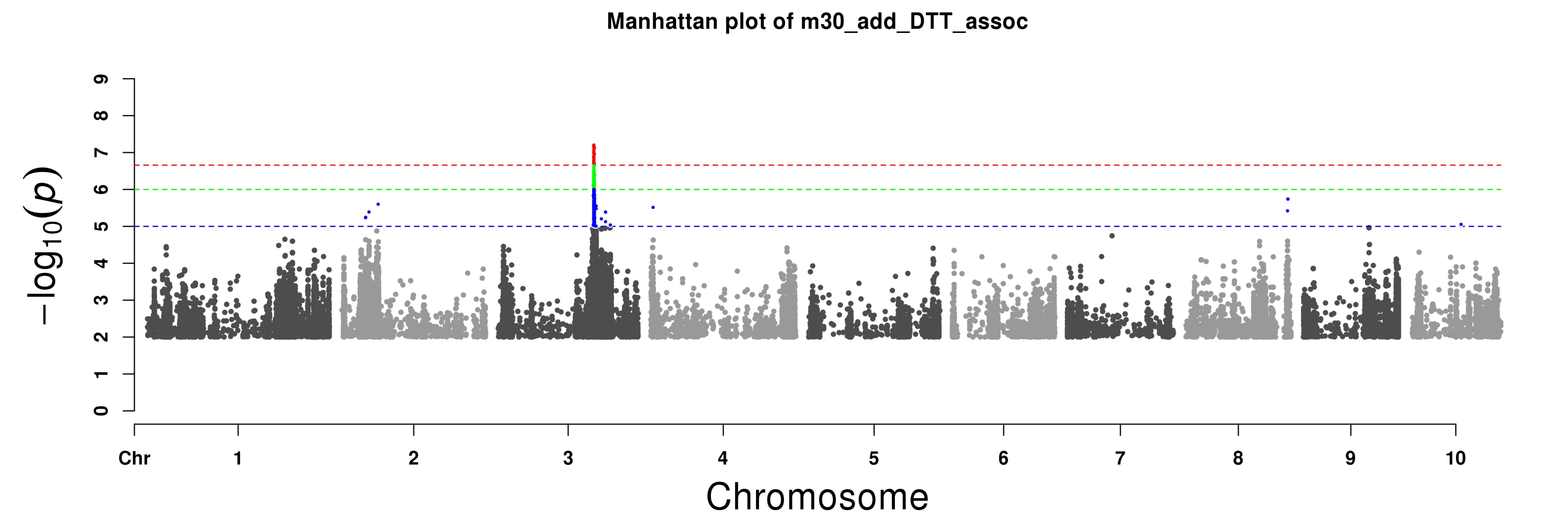
Manhattan plot of m30_add_DTT_assoc
$-\log_{10}(p)$
0
1
2
3
4
5
6
7
8
9
Chr
1
2
3
4
5
6
7
8
9
10
Chromosome

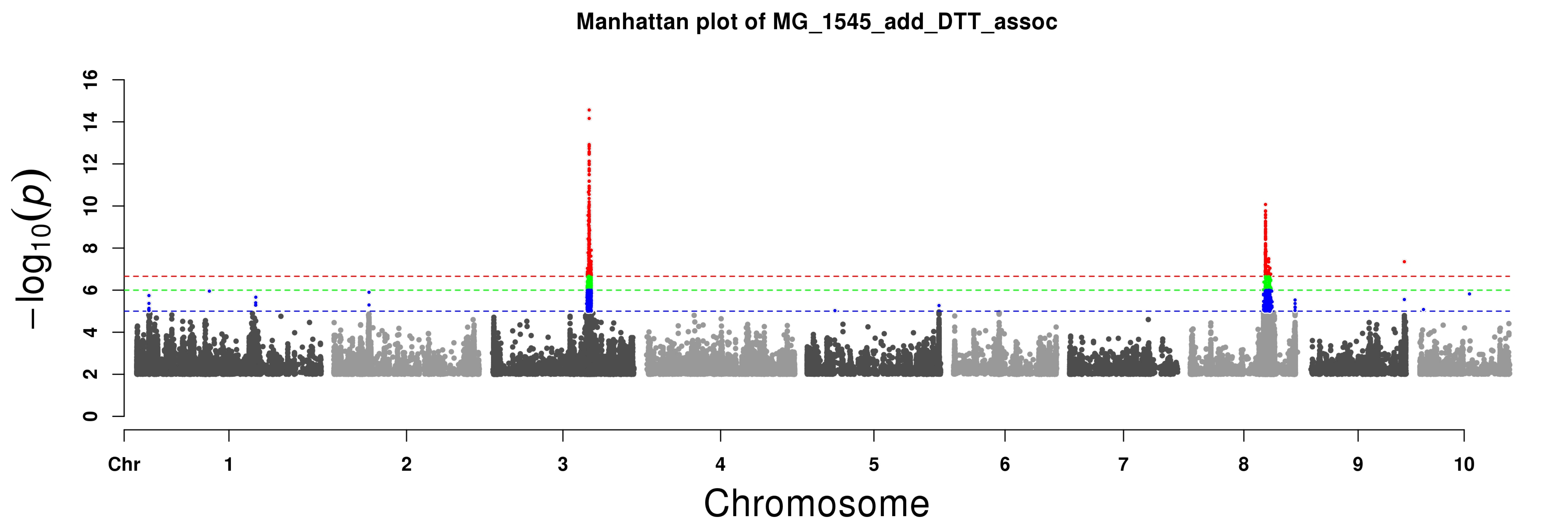

Manhattan plot of MG_1545_add_DTT_assoc
$-\log_{10}(p)$
0
2
4
6
8
10
12
14
16
Chr
1
2
3
4
5
6
7
8
9
10
Chromosome

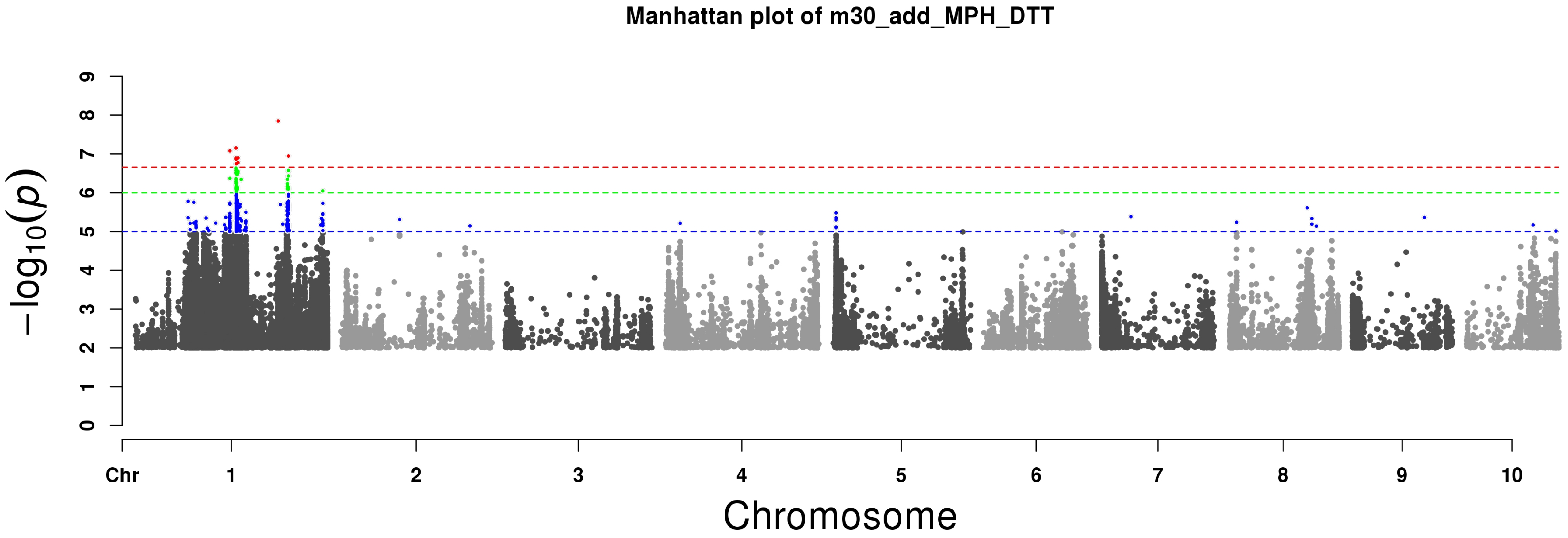

Manhattan plot of m30_add_MPH_DTT
$-\log_{10}(p)$
0
1
2
3
4
5
6
7
8
9
Chr
1
2
3
4
5
6
7
8
9
10
Chromosome

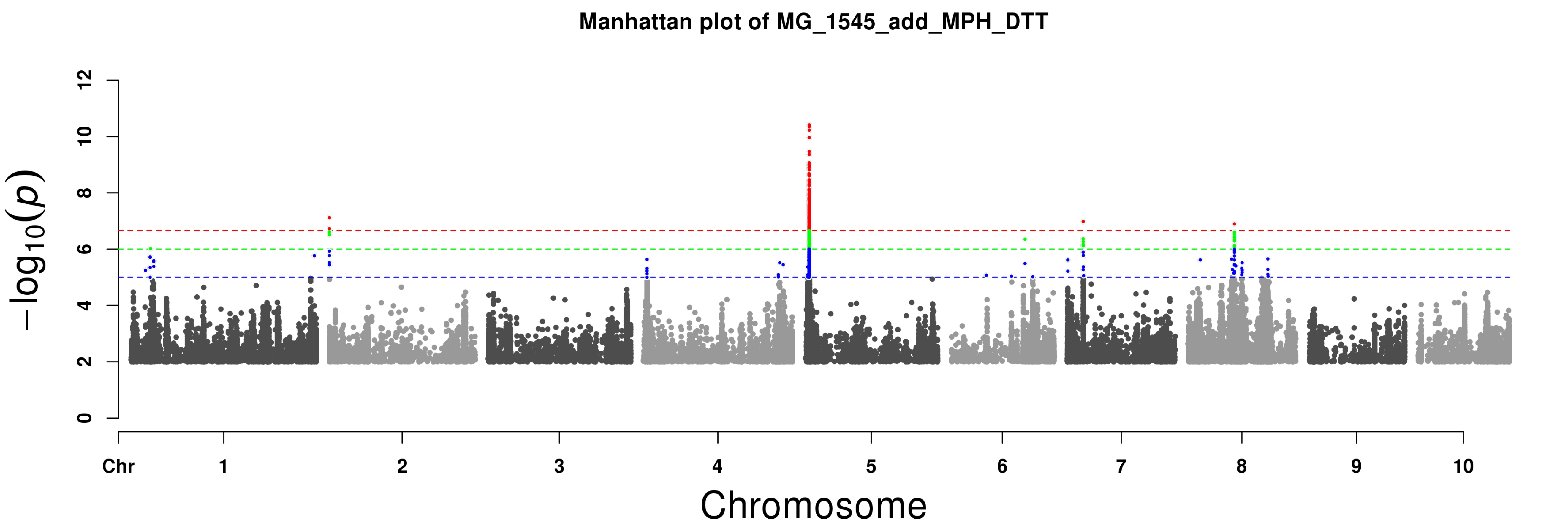

Manhattan plot of MG_1545_add_MPH_DTT
$-\log_{10}(p)$
0
2
4
6
8
10
12
Chr
1
2
3
4
5
6
7
8
9
10
Chromosome

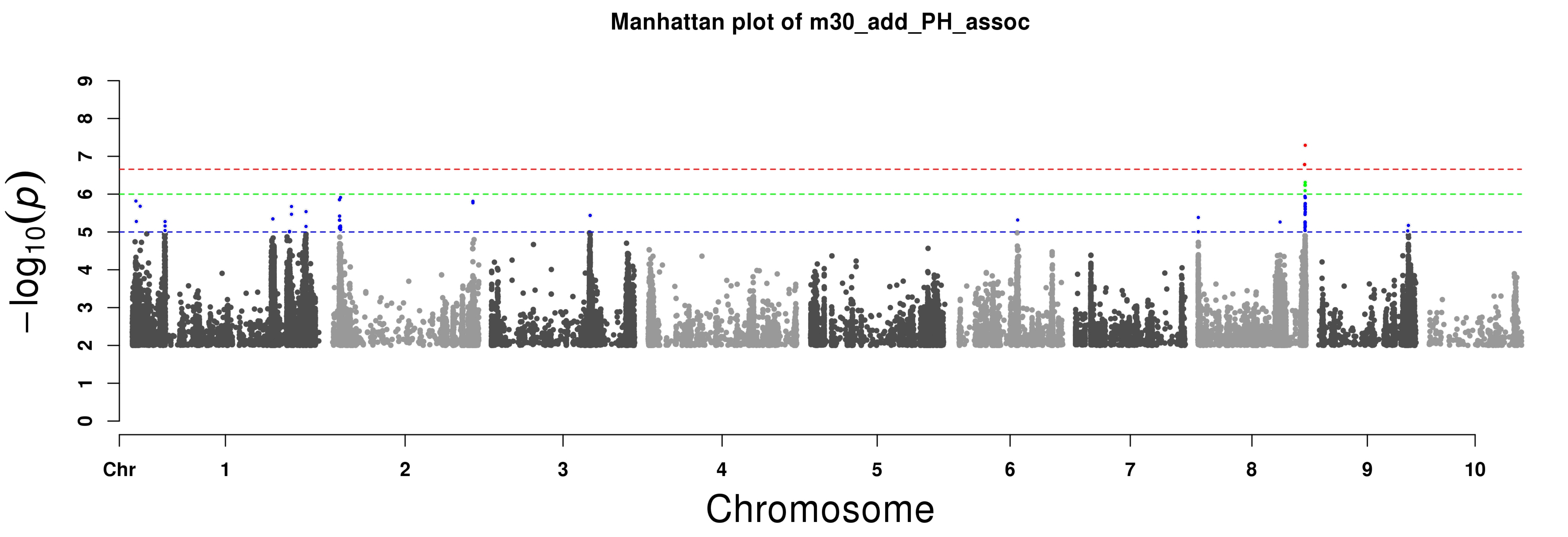
Manhattan plot of m30_add_PH_assoc
$-\log_{10}(p)$
0
1
2
3
4
5
6
7
8
9
Chr
1
2
3
4
5
6
7
8
9
10
Chromosome

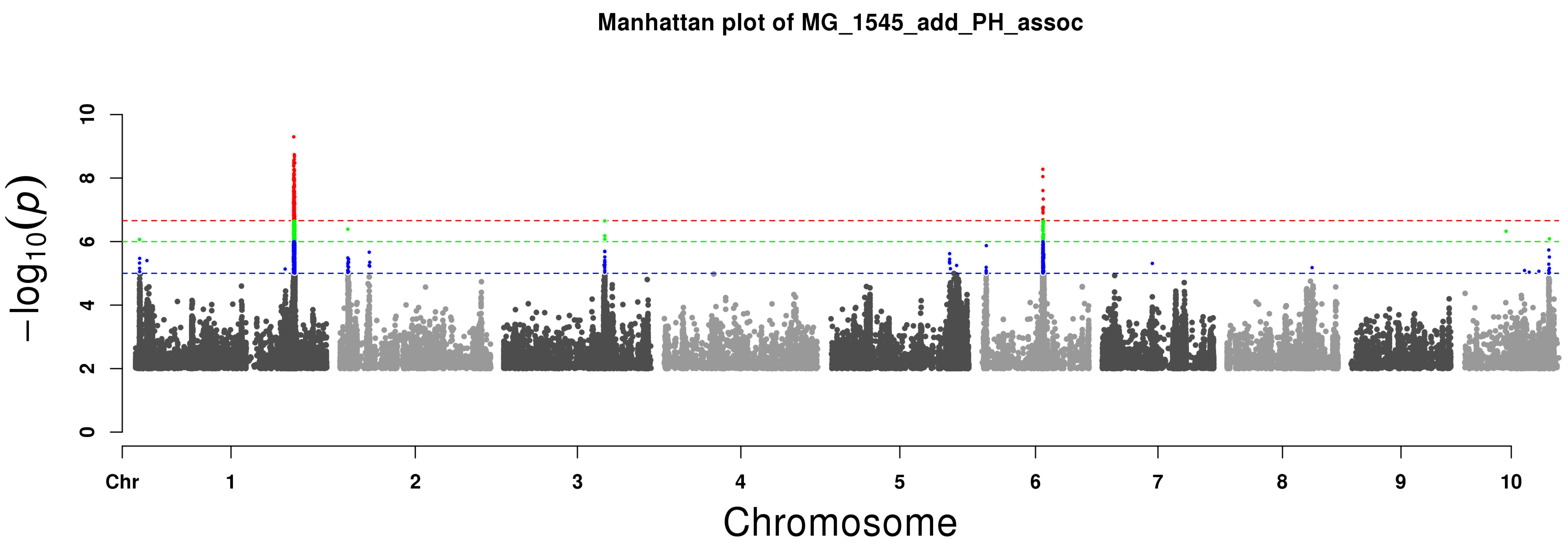

Manhattan plot of MG_1545_add_PH_assoc
$-\log_{10}(p)$
0
2
4
6
8
10
Chr
1
2
3
4
5
6
7
8
9
10
Chromosome

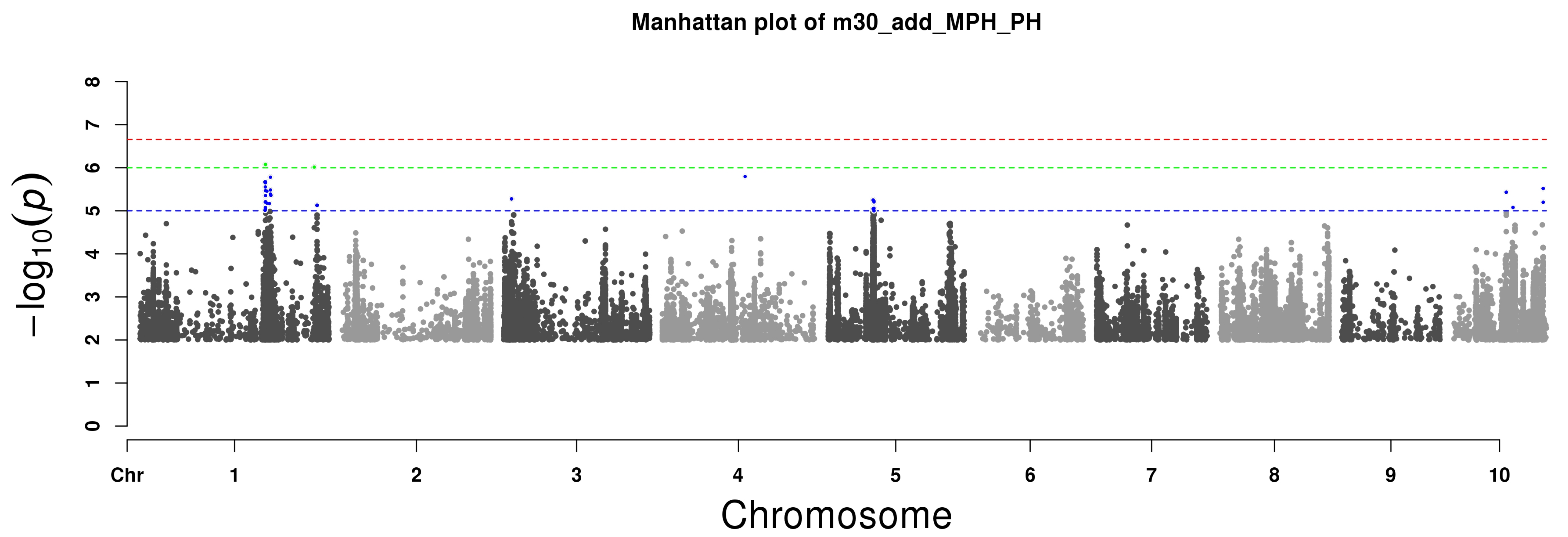

Manhattan plot of m30_add_MPH_PH
$-\log_{10}(p)$
0
1
2
3
4
5
6
7
8
Chr
1
2
3
4
5
6
7
8
9
10
Chromosome

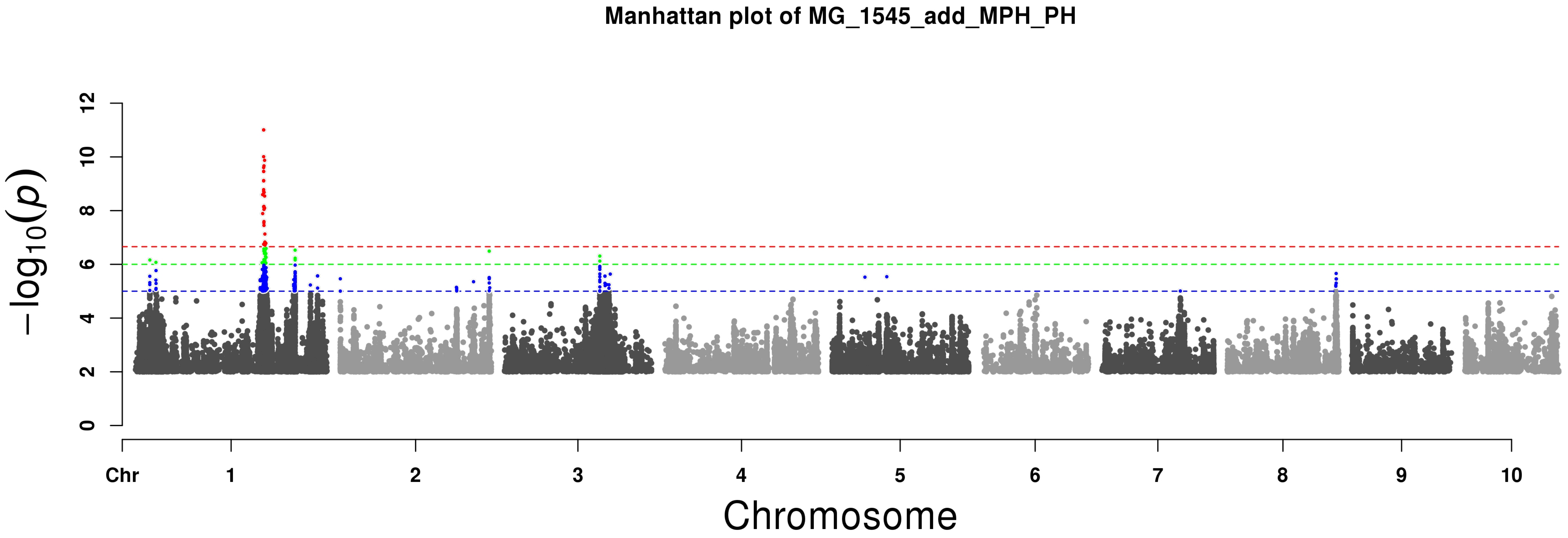
Manhattan plot of MG_1545_add_MPH_PH
$-\log_{10}(p)$
0
2
4
6
8
10
12
Chr
1
2
3
4
5
6
7
8
9
10
Chromosome

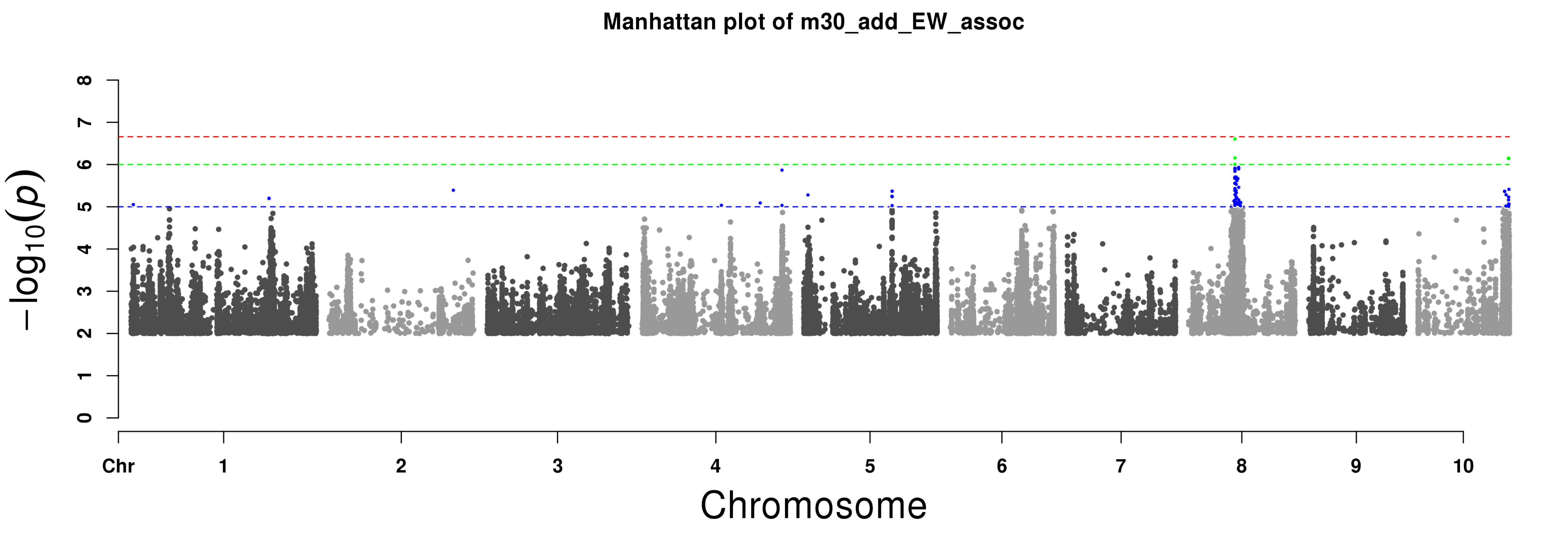
Manhattan plot of m30_add_EW_assoc
$-\log_{10}(p)$
0
1
2
3
4
5
6
7
8
Chr
1
2
3
4
5
6
7
8
9
10
Chromosome

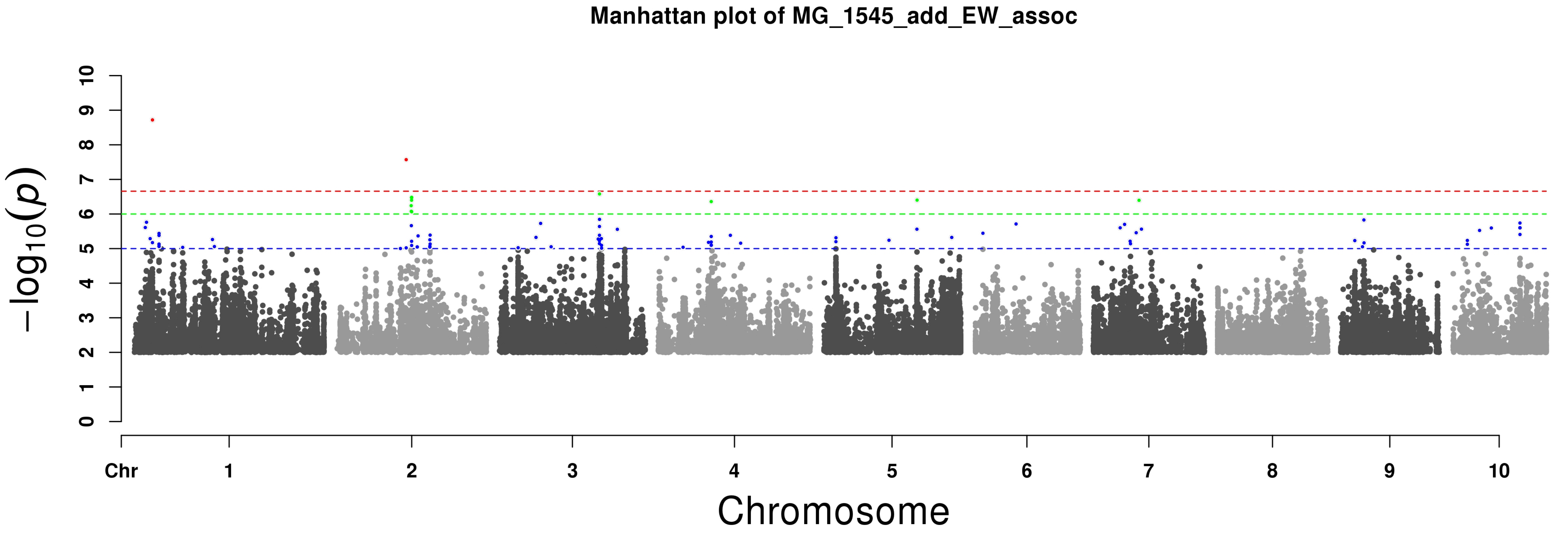
Manhattan plot of MG_1545_add_EW_assoc
$-\log_{10}(p)$
0
1
2
3
4
5
6
7
8
9
10
Chr
1
2
3
4
5
6
7
8
9
10
Chromosome

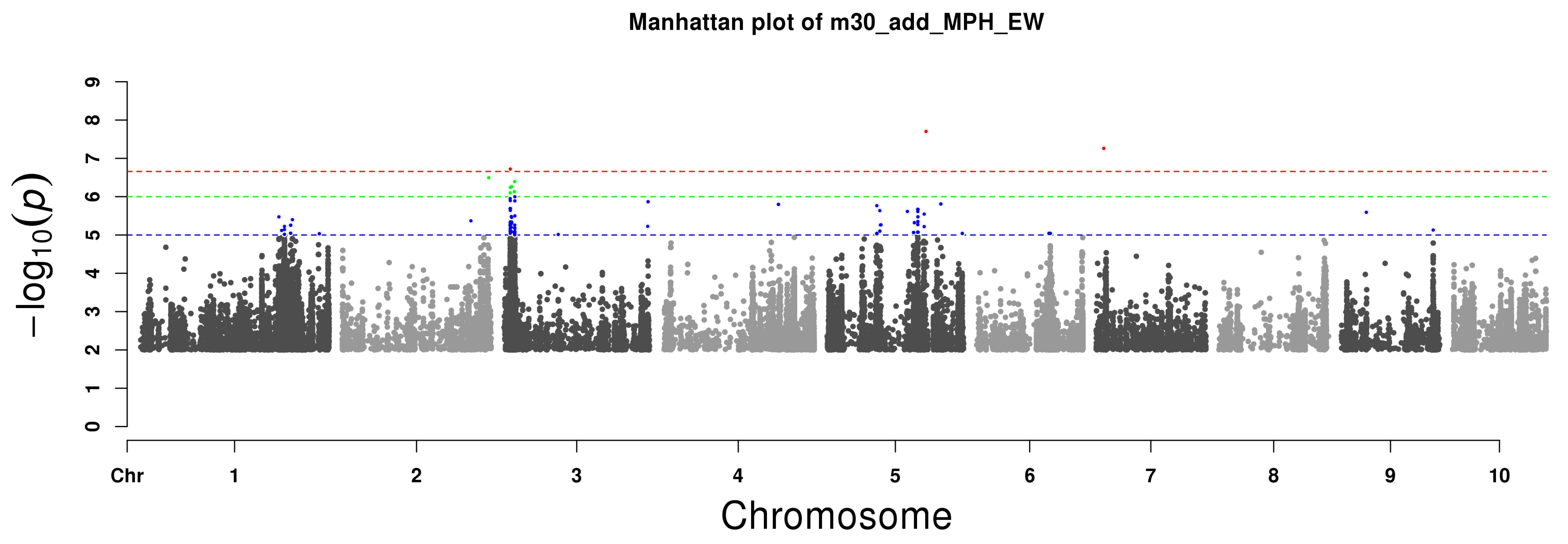
Manhattan plot of m30_add_MPH_EW
$-\log_{10}(p)$
0
1
2
3
4
5
6
7
8
9
Chr
1
2
3
4
5
6
7
8
9
10
Chromosome

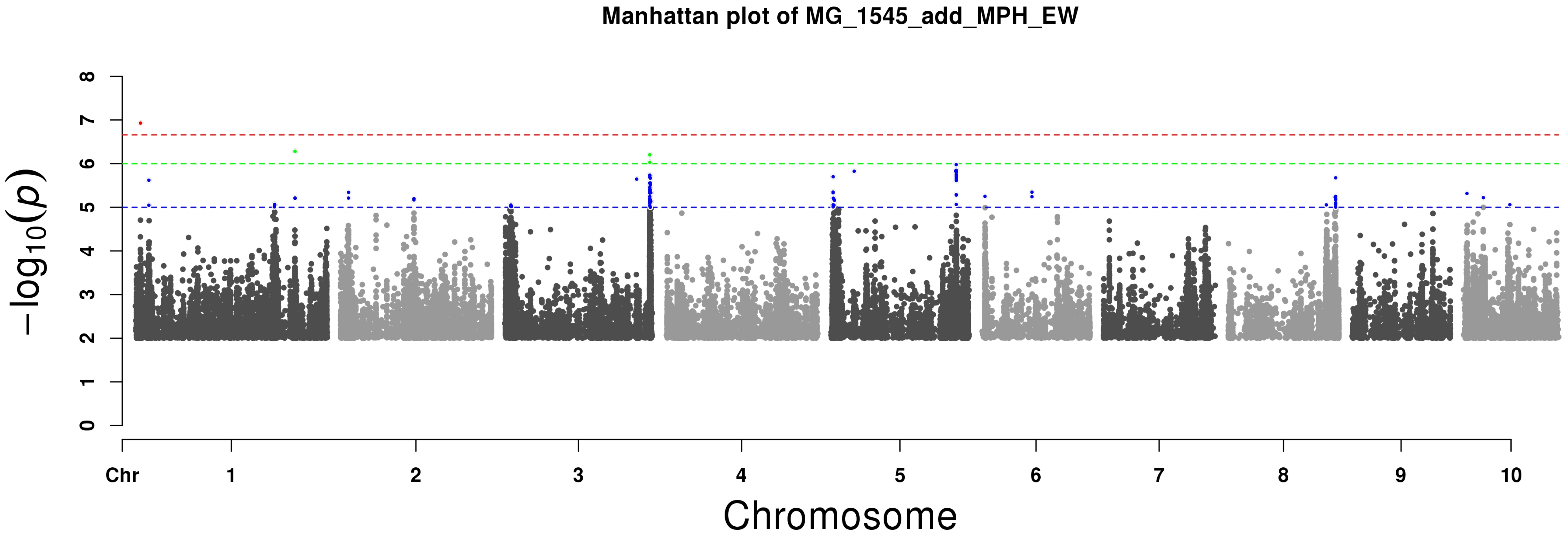

Manhattan plot of MG_1545_add_MPH_EW
$-\log_{10}(p)$
0
1
2
3
4
5
6
7
8
Chr
1
2
3
4
5
6
7
8
9
10
Chromosome
