# Supplementary material for: The genetic mechanism of heterosis utilization in maize improvement
Source: Genome Biol. 2021 May 10;22:148. doi: 10.1186/s13059-021-02370-7 (PMC8108465; doi:10.1186/s13059-021-02370-7)
Supplement: Supplementary file 7 — Additional file 7. Review history. [file 13059_2021_2370_MOESM7_ESM.docx]

Review History

**First round of review**

**Reviewer 1**

**Are you able to assess all statistics in the manuscript, including the appropriateness of statistical tests used?**

Yes, and I have assessed the statistics in my report.

**Comments to author:**

In this manuscript, the authors developed a new population by large-scale test crossing to analyze the genetic basis of maize heterosis. The authors selected 8,652 F1 hybrids to plant in the field for modeling, and predicted three phenotypes of the remaining hybrids. Moreover, by GWAS the authors also identified a total of 166 QTLs with additive, dominant or epistatic effects. Generally, this study provides a rich resource for hybrid maize breeding, and may explain part of the genetic basis of heterosis from a certain perspective.

Some concerns and suggestions for the work:

1. Based on the experimental design (F1s of 1,428 inbred lines with 30 parents), the proportion of three genotypes (AA, Aa and aa) at each locus and allelic combinations of multiple loci must be uneven, which strongly affect the evaluation of genetic effects and epistasis analysis. Any solutions, corrections or discussions?

2. The manuscript only released the raw reads of the parents. The genotypes and phenotypes in this manuscript, and codes for each step of statistical analysis should be provided for readers to make this experiment repeatable.

3. Were all 4.5million SNPs used to train the G2P model or some peak SNPs were selected? It is unclear for readers.

4. What is the form of the phenotypes predicted by the G2P model? Is the data form consistent with the observed phenotypes or normalized? If normalized, how was it converted into the form of observed phenotypes? More details should be added in the Method section.

5. Page9 Line196: "Fig. 2b; Additional file 3", it should be "Fig. 2a; Additional file 3".

**Reviewer 2**

**Are you able to assess all statistics in the manuscript, including the appropriateness of statistical tests used?**

Yes, and I have assessed the statistics in my report.

**Comments to author:**

In their manuscript, Xiao and colleagues explore the determinants of heterosis in a large panel of maize. The authors utilized 1,428 individuals as maternal lines from their previously published CUBIC population which consisted of 1,404 recombinant inbred lines and the 24 elite founder lines from which they were derived (these founders are primarily from two heterotic groups used in Chinese maize breeding). All of these lines were crossed to 2 paternal testers from China and a subset of 207 were crossed to an additional set of 28 elite and more diverse tester lines. All lines were genotyped and phenotypes were taken from the hybrids made through the crosses described above (n=8,652). Phenotypes for the remaining possible 34,188 hybrids were then predicted based on the subset of 8,652. The authors find good prediction accuracy in the hybrids that were not phenotyped directly and find that this process improves power for detecting QTL underlying agronomic traits. The authors also detect patterns of heterosis consistent with a strong prevalence of epistasis, with dominance and complementation of deleterious alleles being only a minor contributor. This is an impressive effort and represents an exceptional amount of work. However, I would encourage the authors to make a few modifications prior to publication.

1) The manuscript would benefit from a more clear and thorough description of the current hypotheses of the causes of heterosis and a summary of empirical support for these hypotheses. Dominance and complementation of deleterious alleles should be more fully described and overdominance should also be considered. An important missing citation is the Yang et al. 2017 paper in PLoS Genetics, titled, "Incomplete dominance of deleterious alleles contributes substantially to trait variation and heterosis in maize." These authors identify putatively deleterious alleles based on patterns of constraint across a multi-species alignment and show that including this information improves phenotypic prediction.

In the Discussion the authors need to better consider their findings on the prevalence of epistasis as causal in heterosis and carefully consider how and why their results differ from previous investigations such as the Yang et al. paper which found an important role for dominance and complementation.

2) The authors also need to be more careful in their consideration of what their particular population is best suited to detect as causal in heterosis. 1,404 recombinant inbred lines derived from 24 founders is an excellent resource for looking at epistatic interactions due to the shuffling of this small number of founder haplotypes across the genome. However, deleterious alleles in maize, because of their effects on fitness, are rare. With 24 founders of the CUBIC population contributing to the 1428 maternal lines and an additional 30 paternal testers, the authors have a limited sample of the rare, deleterious alleles that are likely contributing to heterosis achieved through complementation in modern maize. I would therefore encourage the authors to be more circumspect in their discussion and conclusions about the broad, global determinants of heterosis. I would encourage them to instead focus on the strength of their resource and experimental design in a targeted search for epistasis.

**Authors Response**

**Point-by-point responses to the reviewers’ comments:**

Reviewer comments:

Reviewer #1:

In this manuscript, the authors developed a new population by large-scale test crossing to analyze the genetic basis of maize heterosis. The authors selected 8,652 F1 hybrids to plant in the field for modeling, and predicted three phenotypes of the remaining hybrids. Moreover, by GWAS the authors also identified a total of 166 QTLs with additive, dominant or epistatic effects. Generally, this study provides a rich resource for hybrid maize breeding, and may explain part of the genetic basis of heterosis from a certain perspective.

*Reply: Thank you for the nice comments.*

1. Based on the experimental design (F1s of 1,428 inbred lines with 30 parents), the proportion of three genotypes (AA, Aa and aa) at each locus and allelic combinations of multiple loci must be uneven, which strongly affect the evaluation of genetic effects and epistasis analysis. Any solutions, corrections or discussions?

*Reply: We agree with the reviewer’s opinion. In the 42,840 F1 hybrids, the proportion of the three genotypes for most of the SNPs and allelic combinations of any two SNPs are uneven. To ensure the statistical power for GWAS and genomic prediction, the SNPs with low frequencies of minor alleles (MAFs) must be filtered. The original SNP set contains 14.8 million SNPs called from the resequencing of the 1,458 maternal lines, and almost 70% of them were filtered, resulting in 4.5 million high-quality SNPs with relatively high MAFs (at least 210 hybrids carrying the minor genotypes) for the subsequent analysis. Additionally, the large sample size further improved the statistical power of detecting QTLs, as reflected by the simulation analysis in Figure S5.*

*It’s inevitable that, uneven allelic combinations of pairwise SNPs may greatly underestimate the effects of epistasis or introduce large amount of false detection of epistasis, if using the traditional method of QTL interaction analysis that segregation of the two interacting loci in a population (e.g., an F2 population) must be a necessary requirement. To cope with this issue, we adopted an alternative strategy to detect epistasis taking the advantage of the current design, which was to compare the GWAS signals detected in the maternal population and the half-sibling F1 population. The basic rationale is that, if a QTL (QTL-A) was undetected in the maternal population but detected in the F1 populations, this QTL-A must be epistatically controlled as its effect in the maternal population might be epistatically repressed by the recessive alleles of an alternative QTL (QTL-X) that is not identified. In F1 hybrids, the paternal genome contributes a dominant allele of QTL-X, resulting the relief of its repressing effect on QTL-A. Therefore, the unique design of the half-sibling F1 populations offered a new avenue to uncover epistatically-controlled QTLs and interpret the role of epistasis in heterosis formation (Figure 4). It is also one of the advantages of present design which has been well discussed in the revised manuscript.*

2. The manuscript only released the raw reads of the parents. The genotypes and phenotypes in this manuscript, and codes for each step of statistical analysis should be provided for readers to make this experiment repeatable.

*Reply: We followed the reviewer’s suggestion, and uploaded the genotypic data (4.5 million SNPs), phenotypic data of the 1,458 parental lines and the 42,840 F1 hybrids to the ZEAMAP database, so that the data may be freely obtained via the link http://zeamap.hzau.edu.cn/ftp/99_MaizegoResources/01_CUBIC_related/. The scripts and pipelines used in this work, including phenotypic data normalization, BLUP-correction of phenotypes across multiple environments, genomic prediction, data simulation for GWAS power, were all publicly accessible at GitHub address (https://github.com/yingjiexiao/maizeG2P). The above information was included in the “Availability of data and materials” section in the revised manuscript.*

3. Were all 4.5 million SNPs used to train the G2P model or some peak SNPs were selected? It is unclear for readers.

*Reply:* We apologized for the unclear description of G2P analysis. To train the G2P model, we used all of the 4.5 million SNPs as input to derive a centered genomic relationship matrix (GRM) for phenotype prediction. We did not use the peak SNPs obtained from the GWAS analysis of training population, as utilization of peak SNPs may cause severe overfitting of the G2P model. We added detailed description of G2P prediction in the Methods.

4. What is the form of the phenotypes predicted by the G2P model? Is the data form consistent with the observed phenotypes or normalized? If normalized, how was it converted into the form of observed phenotypes? More details should be added in the Method section.

*Reply: The observed phenotypes of the training samples were normalized by the z-score algorithm to remove the system bias occurred among the thirty sets of F1 populations. Thus, the phenotypes directly predicted by the G2P model were also in the form of z-score values. To convert the z-score form of predicted phenotypes to absolute values, the following algorithm was applied within each of the thirty F1 populations: y_i=z_i×sd(y)+y ̅, where z_i is the z-score form of the predicted phenotypes of F1 hybrid i in the predicting population; y ̅ and sd(y) are the mean and standard deviation of the phenotypes of training samples, respectively. Conversion of the z-score form to absolute form of predicted phenotypes were added in the Methods.*

5. Page9 Line196: "Fig. 2b; Additional file 3", it should be "Fig. 2a; Additional file 3".

*Reply: This mistake was corrected in the revised manuscript.*

Reviewer #2:

In their manuscript, Xiao and colleagues explore the determinants of heterosis in a large panel of maize. The authors utilized 1,428 individuals as maternal lines from their previously published CUBIC population which consisted of 1,404 recombinant inbred lines and the 24 elite founder lines from which they were derived (these founders are primarily from two heterotic groups used in Chinese maize breeding). All of these lines were crossed to 2 paternal testers from China and a subset of 207 were crossed to an additional set of 28 elite and more diverse tester lines. All lines were genotyped and phenotypes were taken from the hybrids made through the crosses described above (n=8,652). Phenotypes for the remaining possible 34,188 hybrids were then predicted based on the subset of 8,652. The authors find good prediction accuracy in the hybrids that were not phenotyped directly and find that this process improves power for detecting QTL underlying agronomic traits. The authors also detect patterns of heterosis consistent with a strong prevalence of epistasis, with dominance and complementation of deleterious alleles being only a minor contributor. This is an impressive effort and represents an exceptional amount of work. However, I would encourage the authors to make a few modifications prior to publication.

*Reply: Thank you for the nice comments.*

1. The manuscript would benefit from a clearer and thorough description of the current hypotheses of the causes of heterosis and a summary of empirical support for these hypotheses. Dominance and complementation of deleterious alleles should be more fully described and overdominance should also be considered. An important missing citation is the Yang et al. 2017 paper in PLoS Genetics, titled, "Incomplete dominance of deleterious alleles contributes substantially to trait variation and heterosis in maize." These authors identify putatively deleterious alleles based on patterns of constraint across a multi-species alignment and show that including this information improves phenotypic prediction.

In the Discussion the authors need to better consider their findings on the prevalence of epistasis as causal in heterosis and carefully consider how and why their results differ from previous investigations such as the Yang et al. paper which found an important role for dominance and complementation.

*Reply: We apologize for missing one of the important papers in heterosis study, the one by Yang et al. 2017 that discovered the important contribution of incomplete dominance of deleterious alleles to heterosis. We also appreciate the reviewer’s suggestion that the Discussion part needs to further explain the difference of the results concluded from our study and Yang et al.’s. Below is a short summary of the explanations that have been added to the revised version of the manuscript.*

*First, during the process of constructing the 1,404 CUBIC lines in six generations of open pollinations and six generations of self-pollinations, selection of lines were based on the agronomic performances of lines per se. Lines with field performances above average were selected for hybridization in the next generation. Therefore, the deleterious alleles with large effects negative to fitness might have been eliminated, as mentioned by the reviewer in the second comment, maintaining many slightly deleterious mutations. On the other hand, we agree with the reviewer’s opinion provided below that deleterious allele in maize are often rare, the effective population size in our study may be limited to detect such rare, modest-effect mutations. That may be the reason why our study directly detected few dominance QTLs through GWAS with very strict significant threshold, although the overall contribution of dominance complementation of deleterious alleles to heterosis can be observed in the Yang et al. paper.*

*Second, detection of fewer dominant QTLs than epistatically-controlled QTLs doesn’t mean that, dominance is less important than epistasis in terms of the contribution to heterosis. In our design, we propose the dominant complementation of deleterious allele in F1 hybrids may release its repressive influence on the detection of downstream QTLs, that were defined as the epistatically-controlled QTL. Thus, given the prevalence of epistatically-controlled QTL in our data, it may instead support that dominance complementation is a crucial factor determining heterosis by activating more downstream interactions.*

*In brief, our findings are not contradictory to the results from Yang et al. Instead, we support the important role of dominance complementation and may help explaining the co-contribution of dominant QTLs and epistatically-controlled QTLs to heterosis.*

2. The authors also need to be more careful in their consideration of what their particular population is best suited to detect as causal in heterosis. 1,404 recombinant inbred lines derived from 24 founders is an excellent resource for looking at epistatic interactions due to the shuffling of this small number of founder haplotypes across the genome. However, deleterious alleles in maize, because of their effects on fitness, are rare. With 24 founders of the CUBIC population contributing to the 1428 maternal lines and an additional 30 paternal testers, the authors have a limited sample of the rare, deleterious alleles that are likely contributing to heterosis achieved through complementation in modern maize. I would therefore encourage the authors to be more circumspect in their discussion and conclusions about the broad, global determinants of heterosis. I would encourage them to instead focus on the strength of their resource and experimental design in a targeted search for epistasis.

*Reply: We appreciated the reviewer’s precious advice on improving our manuscript and have made significant modifications on the discussion part to explain the difference and commonality discovered by our study and the paper by Yang et al. In the new version of the manuscript, we have not only emphasized the value of our data as an important genetic resource to discover epistasis, but also explained the correlation of dominant QTLs and epistatically-controlled QTL. That is, epistasis is a consequence of dominance complementation of deleterious alleles in the hybrid genome.*

**Second round of review**

**Reviewer 1**

The revision has well addressed my concerns. Very nice work!
